# GWAS of Post-Orthodontic Aggressive External Apical Root Resorption Identified Multiple Putative Loci at X-Y Chromosomes

**DOI:** 10.3390/jpm10040169

**Published:** 2020-10-14

**Authors:** Paula Iber-Díaz, Raquel Senen-Carramolino, Alejandro Iglesias-Linares, Pablo Fernández-Navarro, Carlos Flores-Mir, Rosa M Yañez-Vico

**Affiliations:** 1 Section of Orthodontics, School of Dentistry, Complutense University, 28040 Madrid, Spain; piber@ucm.es (P.I.-D.); senenbla@ucm.es (R.S.-C.); rosayane@ucm.es (R.M.Y.-V.); 2 BIOCRAN Craniofacial Biology Research Group, Complutense University, 28040 Madrid, Spain; 3 Cancer and Environmental Epidemiology Unit, National Center for Epidemiology, Carlos III Institute of Health, 28029 Madrid, Spain; pfernandezn@isciii.es; 4 Consortium for Biomedical Research in Epidemiology and Public Health (CIBERESP), 28029 Madrid, Spain; 5 Professor and Interim Graduate Orthodontic Program Director, School of Dentistry, University of Alberta, Edmonton, AB T6G 1C9, Canada; cf1@ualberta.ca

**Keywords:** orthodontics, dentistry, dental trauma, resorption, fixed appliances

## Abstract

Personalized dental medicine requires from precise and customized genomic diagnostic. To conduct an association analysis over multiple putative loci and genes located at chromosomes 2, 4, 8, 12, 18, X, and Y, potentially implicated in an extreme type of external apical root resorption secondary to orthodontic forces (aEARR). A genome-wide association study of aEARR was conducted with 480 patients [ratio~1:3 case/control]. Genomic DNA was extracted and analyzed using the high-throughput Axiom platform with the GeneTitan^®^ MC Instrument. Up to 14,377 single nucleotide polymorphisms (SNPs) were selected at candidate regions and clinical/diagnostic data were recorded. A descriptive analysis of the data along with a backward conditional binary logistic regression was used to calculate odds ratios, with 95% confidence intervals [*p* < 0.05]. To select the best SNP candidates, a logistic regression model was fitted assuming a log-additive genetic model using R software [*p* < 0.0001]. In this sample the top lead genetic variants associated with aEARR were two novel putative genes located in the X chromosome, specifically, STAG 2 gene, rs151184635 and RP1-30E17.2 gene, rs55839915. These variants were found to be associated with an increased risk of aEARR, particularly restricted to men [OR: 6.09; 95%CI: 2.6–14.23 and OR: 6.86; 95%CI: 2.65–17.81, respectively]. Marginal associations were found at previously studied variants such as *SSP1*: rs11730582 [OR: 0.54; 95%CI: 0.34–0.86; *p* = 0.008], *P2RX7*: rs1718119 [OR: 0.6; 95%CI: 0.36–1.01; *p* = 0.047], and *TNFRSF11A*: rs8086340 [OR: 0.6; 95%CI: 0.38–0.95; *p* = 0.024]), found solely in females. Multiple putative genetic variants located at chromosomes X and Y are potentially implicated in an extreme phenotype of aEARR. A gender-linked association was noted.

## 1. Introduction

External apical root resorption (EARR) resulting from orthodontic forces represents one of the most undesirable iatrogenic effects secondary to mechanical strain during orthodontic movement, provoking an irreversible loss of root structure and tooth attachment in the apical third of the tooth root [1,2]. EARR is mostly manifested in its mild to moderate forms [3,4,5]; however, the most aggressive phenotype, with a frequency <1–5% and >5 mm apical loss, might critically compromise tooth viability [6,7].

EARR of any degree represents a complex pathological trait with multilevel etiological-risk factors that have not been completely elucidated to date [8]. Several diagnostic and clinical factors have been associated with EARR [9]. Several factors, such as treatment time, apical displacement, and gender-specific risk have been found to be associated with EARR, but these findings systematically show some degree of inconsistency and controversy in literature [10,11,12]. In fact, EARR occurrence and severity remains unpredictable and is not fully explained by clinical evidence alone. In this context, a genetic component and its contribution to this pathological feature, has been a critical issue that has recently garnered considerable attention [13,14]. Particularly, risk loci in somatic chromosomes have been targeted extensively, whereas very few studies are available regarding regions potentially associated with EARR at sexual chromosomes [15]. To date, a limited number of candidate gene association studies [15,16,17,18,19,20,21,22,23,24] have provided evidence suggesting that some genetic variants might exert a positive or negative influence over EARR of a mild to moderate degree at the level of the *IL1* gene cluster [15,20,21,22,23,24], *TNFRSF11B* [25], *P2RX7* [17,18], *SSP1* [19,23], or *TNFRSF11A* [15,26] at an autosomic level but also at *IRAK1* gene [27] on sexual chromosome X. Despite the above mentioned studies, there is no available scientific evidence regarding how genetic factors might be specifically associated with the most severe phenotype of EARR, i.e., aggressive EARR (aEARR). Moreover, whether aEARR is positively/negatively associated with previous genetic variants and the studied clinical/diagnostic factors remains to be elucidated.

Therefore, the present study aimed to perform the first genome-wide association study conducting an association analysis over multiple putative loci and genes located at somatic chromosomes 2, 4, 8, 12, 18, and at sexual chromosomes X and Y, potentially implicated in aEARR.

## 2. Materials and Methods

### 2.1. Study Design

**Figure 1:**
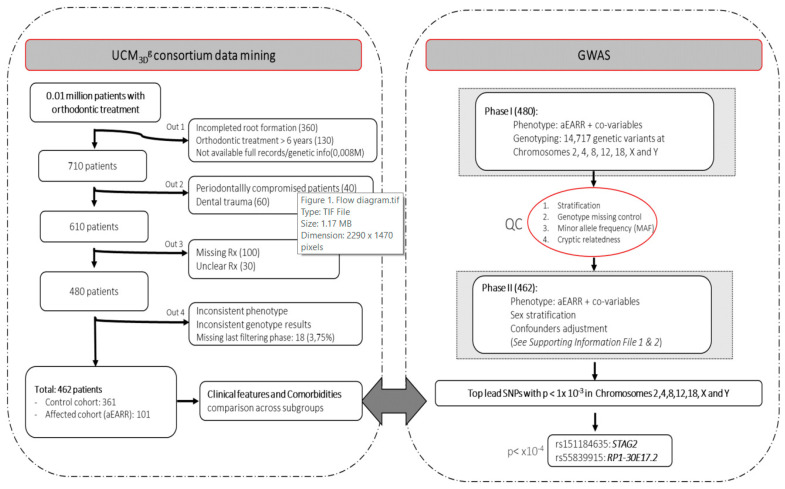
Flow diagram of the filtering process and genome-wide association study.

We performed a genome-wide association study of aEARR, a derived extreme and well-delimited root resorption phenotype, in UCM_3D_^g^ consortium participants (Figure 1). The UCM_3D_^g^ consortium database is based on a general-population cohort of roughly 0.01 million patients aged 9–67 years old, recruited across Spain between 2005 and 2019.

### 2.2. Sample Size, Study Cohorts, and Ethics Statement

#### 2.2.1. Sample Size Calculation

Sample size estimation was based on the minimum sample size required for a genetic association study based on: (a) the prevalence of the disease: 0.01; (b) ratio of cases to controls (~1:3); (c) alpha error 1 × 10^−4^/beta error: <0.20; (d) base relative risk: 2.9; and (f) frequency of risk allele: 0.25. Sample size was calculated using the freely available Genetic Power Calculation software [28]. It was determined that 450 patients would be needed to establish an association between the presence of a single nucleotide polymorphism (SNP) of interest under these conditions and the appearance of an advanced state of EARR, 100 cases for the aggressively affected cohort and 350 controls, with 3% overestimation to include expected dropouts were required.

#### 2.2.2. Cohort Distribution and Ethics Statement

The study population comprised 480 patients [*ratio ~1:3; case:control*] with available radiographic, diagnostic, and clinical records from the UCM_3D_^g^ consortium database, who were eligible for participation in the present study and met the inclusion criteria detailed in *Supporting Information File 1*. Up to 4.8% of the eligible UCM_3D_^g^ consortium patients with available diagnostic, clinical, and radiographic records were enrolled with the complete available genetic data used in the present study. We were granted approval from the Institutional Ethical Review Board of the Clinical Hospital San Carlos, Madrid (IRB) [ref#:17/038-E] and permission was previously obtained from each individual to participate in the present study, allowing their clinical, diagnostic, radiographic, and genomic data to be used for health-related research as part of this study. This study was carried out in accordance with the ethical principles governing medical research and human subjects, as laid down in the Helsinki Declaration (Helsinki Declaration 2013 version. Available online: https://jamanetwork.com/journals/jama/fullarticle/1760318 (accessed on 3 March 2017),). Details of the consenting process are described elsewhere [29].

### 2.3. Knowledge Discovery in UCM_3D_^g^ and SALUD^R^ Databases: Data Mining

#### Quality Control (QC), Data Filtering, and Extraction

A pre-piloted protocol was followed for data filtering and extraction from the UCM_3D_^g^ and SALUD^R^ databases. As detailed in *Supporting Information File 2*, the diagnostic variables, clinical setting variables, and radiological values were collected and subjected to QC (accession registered URI+i *ref#:5-201119*). Manifest-codifying data errors in the database were removed by selecting and identifying implausible values in each category of diagnostic, clinical, or radiological variables (e.g., Discrepancy index (DI) = 400) or others that were not adequately recorded in terms of the type of unit or quantitative/categorical format (e.g., age = a). Extreme values of duration of mechanical loading (treatment time > 72 months) justified elimination as they were more likely to be data errors or non-physiological extremes rather than feasible variable inputs. The quality-checked data were processed in the next steps (Figure 1).

### 2.4. Phenotyping and Radiographic Measurements

All subjects selected for final inclusion in the study were assigned to the affected or control cohorts of patients according to radiological screening, using radiographic measurements performed in duplicate and in a double-blinded (P.I. and R.S) manner on orthopantomographic and teleradiographic projections that were already available and used at the clinic for routine diagnosis and treatment. Subjects were classified and divided into these two groups, based on the presence or absence of the phenotype of aggressive post-orthodontic EARR of more than 5 mm in blinded radiographic measurements. The affected cohort included patients with severe EARR > 5 mm and the control group was composed of patients with EARR < 5 mm [7].

The following methods have been detailed previously; an aEARR phenotype was assessed after the roots were measured from before and after treatment on panoramic radiographs focusing on the maxillary central and lateral incisors [4,5,15]. All pre- and post-treatment images were calibrated beforehand and a correction factor for magnification was applied in all cases. Measurements were performed on digital radiographs using diagnostic software (Adobe Photoshop CS8, Adobe Systems Incorporated, San Jose, CA, USA) enabling the image filters to provide maximum precision when localizing the terminal points of the roots. Accordingly, the tooth with the highest EARR value was selected as the dependent variable of interest for that subject using the method described by Linge and Linge [30], modified by Brezniak et al., (2004) [31]. Pre- and post-treatment radiographs through the initial and final root (r1 and r2, respectively) and crown (c1 and c2, respectively) lengths were used to determine the changes in dental and root length. If the root became shorter during treatment, the EARR value resulted from r1–r2 [c1/c2]).

Apical displacement and variation in tooth inclination were quantified using the superimposition of radiographic measurements on a lateral radiograph, using a modified version of the method described by Baccetti et al., (1998) [32].

### 2.5. Genotyping

#### DNA Extraction, Genotyping

Genomic DNA was extracted from saliva according to the manufacturer’s instructions (prepIT•L2P, DNA Genotek, Ottawa, ON, Canada). Total genomic DNA was checked for purity and integrity [OD_260_/OD_280_: 1.8–2.0; OD_260_/OD_230_ > 1.5; 1% agarose gel integrity: 90% DNA size > 10Kb]. Quality control was then performed per sample using the *Agena Bioscience MassARRAY plattform iPLEX GOLD technology* to eliminate samples with poor quality.

DNA samples were genotyped using the high-throughput *Axiom* platform with the GeneTitan^®^ MC Instrument *(Axiom Genome-Wide Human Assay technology, CeGen)*. This method has been extensively validated in literature [33,34].

Data were analyzed, using the Axiom Analysis Suite 4.0 software for genotype clustering and calling. We then implemented a quality control for the data, where we checked possible sample stratification, missing SNP genotype, SNP monomorphic status, and SNP minor allele frequency (*see Supporting Information File 3*) from 687,133 markers. Next, to achieve our objectives, we selected 14,377 SNPs located in the X and Y-chromosomes along with other candidate genes at chromosomes 2, 4, 8, 12, and 18 for the analysis (*see Supporting Information File 4*).

### 2.6. Statistics

#### 2.6.1. Overall Statistical Analysis of Clinical and Radiological Variables

A descriptive analysis of the data for quantitative and categorical variables based on diagnostic or treatment factors was performed (mean, SD, ranges, frequencies, and distributions). Backward conditional binary logistic regression was used to assess the extent to which specific diagnostic and treatment parameters interfere within the observed aEARR process; odds ratios (OR) with a 95% confidence interval were also calculated. SPSS was used for data analysis (version 22.0; LEAD Technologies, Chicago, IL, USA), with statistical significance set at a value of *p* < 0.05 [35].

#### 2.6.2. Genetic Association Tests

To select our best SNP candidates, we fitted a logistic regression model adjusted by the type of treatment, total length of treatment (load duration), and gender, and assumed a log-additive genetic model. These statistical analyses were performed using R software (R Core Team, Vienna, Austria. version 3.6.1) [35].

#### 2.6.3. Reliability and Accuracy of the Measurement Method

To prevent inter-observer variation, the same experienced operator (R.S) carried out all the measurements defined previously. However, a second experienced examiner (P.I) replicated the measurements on a subset of 50 patients to calculate the inter-observer error. The *kappa* coefficient was analyzed to determine concordance between EARR-affected and non-affected group assignment, based on radiological screening, and the result was a value of one. The method error was also calculated for measurements acquired from radiographic records, comparing double measurements of 20 randomly chosen subjects at an interval of 20 days. A paired Student’s *t*-test was used for calculations, with an absence of significance being regarded as indicative of concordance between repeated measurements; the intraclass correlation coefficient (ICC) for absolute agreement was also calculated for both intra- and inter-observer errors. Accuracy of measurement was obtained from the equation:

SE = √(Σd2/2n)

where d is the difference between the double measurements and n is the number of paired double measurements [36].

## 3. Results

### 3.1. Phenotyping: Reliability of the Radiographic Measurement Methods and the Associated Errors

aEARR was phenotyped according to radiographic measurements with a threshold for root resorption value greater than 5 mm. Reliability of the measurements retrieved no statistically significant differences between replicated assessments (*p* > 0.05) and intraclass correlation coefficient (ICC: 0.930), and the concordance index resulted in optimal values (*k =* 1.00) for both intra- and inter-examiner measurements, respectively [37]. Method error for measurements obtained from panoramic radiographs following the described method was calculated to be below <0.04 mm.

### 3.2. Sample Characteristics, Description and Analysis of aEARR Risk Associated with Clinical Features

The flow chart diagram (Figure 1) describes the sampling filtering steps and the following research strategy. The present study sample included a cohort of patients with relatively homogenous diagnostic characteristics, who had undergone mechanical load during a full orthodontic treatment, as detailed in Table 1. The mean ages of patients treated in the affected and control cohorts were ~21 ± 12 and ~23 ± 12 years old, respectively, with a fair balance found for sex distribution. The American Board of Orthodontics (ABO) discrepancy index was found to be quite homogenous in both groups [~16 ± 9 and ~15 ± 8] with a mean treatment time of ~27 ± 9 and ~25 ± 8 months, respectively (Table 1). Results from the associative testing by regression analysis are detailed in Table 1. The results showed that recorded clinical factors do not confer an additional risk of aEARR when compared to the control cohort. Specifically, differences in treatment time [OR: 0.974; 95%CI: 0.944–1.005; *p* = 0.095] or treatment type, extraction vs. non-extraction [OR: 1.668; 95%CI: 0.839–3.316; *p* = 0.145], did not confer an additional risk for aEARR process when results from both the study cohorts in the regression analysis were compared.

### 3.3. Genotype Distributions and Analysis of aEARR Risk Associated with Genetic Variants at Multiple Putative Loci on Chromosomes 2, 4, 8, 12, 18, X, and Y.

In addition to diagnostic and treatment-related co-variables, patients included in the present study were genotyped for specific novel target genetic variants and other SNPs explored in previous studies with regard to a risk of any degree of EARR found along the genome in chromosomes 2, 4, 8, 12, 18, X, and Y (*see Supporting Information File 3*).

When the whole cohort sample was explored within the associated risk for aEARR, a gender-dependent effect was detected to influence the results. The top lead genetic variants associated with aEARR [*p* value < 1 × 10^−4^], after gender stratification, focused on two novel putative genes located in the X chromosome, specifically, *STAG 2* gene, stromal antigen 2 gene, rs151184635 (*prior* to and after adjustment for confounding factors) and *RP1-30E17.2*, clone-based (Vega) gene rs55839915 (after adjustment for confounding factors). These two target variants were found to be associated with an increased risk of aEARR; this effect was particularly restricted to men [OR: 6.09; 95% CI: 2.6–14.23 and OR: 6.86; 95% CI: 2.65–17.81, respectively] (Table 2 and Table 3).

In addition to these two top genetic variants (as compiled in Table 2 and Table 3), a total number of 27 novel genetic variants out of 14,717 [*p* value < 0.001] were identified with marginal association values, specifically in the sexual chromosomes; some of them were associated particularly with an independent susceptibility risk of experiencing aEARR in men or women secondary to mechanical orthodontic load. None of the previously studied genetic variants located at chromosomes 2, 4, 8, 12, and 18, also included in the present study, were found to be marginally associated with a positive or negative risk of aEARR [*p* value > 0.001]. In this respect, when a *p* value threshold was set to a conventional value of <0.05, the number of potential associations increased substantially as provided in *Supporting Information File 5*. In this line, previously studied variants at chromosomes 4, 12, and 18, i.e., *SSP1*: rs11730582 [OR: 0.54; 95%CI: 0.34–0.86; *p* = 0.008], *P2RX7*: rs1718119 [OR: 0.6; 95%CI: 0.36–1.01; *p* = 0.047], and *TNFRSF11A*: rs8086340 [OR: 0.6; 95%CI: 0.38–0.95; *p* = 0.024], respectively, showed marginal associations that interestingly, were found only in females, not showing the same trend for males. Importantly, all the above described marginal associations and their effects should be interpreted with caution as adjustment for multiple comparisons retrieves false discovery rate (FDR) values superior to 0.05 for all genetic targets, which does not yet allow precise discarding of the variants as not having an unequivocal modulatory effect over aEARR (Table 2 and Table 3, *Supporting Information File 5*).

## 4. Discussion

The present study offers, for the first time, significant valuable data on genetic variants located on chromosomes X and Y that are potentially implicated in aEARR facilitated by orthodontic forces. aEARR has been defined as a permanent loss of apical dental root structure of more than 5 mm. Severe forms of EARR secondary to orthodontic forces are the least frequent type of EARR, which is more often detected as of mild or moderate degrees [7]. Thus, aEARR describes a clearly radiographically identifiable phenotype of EARR with an apical third loss of more than 5 mm that is produced within a limited period, as this is the case of a mean orthodontic treatment length of ~20 months [38]. Despite being relatively rare, aggressive phenotypes are prone to exacerbate patient morbidity and are more likely to provoke mechanical and functional disabling consequences for the tooth, potentially inducing irreversible pulp or periodontal damage and triggering an inflammatory and/or infectious process that might result in eventual tooth loss [6,39].

Therefore, from a clinical perspective, it is clearly urgent to suitably define the triggering factors that might contribute to the etiology of this complex aggressive pathology [7]. In line with this, several diagnostic and clinical factors have been previously associated with moderate degrees of EARR secondary to orthodontic forces [2,40]. Demographic factors such as age and gender, morphological factors such as root shape type or clinical factors such as treatment duration, magnitude of orthodontic forces, previous dental trauma, maxillary expansion degree, direction of tooth movement, extraction treatment, use of intermaxillary elastics, or even appliance type have been suggested [9,41,42,43,44]. Nevertheless, a significant number of controversial related results are also found in literature. Recent meta-analysis and systematic reviews have suggested a greater relevance to some of them, but these are usually based on scientific evidence with low certainty levels [9,45]. Hence, several differing clinical management protocols for aEARR have been suggested [1].

Regression analysis performed in the present study suggests that both cohorts are unlikely to be marginally influenced (*p* > 0.05) by some of the collected covariables. In this regard, the treatment time has been described as a risk factor influencing EARR severity within a degree-time dependent effect [44]; however, this suggestion is not supported for the case of aEARR based on the present research data. A robust risk association was not directly imputed to treatment length by itself according to comparisons between both cohorts in the current study [44,45,46]. It is thus plausible that some confounding factors associated with treatment length and not treatment time itself, might explain some of the differences in some cases [38,47]. In this respect, some of the lengthiest treatments are very often associated with non-conventional tooth-movement rates or uncharacteristic treatment mechanics, which might be linked with infra-diagnosed conditions or factors, i.e., this may be the case for basal differences in bone remodeling metabolism and rates, and also for differences in craniofacial muscular responses which might affect occlusal loading and even tooth micro-trauma during orthodontic treatment and may even interfere with medications such as AINES and bisphosphonates [48,49,50]. Although a meticulous medical history was recorded; no unquestionable certainty should be presumed from the patients’ responses. Further non-compliance, round tripping movements, and movements near the bone cortex might also be associated with lower orthodontic tooth movement rates and/or extended treatment times [45,51]. Even considering these reflections, heterogeneity between study designs, ethnic differences, and the differences within phenotypic characterization might contribute to some of the observed results [17,23,52,53].

Apart from clinical-related factors, accumulating evidence supports the influence that genetic factors exert over the occurrence of post-orthodontic EARR with moderate severity. Previously published studies have provided useful evidence regarding the suggestive role of some specific genetic variants within the EARR process following candidate gene approaches [15,16,17,18,19,20,21,22,23]. In this respect, none of the previously studied genetic variants, also examined in the current study, showed robust statistically significant associations with aEARR [*p* > 0.0001], while a few of them (*SSP1*: rs11730582, *P2RX7*: rs1718119, *TNFRSF11A*: rs8086340) showed marginal associations [*p* < 0.05]. Interestingly, these marginal associations were found just in the female group, not showing the same trend in males, prior to and after adjustment with the clinical confounders. This might suggest a gender-specific effect. In this respect, this is the first study to perform a deep analysis regarding the influence of more than 14,000 specific genetic variants located on sexual chromosomes within an aggressive phenotype of EARR in the context of mechanical orthodontic loading. Moreover, this paper offers very valuable data regarding multiple novel putative loci and the genes potentially implicated in this type of extreme phenotype located not chromosomes X and Y, as well as at the level of other candidate genes with less power imputation located at autosomes 2, 4, 8, 12, and 18. Specifically, just two variants located at chromosome X, STAG 2 rs151184635 and RP1-30E17.2 rs55839915, where identified as the best associated SNPs [*p* value < 0.0001]. Interestingly, these associations showed a gender-dependent association limited to men [54,55]. We detected several statistically significant sex-specific interactions for many SNPs in the targeted genes. This sexual dimorphism is present in a vast majority of human pathologies based on genetic and hormonal differences that might modulate gene expression increase or minimize disease risk and progression [56,57,58,59]. In this regard, differences in bone remodeling rate have been described to be highly influenced by sex-specific features along with potential differences in bone mineral density and metabolism, or even hormone balance status [48,51,60]. Moreover, other gene expression factors directly linked to cytotoxic protection mechanism or related cytokine secretion might be underlying in the sex-specific differences as in other pathological entities [61,62,63]. Future studies should thus clarify if these results imply a potential interaction of these candidate genes with specific pathways related to gonadal steroids or additional gender-dependent mediators [64].

The top identified associations in the current study targeted rs151184635 and rs55839915 variants. RP1-30E17.2 [Ensembl: ENSG00000225689.1] contains 216,374 genetic variants with at least four splice variants. The SNP rs55839915 associated in the present study overlaps in three different transcripts being a long intergenic non-coding RNA type (lincRNA) that lacks coding potential [65]; however, lncRNAs have been described as regulators of gene expression, scaffold formation, and epigenetic control mediating within different pathological complex entities and physiological processes [66,67]. No specific underlying mechanisms have been described associated with this variant, and so, further studies should provide some potential explanatory hypothesis for aEARR. Meanwhile, STAG 2 encodes stromal antigen 2 protein, a subunit of the cohesin complex [Ensembl: ENSG00000101972 MIM: 300826]. This gene is linked to separation of chromatids during cell division, and its inactivation is associated with several types of human cancer [68]. The rs151184635 variant codes a non-coding transcript variant occurring within an intron overlapping three different transcripts at STAG2, TEX13D, and SH2D1A, all long non-coding RNA genes [69,70,71,72]. Although no direct robust functional consequence has been described in literature, SH2D1A, which is a mediator in cytolytic pathways as key activator of T- and NK cell-cytotoxicity, showed differential gene expression associated with specific immunopathologies as is the case of systemic juvenile idiopathic arthritis, associated with the potential onset of macrophage activation syndrome [73]. In this regard, extremely high overproduction of pro-inflammatory cytokines, IFN-γ, IL-1, IL-18, TNF, IL-2, IL-6, and macrophage colony-stimulating factor (M-CSF), as well as repressors such as circulating TNF receptors and IL-1ra are correlated with this macrophage-based pathology [74]. In connection with this, it has been described that prolonged mechanical strain within the periodontal ligament (PDL) around the tooth root is associated with an increase in CD68+, iNOS+ M1-like macrophages, an imbalance in the M1 > M2 ratio polarization and root resorption pathology linked to IFN-γ oversecretion by T-cells and PDL stem cells [75,76]. Whether any potential functional consequence in terms of alternative splicing or gene expression modulation might have a direct/indirect effect on the onset of aggressive phenotype of EARR, remains to be a remote hypothesis that should be explored deeply based on robust future molecular studies. Interestingly, X-chromosome activation of the vast majority of genes linked to the X chromosomes occurs in one of the two X chromosomes of any cell in women. Random or quasi-random selection of which X chromosome will remain inactivated in females follows a different process in males, which might lead to differential expression or repression of some inherited X-linked genes. This raises the possibility that expression of specific long non-coding RNAs, as should be the case for STAG2, TEX13D, or SH2D1A, might exert an influence in modulating gene expression in specific domains that escape X silencing, as occurs in other species, in a gender-dependent way. This should partially explain whether the genetic variants (rs151184635 and rs55839915) in the present study are at least marginally associated in males but not in females [77,78,79].

### Limitations

The present study recruited a representative cohort of severely EARR affected patients that were radiographically screened by means of panoramic projections measurements >5 mm as assessed by two experienced examiners [7]. The radiographic diagnostic method used for patient assignment to affected and control groups is not exempt from limitations in terms of accuracy compared to other x-ray methods such periapical radiographs, Cone Beam Computerized Tomography (CBCT), Computerized Tomography (CT), or ex vivo methods (histological and/or histochemical) [80]. Nevertheless, panoramic radiograph assessments might produce sufficient reliability to perform absolute linear measurements when the head position is controlled in a range of 10° of the inclination in regard to the horizontal plane. Moreover, panoramic radiographs have been associated with underestimation of the tooth root lesion in mild lesions; however, this type of screening for assessing such types of aggressive phenotypes (>5 mm) is less prone to misclassification as shown by optimal reproducibility and error method results retrieved by the two experienced operators in the present study.

Secondly, although the UCM3Dg consortium database provides a unique opportunity to investigate the underlying influence of genetics over aEARR, the sample size is still relatively moderate, once the obtained results are re-analyzed, and the present design did not use an independent external cohort as a replication study due to the particularity of this aggressive phenotype. Thus, spurious associations remain a challenge and the external validity of the present findings need to be confirmed in further investigations by using different external cohorts to test the model.

Lastly, differences observed between study results in terms of the diagnostic, clinical, and genetic factors associated with EARR of different degrees are a major concern that should be addressed. With respect to this, it seems plausible that the heterogeneity found between study designs, ethnic differences [52], absence of a standardized and unique phenotypic characterization of EARR in terms of diagnostic methods and diagnostic criteria values/thresholds between studies might have contributed to some of this controversy. Nevertheless, this is a critical issue that should be clearly revisited and global consensus-based standards should be achieved in this regard to grant a next generation improvement, not only in internal validity of the studies but equally critical, in external validity of the research in the field. This should support bench-to-clinic research findings with increased certainty levels [1,81,82].

Despite all the aforementioned relevant concerns, this study offers novel and extremely valuable data regarding multiple putative loci and genes located at chromosomes X and Y potentially implicated in this type of extreme phenotype, along with other candidate genes with less imputation power at chromosomes 2, 4, 8, 12, 18, X, and Y. To the best of our knowledge, this is the first XY- chromosome-wide association study (XYWAS) to investigate sex-specific genetic effects on XY chromosomes within one of the most severe phenotypes of EARR in the context of orthodontic forces.

## 5. Conclusions

Multiple putative genetic variants located at chromosomes X and Y are potentially implicated in an extreme phenotype of aEARR. Particularly, STAG2 genetic variants rs151184635 and RP1-30E17.2 genetic variant rs55839915 were found to be associated with an increased risk of being afflicted with aEARR, only in men.

## Figures and Tables

**Table 1:** Population demographics, diagnostic and clinical characteristics of the included patients.

D & Cl Parameters	aEARR^¶^ Cohort	Control Group (*n* = 361)	*p* Value **	OR	95% CI	
	(*n* = 101)				Lower	Upper
Mean age [years]	21.52 ± 11.65	22.83 ± 11.66	0.067	1024	0.998	1051
Sex [n (%)]			0.209	1413	0.824	2.42
female	51 (50.49%)	205 (56.78%)				
male	50 (49.50%)	156 (43.21%)				
Angle classification [n (%)]			0.161	-	-	-
Class I	49 (48.51%)	184 (51.80%)	0.628	1149	0.655	2018
Class II	39 (38.61%)	153 (42.38%)	1	0.49	0.209	1145
Class III	13 (12.87%)	24 (6.64%)				
Treatment [n (%)]			0.145	1668	0.839	3316
extraction	20 (19.80%)	59 (16.34%)				
non-extraction	81 (80.19%)	302 (83.65%)				
Treatment time (m)	27.0 ± 9.10	25.43±7.96	0.095	0.974	0.944	1005
ABO Discrepancy index	15.92 ± 8.91	15.10±8.30	0.876	0.997	0.966	1030
Apical displacement						
Vertical (mm)	−4.44 ± 5.91	−2.83 ± 7.75	0.07	1045	0.996	1095
Sagittal (mm)	−0.57 ± 5.39	−0.25 ± 4.63	0.758	1009	0.953	1068
Vertical (mm) [absolute]	5.32 ± 4.57	4.85 ± 6.88	0.932	0.998	0.957	1041
Sagittal (mm) [absolute]	4.04 ± 3.89	3.31 ± 3.12	0.182	0.951	0.883	1024

D&Cl: Diagnostic and clinical factors; aEARR: aggresive external apical root resorption secondary to mechanical strain; m: months; ABO: American Board of Orthodontics; DI: ABO Discrepancy index (*from Cangialosi TJ,* et al. *The ABO discrepancy index: a measure of case complexity. Am J Orthod Dentofacial Orthop. 2004;125:270-278)*; ^¶^ at least one maxillary incisor with EARR lesion > 5 mm; **: Conditional backward binary logistic regression analysis. Dependent variable = control vs affected patients.

**Table 2:** Lead genetic variants associated with aggresive external apical root resorption [*stratified by sex*] *.

Stratification Code	Lead SNP	OR	Lower	Upper	*p*-Value ^£^	FDR	Chromosome	Gene Name; Source and Description
Male	rs111826558	3.8	1.74	8.21	0.000959316	0.894103771	Chr. X	DMD; dystrophin [Source:HGNC Symbol;Acc:2928]
	rs705896	3.6	1.82	7.27	0.000147132	0.522979691	Chr. X	-
	rs4828068	0.6	0.39	0.82	0.000773819	0.894103771	Chr. X	NOX1; NADPH oxidase 1 [Source:HGNC Symbol;Acc:7889]
	rs5911806	1.8	1.29	2.52	0.00056894	0.894103771	Chr. X	STAG2; stromal antigen 2 [Source:HGNC Symbol;Acc:11355]
	rs151184635	5.9	2.55	13.72	0.000033 **	0.480531966	Chr. X	STAG2; stromal antigen 2 [Source:HGNC Symbol;Acc:11355]
	rs5975024	4.5	1.92	10.37	0.00059286	0.894103771	Chr. X	RP1-30E17.2; Clone-based (Vega) gene
	rs55839915	6	2.37	15.12	0.000146319	0.522979691	Chr. X	RP1-30E17.2; Clone-based (Vega) gene
	rs5976834	4.4	1.85	10.46	0.000972951	0.894103771	Chr. X	LOC107985698
	rs926809	3.8	1.92	7.58	0.00013632	0.522979691	Chr. X	-
	rs6636278	3.3	1.7	6.49	0.000407288	0.894103771	Chr. X	-
Female	rs5962226	5.2	2.22	12.02	0.000211995	1	Chr. X	-
	rs112512284	6.2	2.18	17.54	0.000760667	1	Chr. X	TMEM47; transmembrane protein 47 [Source:HGNC Symbol;Acc:18515]
	rs6521042	0.5	0.27	0.74	0.000767082	1	Chr. X	-
	rs7058787	0.4	0.26	0.74	0.000923093	1	Chr. X	-
	rs4827953	0.4	0.25	0.72	0.000889185	1	Chr. X	-
	rs2180271	0.4	0.21	0.65	0.000221128	1	Chr. X	TAF7L; TAF7-like RNA polymerase II, TATA box binding protein (TBP)-associated factor, 50 kDa [Source:HGNC Symbol;Acc:11548]
	rs6638162	2.5	1.52	4.16	0.00023748	1	Chr. X	-
	rs7049661	0.4	0.26	0.75	0.000964935	1	Chr. X	-
	rs28729587	2.8	1.48	5.4	0.000527269	1	Chr. X	SPRY3; sprouty homolog 3 (Drosophila) [Source:HGNC Symbol;Acc:11271]

* Not adjusted; **^£^**Selected *p* value threshold *p* < 1 × 10^−3^; ** *p* < 1 × 10^−4^; OR: Odds Ratio; FDR: false discovery rate; Chr.: Chromosome.

**Table 3:** Lead genetic variants (adjusted) associated with aggresive external apical root resorption [*stratified by sex*].

Stratification Code	Lead SNP	OR	Lower	Upper	*p*-Value ^£^	FDR	Chromosome	Gene Name; Source and Description
Male	rs4892924	0.37	0.18	0.77	0.000687581	0.778251314	Chr. X	-
	rs62581812	0	0		0.000889678	0.778251314	Chr. X	FAM9B; family with sequence similarity 9, member B [Source:HGNC Symbol;Acc:18404]
	rs61463999	0	0		0.000250861	0.57740729	Chr. X	RP11-40F8.2; Clone-based (Vega) gene
	rs150255888	22.23	2.48	199.29	0.000806144	0.778251314	Chr. X	-
	rs705896	3.7	1.84	7.45	0.000141011	0.501260262	Chr. X	-
	rs5969333	0.44	0.25	0.77	0.000717117	0.778251314	Chr. X	-
	rs5956024	0	0		0.000217592	0.57740729	Chr. X	-
	rs4825856	0.3	0.11	0.83	0.000834288	0.778251314	Chr. X	GRIA3; glutamate receptor, ionotropic, AMPA 3 [Source:HGNC Symbol;Acc:4573]
	rs5911806	1.87	1.32	2.63	0.000324865	0.57740729	Chr. X	STAG2; stromal antigen 2 [Source:HGNC Symbol;Acc:11355]
	rs151184635	6.09	2.6	14.23	0,0000291**	0.41510095	Chr. X	STAG2; stromal antigen 2 [Source:HGNC Symbol;Acc:11355]
	rs5975024	4.93	2.09	11.67	0.000316819	0.57740729	Chr. X	RP1-30E17.2; Clone-based (Vega) gene
	rs55839915	6.86	2.65	17.81	0,0000641**	0.456213349	Chr. X	RP1-30E17.2; Clone-based (Vega) gene
	rs5976834	4.69	1.94	11.35	0.000695616	0.778251314	Chr. X	LOC107985698
Female	rs5962226	5.31	2.27	12.43	0.000179554	0.873550665	Chr. X	-
	rs112512284	6.47	2.25	18.6	0.000624115	0.873550665	Chr. X	TMEM47; transmembrane protein 47 [Source:HGNC Symbol;Acc:18515]
	rs4827953	0.42	0.24	0.72	0.000813414	0.873550665	Chr. X	-
	rs2180271	0.35	0.2	0.64	0.000178648	0.873550665	Chr. X	TAF7L; TAF7-like RNA polymerase II, TATA box binding protein (TBP)-associated factor, 50 kDa [Source:HGNC Symbol;Acc:11548]
	rs61736018	0	0		0.00090046	0.873550665	Chr. X	ARMCX4; armadillo repeat containing, X-linked 4 [Source:HGNC Symbol;Acc:28615]

^£^ Selected *p* value threshold *p* < 1 × 10^−3^; **: *p* < 1 × 10^−4^; OR: Odds Ratio; FDR: false discovery rate; Chr.: Chromosome; Co-variables used for adjustment: Sex, Treatment time, Treatment type.

## Supplementary Materials

The following are available online at https://www.mdpi.com/2075-4426/10/4/169/s1, Supporting Information File 1: eligibility criteria for inclusion/exclusion from the study research and diagnostic-clinical-genetic data recorded for each patient; Supporting Information File 2: full set of diagnostic, clinical, radiogrametric variables recorded for each participant; Supporting Information File 3: quality control steps performed in the genotyped sample; Supporting Information File 4: genetic variants explored in the research at chromosomes 2, 4, 8, 12, 18, X and Y; Supporting Information File 5: SNPs marginally associated with aEARR [*p* < 0.05] at chromosomes 2, 4, 8, 12, 18, X and Y.
