# Supplementary material for: GWAS of Post-Orthodontic Aggressive External Apical Root Resorption Identified Multiple Putative Loci at X-Y Chromosomes
Source: J Pers Med. 2020 Oct 14;10(4):169. doi: 10.3390/jpm10040169 (PMC7712155; doi:10.3390/jpm10040169)
Supplement: Supplementary file 1 [file jpm-10-00169-s001.zip › SUPPL INFO FILE 1.pdf]

Supporting Information File 1. Eligibility criteria for inclusion/exclusion from the study research and diagnostic-clinical-genetic data recorded for each patient.

Eligibility criteria	D&Cl recorded	Genetic factors
Complete orthodontic treatment with fixed appliances	Age	Chromosome X- Y [14,700 genetic variants. *See Supporting Information*
Caucasian origin	Sex [female; male] ¶	
Complete root formation	Treatment time¶	
No previous history of dental trauma	Treatment type [Extraction; Non-extraction] ¶	Candidate SNPs previously studied within EARR [rs1800587, rs1143634, rs419598, rs315952, rs11730582, rs11573856, rs2073618, rs731236, rs1718119,rs2230912, rs7237982, rs8086340, rs17069845, rs1805034, rs12970081, rs17069898, rs4426449]
No systemic pathologies altering hard tissue biology	ABO Discrepancy index (DI)	
No root canal treatment therapy on measured teeth	Angle classification [Class I, Class II, Class III]	
No orthodontic retreatment	Apical displacement [Vertical; Sagittal]	
Available lateral and panoramic pre and post-treatment radiographs	Apical displacement [Vertical; Sagittal] (absolute)	
Genotyping data needed to be available	Other: Asthma medication, Angulation changes U1, Anterior elastics use	

D&Cl: Diagnostic and clinical factors; **ABO:** American Board of Orthodontics; **DI:** ABO Discrepancy index (*from Cangialosi TJ, et al. The ABO discrepancy index: a measure of case complexity. Am J Orthod Dentofacial Orthop. 2004;125:270-278)* ; ¶: co-variables used for adjustment within the genetic analysis.
