# Supplementary material for: GWAS of Post-Orthodontic Aggressive External Apical Root Resorption Identified Multiple Putative Loci at X-Y Chromosomes
Source: J Pers Med. 2020 Oct 14;10(4):169. doi: 10.3390/jpm10040169 (PMC7712155; doi:10.3390/jpm10040169)
Supplement: Supplementary file 1 [file jpm-10-00169-s001.zip › SUPPL INFO FILE 2.pdf]

Supporting Information File 2. Full set of diagnostic, clinical, radiogrametric variables recorded for each participant

Gender	Type of treatment [extraction, non extraction]	Apical morphology	Discrepancy index of the ABO
Age	Treatment time	Sharp morphology	Overjet
Type of anterior tooth measured	Type of extraction	Round morphology	Overbite
Endodontic treatment performed	Intraoral distalizer	Squared morphology	Anterior open bite
Maloclussion	Rapid maxillary expansion	Curve morphology	Lateral open bite
Previous tooth trauma	Slow maxillary expansion	Vertical apical absolute displacement	Crowding
Traumatized tooth	Full archwire sequence mantained	Saggital apical absolute displacement	Occlusal relationship
Asthma medication	Rebonding of studied tooth	Vertical apical displacement	Lingual posterior crossbite
Immature deglutition	Class I,II,III elastics	Saggital apical displacement	Buccal posterior crossbite
Onicophagy	Anterior elastics	Upper incisor flawring	DI cephalometric assessment
Oral breathing	Intrusive force on studied tooth	Initial Upper incisors angulation	other
Tonghe piercing	Extrusive force on studied tooth	Final Upper incisors angulation	
Smoking	Selective torsion	Tooth crown-root initial measurements	
Impacted canines	Selective inset	Tooth crown-root final measurements	
Hypoplasic tooth	Selective offset		
Microdoncy			

ABO: American Board of Orthodontics; DI: ABO Discrepancy index (from Cangialosi TJ, et al. The ABO discrepancy index: a measure of case complexity. Am J Orthod Dentofacial Orthop. 2004;125:270-278)
