# Supplementary material for: GWAS of Post-Orthodontic Aggressive External Apical Root Resorption Identified Multiple Putative Loci at X-Y Chromosomes
Source: J Pers Med. 2020 Oct 14;10(4):169. doi: 10.3390/jpm10040169 (PMC7712155; doi:10.3390/jpm10040169)
Supplement: Supplementary file 1 [file jpm-10-00169-s001.zip › SUPPL INFO FILE 3.pdf]

Supporting Information File 3. Quality control steps performed in the genotyped sample

GWAS Quality control
1. - Genotype missing control: The call rate of samples and SNPs analysed was higher than 0.90
2. - Minor allele frequency (MAF): We checked out the MAF in all the SNPs to ensure that enough number of the rare genotypes was present in the dataset. We only included polymorphisms with MAF ≥ 1%.
3- Cryptic relatedness: We assumed that there wasn't cryptic relatedness between study participants. Cryptic relatedness refers to the idea that some subjects of a case-control study might in fact be close relatives, thus their genotypes being not independent from the population frequencies

MAF: Minor allele frequency; SNPs: Single Nucleotide Polymorphisms
