# Supplementary material for: GWAS of Post-Orthodontic Aggressive External Apical Root Resorption Identified Multiple Putative Loci at X-Y Chromosomes
Source: J Pers Med. 2020 Oct 14;10(4):169. doi: 10.3390/jpm10040169 (PMC7712155; doi:10.3390/jpm10040169)
Supplement: Supplementary file 1 [file jpm-10-00169-s001.zip › SUPPL INFO FILE 4.pdf]

Supporting Information File 4 . Genetic variants explored in the research at chromosomes 2, 4, 8, 12, 18, X and Y.

SNP	Chromosome	gene name	gene_source	description
rs1800587		IL1A	HGNC Symbol	interleukin 1, alpha [Source:HGNC Symbol;Acc:5992]
rs1143634	2	IL1B	HGNC Symbol	interleukin 1, beta [Source:HGNC Symbol;Acc:5992]
rs419598	2	IL1RN	HGNC Symbol	interleukin 1 receptor antagonist [Source:HGNC Symbol;Acc:6000]
rs315952	2	IL1RN	HGNC Symbol	interleukin 1 receptor antagonist [Source:HGNC Symbol;Acc:6000]
rs11730582				
rs11573856	8	TNFRSF11B	HGNC Symbol	tumor necrosis factor receptor superfamily, member 11b [Source:HGNC Symbol;Acc:11909]
rs2073618	8	TNFRSF11B	HGNC Symbol	tumor necrosis factor receptor superfamily, member 11b [Source:HGNC Symbol;Acc:11909]
rs731236	12	VDR	HGNC Symbol	vitamin D (1,25- dihydroxyvitamin D3) receptor [Source:HGNC Symbol;Acc:12679]
rs1718119	12	P2RX7	HGNC Symbol	purinergic receptor P2X, ligand-gated ion channel, 7 [Source:HGNC Symbol;Acc:8537]
rs2230912	12	P2RX7	HGNC Symbol	purinergic receptor P2X, ligand-gated ion channel, 7 [Source:HGNC Symbol;Acc:8537]
rs7237982	18	TNFRSF11A	HGNC Symbol	tumor necrosis factor receptor superfamily, member 11a, NFKB activator [Source:HGNC Symbol;Acc:11908]
rs8086340	18	TNFRSF11A	HGNC Symbol	tumor necrosis factor receptor superfamily, member 11a, NFKB activator [Source:HGNC Symbol;Acc:11908]
rs17069845	18	TNFRSF11A	HGNC Symbol	tumor necrosis factor receptor superfamily, member 11a, NFKB activator [Source:HGNC Symbol;Acc:11908]
rs1805034	18	TNFRSF11A	HGNC Symbol	tumor necrosis factor receptor superfamily, member 11a, NFKB activator [Source:HGNC Symbol;Acc:11908]
rs12970081	18	TNFRSF11A	HGNC Symbol	tumor necrosis factor receptor superfamily, member 11a, NFKB activator [Source:HGNC Symbol;Acc:11908]
rs17069898	18	TNFRSF11A	HGNC Symbol	tumor necrosis factor receptor superfamily, member 11a, NFKB activator [Source:HGNC Symbol;Acc:11908]
rs4426449	18	TNFRSF11A;RP11-640A1.3	HGNC Symbol;Clone-based (Vega) gene	tumor necrosis factor receptor superfamily, member 11a, NFKB activator [Source:HGNC Symbol;Acc:11908];
rs60075487	X	XG	HGNC Symbol	Xg blood group [Source:HGNC Symbol;Acc:12806]
rs2306736	X	XG	HGNC Symbol	Xg blood group [Source:HGNC Symbol;Acc:12806]
rs5939319	X	XG	HGNC Symbol	Xg blood group [Source:HGNC Symbol;Acc:12806]
rs5939320	X	XG	HGNC Symbol	Xg blood group [Source:HGNC Symbol;Acc:12806]
rs73433431	X	XG	HGNC Symbol	Xg blood group [Source:HGNC Symbol;Acc:12806]
rs1419931	X	XG	HGNC Symbol	Xg blood group [Source:HGNC Symbol;Acc:12806]
rs4892892	X	XG	HGNC Symbol	Xg blood group [Source:HGNC Symbol;Acc:12806]
rs311196	X	XG	HGNC Symbol	Xg blood group [Source:HGNC Symbol;Acc:12806]

SNP	Chromosome	gene name	gene source	description
rs145587108	X	XG	HGNC Symbol	Xg blood group [Source:HGNC Symbol;Acc:12806]
rs7058222	X	XG	HGNC Symbol	Xg blood group [Source:HGNC Symbol;Acc:12806]
rs3749988	X	XG	HGNC Symbol	Xg blood group [Source:HGNC Symbol;Acc:12806]
rs5939125	X	XG	HGNC Symbol	Xg blood group [Source:HGNC Symbol;Acc:12806]
rs141205366				
rs5982588				
rs5982872				
rs5939350	X	GYG2	HGNC Symbol	glycogenin 2 [Source:HGNC Symbol;Acc:4700]
rs12396748	X	GYG2	HGNC Symbol	glycogenin 2 [Source:HGNC Symbol;Acc:4700]
rs11152548	X	GYG2	HGNC Symbol	glycogenin 2 [Source:HGNC Symbol;Acc:4700]
rs5982593	X	GYG2	HGNC Symbol	glycogenin 2 [Source:HGNC Symbol;Acc:4700]
rs5982891	X	GYG2	HGNC Symbol	glycogenin 2 [Source:HGNC Symbol;Acc:4700]
rs62582317	X	GYG2	HGNC Symbol	glycogenin 2 [Source:HGNC Symbol;Acc:4700]
rs5939362	X	GYG2;GYG2-AS1	HGNC Symbol	glycogenin 2 [Source:HGNC Symbol;Acc:4700];GYG2 antisense RNA 1 [Source:HGNC Symbol;Acc:40270]
rs5982897	X	GYG2	HGNC Symbol	glycogenin 2 [Source:HGNC Symbol;Acc:4700]
rs2306734	X	GYG2	HGNC Symbol	glycogenin 2 [Source:HGNC Symbol;Acc:4700]
rs2306735	X	GYG2	HGNC Symbol	glycogenin 2 [Source:HGNC Symbol;Acc:4700]
rs17330993	X	GYG2	HGNC Symbol	glycogenin 2 [Source:HGNC Symbol;Acc:4700]
rs6642045	X	GYG2	HGNC Symbol	glycogenin 2 [Source:HGNC Symbol;Acc:4700]
rs5939137	X	GYG2	HGNC Symbol	glycogenin 2 [Source:HGNC Symbol;Acc:4700]
rs62582329	X	GYG2	HGNC Symbol	glycogenin 2 [Source:HGNC Symbol;Acc:4700]
rs5982603	X	GYG2	HGNC Symbol	glycogenin 2 [Source:HGNC Symbol;Acc:4700]
rs5939384	X	GYG2	HGNC Symbol	glycogenin 2 [Source:HGNC Symbol;Acc:4700]
rs5939139	X	GYG2	HGNC Symbol	glycogenin 2 [Source:HGNC Symbol;Acc:4700]
rs12008127	X	GYG2	HGNC Symbol	glycogenin 2 [Source:HGNC Symbol;Acc:4700]
rs41311459	X	GYG2	HGNC Symbol	glycogenin 2 [Source:HGNC Symbol;Acc:4700]
rs211660	X	GYG2	HGNC Symbol	glycogenin 2 [Source:HGNC Symbol;Acc:4700]
rs62582348				
rs76495523				
rs56019734				
rs6642064				
rs5939388				

SNP	Chromosome	gene name	gene source	description
rs149319890				
rs5939390				
rs5982611				
rs4141210				
rs2228431	X	ARSD	HGNC Symbol	arylsulfatase D [Source:HGNC Symbol;Acc:717]
rs6642067	X	ARSD	HGNC Symbol	arylsulfatase D [Source:HGNC Symbol;Acc:717]
rs34494682	X	ARSD	HGNC Symbol	arylsulfatase D [Source:HGNC Symbol;Acc:717]
rs211649	X	ARSD	HGNC Symbol	arylsulfatase D [Source:HGNC Symbol;Acc:717]
rs1637778	X	ARSD	HGNC Symbol	arylsulfatase D [Source:HGNC Symbol;Acc:717]
rs45626632	X	ARSD	HGNC Symbol	arylsulfatase D [Source:HGNC Symbol;Acc:717]
rs1698814	X	ARSD	HGNC Symbol	arylsulfatase D [Source:HGNC Symbol;Acc:717]
rs2302077	X	ARSD	HGNC Symbol	arylsulfatase D [Source:HGNC Symbol;Acc:717]
rs3747394	X	ARSD	HGNC Symbol	arylsulfatase D [Source:HGNC Symbol;Acc:717]
rs6567682				
rs1875648				
rs5982926				
rs11055	X	ARSE	HGNC Symbol	arylsulfatase E (chondrodysplasia punctata 1) [Source:HGNC Symbol;Acc:719]
rs11798027	X	ARSE	HGNC Symbol	arylsulfatase E (chondrodysplasia punctata 1) [Source:HGNC Symbol;Acc:719]
rs169828	X	ARSE	HGNC Symbol	arylsulfatase E (chondrodysplasia punctata 1) [Source:HGNC Symbol;Acc:719]
rs211639	X	ARSE	HGNC Symbol	arylsulfatase E (chondrodysplasia punctata 1) [Source:HGNC Symbol;Acc:719]
rs73193028	X	ARSE	HGNC Symbol	arylsulfatase E (chondrodysplasia punctata 1) [Source:HGNC Symbol;Acc:719]
rs147884967				
rs111820062				
rs67266619				
rs61293843				
rs7878711	X	ARSH	HGNC Symbol	arylsulfatase family, member H [Source:HGNC Symbol;Acc:32488]
rs79487908	X	ARSH	HGNC Symbol	arylsulfatase family, member H [Source:HGNC Symbol;Acc:32488]
rs5939404	X	ARSH	HGNC Symbol	arylsulfatase family, member H [Source:HGNC Symbol;Acc:32488]
rs11797171	X	ARSH	HGNC Symbol	arylsulfatase family, member H [Source:HGNC Symbol;Acc:32488]
rs4892830	X	ARSH	HGNC Symbol	arylsulfatase family, member H [Source:HGNC Symbol;Acc:32488]
rs11152555	X	ARSH	HGNC Symbol	arylsulfatase family, member H [Source:HGNC Symbol;Acc:32488]
rs61751925	X	ARSH	HGNC Symbol	arylsulfatase family, member H [Source:HGNC Symbol;Acc:32488]
rs61978642	X	ARSH	HGNC Symbol	arylsulfatase family, member H [Source:HGNC Symbol;Acc:32488]

SNP	Chromosome	gene name	gene source	description
rs5939413				
rs5939424	X	ARSF	HGNC Symbol	arylsulfatase F [Source:HGNC Symbol;Acc:721]
rs4018641	X	ARSF	HGNC Symbol	arylsulfatase F [Source:HGNC Symbol;Acc:721]
rs112655645	X	ARSF	HGNC Symbol	arylsulfatase F [Source:HGNC Symbol;Acc:721]
rs2012433	X	ARSF	HGNC Symbol	arylsulfatase F [Source:HGNC Symbol;Acc:721]
rs5982999	X	ARSF	HGNC Symbol	arylsulfatase F [Source:HGNC Symbol;Acc:721]
rs45550436	X	ARSF	HGNC Symbol	arylsulfatase F [Source:HGNC Symbol;Acc:721]
rs112461718	X	ARSF	HGNC Symbol	arylsulfatase F [Source:HGNC Symbol;Acc:721]
rs5983003	X	ARSF	HGNC Symbol	arylsulfatase F [Source:HGNC Symbol;Acc:721]
rs73193043	X	ARSF	HGNC Symbol	arylsulfatase F [Source:HGNC Symbol;Acc:721]
rs5939440	X	ARSF	HGNC Symbol	arylsulfatase F [Source:HGNC Symbol;Acc:721]
rs1869561	X	ARSF	HGNC Symbol	arylsulfatase F [Source:HGNC Symbol;Acc:721]
rs17051486	X	ARSF	HGNC Symbol	arylsulfatase F [Source:HGNC Symbol;Acc:721]
rs5939453	X	ARSF	HGNC Symbol	arylsulfatase F [Source:HGNC Symbol;Acc:721]
rs1052638	X	ARSF	HGNC Symbol	arylsulfatase F [Source:HGNC Symbol;Acc:721]
rs5983023				
rs12009309				
rs4892924				
rs5982670				
rs73443876				
rs1194776				
rs12559961				
rs2694740				
rs2704855				
rs73193064				
rs17051544				
rs11798555				
rs17051775				
rs2694728				
rs5982686				
rs2694713				
rs4119090				
rs1205544				

SNP	Chromosome	gene name	gene source	description
rs12556961				
rs7064081				
rs12559033	X	CXorf28	HGNC Symbol	chromosome X open reading frame 28 [Source:HGNC Symbol;Acc:27336]
rs73175509	X	CXorf28	HGNC Symbol	chromosome X open reading frame 28 [Source:HGNC Symbol;Acc:27336]
rs1726174				
rs5939486				
rs1726172				
rs1635232				
rs67510430	X	MXRA5	HGNC Symbol	matrix-remodelling associated 5 [Source:HGNC Symbol;Acc:7539]
rs1635242	X	MXRA5	HGNC Symbol	matrix-remodelling associated 5 [Source:HGNC Symbol;Acc:7539]
rs146807533	X	MXRA5	HGNC Symbol	matrix-remodelling associated 5 [Source:HGNC Symbol;Acc:7539]
rs41305155	X	MXRA5	HGNC Symbol	matrix-remodelling associated 5 [Source:HGNC Symbol;Acc:7539]
rs1726199	X	MXRA5	HGNC Symbol	matrix-remodelling associated 5 [Source:HGNC Symbol;Acc:7539]
rs139106444	X	MXRA5	HGNC Symbol	matrix-remodelling associated 5 [Source:HGNC Symbol;Acc:7539]
rs1635246	X	MXRA5	HGNC Symbol	matrix-remodelling associated 5 [Source:HGNC Symbol;Acc:7539]
rs1635247	X	MXRA5	HGNC Symbol	matrix-remodelling associated 5 [Source:HGNC Symbol;Acc:7539]
rs41304689	X	MXRA5	HGNC Symbol	matrix-remodelling associated 5 [Source:HGNC Symbol;Acc:7539]
rs5983119	X	MXRA5	HGNC Symbol	matrix-remodelling associated 5 [Source:HGNC Symbol;Acc:7539]
rs5939496	X	MXRA5	HGNC Symbol	matrix-remodelling associated 5 [Source:HGNC Symbol;Acc:7539]
rs5939184	X	MXRA5	HGNC Symbol	matrix-remodelling associated 5 [Source:HGNC Symbol;Acc:7539]
rs12857026				
rs34767302				
rs7887742				
rs73175543				
rs1617688				
rs28439834				
rs5982705				
rs806617				
rs1989994				
rs5939198				
rs148620565				
rs73175573				
rs73434347				

SNP	Chromosome	gene name	gene source	description
rs73175582				
rs11152500				
rs35181208				
rs6567517				
rs17266501				
rs6567523				
rs6567524				
rs73178105				
rs7880892				
rs7059886				
rs12842535				
rs6641773				
rs146748050				
rs149426820				
rs62581991				
rs112210343				
rs11152506				
rs11152509				
rs11152510				
rs11152511				
rs73178117				
rs7879808				
rs56157309				
rs7889116				
rs11152524				
rs140881654				
rs62582034				
rs72619401				
rs7883545				
rs7884316				
rs150932516				
rs6641797				
rs6567569	X	PRKX	HGNC Symbol	protein kinase, X-linked [Source:HGNC Symbol;Acc:9441]

SNP	Chromosome	gene name	gene source	description
rs138239409	X	PRKX	HGNC Symbol	protein kinase, X-linked [Source:HGNC Symbol;Acc:9441]
rs2287238	X	PRKX	HGNC Symbol	protein kinase, X-linked [Source:HGNC Symbol;Acc:9441]
rs2302750	X	PRKX	HGNC Symbol	protein kinase, X-linked [Source:HGNC Symbol;Acc:9441]
rs75518546	X	PRKX	HGNC Symbol	protein kinase, X-linked [Source:HGNC Symbol;Acc:9441]
rs12388899	X	PRKX	HGNC Symbol	protein kinase, X-linked [Source:HGNC Symbol;Acc:9441]
rs6641804	X	PRKX	HGNC Symbol	protein kinase, X-linked [Source:HGNC Symbol;Acc:9441]
rs6641806	X	PRKX	HGNC Symbol	protein kinase, X-linked [Source:HGNC Symbol;Acc:9441]
rs17051658	X	PRKX	HGNC Symbol	protein kinase, X-linked [Source:HGNC Symbol;Acc:9441]
rs73178144	X	PRKX	HGNC Symbol	protein kinase, X-linked [Source:HGNC Symbol;Acc:9441]
rs12399124	X	PRKX	HGNC Symbol	protein kinase, X-linked [Source:HGNC Symbol;Acc:9441]
rs57086720	X	PRKX	HGNC Symbol	protein kinase, X-linked [Source:HGNC Symbol;Acc:9441]
rs35776967	X	PRKX	HGNC Symbol	protein kinase, X-linked [Source:HGNC Symbol;Acc:9441]
rs67393171	X	PRKX	HGNC Symbol	protein kinase, X-linked [Source:HGNC Symbol;Acc:9441]
rs73438449	X	PRKX	HGNC Symbol	protein kinase, X-linked [Source:HGNC Symbol;Acc:9441]
rs187989564	X	PRKX	HGNC Symbol	protein kinase, X-linked [Source:HGNC Symbol;Acc:9441]
rs12839071	X	PRKX	HGNC Symbol	protein kinase, X-linked [Source:HGNC Symbol;Acc:9441]
rs67222009	X	PRKX	HGNC Symbol	protein kinase, X-linked [Source:HGNC Symbol;Acc:9441]
rs17335275	X	PRKX	HGNC Symbol	protein kinase, X-linked [Source:HGNC Symbol;Acc:9441]
rs17331167	X	PRKX	HGNC Symbol	protein kinase, X-linked [Source:HGNC Symbol;Acc:9441]
rs73438464	X	PRKX	HGNC Symbol	protein kinase, X-linked [Source:HGNC Symbol;Acc:9441]
rs2058865	X	PRKX;PRKX-AS1	HGNC Symbol	protein kinase, X-linked [Source:HGNC Symbol;Acc:9441];PRKX antisense RNA 1 [Source:HGNC Symbol;Acc:40479]
rs17260002	X	PRKX	HGNC Symbol	protein kinase, X-linked [Source:HGNC Symbol;Acc:9441]
rs7880937	X	PRKX	HGNC Symbol	protein kinase, X-linked [Source:HGNC Symbol;Acc:9441]
rs12559003	X	PRKX	HGNC Symbol	protein kinase, X-linked [Source:HGNC Symbol;Acc:9441]
rs12387087	X	PRKX	HGNC Symbol	protein kinase, X-linked [Source:HGNC Symbol;Acc:9441]
rs6641847	X	PRKX	HGNC Symbol	protein kinase, X-linked [Source:HGNC Symbol;Acc:9441]
rs12011735	X	PRKX	HGNC Symbol	protein kinase, X-linked [Source:HGNC Symbol;Acc:9441]
rs55700102	X	PRKX	HGNC Symbol	protein kinase, X-linked [Source:HGNC Symbol;Acc:9441]
rs11795926	X	PRKX	HGNC Symbol	protein kinase, X-linked [Source:HGNC Symbol;Acc:9441]
rs6641856	X	PRKX	HGNC Symbol	protein kinase, X-linked [Source:HGNC Symbol;Acc:9441]
rs7879951	X	PRKX	HGNC Symbol	protein kinase, X-linked [Source:HGNC Symbol;Acc:9441]
rs6567591	X	PRKX	HGNC Symbol	protein kinase, X-linked [Source:HGNC Symbol;Acc:9441]

SNP	Chromosome	gene name	gene source	description
rs57534675				
rs5916456				
rs5961501				
rs28720078				
rs12687129				
rs4826699				
rs5961345				
rs73178179				
rs6641876				
rs59003681				
rs5961447				
rs7877157				
rs3876184				
rs60309358				
rs17331195				
rs6641889				
rs34641353				
rs5916569				
rs34461521				
rs6608351				
rs72611118				
rs5916603				
rs112155371				
rs5961490				
rs111602303				
rs4826886				
rs113795337				
rs17313406				
rs6649581				
rs28465437				
rs62575757				
rs5916684				

SNP	Chromosome	gene name	gene source	description
rs5915430	X	RP11-706O15.1	Clone-based (Vega) gene	HCG1981372, isoform CRA_c; Uncharacterized protein [Source:UniProtKB/TrEMBL;Acc:B1B108]
rs113371969				
rs5961696				
rs113862114				
rs7052908				
rs6649551				
rs111671121				
rs5916101				
rs6649535				
rs17139400				
rs6649501				
rs17218582				
rs5962038				
rs5916449				
rs5962049				
rs5962050				
rs6640754				
rs2365129				
rs12156958				
rs6641085				
rs1526471				
rs72611154				
rs1526465				
rs5962066				
rs6638493				
rs5916513				
rs5962092				
rs16979590				
rs6529899				
rs66634514				
rs142744902				
rs73180343				

SNP	Chromosome	gene name	gene source	description
rs5915726				
rs5916545				
rs5962112				
rs5916553				
rs17218967				
rs2363839				
rs3843796				
rs17219044				
rs139134877				
rs6639795				
rs55989413				
rs7061324				
rs4110045				
rs17266961				
rs1707492				
rs5915749				
rs6638839				
rs73182290				
rs4086429				
rs12838423				
rs17313736				
rs149513856				
rs5916639				
rs144047820				
rs4335291				
rs73448021				
rs2885535				
rs12850340				
rs5962212	X	RP11-707P20.1	Clone-based (Vega) gene	
rs5961510				
rs5916664				
rs5961512				

SNP	Chromosome	gene name	gene source	description
rs73448070				
rs17267093				
rs5916679				
rs5916680				
rs5962222				
rs5916687				
rs6639025				
rs28714275				
rs5962226				
rs5915780				
rs6639033				
rs1980386				
rs11095544				
rs73183879				
rs16981289				
rs2885533				
rs73432316				
rs113812068				
rs6640699				
rs5962239				
rs5915786				
rs73625800				
rs12557752				
rs12858750				
rs5915799				
rs5915434				
rs5915814				
rs17267288				
rs1527125				
rs7888591				
rs59515540				
rs5915449				
rs62592523				

SNP	Chromosome	gene name	gene source	description
rs5915451				
rs5915452				
rs111959810				
rs139211798				
rs6639189				
rs5961278				
rs11795991				
rs144409421				
rs12013853				
rs5961558				
rs6641142				
rs5961574				
rs12556588				
rs57950457				
rs11798922				
rs5961583				
rs5961284				
rs2218680				
rs35219779				
rs36076913				
rs5961289				
rs17304608				
rs12840872				
rs115944192				
rs1448509				
rs17304727				
rs10521585				
rs68013795				
rs73187720				
rs62590991				
rs148002227				
rs5915975				
rs17304854				

SNP	Chromosome	gene name	gene source	description
rs68147309				
rs5915498				
rs5916000				
rs66600356				
rs12833835				
rs2927265				
rs7065808				
rs1993794				
rs73187749				
rs5916019				
rs7054804				
rs5916024				
rs73187750				
rs1026327				
rs73187767				
rs17267930				
rs7888448				
rs113732757				
rs146051654				
rs6638512				
rs5961309				
rs150262381				
rs56394555				
rs73189710				
rs5915533				
rs144132782				
rs111676544				
rs1947410				
rs17219924				
rs713058				
rs67672403				
rs111277500				
rs73189721				

SNP	Chromosome	gene name	gene source	description
rs12008707				
rs73436810				
rs4316280				
rs5916050				
rs12010896				
rs7054190				
rs113503100				
rs16983676				
rs62582070				
rs6529794				
rs5961705				
rs5915538				
rs4439269				
rs5915539				
rs66526345				
rs116730063				
rs5916066				
rs62582074				
rs73189786				
rs112262940				
rs11796356				
rs7063596				
rs12842616				
rs66710845				
rs149156025				
rs5916136				
rs16983729				
rs5916139				
rs5916158				
rs5916162				
rs17220204				
rs5961324				
rs5916174				

SNP	Chromosome	gene name	gene source	description
rs5961325				
rs12846084				
rs11094777				
rs5961751				
rs142833464				
rs9698745				
rs5915584	X	RP11-733O18.1	Clone-based (Vega) gene	
rs11377806	X	RP11-733O18.1	Clone-based (Vega) gene	
rs5915591	X	RP11-733O18.1	Clone-based (Vega) gene	
rs5915594	X	RP11-733O18.1	Clone-based (Vega) gene	
rs10776301	X	RP11-733O18.1	Clone-based (Vega) gene	
rs56401541	X	RP11-733O18.1	Clone-based (Vega) gene	
rs4826802	X	RP11-733O18.1	Clone-based (Vega) gene	
rs1032036	X	RP11-733O18.1	Clone-based (Vega) gene	
rs5916242				
rs112551845				
rs17305516				
rs73451282				
rs5961350				
rs6639489				
rs4826816				
rs56033575				
rs12853035				
rs12847350				
rs1482815				
rs7885928				
rs10482266				
rs10126348				

SNP	Chromosome	gene name	gene source	description
rs143708829				
rs1351651				
rs11094819				
rs73182557				
rs111833397				
rs112699396				
rs5961866				
rs5961371				
rs5961868				
rs1482816				
rs73453381				
rs1384521	X	NLGN4X	HGNC Symbol	neuroligin 4, X-linked [Source:HGNC Symbol;Acc:14287]
rs144051871	X	NLGN4X	HGNC Symbol	neuroligin 4, X-linked [Source:HGNC Symbol;Acc:14287]
rs17305698	X	NLGN4X	HGNC Symbol	neuroligin 4, X-linked [Source:HGNC Symbol;Acc:14287]
rs1384520	X	NLGN4X	HGNC Symbol	neuroligin 4, X-linked [Source:HGNC Symbol;Acc:14287]
rs3848887	X	NLGN4X	HGNC Symbol	neuroligin 4, X-linked [Source:HGNC Symbol;Acc:14287]
rs2128515	X	NLGN4X	HGNC Symbol	neuroligin 4, X-linked [Source:HGNC Symbol;Acc:14287]
rs12559511	X	NLGN4X	HGNC Symbol	neuroligin 4, X-linked [Source:HGNC Symbol;Acc:14287]
rs73182583	X	NLGN4X	HGNC Symbol	neuroligin 4, X-linked [Source:HGNC Symbol;Acc:14287]
rs12860326	X	NLGN4X	HGNC Symbol	neuroligin 4, X-linked [Source:HGNC Symbol;Acc:14287]
rs3810686	X	NLGN4X	HGNC Symbol	neuroligin 4, X-linked [Source:HGNC Symbol;Acc:14287]
rs56336632	X	NLGN4X	HGNC Symbol	neuroligin 4, X-linked [Source:HGNC Symbol;Acc:14287]
rs17315232	X	NLGN4X	HGNC Symbol	neuroligin 4, X-linked [Source:HGNC Symbol;Acc:14287]
rs7049300	X	NLGN4X	HGNC Symbol	neuroligin 4, X-linked [Source:HGNC Symbol;Acc:14287]
rs12394287	X	NLGN4X	HGNC Symbol	neuroligin 4, X-linked [Source:HGNC Symbol;Acc:14287]
rs180826435	X	NLGN4X	HGNC Symbol	neuroligin 4, X-linked [Source:HGNC Symbol;Acc:14287]
rs6638583	X	NLGN4X	HGNC Symbol	neuroligin 4, X-linked [Source:HGNC Symbol;Acc:14287]
rs4132789	X	NLGN4X	HGNC Symbol	neuroligin 4, X-linked [Source:HGNC Symbol;Acc:14287]
rs35515178	X	NLGN4X	HGNC Symbol	neuroligin 4, X-linked [Source:HGNC Symbol;Acc:14287]
rs6639580	X	NLGN4X	HGNC Symbol	neuroligin 4, X-linked [Source:HGNC Symbol;Acc:14287]
rs147450716	X	NLGN4X	HGNC Symbol	neuroligin 4, X-linked [Source:HGNC Symbol;Acc:14287]
rs5915634	X	NLGN4X	HGNC Symbol	neuroligin 4, X-linked [Source:HGNC Symbol;Acc:14287]
rs73184635	X	NLGN4X	HGNC Symbol	neuroligin 4, X-linked [Source:HGNC Symbol;Acc:14287]

SNP	Chromosome	gene name	gene source	description
rs1921948	X	NLGN4X	HGNC Symbol	neuroligin 4, X-linked [Source:HGNC Symbol;Acc:14287]
rs55999636	X	NLGN4X	HGNC Symbol	neuroligin 4, X-linked [Source:HGNC Symbol;Acc:14287]
rs5915644	X	NLGN4X	HGNC Symbol	neuroligin 4, X-linked [Source:HGNC Symbol;Acc:14287]
rs12847926	X	NLGN4X	HGNC Symbol	neuroligin 4, X-linked [Source:HGNC Symbol;Acc:14287]
rs12556649	X	NLGN4X	HGNC Symbol	neuroligin 4, X-linked [Source:HGNC Symbol;Acc:14287]
rs4593705	X	NLGN4X	HGNC Symbol	neuroligin 4, X-linked [Source:HGNC Symbol;Acc:14287]
rs1997481	X	NLGN4X	HGNC Symbol	neuroligin 4, X-linked [Source:HGNC Symbol;Acc:14287]
rs73184638	X	NLGN4X	HGNC Symbol	neuroligin 4, X-linked [Source:HGNC Symbol;Acc:14287]
rs56002996	X	NLGN4X	HGNC Symbol	neuroligin 4, X-linked [Source:HGNC Symbol;Acc:14287]
rs60973092	X	NLGN4X	HGNC Symbol	neuroligin 4, X-linked [Source:HGNC Symbol;Acc:14287]
rs73184645	X	NLGN4X	HGNC Symbol	neuroligin 4, X-linked [Source:HGNC Symbol;Acc:14287]
rs73184648	X	NLGN4X	HGNC Symbol	neuroligin 4, X-linked [Source:HGNC Symbol;Acc:14287]
rs73184652	X	NLGN4X	HGNC Symbol	neuroligin 4, X-linked [Source:HGNC Symbol;Acc:14287]
rs17325953	X	NLGN4X	HGNC Symbol	neuroligin 4, X-linked [Source:HGNC Symbol;Acc:14287]
rs6529924	X	NLGN4X	HGNC Symbol	neuroligin 4, X-linked [Source:HGNC Symbol;Acc:14287]
rs4826837	X	NLGN4X	HGNC Symbol	neuroligin 4, X-linked [Source:HGNC Symbol;Acc:14287]
rs4826714	X	NLGN4X	HGNC Symbol	neuroligin 4, X-linked [Source:HGNC Symbol;Acc:14287]
rs5916335	X	NLGN4X	HGNC Symbol	neuroligin 4, X-linked [Source:HGNC Symbol;Acc:14287]
rs1882409	X	NLGN4X	HGNC Symbol	neuroligin 4, X-linked [Source:HGNC Symbol;Acc:14287]
rs5915659	X	NLGN4X	HGNC Symbol	neuroligin 4, X-linked [Source:HGNC Symbol;Acc:14287]
rs57503062	X	NLGN4X	HGNC Symbol	neuroligin 4, X-linked [Source:HGNC Symbol;Acc:14287]
rs73457060	X	NLGN4X	HGNC Symbol	neuroligin 4, X-linked [Source:HGNC Symbol;Acc:14287]
rs5916356				
rs58476471				
rs5961971				
rs112335448				
rs1455320				
rs55984002				
rs7064335				
rs73461335				
rs12837275				
rs73184673				
rs7885458				

SNP	Chromosome	gene name	gene source	description
rs73443705				
rs5961419				
rs5961982				
rs1455322				
rs2061108				
rs5961424				
rs12842225				
rs720802				
rs897394				
rs7059003				
rs5962008				
rs67925447				
rs12395835				
rs188699000				
rs7051201				
rs5916408				
rs57166568				
rs7889294				
rs4351578				
rs73186886				
rs7877850				
rs5989589				
rs62588650				
rs68111769				
rs12841600				
rs138043500				
rs56227048				
rs56114712				
rs73188618				
rs5989547				
rs73188626				
rs12558428				
rs147295419				

SNP	Chromosome	gene name	gene source	description
rs5989514				
rs5989517				
rs5989523				
rs5989484	X	RP11-1M18.1	Clone-based (Vega) gene	
rs4914919	X	RP11-1M18.1	Clone-based (Vega) gene	
rs5989546	X	RP11-1M18.1	Clone-based (Vega) gene	
rs73188673	X	RP11-1M18.1	Clone-based (Vega) gene	
rs7062697	X	RP11-1M18.1	Clone-based (Vega) gene	
rs5948738	X	RP11-1M18.1	Clone-based (Vega) gene	
rs5948739				
rs5989490				
rs2369822				
rs12836616				
rs73627088				
rs113368753				
rs55637165				
rs16984312				
rs5948724				
rs12013264				
rs5989496				
rs6639662				
rs5948750				
rs7891165				
rs5948753				
rs4914907				
rs77230672				
rs2122441				
rs11798017				
rs66999511				

SNP	Chromosome	gene name	gene source	description
rs6529981				
rs7886333				
rs17306376				
rs12011520				
rs11095215				
rs6639680				
rs62590383				
rs5989586				
rs12011309				
rs73190746				
rs55909210				
rs7471544				
rs12688222				
rs114443125				
rs3924842				
rs146136965				
rs72609587				
rs11095246				
rs12006786				
rs56261234				
rs6638637				
rs6639711				
rs5934247				
rs5978809				
rs6530010				
rs5934677				
rs4830729				
rs5933875				
rs12557257				
rs62590456				
rs17331284				
rs5978827				
rs5978220	X	HDHD1	HGNC Symbol	haloacid dehalogenase-like hydrolase domain containing 1 [Source:HGNC Symbol;Acc:16818]

SNP	Chromosome	gene name	gene source	description
rs868756	X	HDHD1	HGNC Symbol	haloacid dehalogenase-like hydrolase domain containing 1 [Source:HGNC Symbol;Acc:16818]
rs62588691	X	HDHD1	HGNC Symbol	haloacid dehalogenase-like hydrolase domain containing 1 [Source:HGNC Symbol;Acc:16818]
rs2379206	X	HDHD1	HGNC Symbol	haloacid dehalogenase-like hydrolase domain containing 1 [Source:HGNC Symbol;Acc:16818]
rs2379207	X	HDHD1	HGNC Symbol	haloacid dehalogenase-like hydrolase domain containing 1 [Source:HGNC Symbol;Acc:16818]
rs1803675	X	HDHD1	HGNC Symbol	haloacid dehalogenase-like hydrolase domain containing 1 [Source:HGNC Symbol;Acc:16818]
rs146304006	X	HDHD1	HGNC Symbol	haloacid dehalogenase-like hydrolase domain containing 1 [Source:HGNC Symbol;Acc:16818]
rs73192640	X	HDHD1	HGNC Symbol	haloacid dehalogenase-like hydrolase domain containing 1 [Source:HGNC Symbol;Acc:16818]
rs1131197	X	HDHD1	HGNC Symbol	haloacid dehalogenase-like hydrolase domain containing 1 [Source:HGNC Symbol;Acc:16818]
rs11095371	X	HDHD1	HGNC Symbol	haloacid dehalogenase-like hydrolase domain containing 1 [Source:HGNC Symbol;Acc:16818]
rs35569977	X	HDHD1	HGNC Symbol	haloacid dehalogenase-like hydrolase domain containing 1 [Source:HGNC Symbol;Acc:16818]
rs5934670				
rs2098072	X	STS	HGNC Symbol	steroid sulfatase (microsomal), isozyme S [Source:HGNC Symbol;Acc:11425]
rs5934911	X	STS	HGNC Symbol	steroid sulfatase (microsomal), isozyme S [Source:HGNC Symbol;Acc:11425]
rs5934914	X	STS	HGNC Symbol	steroid sulfatase (microsomal), isozyme S [Source:HGNC Symbol;Acc:11425]
rs5934916	X	STS	HGNC Symbol	steroid sulfatase (microsomal), isozyme S [Source:HGNC Symbol;Acc:11425]
rs7064866	X	STS	HGNC Symbol	steroid sulfatase (microsomal), isozyme S [Source:HGNC Symbol;Acc:11425]
rs10126096	X	STS	HGNC Symbol	steroid sulfatase (microsomal), isozyme S [Source:HGNC Symbol;Acc:11425]
rs6639825	X	STS	HGNC Symbol	steroid sulfatase (microsomal), isozyme S [Source:HGNC Symbol;Acc:11425]
rs141179442				
rs73194408				
rs7059918				
rs151122193				
rs5979458				
rs12013873				
rs17306481				
rs112727474				
rs5979531				
rs11796544				
rs5979595				
rs79703906				
rs7887063				
rs5978551				
rs5978567				

SNP	Chromosome	gene name	gene source	description
rs10856468				
rs12394082				
rs55958419				
rs6638698				
rs17306502				
rs10046958				
rs34265446				
rs62584319				
rs73463156				
rs11095468				
rs6638717				
rs12687581				
rs113729795				
rs66503313				
rs73194428				
rs11798180				
rs5980048				
rs4830517				
rs73194430				
rs12688598				
rs56290817				
rs113205466				
rs5980169				
rs1637794				
rs6639941				
rs12688485				
rs6530117				
rs6639942				
rs5936101				
rs1637788				
rs861506				
rs5934305				
rs140164218				

SNP	Chromosome	gene name	gene source	description
rs34709717	X	PNPLA4	HGNC Symbol	patatin-like phospholipase domain containing 4 [Source:HGNC Symbol;Acc:24887]
rs17310972	X	PNPLA4	HGNC Symbol	patatin-like phospholipase domain containing 4 [Source:HGNC Symbol;Acc:24887]
rs1179136	X	PNPLA4	HGNC Symbol	patatin-like phospholipase domain containing 4 [Source:HGNC Symbol;Acc:24887]
rs56158841				
rs2051996				
rs5978796				
rs145046321				
rs2701846				
rs6639985	X	RP11-692P14.1	Clone-based (Vega) gene	
rs5933625				
rs34586034				
rs7876124				
rs111810542				
rs5933631				
rs5933639				
rs115901692				
rs4830567				
rs35260390				
rs145934481				
rs4830389				
rs12559605				
rs1058239	X	VCX2	HGNC Symbol	variable charge, X-linked 2 [Source:HGNC Symbol;Acc:18158]
rs3761599				
rs55981626				
rs58394212				
rs6640009				
rs73194488				
rs145577051				
rs6640012				
rs143750253				
rs145425115				
rs7054042				

SNP	Chromosome	gene name	gene source	description
rs2872219				
rs6640059				
rs17260326				
rs6638789				
rs62584588				
rs5978854				
rs17306621				
rs66624612				
rs144203785				
rs73196762				
rs56182764				
rs2030886				
rs7887199				
rs7892812				
rs12689976				
rs950929				
rs6640134				
rs2404240				
rs6640144				
rs6640156				
rs5978885				
rs57759284				
rs148770494				
rs73196787				
rs73196792				
rs5934436				
rs808143				
rs808133				
rs5934442				
rs5978894	X	KAL1	HGNC Symbol	Kallmann syndrome 1 sequence [Source:HGNC Symbol;Acc:6211]
rs809446	X	KAL1	HGNC Symbol	Kallmann syndrome 1 sequence [Source:HGNC Symbol;Acc:6211]
rs808119	X	KAL1	HGNC Symbol	Kallmann syndrome 1 sequence [Source:HGNC Symbol;Acc:6211]
rs111809277	X	KAL1	HGNC Symbol	Kallmann syndrome 1 sequence [Source:HGNC Symbol;Acc:6211]

SNP	Chromosome	gene name	gene source	description
rs3788882	X	KAL1	HGNC Symbol	Kallmann syndrome 1 sequence [Source:HGNC Symbol;Acc:6211]
rs2284308	X	KAL1	HGNC Symbol	Kallmann syndrome 1 sequence [Source:HGNC Symbol;Acc:6211]
rs73199006	X	KAL1	HGNC Symbol	Kallmann syndrome 1 sequence [Source:HGNC Symbol;Acc:6211]
rs5933667	X	KAL1	HGNC Symbol	Kallmann syndrome 1 sequence [Source:HGNC Symbol;Acc:6211]
rs113309190	X	KAL1	HGNC Symbol	Kallmann syndrome 1 sequence [Source:HGNC Symbol;Acc:6211]
rs12007994	X	KAL1	HGNC Symbol	Kallmann syndrome 1 sequence [Source:HGNC Symbol;Acc:6211]
rs56167579	X	KAL1	HGNC Symbol	Kallmann syndrome 1 sequence [Source:HGNC Symbol;Acc:6211]
rs5934449	X	KAL1	HGNC Symbol	Kallmann syndrome 1 sequence [Source:HGNC Symbol;Acc:6211]
rs12710616	X	KAL1	HGNC Symbol	Kallmann syndrome 1 sequence [Source:HGNC Symbol;Acc:6211]
rs141578003	X	KAL1	HGNC Symbol	Kallmann syndrome 1 sequence [Source:HGNC Symbol;Acc:6211]
rs2214987	X	KAL1	HGNC Symbol	Kallmann syndrome 1 sequence [Source:HGNC Symbol;Acc:6211]
rs5978927	X	KAL1	HGNC Symbol	Kallmann syndrome 1 sequence [Source:HGNC Symbol;Acc:6211]
rs7878369	X	KAL1	HGNC Symbol	Kallmann syndrome 1 sequence [Source:HGNC Symbol;Acc:6211]
rs17306902	X	KAL1	HGNC Symbol	Kallmann syndrome 1 sequence [Source:HGNC Symbol;Acc:6211]
rs4295813	X	KAL1	HGNC Symbol	Kallmann syndrome 1 sequence [Source:HGNC Symbol;Acc:6211]
rs12014726	X	KAL1	HGNC Symbol	Kallmann syndrome 1 sequence [Source:HGNC Symbol;Acc:6211]
rs12007434	X	KAL1	HGNC Symbol	Kallmann syndrome 1 sequence [Source:HGNC Symbol;Acc:6211]
rs5934471	X	KAL1	HGNC Symbol	Kallmann syndrome 1 sequence [Source:HGNC Symbol;Acc:6211]
rs5934475	X	KAL1	HGNC Symbol	Kallmann syndrome 1 sequence [Source:HGNC Symbol;Acc:6211]
rs12008804	X	KAL1	HGNC Symbol	Kallmann syndrome 1 sequence [Source:HGNC Symbol;Acc:6211]
rs5978940	X	KAL1	HGNC Symbol	Kallmann syndrome 1 sequence [Source:HGNC Symbol;Acc:6211]
rs73199063	X	KAL1	HGNC Symbol	Kallmann syndrome 1 sequence [Source:HGNC Symbol;Acc:6211]
rs62584230	X	KAL1	HGNC Symbol	Kallmann syndrome 1 sequence [Source:HGNC Symbol;Acc:6211]
rs6640221	X	KAL1	HGNC Symbol	Kallmann syndrome 1 sequence [Source:HGNC Symbol;Acc:6211]
rs144420093				
rs73199085				
rs7883588				
rs79675415	X	FAM9A	HGNC Symbol	family with sequence similarity 9, member A [Source:HGNC Symbol;Acc:18403]
rs5933682				
rs56339333				
rs6530209				
rs56857867				
rs141340290				

SNP	Chromosome	gene name	gene source	description
rs929227				
rs12852869				
rs149537093				
rs1232141	X	GS1-542M4.4	Clone-based (Vega) gene	
rs113988989				
rs112741397				
rs1232110				
rs1232116				
rs5934488				
rs10521598				
rs1881687				
rs5934495				
rs17269655				
rs5934505				
rs17307280				
rs5934508				
rs2405111				
rs1316470				
rs1997345				
rs73631420				
rs5933701				
rs34693711				
rs16985316				
rs5933707				
rs1395790				
rs5934523				
rs10126241	X	FAM9B	HGNC Symbol	family with sequence similarity 9, member B [Source:HGNC Symbol;Acc:18404]
rs62581812	X	FAM9B	HGNC Symbol	family with sequence similarity 9, member B [Source:HGNC Symbol;Acc:18404]
rs6640275	X	FAM9B	HGNC Symbol	family with sequence similarity 9, member B [Source:HGNC Symbol;Acc:18404]
rs59504107	X	FAM9B	HGNC Symbol	family with sequence similarity 9, member B [Source:HGNC Symbol;Acc:18404]
rs113021379	X	FAM9B	HGNC Symbol	family with sequence similarity 9, member B [Source:HGNC Symbol;Acc:18404]
rs1466983	X	FAM9B	HGNC Symbol	family with sequence similarity 9, member B [Source:HGNC Symbol;Acc:18404]

SNP	Chromosome	gene name	gene source	description
rs6640298	X	FAM9B	HGNC Symbol	family with sequence similarity 9, member B [Source:HGNC Symbol;Acc:18404]
rs1466984	X	FAM9B	HGNC Symbol	family with sequence similarity 9, member B [Source:HGNC Symbol;Acc:18404]
rs11797090	X	FAM9B	HGNC Symbol	family with sequence similarity 9, member B [Source:HGNC Symbol;Acc:18404]
rs35912577	X	FAM9B	HGNC Symbol	family with sequence similarity 9, member B [Source:HGNC Symbol;Acc:18404]
rs10127383	X	FAM9B	HGNC Symbol	family with sequence similarity 9, member B [Source:HGNC Symbol;Acc:18404]
rs17222293	X	FAM9B	HGNC Symbol	family with sequence similarity 9, member B [Source:HGNC Symbol;Acc:18404]
rs1876416	X	FAM9B	HGNC Symbol	family with sequence similarity 9, member B [Source:HGNC Symbol;Acc:18404]
rs112502359	X	FAM9B	HGNC Symbol	family with sequence similarity 9, member B [Source:HGNC Symbol;Acc:18404]
rs7054846	X	FAM9B	HGNC Symbol	family with sequence similarity 9, member B [Source:HGNC Symbol;Acc:18404]
rs34111641	X	FAM9B	HGNC Symbol	family with sequence similarity 9, member B [Source:HGNC Symbol;Acc:18404]
rs41305345	X	FAM9B	HGNC Symbol	family with sequence similarity 9, member B [Source:HGNC Symbol;Acc:18404]
rs150482358				
rs7884606				
rs142159753				
rs6638901				
rs5979044				
rs2214281				
rs2106831				
rs2106832				
rs35996358				
rs60957056				
rs5934544				
rs5979050				
rs5979053				
rs41455048				
rs6638905				
rs5978307				
rs6640329				
rs6640333	X	GS1-519E5.1	Clone-based (Vega) gene	
rs142675995	X	GS1-519E5.1	Clone-based (Vega) gene	
rs5979065	X	GS1-519E5.1	Clone-based (Vega) gene	

SNP	Chromosome	gene name	gene source	description
rs36107887	X	GS1-519E5.1	Clone-based (Vega) gene	
rs4830624	X	GS1-519E5.1	Clone-based (Vega) gene	
rs5934550	X	GS1-519E5.1	Clone-based (Vega) gene	
rs5934551	X	GS1-519E5.1	Clone-based (Vega) gene	
rs7049698				
rs2070				
rs1024443				
rs2023813				
rs12008348				
rs5934556				
rs4997204				
rs16985418				
rs5933723				
rs16985424				
rs12013939				
rs73186314				
rs6419022				
rs4830632				
rs5934564				
rs2405536				
rs5934574				
rs62583434				
rs5933729				
rs73186324				
rs12558473				
rs7877005				
rs4830637				
rs4830639				
rs6640370				
rs715171				

SNP	Chromosome	gene name	gene source	description
rs35895403				
rs13362938				
rs111403073				
rs5934597				
rs6530275				
rs60102760				
rs6640375	X	RP11-126O22.1	Clone-based (Vega) gene	
rs5934606	X	RP11-126O22.1	Clone-based (Vega) gene	
rs17320944				
rs5933734				
rs73186342				
rs6530299				
rs11798110				
rs56750973				
rs146962044				
rs1526421				
rs5934627				
rs4448458				
rs5934630				
rs5741879				
rs55812283				
rs5934617				
rs7878827	X	TBL1X	HGNC Symbol	transducin (beta)-like 1X-linked [Source:HGNC Symbol;Acc:11585]
rs2521395	X	TBL1X	HGNC Symbol	transducin (beta)-like 1X-linked [Source:HGNC Symbol;Acc:11585]
rs886193	X	TBL1X	HGNC Symbol	transducin (beta)-like 1X-linked [Source:HGNC Symbol;Acc:11585]
rs2521387	X	TBL1X	HGNC Symbol	transducin (beta)-like 1X-linked [Source:HGNC Symbol;Acc:11585]
rs2681642	X	TBL1X	HGNC Symbol	transducin (beta)-like 1X-linked [Source:HGNC Symbol;Acc:11585]
rs16998739	X	TBL1X	HGNC Symbol	transducin (beta)-like 1X-linked [Source:HGNC Symbol;Acc:11585]
rs2066887	X	TBL1X	HGNC Symbol	transducin (beta)-like 1X-linked [Source:HGNC Symbol;Acc:11585]
rs2521415	X	TBL1X	HGNC Symbol	transducin (beta)-like 1X-linked [Source:HGNC Symbol;Acc:11585]
rs7063680	X	TBL1X	HGNC Symbol	transducin (beta)-like 1X-linked [Source:HGNC Symbol;Acc:11585]
rs2238854	X	TBL1X	HGNC Symbol	transducin (beta)-like 1X-linked [Source:HGNC Symbol;Acc:11585]

SNP	Chromosome	gene name	gene source	description
rs113738212	X	TBL1X	HGNC Symbol	transducin (beta)-like 1X-linked [Source:HGNC Symbol;Acc:11585]
rs73186396	X	TBL1X	HGNC Symbol	transducin (beta)-like 1X-linked [Source:HGNC Symbol;Acc:11585]
rs2521413	X	TBL1X	HGNC Symbol	transducin (beta)-like 1X-linked [Source:HGNC Symbol;Acc:11585]
rs2681646	X	TBL1X	HGNC Symbol	transducin (beta)-like 1X-linked [Source:HGNC Symbol;Acc:11585]
rs5934650	X	TBL1X	HGNC Symbol	transducin (beta)-like 1X-linked [Source:HGNC Symbol;Acc:11585]
rs2238860	X	TBL1X	HGNC Symbol	transducin (beta)-like 1X-linked [Source:HGNC Symbol;Acc:11585]
rs12393928	X	TBL1X	HGNC Symbol	transducin (beta)-like 1X-linked [Source:HGNC Symbol;Acc:11585]
rs5934655	X	TBL1X	HGNC Symbol	transducin (beta)-like 1X-linked [Source:HGNC Symbol;Acc:11585]
rs2285577	X	TBL1X	HGNC Symbol	transducin (beta)-like 1X-linked [Source:HGNC Symbol;Acc:11585]
rs28704564	X	TBL1X	HGNC Symbol	transducin (beta)-like 1X-linked [Source:HGNC Symbol;Acc:11585]
rs55976424	X	TBL1X	HGNC Symbol	transducin (beta)-like 1X-linked [Source:HGNC Symbol;Acc:11585]
rs2521402	X	TBL1X	HGNC Symbol	transducin (beta)-like 1X-linked [Source:HGNC Symbol;Acc:11585]
rs35465421	X	TBL1X	HGNC Symbol	transducin (beta)-like 1X-linked [Source:HGNC Symbol;Acc:11585]
rs73632608	X	TBL1X	HGNC Symbol	transducin (beta)-like 1X-linked [Source:HGNC Symbol;Acc:11585]
rs151045693	X	TBL1X	HGNC Symbol	transducin (beta)-like 1X-linked [Source:HGNC Symbol;Acc:11585]
rs2074033	X	TBL1X	HGNC Symbol	transducin (beta)-like 1X-linked [Source:HGNC Symbol;Acc:11585]
rs2023750	X	TBL1X	HGNC Symbol	transducin (beta)-like 1X-linked [Source:HGNC Symbol;Acc:11585]
rs73188211	X	TBL1X	HGNC Symbol	transducin (beta)-like 1X-linked [Source:HGNC Symbol;Acc:11585]
rs2023749	X	TBL1X	HGNC Symbol	transducin (beta)-like 1X-linked [Source:HGNC Symbol;Acc:11585]
rs16985632	X	TBL1X	HGNC Symbol	transducin (beta)-like 1X-linked [Source:HGNC Symbol;Acc:11585]
rs2040616	X	TBL1X	HGNC Symbol	transducin (beta)-like 1X-linked [Source:HGNC Symbol;Acc:11585]
rs17321050	X	TBL1X	HGNC Symbol	transducin (beta)-like 1X-linked [Source:HGNC Symbol;Acc:11585]
rs73188221	X	TBL1X	HGNC Symbol	transducin (beta)-like 1X-linked [Source:HGNC Symbol;Acc:11585]
rs2188766	X	TBL1X	HGNC Symbol	transducin (beta)-like 1X-linked [Source:HGNC Symbol;Acc:11585]
rs2521590	X	TBL1X	HGNC Symbol	transducin (beta)-like 1X-linked [Source:HGNC Symbol;Acc:11585]
rs73188228	X	TBL1X	HGNC Symbol	transducin (beta)-like 1X-linked [Source:HGNC Symbol;Acc:11585]
rs2521584	X	TBL1X	HGNC Symbol	transducin (beta)-like 1X-linked [Source:HGNC Symbol;Acc:11585]
rs16985657	X	TBL1X	HGNC Symbol	transducin (beta)-like 1X-linked [Source:HGNC Symbol;Acc:11585]
rs17321092	X	TBL1X	HGNC Symbol	transducin (beta)-like 1X-linked [Source:HGNC Symbol;Acc:11585]
rs2732885	X	TBL1X	HGNC Symbol	transducin (beta)-like 1X-linked [Source:HGNC Symbol;Acc:11585]
rs2732886	X	TBL1X	HGNC Symbol	transducin (beta)-like 1X-linked [Source:HGNC Symbol;Acc:11585]
rs5933751	X	TBL1X	HGNC Symbol	transducin (beta)-like 1X-linked [Source:HGNC Symbol;Acc:11585]
rs73188239	X	TBL1X	HGNC Symbol	transducin (beta)-like 1X-linked [Source:HGNC Symbol;Acc:11585]

SNP	Chromosome	gene name	gene source	description
rs2239411	X	TBL1X	HGNC Symbol	transducin (beta)-like 1X-linked [Source:HGNC Symbol;Acc:11585]
rs2239413	X	TBL1X	HGNC Symbol	transducin (beta)-like 1X-linked [Source:HGNC Symbol;Acc:11585]
rs2521582	X	TBL1X	HGNC Symbol	transducin (beta)-like 1X-linked [Source:HGNC Symbol;Acc:11585]
rs17321113	X	TBL1X	HGNC Symbol	transducin (beta)-like 1X-linked [Source:HGNC Symbol;Acc:11585]
rs111372317	X	TBL1X	HGNC Symbol	transducin (beta)-like 1X-linked [Source:HGNC Symbol;Acc:11585]
rs5979155	X	TBL1X	HGNC Symbol	transducin (beta)-like 1X-linked [Source:HGNC Symbol;Acc:11585]
rs733071	X	TBL1X	HGNC Symbol	transducin (beta)-like 1X-linked [Source:HGNC Symbol;Acc:11585]
rs1006912	X	TBL1X	HGNC Symbol	transducin (beta)-like 1X-linked [Source:HGNC Symbol;Acc:11585]
rs16985668	X	TBL1X	HGNC Symbol	transducin (beta)-like 1X-linked [Source:HGNC Symbol;Acc:11585]
rs17321120	X	TBL1X	HGNC Symbol	transducin (beta)-like 1X-linked [Source:HGNC Symbol;Acc:11585]
rs7256	X	TBL1X	HGNC Symbol	transducin (beta)-like 1X-linked [Source:HGNC Symbol;Acc:11585]
rs10126515				
rs6638956				
rs2521578	X	GPR143	HGNC Symbol	G protein-coupled receptor 143 [Source:HGNC Symbol;Acc:20145]
rs142823952	X	GPR143	HGNC Symbol	G protein-coupled receptor 143 [Source:HGNC Symbol;Acc:20145]
rs6530311	X	GPR143	HGNC Symbol	G protein-coupled receptor 143 [Source:HGNC Symbol;Acc:20145]
rs5979168	X	GPR143	HGNC Symbol	G protein-coupled receptor 143 [Source:HGNC Symbol;Acc:20145]
rs17255230	X	GPR143	HGNC Symbol	G protein-coupled receptor 143 [Source:HGNC Symbol;Acc:20145]
rs41305347	X	GPR143	HGNC Symbol	G protein-coupled receptor 143 [Source:HGNC Symbol;Acc:20145]
rs11796366	X	GPR143	HGNC Symbol	G protein-coupled receptor 143 [Source:HGNC Symbol;Acc:20145]
rs62581876	X	GPR143	HGNC Symbol	G protein-coupled receptor 143 [Source:HGNC Symbol;Acc:20145]
rs5934679	X	GPR143	HGNC Symbol	G protein-coupled receptor 143 [Source:HGNC Symbol;Acc:20145]
rs2521652	X	GPR143	HGNC Symbol	G protein-coupled receptor 143 [Source:HGNC Symbol;Acc:20145]
rs4830656	X	GPR143	HGNC Symbol	G protein-coupled receptor 143 [Source:HGNC Symbol;Acc:20145]
rs929217	X	GPR143	HGNC Symbol	G protein-coupled receptor 143 [Source:HGNC Symbol;Acc:20145]
rs73194156	X	GPR143	HGNC Symbol	G protein-coupled receptor 143 [Source:HGNC Symbol;Acc:20145]
rs2521659	X	GPR143	HGNC Symbol	G protein-coupled receptor 143 [Source:HGNC Symbol;Acc:20145]
rs5934683	X	GPR143	HGNC Symbol	G protein-coupled receptor 143 [Source:HGNC Symbol;Acc:20145]
rs757018	X	SHROOM2	HGNC Symbol	shroom family member 2 [Source:HGNC Symbol;Acc:630]
rs59296266	X	SHROOM2	HGNC Symbol	shroom family member 2 [Source:HGNC Symbol;Acc:630]
rs2521665	X	SHROOM2	HGNC Symbol	shroom family member 2 [Source:HGNC Symbol;Acc:630]
rs5934685	X	SHROOM2	HGNC Symbol	shroom family member 2 [Source:HGNC Symbol;Acc:630]
rs146927570	X	SHROOM2	HGNC Symbol	shroom family member 2 [Source:HGNC Symbol;Acc:630]

SNP	Chromosome	gene name	gene source	description
rs192264728	X	SHROOM2	HGNC Symbol	shroom family member 2 [Source:HGNC Symbol;Acc:630]
rs5934690	X	SHROOM2;RP11-98L4.1	HGNC Symbol;Clone-based (Vega) gene	shroom family member 2 [Source:HGNC Symbol;Acc:630];
rs140442601	X	SHROOM2	HGNC Symbol	shroom family member 2 [Source:HGNC Symbol;Acc:630]
rs116074556	X	SHROOM2	HGNC Symbol	shroom family member 2 [Source:HGNC Symbol;Acc:630]
rs5934693	X	SHROOM2	HGNC Symbol	shroom family member 2 [Source:HGNC Symbol;Acc:630]
rs6638968	X	SHROOM2	HGNC Symbol	shroom family member 2 [Source:HGNC Symbol;Acc:630]
rs2405942	X	SHROOM2	HGNC Symbol	shroom family member 2 [Source:HGNC Symbol;Acc:630]
rs5933765	X	SHROOM2	HGNC Symbol	shroom family member 2 [Source:HGNC Symbol;Acc:630]
rs16985744	X	SHROOM2	HGNC Symbol	shroom family member 2 [Source:HGNC Symbol;Acc:630]
rs17255244	X	SHROOM2	HGNC Symbol	shroom family member 2 [Source:HGNC Symbol;Acc:630]
rs5933772	X	SHROOM2	HGNC Symbol	shroom family member 2 [Source:HGNC Symbol;Acc:630]
rs4830665	X	SHROOM2	HGNC Symbol	shroom family member 2 [Source:HGNC Symbol;Acc:630]
rs12557532	X	SHROOM2	HGNC Symbol	shroom family member 2 [Source:HGNC Symbol;Acc:630]
rs5979199	X	SHROOM2	HGNC Symbol	shroom family member 2 [Source:HGNC Symbol;Acc:630]
rs184371255	X	SHROOM2	HGNC Symbol	shroom family member 2 [Source:HGNC Symbol;Acc:630]
rs1076167	X	SHROOM2	HGNC Symbol	shroom family member 2 [Source:HGNC Symbol;Acc:630]
rs73195964	X	SHROOM2	HGNC Symbol	shroom family member 2 [Source:HGNC Symbol;Acc:630]
rs2405947	X	SHROOM2	HGNC Symbol	shroom family member 2 [Source:HGNC Symbol;Acc:630]
rs5979202	X	SHROOM2	HGNC Symbol	shroom family member 2 [Source:HGNC Symbol;Acc:630]
rs5978349	X	SHROOM2	HGNC Symbol	shroom family member 2 [Source:HGNC Symbol;Acc:630]
rs41302635	X	SHROOM2	HGNC Symbol	shroom family member 2 [Source:HGNC Symbol;Acc:630]
rs2239422	X	SHROOM2	HGNC Symbol	shroom family member 2 [Source:HGNC Symbol;Acc:630]
rs2239425	X	SHROOM2	HGNC Symbol	shroom family member 2 [Source:HGNC Symbol;Acc:630]
rs5934719	X	SHROOM2	HGNC Symbol	shroom family member 2 [Source:HGNC Symbol;Acc:630]
rs79628572	X	SHROOM2	HGNC Symbol	shroom family member 2 [Source:HGNC Symbol;Acc:630]
rs3788951	X	SHROOM2	HGNC Symbol	shroom family member 2 [Source:HGNC Symbol;Acc:630]
rs4830679	X	SHROOM2	HGNC Symbol	shroom family member 2 [Source:HGNC Symbol;Acc:630]
rs2073942	X	SHROOM2	HGNC Symbol	shroom family member 2 [Source:HGNC Symbol;Acc:630]
rs886128	X	SHROOM2	HGNC Symbol	shroom family member 2 [Source:HGNC Symbol;Acc:630]
rs17321225				
rs66520508				
rs4830427				

SNP	Chromosome	gene name	gene source	description
rs5979217				
rs2188385				
rs73476680				
rs5979220				
rs11095524				
rs150044602				
rs734543				
rs6640579				
rs5934730	X	AC002365.1	Clone-based (Ensembl) gene	Homo sapiens uncharacterized LOC100288814 (LOC100288814), mRNA. [Source:RefSeq mRNA;Acc:NM_001195081]
rs5934731	X	AC002365.1	Clone-based (Ensembl) gene	Homo sapiens uncharacterized LOC100288814 (LOC100288814), mRNA. [Source:RefSeq mRNA;Acc:NM_001195081]
rs34664625				
rs4830432				
rs5979224				
rs151196781				
rs73195991	X	WWC3	HGNC Symbol	WWC family member 3 [Source:HGNC Symbol;Acc:29237]
rs5934741	X	WWC3;WWC3-AS1	HGNC Symbol	WWC family member 3 [Source:HGNC Symbol;Acc:29237];WWC3 antisense RNA 1 [Source:HGNC Symbol;Acc:41236]
rs7880027	X	WWC3	HGNC Symbol	WWC family member 3 [Source:HGNC Symbol;Acc:29237]
rs146705392	X	WWC3	HGNC Symbol	WWC family member 3 [Source:HGNC Symbol;Acc:29237]
rs16985882	X	WWC3	HGNC Symbol	WWC family member 3 [Source:HGNC Symbol;Acc:29237]
rs4830691	X	WWC3	HGNC Symbol	WWC family member 3 [Source:HGNC Symbol;Acc:29237]
rs6638990	X	WWC3	HGNC Symbol	WWC family member 3 [Source:HGNC Symbol;Acc:29237]
rs9792752	X	WWC3	HGNC Symbol	WWC family member 3 [Source:HGNC Symbol;Acc:29237]
rs7879307	X	WWC3	HGNC Symbol	WWC family member 3 [Source:HGNC Symbol;Acc:29237]
rs5933801	X	WWC3	HGNC Symbol	WWC family member 3 [Source:HGNC Symbol;Acc:29237]
rs1476467	X	WWC3	HGNC Symbol	WWC family member 3 [Source:HGNC Symbol;Acc:29237]
rs4830695	X	WWC3	HGNC Symbol	WWC family member 3 [Source:HGNC Symbol;Acc:29237]
rs12013058	X	WWC3	HGNC Symbol	WWC family member 3 [Source:HGNC Symbol;Acc:29237]
rs111364242	X	WWC3	HGNC Symbol	WWC family member 3 [Source:HGNC Symbol;Acc:29237]
rs5934750	X	WWC3	HGNC Symbol	WWC family member 3 [Source:HGNC Symbol;Acc:29237]
rs5979253	X	WWC3	HGNC Symbol	WWC family member 3 [Source:HGNC Symbol;Acc:29237]
rs739485	X	WWC3	HGNC Symbol	WWC family member 3 [Source:HGNC Symbol;Acc:29237]

SNP	Chromosome	gene name	gene source	description
rs756828	X	WWC3	HGNC Symbol	WWC family member 3 [Source:HGNC Symbol;Acc:29237]
rs5934754	X	WWC3	HGNC Symbol	WWC family member 3 [Source:HGNC Symbol;Acc:29237]
rs5933804	X	WWC3	HGNC Symbol	WWC family member 3 [Source:HGNC Symbol;Acc:29237]
rs73197804	X	WWC3	HGNC Symbol	WWC family member 3 [Source:HGNC Symbol;Acc:29237]
rs5978376	X	WWC3	HGNC Symbol	WWC family member 3 [Source:HGNC Symbol;Acc:29237]
rs112535032				
rs4830437				
rs7885148				
rs5934772				
rs4636360				
rs3810739				
rs41309719	X	CLCN4	HGNC Symbol	chloride channel, voltage-sensitive 4 [Source:HGNC Symbol;Acc:2022]
rs2239938	X	CLCN4	HGNC Symbol	chloride channel, voltage-sensitive 4 [Source:HGNC Symbol;Acc:2022]
rs17255411	X	CLCN4	HGNC Symbol	chloride channel, voltage-sensitive 4 [Source:HGNC Symbol;Acc:2022]
rs5979272	X	CLCN4	HGNC Symbol	chloride channel, voltage-sensitive 4 [Source:HGNC Symbol;Acc:2022]
rs16985984	X	CLCN4	HGNC Symbol	chloride channel, voltage-sensitive 4 [Source:HGNC Symbol;Acc:2022]
rs5979274	X	CLCN4	HGNC Symbol	chloride channel, voltage-sensitive 4 [Source:HGNC Symbol;Acc:2022]
rs5934810	X	CLCN4	HGNC Symbol	chloride channel, voltage-sensitive 4 [Source:HGNC Symbol;Acc:2022]
rs1003368	X	CLCN4	HGNC Symbol	chloride channel, voltage-sensitive 4 [Source:HGNC Symbol;Acc:2022]
rs2301691	X	CLCN4	HGNC Symbol	chloride channel, voltage-sensitive 4 [Source:HGNC Symbol;Acc:2022]
rs7065191	X	CLCN4	HGNC Symbol	chloride channel, voltage-sensitive 4 [Source:HGNC Symbol;Acc:2022]
rs2078275	X	CLCN4	HGNC Symbol	chloride channel, voltage-sensitive 4 [Source:HGNC Symbol;Acc:2022]
rs1000670	X	CLCN4	HGNC Symbol	chloride channel, voltage-sensitive 4 [Source:HGNC Symbol;Acc:2022]
rs11795877	X	CLCN4	HGNC Symbol	chloride channel, voltage-sensitive 4 [Source:HGNC Symbol;Acc:2022]
rs55885942	X	CLCN4	HGNC Symbol	chloride channel, voltage-sensitive 4 [Source:HGNC Symbol;Acc:2022]
rs41404246	X	CLCN4	HGNC Symbol	chloride channel, voltage-sensitive 4 [Source:HGNC Symbol;Acc:2022]
rs5934812	X	CLCN4	HGNC Symbol	chloride channel, voltage-sensitive 4 [Source:HGNC Symbol;Acc:2022]
rs67583581	X	CLCN4	HGNC Symbol	chloride channel, voltage-sensitive 4 [Source:HGNC Symbol;Acc:2022]
rs2269815	X	CLCN4	HGNC Symbol	chloride channel, voltage-sensitive 4 [Source:HGNC Symbol;Acc:2022]
rs5934817	X	CLCN4	HGNC Symbol	chloride channel, voltage-sensitive 4 [Source:HGNC Symbol;Acc:2022]
rs5933828	X	RP6-1O2.1	Clone-based (Vega) gene	
rs142115074	X	RP6-1O2.1	Clone-based (Vega) gene	

SNP	Chromosome	gene name	gene source	description
rs2012195	X	RP6-1O2.1	Clone-based (Vega) gene	
rs5934830	X	RP6-1O2.1	Clone-based (Vega) gene	
rs12388359	X	RP6-1O2.1	Clone-based (Vega) gene	
rs2188900	X	RP6-1O2.1	Clone-based (Vega) gene	
rs58508864	X	RP6-1O2.1	Clone-based (Vega) gene	
rs5933832	X	RP6-1O2.1	Clone-based (Vega) gene	
rs1012782	X	RP6-1O2.1	Clone-based (Vega) gene	
rs73491141	X	RP6-1O2.1	Clone-based (Vega) gene	
rs17327648	X	RP6-1O2.1	Clone-based (Vega) gene	
rs2406413	X	RP6-1O2.1	Clone-based (Vega) gene	
rs113978610	X	RP6-1O2.1	Clone-based (Vega) gene	
rs5934884	X	RP6-1O2.1	Clone-based (Vega) gene	
rs5979305				
rs5934902				
rs7877277				
rs5978399				
rs7054879				
rs17281018				
rs111433690				
rs6640640				
rs73202007	X	MID1	HGNC Symbol	midline 1 (Opitz/BBB syndrome) [Source:HGNC Symbol;Acc:7095]
rs5934908	X	MID1	HGNC Symbol	midline 1 (Opitz/BBB syndrome) [Source:HGNC Symbol;Acc:7095]
rs7891302	X	MID1	HGNC Symbol	midline 1 (Opitz/BBB syndrome) [Source:HGNC Symbol;Acc:7095]
rs145697841	X	MID1	HGNC Symbol	midline 1 (Opitz/BBB syndrome) [Source:HGNC Symbol;Acc:7095]
rs73202011	X	MID1	HGNC Symbol	midline 1 (Opitz/BBB syndrome) [Source:HGNC Symbol;Acc:7095]

SNP	Chromosome	gene name	gene source	description
rs7065297	X	MID1	HGNC Symbol	midline 1 (Opitz/BBB syndrome) [Source:HGNC Symbol;Acc:7095]
rs61536731	X	MID1	HGNC Symbol	midline 1 (Opitz/BBB syndrome) [Source:HGNC Symbol;Acc:7095]
rs2464658	X	MID1	HGNC Symbol	midline 1 (Opitz/BBB syndrome) [Source:HGNC Symbol;Acc:7095]
rs73202012	X	MID1	HGNC Symbol	midline 1 (Opitz/BBB syndrome) [Source:HGNC Symbol;Acc:7095]
rs143035204	X	MID1	HGNC Symbol	midline 1 (Opitz/BBB syndrome) [Source:HGNC Symbol;Acc:7095]
rs35027532	X	MID1	HGNC Symbol	midline 1 (Opitz/BBB syndrome) [Source:HGNC Symbol;Acc:7095]
rs869917	X	MID1	HGNC Symbol	midline 1 (Opitz/BBB syndrome) [Source:HGNC Symbol;Acc:7095]
rs2525070	X	MID1	HGNC Symbol	midline 1 (Opitz/BBB syndrome) [Source:HGNC Symbol;Acc:7095]
rs138340584	X	MID1	HGNC Symbol	midline 1 (Opitz/BBB syndrome) [Source:HGNC Symbol;Acc:7095]
rs12164413	X	MID1	HGNC Symbol	midline 1 (Opitz/BBB syndrome) [Source:HGNC Symbol;Acc:7095]
rs2428595	X	MID1	HGNC Symbol	midline 1 (Opitz/BBB syndrome) [Source:HGNC Symbol;Acc:7095]
rs16986210	X	MID1	HGNC Symbol	midline 1 (Opitz/BBB syndrome) [Source:HGNC Symbol;Acc:7095]
rs1548520	X	MID1	HGNC Symbol	midline 1 (Opitz/BBB syndrome) [Source:HGNC Symbol;Acc:7095]
rs11796084	X	MID1	HGNC Symbol	midline 1 (Opitz/BBB syndrome) [Source:HGNC Symbol;Acc:7095]
rs12394761	X	MID1	HGNC Symbol	midline 1 (Opitz/BBB syndrome) [Source:HGNC Symbol;Acc:7095]
rs16986252	X	MID1	HGNC Symbol	midline 1 (Opitz/BBB syndrome) [Source:HGNC Symbol;Acc:7095]
rs960420	X	MID1	HGNC Symbol	midline 1 (Opitz/BBB syndrome) [Source:HGNC Symbol;Acc:7095]
rs62589986	X	MID1	HGNC Symbol	midline 1 (Opitz/BBB syndrome) [Source:HGNC Symbol;Acc:7095]
rs147699219	X	MID1	HGNC Symbol	midline 1 (Opitz/BBB syndrome) [Source:HGNC Symbol;Acc:7095]
rs11095543	X	MID1	HGNC Symbol	midline 1 (Opitz/BBB syndrome) [Source:HGNC Symbol;Acc:7095]
rs7878745	X	MID1	HGNC Symbol	midline 1 (Opitz/BBB syndrome) [Source:HGNC Symbol;Acc:7095]
rs974582	X	MID1	HGNC Symbol	midline 1 (Opitz/BBB syndrome) [Source:HGNC Symbol;Acc:7095]
rs974581	X	MID1	HGNC Symbol	midline 1 (Opitz/BBB syndrome) [Source:HGNC Symbol;Acc:7095]
rs147497687	X	MID1	HGNC Symbol	midline 1 (Opitz/BBB syndrome) [Source:HGNC Symbol;Acc:7095]
rs11796037	X	MID1	HGNC Symbol	midline 1 (Opitz/BBB syndrome) [Source:HGNC Symbol;Acc:7095]
rs184989752	X	MID1	HGNC Symbol	midline 1 (Opitz/BBB syndrome) [Source:HGNC Symbol;Acc:7095]
rs35991810	X	MID1	HGNC Symbol	midline 1 (Opitz/BBB syndrome) [Source:HGNC Symbol;Acc:7095]
rs12845846				
rs17255516				
rs5978423	X	RP11-120D5.1	Clone-based (Vega) gene	
rs6530417	X	RP11-120D5.1	Clone-based (Vega) gene	

SNP	Chromosome	gene name	gene source	description
rs763696	X	RP11-120D5.1	Clone-based (Vega) gene	
rs1799436	X	RP11-120D5.1	Clone-based (Vega) gene	
rs73202039	X	RP11-120D5.1	Clone-based (Vega) gene	
rs73202040	X	RP11-120D5.1	Clone-based (Vega) gene	
rs5934953	X	RP11-120D5.1	Clone-based (Vega) gene	
rs6640703	X	RP11-120D5.1	Clone-based (Vega) gene	
rs2070163	X	HCCS	HGNC Symbol	holocytochrome c synthase [Source:HGNC Symbol;Acc:4837]
rs6640710	X	ARHGAP6	HGNC Symbol	Rho GTPase activating protein 6 [Source:HGNC Symbol;Acc:676]
rs5979372	X	ARHGAP6	HGNC Symbol	Rho GTPase activating protein 6 [Source:HGNC Symbol;Acc:676]
rs41471549	X	ARHGAP6	HGNC Symbol	Rho GTPase activating protein 6 [Source:HGNC Symbol;Acc:676]
rs28625269	X	ARHGAP6	HGNC Symbol	Rho GTPase activating protein 6 [Source:HGNC Symbol;Acc:676]
rs17321440	X	ARHGAP6	HGNC Symbol	Rho GTPase activating protein 6 [Source:HGNC Symbol;Acc:676]
rs66887330	X	ARHGAP6	HGNC Symbol	Rho GTPase activating protein 6 [Source:HGNC Symbol;Acc:676]
rs10521616	X	ARHGAP6	HGNC Symbol	Rho GTPase activating protein 6 [Source:HGNC Symbol;Acc:676]
rs73184322	X	ARHGAP6	HGNC Symbol	Rho GTPase activating protein 6 [Source:HGNC Symbol;Acc:676]
rs66511610	X	ARHGAP6	HGNC Symbol	Rho GTPase activating protein 6 [Source:HGNC Symbol;Acc:676]
rs5934984	X	ARHGAP6	HGNC Symbol	Rho GTPase activating protein 6 [Source:HGNC Symbol;Acc:676]
rs73184326	X	ARHGAP6	HGNC Symbol	Rho GTPase activating protein 6 [Source:HGNC Symbol;Acc:676]
rs144645613	X	ARHGAP6	HGNC Symbol	Rho GTPase activating protein 6 [Source:HGNC Symbol;Acc:676]
rs17255559	X	ARHGAP6	HGNC Symbol	Rho GTPase activating protein 6 [Source:HGNC Symbol;Acc:676]
rs55984432	X	ARHGAP6	HGNC Symbol	Rho GTPase activating protein 6 [Source:HGNC Symbol;Acc:676]
rs946252	X	ARHGAP6;AMELX	HGNC Symbol	Rho GTPase activating protein 6 [Source:HGNC Symbol;Acc:676];amelogenin, X-linked [Source:HGNC Symbol;Acc:461]
rs149536248	X	ARHGAP6	HGNC Symbol	Rho GTPase activating protein 6 [Source:HGNC Symbol;Acc:676]
rs35887552	X	ARHGAP6	HGNC Symbol	Rho GTPase activating protein 6 [Source:HGNC Symbol;Acc:676]
rs6640729	X	ARHGAP6	HGNC Symbol	Rho GTPase activating protein 6 [Source:HGNC Symbol;Acc:676]
rs1265863	X	ARHGAP6	HGNC Symbol	Rho GTPase activating protein 6 [Source:HGNC Symbol;Acc:676]
rs12850174	X	ARHGAP6	HGNC Symbol	Rho GTPase activating protein 6 [Source:HGNC Symbol;Acc:676]
rs17255587	X	ARHGAP6	HGNC Symbol	Rho GTPase activating protein 6 [Source:HGNC Symbol;Acc:676]

SNP	Chromosome	gene name	gene source	description
rs12559362	X	ARHGAP6	HGNC Symbol	Rho GTPase activating protein 6 [Source:HGNC Symbol;Acc:676]
rs55782070	X	ARHGAP6	HGNC Symbol	Rho GTPase activating protein 6 [Source:HGNC Symbol;Acc:676]
rs149582841	X	ARHGAP6	HGNC Symbol	Rho GTPase activating protein 6 [Source:HGNC Symbol;Acc:676]
rs17281073	X	ARHGAP6	HGNC Symbol	Rho GTPase activating protein 6 [Source:HGNC Symbol;Acc:676]
rs5935081	X	ARHGAP6	HGNC Symbol	Rho GTPase activating protein 6 [Source:HGNC Symbol;Acc:676]
rs34844588	X	ARHGAP6	HGNC Symbol	Rho GTPase activating protein 6 [Source:HGNC Symbol;Acc:676]
rs5935088	X	ARHGAP6	HGNC Symbol	Rho GTPase activating protein 6 [Source:HGNC Symbol;Acc:676]
rs62587974	X	ARHGAP6	HGNC Symbol	Rho GTPase activating protein 6 [Source:HGNC Symbol;Acc:676]
rs146802796	X	ARHGAP6	HGNC Symbol	Rho GTPase activating protein 6 [Source:HGNC Symbol;Acc:676]
rs5933896	X	ARHGAP6	HGNC Symbol	Rho GTPase activating protein 6 [Source:HGNC Symbol;Acc:676]
rs4830738	X	ARHGAP6	HGNC Symbol	Rho GTPase activating protein 6 [Source:HGNC Symbol;Acc:676]
rs5978438	X	ARHGAP6	HGNC Symbol	Rho GTPase activating protein 6 [Source:HGNC Symbol;Acc:676]
rs141052421	X	ARHGAP6	HGNC Symbol	Rho GTPase activating protein 6 [Source:HGNC Symbol;Acc:676]
rs7350366	X	ARHGAP6	HGNC Symbol	Rho GTPase activating protein 6 [Source:HGNC Symbol;Acc:676]
rs6654942	X	ARHGAP6	HGNC Symbol	Rho GTPase activating protein 6 [Source:HGNC Symbol;Acc:676]
rs5979429	X	ARHGAP6	HGNC Symbol	Rho GTPase activating protein 6 [Source:HGNC Symbol;Acc:676]
rs67365912	X	ARHGAP6	HGNC Symbol	Rho GTPase activating protein 6 [Source:HGNC Symbol;Acc:676]
rs4344257	X	ARHGAP6	HGNC Symbol	Rho GTPase activating protein 6 [Source:HGNC Symbol;Acc:676]
rs12116291				
rs6640760				
rs2716092				
rs2525877	X	GS1-590J6.2	Clone-based (Vega) gene	
rs5933917				
rs5979471				
rs2023901				
rs55776920				
rs60220870				
rs2716094				
rs55732427				
rs12558575	X	MSL3	HGNC Symbol	male-specific lethal 3 homolog (Drosophila) [Source:HGNC Symbol;Acc:7370]
rs17281129				
rs2716101				

SNP	Chromosome	gene name	gene source	description
rs858080				
rs2525870				
rs1231541				
rs708467				
rs17321544				
rs5935176				
rs4830454				
rs150022844				
rs17255656				
rs2091417				
rs2579848				
rs5979495				
rs5935203				
rs1151484				
rs10521620				
rs73190517				
rs6640855				
rs1231461				
rs857533				
rs6640876				
rs1526790				
rs5935236				
rs149506334				
rs10449070				
rs1076176				
rs857340				
rs62591581	X	FRMPD4	HGNC Symbol	FERM and PDZ domain containing 4 [Source:HGNC Symbol;Acc:29007]
rs115430707	X	FRMPD4	HGNC Symbol	FERM and PDZ domain containing 4 [Source:HGNC Symbol;Acc:29007]
rs6640915	X	FRMPD4	HGNC Symbol	FERM and PDZ domain containing 4 [Source:HGNC Symbol;Acc:29007]
rs6640922	X	FRMPD4	HGNC Symbol	FERM and PDZ domain containing 4 [Source:HGNC Symbol;Acc:29007]
rs55727510	X	FRMPD4	HGNC Symbol	FERM and PDZ domain containing 4 [Source:HGNC Symbol;Acc:29007]
rs66948843	X	FRMPD4	HGNC Symbol	FERM and PDZ domain containing 4 [Source:HGNC Symbol;Acc:29007]
rs12838825	X	FRMPD4	HGNC Symbol	FERM and PDZ domain containing 4 [Source:HGNC Symbol;Acc:29007]

SNP	Chromosome	gene name	gene source	description
rs5935271	X	FRMPD4	HGNC Symbol	FERM and PDZ domain containing 4 [Source:HGNC Symbol;Acc:29007]
rs3764803	X	FRMPD4	HGNC Symbol	FERM and PDZ domain containing 4 [Source:HGNC Symbol;Acc:29007]
rs739576	X	FRMPD4	HGNC Symbol	FERM and PDZ domain containing 4 [Source:HGNC Symbol;Acc:29007]
rs112046747	X	FRMPD4	HGNC Symbol	FERM and PDZ domain containing 4 [Source:HGNC Symbol;Acc:29007]
rs5933977	X	FRMPD4	HGNC Symbol	FERM and PDZ domain containing 4 [Source:HGNC Symbol;Acc:29007]
rs5979557	X	FRMPD4	HGNC Symbol	FERM and PDZ domain containing 4 [Source:HGNC Symbol;Acc:29007]
rs5935286	X	FRMPD4	HGNC Symbol	FERM and PDZ domain containing 4 [Source:HGNC Symbol;Acc:29007]
rs4073740	X	FRMPD4	HGNC Symbol	FERM and PDZ domain containing 4 [Source:HGNC Symbol;Acc:29007]
rs12846283	X	FRMPD4	HGNC Symbol	FERM and PDZ domain containing 4 [Source:HGNC Symbol;Acc:29007]
rs4411077	X	FRMPD4	HGNC Symbol	FERM and PDZ domain containing 4 [Source:HGNC Symbol;Acc:29007]
rs113989044	X	FRMPD4	HGNC Symbol	FERM and PDZ domain containing 4 [Source:HGNC Symbol;Acc:29007]
rs62588580	X	FRMPD4	HGNC Symbol	FERM and PDZ domain containing 4 [Source:HGNC Symbol;Acc:29007]
rs4387121	X	FRMPD4	HGNC Symbol	FERM and PDZ domain containing 4 [Source:HGNC Symbol;Acc:29007]
rs6640969	X	FRMPD4	HGNC Symbol	FERM and PDZ domain containing 4 [Source:HGNC Symbol;Acc:29007]
rs5933985	X	FRMPD4	HGNC Symbol	FERM and PDZ domain containing 4 [Source:HGNC Symbol;Acc:29007]
rs56011040	X	FRMPD4	HGNC Symbol	FERM and PDZ domain containing 4 [Source:HGNC Symbol;Acc:29007]
rs4300167	X	FRMPD4	HGNC Symbol	FERM and PDZ domain containing 4 [Source:HGNC Symbol;Acc:29007]
rs5979606	X	FRMPD4	HGNC Symbol	FERM and PDZ domain containing 4 [Source:HGNC Symbol;Acc:29007]
rs56919142	X	FRMPD4	HGNC Symbol	FERM and PDZ domain containing 4 [Source:HGNC Symbol;Acc:29007]
rs73192419	X	FRMPD4	HGNC Symbol	FERM and PDZ domain containing 4 [Source:HGNC Symbol;Acc:29007]
rs6639175	X	FRMPD4	HGNC Symbol	FERM and PDZ domain containing 4 [Source:HGNC Symbol;Acc:29007]
rs5933995	X	FRMPD4	HGNC Symbol	FERM and PDZ domain containing 4 [Source:HGNC Symbol;Acc:29007]
rs5933996	X	FRMPD4	HGNC Symbol	FERM and PDZ domain containing 4 [Source:HGNC Symbol;Acc:29007]
rs62590562	X	FRMPD4	HGNC Symbol	FERM and PDZ domain containing 4 [Source:HGNC Symbol;Acc:29007]
rs35657063	X	FRMPD4	HGNC Symbol	FERM and PDZ domain containing 4 [Source:HGNC Symbol;Acc:29007]
rs76486494	X	FRMPD4	HGNC Symbol	FERM and PDZ domain containing 4 [Source:HGNC Symbol;Acc:29007]
rs2407774	X	FRMPD4	HGNC Symbol	FERM and PDZ domain containing 4 [Source:HGNC Symbol;Acc:29007]
rs4830775	X	FRMPD4	HGNC Symbol	FERM and PDZ domain containing 4 [Source:HGNC Symbol;Acc:29007]
rs6640981	X	FRMPD4	HGNC Symbol	FERM and PDZ domain containing 4 [Source:HGNC Symbol;Acc:29007]
rs1874038	X	FRMPD4	HGNC Symbol	FERM and PDZ domain containing 4 [Source:HGNC Symbol;Acc:29007]
rs112784266	X	FRMPD4	HGNC Symbol	FERM and PDZ domain containing 4 [Source:HGNC Symbol;Acc:29007]
rs12010947	X	FRMPD4	HGNC Symbol	FERM and PDZ domain containing 4 [Source:HGNC Symbol;Acc:29007]
rs55761104	X	FRMPD4	HGNC Symbol	FERM and PDZ domain containing 4 [Source:HGNC Symbol;Acc:29007]

SNP	Chromosome	gene name	gene source	description
rs2407840	X	FRMPD4	HGNC Symbol	FERM and PDZ domain containing 4 [Source:HGNC Symbol;Acc:29007]
rs5935336	X	FRMPD4	HGNC Symbol	FERM and PDZ domain containing 4 [Source:HGNC Symbol;Acc:29007]
rs73192437	X	FRMPD4	HGNC Symbol	FERM and PDZ domain containing 4 [Source:HGNC Symbol;Acc:29007]
rs5978532	X	FRMPD4	HGNC Symbol	FERM and PDZ domain containing 4 [Source:HGNC Symbol;Acc:29007]
rs6419058	X	FRMPD4	HGNC Symbol	FERM and PDZ domain containing 4 [Source:HGNC Symbol;Acc:29007]
rs5935344	X	FRMPD4	HGNC Symbol	FERM and PDZ domain containing 4 [Source:HGNC Symbol;Acc:29007]
rs149324165	X	FRMPD4	HGNC Symbol	FERM and PDZ domain containing 4 [Source:HGNC Symbol;Acc:29007]
rs12838746	X	FRMPD4	HGNC Symbol	FERM and PDZ domain containing 4 [Source:HGNC Symbol;Acc:29007]
rs5935348	X	FRMPD4	HGNC Symbol	FERM and PDZ domain containing 4 [Source:HGNC Symbol;Acc:29007]
rs150344724	X	FRMPD4	HGNC Symbol	FERM and PDZ domain containing 4 [Source:HGNC Symbol;Acc:29007]
rs2407851	X	FRMPD4	HGNC Symbol	FERM and PDZ domain containing 4 [Source:HGNC Symbol;Acc:29007]
rs6639186	X	FRMPD4	HGNC Symbol	FERM and PDZ domain containing 4 [Source:HGNC Symbol;Acc:29007]
rs5935355	X	FRMPD4	HGNC Symbol	FERM and PDZ domain containing 4 [Source:HGNC Symbol;Acc:29007]
rs5978537	X	FRMPD4	HGNC Symbol	FERM and PDZ domain containing 4 [Source:HGNC Symbol;Acc:29007]
rs34474447	X	FRMPD4	HGNC Symbol	FERM and PDZ domain containing 4 [Source:HGNC Symbol;Acc:29007]
rs149008462	X	FRMPD4	HGNC Symbol	FERM and PDZ domain containing 4 [Source:HGNC Symbol;Acc:29007]
rs56031937	X	FRMPD4	HGNC Symbol	FERM and PDZ domain containing 4 [Source:HGNC Symbol;Acc:29007]
rs73192457	X	FRMPD4	HGNC Symbol	FERM and PDZ domain containing 4 [Source:HGNC Symbol;Acc:29007]
rs73192460	X	FRMPD4	HGNC Symbol	FERM and PDZ domain containing 4 [Source:HGNC Symbol;Acc:29007]
rs5935361	X	FRMPD4	HGNC Symbol	FERM and PDZ domain containing 4 [Source:HGNC Symbol;Acc:29007]
rs5979642	X	FRMPD4	HGNC Symbol	FERM and PDZ domain containing 4 [Source:HGNC Symbol;Acc:29007]
rs11095573	X	FRMPD4	HGNC Symbol	FERM and PDZ domain containing 4 [Source:HGNC Symbol;Acc:29007]
rs73440714	X	FRMPD4	HGNC Symbol	FERM and PDZ domain containing 4 [Source:HGNC Symbol;Acc:29007]
rs5935370	X	FRMPD4	HGNC Symbol	FERM and PDZ domain containing 4 [Source:HGNC Symbol;Acc:29007]
rs972241	X	FRMPD4	HGNC Symbol	FERM and PDZ domain containing 4 [Source:HGNC Symbol;Acc:29007]
rs4830783	X	FRMPD4	HGNC Symbol	FERM and PDZ domain containing 4 [Source:HGNC Symbol;Acc:29007]
rs5934022	X	FRMPD4	HGNC Symbol	FERM and PDZ domain containing 4 [Source:HGNC Symbol;Acc:29007]
rs4830785	X	FRMPD4	HGNC Symbol	FERM and PDZ domain containing 4 [Source:HGNC Symbol;Acc:29007]
rs6530538	X	FRMPD4	HGNC Symbol	FERM and PDZ domain containing 4 [Source:HGNC Symbol;Acc:29007]
rs2897787	X	FRMPD4	HGNC Symbol	FERM and PDZ domain containing 4 [Source:HGNC Symbol;Acc:29007]
rs1438483	X	FRMPD4	HGNC Symbol	FERM and PDZ domain containing 4 [Source:HGNC Symbol;Acc:29007]
rs5979669	X	FRMPD4	HGNC Symbol	FERM and PDZ domain containing 4 [Source:HGNC Symbol;Acc:29007]
rs5979671	X	FRMPD4	HGNC Symbol	FERM and PDZ domain containing 4 [Source:HGNC Symbol;Acc:29007]

SNP	Chromosome	gene name	gene source	description
rs6639207	X	FRMPD4	HGNC Symbol	FERM and PDZ domain containing 4 [Source:HGNC Symbol;Acc:29007]
rs147532402	X	FRMPD4	HGNC Symbol	FERM and PDZ domain containing 4 [Source:HGNC Symbol;Acc:29007]
rs5979681	X	FRMPD4	HGNC Symbol	FERM and PDZ domain containing 4 [Source:HGNC Symbol;Acc:29007]
rs142726552	X	FRMPD4	HGNC Symbol	FERM and PDZ domain containing 4 [Source:HGNC Symbol;Acc:29007]
rs12860185	X	FRMPD4	HGNC Symbol	FERM and PDZ domain containing 4 [Source:HGNC Symbol;Acc:29007]
rs5979701	X	FRMPD4	HGNC Symbol	FERM and PDZ domain containing 4 [Source:HGNC Symbol;Acc:29007]
rs10521627	X	FRMPD4	HGNC Symbol	FERM and PDZ domain containing 4 [Source:HGNC Symbol;Acc:29007]
rs73196401	X	FRMPD4	HGNC Symbol	FERM and PDZ domain containing 4 [Source:HGNC Symbol;Acc:29007]
rs6530551	X	FRMPD4	HGNC Symbol	FERM and PDZ domain containing 4 [Source:HGNC Symbol;Acc:29007]
rs5979703	X	FRMPD4	HGNC Symbol	FERM and PDZ domain containing 4 [Source:HGNC Symbol;Acc:29007]
rs190831088	X	FRMPD4	HGNC Symbol	FERM and PDZ domain containing 4 [Source:HGNC Symbol;Acc:29007]
rs62590595	X	FRMPD4	HGNC Symbol	FERM and PDZ domain containing 4 [Source:HGNC Symbol;Acc:29007]
rs5979709	X	FRMPD4	HGNC Symbol	FERM and PDZ domain containing 4 [Source:HGNC Symbol;Acc:29007]
rs112018100	X	FRMPD4	HGNC Symbol	FERM and PDZ domain containing 4 [Source:HGNC Symbol;Acc:29007]
rs11095580	X	FRMPD4	HGNC Symbol	FERM and PDZ domain containing 4 [Source:HGNC Symbol;Acc:29007]
rs73198276	X	FRMPD4	HGNC Symbol	FERM and PDZ domain containing 4 [Source:HGNC Symbol;Acc:29007]
rs6641073	X	FRMPD4	HGNC Symbol	FERM and PDZ domain containing 4 [Source:HGNC Symbol;Acc:29007]
rs141566161	X	FRMPD4	HGNC Symbol	FERM and PDZ domain containing 4 [Source:HGNC Symbol;Acc:29007]
rs4355984	X	FRMPD4	HGNC Symbol	FERM and PDZ domain containing 4 [Source:HGNC Symbol;Acc:29007]
rs56254922	X	FRMPD4	HGNC Symbol	FERM and PDZ domain containing 4 [Source:HGNC Symbol;Acc:29007]
rs4830792	X	FRMPD4	HGNC Symbol	FERM and PDZ domain containing 4 [Source:HGNC Symbol;Acc:29007]
rs73198283	X	FRMPD4	HGNC Symbol	FERM and PDZ domain containing 4 [Source:HGNC Symbol;Acc:29007]
rs5978569	X	FRMPD4	HGNC Symbol	FERM and PDZ domain containing 4 [Source:HGNC Symbol;Acc:29007]
rs4830797	X	FRMPD4	HGNC Symbol	FERM and PDZ domain containing 4 [Source:HGNC Symbol;Acc:29007]
rs4483343	X	FRMPD4	HGNC Symbol	FERM and PDZ domain containing 4 [Source:HGNC Symbol;Acc:29007]
rs4830475	X	FRMPD4	HGNC Symbol	FERM and PDZ domain containing 4 [Source:HGNC Symbol;Acc:29007]
rs5935388	X	FRMPD4	HGNC Symbol	FERM and PDZ domain containing 4 [Source:HGNC Symbol;Acc:29007]
rs5934031	X	FRMPD4	HGNC Symbol	FERM and PDZ domain containing 4 [Source:HGNC Symbol;Acc:29007]
rs12557959	X	FRMPD4	HGNC Symbol	FERM and PDZ domain containing 4 [Source:HGNC Symbol;Acc:29007]
rs17321823	X	FRMPD4	HGNC Symbol	FERM and PDZ domain containing 4 [Source:HGNC Symbol;Acc:29007]
rs73434772				
rs1266334				
rs1544540				

SNP	Chromosome	gene name	gene source	description
rs1266341				
rs17321858				
rs11095581				
rs60563087				
rs1731480				
rs6639217				
rs1266360				
rs59674337				
rs1731475				
rs1406788				
rs62589384				
rs1731471				
rs62589389	X	PRPS2	HGNC Symbol	phosphoribosyl pyrophosphate synthetase 2 [Source:HGNC Symbol;Acc:9465]
rs1632295	X	PRPS2	HGNC Symbol	phosphoribosyl pyrophosphate synthetase 2 [Source:HGNC Symbol;Acc:9465]
rs4639691	X	PRPS2	HGNC Symbol	phosphoribosyl pyrophosphate synthetase 2 [Source:HGNC Symbol;Acc:9465]
rs17281523	X	PRPS2	HGNC Symbol	phosphoribosyl pyrophosphate synthetase 2 [Source:HGNC Symbol;Acc:9465]
rs2239490	X	PRPS2	HGNC Symbol	phosphoribosyl pyrophosphate synthetase 2 [Source:HGNC Symbol;Acc:9465]
rs1269016	X	PRPS2	HGNC Symbol	phosphoribosyl pyrophosphate synthetase 2 [Source:HGNC Symbol;Acc:9465]
rs7892586	X	PRPS2	HGNC Symbol	phosphoribosyl pyrophosphate synthetase 2 [Source:HGNC Symbol;Acc:9465]
rs1266349	X	PRPS2	HGNC Symbol	phosphoribosyl pyrophosphate synthetase 2 [Source:HGNC Symbol;Acc:9465]
rs5935399	X	PRPS2	HGNC Symbol	phosphoribosyl pyrophosphate synthetase 2 [Source:HGNC Symbol;Acc:9465]
rs55687612				
rs5979746				
rs5935402				
rs5935414				
rs5935415				
rs5935419				
rs6530584				
rs2897826				
rs2699995				
rs12843803				
rs5743733	X	TLR7	HGNC Symbol	toll-like receptor 7 [Source:HGNC Symbol;Acc:15631]
rs5743740	X	TLR7	HGNC Symbol	toll-like receptor 7 [Source:HGNC Symbol;Acc:15631]

SNP	Chromosome	gene name	gene source	description
rs1638595	X	TLR7	HGNC Symbol	toll-like receptor 7 [Source:HGNC Symbol;Acc:15631]
rs1634319	X	TLR7	HGNC Symbol	toll-like receptor 7 [Source:HGNC Symbol;Acc:15631]
rs179009	X	TLR7	HGNC Symbol	toll-like receptor 7 [Source:HGNC Symbol;Acc:15631]
rs5743780	X	TLR7	HGNC Symbol	toll-like receptor 7 [Source:HGNC Symbol;Acc:15631]
rs864058	X	TLR7	HGNC Symbol	toll-like receptor 7 [Source:HGNC Symbol;Acc:15631]
rs1634318				
rs36043399				
rs179000				
rs178999				
rs178996				
rs3761622	X	TLR8-AS1	HGNC Symbol	TLR8 antisense RNA 1 [Source:HGNC Symbol;Acc:40720]
rs3761623	X	TLR8-AS1	HGNC Symbol	TLR8 antisense RNA 1 [Source:HGNC Symbol;Acc:40720]
rs5744043	X	TLR8;TLR8-AS1	HGNC Symbol	toll-like receptor 8 [Source:HGNC Symbol;Acc:15632];TLR8 antisense RNA 1 [Source:HGNC Symbol;Acc:40720]
rs3764880	X	TLR8;TLR8-AS1	HGNC Symbol	toll-like receptor 8 [Source:HGNC Symbol;Acc:15632];TLR8 antisense RNA 1 [Source:HGNC Symbol;Acc:40720]
rs4830805	X	TLR8	HGNC Symbol	toll-like receptor 8 [Source:HGNC Symbol;Acc:15632]
rs5744055	X	TLR8	HGNC Symbol	toll-like receptor 8 [Source:HGNC Symbol;Acc:15632]
rs2159377	X	TLR8	HGNC Symbol	toll-like receptor 8 [Source:HGNC Symbol;Acc:15632]
rs5744080	X	TLR8	HGNC Symbol	toll-like receptor 8 [Source:HGNC Symbol;Acc:15632]
rs2407992	X	TLR8	HGNC Symbol	toll-like receptor 8 [Source:HGNC Symbol;Acc:15632]
rs5744083	X	TLR8	HGNC Symbol	toll-like receptor 8 [Source:HGNC Symbol;Acc:15632]
rs3747414	X	TLR8	HGNC Symbol	toll-like receptor 8 [Source:HGNC Symbol;Acc:15632]
rs5744088	X	TLR8	HGNC Symbol	toll-like receptor 8 [Source:HGNC Symbol;Acc:15632]
rs5935465				
rs139649511				
rs62591209				
rs5979784				
rs113500987				
rs11798269				
rs5979785				
rs62591210				
rs9284570				
rs73184236				

SNP	Chromosome	gene name	gene source	description
rs5934048				
rs9778693				
rs9779183				
rs58654978				
rs850636				
rs850634				
rs5978598				
rs17322025				
rs141541315				
rs16978608				
rs2008165				
rs11095602	X	FAM9C	HGNC Symbol	family with sequence similarity 9, member C [Source:HGNC Symbol;Acc:18405]
rs12016287				
rs5935485				
rs140515167				
rs850631				
rs1230145				
rs141005395				
rs73192218				
rs3851826				
rs5934071				
rs66778832				
rs5978611				
rs73192231				
rs11797723				
rs5934077				
rs5935517				
rs6526375				
rs61384926				
rs11797724				
rs5935523				
rs73192240				
rs10856515				

SNP	Chromosome	gene name	gene source	description
rs1230224				
rs17281613				
rs5979827				
rs5979829				
rs12851640				
rs5979835				
rs6631139				
rs5979841				
rs6631468				
rs10465385				
rs12834438	X	GS1-600G8.5	Clone-based (Vega) gene	
rs17256262	X	GS1-600G8.5	Clone-based (Vega) gene	
rs4830840	X	GS1-600G8.5	Clone-based (Vega) gene	
rs4830494	X	GS1-600G8.5	Clone-based (Vega) gene	
rs113670844	X	GS1-600G8.3	Clone-based (Vega) gene	
rs4830842	X	ATXN3L;GS1-600G8.3	HGNC Symbol;Clone-based (Vega) gene	ataxin 3-like [Source:HGNC Symbol;Acc:24173];
rs12842741				
rs6632679				
rs17256276				
rs5934088				
rs150752203	X	RP11-142G7.2	Clone-based (Vega) gene	
rs73192267	X	RP11-142G7.2	Clone-based (Vega) gene	
rs17322178	X	RP11-142G7.2	Clone-based (Vega) gene	
rs1015560	X	RP11-142G7.2	Clone-based (Vega) gene	
rs66659952	X	RP11-1L9.1	Clone-based (Vega) gene	

SNP	Chromosome	gene name	gene source	description
rs5934095	X	RP11-1L9.1	Clone-based (Vega) gene	
rs4830853				
rs62587807				
rs73193908				
rs11302155				
rs5979867				
rs73193916				
rs12011237				
rs11798257				
rs4830503				
rs148991762				
rs6632904				
rs12689294				
rs5934103				
rs5979878				
rs5935579				
rs144340366				
rs5979882				
rs113081319				
rs4830505				
rs1431726				
rs1982045				
rs1982044				
rs73193934				
rs5978638				
rs60543643				
rs7066674				
rs17281676				
rs5978640				
rs113747363				
rs62586065				
rs12844988				

SNP	Chromosome	gene name	gene source	description
rs28727959				
rs5935589				
rs12015040				
rs12863606				
rs4830867				
rs6527809				
rs5935592				
rs73195762				
rs6629237				
rs6633004				
rs5935594				
rs10521634				
rs6633022				
rs5935597				
rs1010019				
rs73195770				
rs12559478				
rs12844566				
rs1431723				
rs58835238				
rs111796707				
rs139126101				
rs12860292				
rs5935604				
rs5935605				
rs113662208				
rs891703				
rs141517989	X	EGFL6	HGNC Symbol	EGF-like-domain, multiple 6 [Source:HGNC Symbol;Acc:3235]
rs36008953	X	EGFL6	HGNC Symbol	EGF-like-domain, multiple 6 [Source:HGNC Symbol;Acc:3235]
rs5978648	X	EGFL6	HGNC Symbol	EGF-like-domain, multiple 6 [Source:HGNC Symbol;Acc:3235]
rs5935627	X	EGFL6	HGNC Symbol	EGF-like-domain, multiple 6 [Source:HGNC Symbol;Acc:3235]
rs5935628	X	EGFL6	HGNC Symbol	EGF-like-domain, multiple 6 [Source:HGNC Symbol;Acc:3235]
rs34186328	X	EGFL6	HGNC Symbol	EGF-like-domain, multiple 6 [Source:HGNC Symbol;Acc:3235]

SNP	Chromosome	gene name	gene source	description
rs5978653	X	EGFL6	HGNC Symbol	EGF-like-domain, multiple 6 [Source:HGNC Symbol;Acc:3235]
rs5935635	X	EGFL6	HGNC Symbol	EGF-like-domain, multiple 6 [Source:HGNC Symbol;Acc:3235]
rs16979033	X	EGFL6	HGNC Symbol	EGF-like-domain, multiple 6 [Source:HGNC Symbol;Acc:3235]
rs73195794				
rs55773149				
rs144911355				
rs4830882	X	TCEANC	HGNC Symbol	transcription elongation factor A (SII) N-terminal and central domain containing [Source:HGNC Symbol;Acc:28277]
rs2361159	X	TCEANC	HGNC Symbol	transcription elongation factor A (SII) N-terminal and central domain containing [Source:HGNC Symbol;Acc:28277]
rs5935649	X	TCEANC	HGNC Symbol	transcription elongation factor A (SII) N-terminal and central domain containing [Source:HGNC Symbol;Acc:28277]
rs5935650	X	TCEANC	HGNC Symbol	transcription elongation factor A (SII) N-terminal and central domain containing [Source:HGNC Symbol;Acc:28277]
rs5979938	X	TCEANC	HGNC Symbol	transcription elongation factor A (SII) N-terminal and central domain containing [Source:HGNC Symbol;Acc:28277]
rs2370240				
rs1579179				
rs60543618	X	RAB9A	HGNC Symbol	RAB9A, member RAS oncogene family [Source:HGNC Symbol;Acc:9792]
rs12558341	X	RAB9A	HGNC Symbol	RAB9A, member RAS oncogene family [Source:HGNC Symbol;Acc:9792]
rs2028066	X	RAB9A	HGNC Symbol	RAB9A, member RAS oncogene family [Source:HGNC Symbol;Acc:9792]
rs2285635	X	OFD1	HGNC Symbol	oral-facial-digital syndrome 1 [Source:HGNC Symbol;Acc:2567]
rs2238908	X	OFD1	HGNC Symbol	oral-facial-digital syndrome 1 [Source:HGNC Symbol;Acc:2567]
rs5979958	X	OFD1	HGNC Symbol	oral-facial-digital syndrome 1 [Source:HGNC Symbol;Acc:2567]
rs11095627	X	GPM6B	HGNC Symbol	glycoprotein M6B [Source:HGNC Symbol;Acc:4461]
rs147232494	X	GPM6B	HGNC Symbol	glycoprotein M6B [Source:HGNC Symbol;Acc:4461]
rs36050008	X	GPM6B	HGNC Symbol	glycoprotein M6B [Source:HGNC Symbol;Acc:4461]
rs5979973	X	GPM6B	HGNC Symbol	glycoprotein M6B [Source:HGNC Symbol;Acc:4461]
rs7055085	X	GPM6B	HGNC Symbol	glycoprotein M6B [Source:HGNC Symbol;Acc:4461]
rs62587474	X	GPM6B	HGNC Symbol	glycoprotein M6B [Source:HGNC Symbol;Acc:4461]
rs55646372	X	GPM6B	HGNC Symbol	glycoprotein M6B [Source:HGNC Symbol;Acc:4461]
rs5935663	X	GPM6B	HGNC Symbol	glycoprotein M6B [Source:HGNC Symbol;Acc:4461]
rs11095629	X	GPM6B	HGNC Symbol	glycoprotein M6B [Source:HGNC Symbol;Acc:4461]
rs5979977	X	GPM6B	HGNC Symbol	glycoprotein M6B [Source:HGNC Symbol;Acc:4461]
rs72614523	X	GPM6B	HGNC Symbol	glycoprotein M6B [Source:HGNC Symbol;Acc:4461]

SNP	Chromosome	gene name	gene source	description
rs7052395	X	GPM6B	HGNC Symbol	glycoprotein M6B [Source:HGNC Symbol;Acc:4461]
rs6633386	X	GPM6B	HGNC Symbol	glycoprotein M6B [Source:HGNC Symbol;Acc:4461]
rs5979987	X	GPM6B	HGNC Symbol	glycoprotein M6B [Source:HGNC Symbol;Acc:4461]
rs5935669	X	GPM6B	HGNC Symbol	glycoprotein M6B [Source:HGNC Symbol;Acc:4461]
rs5978666	X	GPM6B	HGNC Symbol	glycoprotein M6B [Source:HGNC Symbol;Acc:4461]
rs12849213	X	GPM6B	HGNC Symbol	glycoprotein M6B [Source:HGNC Symbol;Acc:4461]
rs73197733	X	GPM6B	HGNC Symbol	glycoprotein M6B [Source:HGNC Symbol;Acc:4461]
rs1005589	X	GPM6B	HGNC Symbol	glycoprotein M6B [Source:HGNC Symbol;Acc:4461]
rs4830900	X	GPM6B	HGNC Symbol	glycoprotein M6B [Source:HGNC Symbol;Acc:4461]
rs12558882	X	GPM6B	HGNC Symbol	glycoprotein M6B [Source:HGNC Symbol;Acc:4461]
rs4830512	X	GPM6B	HGNC Symbol	glycoprotein M6B [Source:HGNC Symbol;Acc:4461]
rs66634009	X	GPM6B	HGNC Symbol	glycoprotein M6B [Source:HGNC Symbol;Acc:4461]
rs16979342	X	GPM6B	HGNC Symbol	glycoprotein M6B [Source:HGNC Symbol;Acc:4461]
rs12855293	X	GPM6B	HGNC Symbol	glycoprotein M6B [Source:HGNC Symbol;Acc:4461]
rs17300814	X	GPM6B	HGNC Symbol	glycoprotein M6B [Source:HGNC Symbol;Acc:4461]
rs11796423	X	GPM6B	HGNC Symbol	glycoprotein M6B [Source:HGNC Symbol;Acc:4461]
rs7056680	X	GPM6B	HGNC Symbol	glycoprotein M6B [Source:HGNC Symbol;Acc:4461]
rs6654096	X	GPM6B	HGNC Symbol	glycoprotein M6B [Source:HGNC Symbol;Acc:4461]
rs35804867	X	GPM6B	HGNC Symbol	glycoprotein M6B [Source:HGNC Symbol;Acc:4461]
rs66560303	X	GPM6B	HGNC Symbol	glycoprotein M6B [Source:HGNC Symbol;Acc:4461]
rs11798993				
rs16979374	X	RP1-122K4.2	Clone-based (Vega) gene	
rs113031247	X	RP1-122K4.2	Clone-based (Vega) gene	
rs138699197				
rs7060169				
rs17215777	X	GEMIN8	HGNC Symbol	gem (nuclear organelle) associated protein 8 [Source:HGNC Symbol;Acc:26044]
rs112961490				
rs6633729				
rs73197755				
rs6526226				
rs7050970				
rs55959731				

SNP	Chromosome	gene name	gene source	description
rs5935687				
rs57948619				
rs112304727				
rs7879032				
rs5934153				
rs7053403				
rs56827736				
rs112694821				
rs1989844				
rs5978676				
rs61490089				
rs987720				
rs2074006				
rs41523250				
rs73197799				
rs148720499				
rs10521640				
rs5934165				
rs764232				
rs35599364				
rs16979560				
rs5978683				
rs183347664				
rs5935758				
rs112869086				
rs147371792	X	GLRA2	HGNC Symbol	glycine receptor, alpha 2 [Source:HGNC Symbol;Acc:4327]
rs5935767	X	GLRA2	HGNC Symbol	glycine receptor, alpha 2 [Source:HGNC Symbol;Acc:4327]
rs3027332	X	GLRA2	HGNC Symbol	glycine receptor, alpha 2 [Source:HGNC Symbol;Acc:4327]
rs7889706	X	GLRA2	HGNC Symbol	glycine receptor, alpha 2 [Source:HGNC Symbol;Acc:4327]
rs5935775	X	GLRA2	HGNC Symbol	glycine receptor, alpha 2 [Source:HGNC Symbol;Acc:4327]
rs151306333	X	GLRA2	HGNC Symbol	glycine receptor, alpha 2 [Source:HGNC Symbol;Acc:4327]
rs3027354	X	GLRA2	HGNC Symbol	glycine receptor, alpha 2 [Source:HGNC Symbol;Acc:4327]
rs3027361	X	GLRA2	HGNC Symbol	glycine receptor, alpha 2 [Source:HGNC Symbol;Acc:4327]

SNP	Chromosome	gene name	gene source	description
rs73199669	X	GLRA2	HGNC Symbol	glycine receptor, alpha 2 [Source:HGNC Symbol;Acc:4327]
rs112388620	X	GLRA2	HGNC Symbol	glycine receptor, alpha 2 [Source:HGNC Symbol;Acc:4327]
rs151150481	X	GLRA2	HGNC Symbol	glycine receptor, alpha 2 [Source:HGNC Symbol;Acc:4327]
rs67580665	X	GLRA2	HGNC Symbol	glycine receptor, alpha 2 [Source:HGNC Symbol;Acc:4327]
rs5980062	X	GLRA2	HGNC Symbol	glycine receptor, alpha 2 [Source:HGNC Symbol;Acc:4327]
rs34324341	X	GLRA2	HGNC Symbol	glycine receptor, alpha 2 [Source:HGNC Symbol;Acc:4327]
rs11796093	X	GLRA2	HGNC Symbol	glycine receptor, alpha 2 [Source:HGNC Symbol;Acc:4327]
rs5935805				
rs7058918				
rs73199698				
rs3020859				
rs185712238				
rs727976				
rs6527027	X	FANCB	HGNC Symbol	Fanconi anemia, complementation group B [Source:HGNC Symbol;Acc:3583]
rs41309679	X	FANCB	HGNC Symbol	Fanconi anemia, complementation group B [Source:HGNC Symbol;Acc:3583]
rs11795930	X	FANCB	HGNC Symbol	Fanconi anemia, complementation group B [Source:HGNC Symbol;Acc:3583]
rs139120857	X	MOSPD2	HGNC Symbol	motile sperm domain containing 2 [Source:HGNC Symbol;Acc:28381]
rs2106413	X	MOSPD2	HGNC Symbol	motile sperm domain containing 2 [Source:HGNC Symbol;Acc:28381]
rs35164803	X	MOSPD2	HGNC Symbol	motile sperm domain containing 2 [Source:HGNC Symbol;Acc:28381]
rs41305211	X	MOSPD2	HGNC Symbol	motile sperm domain containing 2 [Source:HGNC Symbol;Acc:28381]
rs73441346				
rs5935828				
rs35080706				
rs73441359				
rs148967689				
rs16979717				
rs6527165	X	TPT1P14	HGNC Symbol	tumor protein, translationally-controlled 1 pseudogene 14 [Source:HGNC Symbol;Acc:49305]
rs140411897				
rs16997003				
rs5935874				
rs142345312				
rs5935878				
rs35748104				

SNP	Chromosome	gene name	gene source	description
rs12391678				
rs5980114				
rs148980321				
rs17216162				
rs6631855				
rs6631870				
rs1564403				
rs2873427				
rs5935907				
rs12862900				
rs11797338				
rs5935911				
rs112827279				
rs14252	X	ASB9	HGNC Symbol	ankyrin repeat and SOCS box containing 9 [Source:HGNC Symbol;Acc:17184]
rs12556121	X	ASB9	HGNC Symbol	ankyrin repeat and SOCS box containing 9 [Source:HGNC Symbol;Acc:17184]
rs10856268	X	ASB9	HGNC Symbol	ankyrin repeat and SOCS box containing 9 [Source:HGNC Symbol;Acc:17184]
rs73189523	X	ASB9	HGNC Symbol	ankyrin repeat and SOCS box containing 9 [Source:HGNC Symbol;Acc:17184]
rs1139868	X	ASB9	HGNC Symbol	ankyrin repeat and SOCS box containing 9 [Source:HGNC Symbol;Acc:17184]
rs731197	X	ASB9	HGNC Symbol	ankyrin repeat and SOCS box containing 9 [Source:HGNC Symbol;Acc:17184]
rs6527396	X	ASB9	HGNC Symbol	ankyrin repeat and SOCS box containing 9 [Source:HGNC Symbol;Acc:17184]
rs6418672	X	ASB9	HGNC Symbol	ankyrin repeat and SOCS box containing 9 [Source:HGNC Symbol;Acc:17184]
rs899646	X	ASB9	HGNC Symbol	ankyrin repeat and SOCS box containing 9 [Source:HGNC Symbol;Acc:17184]
rs4830935				
rs5935925	X	ASB11	HGNC Symbol	ankyrin repeat and SOCS box containing 11 [Source:HGNC Symbol;Acc:17186]
rs41309559	X	ASB11	HGNC Symbol	ankyrin repeat and SOCS box containing 11 [Source:HGNC Symbol;Acc:17186]
rs35859007	X	ASB11	HGNC Symbol	ankyrin repeat and SOCS box containing 11 [Source:HGNC Symbol;Acc:17186]
rs6628943	X	ASB11	HGNC Symbol	ankyrin repeat and SOCS box containing 11 [Source:HGNC Symbol;Acc:17186]
rs5935944	X	ASB11	HGNC Symbol	ankyrin repeat and SOCS box containing 11 [Source:HGNC Symbol;Acc:17186]
rs7878777				
rs34422225	X	PIGA	HGNC Symbol	phosphatidylinositol glycan anchor biosynthesis, class A [Source:HGNC Symbol;Acc:8957]
rs138749277	X	FIGF	HGNC Symbol	c-fos induced growth factor (vascular endothelial growth factor D) [Source:HGNC Symbol;Acc:3708]
rs12857904	X	FIGF	HGNC Symbol	c-fos induced growth factor (vascular endothelial growth factor D) [Source:HGNC Symbol;Acc:3708]
rs12011065	X	FIGF	HGNC Symbol	c-fos induced growth factor (vascular endothelial growth factor D) [Source:HGNC Symbol;Acc:3708]

SNP	Chromosome	gene name	gene source	description
rs4830939	X	FIGF	HGNC Symbol	c-fos induced growth factor (vascular endothelial growth factor D) [Source:HGNC Symbol;Acc:3708]
rs6632519	X	FIGF	HGNC Symbol	c-fos induced growth factor (vascular endothelial growth factor D) [Source:HGNC Symbol;Acc:3708]
rs6632528	X	FIGF	HGNC Symbol	c-fos induced growth factor (vascular endothelial growth factor D) [Source:HGNC Symbol;Acc:3708]
rs2071178	X	PIR	HGNC Symbol	pirin (iron-binding nuclear protein) [Source:HGNC Symbol;Acc:30048]
rs8094	X	PIR	HGNC Symbol	pirin (iron-binding nuclear protein) [Source:HGNC Symbol;Acc:30048]
rs234491	X	PIR	HGNC Symbol	pirin (iron-binding nuclear protein) [Source:HGNC Symbol;Acc:30048]
rs5935982	X	PIR	HGNC Symbol	pirin (iron-binding nuclear protein) [Source:HGNC Symbol;Acc:30048]
rs5935984	X	PIR;BMX	HGNC Symbol	pirin (iron-binding nuclear protein) [Source:HGNC Symbol;Acc:30048];BMX non-receptor tyrosine kinase [Source:HGNC Symbol;Acc:1079]
rs1983287	X	PIR;BMX	HGNC Symbol	pirin (iron-binding nuclear protein) [Source:HGNC Symbol;Acc:30048];BMX non-receptor tyrosine kinase [Source:HGNC Symbol;Acc:1079]
rs5935986	X	PIR;BMX	HGNC Symbol	pirin (iron-binding nuclear protein) [Source:HGNC Symbol;Acc:30048];BMX non-receptor tyrosine kinase [Source:HGNC Symbol;Acc:1079]
rs62578899	X	BMX	HGNC Symbol	BMX non-receptor tyrosine kinase [Source:HGNC Symbol;Acc:1079]
rs233567	X	BMX	HGNC Symbol	BMX non-receptor tyrosine kinase [Source:HGNC Symbol;Acc:1079]
rs62578901	X	BMX	HGNC Symbol	BMX non-receptor tyrosine kinase [Source:HGNC Symbol;Acc:1079]
rs35803318	X	ACE2	HGNC Symbol	angiotensin I converting enzyme 2 [Source:HGNC Symbol;Acc:13557]
rs41303171	X	ACE2	HGNC Symbol	angiotensin I converting enzyme 2 [Source:HGNC Symbol;Acc:13557]
rs2023802	X	ACE2	HGNC Symbol	angiotensin I converting enzyme 2 [Source:HGNC Symbol;Acc:13557]
rs2106809	X	ACE2	HGNC Symbol	angiotensin I converting enzyme 2 [Source:HGNC Symbol;Acc:13557]
rs141068813	X	GS1-594A7.3	Clone-based (Vega) gene	
rs1548474	X	GS1-594A7.3	Clone-based (Vega) gene	
rs1356037				
rs5936004	X	TMEM27	HGNC Symbol	transmembrane protein 27 [Source:HGNC Symbol;Acc:29437]
rs4830979	X	CA5BP1	HGNC Symbol	carbonic anhydrase VB pseudogene 1 [Source:HGNC Symbol;Acc:29544]
rs62578920	X	CA5BP1	HGNC Symbol	carbonic anhydrase VB pseudogene 1 [Source:HGNC Symbol;Acc:29544]
rs140607783	X	CA5B	HGNC Symbol	carbonic anhydrase VB, mitochondrial [Source:HGNC Symbol;Acc:1378]
rs6632722	X	CA5B	HGNC Symbol	carbonic anhydrase VB, mitochondrial [Source:HGNC Symbol;Acc:1378]
rs5936033	X	CA5B	HGNC Symbol	carbonic anhydrase VB, mitochondrial [Source:HGNC Symbol;Acc:1378]
rs5934272	X	CA5B	HGNC Symbol	carbonic anhydrase VB, mitochondrial [Source:HGNC Symbol;Acc:1378]
rs2316880	X	CA5B	HGNC Symbol	carbonic anhydrase VB, mitochondrial [Source:HGNC Symbol;Acc:1378]
rs11797879	X	CA5B	HGNC Symbol	carbonic anhydrase VB, mitochondrial [Source:HGNC Symbol;Acc:1378]
rs5980180	X	CA5B	HGNC Symbol	carbonic anhydrase VB, mitochondrial [Source:HGNC Symbol;Acc:1378]

SNP	Chromosome	gene name	gene source	description
rs1808	X	CA5B	HGNC Symbol	carbonic anhydrase VB, mitochondrial [Source:HGNC Symbol;Acc:1378]
rs140663644	X	ZRSR2	HGNC Symbol	zinc finger (CCCH type), RNA-binding motif and serine/arginine rich 2 [Source:HGNC Symbol;Acc:23019]
rs1557807	X	ZRSR2	HGNC Symbol	zinc finger (CCCH type), RNA-binding motif and serine/arginine rich 2 [Source:HGNC Symbol;Acc:23019]
rs2214428	X	AP1S2	HGNC Symbol	adaptor-related protein complex 1, sigma 2 subunit [Source:HGNC Symbol;Acc:560]
rs798166	X	AP1S2	HGNC Symbol	adaptor-related protein complex 1, sigma 2 subunit [Source:HGNC Symbol;Acc:560]
rs798151				
rs73444020				
rs60749614				
rs5980202				
rs5936081				
rs73202884				
rs705860				
rs1024459				
rs12006589				
rs11798628				
rs5934299				
rs12846884				
rs5936099				
rs150314154				
rs12859174				
rs34322323				
rs73202901				
rs4986945	X	GRPR	HGNC Symbol	gastrin-releasing peptide receptor [Source:HGNC Symbol;Acc:4609]
rs4986946	X	GRPR	HGNC Symbol	gastrin-releasing peptide receptor [Source:HGNC Symbol;Acc:4609]
rs5980234	X	RP11-431J24.2	Clone-based (Vega) gene	
rs73204604	X	RP11-431J24.2	Clone-based (Vega) gene	
rs3903014	X	MAGEB17;RP11-431J24.2	HGNC Symbol;Clone-based (Vega) gene	melanoma antigen family B, 17 [Source:HGNC Symbol;Acc:17418];
rs2107107				
rs5980241				

SNP	Chromosome	gene name	gene source	description
rs2522982				
rs5901583				
rs2702234				
rs6632790				
rs35959352				
rs6527675				
rs73204616				
rs10521659				
rs62585413				
rs6632802				
rs17311906				
rs5936129				
rs62585415				
rs73454287				
rs4830554				
rs72616010				
rs1527071				
rs73204659				
rs5936132				
rs17311982				
rs55833891				
rs7059394				
rs59598325				
rs4355975				
rs4831035				
rs7058049				
rs7061971				
rs73204691				
rs59713159				
rs7063937				
rs745913				
rs2428447				
rs5936144				

SNP	Chromosome	gene name	gene source	description
rs141894210				
rs1527805	X	CTPS2	HGNC Symbol	CTP synthase 2 [Source:HGNC Symbol;Acc:2520]
rs6527691	X	CTPS2	HGNC Symbol	CTP synthase 2 [Source:HGNC Symbol;Acc:2520]
rs17217318	X	CTPS2	HGNC Symbol	CTP synthase 2 [Source:HGNC Symbol;Acc:2520]
rs139959576	X	CTPS2	HGNC Symbol	CTP synthase 2 [Source:HGNC Symbol;Acc:2520]
rs5980310	X	CTPS2	HGNC Symbol	CTP synthase 2 [Source:HGNC Symbol;Acc:2520]
rs41311513	X	CTPS2;S100G	HGNC Symbol	CTP synthase 2 [Source:HGNC Symbol;Acc:2520];S100 calcium binding protein G [Source:HGNC Symbol;Acc:1436]
rs62589078	X	CTPS2;S100G	HGNC Symbol	CTP synthase 2 [Source:HGNC Symbol;Acc:2520];S100 calcium binding protein G [Source:HGNC Symbol;Acc:1436]
rs66841637	X	CTPS2	HGNC Symbol	CTP synthase 2 [Source:HGNC Symbol;Acc:2520]
rs6632862	X	CTPS2	HGNC Symbol	CTP synthase 2 [Source:HGNC Symbol;Acc:2520]
rs7061921	X	CTPS2	HGNC Symbol	CTP synthase 2 [Source:HGNC Symbol;Acc:2520]
rs140173662	X	CTPS2	HGNC Symbol	CTP synthase 2 [Source:HGNC Symbol;Acc:2520]
rs5980317	X	CTPS2	HGNC Symbol	CTP synthase 2 [Source:HGNC Symbol;Acc:2520]
rs5978787	X	CTPS2	HGNC Symbol	CTP synthase 2 [Source:HGNC Symbol;Acc:2520]
rs11379303	X	CTPS2	HGNC Symbol	CTP synthase 2 [Source:HGNC Symbol;Acc:2520]
rs11798449	X	CTPS2	HGNC Symbol	CTP synthase 2 [Source:HGNC Symbol;Acc:2520]
rs5936161				
rs139960326	X	SYAP1	HGNC Symbol	synapse associated protein 1 [Source:HGNC Symbol;Acc:16273]
rs113141175	X	SYAP1	HGNC Symbol	synapse associated protein 1 [Source:HGNC Symbol;Acc:16273]
rs5936169	X	SYAP1	HGNC Symbol	synapse associated protein 1 [Source:HGNC Symbol;Acc:16273]
rs137867939	X	TXLNG	HGNC Symbol	taxilin gamma [Source:HGNC Symbol;Acc:18578]
rs5924530	X	TXLNG;RBBP7	HGNC Symbol	taxilin gamma [Source:HGNC Symbol;Acc:18578];retinoblastoma binding protein 7 [Source:HGNC Symbol;Acc:9890]
rs3747366	X	TXLNG;RBBP7	HGNC Symbol	taxilin gamma [Source:HGNC Symbol;Acc:18578];retinoblastoma binding protein 7 [Source:HGNC Symbol;Acc:9890]
rs4828534	X	TXLNG;RBBP7	HGNC Symbol	taxilin gamma [Source:HGNC Symbol;Acc:18578];retinoblastoma binding protein 7 [Source:HGNC Symbol;Acc:9890]
rs5924560	X	RBBP7	HGNC Symbol	retinoblastoma binding protein 7 [Source:HGNC Symbol;Acc:9890]
rs67984110	X	RBBP7	HGNC Symbol	retinoblastoma binding protein 7 [Source:HGNC Symbol;Acc:9890]
rs5924611				
rs12560197				
rs5924607				
rs5969772				

SNP	Chromosome	gene name	gene source	description
rs1871514				
rs4528028				
rs7060904	X	REPS2	HGNC Symbol	RALBP1 associated Eps domain containing 2 [Source:HGNC Symbol;Acc:9963]
rs4828523	X	REPS2	HGNC Symbol	RALBP1 associated Eps domain containing 2 [Source:HGNC Symbol;Acc:9963]
rs17273615	X	REPS2	HGNC Symbol	RALBP1 associated Eps domain containing 2 [Source:HGNC Symbol;Acc:9963]
rs112444219	X	REPS2	HGNC Symbol	RALBP1 associated Eps domain containing 2 [Source:HGNC Symbol;Acc:9963]
rs9887218	X	REPS2	HGNC Symbol	RALBP1 associated Eps domain containing 2 [Source:HGNC Symbol;Acc:9963]
rs73189114	X	REPS2	HGNC Symbol	RALBP1 associated Eps domain containing 2 [Source:HGNC Symbol;Acc:9963]
rs1365528	X	REPS2	HGNC Symbol	RALBP1 associated Eps domain containing 2 [Source:HGNC Symbol;Acc:9963]
rs12396700	X	REPS2	HGNC Symbol	RALBP1 associated Eps domain containing 2 [Source:HGNC Symbol;Acc:9963]
rs5924601	X	REPS2	HGNC Symbol	RALBP1 associated Eps domain containing 2 [Source:HGNC Symbol;Acc:9963]
rs76739801				
rs2382815				
rs7878986				
rs142144853				
rs5950869				
rs5950862				
rs73630588				
rs73636638				
rs12689980				
rs5950859				
rs79955925				
rs11798344	X	NHS	HGNC Symbol	Nance-Horan syndrome (congenital cataracts and dental anomalies) [Source:HGNC Symbol;Acc:7820]
rs6632983	X	NHS	HGNC Symbol	Nance-Horan syndrome (congenital cataracts and dental anomalies) [Source:HGNC Symbol;Acc:7820]
rs66479566	X	NHS	HGNC Symbol	Nance-Horan syndrome (congenital cataracts and dental anomalies) [Source:HGNC Symbol;Acc:7820]
rs73189137	X	NHS	HGNC Symbol	Nance-Horan syndrome (congenital cataracts and dental anomalies) [Source:HGNC Symbol;Acc:7820]
rs10856284	X	NHS	HGNC Symbol	Nance-Horan syndrome (congenital cataracts and dental anomalies) [Source:HGNC Symbol;Acc:7820]
rs114290305	X	NHS	HGNC Symbol	Nance-Horan syndrome (congenital cataracts and dental anomalies) [Source:HGNC Symbol;Acc:7820]

SNP	Chromosome	gene name	gene source	description
rs6632987	X	NHS	HGNC Symbol	Nance-Horan syndrome (congenital cataracts and dental anomalies) [Source:HGNC Symbol;Acc:7820]
rs916313	X	NHS	HGNC Symbol	Nance-Horan syndrome (congenital cataracts and dental anomalies) [Source:HGNC Symbol;Acc:7820]
rs35108053	X	NHS;RP1-60N8.1	HGNC Symbol;Clone-based (Vega) gene	Nance-Horan syndrome (congenital cataracts and dental anomalies) [Source:HGNC Symbol;Acc:7820];
rs1894579	X	NHS;RP1-60N8.1	HGNC Symbol;Clone-based (Vega) gene	Nance-Horan syndrome (congenital cataracts and dental anomalies) [Source:HGNC Symbol;Acc:7820];
rs146561105	X	NHS;RP1-60N8.1	HGNC Symbol;Clone-based (Vega) gene	Nance-Horan syndrome (congenital cataracts and dental anomalies) [Source:HGNC Symbol;Acc:7820];
rs4825254	X	NHS;NHS-AS1;RP1-60N8.1	HGNC Symbol;Clone-based (Vega) gene	Nance-Horan syndrome (congenital cataracts and dental anomalies) [Source:HGNC Symbol;Acc:7820];NHS antisense RNA 1 [Source:HGNC Symbol;Acc:40403];
rs12836443	X	NHS;NHS-AS1;RP1-60N8.1	HGNC Symbol;Clone-based (Vega) gene	Nance-Horan syndrome (congenital cataracts and dental anomalies) [Source:HGNC Symbol;Acc:7820];NHS antisense RNA 1 [Source:HGNC Symbol;Acc:40403];
rs4825256	X	NHS;NHS-AS1;RP1-60N8.1	HGNC Symbol;Clone-based (Vega) gene	Nance-Horan syndrome (congenital cataracts and dental anomalies) [Source:HGNC Symbol;Acc:7820];NHS antisense RNA 1 [Source:HGNC Symbol;Acc:40403];
rs73189153	X	NHS;RP1-60N8.1	HGNC Symbol;Clone-based (Vega) gene	Nance-Horan syndrome (congenital cataracts and dental anomalies) [Source:HGNC Symbol;Acc:7820];
rs743880	X	NHS;RP1-60N8.1	HGNC Symbol;Clone-based (Vega) gene	Nance-Horan syndrome (congenital cataracts and dental anomalies) [Source:HGNC Symbol;Acc:7820];
rs5909437	X	NHS;RP1-60N8.1	HGNC Symbol;Clone-based (Vega) gene	Nance-Horan syndrome (congenital cataracts and dental anomalies) [Source:HGNC Symbol;Acc:7820];
rs5909459	X	NHS	HGNC Symbol	Nance-Horan syndrome (congenital cataracts and dental anomalies) [Source:HGNC Symbol;Acc:7820]
rs139099150	X	NHS	HGNC Symbol	Nance-Horan syndrome (congenital cataracts and dental anomalies) [Source:HGNC Symbol;Acc:7820]
rs150802792	X	NHS	HGNC Symbol	Nance-Horan syndrome (congenital cataracts and dental anomalies) [Source:HGNC Symbol;Acc:7820]
rs5955543	X	NHS	HGNC Symbol	Nance-Horan syndrome (congenital cataracts and dental anomalies) [Source:HGNC Symbol;Acc:7820]

SNP	Chromosome	gene name	gene source	description
rs1894583	X	NHS	HGNC Symbol	Nance-Horan syndrome (congenital cataracts and dental anomalies) [Source:HGNC Symbol;Acc:7820]
rs3747295	X	NHS	HGNC Symbol	Nance-Horan syndrome (congenital cataracts and dental anomalies) [Source:HGNC Symbol;Acc:7820]
rs17246582	X	NHS	HGNC Symbol	Nance-Horan syndrome (congenital cataracts and dental anomalies) [Source:HGNC Symbol;Acc:7820]
rs73189163	X	SCML1	HGNC Symbol	sex comb on midleg-like 1 (Drosophila) [Source:HGNC Symbol;Acc:10580]
rs17246603	X	SCML1	HGNC Symbol	sex comb on midleg-like 1 (Drosophila) [Source:HGNC Symbol;Acc:10580]
rs12387249				
rs16980679	X	RAI2	HGNC Symbol	retinoic acid induced 2 [Source:HGNC Symbol;Acc:9835]
rs10521677	X	RAI2	HGNC Symbol	retinoic acid induced 2 [Source:HGNC Symbol;Acc:9835]
rs111253580	X	RAI2	HGNC Symbol	retinoic acid induced 2 [Source:HGNC Symbol;Acc:9835]
rs150066059	X	RAI2	HGNC Symbol	retinoic acid induced 2 [Source:HGNC Symbol;Acc:9835]
rs5909370				
rs2051606				
rs142124900				
rs139411880				
rs17246666	X	RP3-410B11.1	Clone-based (Vega) gene	
rs73189178	X	RP3-410B11.1	Clone-based (Vega) gene	
rs4825255	X	RP3-410B11.1	Clone-based (Vega) gene	
rs5909140	X	RP3-410B11.1	Clone-based (Vega) gene	
rs6527822	X	RP3-410B11.1	Clone-based (Vega) gene	
rs73189179	X	RP3-410B11.1	Clone-based (Vega) gene	
rs5955598	X	RP3-410B11.1	Clone-based (Vega) gene	
rs17246701	X	RP3-410B11.1	Clone-based (Vega) gene	
rs5909379	X	RP3-410B11.1	Clone-based (Vega) gene	
rs12689258	X	RP3-410B11.1	Clone-based (Vega) gene	

SNP	Chromosome	gene name	gene source	description
rs111664040	X	RP3-410B11.1	Clone-based (Vega) gene	
rs55845833	X	RP3-410B11.1	Clone-based (Vega) gene	
rs5955927	X	RP3-410B11.1	Clone-based (Vega) gene	
rs5909143	X	RP3-410B11.1	Clone-based (Vega) gene	
rs2227145				
rs9792797				
rs5909390				
rs5909150				
rs11796956				
rs112197439				
rs73189195				
rs34413440	X	BEND2	HGNC Symbol	BEN domain containing 2 [Source:HGNC Symbol;Acc:28509]
rs5909419	X	BEND2	HGNC Symbol	BEN domain containing 2 [Source:HGNC Symbol;Acc:28509]
rs5909165	X	BEND2	HGNC Symbol	BEN domain containing 2 [Source:HGNC Symbol;Acc:28509]
rs35401330	X	BEND2	HGNC Symbol	BEN domain containing 2 [Source:HGNC Symbol;Acc:28509]
rs17274127	X	BEND2	HGNC Symbol	BEN domain containing 2 [Source:HGNC Symbol;Acc:28509]
rs41309701	X	SCML2	HGNC Symbol	sex comb on midleg-like 2 (Drosophila) [Source:HGNC Symbol;Acc:10581]
rs137936150	X	SCML2	HGNC Symbol	sex comb on midleg-like 2 (Drosophila) [Source:HGNC Symbol;Acc:10581]
rs16980766	X	SCML2	HGNC Symbol	sex comb on midleg-like 2 (Drosophila) [Source:HGNC Symbol;Acc:10581]
rs5955962	X	SCML2	HGNC Symbol	sex comb on midleg-like 2 (Drosophila) [Source:HGNC Symbol;Acc:10581]
rs16997224	X	SCML2	HGNC Symbol	sex comb on midleg-like 2 (Drosophila) [Source:HGNC Symbol;Acc:10581]
rs145926052	X	SCML2	HGNC Symbol	sex comb on midleg-like 2 (Drosophila) [Source:HGNC Symbol;Acc:10581]
rs5909473				
rs5955621	X	CDKL5	HGNC Symbol	cyclin-dependent kinase-like 5 [Source:HGNC Symbol;Acc:11411]
rs150832645	X	CDKL5	HGNC Symbol	cyclin-dependent kinase-like 5 [Source:HGNC Symbol;Acc:11411]
rs56245820	X	CDKL5	HGNC Symbol	cyclin-dependent kinase-like 5 [Source:HGNC Symbol;Acc:11411]
rs146009942	X	CDKL5	HGNC Symbol	cyclin-dependent kinase-like 5 [Source:HGNC Symbol;Acc:11411]
rs73191522	X	CDKL5	HGNC Symbol	cyclin-dependent kinase-like 5 [Source:HGNC Symbol;Acc:11411]
rs5909188	X	CDKL5	HGNC Symbol	cyclin-dependent kinase-like 5 [Source:HGNC Symbol;Acc:11411]
rs112170850	X	CDKL5	HGNC Symbol	cyclin-dependent kinase-like 5 [Source:HGNC Symbol;Acc:11411]

SNP	Chromosome	gene name	gene source	description
rs144801554	X	CDKL5	HGNC Symbol	cyclin-dependent kinase-like 5 [Source:HGNC Symbol;Acc:11411]
rs137913310	X	CDKL5	HGNC Symbol	cyclin-dependent kinase-like 5 [Source:HGNC Symbol;Acc:11411]
rs73191523	X	CDKL5	HGNC Symbol	cyclin-dependent kinase-like 5 [Source:HGNC Symbol;Acc:11411]
rs4825261	X	CDKL5	HGNC Symbol	cyclin-dependent kinase-like 5 [Source:HGNC Symbol;Acc:11411]
rs73191527	X	CDKL5	HGNC Symbol	cyclin-dependent kinase-like 5 [Source:HGNC Symbol;Acc:11411]
rs35478150	X	CDKL5	HGNC Symbol	cyclin-dependent kinase-like 5 [Source:HGNC Symbol;Acc:11411]
rs2238952	X	CDKL5;RS1	HGNC Symbol	cyclin-dependent kinase-like 5 [Source:HGNC Symbol;Acc:11411];retinoschisin 1 [Source:HGNC Symbol;Acc:10457]
rs763051	X	RS1	HGNC Symbol	retinoschisin 1 [Source:HGNC Symbol;Acc:10457]
rs5909071	X	RS1	HGNC Symbol	retinoschisin 1 [Source:HGNC Symbol;Acc:10457]
rs5955504	X	RS1	HGNC Symbol	retinoschisin 1 [Source:HGNC Symbol;Acc:10457]
rs17247222	X	RS1	HGNC Symbol	retinoschisin 1 [Source:HGNC Symbol;Acc:10457]
rs6633114	X	PPEF1	HGNC Symbol	protein phosphatase, EF-hand calcium binding domain 1 [Source:HGNC Symbol;Acc:9243]
rs17247243	X	PPEF1;PPEF1-AS1	HGNC Symbol	protein phosphatase, EF-hand calcium binding domain 1 [Source:HGNC Symbol;Acc:9243];PPEF1 antisense RNA 1 [Source:HGNC Symbol;Acc:40463]
rs73191533	X	PPEF1	HGNC Symbol	protein phosphatase, EF-hand calcium binding domain 1 [Source:HGNC Symbol;Acc:9243]
rs5909202	X	PPEF1	HGNC Symbol	protein phosphatase, EF-hand calcium binding domain 1 [Source:HGNC Symbol;Acc:9243]
rs5909213	X	PPEF1	HGNC Symbol	protein phosphatase, EF-hand calcium binding domain 1 [Source:HGNC Symbol;Acc:9243]
rs5955635	X	PPEF1	HGNC Symbol	protein phosphatase, EF-hand calcium binding domain 1 [Source:HGNC Symbol;Acc:9243]
rs5955643	X	PPEF1	HGNC Symbol	protein phosphatase, EF-hand calcium binding domain 1 [Source:HGNC Symbol;Acc:9243]
rs73191552	X	PPEF1	HGNC Symbol	protein phosphatase, EF-hand calcium binding domain 1 [Source:HGNC Symbol;Acc:9243]
rs56226817	X	PPEF1	HGNC Symbol	protein phosphatase, EF-hand calcium binding domain 1 [Source:HGNC Symbol;Acc:9243]
rs2239439	X	PPEF1	HGNC Symbol	protein phosphatase, EF-hand calcium binding domain 1 [Source:HGNC Symbol;Acc:9243]
rs11796620	X	PPEF1	HGNC Symbol	protein phosphatase, EF-hand calcium binding domain 1 [Source:HGNC Symbol;Acc:9243]
rs2239437	X	PPEF1	HGNC Symbol	protein phosphatase, EF-hand calcium binding domain 1 [Source:HGNC Symbol;Acc:9243]
rs239740				
rs5955649				
rs239757				
rs239767				
rs5909256				
rs239746				
rs239750				
rs12388456				
rs73191588	X	PHKA2	HGNC Symbol	phosphorylase kinase, alpha 2 (liver) [Source:HGNC Symbol;Acc:8926]

SNP	Chromosome	gene name	gene source	description
rs6633170	X	PHKA2	HGNC Symbol	phosphorylase kinase, alpha 2 (liver) [Source:HGNC Symbol;Acc:8926]
rs16980924	X	PHKA2	HGNC Symbol	phosphorylase kinase, alpha 2 (liver) [Source:HGNC Symbol;Acc:8926]
rs17313504	X	GPR64	HGNC Symbol	G protein-coupled receptor 64 [Source:HGNC Symbol;Acc:4516]
rs12847500	X	GPR64	HGNC Symbol	G protein-coupled receptor 64 [Source:HGNC Symbol;Acc:4516]
rs60594383	X	GPR64	HGNC Symbol	G protein-coupled receptor 64 [Source:HGNC Symbol;Acc:4516]
rs41304719	X	GPR64	HGNC Symbol	G protein-coupled receptor 64 [Source:HGNC Symbol;Acc:4516]
rs41308443	X	GPR64	HGNC Symbol	G protein-coupled receptor 64 [Source:HGNC Symbol;Acc:4516]
rs6629310	X	GPR64	HGNC Symbol	G protein-coupled receptor 64 [Source:HGNC Symbol;Acc:4516]
rs73445661	X	GPR64	HGNC Symbol	G protein-coupled receptor 64 [Source:HGNC Symbol;Acc:4516]
rs5955677	X	GPR64	HGNC Symbol	G protein-coupled receptor 64 [Source:HGNC Symbol;Acc:4516]
rs73191598	X	GPR64	HGNC Symbol	G protein-coupled receptor 64 [Source:HGNC Symbol;Acc:4516]
rs140709021	X	GPR64	HGNC Symbol	G protein-coupled receptor 64 [Source:HGNC Symbol;Acc:4516]
rs4825283	X	GPR64	HGNC Symbol	G protein-coupled receptor 64 [Source:HGNC Symbol;Acc:4516]
rs73447624	X	GPR64	HGNC Symbol	G protein-coupled receptor 64 [Source:HGNC Symbol;Acc:4516]
rs5955693	X	GPR64	HGNC Symbol	G protein-coupled receptor 64 [Source:HGNC Symbol;Acc:4516]
rs113371478	X	GPR64	HGNC Symbol	G protein-coupled receptor 64 [Source:HGNC Symbol;Acc:4516]
rs6633197	X	GPR64	HGNC Symbol	G protein-coupled receptor 64 [Source:HGNC Symbol;Acc:4516]
rs5955695	X	GPR64	HGNC Symbol	G protein-coupled receptor 64 [Source:HGNC Symbol;Acc:4516]
rs5955701				
rs35989739				
rs113207257				
rs5955718				
rs5955719				
rs5955537				
rs145267692				
rs960190				
rs5955732				
rs12010568				
rs1870289				
rs5955734				
rs5955735				
rs5955542				
rs6527907				

SNP	Chromosome	gene name	gene source	description
rs73637611				
rs6633216				
rs73637613				
rs957026				
rs73449597				
rs73193519				
rs7053743				
rs10218356				
rs6633229				
rs148536478				
rs7058360				
rs73193522				
rs7065106				
rs111232112				
rs7890403				
rs5955548	X	PDHA1	HGNC Symbol	pyruvate dehydrogenase (lipoamide) alpha 1 [Source:HGNC Symbol;Acc:8806]
rs17247716	X	PDHA1	HGNC Symbol	pyruvate dehydrogenase (lipoamide) alpha 1 [Source:HGNC Symbol;Acc:8806]
rs15943	X	PDHA1;MAP3K15	HGNC Symbol	pyruvate dehydrogenase (lipoamide) alpha 1 [Source:HGNC Symbol;Acc:8806];mitogen-activated protein kinase kinase kinase 15 [Source:HGNC Symbol;Acc:31689]
rs73637638	X	MAP3K15	HGNC Symbol	mitogen-activated protein kinase kinase kinase 15 [Source:HGNC Symbol;Acc:31689]
rs73193528	X	MAP3K15	HGNC Symbol	mitogen-activated protein kinase kinase kinase 15 [Source:HGNC Symbol;Acc:31689]
rs56212339	X	MAP3K15	HGNC Symbol	mitogen-activated protein kinase kinase kinase 15 [Source:HGNC Symbol;Acc:31689]
rs5909292	X	MAP3K15	HGNC Symbol	mitogen-activated protein kinase kinase kinase 15 [Source:HGNC Symbol;Acc:31689]
rs73193534	X	MAP3K15	HGNC Symbol	mitogen-activated protein kinase kinase kinase 15 [Source:HGNC Symbol;Acc:31689]
rs767597	X	MAP3K15	HGNC Symbol	mitogen-activated protein kinase kinase kinase 15 [Source:HGNC Symbol;Acc:31689]
rs5909299	X	MAP3K15	HGNC Symbol	mitogen-activated protein kinase kinase kinase 15 [Source:HGNC Symbol;Acc:31689]
rs6633268	X	MAP3K15	HGNC Symbol	mitogen-activated protein kinase kinase kinase 15 [Source:HGNC Symbol;Acc:31689]
rs60185379	X	MAP3K15	HGNC Symbol	mitogen-activated protein kinase kinase kinase 15 [Source:HGNC Symbol;Acc:31689]
rs6418729	X	MAP3K15	HGNC Symbol	mitogen-activated protein kinase kinase kinase 15 [Source:HGNC Symbol;Acc:31689]
rs6653645	X	MAP3K15	HGNC Symbol	mitogen-activated protein kinase kinase kinase 15 [Source:HGNC Symbol;Acc:31689]
rs4825302	X	MAP3K15	HGNC Symbol	mitogen-activated protein kinase kinase kinase 15 [Source:HGNC Symbol;Acc:31689]
rs5955796	X	MAP3K15	HGNC Symbol	mitogen-activated protein kinase kinase kinase 15 [Source:HGNC Symbol;Acc:31689]
rs7052536	X	MAP3K15	HGNC Symbol	mitogen-activated protein kinase kinase kinase 15 [Source:HGNC Symbol;Acc:31689]

SNP	Chromosome	gene name	gene source	description
rs5955562	X	MAP3K15	HGNC Symbol	mitogen-activated protein kinase kinase kinase 15 [Source:HGNC Symbol;Acc:31689]
rs5955806	X	MAP3K15	HGNC Symbol	mitogen-activated protein kinase kinase kinase 15 [Source:HGNC Symbol;Acc:31689]
rs73193543	X	MAP3K15	HGNC Symbol	mitogen-activated protein kinase kinase kinase 15 [Source:HGNC Symbol;Acc:31689]
rs142403021	X	SH3KBP1	HGNC Symbol	SH3-domain kinase binding protein 1 [Source:HGNC Symbol;Acc:13867]
rs4825304	X	SH3KBP1	HGNC Symbol	SH3-domain kinase binding protein 1 [Source:HGNC Symbol;Acc:13867]
rs55897193	X	SH3KBP1	HGNC Symbol	SH3-domain kinase binding protein 1 [Source:HGNC Symbol;Acc:13867]
rs149766460	X	SH3KBP1	HGNC Symbol	SH3-domain kinase binding protein 1 [Source:HGNC Symbol;Acc:13867]
rs5955816	X	SH3KBP1	HGNC Symbol	SH3-domain kinase binding protein 1 [Source:HGNC Symbol;Acc:13867]
rs5955819	X	SH3KBP1	HGNC Symbol	SH3-domain kinase binding protein 1 [Source:HGNC Symbol;Acc:13867]
rs73193551	X	SH3KBP1	HGNC Symbol	SH3-domain kinase binding protein 1 [Source:HGNC Symbol;Acc:13867]
rs5909324	X	SH3KBP1	HGNC Symbol	SH3-domain kinase binding protein 1 [Source:HGNC Symbol;Acc:13867]
rs112539346	X	SH3KBP1	HGNC Symbol	SH3-domain kinase binding protein 1 [Source:HGNC Symbol;Acc:13867]
rs35160730	X	SH3KBP1	HGNC Symbol	SH3-domain kinase binding protein 1 [Source:HGNC Symbol;Acc:13867]
rs73193555	X	SH3KBP1	HGNC Symbol	SH3-domain kinase binding protein 1 [Source:HGNC Symbol;Acc:13867]
rs148543908	X	SH3KBP1	HGNC Symbol	SH3-domain kinase binding protein 1 [Source:HGNC Symbol;Acc:13867]
rs767386	X	SH3KBP1	HGNC Symbol	SH3-domain kinase binding protein 1 [Source:HGNC Symbol;Acc:13867]
rs5909130	X	SH3KBP1	HGNC Symbol	SH3-domain kinase binding protein 1 [Source:HGNC Symbol;Acc:13867]
rs146288382	X	SH3KBP1	HGNC Symbol	SH3-domain kinase binding protein 1 [Source:HGNC Symbol;Acc:13867]
rs5909133	X	SH3KBP1	HGNC Symbol	SH3-domain kinase binding protein 1 [Source:HGNC Symbol;Acc:13867]
rs73193559	X	SH3KBP1	HGNC Symbol	SH3-domain kinase binding protein 1 [Source:HGNC Symbol;Acc:13867]
rs7060796	X	CXorf23	HGNC Symbol	chromosome X open reading frame 23 [Source:HGNC Symbol;Acc:27413]
rs5909346	X	CXorf23	HGNC Symbol	chromosome X open reading frame 23 [Source:HGNC Symbol;Acc:27413]
rs5955871				
rs41305209	X	MAP7D2	HGNC Symbol	MAP7 domain containing 2 [Source:HGNC Symbol;Acc:25899]
rs41305207	X	MAP7D2	HGNC Symbol	MAP7 domain containing 2 [Source:HGNC Symbol;Acc:25899]
rs11796633	X	MAP7D2	HGNC Symbol	MAP7 domain containing 2 [Source:HGNC Symbol;Acc:25899]
rs41311495	X	MAP7D2	HGNC Symbol	MAP7 domain containing 2 [Source:HGNC Symbol;Acc:25899]
rs34519770	X	MAP7D2	HGNC Symbol	MAP7 domain containing 2 [Source:HGNC Symbol;Acc:25899]
rs139845621	X	MAP7D2	HGNC Symbol	MAP7 domain containing 2 [Source:HGNC Symbol;Acc:25899]
rs6527960	X	MAP7D2	HGNC Symbol	MAP7 domain containing 2 [Source:HGNC Symbol;Acc:25899]
rs60849679	X	MAP7D2	HGNC Symbol	MAP7 domain containing 2 [Source:HGNC Symbol;Acc:25899]
rs13179	X	EIF1AX	HGNC Symbol	eukaryotic translation initiation factor 1A, X-linked [Source:HGNC Symbol;Acc:3250]
rs55635237	X	EIF1AX	HGNC Symbol	eukaryotic translation initiation factor 1A, X-linked [Source:HGNC Symbol;Acc:3250]

SNP	Chromosome	gene name	gene source	description
rs113934690	X	EIF1AX	HGNC Symbol	eukaryotic translation initiation factor 1A, X-linked [Source:HGNC Symbol;Acc:3250]
rs5955887	X	EIF1AX	HGNC Symbol	eukaryotic translation initiation factor 1A, X-linked [Source:HGNC Symbol;Acc:3250]
rs112202240	X	EIF1AX	HGNC Symbol	eukaryotic translation initiation factor 1A, X-linked [Source:HGNC Symbol;Acc:3250]
rs7049476	X	RPS6KA3	HGNC Symbol	ribosomal protein S6 kinase, 90kDa, polypeptide 3 [Source:HGNC Symbol;Acc:10432]
rs143519267	X	RPS6KA3	HGNC Symbol	ribosomal protein S6 kinase, 90kDa, polypeptide 3 [Source:HGNC Symbol;Acc:10432]
rs2230488	X	RPS6KA3	HGNC Symbol	ribosomal protein S6 kinase, 90kDa, polypeptide 3 [Source:HGNC Symbol;Acc:10432]
rs5909364	X	RPS6KA3	HGNC Symbol	ribosomal protein S6 kinase, 90kDa, polypeptide 3 [Source:HGNC Symbol;Acc:10432]
rs56218010	X	RPS6KA3	HGNC Symbol	ribosomal protein S6 kinase, 90kDa, polypeptide 3 [Source:HGNC Symbol;Acc:10432]
rs73631384				
rs7051726				
rs7472814				
rs73631390				
rs73447169				
rs62590490				
rs5950379				
rs5950380				
rs72620232				
rs5950296				
rs113848564				
rs11798156				
rs5950408				
rs73193590				
rs28410155				
rs7471538				
rs7054054				
rs5950309				
rs5950311				
rs12839481				
rs7881721				
rs58057535				
rs7057250				
rs7472089				
rs41335952				

SNP	Chromosome	gene name	gene source	description
rs922257				
rs73195314				
rs5950268				
rs5950269				
rs73195315				
rs7059647				
rs4969754				
rs5990881				
rs12394856				
rs72620238				
rs12854871				
rs6527994				
rs73197241				
rs73450881				
rs73197245				
rs36067419				
rs4969759				
rs57666057				
rs1523824				
rs5990819				
rs17313971				
rs5990821				
rs143954382				
rs150894090				
rs10856300				
rs12392347				
rs5990929				
rs1900730				
rs5990941				
rs147142129				
rs149079863				
rs5990957				
rs62590528				

SNP	Chromosome	gene name	gene source	description
rs12558964				
rs5990961				
rs5950391				
rs62590533				
rs73197270				
rs5990977				
rs73635509				
rs5990984				
rs1531597				
rs72620260				
rs73197276				
rs5991008				
rs5950407				
rs73197282				
rs9306760				
rs9645473				
rs143091476				
rs73197286				
rs5904568	X	CNKSR2	HGNC Symbol	connector enhancer of kinase suppressor of Ras 2 [Source:HGNC Symbol;Acc:19701]
rs73203336	X	CNKSR2	HGNC Symbol	connector enhancer of kinase suppressor of Ras 2 [Source:HGNC Symbol;Acc:19701]
rs62590551	X	CNKSR2	HGNC Symbol	connector enhancer of kinase suppressor of Ras 2 [Source:HGNC Symbol;Acc:19701]
rs5951448	X	CNKSR2	HGNC Symbol	connector enhancer of kinase suppressor of Ras 2 [Source:HGNC Symbol;Acc:19701]
rs5951449	X	CNKSR2	HGNC Symbol	connector enhancer of kinase suppressor of Ras 2 [Source:HGNC Symbol;Acc:19701]
rs6629410	X	CNKSR2	HGNC Symbol	connector enhancer of kinase suppressor of Ras 2 [Source:HGNC Symbol;Acc:19701]
rs73203337	X	CNKSR2	HGNC Symbol	connector enhancer of kinase suppressor of Ras 2 [Source:HGNC Symbol;Acc:19701]
rs141792706	X	CNKSR2	HGNC Symbol	connector enhancer of kinase suppressor of Ras 2 [Source:HGNC Symbol;Acc:19701]
rs73203340	X	CNKSR2	HGNC Symbol	connector enhancer of kinase suppressor of Ras 2 [Source:HGNC Symbol;Acc:19701]
rs112041473	X	CNKSR2	HGNC Symbol	connector enhancer of kinase suppressor of Ras 2 [Source:HGNC Symbol;Acc:19701]
rs7882377	X	CNKSR2	HGNC Symbol	connector enhancer of kinase suppressor of Ras 2 [Source:HGNC Symbol;Acc:19701]
rs5904487	X	CNKSR2	HGNC Symbol	connector enhancer of kinase suppressor of Ras 2 [Source:HGNC Symbol;Acc:19701]
rs112942819	X	CNKSR2	HGNC Symbol	connector enhancer of kinase suppressor of Ras 2 [Source:HGNC Symbol;Acc:19701]
rs5904577				
rs140079749				

SNP	Chromosome	gene name	gene source	description
rs4317707				
rs13328629				
rs5951620				
rs2382625	X	SMPX	HGNC Symbol	small muscle protein, X-linked [Source:HGNC Symbol;Acc:11122]
rs73453555	X	SMPX	HGNC Symbol	small muscle protein, X-linked [Source:HGNC Symbol;Acc:11122]
rs6633438	X	SMPX	HGNC Symbol	small muscle protein, X-linked [Source:HGNC Symbol;Acc:11122]
rs5904581	X	SMPX	HGNC Symbol	small muscle protein, X-linked [Source:HGNC Symbol;Acc:11122]
rs4824208	X	SMPX	HGNC Symbol	small muscle protein, X-linked [Source:HGNC Symbol;Acc:11122]
rs5951463				
rs144472779				
rs5951634				
rs7878576				
rs141230888				
rs142371700				
rs10284169				
rs3213451	X	MBTPS2	HGNC Symbol	membrane-bound transcription factor peptidase, site 2 [Source:HGNC Symbol;Acc:15455]
rs5951640	X	MBTPS2	HGNC Symbol	membrane-bound transcription factor peptidase, site 2 [Source:HGNC Symbol;Acc:15455]
rs4446856	X	MBTPS2	HGNC Symbol	membrane-bound transcription factor peptidase, site 2 [Source:HGNC Symbol;Acc:15455]
rs56079719	X	MBTPS2	HGNC Symbol	membrane-bound transcription factor peptidase, site 2 [Source:HGNC Symbol;Acc:15455]
rs5951476	X	MBTPS2	HGNC Symbol	membrane-bound transcription factor peptidase, site 2 [Source:HGNC Symbol;Acc:15455]
rs6633480				
rs2361662				
rs73635585				
rs5951668				
rs5951487	X	LL0XNC01-39B3.1	Clone-based (Vega) gene	
rs73195164	X	SMS	HGNC Symbol	spermine synthase [Source:HGNC Symbol;Acc:11123]
rs6629443	X	SMS	HGNC Symbol	spermine synthase [Source:HGNC Symbol;Acc:11123]
rs2283723	X	SMS	HGNC Symbol	spermine synthase [Source:HGNC Symbol;Acc:11123]
rs62591198	X	SMS	HGNC Symbol	spermine synthase [Source:HGNC Symbol;Acc:11123]
rs150900156	X	SMS	HGNC Symbol	spermine synthase [Source:HGNC Symbol;Acc:11123]
rs1009643	X	SMS	HGNC Symbol	spermine synthase [Source:HGNC Symbol;Acc:11123]
rs2071136	X	SMS	HGNC Symbol	spermine synthase [Source:HGNC Symbol;Acc:11123]

SNP	Chromosome	gene name	gene source	description
rs4824216	X	SMS	HGNC Symbol	spermine synthase [Source:HGNC Symbol;Acc:11123]
rs73195180	X	SMS	HGNC Symbol	spermine synthase [Source:HGNC Symbol;Acc:11123]
rs5904600	X	SMS	HGNC Symbol	spermine synthase [Source:HGNC Symbol;Acc:11123]
rs17248500	X	SMS	HGNC Symbol	spermine synthase [Source:HGNC Symbol;Acc:11123]
rs2664858				
rs6418752				
rs12009915				
rs178702				
rs178710	X	PHEX	HGNC Symbol	phosphate regulating endopeptidase homolog, X-linked [Source:HGNC Symbol;Acc:8918]
rs5951494	X	PHEX	HGNC Symbol	phosphate regulating endopeptidase homolog, X-linked [Source:HGNC Symbol;Acc:8918]
rs2071201	X	PHEX	HGNC Symbol	phosphate regulating endopeptidase homolog, X-linked [Source:HGNC Symbol;Acc:8918]
rs2301304	X	PHEX	HGNC Symbol	phosphate regulating endopeptidase homolog, X-linked [Source:HGNC Symbol;Acc:8918]
rs178720	X	PHEX	HGNC Symbol	phosphate regulating endopeptidase homolog, X-linked [Source:HGNC Symbol;Acc:8918]
rs59949485	X	PHEX	HGNC Symbol	phosphate regulating endopeptidase homolog, X-linked [Source:HGNC Symbol;Acc:8918]
rs6633508	X	PHEX	HGNC Symbol	phosphate regulating endopeptidase homolog, X-linked [Source:HGNC Symbol;Acc:8918]
rs2301305	X	PHEX	HGNC Symbol	phosphate regulating endopeptidase homolog, X-linked [Source:HGNC Symbol;Acc:8918]
rs73201128	X	PHEX	HGNC Symbol	phosphate regulating endopeptidase homolog, X-linked [Source:HGNC Symbol;Acc:8918]
rs5904506	X	PHEX	HGNC Symbol	phosphate regulating endopeptidase homolog, X-linked [Source:HGNC Symbol;Acc:8918]
rs73201129	X	PHEX	HGNC Symbol	phosphate regulating endopeptidase homolog, X-linked [Source:HGNC Symbol;Acc:8918]
rs35657111	X	PHEX	HGNC Symbol	phosphate regulating endopeptidase homolog, X-linked [Source:HGNC Symbol;Acc:8918]
rs73201130	X	PHEX	HGNC Symbol	phosphate regulating endopeptidase homolog, X-linked [Source:HGNC Symbol;Acc:8918]
rs2071585	X	PHEX	HGNC Symbol	phosphate regulating endopeptidase homolog, X-linked [Source:HGNC Symbol;Acc:8918]
rs67041968	X	PHEX	HGNC Symbol	phosphate regulating endopeptidase homolog, X-linked [Source:HGNC Symbol;Acc:8918]
rs111284939	X	PHEX	HGNC Symbol	phosphate regulating endopeptidase homolog, X-linked [Source:HGNC Symbol;Acc:8918]
rs2301311	X	PHEX	HGNC Symbol	phosphate regulating endopeptidase homolog, X-linked [Source:HGNC Symbol;Acc:8918]
rs2362538	X	PHEX	HGNC Symbol	phosphate regulating endopeptidase homolog, X-linked [Source:HGNC Symbol;Acc:8918]
rs6528091	X	PHEX	HGNC Symbol	phosphate regulating endopeptidase homolog, X-linked [Source:HGNC Symbol;Acc:8918]
rs5951502	X	PHEX	HGNC Symbol	phosphate regulating endopeptidase homolog, X-linked [Source:HGNC Symbol;Acc:8918]
rs2285066	X	PHEX	HGNC Symbol	phosphate regulating endopeptidase homolog, X-linked [Source:HGNC Symbol;Acc:8918]
rs16981757	X	PHEX	HGNC Symbol	phosphate regulating endopeptidase homolog, X-linked [Source:HGNC Symbol;Acc:8918]
rs5951701	X	PHEX	HGNC Symbol	phosphate regulating endopeptidase homolog, X-linked [Source:HGNC Symbol;Acc:8918]
rs5951703	X	PHEX	HGNC Symbol	phosphate regulating endopeptidase homolog, X-linked [Source:HGNC Symbol;Acc:8918]
rs1540282	X	PHEX	HGNC Symbol	phosphate regulating endopeptidase homolog, X-linked [Source:HGNC Symbol;Acc:8918]

SNP	Chromosome	gene name	gene source	description
rs1540283	X	PHEX	HGNC Symbol	phosphate regulating endopeptidase homolog, X-linked [Source:HGNC Symbol;Acc:8918]
rs2269466	X	PHEX	HGNC Symbol	phosphate regulating endopeptidase homolog, X-linked [Source:HGNC Symbol;Acc:8918]
rs5951505	X	PHEX	HGNC Symbol	phosphate regulating endopeptidase homolog, X-linked [Source:HGNC Symbol;Acc:8918]
rs12559632	X	PHEX	HGNC Symbol	phosphate regulating endopeptidase homolog, X-linked [Source:HGNC Symbol;Acc:8918]
rs4824228	X	PHEX	HGNC Symbol	phosphate regulating endopeptidase homolog, X-linked [Source:HGNC Symbol;Acc:8918]
rs5951709	X	PHEX	HGNC Symbol	phosphate regulating endopeptidase homolog, X-linked [Source:HGNC Symbol;Acc:8918]
rs6633525	X	PHEX	HGNC Symbol	phosphate regulating endopeptidase homolog, X-linked [Source:HGNC Symbol;Acc:8918]
rs73462666	X	PHEX	HGNC Symbol	phosphate regulating endopeptidase homolog, X-linked [Source:HGNC Symbol;Acc:8918]
rs10126508	X	PHEX;PHEX-AS1	HGNC Symbol	phosphate regulating endopeptidase homolog, X-linked [Source:HGNC Symbol;Acc:8918];PHEX antisense RNA 1 [Source:HGNC Symbol;Acc:40445]
rs113122599	X	PHEX	HGNC Symbol	phosphate regulating endopeptidase homolog, X-linked [Source:HGNC Symbol;Acc:8918]
rs5951718	X	PHEX	HGNC Symbol	phosphate regulating endopeptidase homolog, X-linked [Source:HGNC Symbol;Acc:8918]
rs149501103	X	PHEX	HGNC Symbol	phosphate regulating endopeptidase homolog, X-linked [Source:HGNC Symbol;Acc:8918]
rs12396173	X	PHEX	HGNC Symbol	phosphate regulating endopeptidase homolog, X-linked [Source:HGNC Symbol;Acc:8918]
rs5951723	X	PHEX	HGNC Symbol	phosphate regulating endopeptidase homolog, X-linked [Source:HGNC Symbol;Acc:8918]
rs6629454	X	PHEX	HGNC Symbol	phosphate regulating endopeptidase homolog, X-linked [Source:HGNC Symbol;Acc:8918]
rs6654106	X	PHEX	HGNC Symbol	phosphate regulating endopeptidase homolog, X-linked [Source:HGNC Symbol;Acc:8918]
rs2285071	X	PHEX	HGNC Symbol	phosphate regulating endopeptidase homolog, X-linked [Source:HGNC Symbol;Acc:8918]
rs3213493	X	PHEX	HGNC Symbol	phosphate regulating endopeptidase homolog, X-linked [Source:HGNC Symbol;Acc:8918]
rs2285073	X	PHEX	HGNC Symbol	phosphate regulating endopeptidase homolog, X-linked [Source:HGNC Symbol;Acc:8918]
rs5904625	X	PHEX	HGNC Symbol	phosphate regulating endopeptidase homolog, X-linked [Source:HGNC Symbol;Acc:8918]
rs5951729	X	PHEX	HGNC Symbol	phosphate regulating endopeptidase homolog, X-linked [Source:HGNC Symbol;Acc:8918]
rs62584917	X	PHEX	HGNC Symbol	phosphate regulating endopeptidase homolog, X-linked [Source:HGNC Symbol;Acc:8918]
rs112585845	X	PHEX	HGNC Symbol	phosphate regulating endopeptidase homolog, X-linked [Source:HGNC Symbol;Acc:8918]
rs7056269				
rs12397283	X	RP11-40F8.2	Clone-based (Vega) gene	
rs7877302	X	RP11-40F8.2	Clone-based (Vega) gene	
rs5951426	X	ZNF645;RP11-40F8.2	HGNC Symbol;Clone-based (Vega) gene	zinc finger protein 645 [Source:HGNC Symbol;Acc:26371];
rs5951547	X	RP11-40F8.2	Clone-based (Vega) gene	

SNP	Chromosome	gene name	gene source	description
rs6418753	X	RP11-40F8.2	Clone-based (Vega) gene	
rs5951423	X	RP11-40F8.2	Clone-based (Vega) gene	
rs4373645	X	RP11-40F8.2	Clone-based (Vega) gene	
rs4439258	X	RP11-40F8.2	Clone-based (Vega) gene	
rs17248934	X	RP11-40F8.2	Clone-based (Vega) gene	
rs5951572	X	RP11-40F8.2	Clone-based (Vega) gene	
rs5951571	X	RP11-40F8.2	Clone-based (Vega) gene	
rs5951570	X	RP11-40F8.2	Clone-based (Vega) gene	
rs11094829	X	RP11-40F8.2	Clone-based (Vega) gene	
rs4075693	X	RP11-40F8.2	Clone-based (Vega) gene	
rs149291302	X	RP11-40F8.2	Clone-based (Vega) gene	
rs62583904	X	RP11-40F8.2	Clone-based (Vega) gene	
rs6633548	X	RP11-40F8.2	Clone-based (Vega) gene	
rs4824185	X	RP11-40F8.2	Clone-based (Vega) gene	
rs35754602	X	RP11-40F8.2	Clone-based (Vega) gene	
rs67468133	X	RP11-40F8.2	Clone-based (Vega) gene	
rs62583948	X	RP11-40F8.2	Clone-based (Vega) gene	
rs68050094	X	RP11-40F8.2	Clone-based (Vega) gene	
rs55904413	X	RP11-40F8.2	Clone-based (Vega) gene	
rs4636328	X	RP11-40F8.2	Clone-based (Vega) gene	

SNP	Chromosome	gene name	gene source	description
rs4824168	X	RP11-40F8.2	Clone-based (Vega) gene	
rs62585985	X	RP11-40F8.2	Clone-based (Vega) gene	
rs7891648	X	RP11-40F8.2	Clone-based (Vega) gene	
rs56196258	X	RP11-40F8.2	Clone-based (Vega) gene	
rs5951584	X	RP11-40F8.2	Clone-based (Vega) gene	
rs73200727	X	RP11-40F8.2	Clone-based (Vega) gene	
rs60657867	X	RP11-40F8.2	Clone-based (Vega) gene	
rs5904557	X	RP11-40F8.2	Clone-based (Vega) gene	
rs6633571	X	RP11-40F8.2	Clone-based (Vega) gene	
rs138942282	X	RP11-40F8.2	Clone-based (Vega) gene	
rs7057115	X	RP11-494I9.2;RP11-40F8.2	Clone-based (Vega) gene	
rs67802966	X	RP11-494I9.2;RP11-40F8.2	Clone-based (Vega) gene	
rs12399184	X	RP11-494I9.2;RP11-40F8.2	Clone-based (Vega) gene	
rs56372937	X	RP11-494I9.2;RP11-40F8.2	Clone-based (Vega) gene	
rs7886014	X	RP11-494I9.2;RP11-40F8.2	Clone-based (Vega) gene	
rs10498927	X	RP11-494I9.2;RP11-40F8.2	Clone-based (Vega) gene	
rs1507791	X	RP11-494I9.2;RP11-40F8.2	Clone-based (Vega) gene	
rs113677646	X	RP11-494I9.2;RP11-40F8.2	Clone-based (Vega) gene	
rs12834145	X	RP11-494I9.2;RP11-40F8.2	Clone-based (Vega) gene	
rs5970909	X	RP11-494I9.2;RP11-40F8.2	Clone-based (Vega) gene	

SNP	Chromosome	gene name	gene source	description
rs17343095	X	RP11-40F8.2	Clone-based (Vega) gene	
rs62584043	X	RP11-40F8.2	Clone-based (Vega) gene	
rs59006248	X	RP11-40F8.2	Clone-based (Vega) gene	
rs73209905	X	RP11-40F8.2	Clone-based (Vega) gene	
rs5925723	X	RP11-40F8.2	Clone-based (Vega) gene	
rs5926232	X	RP11-40F8.2	Clone-based (Vega) gene	
rs150486959	X	RP11-40F8.2	Clone-based (Vega) gene	
rs56792135	X	RP11-40F8.2	Clone-based (Vega) gene	
rs12859112	X	RP11-40F8.2	Clone-based (Vega) gene	
rs5925810	X	RP11-40F8.2	Clone-based (Vega) gene	
rs73630766	X	RP11-40F8.2	Clone-based (Vega) gene	
rs61001966	X	RP11-40F8.2	Clone-based (Vega) gene	
rs5925912	X	RP11-40F8.2	Clone-based (Vega) gene	
rs57280896	X	RP11-40F8.2	Clone-based (Vega) gene	
rs115751957	X	RP11-40F8.2	Clone-based (Vega) gene	
rs66628089	X	RP11-40F8.2	Clone-based (Vega) gene	
rs9919095	X	RP11-40F8.2	Clone-based (Vega) gene	
rs5926008	X	RP11-494I9.1;RP11-40F8.2	Clone-based (Vega) gene	
rs16982160	X	RP11-40F8.2	Clone-based (Vega) gene	
rs6633594	X	RP11-40F8.2	Clone-based (Vega) gene	

SNP	Chromosome	gene name	gene source	description
rs5926019	X	RP11-40F8.2	Clone-based (Vega) gene	
rs16982185	X	RP11-40F8.2	Clone-based (Vega) gene	
rs58109113	X	RP11-40F8.2	Clone-based (Vega) gene	
rs58557634	X	RP11-40F8.2	Clone-based (Vega) gene	
rs112505478	X	RP11-40F8.2	Clone-based (Vega) gene	
rs148067302	X	RP11-40F8.2	Clone-based (Vega) gene	
rs6633599	X	RP11-40F8.2	Clone-based (Vega) gene	
rs61463999	X	RP11-40F8.2	Clone-based (Vega) gene	
rs58051838	X	RP11-40F8.2	Clone-based (Vega) gene	
rs12557952	X	RP11-40F8.2	Clone-based (Vega) gene	
rs3885948	X	RP11-40F8.2;GS1-433O24.1	Clone-based (Vega) gene	
rs4345750	X	RP11-40F8.2;GS1-433O24.1	Clone-based (Vega) gene	
rs7059086	X	RP11-40F8.2;GS1-433O24.1	Clone-based (Vega) gene	
rs6629484	X	RP11-40F8.2;GS1-433O24.1	Clone-based (Vega) gene	
rs4484871	X	RP11-40F8.2;GS1-433O24.1	Clone-based (Vega) gene	
rs5970873	X	RP11-40F8.2;GS1-433O24.1	Clone-based (Vega) gene	
rs916946	X	RP11-40F8.2;GS1-433O24.1	Clone-based (Vega) gene	
rs2057758	X	RP11-40F8.2;GS1-433O24.1	Clone-based (Vega) gene	
rs62585789	X	RP11-40F8.2;GS1-433O24.1	Clone-based (Vega) gene	
rs986324	X	RP11-40F8.2;GS1-433O24.1	Clone-based (Vega) gene	

SNP	Chromosome	gene name	gene source	description
rs2214521	X	RP11-40F8.2;GS1-433O24.1	Clone-based (Vega) gene	
rs2189486	X	RP11-40F8.2;GS1-433O24.1	Clone-based (Vega) gene	
rs146375882	X	RP11-40F8.2;GS1-433O24.1	Clone-based (Vega) gene	
rs5926053	X	RP11-40F8.2;GS1-433O24.1	Clone-based (Vega) gene	
rs148791795	X	RP11-40F8.2;GS1-433O24.1	Clone-based (Vega) gene	
rs7057694	X	RP11-40F8.2;GS1-433O24.1	Clone-based (Vega) gene	
rs58369678	X	RP11-40F8.2	Clone-based (Vega) gene	
rs57890705	X	RP11-40F8.2	Clone-based (Vega) gene	
rs12556950	X	RP11-40F8.2	Clone-based (Vega) gene	
rs5926057	X	RP11-40F8.2	Clone-based (Vega) gene	
rs6528188	X	RP11-40F8.2	Clone-based (Vega) gene	
rs4828923	X	RP11-40F8.2	Clone-based (Vega) gene	
rs5925668	X	RP11-40F8.2	Clone-based (Vega) gene	
rs13447314	X	RP11-40F8.2	Clone-based (Vega) gene	
rs5970898	X	RP11-40F8.2	Clone-based (Vega) gene	
rs34934530	X	RP11-40F8.2	Clone-based (Vega) gene	
rs66476440	X	RP11-40F8.2	Clone-based (Vega) gene	
rs6633655	X	RP11-40F8.2	Clone-based (Vega) gene	
rs2107306	X	RP11-40F8.2	Clone-based (Vega) gene	
rs6633672	X	RP11-40F8.2	Clone-based (Vega) gene	

SNP	Chromosome	gene name	gene source	description
rs2214519	X	RP11-40F8.2	Clone-based (Vega) gene	
rs11094865	X	RP11-40F8.2	Clone-based (Vega) gene	
rs6528201	X	RP11-40F8.2	Clone-based (Vega) gene	
rs5926097	X	RP11-40F8.2	Clone-based (Vega) gene	
rs5926098	X	RP11-40F8.2	Clone-based (Vega) gene	
rs5970651	X	RP11-40F8.2	Clone-based (Vega) gene	
rs12835979	X	RP11-40F8.2	Clone-based (Vega) gene	
rs5926110	X	RP11-40F8.2	Clone-based (Vega) gene	
rs962072	X	RP11-40F8.2	Clone-based (Vega) gene	
rs66666501	X	RP11-40F8.2	Clone-based (Vega) gene	
rs5925696	X	RP11-40F8.2	Clone-based (Vega) gene	
rs12849889	X	RP11-40F8.2	Clone-based (Vega) gene	
rs34927890	X	RP11-40F8.2	Clone-based (Vega) gene	
rs5926147	X	RP11-40F8.2	Clone-based (Vega) gene	
rs5970662	X	RP11-40F8.2	Clone-based (Vega) gene	
rs10046957	X	RP11-40F8.2	Clone-based (Vega) gene	
rs59339804	X	RP11-40F8.2	Clone-based (Vega) gene	
rs5970935	X	RP11-40F8.2	Clone-based (Vega) gene	
rs76678492	X	RP11-40F8.2	Clone-based (Vega) gene	
rs5926152	X	RP11-40F8.2	Clone-based (Vega) gene	

SNP	Chromosome	gene name	gene source	description
rs56053433	X	RP11-40F8.2	Clone-based (Vega) gene	
rs66532973	X	RP11-40F8.2	Clone-based (Vega) gene	
rs5970940	X	RP11-40F8.2	Clone-based (Vega) gene	
rs10126959	X	RP11-40F8.2	Clone-based (Vega) gene	
rs964467	X	RP11-40F8.2	Clone-based (Vega) gene	
rs56201502	X	RP11-40F8.2	Clone-based (Vega) gene	
rs5970946	X	RP11-40F8.2	Clone-based (Vega) gene	
rs73198923	X	RP11-40F8.2	Clone-based (Vega) gene	
rs5970947	X	RP11-40F8.2	Clone-based (Vega) gene	
rs73198927	X	RP11-40F8.2	Clone-based (Vega) gene	
rs6629518	X	RP11-40F8.2	Clone-based (Vega) gene	
rs6629519	X	RP11-40F8.2	Clone-based (Vega) gene	
rs5925704	X	RP11-40F8.2	Clone-based (Vega) gene	
rs4828946	X	RP11-40F8.2	Clone-based (Vega) gene	
rs113294707	X	RP11-40F8.2	Clone-based (Vega) gene	
rs11094875	X	RP11-40F8.2	Clone-based (Vega) gene	
rs5926178	X	RP11-40F8.2	Clone-based (Vega) gene	
rs5926181	X	RP11-40F8.2	Clone-based (Vega) gene	
rs11094876	X	RP11-40F8.2	Clone-based (Vega) gene	
rs6528234	X	RP11-40F8.2	Clone-based (Vega) gene	

SNP	Chromosome	gene name	gene source	description
rs111326022	X	RP11-40F8.2	Clone-based (Vega) gene	
rs5926184	X	RP11-40F8.2	Clone-based (Vega) gene	
rs4074765	X	RP11-40F8.2	Clone-based (Vega) gene	
rs7881783	X	RP11-40F8.2	Clone-based (Vega) gene	
rs5970959	X	RP11-40F8.2	Clone-based (Vega) gene	
rs5925713	X	RP11-40F8.2	Clone-based (Vega) gene	
rs73198936	X	RP11-40F8.2	Clone-based (Vega) gene	
rs12690030	X	RP11-40F8.2	Clone-based (Vega) gene	
rs6629527	X	RP11-40F8.2	Clone-based (Vega) gene	
rs5970962	X	RP11-40F8.2	Clone-based (Vega) gene	
rs1405775	X	RP11-40F8.2	Clone-based (Vega) gene	
rs12690411	X	RP11-40F8.2	Clone-based (Vega) gene	
rs5925717	X	RP11-40F8.2	Clone-based (Vega) gene	
rs5970671	X	RP11-40F8.2	Clone-based (Vega) gene	
rs4433305	X	RP11-40F8.2	Clone-based (Vega) gene	
rs34737763	X	DDX53;RP11-40F8.2	HGNC Symbol;Clone-based (Vega) gene	DEAD (Asp-Glu-Ala-Asp) box polypeptide 53 [Source:HGNC Symbol;Acc:20083];
rs5925720	X	DDX53;RP11-40F8.2	HGNC Symbol;Clone-based (Vega) gene	DEAD (Asp-Glu-Ala-Asp) box polypeptide 53 [Source:HGNC Symbol;Acc:20083];
rs5926203	X	DDX53;RP11-40F8.2	HGNC Symbol;Clone-based (Vega) gene	DEAD (Asp-Glu-Ala-Asp) box polypeptide 53 [Source:HGNC Symbol;Acc:20083];

SNP	Chromosome	gene name	gene source	description
rs5970975	X	RP11-40F8.2	Clone-based (Vega) gene	
rs6528242	X	RP11-40F8.2	Clone-based (Vega) gene	
rs4828955	X	RP11-40F8.2	Clone-based (Vega) gene	
rs5970980	X	RP11-40F8.2	Clone-based (Vega) gene	
rs4486354	X	RP11-40F8.2	Clone-based (Vega) gene	
rs5970987	X	RP11-40F8.2	Clone-based (Vega) gene	
rs12688383	X	RP11-40F8.2	Clone-based (Vega) gene	
rs62584150	X	RP11-40F8.2	Clone-based (Vega) gene	
rs73465050	X	RP11-40F8.2	Clone-based (Vega) gene	
rs73465063	X	RP11-40F8.2	Clone-based (Vega) gene	
rs17332480	X	RP11-40F8.2	Clone-based (Vega) gene	
rs11094885	X	RP11-40F8.2	Clone-based (Vega) gene	
rs11094888	X	RP11-40F8.2	Clone-based (Vega) gene	
rs115522617	X	RP11-40F8.2	Clone-based (Vega) gene	
rs12008467	X	RP11-40F8.2	Clone-based (Vega) gene	
rs2011288	X	RP11-40F8.2	Clone-based (Vega) gene	
rs73198955	X	RP11-40F8.2	Clone-based (Vega) gene	
rs73198957	X	RP11-40F8.2	Clone-based (Vega) gene	
rs138823455	X	RP11-40F8.2	Clone-based (Vega) gene	
rs16982449	X	RP11-40F8.2	Clone-based (Vega) gene	

SNP	Chromosome	gene name	gene source	description
rs2465827	X	RP11-40F8.2	Clone-based (Vega) gene	
rs16982450	X	RP11-40F8.2	Clone-based (Vega) gene	
rs5926223	X	RP11-40F8.2	Clone-based (Vega) gene	
rs5971034	X	RP11-40F8.2	Clone-based (Vega) gene	
rs16997566	X	RP11-40F8.2	Clone-based (Vega) gene	
rs978391	X	RP11-40F8.2	Clone-based (Vega) gene	
rs59142962	X	RP11-40F8.2	Clone-based (Vega) gene	
rs5926231	X	RP11-40F8.2	Clone-based (Vega) gene	
rs142914756	X	RP11-40F8.2	Clone-based (Vega) gene	
rs7471077	X	RP11-40F8.2	Clone-based (Vega) gene	
rs58351470	X	RP11-40F8.2	Clone-based (Vega) gene	
rs73198969	X	RP11-40F8.2	Clone-based (Vega) gene	
rs67994587	X	RP11-40F8.2	Clone-based (Vega) gene	
rs5971044	X	RP11-40F8.2	Clone-based (Vega) gene	
rs73478158	X	RP11-40F8.2	Clone-based (Vega) gene	
rs67040061	X	RP11-40F8.2	Clone-based (Vega) gene	
rs5926242	X	RP11-40F8.2	Clone-based (Vega) gene	
rs7056652	X	RP11-40F8.2	Clone-based (Vega) gene	
rs12556171	X	RP11-40F8.2	Clone-based (Vega) gene	
rs12689410	X	RP11-40F8.2	Clone-based (Vega) gene	

SNP	Chromosome	gene name	gene source	description
rs12013772	X	RP11-40F8.2	Clone-based (Vega) gene	
rs5926257	X	RP11-40F8.2	Clone-based (Vega) gene	
rs6526238	X	GS1-590J15.1;RP11-40F8.2	Clone-based (Vega) gene	
rs5970700	X	RP11-40F8.2	Clone-based (Vega) gene	
rs9887131	X	RP11-40F8.2	Clone-based (Vega) gene	
rs5926265	X	RP11-40F8.2	Clone-based (Vega) gene	
rs34378856	X	RP11-40F8.2	Clone-based (Vega) gene	
rs7880473	X	RP11-40F8.2	Clone-based (Vega) gene	
rs6526250	X	RP11-40F8.2	Clone-based (Vega) gene	
rs5926269	X	RP11-40F8.2	Clone-based (Vega) gene	
rs6627910	X	RP11-40F8.2	Clone-based (Vega) gene	
rs10284023	X	RP11-40F8.2	Clone-based (Vega) gene	
rs5971066	X	RP11-40F8.2	Clone-based (Vega) gene	
rs5971078	X	RP11-40F8.2	Clone-based (Vega) gene	
rs4828968	X	RP11-40F8.2	Clone-based (Vega) gene	
rs11094904	X	RP11-40F8.2	Clone-based (Vega) gene	
rs6629602	X	RP11-40F8.2	Clone-based (Vega) gene	
rs111778314	X	RP11-40F8.2	Clone-based (Vega) gene	
rs12560196	X	RP11-40F8.2	Clone-based (Vega) gene	
rs5971087	X	RP11-40F8.2	Clone-based (Vega) gene	

SNP	Chromosome	gene name	gene source	description
rs141819735	X	RP11-40F8.2	Clone-based (Vega) gene	
rs4828976	X	RP11-40F8.2	Clone-based (Vega) gene	
rs16997587	X	RP11-40F8.2	Clone-based (Vega) gene	
rs73205348	X	RP11-40F8.2	Clone-based (Vega) gene	
rs972834	X	RP11-40F8.2	Clone-based (Vega) gene	
rs5926280	X	RP11-40F8.2	Clone-based (Vega) gene	
rs5925750	X	RP11-40F8.2	Clone-based (Vega) gene	
rs5926281	X	RP11-40F8.2	Clone-based (Vega) gene	
rs10521926	X	RP11-40F8.2	Clone-based (Vega) gene	
rs6629611	X	RP11-40F8.2	Clone-based (Vega) gene	
rs56121665	X	RP11-40F8.2	Clone-based (Vega) gene	
rs4828981				
rs4828982				
rs757478				
rs886546				
rs17285804				
rs1527806				
rs11797678				
rs28429011				
rs17332584				
rs17347454				
rs12014412	X	PTCHD1	HGNC Symbol	patched domain containing 1 [Source:HGNC Symbol;Acc:26392]
rs7878766	X	PTCHD1	HGNC Symbol	patched domain containing 1 [Source:HGNC Symbol;Acc:26392]
rs146027747	X	PTCHD1	HGNC Symbol	patched domain containing 1 [Source:HGNC Symbol;Acc:26392]
rs12859195	X	PTCHD1	HGNC Symbol	patched domain containing 1 [Source:HGNC Symbol;Acc:26392]
rs11094909	X	PTCHD1	HGNC Symbol	patched domain containing 1 [Source:HGNC Symbol;Acc:26392]

SNP	Chromosome	gene name	gene source	description
rs1527811	X	PTCHD1	HGNC Symbol	patched domain containing 1 [Source:HGNC Symbol;Acc:26392]
rs7064366	X	PTCHD1	HGNC Symbol	patched domain containing 1 [Source:HGNC Symbol;Acc:26392]
rs1918560	X	PTCHD1	HGNC Symbol	patched domain containing 1 [Source:HGNC Symbol;Acc:26392]
rs7052177	X	PTCHD1	HGNC Symbol	patched domain containing 1 [Source:HGNC Symbol;Acc:26392]
rs62584517	X	PTCHD1	HGNC Symbol	patched domain containing 1 [Source:HGNC Symbol;Acc:26392]
rs5971108	X	PTCHD1	HGNC Symbol	patched domain containing 1 [Source:HGNC Symbol;Acc:26392]
rs1881055	X	PTCHD1	HGNC Symbol	patched domain containing 1 [Source:HGNC Symbol;Acc:26392]
rs5925760	X	PTCHD1	HGNC Symbol	patched domain containing 1 [Source:HGNC Symbol;Acc:26392]
rs12559982	X	PTCHD1	HGNC Symbol	patched domain containing 1 [Source:HGNC Symbol;Acc:26392]
rs5971110	X	PTCHD1	HGNC Symbol	patched domain containing 1 [Source:HGNC Symbol;Acc:26392]
rs5925761	X	PTCHD1	HGNC Symbol	patched domain containing 1 [Source:HGNC Symbol;Acc:26392]
rs4828859	X	PTCHD1	HGNC Symbol	patched domain containing 1 [Source:HGNC Symbol;Acc:26392]
rs10521927	X	PTCHD1	HGNC Symbol	patched domain containing 1 [Source:HGNC Symbol;Acc:26392]
rs5926303	X	PTCHD1	HGNC Symbol	patched domain containing 1 [Source:HGNC Symbol;Acc:26392]
rs5926304	X	PTCHD1	HGNC Symbol	patched domain containing 1 [Source:HGNC Symbol;Acc:26392]
rs73205366	X	PTCHD1	HGNC Symbol	patched domain containing 1 [Source:HGNC Symbol;Acc:26392]
rs73205373	X	PTCHD1	HGNC Symbol	patched domain containing 1 [Source:HGNC Symbol;Acc:26392]
rs5971114	X	PTCHD1	HGNC Symbol	patched domain containing 1 [Source:HGNC Symbol;Acc:26392]
rs7889469	X	PTCHD1	HGNC Symbol	patched domain containing 1 [Source:HGNC Symbol;Acc:26392]
rs12852420	X	PTCHD1	HGNC Symbol	patched domain containing 1 [Source:HGNC Symbol;Acc:26392]
rs3810719	X	PTCHD1	HGNC Symbol	patched domain containing 1 [Source:HGNC Symbol;Acc:26392]
rs150840243	X	PTCHD1	HGNC Symbol	patched domain containing 1 [Source:HGNC Symbol;Acc:26392]
rs73207441				
rs5926319				
rs5926321				
rs17285881				
rs4828990				
rs73473321				
rs10521930				
rs5925793				
rs6627937				
rs5925802				
rs11094927				

SNP	Chromosome	gene name	gene source	description
rs113771972				
rs5970590				
rs73207481				
rs73207482				
rs1033055				
rs55815505				
rs16982677				
rs6629697				
rs10521931				
rs5970593				
rs60561291				
rs55890684				
rs2202958				
rs5925582				
rs2202957				
rs137882247				
rs12400742				
rs955775				
rs533878				
rs75261547				
rs578606				
rs561909				
rs513573	X	PRDX4	HGNC Symbol	peroxiredoxin 4 [Source:HGNC Symbol;Acc:17169]
rs518329	X	PRDX4	HGNC Symbol	peroxiredoxin 4 [Source:HGNC Symbol;Acc:17169]
rs477233				
rs6629721				
rs2665363				
rs11175	X	ACOT9	HGNC Symbol	acyl-CoA thioesterase 9 [Source:HGNC Symbol;Acc:17152]
rs56378612	X	ACOT9	HGNC Symbol	acyl-CoA thioesterase 9 [Source:HGNC Symbol;Acc:17152]
rs5925594	X	ACOT9	HGNC Symbol	acyl-CoA thioesterase 9 [Source:HGNC Symbol;Acc:17152]
rs6526333	X	ACOT9	HGNC Symbol	acyl-CoA thioesterase 9 [Source:HGNC Symbol;Acc:17152]
rs12009956	X	ACOT9	HGNC Symbol	acyl-CoA thioesterase 9 [Source:HGNC Symbol;Acc:17152]
rs73209284	X	ACOT9	HGNC Symbol	acyl-CoA thioesterase 9 [Source:HGNC Symbol;Acc:17152]

SNP	Chromosome	gene name	gene source	description
rs5925887	X	ACOT9	HGNC Symbol	acyl-CoA thioesterase 9 [Source:HGNC Symbol;Acc:17152]
rs112323879	X	ACOT9	HGNC Symbol	acyl-CoA thioesterase 9 [Source:HGNC Symbol;Acc:17152]
rs59548719	X	ACOT9	HGNC Symbol	acyl-CoA thioesterase 9 [Source:HGNC Symbol;Acc:17152]
rs7066756	X	ACOT9	HGNC Symbol	acyl-CoA thioesterase 9 [Source:HGNC Symbol;Acc:17152]
rs17332771	X	ACOT9	HGNC Symbol	acyl-CoA thioesterase 9 [Source:HGNC Symbol;Acc:17152]
rs149531197	X	ACOT9	HGNC Symbol	acyl-CoA thioesterase 9 [Source:HGNC Symbol;Acc:17152]
rs72620454	X	ACOT9	HGNC Symbol	acyl-CoA thioesterase 9 [Source:HGNC Symbol;Acc:17152]
rs5925920	X	ACOT9	HGNC Symbol	acyl-CoA thioesterase 9 [Source:HGNC Symbol;Acc:17152]
rs933404	X	ACOT9	HGNC Symbol	acyl-CoA thioesterase 9 [Source:HGNC Symbol;Acc:17152]
rs5970790				
rs12689254				
rs5970791				
rs41305159				
rs34092038	X	RP13-314C10.5	Clone-based (Vega) gene	
rs58123806	X	RP13-314C10.5	Clone-based (Vega) gene	
rs12838869	X	RP13-314C10.5	Clone-based (Vega) gene	
rs6526342	X	RP13-314C10.5	Clone-based (Vega) gene	
rs928932	X	RP13-314C10.5	Clone-based (Vega) gene	
rs41305193	X	SAT1	HGNC Symbol	spermidine/spermine N1-acetyltransferase 1 [Source:HGNC Symbol;Acc:10540]
rs6627978				
rs6526347				
rs55877516				
rs545426401				
rs8680	X	APOO	HGNC Symbol	apolipoprotein O [Source:HGNC Symbol;Acc:28727]
rs112978835	X	APOO	HGNC Symbol	apolipoprotein O [Source:HGNC Symbol;Acc:28727]
rs4828835	X	APOO	HGNC Symbol	apolipoprotein O [Source:HGNC Symbol;Acc:28727]
rs2464715	X	CXorf58	HGNC Symbol	chromosome X open reading frame 58 [Source:HGNC Symbol;Acc:26356]
rs144408039				
rs138596629				
rs2520226				

SNP	Chromosome	gene name	gene source	description
rs73197324				
rs5925972	X	KLHL15	HGNC Symbol	kelch-like family member 15 [Source:HGNC Symbol;Acc:29347]
rs73197327	X	KLHL15	HGNC Symbol	kelch-like family member 15 [Source:HGNC Symbol;Acc:29347]
rs73197333	X	KLHL15	HGNC Symbol	kelch-like family member 15 [Source:HGNC Symbol;Acc:29347]
rs6418536	X	KLHL15	HGNC Symbol	kelch-like family member 15 [Source:HGNC Symbol;Acc:29347]
rs5970824	X	KLHL15	HGNC Symbol	kelch-like family member 15 [Source:HGNC Symbol;Acc:29347]
rs16997659	X	EIF2S3	HGNC Symbol	eukaryotic translation initiation factor 2, subunit 3 gamma, 52kDa [Source:HGNC Symbol;Acc:3267]
rs16997670	X	EIF2S3	HGNC Symbol	eukaryotic translation initiation factor 2, subunit 3 gamma, 52kDa [Source:HGNC Symbol;Acc:3267]
rs6627997				
rs11094950				
rs7880337				
rs4969545				
rs192547225				
rs2464544	X	ZFX	HGNC Symbol	zinc finger protein, X-linked [Source:HGNC Symbol;Acc:12869]
rs2704824	X	ZFX	HGNC Symbol	zinc finger protein, X-linked [Source:HGNC Symbol;Acc:12869]
rs2073961				
rs79590384				
rs111664598				
rs4285645				
rs7059478				
rs66666633				
rs1880977				
rs5990048				
rs148651598				
rs73203501				
rs141165207				
rs12010679				
rs5990056				
rs5990061				
rs111323038				
rs113045766				
rs72620481				
rs35992958				

SNP	Chromosome	gene name	gene source	description
rs148901532				
rs2107421				
rs11798684				
rs17217563				
rs7056056				
rs12013712	X	PDK3	HGNC Symbol	pyruvate dehydrogenase kinase, isozyme 3 [Source:HGNC Symbol;Acc:8811]
rs5944136	X	PDK3	HGNC Symbol	pyruvate dehydrogenase kinase, isozyme 3 [Source:HGNC Symbol;Acc:8811]
rs2074614	X	PDK3	HGNC Symbol	pyruvate dehydrogenase kinase, isozyme 3 [Source:HGNC Symbol;Acc:8811]
rs58988588	X	PDK3	HGNC Symbol	pyruvate dehydrogenase kinase, isozyme 3 [Source:HGNC Symbol;Acc:8811]
rs111951428	X	PDK3	HGNC Symbol	pyruvate dehydrogenase kinase, isozyme 3 [Source:HGNC Symbol;Acc:8811]
rs16983110	X	PDK3	HGNC Symbol	pyruvate dehydrogenase kinase, isozyme 3 [Source:HGNC Symbol;Acc:8811]
rs1210902				
rs1055186	X	GS1-358P8.4	Clone-based (Vega) gene	
rs146194002				
rs61756161	X	PCYT1B	HGNC Symbol	phosphate cytidylyltransferase 1, choline, beta [Source:HGNC Symbol;Acc:8755]
rs3761612	X	PCYT1B	HGNC Symbol	phosphate cytidylyltransferase 1, choline, beta [Source:HGNC Symbol;Acc:8755]
rs3761611	X	PCYT1B	HGNC Symbol	phosphate cytidylyltransferase 1, choline, beta [Source:HGNC Symbol;Acc:8755]
rs10156959	X	PCYT1B	HGNC Symbol	phosphate cytidylyltransferase 1, choline, beta [Source:HGNC Symbol;Acc:8755]
rs41303161	X	PCYT1B	HGNC Symbol	phosphate cytidylyltransferase 1, choline, beta [Source:HGNC Symbol;Acc:8755]
rs1882402	X	PCYT1B	HGNC Symbol	phosphate cytidylyltransferase 1, choline, beta [Source:HGNC Symbol;Acc:8755]
rs12689847	X	PCYT1B	HGNC Symbol	phosphate cytidylyltransferase 1, choline, beta [Source:HGNC Symbol;Acc:8755]
rs6629883	X	PCYT1B	HGNC Symbol	phosphate cytidylyltransferase 1, choline, beta [Source:HGNC Symbol;Acc:8755]
rs5944622	X	PCYT1B	HGNC Symbol	phosphate cytidylyltransferase 1, choline, beta [Source:HGNC Symbol;Acc:8755]
rs73207233	X	PCYT1B	HGNC Symbol	phosphate cytidylyltransferase 1, choline, beta [Source:HGNC Symbol;Acc:8755]
rs1921917	X	PCYT1B	HGNC Symbol	phosphate cytidylyltransferase 1, choline, beta [Source:HGNC Symbol;Acc:8755]
rs61760929	X	PCYT1B	HGNC Symbol	phosphate cytidylyltransferase 1, choline, beta [Source:HGNC Symbol;Acc:8755]
rs61760928	X	PCYT1B	HGNC Symbol	phosphate cytidylyltransferase 1, choline, beta [Source:HGNC Symbol;Acc:8755]
rs111855794	X	PCYT1B	HGNC Symbol	phosphate cytidylyltransferase 1, choline, beta [Source:HGNC Symbol;Acc:8755]
rs73207242				
rs5944670				
rs11573307	X	POLA1	HGNC Symbol	polymerase (DNA directed), alpha 1, catalytic subunit [Source:HGNC Symbol;Acc:9173]
rs11573331	X	POLA1	HGNC Symbol	polymerase (DNA directed), alpha 1, catalytic subunit [Source:HGNC Symbol;Acc:9173]

SNP	Chromosome	gene name	gene source	description
rs11573347	X	POLA1	HGNC Symbol	polymerase (DNA directed), alpha 1, catalytic subunit [Source:HGNC Symbol;Acc:9173]
rs4369141	X	POLA1	HGNC Symbol	polymerase (DNA directed), alpha 1, catalytic subunit [Source:HGNC Symbol;Acc:9173]
rs11573406	X	POLA1	HGNC Symbol	polymerase (DNA directed), alpha 1, catalytic subunit [Source:HGNC Symbol;Acc:9173]
rs73207264	X	POLA1	HGNC Symbol	polymerase (DNA directed), alpha 1, catalytic subunit [Source:HGNC Symbol;Acc:9173]
rs41548013	X	POLA1	HGNC Symbol	polymerase (DNA directed), alpha 1, catalytic subunit [Source:HGNC Symbol;Acc:9173]
rs11573423	X	POLA1	HGNC Symbol	polymerase (DNA directed), alpha 1, catalytic subunit [Source:HGNC Symbol;Acc:9173]
rs5944693	X	POLA1	HGNC Symbol	polymerase (DNA directed), alpha 1, catalytic subunit [Source:HGNC Symbol;Acc:9173]
rs58097728	X	POLA1	HGNC Symbol	polymerase (DNA directed), alpha 1, catalytic subunit [Source:HGNC Symbol;Acc:9173]
rs5986715	X	POLA1	HGNC Symbol	polymerase (DNA directed), alpha 1, catalytic subunit [Source:HGNC Symbol;Acc:9173]
rs73207280	X	POLA1	HGNC Symbol	polymerase (DNA directed), alpha 1, catalytic subunit [Source:HGNC Symbol;Acc:9173]
rs5944708	X	POLA1	HGNC Symbol	polymerase (DNA directed), alpha 1, catalytic subunit [Source:HGNC Symbol;Acc:9173]
rs12861811	X	POLA1	HGNC Symbol	polymerase (DNA directed), alpha 1, catalytic subunit [Source:HGNC Symbol;Acc:9173]
rs11573525	X	POLA1	HGNC Symbol	polymerase (DNA directed), alpha 1, catalytic subunit [Source:HGNC Symbol;Acc:9173]
rs5943999				
rs2285563	X	ARX	HGNC Symbol	aristaless related homeobox [Source:HGNC Symbol;Acc:18060]
rs7059234				
rs6629939				
rs4898302				
rs17332785				
rs5986729				
rs5944005				
rs112566028				
rs4634827				
rs145112675				
rs6629950				
rs16983315				
rs4243534				
rs113659430				
rs6629957				
rs148210435				
rs10126819				
rs5986744				
rs10521934				

SNP	Chromosome	gene name	gene source	description
rs73209003				
rs5944024				
rs5944025				
rs5986370				
rs6629997				
rs73209020				
rs4898209				
rs5986786				
rs4456033				
rs73209032				
rs141650076				
rs73209048				
rs4131175				
rs142866498				
rs113198856				
rs5944074				
rs4633216				
rs5944085				
rs146408323				
rs12390663				
rs144911117				
rs7055077				
rs7890656				
rs151043588				
rs5986250				
rs5986439				
rs11798012				
rs4460561				
rs6630087				
rs34692388				
rs12014610				
rs6630104				
rs5944131				

SNP	Chromosome	gene name	gene source	description
rs6628119				
rs16983559				
rs58859947				
rs5944138				
rs12688703				
rs73195041				
rs7880679				
rs1444718				
rs5944150				
rs6630136				
rs959644				
rs73195057				
rs149021667				
rs5944185				
rs5986493				
rs73491663	X	RP11-86A5.1	Clone-based (Vega) gene	
rs2340471				
rs6630211				
rs1487638				
rs113971937				
rs62586841				
rs5986536				
rs5944236				
rs148149041				
rs67884998				
rs73200521				
rs4489480				
rs12840159				
rs115186268				
rs73200529				
rs67066022				
rs5986560				

SNP	Chromosome	gene name	gene source	description
rs12687007				
rs137963769				
rs4129450				
rs12861777				
rs140120359				
rs5986310				
rs72623044				
rs5943844	X	MAGEB18	HGNC Symbol	melanoma antigen family B, 18 [Source:HGNC Symbol;Acc:28515]
rs5943845	X	MAGEB18	HGNC Symbol	melanoma antigen family B, 18 [Source:HGNC Symbol;Acc:28515]
rs5944317	X	MAGEB18	HGNC Symbol	melanoma antigen family B, 18 [Source:HGNC Symbol;Acc:28515]
rs5944318	X	MAGEB18	HGNC Symbol	melanoma antigen family B, 18 [Source:HGNC Symbol;Acc:28515]
rs73202609	X	MAGEB18	HGNC Symbol	melanoma antigen family B, 18 [Source:HGNC Symbol;Acc:28515]
rs148361973				
rs11795624	X	MAGEB6	HGNC Symbol	melanoma antigen family B, 6 [Source:HGNC Symbol;Acc:23796]
rs147228278	X	MAGEB6	HGNC Symbol	melanoma antigen family B, 6 [Source:HGNC Symbol;Acc:23796]
rs5943864				
rs5986592				
rs5943870				
rs3788955				
rs73202643				
rs1548657				
rs150153705				
rs5986610				
rs5944455				
rs11797603				
rs111330176				
rs5986622				
rs12845487				
rs4133972				
rs733019				
rs5943949				
rs141815299				
rs12007793				

SNP	Chromosome	gene name	gene source	description
rs11796625				
rs4567203				
rs5944580				
rs112056447				
rs5944587				
rs12851310				
rs56026808				
rs5986671				
rs73206325				
rs56229117				
rs11798748				
rs141562360				
rs5971231				
rs148342578				
rs1479413				
rs73204844				
rs6630312				
rs12861496				
rs5926526				
rs7056541				
rs6630338				
rs73204855				
rs6630347	X	RP11-268G12.3	Clone-based (Vega) gene	
rs5971335	X	RP11-268G12.3	Clone-based (Vega) gene	
rs12842910	X	RP11-268G12.3	Clone-based (Vega) gene	
rs114793271	X	RP11-268G12.3	Clone-based (Vega) gene	
rs1898742	X	RP11-268G12.3	Clone-based (Vega) gene	
rs6630351	X	RP11-268G12.3	Clone-based (Vega) gene	

SNP	Chromosome	gene name	gene source	description
rs11095080	X	RP11-268G12.3	Clone-based (Vega) gene	
rs5926592	X	RP11-268G12.3	Clone-based (Vega) gene	
rs62586691	X	RP11-268G12.3	Clone-based (Vega) gene	
rs5926363	X	RP11-268G12.3	Clone-based (Vega) gene	
rs73204867	X	RP11-268G12.3	Clone-based (Vega) gene	
rs73204870	X	RP11-268G12.3	Clone-based (Vega) gene	
rs66898946	X	RP11-268G12.3	Clone-based (Vega) gene	
rs5926611	X	RP11-268G12.3	Clone-based (Vega) gene	
rs1972978	X	RP11-268G12.3	Clone-based (Vega) gene	
rs78739626	X	RP11-268G12.1	Clone-based (Vega) gene	
rs2197785	X	RP11-268G12.1	Clone-based (Vega) gene	
rs7055337	X	RP11-268G12.1	Clone-based (Vega) gene	
rs73204361	X	RP11-268G12.1	Clone-based (Vega) gene	
rs4451464	X	RP11-268G12.1	Clone-based (Vega) gene	
rs73204398	X	RP11-268G12.1	Clone-based (Vega) gene	
rs73206631	X	RP11-268G12.1	Clone-based (Vega) gene	
rs12847621	X	RP11-268G12.1	Clone-based (Vega) gene	
rs151154140	X	RP11-268G12.1	Clone-based (Vega) gene	
rs73206641	X	RP11-268G12.1	Clone-based (Vega) gene	
rs56123920	X	RP11-268G12.1	Clone-based (Vega) gene	

SNP	Chromosome	gene name	gene source	description
rs5926760				
rs5926763				
rs61029990				
rs5926780				
rs55780661				
rs55834609				
rs4534271				
rs6526672				
rs5926450				
rs139759135				
rs5926811				
rs221398				
rs221387				
rs221385				
rs5926847	X	DCAF8L2	HGNC Symbol	DDB1 and CUL4 associated factor 8-like 2 [Source:HGNC Symbol;Acc:31811]
rs964481	X	DCAF8L2	HGNC Symbol	DDB1 and CUL4 associated factor 8-like 2 [Source:HGNC Symbol;Acc:31811]
rs111500985	X	DCAF8L2	HGNC Symbol	DDB1 and CUL4 associated factor 8-like 2 [Source:HGNC Symbol;Acc:31811]
rs6653776	X	DCAF8L2	HGNC Symbol	DDB1 and CUL4 associated factor 8-like 2 [Source:HGNC Symbol;Acc:31811]
rs5971429	X	DCAF8L2	HGNC Symbol	DDB1 and CUL4 associated factor 8-like 2 [Source:HGNC Symbol;Acc:31811]
rs147504230	X	DCAF8L2	HGNC Symbol	DDB1 and CUL4 associated factor 8-like 2 [Source:HGNC Symbol;Acc:31811]
rs3905591	X	DCAF8L2	HGNC Symbol	DDB1 and CUL4 associated factor 8-like 2 [Source:HGNC Symbol;Acc:31811]
rs5926875	X	DCAF8L2	HGNC Symbol	DDB1 and CUL4 associated factor 8-like 2 [Source:HGNC Symbol;Acc:31811]
rs5926885	X	DCAF8L2	HGNC Symbol	DDB1 and CUL4 associated factor 8-like 2 [Source:HGNC Symbol;Acc:31811]
rs5926888	X	DCAF8L2	HGNC Symbol	DDB1 and CUL4 associated factor 8-like 2 [Source:HGNC Symbol;Acc:31811]
rs5926892	X	DCAF8L2	HGNC Symbol	DDB1 and CUL4 associated factor 8-like 2 [Source:HGNC Symbol;Acc:31811]
rs5926895	X	DCAF8L2;AC107613.1	HGNC Symbol;Clone-based (Ensembl) gene	DDB1 and CUL4 associated factor 8-like 2 [Source:HGNC Symbol;Acc:31811];
rs45553337	X	DCAF8L2;AC107613.1	HGNC Symbol;Clone-based (Ensembl) gene	DDB1 and CUL4 associated factor 8-like 2 [Source:HGNC Symbol;Acc:31811];
rs148939759				
rs143892005				

SNP	Chromosome	gene name	gene source	description
rs7891169				
rs146886729				
rs55941150				
rs5926501				
rs1368769	X	MAGEB10	HGNC Symbol	melanoma antigen family B, 10 [Source:HGNC Symbol;Acc:25377]
rs12557898	X	MAGEB10	HGNC Symbol	melanoma antigen family B, 10 [Source:HGNC Symbol;Acc:25377]
rs5926503				
rs5926917				
rs7888623				
rs111692933				
rs7056058				
rs10081808				
rs146365314				
rs139706051				
rs73212262				
rs34563960				
rs138723784				
rs34842460				
rs2057784				
rs73546358				
rs73546362				
rs5971266				
rs72623794				
rs181247891				
rs17286806				
rs5926549				
rs1493688				
rs5971305				
rs140419654				
rs1234509				
rs7064558				
rs7882528				
rs3848956				

SNP	Chromosome	gene name	gene source	description
rs7058779				
rs57690183				
rs57422165				
rs3850161				
rs6628345				
rs5985905				
rs73203950				
rs73203957				
rs7065687				
rs7054736				
rs5985832				
rs12012324				
rs56014958				
rs4893571				
rs5943518				
rs5985868				
rs727381				
rs4893551				
rs7876256				
rs16988261				
rs11797044	X	IL1RAPL1	HGNC Symbol	interleukin 1 receptor accessory protein-like 1 [Source:HGNC Symbol;Acc:5996]
rs73630061	X	IL1RAPL1	HGNC Symbol	interleukin 1 receptor accessory protein-like 1 [Source:HGNC Symbol;Acc:5996]
rs196998	X	IL1RAPL1	HGNC Symbol	interleukin 1 receptor accessory protein-like 1 [Source:HGNC Symbol;Acc:5996]
rs196999	X	IL1RAPL1	HGNC Symbol	interleukin 1 receptor accessory protein-like 1 [Source:HGNC Symbol;Acc:5996]
rs197020	X	IL1RAPL1	HGNC Symbol	interleukin 1 receptor accessory protein-like 1 [Source:HGNC Symbol;Acc:5996]
rs197021	X	IL1RAPL1	HGNC Symbol	interleukin 1 receptor accessory protein-like 1 [Source:HGNC Symbol;Acc:5996]
rs404274	X	IL1RAPL1	HGNC Symbol	interleukin 1 receptor accessory protein-like 1 [Source:HGNC Symbol;Acc:5996]
rs196973	X	IL1RAPL1	HGNC Symbol	interleukin 1 receptor accessory protein-like 1 [Source:HGNC Symbol;Acc:5996]
rs73205779	X	IL1RAPL1	HGNC Symbol	interleukin 1 receptor accessory protein-like 1 [Source:HGNC Symbol;Acc:5996]
rs12387953	X	IL1RAPL1	HGNC Symbol	interleukin 1 receptor accessory protein-like 1 [Source:HGNC Symbol;Acc:5996]
rs10521946	X	IL1RAPL1	HGNC Symbol	interleukin 1 receptor accessory protein-like 1 [Source:HGNC Symbol;Acc:5996]
rs4483325	X	IL1RAPL1	HGNC Symbol	interleukin 1 receptor accessory protein-like 1 [Source:HGNC Symbol;Acc:5996]
rs150225835	X	IL1RAPL1	HGNC Symbol	interleukin 1 receptor accessory protein-like 1 [Source:HGNC Symbol;Acc:5996]

SNP	Chromosome	gene name	gene source	description
rs5985802	X	IL1RAPL1	HGNC Symbol	interleukin 1 receptor accessory protein-like 1 [Source:HGNC Symbol;Acc:5996]
rs1384575	X	IL1RAPL1	HGNC Symbol	interleukin 1 receptor accessory protein-like 1 [Source:HGNC Symbol;Acc:5996]
rs1482952	X	IL1RAPL1	HGNC Symbol	interleukin 1 receptor accessory protein-like 1 [Source:HGNC Symbol;Acc:5996]
rs151063260	X	IL1RAPL1	HGNC Symbol	interleukin 1 receptor accessory protein-like 1 [Source:HGNC Symbol;Acc:5996]
rs590796	X	IL1RAPL1	HGNC Symbol	interleukin 1 receptor accessory protein-like 1 [Source:HGNC Symbol;Acc:5996]
rs12860943	X	IL1RAPL1	HGNC Symbol	interleukin 1 receptor accessory protein-like 1 [Source:HGNC Symbol;Acc:5996]
rs62587526	X	IL1RAPL1	HGNC Symbol	interleukin 1 receptor accessory protein-like 1 [Source:HGNC Symbol;Acc:5996]
rs73208062	X	IL1RAPL1	HGNC Symbol	interleukin 1 receptor accessory protein-like 1 [Source:HGNC Symbol;Acc:5996]
rs1680960	X	IL1RAPL1	HGNC Symbol	interleukin 1 receptor accessory protein-like 1 [Source:HGNC Symbol;Acc:5996]
rs66759111	X	IL1RAPL1	HGNC Symbol	interleukin 1 receptor accessory protein-like 1 [Source:HGNC Symbol;Acc:5996]
rs5985930	X	IL1RAPL1	HGNC Symbol	interleukin 1 receptor accessory protein-like 1 [Source:HGNC Symbol;Acc:5996]
rs6630792	X	IL1RAPL1	HGNC Symbol	interleukin 1 receptor accessory protein-like 1 [Source:HGNC Symbol;Acc:5996]
rs56960976	X	IL1RAPL1	HGNC Symbol	interleukin 1 receptor accessory protein-like 1 [Source:HGNC Symbol;Acc:5996]
rs641288	X	IL1RAPL1	HGNC Symbol	interleukin 1 receptor accessory protein-like 1 [Source:HGNC Symbol;Acc:5996]
rs5985934	X	IL1RAPL1	HGNC Symbol	interleukin 1 receptor accessory protein-like 1 [Source:HGNC Symbol;Acc:5996]
rs5943581	X	IL1RAPL1	HGNC Symbol	interleukin 1 receptor accessory protein-like 1 [Source:HGNC Symbol;Acc:5996]
rs66464794	X	IL1RAPL1	HGNC Symbol	interleukin 1 receptor accessory protein-like 1 [Source:HGNC Symbol;Acc:5996]
rs5943585	X	IL1RAPL1	HGNC Symbol	interleukin 1 receptor accessory protein-like 1 [Source:HGNC Symbol;Acc:5996]
rs73208096	X	IL1RAPL1	HGNC Symbol	interleukin 1 receptor accessory protein-like 1 [Source:HGNC Symbol;Acc:5996]
rs58474954	X	IL1RAPL1	HGNC Symbol	interleukin 1 receptor accessory protein-like 1 [Source:HGNC Symbol;Acc:5996]
rs73210004	X	IL1RAPL1	HGNC Symbol	interleukin 1 receptor accessory protein-like 1 [Source:HGNC Symbol;Acc:5996]
rs5943473	X	IL1RAPL1	HGNC Symbol	interleukin 1 receptor accessory protein-like 1 [Source:HGNC Symbol;Acc:5996]
rs36033238	X	IL1RAPL1	HGNC Symbol	interleukin 1 receptor accessory protein-like 1 [Source:HGNC Symbol;Acc:5996]
rs67762551	X	IL1RAPL1	HGNC Symbol	interleukin 1 receptor accessory protein-like 1 [Source:HGNC Symbol;Acc:5996]
rs1500727	X	IL1RAPL1	HGNC Symbol	interleukin 1 receptor accessory protein-like 1 [Source:HGNC Symbol;Acc:5996]
rs6526832	X	IL1RAPL1	HGNC Symbol	interleukin 1 receptor accessory protein-like 1 [Source:HGNC Symbol;Acc:5996]
rs9887423	X	IL1RAPL1	HGNC Symbol	interleukin 1 receptor accessory protein-like 1 [Source:HGNC Symbol;Acc:5996]
rs12557212	X	IL1RAPL1	HGNC Symbol	interleukin 1 receptor accessory protein-like 1 [Source:HGNC Symbol;Acc:5996]
rs73210036	X	IL1RAPL1	HGNC Symbol	interleukin 1 receptor accessory protein-like 1 [Source:HGNC Symbol;Acc:5996]
rs73210051	X	IL1RAPL1	HGNC Symbol	interleukin 1 receptor accessory protein-like 1 [Source:HGNC Symbol;Acc:5996]
rs73210056	X	IL1RAPL1	HGNC Symbol	interleukin 1 receptor accessory protein-like 1 [Source:HGNC Symbol;Acc:5996]
rs73210063	X	IL1RAPL1	HGNC Symbol	interleukin 1 receptor accessory protein-like 1 [Source:HGNC Symbol;Acc:5996]
rs225050	X	IL1RAPL1	HGNC Symbol	interleukin 1 receptor accessory protein-like 1 [Source:HGNC Symbol;Acc:5996]

SNP	Chromosome	gene name	gene source	description
rs60367555	X	IL1RAPL1	HGNC Symbol	interleukin 1 receptor accessory protein-like 1 [Source:HGNC Symbol;Acc:5996]
rs4893607	X	IL1RAPL1	HGNC Symbol	interleukin 1 receptor accessory protein-like 1 [Source:HGNC Symbol;Acc:5996]
rs225461	X	IL1RAPL1	HGNC Symbol	interleukin 1 receptor accessory protein-like 1 [Source:HGNC Symbol;Acc:5996]
rs17282570	X	IL1RAPL1	HGNC Symbol	interleukin 1 receptor accessory protein-like 1 [Source:HGNC Symbol;Acc:5996]
rs5943606	X	IL1RAPL1	HGNC Symbol	interleukin 1 receptor accessory protein-like 1 [Source:HGNC Symbol;Acc:5996]
rs62586207	X	IL1RAPL1	HGNC Symbol	interleukin 1 receptor accessory protein-like 1 [Source:HGNC Symbol;Acc:5996]
rs17282584	X	IL1RAPL1	HGNC Symbol	interleukin 1 receptor accessory protein-like 1 [Source:HGNC Symbol;Acc:5996]
rs113428419	X	IL1RAPL1	HGNC Symbol	interleukin 1 receptor accessory protein-like 1 [Source:HGNC Symbol;Acc:5996]
rs138124070	X	IL1RAPL1	HGNC Symbol	interleukin 1 receptor accessory protein-like 1 [Source:HGNC Symbol;Acc:5996]
rs5943613	X	IL1RAPL1	HGNC Symbol	interleukin 1 receptor accessory protein-like 1 [Source:HGNC Symbol;Acc:5996]
rs2223505	X	IL1RAPL1	HGNC Symbol	interleukin 1 receptor accessory protein-like 1 [Source:HGNC Symbol;Acc:5996]
rs2206353	X	IL1RAPL1	HGNC Symbol	interleukin 1 receptor accessory protein-like 1 [Source:HGNC Symbol;Acc:5996]
rs7055426	X	IL1RAPL1	HGNC Symbol	interleukin 1 receptor accessory protein-like 1 [Source:HGNC Symbol;Acc:5996]
rs5943626	X	IL1RAPL1	HGNC Symbol	interleukin 1 receptor accessory protein-like 1 [Source:HGNC Symbol;Acc:5996]
rs12558805	X	IL1RAPL1	HGNC Symbol	interleukin 1 receptor accessory protein-like 1 [Source:HGNC Symbol;Acc:5996]
rs62586250	X	IL1RAPL1	HGNC Symbol	interleukin 1 receptor accessory protein-like 1 [Source:HGNC Symbol;Acc:5996]
rs5943630	X	IL1RAPL1	HGNC Symbol	interleukin 1 receptor accessory protein-like 1 [Source:HGNC Symbol;Acc:5996]
rs73212147	X	IL1RAPL1	HGNC Symbol	interleukin 1 receptor accessory protein-like 1 [Source:HGNC Symbol;Acc:5996]
rs138371594	X	IL1RAPL1	HGNC Symbol	interleukin 1 receptor accessory protein-like 1 [Source:HGNC Symbol;Acc:5996]
rs62588086	X	IL1RAPL1	HGNC Symbol	interleukin 1 receptor accessory protein-like 1 [Source:HGNC Symbol;Acc:5996]
rs73212153	X	IL1RAPL1	HGNC Symbol	interleukin 1 receptor accessory protein-like 1 [Source:HGNC Symbol;Acc:5996]
rs4893560	X	IL1RAPL1	HGNC Symbol	interleukin 1 receptor accessory protein-like 1 [Source:HGNC Symbol;Acc:5996]
rs7050714	X	IL1RAPL1	HGNC Symbol	interleukin 1 receptor accessory protein-like 1 [Source:HGNC Symbol;Acc:5996]
rs144937713	X	IL1RAPL1	HGNC Symbol	interleukin 1 receptor accessory protein-like 1 [Source:HGNC Symbol;Acc:5996]
rs11797880	X	IL1RAPL1	HGNC Symbol	interleukin 1 receptor accessory protein-like 1 [Source:HGNC Symbol;Acc:5996]
rs55797583	X	IL1RAPL1	HGNC Symbol	interleukin 1 receptor accessory protein-like 1 [Source:HGNC Symbol;Acc:5996]
rs16988507	X	IL1RAPL1	HGNC Symbol	interleukin 1 receptor accessory protein-like 1 [Source:HGNC Symbol;Acc:5996]
rs17282598	X	IL1RAPL1	HGNC Symbol	interleukin 1 receptor accessory protein-like 1 [Source:HGNC Symbol;Acc:5996]
rs5927671	X	IL1RAPL1	HGNC Symbol	interleukin 1 receptor accessory protein-like 1 [Source:HGNC Symbol;Acc:5996]
rs7061580	X	IL1RAPL1	HGNC Symbol	interleukin 1 receptor accessory protein-like 1 [Source:HGNC Symbol;Acc:5996]
rs5927223	X	IL1RAPL1	HGNC Symbol	interleukin 1 receptor accessory protein-like 1 [Source:HGNC Symbol;Acc:5996]
rs55767707	X	IL1RAPL1	HGNC Symbol	interleukin 1 receptor accessory protein-like 1 [Source:HGNC Symbol;Acc:5996]
rs28597906	X	IL1RAPL1	HGNC Symbol	interleukin 1 receptor accessory protein-like 1 [Source:HGNC Symbol;Acc:5996]

SNP	Chromosome	gene name	gene source	description
rs6526854	X	IL1RAPL1	HGNC Symbol	interleukin 1 receptor accessory protein-like 1 [Source:HGNC Symbol;Acc:5996]
rs5972234	X	IL1RAPL1	HGNC Symbol	interleukin 1 receptor accessory protein-like 1 [Source:HGNC Symbol;Acc:5996]
rs59870995	X	IL1RAPL1	HGNC Symbol	interleukin 1 receptor accessory protein-like 1 [Source:HGNC Symbol;Acc:5996]
rs17282704	X	IL1RAPL1	HGNC Symbol	interleukin 1 receptor accessory protein-like 1 [Source:HGNC Symbol;Acc:5996]
rs6630882	X	IL1RAPL1	HGNC Symbol	interleukin 1 receptor accessory protein-like 1 [Source:HGNC Symbol;Acc:5996]
rs12556817	X	IL1RAPL1	HGNC Symbol	interleukin 1 receptor accessory protein-like 1 [Source:HGNC Symbol;Acc:5996]
rs5927687	X	IL1RAPL1	HGNC Symbol	interleukin 1 receptor accessory protein-like 1 [Source:HGNC Symbol;Acc:5996]
rs16988634	X	IL1RAPL1	HGNC Symbol	interleukin 1 receptor accessory protein-like 1 [Source:HGNC Symbol;Acc:5996]
rs5971569	X	IL1RAPL1	HGNC Symbol	interleukin 1 receptor accessory protein-like 1 [Source:HGNC Symbol;Acc:5996]
rs7058208	X	IL1RAPL1	HGNC Symbol	interleukin 1 receptor accessory protein-like 1 [Source:HGNC Symbol;Acc:5996]
rs12007306	X	IL1RAPL1	HGNC Symbol	interleukin 1 receptor accessory protein-like 1 [Source:HGNC Symbol;Acc:5996]
rs1022525	X	IL1RAPL1	HGNC Symbol	interleukin 1 receptor accessory protein-like 1 [Source:HGNC Symbol;Acc:5996]
rs12849965	X	IL1RAPL1	HGNC Symbol	interleukin 1 receptor accessory protein-like 1 [Source:HGNC Symbol;Acc:5996]
rs12014473	X	IL1RAPL1	HGNC Symbol	interleukin 1 receptor accessory protein-like 1 [Source:HGNC Symbol;Acc:5996]
rs6526880	X	IL1RAPL1	HGNC Symbol	interleukin 1 receptor accessory protein-like 1 [Source:HGNC Symbol;Acc:5996]
rs3813179	X	IL1RAPL1	HGNC Symbol	interleukin 1 receptor accessory protein-like 1 [Source:HGNC Symbol;Acc:5996]
rs147032170	X	IL1RAPL1	HGNC Symbol	interleukin 1 receptor accessory protein-like 1 [Source:HGNC Symbol;Acc:5996]
rs6628454	X	IL1RAPL1	HGNC Symbol	interleukin 1 receptor accessory protein-like 1 [Source:HGNC Symbol;Acc:5996]
rs59968462	X	IL1RAPL1	HGNC Symbol	interleukin 1 receptor accessory protein-like 1 [Source:HGNC Symbol;Acc:5996]
rs5927844	X	IL1RAPL1	HGNC Symbol	interleukin 1 receptor accessory protein-like 1 [Source:HGNC Symbol;Acc:5996]
rs139798823	X	IL1RAPL1	HGNC Symbol	interleukin 1 receptor accessory protein-like 1 [Source:HGNC Symbol;Acc:5996]
rs9969869	X	IL1RAPL1	HGNC Symbol	interleukin 1 receptor accessory protein-like 1 [Source:HGNC Symbol;Acc:5996]
rs4829117	X	IL1RAPL1	HGNC Symbol	interleukin 1 receptor accessory protein-like 1 [Source:HGNC Symbol;Acc:5996]
rs5972489	X	IL1RAPL1	HGNC Symbol	interleukin 1 receptor accessory protein-like 1 [Source:HGNC Symbol;Acc:5996]
rs141855038	X	IL1RAPL1	HGNC Symbol	interleukin 1 receptor accessory protein-like 1 [Source:HGNC Symbol;Acc:5996]
rs147175681	X	IL1RAPL1	HGNC Symbol	interleukin 1 receptor accessory protein-like 1 [Source:HGNC Symbol;Acc:5996]
rs5971618	X	IL1RAPL1	HGNC Symbol	interleukin 1 receptor accessory protein-like 1 [Source:HGNC Symbol;Acc:5996]
rs112459152	X	IL1RAPL1	HGNC Symbol	interleukin 1 receptor accessory protein-like 1 [Source:HGNC Symbol;Acc:5996]
rs5972676	X	IL1RAPL1	HGNC Symbol	interleukin 1 receptor accessory protein-like 1 [Source:HGNC Symbol;Acc:5996]
rs73456409	X	IL1RAPL1	HGNC Symbol	interleukin 1 receptor accessory protein-like 1 [Source:HGNC Symbol;Acc:5996]
rs147599550	X	IL1RAPL1	HGNC Symbol	interleukin 1 receptor accessory protein-like 1 [Source:HGNC Symbol;Acc:5996]
rs147408803	X	IL1RAPL1	HGNC Symbol	interleukin 1 receptor accessory protein-like 1 [Source:HGNC Symbol;Acc:5996]
rs5927181	X	IL1RAPL1	HGNC Symbol	interleukin 1 receptor accessory protein-like 1 [Source:HGNC Symbol;Acc:5996]

SNP	Chromosome	gene name	gene source	description
rs6526926	X	IL1RAPL1	HGNC Symbol	interleukin 1 receptor accessory protein-like 1 [Source:HGNC Symbol;Acc:5996]
rs12835067	X	IL1RAPL1	HGNC Symbol	interleukin 1 receptor accessory protein-like 1 [Source:HGNC Symbol;Acc:5996]
rs11798343	X	IL1RAPL1	HGNC Symbol	interleukin 1 receptor accessory protein-like 1 [Source:HGNC Symbol;Acc:5996]
rs5927208	X	IL1RAPL1	HGNC Symbol	interleukin 1 receptor accessory protein-like 1 [Source:HGNC Symbol;Acc:5996]
rs5973030	X	IL1RAPL1	HGNC Symbol	interleukin 1 receptor accessory protein-like 1 [Source:HGNC Symbol;Acc:5996]
rs55959086	X	IL1RAPL1	HGNC Symbol	interleukin 1 receptor accessory protein-like 1 [Source:HGNC Symbol;Acc:5996]
rs5928423	X	IL1RAPL1	HGNC Symbol	interleukin 1 receptor accessory protein-like 1 [Source:HGNC Symbol;Acc:5996]
rs12859816	X	IL1RAPL1	HGNC Symbol	interleukin 1 receptor accessory protein-like 1 [Source:HGNC Symbol;Acc:5996]
rs73221614	X	IL1RAPL1	HGNC Symbol	interleukin 1 receptor accessory protein-like 1 [Source:HGNC Symbol;Acc:5996]
rs5973250	X	IL1RAPL1	HGNC Symbol	interleukin 1 receptor accessory protein-like 1 [Source:HGNC Symbol;Acc:5996]
rs12558491	X	IL1RAPL1	HGNC Symbol	interleukin 1 receptor accessory protein-like 1 [Source:HGNC Symbol;Acc:5996]
rs114918903	X	IL1RAPL1	HGNC Symbol	interleukin 1 receptor accessory protein-like 1 [Source:HGNC Symbol;Acc:5996]
rs12835711	X	IL1RAPL1	HGNC Symbol	interleukin 1 receptor accessory protein-like 1 [Source:HGNC Symbol;Acc:5996]
rs147730436	X	IL1RAPL1	HGNC Symbol	interleukin 1 receptor accessory protein-like 1 [Source:HGNC Symbol;Acc:5996]
rs34802668	X	IL1RAPL1	HGNC Symbol	interleukin 1 receptor accessory protein-like 1 [Source:HGNC Symbol;Acc:5996]
rs116130281	X	IL1RAPL1	HGNC Symbol	interleukin 1 receptor accessory protein-like 1 [Source:HGNC Symbol;Acc:5996]
rs1419850	X	IL1RAPL1	HGNC Symbol	interleukin 1 receptor accessory protein-like 1 [Source:HGNC Symbol;Acc:5996]
rs12839149	X	IL1RAPL1	HGNC Symbol	interleukin 1 receptor accessory protein-like 1 [Source:HGNC Symbol;Acc:5996]
rs1344448	X	IL1RAPL1	HGNC Symbol	interleukin 1 receptor accessory protein-like 1 [Source:HGNC Symbol;Acc:5996]
rs7891310	X	IL1RAPL1	HGNC Symbol	interleukin 1 receptor accessory protein-like 1 [Source:HGNC Symbol;Acc:5996]
rs5973335	X	IL1RAPL1	HGNC Symbol	interleukin 1 receptor accessory protein-like 1 [Source:HGNC Symbol;Acc:5996]
rs140795074	X	IL1RAPL1	HGNC Symbol	interleukin 1 receptor accessory protein-like 1 [Source:HGNC Symbol;Acc:5996]
rs147042835	X	IL1RAPL1	HGNC Symbol	interleukin 1 receptor accessory protein-like 1 [Source:HGNC Symbol;Acc:5996]
rs145691017	X	IL1RAPL1	HGNC Symbol	interleukin 1 receptor accessory protein-like 1 [Source:HGNC Symbol;Acc:5996]
rs145600772				
rs5973509				
rs5973570				
rs5973641				
rs73631681				
rs7473093				
rs2158908				
rs73203708				
rs28643295				

SNP	Chromosome	gene name	gene source	description
rs56392806				
rs6628506				
rs12846225				
rs4610906				
rs144057297				
rs6628511				
rs55665775				
rs5929069				
rs973212				
rs62589084				
rs12848838				
rs73205817				
rs147559118	X	MAGEB2	HGNC Symbol	melanoma antigen family B, 2 [Source:HGNC Symbol;Acc:6809]
rs2074793	X	MAGEB2	HGNC Symbol	melanoma antigen family B, 2 [Source:HGNC Symbol;Acc:6809]
rs5972091	X	MAGEB2	HGNC Symbol	melanoma antigen family B, 2 [Source:HGNC Symbol;Acc:6809]
rs1053918	X	MAGEB2	HGNC Symbol	melanoma antigen family B, 2 [Source:HGNC Symbol;Acc:6809]
rs12007870				
rs2529543				
rs12008668	X	MAGEB3	HGNC Symbol	melanoma antigen family B, 3 [Source:HGNC Symbol;Acc:6810]
rs2071308	X	MAGEB3	HGNC Symbol	melanoma antigen family B, 3 [Source:HGNC Symbol;Acc:6810]
rs2071309	X	MAGEB3	HGNC Symbol	melanoma antigen family B, 3 [Source:HGNC Symbol;Acc:6810]
rs2071310	X	MAGEB3	HGNC Symbol	melanoma antigen family B, 3 [Source:HGNC Symbol;Acc:6810]
rs2071311	X	MAGEB4	HGNC Symbol	melanoma antigen family B, 4 [Source:HGNC Symbol;Acc:6811]
rs2856733	X	MAGEB4	HGNC Symbol	melanoma antigen family B, 4 [Source:HGNC Symbol;Acc:6811]
rs2864919				
rs56850500				
rs12837639				
rs10429737				
rs4141067				
rs5929084				
rs7062500				
rs5973812				
rs2864927				

SNP	Chromosome	gene name	gene source	description
rs12846323				
rs4829424				
rs6150	X	NR0B1	HGNC Symbol	nuclear receptor subfamily 0, group B, member 1 [Source:HGNC Symbol;Acc:7960]
rs4829169				
rs1034948				
rs73205867				
rs5972103				
rs9969915				
rs73205886				
rs13440584				
rs4829173				
rs6526973				
rs7059817				
rs75920607				
rs4829180				
rs62589870				
rs5972165				
rs7891921				
rs5971491				
rs73631702				
rs5972174				
rs113583786				
rs5927538				
rs73205899				
rs16989086				
rs60676446				
rs2903515				
rs12851811				
rs2532862				
rs12859457				
rs887369	X	CXorf21	HGNC Symbol	chromosome X open reading frame 21 [Source:HGNC Symbol;Acc:25667]
rs2710401	X	CXorf21	HGNC Symbol	chromosome X open reading frame 21 [Source:HGNC Symbol;Acc:25667]
rs2710405	X	CXorf21	HGNC Symbol	chromosome X open reading frame 21 [Source:HGNC Symbol;Acc:25667]

SNP	Chromosome	gene name	gene source	description
rs12014174	X	CXorf21	HGNC Symbol	chromosome X open reading frame 21 [Source:HGNC Symbol;Acc:25667]
rs5971504				
rs1074193				
rs17282991				
rs5927576				
rs5926936				
rs6631133				
rs111458988				
rs66676923				
rs12844553	X	GK	HGNC Symbol	glycerol kinase [Source:HGNC Symbol;Acc:4289]
rs138467696	X	GK	HGNC Symbol	glycerol kinase [Source:HGNC Symbol;Acc:4289]
rs56127344	X	GK	HGNC Symbol	glycerol kinase [Source:HGNC Symbol;Acc:4289]
rs55895067	X	GK	HGNC Symbol	glycerol kinase [Source:HGNC Symbol;Acc:4289]
rs5927589	X	GK;RP11-242C19.2;GK-AS1	HGNC Symbol;Clone-based (Vega) gene	glycerol kinase [Source:HGNC Symbol;Acc:4289];;GK antisense RNA 1 [Source:HGNC Symbol;Acc:40255]
rs5927599				
rs17329748				
rs1077494				
rs5926956				
rs5927624				
rs28524893	X	TAB3	HGNC Symbol	TGF-beta activated kinase 1/MAP3K7 binding protein 3 [Source:HGNC Symbol;Acc:30681]
rs5972257	X	TAB3	HGNC Symbol	TGF-beta activated kinase 1/MAP3K7 binding protein 3 [Source:HGNC Symbol;Acc:30681]
rs5972260	X	TAB3	HGNC Symbol	TGF-beta activated kinase 1/MAP3K7 binding protein 3 [Source:HGNC Symbol;Acc:30681]
rs12836603	X	TAB3	HGNC Symbol	TGF-beta activated kinase 1/MAP3K7 binding protein 3 [Source:HGNC Symbol;Acc:30681]
rs6631208	X	TAB3	HGNC Symbol	TGF-beta activated kinase 1/MAP3K7 binding protein 3 [Source:HGNC Symbol;Acc:30681]
rs6527034	X	TAB3	HGNC Symbol	TGF-beta activated kinase 1/MAP3K7 binding protein 3 [Source:HGNC Symbol;Acc:30681]
rs10126924	X	TAB3	HGNC Symbol	TGF-beta activated kinase 1/MAP3K7 binding protein 3 [Source:HGNC Symbol;Acc:30681]
rs5972265	X	TAB3	HGNC Symbol	TGF-beta activated kinase 1/MAP3K7 binding protein 3 [Source:HGNC Symbol;Acc:30681]
rs12860337	X	TAB3	HGNC Symbol	TGF-beta activated kinase 1/MAP3K7 binding protein 3 [Source:HGNC Symbol;Acc:30681]
rs4333770	X	TAB3	HGNC Symbol	TGF-beta activated kinase 1/MAP3K7 binding protein 3 [Source:HGNC Symbol;Acc:30681]
rs4129460	X	TAB3	HGNC Symbol	TGF-beta activated kinase 1/MAP3K7 binding protein 3 [Source:HGNC Symbol;Acc:30681]
rs4601493	X	TAB3	HGNC Symbol	TGF-beta activated kinase 1/MAP3K7 binding protein 3 [Source:HGNC Symbol;Acc:30681]
rs4512552	X	TAB3	HGNC Symbol	TGF-beta activated kinase 1/MAP3K7 binding protein 3 [Source:HGNC Symbol;Acc:30681]

SNP	Chromosome	gene name	gene source	description
rs73210507	X	TAB3	HGNC Symbol	TGF-beta activated kinase 1/MAP3K7 binding protein 3 [Source:HGNC Symbol;Acc:30681]
rs6631212	X	TAB3	HGNC Symbol	TGF-beta activated kinase 1/MAP3K7 binding protein 3 [Source:HGNC Symbol;Acc:30681]
rs6631216	X	TAB3	HGNC Symbol	TGF-beta activated kinase 1/MAP3K7 binding protein 3 [Source:HGNC Symbol;Acc:30681]
rs34299085	X	TAB3	HGNC Symbol	TGF-beta activated kinase 1/MAP3K7 binding protein 3 [Source:HGNC Symbol;Acc:30681]
rs5972275	X	TAB3	HGNC Symbol	TGF-beta activated kinase 1/MAP3K7 binding protein 3 [Source:HGNC Symbol;Acc:30681]
rs7067182	X	TAB3	HGNC Symbol	TGF-beta activated kinase 1/MAP3K7 binding protein 3 [Source:HGNC Symbol;Acc:30681]
rs4626781	X	TAB3	HGNC Symbol	TGF-beta activated kinase 1/MAP3K7 binding protein 3 [Source:HGNC Symbol;Acc:30681]
rs5927653	X	TAB3	HGNC Symbol	TGF-beta activated kinase 1/MAP3K7 binding protein 3 [Source:HGNC Symbol;Acc:30681]
rs2404151				
rs150831161				
rs5972289				
rs11095199				
rs113429490				
rs62588738				
rs12012680				
rs387042				
rs417987				
rs452542				
rs397533				
rs146504146				
rs808178	X	DMD	HGNC Symbol	dystrophin [Source:HGNC Symbol;Acc:2928]
rs17329805	X	DMD	HGNC Symbol	dystrophin [Source:HGNC Symbol;Acc:2928]
rs5927689	X	DMD	HGNC Symbol	dystrophin [Source:HGNC Symbol;Acc:2928]
rs2170963	X	DMD	HGNC Symbol	dystrophin [Source:HGNC Symbol;Acc:2928]
rs1484852	X	DMD	HGNC Symbol	dystrophin [Source:HGNC Symbol;Acc:2928]
rs62590083	X	DMD	HGNC Symbol	dystrophin [Source:HGNC Symbol;Acc:2928]
rs17338367	X	DMD	HGNC Symbol	dystrophin [Source:HGNC Symbol;Acc:2928]
rs72466544	X	DMD	HGNC Symbol	dystrophin [Source:HGNC Symbol;Acc:2928]
rs5927694	X	DMD	HGNC Symbol	dystrophin [Source:HGNC Symbol;Acc:2928]
rs6631256	X	DMD	HGNC Symbol	dystrophin [Source:HGNC Symbol;Acc:2928]
rs41312092	X	DMD	HGNC Symbol	dystrophin [Source:HGNC Symbol;Acc:2928]
rs73462347	X	DMD	HGNC Symbol	dystrophin [Source:HGNC Symbol;Acc:2928]
rs2178539	X	DMD	HGNC Symbol	dystrophin [Source:HGNC Symbol;Acc:2928]

SNP	Chromosome	gene name	gene source	description
rs5972331	X	DMD	HGNC Symbol	dystrophin [Source:HGNC Symbol;Acc:2928]
rs6631263	X	DMD	HGNC Symbol	dystrophin [Source:HGNC Symbol;Acc:2928]
rs1317640	X	DMD	HGNC Symbol	dystrophin [Source:HGNC Symbol;Acc:2928]
rs2293668	X	DMD	HGNC Symbol	dystrophin [Source:HGNC Symbol;Acc:2928]
rs5927706	X	DMD	HGNC Symbol	dystrophin [Source:HGNC Symbol;Acc:2928]
rs10482232	X	DMD	HGNC Symbol	dystrophin [Source:HGNC Symbol;Acc:2928]
rs73617055	X	DMD	HGNC Symbol	dystrophin [Source:HGNC Symbol;Acc:2928]
rs12389409	X	DMD	HGNC Symbol	dystrophin [Source:HGNC Symbol;Acc:2928]
rs5971552	X	DMD	HGNC Symbol	dystrophin [Source:HGNC Symbol;Acc:2928]
rs2141755	X	DMD	HGNC Symbol	dystrophin [Source:HGNC Symbol;Acc:2928]
rs5926996	X	DMD	HGNC Symbol	dystrophin [Source:HGNC Symbol;Acc:2928]
rs5927708	X	DMD	HGNC Symbol	dystrophin [Source:HGNC Symbol;Acc:2928]
rs7889559	X	DMD	HGNC Symbol	dystrophin [Source:HGNC Symbol;Acc:2928]
rs12690302	X	DMD	HGNC Symbol	dystrophin [Source:HGNC Symbol;Acc:2928]
rs5971553	X	DMD	HGNC Symbol	dystrophin [Source:HGNC Symbol;Acc:2928]
rs12855949	X	DMD	HGNC Symbol	dystrophin [Source:HGNC Symbol;Acc:2928]
rs1921381	X	DMD	HGNC Symbol	dystrophin [Source:HGNC Symbol;Acc:2928]
rs5972341	X	DMD	HGNC Symbol	dystrophin [Source:HGNC Symbol;Acc:2928]
rs77986513	X	DMD	HGNC Symbol	dystrophin [Source:HGNC Symbol;Acc:2928]
rs10127097	X	DMD	HGNC Symbol	dystrophin [Source:HGNC Symbol;Acc:2928]
rs5927000	X	DMD	HGNC Symbol	dystrophin [Source:HGNC Symbol;Acc:2928]
rs2404500	X	DMD	HGNC Symbol	dystrophin [Source:HGNC Symbol;Acc:2928]
rs7051587	X	DMD	HGNC Symbol	dystrophin [Source:HGNC Symbol;Acc:2928]
rs12840452	X	DMD	HGNC Symbol	dystrophin [Source:HGNC Symbol;Acc:2928]
rs5927001	X	DMD	HGNC Symbol	dystrophin [Source:HGNC Symbol;Acc:2928]
rs12013572	X	DMD	HGNC Symbol	dystrophin [Source:HGNC Symbol;Acc:2928]
rs1921396	X	DMD	HGNC Symbol	dystrophin [Source:HGNC Symbol;Acc:2928]
rs12843613	X	DMD	HGNC Symbol	dystrophin [Source:HGNC Symbol;Acc:2928]
rs5972346	X	DMD	HGNC Symbol	dystrophin [Source:HGNC Symbol;Acc:2928]
rs56157001	X	DMD	HGNC Symbol	dystrophin [Source:HGNC Symbol;Acc:2928]
rs28494139	X	DMD	HGNC Symbol	dystrophin [Source:HGNC Symbol;Acc:2928]
rs1573952	X	DMD	HGNC Symbol	dystrophin [Source:HGNC Symbol;Acc:2928]
rs17340630	X	DMD	HGNC Symbol	dystrophin [Source:HGNC Symbol;Acc:2928]

SNP	Chromosome	gene name	gene source	description
rs17338423	X	DMD	HGNC Symbol	dystrophin [Source:HGNC Symbol;Acc:2928]
rs12015000	X	DMD	HGNC Symbol	dystrophin [Source:HGNC Symbol;Acc:2928]
rs151056989	X	DMD	HGNC Symbol	dystrophin [Source:HGNC Symbol;Acc:2928]
rs146998764	X	DMD	HGNC Symbol	dystrophin [Source:HGNC Symbol;Acc:2928]
rs55803048	X	DMD	HGNC Symbol	dystrophin [Source:HGNC Symbol;Acc:2928]
rs139539984	X	DMD	HGNC Symbol	dystrophin [Source:HGNC Symbol;Acc:2928]
rs5972363	X	DMD	HGNC Symbol	dystrophin [Source:HGNC Symbol;Acc:2928]
rs11095206	X	DMD	HGNC Symbol	dystrophin [Source:HGNC Symbol;Acc:2928]
rs5927716	X	DMD	HGNC Symbol	dystrophin [Source:HGNC Symbol;Acc:2928]
rs12559712	X	DMD	HGNC Symbol	dystrophin [Source:HGNC Symbol;Acc:2928]
rs1950114	X	DMD	HGNC Symbol	dystrophin [Source:HGNC Symbol;Acc:2928]
rs72625571	X	DMD	HGNC Symbol	dystrophin [Source:HGNC Symbol;Acc:2928]
rs12836134	X	DMD	HGNC Symbol	dystrophin [Source:HGNC Symbol;Acc:2928]
rs12008262	X	DMD	HGNC Symbol	dystrophin [Source:HGNC Symbol;Acc:2928]
rs138321724	X	DMD	HGNC Symbol	dystrophin [Source:HGNC Symbol;Acc:2928]
rs5927004	X	DMD	HGNC Symbol	dystrophin [Source:HGNC Symbol;Acc:2928]
rs12557091	X	DMD	HGNC Symbol	dystrophin [Source:HGNC Symbol;Acc:2928]
rs5927721	X	DMD	HGNC Symbol	dystrophin [Source:HGNC Symbol;Acc:2928]
rs12353755	X	DMD	HGNC Symbol	dystrophin [Source:HGNC Symbol;Acc:2928]
rs12844827	X	DMD	HGNC Symbol	dystrophin [Source:HGNC Symbol;Acc:2928]
rs5927726	X	DMD	HGNC Symbol	dystrophin [Source:HGNC Symbol;Acc:2928]
rs142296114	X	DMD	HGNC Symbol	dystrophin [Source:HGNC Symbol;Acc:2928]
rs5927012	X	DMD	HGNC Symbol	dystrophin [Source:HGNC Symbol;Acc:2928]
rs2733459	X	DMD	HGNC Symbol	dystrophin [Source:HGNC Symbol;Acc:2928]
rs2178618	X	DMD	HGNC Symbol	dystrophin [Source:HGNC Symbol;Acc:2928]
rs61633449	X	DMD	HGNC Symbol	dystrophin [Source:HGNC Symbol;Acc:2928]
rs2704904	X	DMD	HGNC Symbol	dystrophin [Source:HGNC Symbol;Acc:2928]
rs2704908	X	DMD	HGNC Symbol	dystrophin [Source:HGNC Symbol;Acc:2928]
rs2692984	X	DMD	HGNC Symbol	dystrophin [Source:HGNC Symbol;Acc:2928]
rs5927747	X	DMD	HGNC Symbol	dystrophin [Source:HGNC Symbol;Acc:2928]
rs16998181	X	DMD	HGNC Symbol	dystrophin [Source:HGNC Symbol;Acc:2928]
rs58502991	X	DMD	HGNC Symbol	dystrophin [Source:HGNC Symbol;Acc:2928]
rs7062234	X	DMD	HGNC Symbol	dystrophin [Source:HGNC Symbol;Acc:2928]

SNP	Chromosome	gene name	gene source	description
rs16989598	X	DMD	HGNC Symbol	dystrophin [Source:HGNC Symbol;Acc:2928]
rs2704909	X	DMD	HGNC Symbol	dystrophin [Source:HGNC Symbol;Acc:2928]
rs17338507	X	DMD	HGNC Symbol	dystrophin [Source:HGNC Symbol;Acc:2928]
rs2733466	X	DMD	HGNC Symbol	dystrophin [Source:HGNC Symbol;Acc:2928]
rs6527086	X	DMD	HGNC Symbol	dystrophin [Source:HGNC Symbol;Acc:2928]
rs1921968	X	DMD	HGNC Symbol	dystrophin [Source:HGNC Symbol;Acc:2928]
rs17329999	X	DMD	HGNC Symbol	dystrophin [Source:HGNC Symbol;Acc:2928]
rs7054748	X	DMD	HGNC Symbol	dystrophin [Source:HGNC Symbol;Acc:2928]
rs17330006	X	DMD	HGNC Symbol	dystrophin [Source:HGNC Symbol;Acc:2928]
rs17338535	X	DMD	HGNC Symbol	dystrophin [Source:HGNC Symbol;Acc:2928]
rs4829222	X	DMD	HGNC Symbol	dystrophin [Source:HGNC Symbol;Acc:2928]
rs1112432	X	DMD	HGNC Symbol	dystrophin [Source:HGNC Symbol;Acc:2928]
rs73617087	X	DMD	HGNC Symbol	dystrophin [Source:HGNC Symbol;Acc:2928]
rs34151290	X	DMD	HGNC Symbol	dystrophin [Source:HGNC Symbol;Acc:2928]
rs7064141	X	DMD	HGNC Symbol	dystrophin [Source:HGNC Symbol;Acc:2928]
rs17340798	X	DMD	HGNC Symbol	dystrophin [Source:HGNC Symbol;Acc:2928]
rs12014023	X	DMD	HGNC Symbol	dystrophin [Source:HGNC Symbol;Acc:2928]
rs59882236	X	DMD	HGNC Symbol	dystrophin [Source:HGNC Symbol;Acc:2928]
rs72466566	X	DMD	HGNC Symbol	dystrophin [Source:HGNC Symbol;Acc:2928]
rs1800280	X	DMD	HGNC Symbol	dystrophin [Source:HGNC Symbol;Acc:2928]
rs1800279	X	DMD	HGNC Symbol	dystrophin [Source:HGNC Symbol;Acc:2928]
rs1800278	X	DMD	HGNC Symbol	dystrophin [Source:HGNC Symbol;Acc:2928]
rs72466572	X	DMD	HGNC Symbol	dystrophin [Source:HGNC Symbol;Acc:2928]
rs17338604	X	DMD	HGNC Symbol	dystrophin [Source:HGNC Symbol;Acc:2928]
rs6628610	X	DMD	HGNC Symbol	dystrophin [Source:HGNC Symbol;Acc:2928]
rs55875700	X	DMD	HGNC Symbol	dystrophin [Source:HGNC Symbol;Acc:2928]
rs16989681	X	DMD	HGNC Symbol	dystrophin [Source:HGNC Symbol;Acc:2928]
rs116594347	X	DMD	HGNC Symbol	dystrophin [Source:HGNC Symbol;Acc:2928]
rs5927762	X	DMD	HGNC Symbol	dystrophin [Source:HGNC Symbol;Acc:2928]
rs10126933	X	DMD	HGNC Symbol	dystrophin [Source:HGNC Symbol;Acc:2928]
rs5927017	X	DMD	HGNC Symbol	dystrophin [Source:HGNC Symbol;Acc:2928]
rs5927764	X	DMD	HGNC Symbol	dystrophin [Source:HGNC Symbol;Acc:2928]
rs7881088	X	DMD	HGNC Symbol	dystrophin [Source:HGNC Symbol;Acc:2928]

SNP	Chromosome	gene name	gene source	description
rs5972404	X	DMD	HGNC Symbol	dystrophin [Source:HGNC Symbol;Acc:2928]
rs17338611	X	DMD	HGNC Symbol	dystrophin [Source:HGNC Symbol;Acc:2928]
rs11797468	X	DMD	HGNC Symbol	dystrophin [Source:HGNC Symbol;Acc:2928]
rs5927779	X	DMD	HGNC Symbol	dystrophin [Source:HGNC Symbol;Acc:2928]
rs716354	X	DMD	HGNC Symbol	dystrophin [Source:HGNC Symbol;Acc:2928]
rs112870072	X	DMD	HGNC Symbol	dystrophin [Source:HGNC Symbol;Acc:2928]
rs34542798	X	DMD	HGNC Symbol	dystrophin [Source:HGNC Symbol;Acc:2928]
rs62587358	X	DMD	HGNC Symbol	dystrophin [Source:HGNC Symbol;Acc:2928]
rs5971586	X	DMD	HGNC Symbol	dystrophin [Source:HGNC Symbol;Acc:2928]
rs73210126	X	DMD	HGNC Symbol	dystrophin [Source:HGNC Symbol;Acc:2928]
rs5927030	X	DMD	HGNC Symbol	dystrophin [Source:HGNC Symbol;Acc:2928]
rs1540705	X	DMD	HGNC Symbol	dystrophin [Source:HGNC Symbol;Acc:2928]
rs17283259	X	DMD	HGNC Symbol	dystrophin [Source:HGNC Symbol;Acc:2928]
rs766671	X	DMD	HGNC Symbol	dystrophin [Source:HGNC Symbol;Acc:2928]
rs140658704	X	DMD	HGNC Symbol	dystrophin [Source:HGNC Symbol;Acc:2928]
rs5927822	X	DMD	HGNC Symbol	dystrophin [Source:HGNC Symbol;Acc:2928]
rs17330048	X	DMD	HGNC Symbol	dystrophin [Source:HGNC Symbol;Acc:2928]
rs73210155	X	DMD	HGNC Symbol	dystrophin [Source:HGNC Symbol;Acc:2928]
rs5927825	X	DMD	HGNC Symbol	dystrophin [Source:HGNC Symbol;Acc:2928]
rs73210174	X	DMD	HGNC Symbol	dystrophin [Source:HGNC Symbol;Acc:2928]
rs56678497	X	DMD	HGNC Symbol	dystrophin [Source:HGNC Symbol;Acc:2928]
rs4433306	X	DMD	HGNC Symbol	dystrophin [Source:HGNC Symbol;Acc:2928]
rs73213848	X	DMD	HGNC Symbol	dystrophin [Source:HGNC Symbol;Acc:2928]
rs2646305	X	DMD	HGNC Symbol	dystrophin [Source:HGNC Symbol;Acc:2928]
rs112041498	X	DMD	HGNC Symbol	dystrophin [Source:HGNC Symbol;Acc:2928]
rs10521979	X	DMD	HGNC Symbol	dystrophin [Source:HGNC Symbol;Acc:2928]
rs112663088	X	DMD	HGNC Symbol	dystrophin [Source:HGNC Symbol;Acc:2928]
rs5972445	X	DMD	HGNC Symbol	dystrophin [Source:HGNC Symbol;Acc:2928]
rs5927042	X	DMD	HGNC Symbol	dystrophin [Source:HGNC Symbol;Acc:2928]
rs6628646	X	DMD	HGNC Symbol	dystrophin [Source:HGNC Symbol;Acc:2928]
rs73213882	X	DMD	HGNC Symbol	dystrophin [Source:HGNC Symbol;Acc:2928]
rs73464049	X	DMD	HGNC Symbol	dystrophin [Source:HGNC Symbol;Acc:2928]
rs73619010	X	DMD	HGNC Symbol	dystrophin [Source:HGNC Symbol;Acc:2928]

SNP	Chromosome	gene name	gene source	description
rs148452632	X	DMD	HGNC Symbol	dystrophin [Source:HGNC Symbol;Acc:2928]
rs2952922	X	DMD	HGNC Symbol	dystrophin [Source:HGNC Symbol;Acc:2928]
rs17341038	X	DMD	HGNC Symbol	dystrophin [Source:HGNC Symbol;Acc:2928]
rs56279781	X	DMD	HGNC Symbol	dystrophin [Source:HGNC Symbol;Acc:2928]
rs5972452	X	DMD	HGNC Symbol	dystrophin [Source:HGNC Symbol;Acc:2928]
rs1545662	X	DMD	HGNC Symbol	dystrophin [Source:HGNC Symbol;Acc:2928]
rs12557076	X	DMD	HGNC Symbol	dystrophin [Source:HGNC Symbol;Acc:2928]
rs147024633	X	DMD	HGNC Symbol	dystrophin [Source:HGNC Symbol;Acc:2928]
rs7052181	X	DMD	HGNC Symbol	dystrophin [Source:HGNC Symbol;Acc:2928]
rs7892505	X	DMD	HGNC Symbol	dystrophin [Source:HGNC Symbol;Acc:2928]
rs66520172	X	DMD	HGNC Symbol	dystrophin [Source:HGNC Symbol;Acc:2928]
rs17341045	X	DMD	HGNC Symbol	dystrophin [Source:HGNC Symbol;Acc:2928]
rs16998240	X	DMD	HGNC Symbol	dystrophin [Source:HGNC Symbol;Acc:2928]
rs55787823	X	DMD	HGNC Symbol	dystrophin [Source:HGNC Symbol;Acc:2928]
rs73217964	X	DMD	HGNC Symbol	dystrophin [Source:HGNC Symbol;Acc:2928]
rs1463495	X	DMD	HGNC Symbol	dystrophin [Source:HGNC Symbol;Acc:2928]
rs6527132	X	DMD	HGNC Symbol	dystrophin [Source:HGNC Symbol;Acc:2928]
rs140820221	X	DMD	HGNC Symbol	dystrophin [Source:HGNC Symbol;Acc:2928]
rs73213546	X	DMD	HGNC Symbol	dystrophin [Source:HGNC Symbol;Acc:2928]
rs1350477	X	DMD	HGNC Symbol	dystrophin [Source:HGNC Symbol;Acc:2928]
rs73619021	X	DMD	HGNC Symbol	dystrophin [Source:HGNC Symbol;Acc:2928]
rs3761604	X	DMD	HGNC Symbol	dystrophin [Source:HGNC Symbol;Acc:2928]
rs1800273	X	DMD	HGNC Symbol	dystrophin [Source:HGNC Symbol;Acc:2928]
rs113404060	X	DMD	HGNC Symbol	dystrophin [Source:HGNC Symbol;Acc:2928]
rs114484622	X	DMD	HGNC Symbol	dystrophin [Source:HGNC Symbol;Acc:2928]
rs5972470	X	DMD	HGNC Symbol	dystrophin [Source:HGNC Symbol;Acc:2928]
rs16990005	X	DMD	HGNC Symbol	dystrophin [Source:HGNC Symbol;Acc:2928]
rs1293908	X	DMD	HGNC Symbol	dystrophin [Source:HGNC Symbol;Acc:2928]
rs5972472	X	DMD	HGNC Symbol	dystrophin [Source:HGNC Symbol;Acc:2928]
rs12007475	X	DMD	HGNC Symbol	dystrophin [Source:HGNC Symbol;Acc:2928]
rs12014251	X	DMD	HGNC Symbol	dystrophin [Source:HGNC Symbol;Acc:2928]
rs1293891	X	DMD	HGNC Symbol	dystrophin [Source:HGNC Symbol;Acc:2928]
rs1293905	X	DMD	HGNC Symbol	dystrophin [Source:HGNC Symbol;Acc:2928]

SNP	Chromosome	gene name	gene source	description
rs12010294	X	DMD	HGNC Symbol	dystrophin [Source:HGNC Symbol;Acc:2928]
rs1293906	X	DMD	HGNC Symbol	dystrophin [Source:HGNC Symbol;Acc:2928]
rs12008458	X	DMD	HGNC Symbol	dystrophin [Source:HGNC Symbol;Acc:2928]
rs1293875	X	DMD	HGNC Symbol	dystrophin [Source:HGNC Symbol;Acc:2928]
rs17270765	X	DMD	HGNC Symbol	dystrophin [Source:HGNC Symbol;Acc:2928]
rs2253300	X	DMD	HGNC Symbol	dystrophin [Source:HGNC Symbol;Acc:2928]
rs5927924	X	DMD	HGNC Symbol	dystrophin [Source:HGNC Symbol;Acc:2928]
rs1293924	X	DMD	HGNC Symbol	dystrophin [Source:HGNC Symbol;Acc:2928]
rs144824129	X	DMD	HGNC Symbol	dystrophin [Source:HGNC Symbol;Acc:2928]
rs149058642	X	DMD	HGNC Symbol	dystrophin [Source:HGNC Symbol;Acc:2928]
rs66707838	X	DMD	HGNC Symbol	dystrophin [Source:HGNC Symbol;Acc:2928]
rs7054546	X	DMD	HGNC Symbol	dystrophin [Source:HGNC Symbol;Acc:2928]
rs12556642	X	DMD	HGNC Symbol	dystrophin [Source:HGNC Symbol;Acc:2928]
rs35992741	X	DMD	HGNC Symbol	dystrophin [Source:HGNC Symbol;Acc:2928]
rs12842666	X	DMD	HGNC Symbol	dystrophin [Source:HGNC Symbol;Acc:2928]
rs41469547	X	DMD	HGNC Symbol	dystrophin [Source:HGNC Symbol;Acc:2928]
rs7059099	X	DMD	HGNC Symbol	dystrophin [Source:HGNC Symbol;Acc:2928]
rs6631472	X	DMD	HGNC Symbol	dystrophin [Source:HGNC Symbol;Acc:2928]
rs62589974	X	DMD	HGNC Symbol	dystrophin [Source:HGNC Symbol;Acc:2928]
rs16998256	X	DMD	HGNC Symbol	dystrophin [Source:HGNC Symbol;Acc:2928]
rs6418637	X	DMD	HGNC Symbol	dystrophin [Source:HGNC Symbol;Acc:2928]
rs6628673	X	DMD	HGNC Symbol	dystrophin [Source:HGNC Symbol;Acc:2928]
rs16990071	X	DMD	HGNC Symbol	dystrophin [Source:HGNC Symbol;Acc:2928]
rs16998260	X	DMD	HGNC Symbol	dystrophin [Source:HGNC Symbol;Acc:2928]
rs1718043	X	DMD	HGNC Symbol	dystrophin [Source:HGNC Symbol;Acc:2928]
rs73619026	X	DMD	HGNC Symbol	dystrophin [Source:HGNC Symbol;Acc:2928]
rs2141753	X	DMD	HGNC Symbol	dystrophin [Source:HGNC Symbol;Acc:2928]
rs56663344	X	DMD	HGNC Symbol	dystrophin [Source:HGNC Symbol;Acc:2928]
rs2897164	X	DMD	HGNC Symbol	dystrophin [Source:HGNC Symbol;Acc:2928]
rs5972488	X	DMD	HGNC Symbol	dystrophin [Source:HGNC Symbol;Acc:2928]
rs6631486	X	DMD	HGNC Symbol	dystrophin [Source:HGNC Symbol;Acc:2928]
rs55982304	X	DMD	HGNC Symbol	dystrophin [Source:HGNC Symbol;Acc:2928]
rs1795578	X	DMD	HGNC Symbol	dystrophin [Source:HGNC Symbol;Acc:2928]

SNP	Chromosome	gene name	gene source	description
rs12840034	X	DMD	HGNC Symbol	dystrophin [Source:HGNC Symbol;Acc:2928]
rs1718039	X	DMD	HGNC Symbol	dystrophin [Source:HGNC Symbol;Acc:2928]
rs6631492	X	DMD	HGNC Symbol	dystrophin [Source:HGNC Symbol;Acc:2928]
rs7882019	X	DMD	HGNC Symbol	dystrophin [Source:HGNC Symbol;Acc:2928]
rs6653578	X	DMD	HGNC Symbol	dystrophin [Source:HGNC Symbol;Acc:2928]
rs16990111	X	DMD	HGNC Symbol	dystrophin [Source:HGNC Symbol;Acc:2928]
rs12843752	X	DMD	HGNC Symbol	dystrophin [Source:HGNC Symbol;Acc:2928]
rs61549924	X	DMD	HGNC Symbol	dystrophin [Source:HGNC Symbol;Acc:2928]
rs16990116	X	DMD	HGNC Symbol	dystrophin [Source:HGNC Symbol;Acc:2928]
rs1718041	X	DMD	HGNC Symbol	dystrophin [Source:HGNC Symbol;Acc:2928]
rs1795592	X	DMD	HGNC Symbol	dystrophin [Source:HGNC Symbol;Acc:2928]
rs1795595	X	DMD	HGNC Symbol	dystrophin [Source:HGNC Symbol;Acc:2928]
rs68092862	X	DMD	HGNC Symbol	dystrophin [Source:HGNC Symbol;Acc:2928]
rs146532983	X	DMD	HGNC Symbol	dystrophin [Source:HGNC Symbol;Acc:2928]
rs10127060	X	DMD	HGNC Symbol	dystrophin [Source:HGNC Symbol;Acc:2928]
rs11095222	X	DMD	HGNC Symbol	dystrophin [Source:HGNC Symbol;Acc:2928]
rs5927938	X	DMD	HGNC Symbol	dystrophin [Source:HGNC Symbol;Acc:2928]
rs1317098	X	DMD	HGNC Symbol	dystrophin [Source:HGNC Symbol;Acc:2928]
rs16990122	X	DMD	HGNC Symbol	dystrophin [Source:HGNC Symbol;Acc:2928]
rs6628677	X	DMD	HGNC Symbol	dystrophin [Source:HGNC Symbol;Acc:2928]
rs7065982	X	DMD	HGNC Symbol	dystrophin [Source:HGNC Symbol;Acc:2928]
rs5972499	X	DMD	HGNC Symbol	dystrophin [Source:HGNC Symbol;Acc:2928]
rs2701794	X	DMD	HGNC Symbol	dystrophin [Source:HGNC Symbol;Acc:2928]
rs1795579	X	DMD	HGNC Symbol	dystrophin [Source:HGNC Symbol;Acc:2928]
rs1718053	X	DMD	HGNC Symbol	dystrophin [Source:HGNC Symbol;Acc:2928]
rs55672607	X	DMD	HGNC Symbol	dystrophin [Source:HGNC Symbol;Acc:2928]
rs59399365	X	DMD	HGNC Symbol	dystrophin [Source:HGNC Symbol;Acc:2928]
rs10126878	X	DMD	HGNC Symbol	dystrophin [Source:HGNC Symbol;Acc:2928]
rs1718047	X	DMD	HGNC Symbol	dystrophin [Source:HGNC Symbol;Acc:2928]
rs2405684	X	DMD	HGNC Symbol	dystrophin [Source:HGNC Symbol;Acc:2928]
rs72626020	X	DMD	HGNC Symbol	dystrophin [Source:HGNC Symbol;Acc:2928]
rs1795588	X	DMD	HGNC Symbol	dystrophin [Source:HGNC Symbol;Acc:2928]
rs12833101	X	DMD	HGNC Symbol	dystrophin [Source:HGNC Symbol;Acc:2928]

SNP	Chromosome	gene name	gene source	description
rs16990140	X	DMD	HGNC Symbol	dystrophin [Source:HGNC Symbol;Acc:2928]
rs2606663	X	DMD	HGNC Symbol	dystrophin [Source:HGNC Symbol;Acc:2928]
rs2606664	X	DMD	HGNC Symbol	dystrophin [Source:HGNC Symbol;Acc:2928]
rs2685908	X	DMD	HGNC Symbol	dystrophin [Source:HGNC Symbol;Acc:2928]
rs2606665	X	DMD	HGNC Symbol	dystrophin [Source:HGNC Symbol;Acc:2928]
rs2606683	X	DMD	HGNC Symbol	dystrophin [Source:HGNC Symbol;Acc:2928]
rs2685909	X	DMD	HGNC Symbol	dystrophin [Source:HGNC Symbol;Acc:2928]
rs1456734	X	DMD	HGNC Symbol	dystrophin [Source:HGNC Symbol;Acc:2928]
rs5927942	X	DMD	HGNC Symbol	dystrophin [Source:HGNC Symbol;Acc:2928]
rs2685891	X	DMD	HGNC Symbol	dystrophin [Source:HGNC Symbol;Acc:2928]
rs7879462	X	DMD	HGNC Symbol	dystrophin [Source:HGNC Symbol;Acc:2928]
rs2685892	X	DMD	HGNC Symbol	dystrophin [Source:HGNC Symbol;Acc:2928]
rs2606667	X	DMD	HGNC Symbol	dystrophin [Source:HGNC Symbol;Acc:2928]
rs73458036	X	DMD	HGNC Symbol	dystrophin [Source:HGNC Symbol;Acc:2928]
rs2685893	X	DMD	HGNC Symbol	dystrophin [Source:HGNC Symbol;Acc:2928]
rs58151710	X	DMD	HGNC Symbol	dystrophin [Source:HGNC Symbol;Acc:2928]
rs2685896	X	DMD	HGNC Symbol	dystrophin [Source:HGNC Symbol;Acc:2928]
rs2606672	X	DMD	HGNC Symbol	dystrophin [Source:HGNC Symbol;Acc:2928]
rs145272492	X	DMD	HGNC Symbol	dystrophin [Source:HGNC Symbol;Acc:2928]
rs7058785	X	DMD	HGNC Symbol	dystrophin [Source:HGNC Symbol;Acc:2928]
rs5972504	X	DMD	HGNC Symbol	dystrophin [Source:HGNC Symbol;Acc:2928]
rs73458061	X	DMD	HGNC Symbol	dystrophin [Source:HGNC Symbol;Acc:2928]
rs1456737	X	DMD	HGNC Symbol	dystrophin [Source:HGNC Symbol;Acc:2928]
rs2685902	X	DMD	HGNC Symbol	dystrophin [Source:HGNC Symbol;Acc:2928]
rs874830	X	DMD	HGNC Symbol	dystrophin [Source:HGNC Symbol;Acc:2928]
rs16990166	X	DMD	HGNC Symbol	dystrophin [Source:HGNC Symbol;Acc:2928]
rs2854966	X	DMD	HGNC Symbol	dystrophin [Source:HGNC Symbol;Acc:2928]
rs55686834	X	DMD	HGNC Symbol	dystrophin [Source:HGNC Symbol;Acc:2928]
rs331368	X	DMD	HGNC Symbol	dystrophin [Source:HGNC Symbol;Acc:2928]
rs331362	X	DMD	HGNC Symbol	dystrophin [Source:HGNC Symbol;Acc:2928]
rs61661964	X	DMD	HGNC Symbol	dystrophin [Source:HGNC Symbol;Acc:2928]
rs143066970	X	DMD	HGNC Symbol	dystrophin [Source:HGNC Symbol;Acc:2928]
rs111826558	X	DMD	HGNC Symbol	dystrophin [Source:HGNC Symbol;Acc:2928]

SNP	Chromosome	gene name	gene source	description
rs966451	X	DMD	HGNC Symbol	dystrophin [Source:HGNC Symbol;Acc:2928]
rs5927948	X	DMD	HGNC Symbol	dystrophin [Source:HGNC Symbol;Acc:2928]
rs1456727	X	DMD	HGNC Symbol	dystrophin [Source:HGNC Symbol;Acc:2928]
rs1456729	X	DMD	HGNC Symbol	dystrophin [Source:HGNC Symbol;Acc:2928]
rs331349	X	DMD	HGNC Symbol	dystrophin [Source:HGNC Symbol;Acc:2928]
rs12400794	X	DMD	HGNC Symbol	dystrophin [Source:HGNC Symbol;Acc:2928]
rs6631536	X	DMD	HGNC Symbol	dystrophin [Source:HGNC Symbol;Acc:2928]
rs1160301	X	DMD	HGNC Symbol	dystrophin [Source:HGNC Symbol;Acc:2928]
rs1408983	X	DMD	HGNC Symbol	dystrophin [Source:HGNC Symbol;Acc:2928]
rs331331	X	DMD	HGNC Symbol	dystrophin [Source:HGNC Symbol;Acc:2928]
rs331323	X	DMD	HGNC Symbol	dystrophin [Source:HGNC Symbol;Acc:2928]
rs6418643	X	DMD	HGNC Symbol	dystrophin [Source:HGNC Symbol;Acc:2928]
rs62590662	X	DMD	HGNC Symbol	dystrophin [Source:HGNC Symbol;Acc:2928]
rs331321	X	DMD	HGNC Symbol	dystrophin [Source:HGNC Symbol;Acc:2928]
rs12007028	X	DMD	HGNC Symbol	dystrophin [Source:HGNC Symbol;Acc:2928]
rs331318	X	DMD	HGNC Symbol	dystrophin [Source:HGNC Symbol;Acc:2928]
rs331317	X	DMD	HGNC Symbol	dystrophin [Source:HGNC Symbol;Acc:2928]
rs331316	X	DMD	HGNC Symbol	dystrophin [Source:HGNC Symbol;Acc:2928]
rs189117	X	DMD	HGNC Symbol	dystrophin [Source:HGNC Symbol;Acc:2928]
rs73221139	X	DMD	HGNC Symbol	dystrophin [Source:HGNC Symbol;Acc:2928]
rs331313	X	DMD	HGNC Symbol	dystrophin [Source:HGNC Symbol;Acc:2928]
rs115862380	X	DMD	HGNC Symbol	dystrophin [Source:HGNC Symbol;Acc:2928]
rs5927966	X	DMD	HGNC Symbol	dystrophin [Source:HGNC Symbol;Acc:2928]
rs35961347	X	DMD	HGNC Symbol	dystrophin [Source:HGNC Symbol;Acc:2928]
rs12388894	X	DMD	HGNC Symbol	dystrophin [Source:HGNC Symbol;Acc:2928]
rs143091919	X	DMD	HGNC Symbol	dystrophin [Source:HGNC Symbol;Acc:2928]
rs2295329	X	DMD	HGNC Symbol	dystrophin [Source:HGNC Symbol;Acc:2928]
rs72468628	X	DMD	HGNC Symbol	dystrophin [Source:HGNC Symbol;Acc:2928]
rs1293821	X	DMD	HGNC Symbol	dystrophin [Source:HGNC Symbol;Acc:2928]
rs5927972	X	DMD	HGNC Symbol	dystrophin [Source:HGNC Symbol;Acc:2928]
rs5927974	X	DMD	HGNC Symbol	dystrophin [Source:HGNC Symbol;Acc:2928]
rs1801187	X	DMD	HGNC Symbol	dystrophin [Source:HGNC Symbol;Acc:2928]
rs1963703	X	DMD	HGNC Symbol	dystrophin [Source:HGNC Symbol;Acc:2928]

SNP	Chromosome	gene name	gene source	description
rs5972548	X	DMD	HGNC Symbol	dystrophin [Source:HGNC Symbol;Acc:2928]
rs16990296	X	DMD	HGNC Symbol	dystrophin [Source:HGNC Symbol;Acc:2928]
rs73222843	X	DMD	HGNC Symbol	dystrophin [Source:HGNC Symbol;Acc:2928]
rs73619075	X	DMD	HGNC Symbol	dystrophin [Source:HGNC Symbol;Acc:2928]
rs10521994	X	DMD	HGNC Symbol	dystrophin [Source:HGNC Symbol;Acc:2928]
rs7053451	X	DMD	HGNC Symbol	dystrophin [Source:HGNC Symbol;Acc:2928]
rs16990375	X	DMD	HGNC Symbol	dystrophin [Source:HGNC Symbol;Acc:2928]
rs2405829	X	DMD	HGNC Symbol	dystrophin [Source:HGNC Symbol;Acc:2928]
rs2076366	X	DMD	HGNC Symbol	dystrophin [Source:HGNC Symbol;Acc:2928]
rs1800269	X	DMD	HGNC Symbol	dystrophin [Source:HGNC Symbol;Acc:2928]
rs56027026	X	DMD	HGNC Symbol	dystrophin [Source:HGNC Symbol;Acc:2928]
rs5972576	X	DMD	HGNC Symbol	dystrophin [Source:HGNC Symbol;Acc:2928]
rs6527202	X	DMD	HGNC Symbol	dystrophin [Source:HGNC Symbol;Acc:2928]
rs228390	X	DMD	HGNC Symbol	dystrophin [Source:HGNC Symbol;Acc:2928]
rs5972579	X	DMD	HGNC Symbol	dystrophin [Source:HGNC Symbol;Acc:2928]
rs228405	X	DMD	HGNC Symbol	dystrophin [Source:HGNC Symbol;Acc:2928]
rs228406	X	DMD	HGNC Symbol	dystrophin [Source:HGNC Symbol;Acc:2928]
rs5972580	X	DMD	HGNC Symbol	dystrophin [Source:HGNC Symbol;Acc:2928]
rs228312	X	DMD	HGNC Symbol	dystrophin [Source:HGNC Symbol;Acc:2928]
rs5972586	X	DMD	HGNC Symbol	dystrophin [Source:HGNC Symbol;Acc:2928]
rs228324	X	DMD	HGNC Symbol	dystrophin [Source:HGNC Symbol;Acc:2928]
rs6527205	X	DMD	HGNC Symbol	dystrophin [Source:HGNC Symbol;Acc:2928]
rs228327	X	DMD	HGNC Symbol	dystrophin [Source:HGNC Symbol;Acc:2928]
rs228336	X	DMD	HGNC Symbol	dystrophin [Source:HGNC Symbol;Acc:2928]
rs5928002	X	DMD	HGNC Symbol	dystrophin [Source:HGNC Symbol;Acc:2928]
rs228354	X	DMD	HGNC Symbol	dystrophin [Source:HGNC Symbol;Acc:2928]
rs73207771	X	DMD	HGNC Symbol	dystrophin [Source:HGNC Symbol;Acc:2928]
rs228355	X	DMD	HGNC Symbol	dystrophin [Source:HGNC Symbol;Acc:2928]
rs10126490	X	DMD	HGNC Symbol	dystrophin [Source:HGNC Symbol;Acc:2928]
rs228378	X	DMD	HGNC Symbol	dystrophin [Source:HGNC Symbol;Acc:2928]
rs140474271	X	DMD	HGNC Symbol	dystrophin [Source:HGNC Symbol;Acc:2928]
rs1435733	X	DMD	HGNC Symbol	dystrophin [Source:HGNC Symbol;Acc:2928]
rs4829123	X	DMD	HGNC Symbol	dystrophin [Source:HGNC Symbol;Acc:2928]

SNP	Chromosome	gene name	gene source	description
rs808578	X	DMD	HGNC Symbol	dystrophin [Source:HGNC Symbol;Acc:2928]
rs808576	X	DMD	HGNC Symbol	dystrophin [Source:HGNC Symbol;Acc:2928]
rs7057273	X	DMD	HGNC Symbol	dystrophin [Source:HGNC Symbol;Acc:2928]
rs4829261	X	DMD	HGNC Symbol	dystrophin [Source:HGNC Symbol;Acc:2928]
rs6653878	X	DMD	HGNC Symbol	dystrophin [Source:HGNC Symbol;Acc:2928]
rs808573	X	DMD	HGNC Symbol	dystrophin [Source:HGNC Symbol;Acc:2928]
rs5927083	X	DMD	HGNC Symbol	dystrophin [Source:HGNC Symbol;Acc:2928]
rs5928027	X	DMD	HGNC Symbol	dystrophin [Source:HGNC Symbol;Acc:2928]
rs808547	X	DMD	HGNC Symbol	dystrophin [Source:HGNC Symbol;Acc:2928]
rs808536	X	DMD	HGNC Symbol	dystrophin [Source:HGNC Symbol;Acc:2928]
rs5928040	X	DMD	HGNC Symbol	dystrophin [Source:HGNC Symbol;Acc:2928]
rs808520	X	DMD	HGNC Symbol	dystrophin [Source:HGNC Symbol;Acc:2928]
rs808518	X	DMD	HGNC Symbol	dystrophin [Source:HGNC Symbol;Acc:2928]
rs142762831	X	DMD	HGNC Symbol	dystrophin [Source:HGNC Symbol;Acc:2928]
rs149352277	X	DMD	HGNC Symbol	dystrophin [Source:HGNC Symbol;Acc:2928]
rs808513	X	DMD	HGNC Symbol	dystrophin [Source:HGNC Symbol;Acc:2928]
rs57110381	X	DMD	HGNC Symbol	dystrophin [Source:HGNC Symbol;Acc:2928]
rs5927089	X	DMD	HGNC Symbol	dystrophin [Source:HGNC Symbol;Acc:2928]
rs5928062	X	DMD	HGNC Symbol	dystrophin [Source:HGNC Symbol;Acc:2928]
rs72470511	X	DMD	HGNC Symbol	dystrophin [Source:HGNC Symbol;Acc:2928]
rs57916323	X	DMD	HGNC Symbol	dystrophin [Source:HGNC Symbol;Acc:2928]
rs112427626	X	DMD	HGNC Symbol	dystrophin [Source:HGNC Symbol;Acc:2928]
rs5972643	X	DMD	HGNC Symbol	dystrophin [Source:HGNC Symbol;Acc:2928]
rs5972644	X	DMD	HGNC Symbol	dystrophin [Source:HGNC Symbol;Acc:2928]
rs5972645	X	DMD	HGNC Symbol	dystrophin [Source:HGNC Symbol;Acc:2928]
rs5928067	X	DMD	HGNC Symbol	dystrophin [Source:HGNC Symbol;Acc:2928]
rs146155628	X	DMD	HGNC Symbol	dystrophin [Source:HGNC Symbol;Acc:2928]
rs5928069	X	DMD	HGNC Symbol	dystrophin [Source:HGNC Symbol;Acc:2928]
rs12845594	X	DMD	HGNC Symbol	dystrophin [Source:HGNC Symbol;Acc:2928]
rs1800265	X	DMD	HGNC Symbol	dystrophin [Source:HGNC Symbol;Acc:2928]
rs72470514	X	DMD	HGNC Symbol	dystrophin [Source:HGNC Symbol;Acc:2928]
rs41303183	X	DMD	HGNC Symbol	dystrophin [Source:HGNC Symbol;Acc:2928]
rs73209881	X	DMD	HGNC Symbol	dystrophin [Source:HGNC Symbol;Acc:2928]

SNP	Chromosome	gene name	gene source	description
rs141379267	X	DMD	HGNC Symbol	dystrophin [Source:HGNC Symbol;Acc:2928]
rs6653885	X	DMD	HGNC Symbol	dystrophin [Source:HGNC Symbol;Acc:2928]
rs12116231	X	DMD	HGNC Symbol	dystrophin [Source:HGNC Symbol;Acc:2928]
rs2748307	X	DMD	HGNC Symbol	dystrophin [Source:HGNC Symbol;Acc:2928]
rs67726258	X	DMD	HGNC Symbol	dystrophin [Source:HGNC Symbol;Acc:2928]
rs2748318	X	DMD	HGNC Symbol	dystrophin [Source:HGNC Symbol;Acc:2928]
rs2855695	X	DMD	HGNC Symbol	dystrophin [Source:HGNC Symbol;Acc:2928]
rs5972668	X	DMD	HGNC Symbol	dystrophin [Source:HGNC Symbol;Acc:2928]
rs141199105	X	DMD	HGNC Symbol	dystrophin [Source:HGNC Symbol;Acc:2928]
rs2855689	X	DMD	HGNC Symbol	dystrophin [Source:HGNC Symbol;Acc:2928]
rs7067109	X	DMD	HGNC Symbol	dystrophin [Source:HGNC Symbol;Acc:2928]
rs112382490	X	DMD	HGNC Symbol	dystrophin [Source:HGNC Symbol;Acc:2928]
rs5927099	X	DMD	HGNC Symbol	dystrophin [Source:HGNC Symbol;Acc:2928]
rs2855686	X	DMD	HGNC Symbol	dystrophin [Source:HGNC Symbol;Acc:2928]
rs1158629	X	DMD	HGNC Symbol	dystrophin [Source:HGNC Symbol;Acc:2928]
rs72470526	X	DMD	HGNC Symbol	dystrophin [Source:HGNC Symbol;Acc:2928]
rs139319218	X	DMD	HGNC Symbol	dystrophin [Source:HGNC Symbol;Acc:2928]
rs5928080	X	DMD	HGNC Symbol	dystrophin [Source:HGNC Symbol;Acc:2928]
rs4829269	X	DMD	HGNC Symbol	dystrophin [Source:HGNC Symbol;Acc:2928]
rs66760061	X	DMD	HGNC Symbol	dystrophin [Source:HGNC Symbol;Acc:2928]
rs5972684	X	DMD	HGNC Symbol	dystrophin [Source:HGNC Symbol;Acc:2928]
rs73621844	X	DMD	HGNC Symbol	dystrophin [Source:HGNC Symbol;Acc:2928]
rs5928089	X	DMD	HGNC Symbol	dystrophin [Source:HGNC Symbol;Acc:2928]
rs7879662	X	DMD	HGNC Symbol	dystrophin [Source:HGNC Symbol;Acc:2928]
rs17283421	X	DMD	HGNC Symbol	dystrophin [Source:HGNC Symbol;Acc:2928]
rs5972690	X	DMD	HGNC Symbol	dystrophin [Source:HGNC Symbol;Acc:2928]
rs3764763	X	DMD	HGNC Symbol	dystrophin [Source:HGNC Symbol;Acc:2928]
rs7880016	X	DMD	HGNC Symbol	dystrophin [Source:HGNC Symbol;Acc:2928]
rs5972699	X	DMD	HGNC Symbol	dystrophin [Source:HGNC Symbol;Acc:2928]
rs9887516	X	DMD	HGNC Symbol	dystrophin [Source:HGNC Symbol;Acc:2928]
rs5928095	X	DMD	HGNC Symbol	dystrophin [Source:HGNC Symbol;Acc:2928]
rs5005249	X	DMD	HGNC Symbol	dystrophin [Source:HGNC Symbol;Acc:2928]
rs5928099	X	DMD	HGNC Symbol	dystrophin [Source:HGNC Symbol;Acc:2928]

SNP	Chromosome	gene name	gene source	description
rs12395380	X	DMD	HGNC Symbol	dystrophin [Source:HGNC Symbol;Acc:2928]
rs12559198	X	DMD	HGNC Symbol	dystrophin [Source:HGNC Symbol;Acc:2928]
rs17338877	X	DMD	HGNC Symbol	dystrophin [Source:HGNC Symbol;Acc:2928]
rs7061782	X	DMD	HGNC Symbol	dystrophin [Source:HGNC Symbol;Acc:2928]
rs5928102	X	DMD	HGNC Symbol	dystrophin [Source:HGNC Symbol;Acc:2928]
rs6628748	X	DMD	HGNC Symbol	dystrophin [Source:HGNC Symbol;Acc:2928]
rs5928104	X	DMD	HGNC Symbol	dystrophin [Source:HGNC Symbol;Acc:2928]
rs17338898	X	DMD	HGNC Symbol	dystrophin [Source:HGNC Symbol;Acc:2928]
rs62593385	X	DMD	HGNC Symbol	dystrophin [Source:HGNC Symbol;Acc:2928]
rs5928109	X	DMD	HGNC Symbol	dystrophin [Source:HGNC Symbol;Acc:2928]
rs67636309	X	DMD	HGNC Symbol	dystrophin [Source:HGNC Symbol;Acc:2928]
rs5928111	X	DMD	HGNC Symbol	dystrophin [Source:HGNC Symbol;Acc:2928]
rs1321394	X	DMD	HGNC Symbol	dystrophin [Source:HGNC Symbol;Acc:2928]
rs1321395	X	DMD	HGNC Symbol	dystrophin [Source:HGNC Symbol;Acc:2928]
rs17330215	X	DMD	HGNC Symbol	dystrophin [Source:HGNC Symbol;Acc:2928]
rs10522017	X	DMD	HGNC Symbol	dystrophin [Source:HGNC Symbol;Acc:2928]
rs56982984	X	DMD	HGNC Symbol	dystrophin [Source:HGNC Symbol;Acc:2928]
rs6628752	X	DMD	HGNC Symbol	dystrophin [Source:HGNC Symbol;Acc:2928]
rs1570029	X	DMD	HGNC Symbol	dystrophin [Source:HGNC Symbol;Acc:2928]
rs5927113	X	DMD	HGNC Symbol	dystrophin [Source:HGNC Symbol;Acc:2928]
rs5972715	X	DMD	HGNC Symbol	dystrophin [Source:HGNC Symbol;Acc:2928]
rs113605304	X	DMD	HGNC Symbol	dystrophin [Source:HGNC Symbol;Acc:2928]
rs73466863	X	DMD	HGNC Symbol	dystrophin [Source:HGNC Symbol;Acc:2928]
rs2180648	X	DMD	HGNC Symbol	dystrophin [Source:HGNC Symbol;Acc:2928]
rs73188349	X	DMD	HGNC Symbol	dystrophin [Source:HGNC Symbol;Acc:2928]
rs5972721	X	DMD	HGNC Symbol	dystrophin [Source:HGNC Symbol;Acc:2928]
rs6527243	X	DMD	HGNC Symbol	dystrophin [Source:HGNC Symbol;Acc:2928]
rs55790705	X	DMD	HGNC Symbol	dystrophin [Source:HGNC Symbol;Acc:2928]
rs6628755	X	DMD	HGNC Symbol	dystrophin [Source:HGNC Symbol;Acc:2928]
rs4639663	X	DMD	HGNC Symbol	dystrophin [Source:HGNC Symbol;Acc:2928]
rs12837811	X	DMD	HGNC Symbol	dystrophin [Source:HGNC Symbol;Acc:2928]
rs5928139	X	DMD	HGNC Symbol	dystrophin [Source:HGNC Symbol;Acc:2928]
rs12843460	X	DMD	HGNC Symbol	dystrophin [Source:HGNC Symbol;Acc:2928]

SNP	Chromosome	gene name	gene source	description
rs7886739	X	DMD	HGNC Symbol	dystrophin [Source:HGNC Symbol;Acc:2928]
rs6631712	X	DMD	HGNC Symbol	dystrophin [Source:HGNC Symbol;Acc:2928]
rs73188265	X	DMD	HGNC Symbol	dystrophin [Source:HGNC Symbol;Acc:2928]
rs5928148	X	DMD	HGNC Symbol	dystrophin [Source:HGNC Symbol;Acc:2928]
rs17330270	X	DMD	HGNC Symbol	dystrophin [Source:HGNC Symbol;Acc:2928]
rs4345727	X	DMD	HGNC Symbol	dystrophin [Source:HGNC Symbol;Acc:2928]
rs5972737	X	DMD	HGNC Symbol	dystrophin [Source:HGNC Symbol;Acc:2928]
rs5927130	X	DMD	HGNC Symbol	dystrophin [Source:HGNC Symbol;Acc:2928]
rs66873920	X	DMD	HGNC Symbol	dystrophin [Source:HGNC Symbol;Acc:2928]
rs17338974	X	DMD	HGNC Symbol	dystrophin [Source:HGNC Symbol;Acc:2928]
rs2765379	X	DMD	HGNC Symbol	dystrophin [Source:HGNC Symbol;Acc:2928]
rs5972739	X	DMD	HGNC Symbol	dystrophin [Source:HGNC Symbol;Acc:2928]
rs2765381	X	DMD	HGNC Symbol	dystrophin [Source:HGNC Symbol;Acc:2928]
rs143027254	X	DMD	HGNC Symbol	dystrophin [Source:HGNC Symbol;Acc:2928]
rs73188288	X	DMD	HGNC Symbol	dystrophin [Source:HGNC Symbol;Acc:2928]
rs6631726	X	DMD	HGNC Symbol	dystrophin [Source:HGNC Symbol;Acc:2928]
rs2765387	X	DMD	HGNC Symbol	dystrophin [Source:HGNC Symbol;Acc:2928]
rs6631731	X	DMD	HGNC Symbol	dystrophin [Source:HGNC Symbol;Acc:2928]
rs5972743	X	DMD	HGNC Symbol	dystrophin [Source:HGNC Symbol;Acc:2928]
rs6418647	X	DMD	HGNC Symbol	dystrophin [Source:HGNC Symbol;Acc:2928]
rs5928167	X	DMD	HGNC Symbol	dystrophin [Source:HGNC Symbol;Acc:2928]
rs72626075	X	DMD	HGNC Symbol	dystrophin [Source:HGNC Symbol;Acc:2928]
rs16998378	X	DMD	HGNC Symbol	dystrophin [Source:HGNC Symbol;Acc:2928]
rs2094147	X	DMD	HGNC Symbol	dystrophin [Source:HGNC Symbol;Acc:2928]
rs2207086	X	DMD	HGNC Symbol	dystrophin [Source:HGNC Symbol;Acc:2928]
rs7064140	X	DMD	HGNC Symbol	dystrophin [Source:HGNC Symbol;Acc:2928]
rs2024614	X	DMD	HGNC Symbol	dystrophin [Source:HGNC Symbol;Acc:2928]
rs73190225	X	DMD	HGNC Symbol	dystrophin [Source:HGNC Symbol;Acc:2928]
rs10127390	X	DMD	HGNC Symbol	dystrophin [Source:HGNC Symbol;Acc:2928]
rs35874041	X	DMD	HGNC Symbol	dystrophin [Source:HGNC Symbol;Acc:2928]
rs5972769	X	DMD	HGNC Symbol	dystrophin [Source:HGNC Symbol;Acc:2928]
rs58843483	X	DMD	HGNC Symbol	dystrophin [Source:HGNC Symbol;Acc:2928]
rs5928193	X	DMD	HGNC Symbol	dystrophin [Source:HGNC Symbol;Acc:2928]

SNP	Chromosome	gene name	gene source	description
rs5971693	X	DMD	HGNC Symbol	dystrophin [Source:HGNC Symbol;Acc:2928]
rs5928201	X	DMD	HGNC Symbol	dystrophin [Source:HGNC Symbol;Acc:2928]
rs12557125	X	DMD	HGNC Symbol	dystrophin [Source:HGNC Symbol;Acc:2928]
rs5971696	X	DMD	HGNC Symbol	dystrophin [Source:HGNC Symbol;Acc:2928]
rs150494081	X	DMD	HGNC Symbol	dystrophin [Source:HGNC Symbol;Acc:2928]
rs5928207	X	DMD	HGNC Symbol	dystrophin [Source:HGNC Symbol;Acc:2928]
rs10284225	X	DMD	HGNC Symbol	dystrophin [Source:HGNC Symbol;Acc:2928]
rs73623931	X	DMD	HGNC Symbol	dystrophin [Source:HGNC Symbol;Acc:2928]
rs12392440	X	DMD	HGNC Symbol	dystrophin [Source:HGNC Symbol;Acc:2928]
rs72626080	X	DMD	HGNC Symbol	dystrophin [Source:HGNC Symbol;Acc:2928]
rs2050074	X	DMD	HGNC Symbol	dystrophin [Source:HGNC Symbol;Acc:2928]
rs2050076	X	DMD	HGNC Symbol	dystrophin [Source:HGNC Symbol;Acc:2928]
rs12396164	X	DMD	HGNC Symbol	dystrophin [Source:HGNC Symbol;Acc:2928]
rs5972802	X	DMD	HGNC Symbol	dystrophin [Source:HGNC Symbol;Acc:2928]
rs5972805	X	DMD	HGNC Symbol	dystrophin [Source:HGNC Symbol;Acc:2928]
rs60235628	X	DMD	HGNC Symbol	dystrophin [Source:HGNC Symbol;Acc:2928]
rs12559939	X	DMD	HGNC Symbol	dystrophin [Source:HGNC Symbol;Acc:2928]
rs3946125	X	DMD	HGNC Symbol	dystrophin [Source:HGNC Symbol;Acc:2928]
rs141927233	X	DMD	HGNC Symbol	dystrophin [Source:HGNC Symbol;Acc:2928]
rs73623943	X	DMD	HGNC Symbol	dystrophin [Source:HGNC Symbol;Acc:2928]
rs5972815	X	DMD	HGNC Symbol	dystrophin [Source:HGNC Symbol;Acc:2928]
rs17341316	X	DMD	HGNC Symbol	dystrophin [Source:HGNC Symbol;Acc:2928]
rs58029094	X	DMD	HGNC Symbol	dystrophin [Source:HGNC Symbol;Acc:2928]
rs17283561	X	DMD	HGNC Symbol	dystrophin [Source:HGNC Symbol;Acc:2928]
rs5927163				
rs12008340				
rs2406668				
rs5928244				
rs148926468				
rs9887051				
rs148664319				
rs5927166				
rs60398114				

SNP	Chromosome	gene name	gene source	description
rs6631789				
rs35534216				
rs5972850				
rs73192337				
rs5972863				
rs5928296				
rs5972867				
rs5971735				
rs66597629				
rs73194116				
rs73194121				
rs4829294				
rs13441039				
rs1356619				
rs5971745				
rs62593574				
rs17330430				
rs143580525				
rs1518978				
rs11095290				
rs17341385				
rs66953611				
rs5972938				
rs2382578				
rs5972941				
rs142253671				
rs146002754				
rs144605098	X	RP11-305F18.1	Clone-based (Vega) gene	
rs140950611	X	RP11-305F18.1	Clone-based (Vega) gene	
rs5928366	X	RP11-305F18.1	Clone-based (Vega) gene	

SNP	Chromosome	gene name	gene source	description
rs12387697	X	RP11-305F18.1	Clone-based (Vega) gene	
rs17330514	X	RP11-305F18.1	Clone-based (Vega) gene	
rs72626092	X	RP11-305F18.1	Clone-based (Vega) gene	
rs1870680	X	RP11-305F18.1	Clone-based (Vega) gene	
rs5928375	X	RP11-305F18.1	Clone-based (Vega) gene	
rs7066087	X	RP11-305F18.1	Clone-based (Vega) gene	
rs1393620	X	RP11-305F18.1	Clone-based (Vega) gene	
rs1995434				
rs1502834				
rs141133469				
rs3006131				
rs62594418				
rs5973046				
rs73199243				
rs149144154				
rs34213377				
rs73199280				
rs3006161				
rs3006092				
rs3105698				
rs141431271				
rs2940000				
rs3006129				
rs4829144				
rs5971830	X	RP11-545D19.1	Clone-based (Vega) gene	
rs12557344	X	RP11-545D19.1	Clone-based (Vega) gene	
rs62588191	X	RP11-545D19.1	Clone-based (Vega) gene	

SNP	Chromosome	gene name	gene source	description
rs11095329	X	RP11-545D19.1	Clone-based (Vega) gene	
rs17309863	X	RP11-545D19.1	Clone-based (Vega) gene	
rs5928503	X	RP11-545D19.1	Clone-based (Vega) gene	
rs138401839	X	RP11-545D19.1	Clone-based (Vega) gene	
rs41515051	X	RP11-545D19.1	Clone-based (Vega) gene	
rs4335292	X	RP11-545D19.1	Clone-based (Vega) gene	
rs4829334	X	RP11-545D19.1	Clone-based (Vega) gene	
rs5928536	X	RP11-545D19.1	Clone-based (Vega) gene	
rs17318613	X	RP11-545D19.1	Clone-based (Vega) gene	
rs111683988	X	RP11-545D19.1	Clone-based (Vega) gene	
rs12395785				
rs5928563				
rs4554633				
rs5973200				
rs59678444				
rs11095347				
rs7055554				
rs145556607				
rs5927282				
rs6631992				
rs4610907				
rs4276835	X	TMEM47	HGNC Symbol	transmembrane protein 47 [Source:HGNC Symbol;Acc:18515]
rs112512284	X	TMEM47	HGNC Symbol	transmembrane protein 47 [Source:HGNC Symbol;Acc:18515]
rs139937677	X	TMEM47	HGNC Symbol	transmembrane protein 47 [Source:HGNC Symbol;Acc:18515]
rs35200924				
rs73205497				
rs5928623				

SNP	Chromosome	gene name	gene source	description
rs73207305				
rs6527369				
rs73207316				
rs7065813				
rs12834922				
rs11796335				
rs5927329				
rs5927333				
rs6632098				
rs4424418				
rs11095368				
rs5928685				
rs59373047				
rs5928688				
rs73459571				
rs5928689				
rs6632121				
rs6527394				
rs7060411				
rs41305183	X	FAM47B	HGNC Symbol	family with sequence similarity 47, member B [Source:HGNC Symbol;Acc:26659]
rs147688579	X	FAM47B	HGNC Symbol	family with sequence similarity 47, member B [Source:HGNC Symbol;Acc:26659]
rs73189418				
rs3109519				
rs3128082				
rs4073063				
rs3124838				
rs3109864				
rs5927356				
rs3109861	X	RP11-504E21.1	Clone-based (Vega) gene	
rs12396884				
rs73189436				
rs142628433				

SNP	Chromosome	gene name	gene source	description
rs150433532				
rs876377				
rs73189443				
rs4145189				
rs5928786				
rs12850228				
rs186789903				
rs6632226				
rs1837895				
rs150920582				
rs149593124				
rs150121548				
rs5928838				
rs5927399				
rs2335396				
rs55939780				
rs5927408				
rs5973375				
rs73193318				
rs146577751				
rs5928903				
rs5927425				
rs143252239				
rs4829387				
rs73193353				
rs5928949				
rs141743776				
rs16992015	X	RP11-497J7.1	Clone-based (Vega) gene	
rs12837065				
rs2335996	X	RP11-497J7.2	Clone-based (Vega) gene	
rs73461109				
rs1410962	X	MAGEB16	HGNC Symbol	melanoma antigen family B, 16 [Source:HGNC Symbol;Acc:21188]

SNP	Chromosome	gene name	gene source	description
rs5973488	X	MAGEB16	HGNC Symbol	melanoma antigen family B, 16 [Source:HGNC Symbol;Acc:21188]
rs4829390	X	MAGEB16	HGNC Symbol	melanoma antigen family B, 16 [Source:HGNC Symbol;Acc:21188]
rs4829392	X	MAGEB16	HGNC Symbol	melanoma antigen family B, 16 [Source:HGNC Symbol;Acc:21188]
rs148913743				
rs5928991				
rs17245384				
rs1536848				
rs73195135				
rs6628999				
rs6629003				
rs2336402				
rs5973517				
rs6632376				
rs55725231				
rs28716264				
rs12391979				
rs5972007				
rs6629019	X	CXorf22	HGNC Symbol	chromosome X open reading frame 22 [Source:HGNC Symbol;Acc:28546]
rs6527521	X	CXorf22	HGNC Symbol	chromosome X open reading frame 22 [Source:HGNC Symbol;Acc:28546]
rs11798799	X	CXorf22	HGNC Symbol	chromosome X open reading frame 22 [Source:HGNC Symbol;Acc:28546]
rs2336029	X	CXorf22	HGNC Symbol	chromosome X open reading frame 22 [Source:HGNC Symbol;Acc:28546]
rs6629027	X	CXorf22	HGNC Symbol	chromosome X open reading frame 22 [Source:HGNC Symbol;Acc:28546]
rs6527531				
rs7886937	X	CHDC2	HGNC Symbol	calponin homology domain containing 2 [Source:HGNC Symbol;Acc:26708]
rs139211446	X	CHDC2	HGNC Symbol	calponin homology domain containing 2 [Source:HGNC Symbol;Acc:26708]
rs6632476	X	CHDC2	HGNC Symbol	calponin homology domain containing 2 [Source:HGNC Symbol;Acc:26708]
rs12855538	X	CHDC2	HGNC Symbol	calponin homology domain containing 2 [Source:HGNC Symbol;Acc:26708]
rs58938201	X	CHDC2	HGNC Symbol	calponin homology domain containing 2 [Source:HGNC Symbol;Acc:26708]
rs6629048	X	CHDC2	HGNC Symbol	calponin homology domain containing 2 [Source:HGNC Symbol;Acc:26708]
rs73197155				
rs2336658				
rs5927472				
rs56165720				

SNP	Chromosome	gene name	gene source	description
rs5973618	X;HG1423_PATCH	CXorf30	HGNC Symbol	chromosome X open reading frame 30 [Source:HGNC Symbol;Acc:27298]
rs7879188	X;HG1423_PATCH	CXorf30	HGNC Symbol	chromosome X open reading frame 30 [Source:HGNC Symbol;Acc:27298]
rs10522038	X;HG1423_PATCH	CXorf30	HGNC Symbol	chromosome X open reading frame 30 [Source:HGNC Symbol;Acc:27298]
rs151044769	X;HG1423_PATCH	CXorf30	HGNC Symbol	chromosome X open reading frame 30 [Source:HGNC Symbol;Acc:27298]
rs6629071	X;HG1423_PATCH	CXorf30	HGNC Symbol	chromosome X open reading frame 30 [Source:HGNC Symbol;Acc:27298]
rs6632561	X;HG1423_PATCH	CXorf30	HGNC Symbol	chromosome X open reading frame 30 [Source:HGNC Symbol;Acc:27298]
rs4641222	X;HG1423_PATCH	CXorf30	HGNC Symbol	chromosome X open reading frame 30 [Source:HGNC Symbol;Acc:27298]
rs55805036	X;HG1423_PATCH	CXorf30	HGNC Symbol	chromosome X open reading frame 30 [Source:HGNC Symbol;Acc:27298]
rs17319948	X;HG1423_PATCH	CXorf30	HGNC Symbol	chromosome X open reading frame 30 [Source:HGNC Symbol;Acc:27298]
rs41309695	X;HG1423_PATCH	CXorf30	HGNC Symbol	chromosome X open reading frame 30 [Source:HGNC Symbol;Acc:27298]
rs7053071	X;HG1423_PATCH	CXorf30	HGNC Symbol	chromosome X open reading frame 30 [Source:HGNC Symbol;Acc:27298]
rs6527569	X;HG1423_PATCH	CXorf30;RP11-87M18.2	HGNC Symbol;Clone-based (Vega) gene	chromosome X open reading frame 30 [Source:HGNC Symbol;Acc:27298];
rs5973654	X	RP11-87M18.2	Clone-based (Vega) gene	
rs5973655	X;HG1423_PATCH	RP11-87M18.2	Clone-based (Vega) gene	
rs146419966	X	RP11-87M18.2	Clone-based (Vega) gene	
rs138358929	X	RP11-87M18.2	Clone-based (Vega) gene	
rs5927479				
rs17245760				
rs4382629				
rs4829405				
rs4370685				
rs58553415				

SNP	Chromosome	gene name	gene source	description
rs5973681				
rs5972057				
rs17273427				
rs12011121				
rs7879062				
rs73203136				
rs36009382				
rs190370604				
rs5929050				
rs6653977				
rs111584965				
rs6629093				
rs62587863				
rs16987657				
rs73203147				
rs138230754				
rs142442507				
rs28566743				
rs138611849				
rs28698275				
rs142631322				
rs1008390				
rs28701851				
rs2024317				
rs62588461				
rs28488460				
rs73203167				
rs143535731				
rs28658472				
rs28547043				
rs56394021				
rs28556996				
rs28619196				

SNP	Chromosome	gene name	gene source	description
rs1995914	X;HG1423_PATCH	FAM47C	HGNC Symbol	family with sequence similarity 47, member C [Source:HGNC Symbol;Acc:25301]
rs28438823				
rs73203186				
rs73203188				
rs5917252				
rs73464117				
rs146200678				
rs73203192	X;HG1424_PATCH	PRRG1;TM4SF2	HGNC Symbol;UniProtKB Gene Name	proline rich Gla (G-carboxyglutamic acid) 1 [Source:HGNC Symbol;Acc:9469];Uncharacterized protein; cDNA FLJ59144, highly similar to Tetraspanin-7 [Source:UniProtKB/TrEMBL;Acc:B4E171]
rs56911976	X;HG1424_PATCH	PRRG1;TM4SF2	HGNC Symbol;UniProtKB Gene Name	proline rich Gla (G-carboxyglutamic acid) 1 [Source:HGNC Symbol;Acc:9469];Uncharacterized protein; cDNA FLJ59144, highly similar to Tetraspanin-7 [Source:UniProtKB/TrEMBL;Acc:B4E171]
rs5917507	X;HG1424_PATCH	PRRG1;TM4SF2	HGNC Symbol;UniProtKB Gene Name	proline rich Gla (G-carboxyglutamic acid) 1 [Source:HGNC Symbol;Acc:9469];Uncharacterized protein; cDNA FLJ59144, highly similar to Tetraspanin-7 [Source:UniProtKB/TrEMBL;Acc:B4E171]
rs1572620	X;HG1424_PATCH	PRRG1;TM4SF2	HGNC Symbol;UniProtKB Gene Name	proline rich Gla (G-carboxyglutamic acid) 1 [Source:HGNC Symbol;Acc:9469];Uncharacterized protein; cDNA FLJ59144, highly similar to Tetraspanin-7 [Source:UniProtKB/TrEMBL;Acc:B4E171]
rs17246106	X;HG1424_PATCH	PRRG1;TM4SF2	HGNC Symbol;UniProtKB Gene Name	proline rich Gla (G-carboxyglutamic acid) 1 [Source:HGNC Symbol;Acc:9469];Uncharacterized protein; cDNA FLJ59144, highly similar to Tetraspanin-7 [Source:UniProtKB/TrEMBL;Acc:B4E171]
rs12841220	X;HG1424_PATCH	PRRG1;TM4SF2	HGNC Symbol;UniProtKB Gene Name	proline rich Gla (G-carboxyglutamic acid) 1 [Source:HGNC Symbol;Acc:9469];Uncharacterized protein; cDNA FLJ59144, highly similar to Tetraspanin-7 [Source:UniProtKB/TrEMBL;Acc:B4E171]
rs3813166	X;HG1424_PATCH	PRRG1;TM4SF2	HGNC Symbol;UniProtKB Gene Name	proline rich Gla (G-carboxyglutamic acid) 1 [Source:HGNC Symbol;Acc:9469];Uncharacterized protein; cDNA FLJ59144, highly similar to Tetraspanin-7 [Source:UniProtKB/TrEMBL;Acc:B4E171]
rs5963574	X;HG1424_PATCH	TM4SF2	UniProtKB Gene Name	Uncharacterized protein; cDNA FLJ59144, highly similar to Tetraspanin-7 [Source:UniProtKB/TrEMBL;Acc:B4E171]
rs7877847	X	FAM47DP;TM4SF2	HGNC Symbol;UniProtKB Gene Name	family with sequence similarity 47, member D, pseudogene [Source:HGNC Symbol;Acc:34342];Uncharacterized protein; cDNA FLJ59144, highly similar to Tetraspanin-7 [Source:UniProtKB/TrEMBL;Acc:B4E171]
rs11796094	X;HG1424_PATCH	LANCL3;TM4SF2	HGNC Symbol;UniProtKB Gene Name	LanC lantibiotic synthetase component C-like 3 (bacterial) [Source:HGNC Symbol;Acc:24767];Uncharacterized protein; cDNA FLJ59144, highly similar to Tetraspanin-7 [Source:UniProtKB/TrEMBL;Acc:B4E171]

SNP	Chromosome	gene name	gene source	description
rs5963783	X;HG1424_PATCH	LANCL3;TM4SF2	HGNC Symbol;UniProtKB Gene Name	LanC lantibiotic synthetase component C-like 3 (bacterial) [Source:HGNC Symbol;Acc:24767];Uncharacterized protein; cDNA FLJ59144, highly similar to Tetraspanin-7 [Source:UniProtKB/TrEMBL;Acc:B4E171]
rs5963786	X;HG1424_PATCH	LANCL3;TM4SF2	HGNC Symbol;UniProtKB Gene Name	LanC lantibiotic synthetase component C-like 3 (bacterial) [Source:HGNC Symbol;Acc:24767];Uncharacterized protein; cDNA FLJ59144, highly similar to Tetraspanin-7 [Source:UniProtKB/TrEMBL;Acc:B4E171]
rs17145926	X;HG1424_PATCH	LANCL3;TM4SF2	HGNC Symbol;UniProtKB Gene Name	LanC lantibiotic synthetase component C-like 3 (bacterial) [Source:HGNC Symbol;Acc:24767];Uncharacterized protein; cDNA FLJ59144, highly similar to Tetraspanin-7 [Source:UniProtKB/TrEMBL;Acc:B4E171]
rs141479531	X;HG1424_PATCH	LANCL3;TM4SF2	HGNC Symbol;UniProtKB Gene Name	LanC lantibiotic synthetase component C-like 3 (bacterial) [Source:HGNC Symbol;Acc:24767];Uncharacterized protein; cDNA FLJ59144, highly similar to Tetraspanin-7 [Source:UniProtKB/TrEMBL;Acc:B4E171]
rs12009345	X;HG1424_PATCH	LANCL3;TM4SF2	HGNC Symbol;UniProtKB Gene Name	LanC lantibiotic synthetase component C-like 3 (bacterial) [Source:HGNC Symbol;Acc:24767];Uncharacterized protein; cDNA FLJ59144, highly similar to Tetraspanin-7 [Source:UniProtKB/TrEMBL;Acc:B4E171]
rs151330098	X;HG1424_PATCH	LANCL3;TM4SF2	HGNC Symbol;UniProtKB Gene Name	LanC lantibiotic synthetase component C-like 3 (bacterial) [Source:HGNC Symbol;Acc:24767];Uncharacterized protein; cDNA FLJ59144, highly similar to Tetraspanin-7 [Source:UniProtKB/TrEMBL;Acc:B4E171]
rs28503960	X;HG1424_PATCH	LANCL3;TM4SF2	HGNC Symbol;UniProtKB Gene Name	LanC lantibiotic synthetase component C-like 3 (bacterial) [Source:HGNC Symbol;Acc:24767];Uncharacterized protein; cDNA FLJ59144, highly similar to Tetraspanin-7 [Source:UniProtKB/TrEMBL;Acc:B4E171]
rs2295447	X;HG1424_PATCH	XK;TM4SF2	HGNC Symbol;UniProtKB Gene Name	X-linked Kx blood group (McLeod syndrome) [Source:HGNC Symbol;Acc:12811];Uncharacterized protein; cDNA FLJ59144, highly similar to Tetraspanin-7 [Source:UniProtKB/TrEMBL;Acc:B4E171]
rs28940602	X;HG1424_PATCH	XK;TM4SF2	HGNC Symbol;UniProtKB Gene Name	X-linked Kx blood group (McLeod syndrome) [Source:HGNC Symbol;Acc:12811];Uncharacterized protein; cDNA FLJ59144, highly similar to Tetraspanin-7 [Source:UniProtKB/TrEMBL;Acc:B4E171]
rs35184915	X;HG1424_PATCH	TM4SF2	UniProtKB Gene Name	Uncharacterized protein; cDNA FLJ59144, highly similar to Tetraspanin-7 [Source:UniProtKB/TrEMBL;Acc:B4E171]
rs7059081	X;HG1424_PATCH	CYBB;TM4SF2	HGNC Symbol;UniProtKB Gene Name	cytochrome b-245, beta polypeptide [Source:HGNC Symbol;Acc:2578];Uncharacterized protein; cDNA FLJ59144, highly similar to Tetraspanin-7 [Source:UniProtKB/TrEMBL;Acc:B4E171]
rs5917471	X;HG1424_PATCH	CYBB;TM4SF2	HGNC Symbol;UniProtKB Gene Name	cytochrome b-245, beta polypeptide [Source:HGNC Symbol;Acc:2578];Uncharacterized protein; cDNA FLJ59144, highly similar to Tetraspanin-7 [Source:UniProtKB/TrEMBL;Acc:B4E171]
rs34097325	X;HG1424_PATCH	CYBB;TM4SF2	HGNC Symbol;UniProtKB Gene Name	cytochrome b-245, beta polypeptide [Source:HGNC Symbol;Acc:2578];Uncharacterized protein; cDNA FLJ59144, highly similar to Tetraspanin-7 [Source:UniProtKB/TrEMBL;Acc:B4E171]
rs34697490	X;HG1424_PATCH	CYBB;TM4SF2	HGNC Symbol;UniProtKB Gene Name	cytochrome b-245, beta polypeptide [Source:HGNC Symbol;Acc:2578];Uncharacterized protein; cDNA FLJ59144, highly similar to Tetraspanin-7 [Source:UniProtKB/TrEMBL;Acc:B4E171]

SNP	Chromosome	gene name	gene source	description
rs5964151	X	CYBB;TM4SF2	HGNC Symbol;UniProtKB Gene Name	cytochrome b-245, beta polypeptide [Source:HGNC Symbol;Acc:2578];Uncharacterized protein; cDNA FLJ59144, highly similar to Tetraspanin-7 [Source:UniProtKB/TrEMBL;Acc:B4E171]
rs5963339	X	TM4SF2	UniProtKB Gene Name	Uncharacterized protein; cDNA FLJ59144, highly similar to Tetraspanin-7 [Source:UniProtKB/TrEMBL;Acc:B4E171]
rs7060979	X	TM4SF2	UniProtKB Gene Name	Uncharacterized protein; cDNA FLJ59144, highly similar to Tetraspanin-7 [Source:UniProtKB/TrEMBL;Acc:B4E171]
rs4506367	X	DYNLT3;TM4SF2	HGNC Symbol;UniProtKB Gene Name	dynein, light chain, Tctex-type 3 [Source:HGNC Symbol;Acc:11694];Uncharacterized protein; cDNA FLJ59144, highly similar to Tetraspanin-7 [Source:UniProtKB/TrEMBL;Acc:B4E171]
rs60939991	X	TM4SF2	UniProtKB Gene Name	Uncharacterized protein; cDNA FLJ59144, highly similar to Tetraspanin-7 [Source:UniProtKB/TrEMBL;Acc:B4E171]
rs67875193	X	TM4SF2	UniProtKB Gene Name	Uncharacterized protein; cDNA FLJ59144, highly similar to Tetraspanin-7 [Source:UniProtKB/TrEMBL;Acc:B4E171]
rs12009709	X	TM4SF2	UniProtKB Gene Name	Uncharacterized protein; cDNA FLJ59144, highly similar to Tetraspanin-7 [Source:UniProtKB/TrEMBL;Acc:B4E171]
rs7879191	X	TM4SF2	UniProtKB Gene Name	Uncharacterized protein; cDNA FLJ59144, highly similar to Tetraspanin-7 [Source:UniProtKB/TrEMBL;Acc:B4E171]
rs17246442	X	TM4SF2;AL121578.2	UniProtKB Gene Name;Clone-based (Vega) gene	Uncharacterized protein; cDNA FLJ59144, highly similar to Tetraspanin-7 [Source:UniProtKB/TrEMBL;Acc:B4E171];
rs993441	X	TM4SF2;AL121578.2	UniProtKB Gene Name;Clone-based (Vega) gene	Uncharacterized protein; cDNA FLJ59144, highly similar to Tetraspanin-7 [Source:UniProtKB/TrEMBL;Acc:B4E171];
rs5918419	X	TM4SF2;AL121578.2	UniProtKB Gene Name;Clone-based (Vega) gene	Uncharacterized protein; cDNA FLJ59144, highly similar to Tetraspanin-7 [Source:UniProtKB/TrEMBL;Acc:B4E171];
rs5964258	X	TM4SF2;AL121578.2	UniProtKB Gene Name;Clone-based (Vega) gene	Uncharacterized protein; cDNA FLJ59144, highly similar to Tetraspanin-7 [Source:UniProtKB/TrEMBL;Acc:B4E171];
rs5917509	X	TM4SF2;AL121578.2	UniProtKB Gene Name;Clone-based (Vega) gene	Uncharacterized protein; cDNA FLJ59144, highly similar to Tetraspanin-7 [Source:UniProtKB/TrEMBL;Acc:B4E171];
rs17146736	X	AL121578.5;TM4SF2;AL121578.2	Clone-based (Vega) gene;UniProtKB Gene Name	;Uncharacterized protein; cDNA FLJ59144, highly similar to Tetraspanin-7 [Source:UniProtKB/TrEMBL;Acc:B4E171]
rs5964261	X	TM4SF2;AL121578.2	UniProtKB Gene Name;Clone-based (Vega) gene	Uncharacterized protein; cDNA FLJ59144, highly similar to Tetraspanin-7 [Source:UniProtKB/TrEMBL;Acc:B4E171];

SNP	Chromosome	gene name	gene source	description
rs5918423	X	TM4SF2	UniProtKB Gene Name	Uncharacterized protein; cDNA FLJ59144, highly similar to Tetraspanin-7 [Source:UniProtKB/TrEMBL;Acc:B4E171]
rs5917518	X	TM4SF2	UniProtKB Gene Name	Uncharacterized protein; cDNA FLJ59144, highly similar to Tetraspanin-7 [Source:UniProtKB/TrEMBL;Acc:B4E171]
rs28718423	X	TM4SF2	UniProtKB Gene Name	Uncharacterized protein; cDNA FLJ59144, highly similar to Tetraspanin-7 [Source:UniProtKB/TrEMBL;Acc:B4E171]
rs148416225	X	TM4SF2	UniProtKB Gene Name	Uncharacterized protein; cDNA FLJ59144, highly similar to Tetraspanin-7 [Source:UniProtKB/TrEMBL;Acc:B4E171]
rs5964274	X	TM4SF2	UniProtKB Gene Name	Uncharacterized protein; cDNA FLJ59144, highly similar to Tetraspanin-7 [Source:UniProtKB/TrEMBL;Acc:B4E171]
rs3848892	X	TM4SF2	UniProtKB Gene Name	Uncharacterized protein; cDNA FLJ59144, highly similar to Tetraspanin-7 [Source:UniProtKB/TrEMBL;Acc:B4E171]
rs2093031	X	TM4SF2	UniProtKB Gene Name	Uncharacterized protein; cDNA FLJ59144, highly similar to Tetraspanin-7 [Source:UniProtKB/TrEMBL;Acc:B4E171]
rs73204989	X	SYTL5;TM4SF2	HGNC Symbol;UniProtKB Gene Name	synaptotagmin-like 5 [Source:HGNC Symbol;Acc:15589];Uncharacterized protein; cDNA FLJ59144, highly similar to Tetraspanin-7 [Source:UniProtKB/TrEMBL;Acc:B4E171]
rs148317143	X	SYTL5;TM4SF2	HGNC Symbol;UniProtKB Gene Name	synaptotagmin-like 5 [Source:HGNC Symbol;Acc:15589];Uncharacterized protein; cDNA FLJ59144, highly similar to Tetraspanin-7 [Source:UniProtKB/TrEMBL;Acc:B4E171]
rs7881956	X	SYTL5;TM4SF2	HGNC Symbol;UniProtKB Gene Name	synaptotagmin-like 5 [Source:HGNC Symbol;Acc:15589];Uncharacterized protein; cDNA FLJ59144, highly similar to Tetraspanin-7 [Source:UniProtKB/TrEMBL;Acc:B4E171]
rs73632440	X	SYTL5;TM4SF2	HGNC Symbol;UniProtKB Gene Name	synaptotagmin-like 5 [Source:HGNC Symbol;Acc:15589];Uncharacterized protein; cDNA FLJ59144, highly similar to Tetraspanin-7 [Source:UniProtKB/TrEMBL;Acc:B4E171]
rs1006517	X	SYTL5;TM4SF2	HGNC Symbol;UniProtKB Gene Name	synaptotagmin-like 5 [Source:HGNC Symbol;Acc:15589];Uncharacterized protein; cDNA FLJ59144, highly similar to Tetraspanin-7 [Source:UniProtKB/TrEMBL;Acc:B4E171]
rs991502	X	SYTL5;TM4SF2	HGNC Symbol;UniProtKB Gene Name	synaptotagmin-like 5 [Source:HGNC Symbol;Acc:15589];Uncharacterized protein; cDNA FLJ59144, highly similar to Tetraspanin-7 [Source:UniProtKB/TrEMBL;Acc:B4E171]
rs4827331	X	SYTL5;TM4SF2	HGNC Symbol;UniProtKB Gene Name	synaptotagmin-like 5 [Source:HGNC Symbol;Acc:15589];Uncharacterized protein; cDNA FLJ59144, highly similar to Tetraspanin-7 [Source:UniProtKB/TrEMBL;Acc:B4E171]
rs57226394	X	SYTL5;TM4SF2	HGNC Symbol;UniProtKB Gene Name	synaptotagmin-like 5 [Source:HGNC Symbol;Acc:15589];Uncharacterized protein; cDNA FLJ59144, highly similar to Tetraspanin-7 [Source:UniProtKB/TrEMBL;Acc:B4E171]

SNP	Chromosome	gene name	gene source	description
rs5918476	X	SYTL5;TM4SF2	HGNC Symbol;UniProtKB Gene Name	synaptotagmin-like 5 [Source:HGNC Symbol;Acc:15589];Uncharacterized protein; cDNA FLJ59144, highly similar to Tetraspanin-7 [Source:UniProtKB/TrEMBL;Acc:B4E171]
rs4827335	X	TM4SF2	UniProtKB Gene Name	Uncharacterized protein; cDNA FLJ59144, highly similar to Tetraspanin-7 [Source:UniProtKB/TrEMBL;Acc:B4E171]
rs146406003	X	TM4SF2	UniProtKB Gene Name	Uncharacterized protein; cDNA FLJ59144, highly similar to Tetraspanin-7 [Source:UniProtKB/TrEMBL;Acc:B4E171]
rs141547570	X	TM4SF2	UniProtKB Gene Name	Uncharacterized protein; cDNA FLJ59144, highly similar to Tetraspanin-7 [Source:UniProtKB/TrEMBL;Acc:B4E171]
rs35318931	X	SRPX;TM4SF2	HGNC Symbol;UniProtKB Gene Name	sushi-repeat containing protein, X-linked [Source:HGNC Symbol;Acc:11309];Uncharacterized protein; cDNA FLJ59144, highly similar to Tetraspanin-7 [Source:UniProtKB/TrEMBL;Acc:B4E171]
rs1123773	X	SRPX;TM4SF2	HGNC Symbol;UniProtKB Gene Name	sushi-repeat containing protein, X-linked [Source:HGNC Symbol;Acc:11309];Uncharacterized protein; cDNA FLJ59144, highly similar to Tetraspanin-7 [Source:UniProtKB/TrEMBL;Acc:B4E171]
rs743151	X	SRPX;TM4SF2	HGNC Symbol;UniProtKB Gene Name	sushi-repeat containing protein, X-linked [Source:HGNC Symbol;Acc:11309];Uncharacterized protein; cDNA FLJ59144, highly similar to Tetraspanin-7 [Source:UniProtKB/TrEMBL;Acc:B4E171]
rs62587006	X	SRPX;TM4SF2	HGNC Symbol;UniProtKB Gene Name	sushi-repeat containing protein, X-linked [Source:HGNC Symbol;Acc:11309];Uncharacterized protein; cDNA FLJ59144, highly similar to Tetraspanin-7 [Source:UniProtKB/TrEMBL;Acc:B4E171]
rs17246673	X	SRPX;TM4SF2	HGNC Symbol;UniProtKB Gene Name	sushi-repeat containing protein, X-linked [Source:HGNC Symbol;Acc:11309];Uncharacterized protein; cDNA FLJ59144, highly similar to Tetraspanin-7 [Source:UniProtKB/TrEMBL;Acc:B4E171]
rs5917549	X	SRPX;TM4SF2	HGNC Symbol;UniProtKB Gene Name	sushi-repeat containing protein, X-linked [Source:HGNC Symbol;Acc:11309];Uncharacterized protein; cDNA FLJ59144, highly similar to Tetraspanin-7 [Source:UniProtKB/TrEMBL;Acc:B4E171]
rs7883680	X	SRPX;TM4SF2	HGNC Symbol;UniProtKB Gene Name	sushi-repeat containing protein, X-linked [Source:HGNC Symbol;Acc:11309];Uncharacterized protein; cDNA FLJ59144, highly similar to Tetraspanin-7 [Source:UniProtKB/TrEMBL;Acc:B4E171]
rs17312631	X	SRPX;TM4SF2	HGNC Symbol;UniProtKB Gene Name	sushi-repeat containing protein, X-linked [Source:HGNC Symbol;Acc:11309];Uncharacterized protein; cDNA FLJ59144, highly similar to Tetraspanin-7 [Source:UniProtKB/TrEMBL;Acc:B4E171]
rs5918503	X	SRPX;TM4SF2	HGNC Symbol;UniProtKB Gene Name	sushi-repeat containing protein, X-linked [Source:HGNC Symbol;Acc:11309];Uncharacterized protein; cDNA FLJ59144, highly similar to Tetraspanin-7 [Source:UniProtKB/TrEMBL;Acc:B4E171]
rs6521110	X	SRPX;TM4SF2	HGNC Symbol;UniProtKB Gene Name	sushi-repeat containing protein, X-linked [Source:HGNC Symbol;Acc:11309];Uncharacterized protein; cDNA FLJ59144, highly similar to Tetraspanin-7 [Source:UniProtKB/TrEMBL;Acc:B4E171]

SNP	Chromosome	gene name	gene source	description
rs34797252	X	SRPX;TM4SF2	HGNC Symbol;UniProtKB Gene Name	sushi-repeat containing protein, X-linked [Source:HGNC Symbol;Acc:11309];Uncharacterized protein; cDNA FLJ59144, highly similar to Tetraspanin-7 [Source:UniProtKB/TrEMBL;Acc:B4E171]
rs73632468	X	SRPX;TM4SF2	HGNC Symbol;UniProtKB Gene Name	sushi-repeat containing protein, X-linked [Source:HGNC Symbol;Acc:11309];Uncharacterized protein; cDNA FLJ59144, highly similar to Tetraspanin-7 [Source:UniProtKB/TrEMBL;Acc:B4E171]
rs7890064	X	TM4SF2	UniProtKB Gene Name	Uncharacterized protein; cDNA FLJ59144, highly similar to Tetraspanin-7 [Source:UniProtKB/TrEMBL;Acc:B4E171]
rs58807730	X	TM4SF2	UniProtKB Gene Name	Uncharacterized protein; cDNA FLJ59144, highly similar to Tetraspanin-7 [Source:UniProtKB/TrEMBL;Acc:B4E171]
rs4336751	X	TM4SF2	UniProtKB Gene Name	Uncharacterized protein; cDNA FLJ59144, highly similar to Tetraspanin-7 [Source:UniProtKB/TrEMBL;Acc:B4E171]
rs12688554	X	TM4SF2	UniProtKB Gene Name	Uncharacterized protein; cDNA FLJ59144, highly similar to Tetraspanin-7 [Source:UniProtKB/TrEMBL;Acc:B4E171]
rs11796615	X	TM4SF2	UniProtKB Gene Name	Uncharacterized protein; cDNA FLJ59144, highly similar to Tetraspanin-7 [Source:UniProtKB/TrEMBL;Acc:B4E171]
rs5918512	X	TM4SF2	UniProtKB Gene Name	Uncharacterized protein; cDNA FLJ59144, highly similar to Tetraspanin-7 [Source:UniProtKB/TrEMBL;Acc:B4E171]
rs10521410	X	TM4SF2	UniProtKB Gene Name	Uncharacterized protein; cDNA FLJ59144, highly similar to Tetraspanin-7 [Source:UniProtKB/TrEMBL;Acc:B4E171]
rs3021103	X	TM4SF2	UniProtKB Gene Name	Uncharacterized protein; cDNA FLJ59144, highly similar to Tetraspanin-7 [Source:UniProtKB/TrEMBL;Acc:B4E171]
rs72619446	X	TM4SF2	UniProtKB Gene Name	Uncharacterized protein; cDNA FLJ59144, highly similar to Tetraspanin-7 [Source:UniProtKB/TrEMBL;Acc:B4E171]
rs12688347	X	RPGR;TM4SF2	HGNC Symbol;UniProtKB Gene Name	retinitis pigmentosa GTPase regulator [Source:HGNC Symbol;Acc:10295];Uncharacterized protein; cDNA FLJ59144, highly similar to Tetraspanin-7 [Source:UniProtKB/TrEMBL;Acc:B4E171]
rs55711031	X	RPGR;TM4SF2	HGNC Symbol;UniProtKB Gene Name	retinitis pigmentosa GTPase regulator [Source:HGNC Symbol;Acc:10295];Uncharacterized protein; cDNA FLJ59144, highly similar to Tetraspanin-7 [Source:UniProtKB/TrEMBL;Acc:B4E171]
rs139467624	X	RPGR;TM4SF2	HGNC Symbol;UniProtKB Gene Name	retinitis pigmentosa GTPase regulator [Source:HGNC Symbol;Acc:10295];Uncharacterized protein; cDNA FLJ59144, highly similar to Tetraspanin-7 [Source:UniProtKB/TrEMBL;Acc:B4E171]
rs12688514	X	RPGR;TM4SF2	HGNC Symbol;UniProtKB Gene Name	retinitis pigmentosa GTPase regulator [Source:HGNC Symbol;Acc:10295];Uncharacterized protein; cDNA FLJ59144, highly similar to Tetraspanin-7 [Source:UniProtKB/TrEMBL;Acc:B4E171]
rs12687163	X	RPGR;TM4SF2	HGNC Symbol;UniProtKB Gene Name	retinitis pigmentosa GTPase regulator [Source:HGNC Symbol;Acc:10295];Uncharacterized protein; cDNA FLJ59144, highly similar to Tetraspanin-7 [Source:UniProtKB/TrEMBL;Acc:B4E171]

SNP	Chromosome	gene name	gene source	description
rs41303691	X	RPGR;TM4SF2	HGNC Symbol;UniProtKB Gene Name	retinitis pigmentosa GTPase regulator [Source:HGNC Symbol;Acc:10295];Uncharacterized protein; cDNA FLJ59144, highly similar to Tetraspanin-7 [Source:UniProtKB/TrEMBL;Acc:B4E171]
rs1801688	X	RPGR;TM4SF2	HGNC Symbol;UniProtKB Gene Name	retinitis pigmentosa GTPase regulator [Source:HGNC Symbol;Acc:10295];Uncharacterized protein; cDNA FLJ59144, highly similar to Tetraspanin-7 [Source:UniProtKB/TrEMBL;Acc:B4E171]
rs41312104	X	RPGR;TM4SF2	HGNC Symbol;UniProtKB Gene Name	retinitis pigmentosa GTPase regulator [Source:HGNC Symbol;Acc:10295];Uncharacterized protein; cDNA FLJ59144, highly similar to Tetraspanin-7 [Source:UniProtKB/TrEMBL;Acc:B4E171]
rs73192542	X	RPGR;TM4SF2	HGNC Symbol;UniProtKB Gene Name	retinitis pigmentosa GTPase regulator [Source:HGNC Symbol;Acc:10295];Uncharacterized protein; cDNA FLJ59144, highly similar to Tetraspanin-7 [Source:UniProtKB/TrEMBL;Acc:B4E171]
rs60410666	X	RPGR;TM4SF2	HGNC Symbol;UniProtKB Gene Name	retinitis pigmentosa GTPase regulator [Source:HGNC Symbol;Acc:10295];Uncharacterized protein; cDNA FLJ59144, highly similar to Tetraspanin-7 [Source:UniProtKB/TrEMBL;Acc:B4E171]
rs62635003	X	RPGR;TM4SF2	HGNC Symbol;UniProtKB Gene Name	retinitis pigmentosa GTPase regulator [Source:HGNC Symbol;Acc:10295];Uncharacterized protein; cDNA FLJ59144, highly similar to Tetraspanin-7 [Source:UniProtKB/TrEMBL;Acc:B4E171]
rs1801687	X	RPGR;TM4SF2	HGNC Symbol;UniProtKB Gene Name	retinitis pigmentosa GTPase regulator [Source:HGNC Symbol;Acc:10295];Uncharacterized protein; cDNA FLJ59144, highly similar to Tetraspanin-7 [Source:UniProtKB/TrEMBL;Acc:B4E171]
rs1801686	X	RPGR;TM4SF2	HGNC Symbol;UniProtKB Gene Name	retinitis pigmentosa GTPase regulator [Source:HGNC Symbol;Acc:10295];Uncharacterized protein; cDNA FLJ59144, highly similar to Tetraspanin-7 [Source:UniProtKB/TrEMBL;Acc:B4E171]
rs11266204	X	RPGR;TM4SF2	HGNC Symbol;UniProtKB Gene Name	retinitis pigmentosa GTPase regulator [Source:HGNC Symbol;Acc:10295];Uncharacterized protein; cDNA FLJ59144, highly similar to Tetraspanin-7 [Source:UniProtKB/TrEMBL;Acc:B4E171]
rs5963400	X	RPGR;TM4SF2	HGNC Symbol;UniProtKB Gene Name	retinitis pigmentosa GTPase regulator [Source:HGNC Symbol;Acc:10295];Uncharacterized protein; cDNA FLJ59144, highly similar to Tetraspanin-7 [Source:UniProtKB/TrEMBL;Acc:B4E171]
rs3810691	X	RPGR;TM4SF2	HGNC Symbol;UniProtKB Gene Name	retinitis pigmentosa GTPase regulator [Source:HGNC Symbol;Acc:10295];Uncharacterized protein; cDNA FLJ59144, highly similar to Tetraspanin-7 [Source:UniProtKB/TrEMBL;Acc:B4E171]
rs73192570	X	RPGR;TM4SF2	HGNC Symbol;UniProtKB Gene Name	retinitis pigmentosa GTPase regulator [Source:HGNC Symbol;Acc:10295];Uncharacterized protein; cDNA FLJ59144, highly similar to Tetraspanin-7 [Source:UniProtKB/TrEMBL;Acc:B4E171]
rs73192571	X	RPGR;TM4SF2	HGNC Symbol;UniProtKB Gene Name	retinitis pigmentosa GTPase regulator [Source:HGNC Symbol;Acc:10295];Uncharacterized protein; cDNA FLJ59144, highly similar to Tetraspanin-7 [Source:UniProtKB/TrEMBL;Acc:B4E171]

SNP	Chromosome	gene name	gene source	description
rs111631988	X	RPGR;TM4SF2	HGNC Symbol;UniProtKB Gene Name	retinitis pigmentosa GTPase regulator [Source:HGNC Symbol;Acc:10295];Uncharacterized protein; cDNA FLJ59144, highly similar to Tetraspanin-7 [Source:UniProtKB/TrEMBL;Acc:B4E171]
rs147711382	X	RPGR;TM4SF2	HGNC Symbol;UniProtKB Gene Name	retinitis pigmentosa GTPase regulator [Source:HGNC Symbol;Acc:10295];Uncharacterized protein; cDNA FLJ59144, highly similar to Tetraspanin-7 [Source:UniProtKB/TrEMBL;Acc:B4E171]
rs12853995	X	TM4SF2	UniProtKB Gene Name	Uncharacterized protein; cDNA FLJ59144, highly similar to Tetraspanin-7 [Source:UniProtKB/TrEMBL;Acc:B4E171]
rs12845367	X	TM4SF2	UniProtKB Gene Name	Uncharacterized protein; cDNA FLJ59144, highly similar to Tetraspanin-7 [Source:UniProtKB/TrEMBL;Acc:B4E171]
rs6609578	X	TM4SF2	UniProtKB Gene Name	Uncharacterized protein; cDNA FLJ59144, highly similar to Tetraspanin-7 [Source:UniProtKB/TrEMBL;Acc:B4E171]
rs6609582	X	TM4SF2	UniProtKB Gene Name	Uncharacterized protein; cDNA FLJ59144, highly similar to Tetraspanin-7 [Source:UniProtKB/TrEMBL;Acc:B4E171]
rs17246924	X	OTC;TM4SF2	HGNC Symbol;UniProtKB Gene Name	ornithine carbamoyltransferase [Source:HGNC Symbol;Acc:8512];Uncharacterized protein; cDNA FLJ59144, highly similar to Tetraspanin-7 [Source:UniProtKB/TrEMBL;Acc:B4E171]
rs1800321	X	OTC;TM4SF2	HGNC Symbol;UniProtKB Gene Name	ornithine carbamoyltransferase [Source:HGNC Symbol;Acc:8512];Uncharacterized protein; cDNA FLJ59144, highly similar to Tetraspanin-7 [Source:UniProtKB/TrEMBL;Acc:B4E171]
rs7056414	X	OTC;TM4SF2	HGNC Symbol;UniProtKB Gene Name	ornithine carbamoyltransferase [Source:HGNC Symbol;Acc:8512];Uncharacterized protein; cDNA FLJ59144, highly similar to Tetraspanin-7 [Source:UniProtKB/TrEMBL;Acc:B4E171]
rs12854106	X	OTC;TM4SF2	HGNC Symbol;UniProtKB Gene Name	ornithine carbamoyltransferase [Source:HGNC Symbol;Acc:8512];Uncharacterized protein; cDNA FLJ59144, highly similar to Tetraspanin-7 [Source:UniProtKB/TrEMBL;Acc:B4E171]
rs41449246	X	OTC;TM4SF2	HGNC Symbol;UniProtKB Gene Name	ornithine carbamoyltransferase [Source:HGNC Symbol;Acc:8512];Uncharacterized protein; cDNA FLJ59144, highly similar to Tetraspanin-7 [Source:UniProtKB/TrEMBL;Acc:B4E171]
rs12557315	X	OTC;TM4SF2	HGNC Symbol;UniProtKB Gene Name	ornithine carbamoyltransferase [Source:HGNC Symbol;Acc:8512];Uncharacterized protein; cDNA FLJ59144, highly similar to Tetraspanin-7 [Source:UniProtKB/TrEMBL;Acc:B4E171]
rs12846992	X	TM4SF2	UniProtKB Gene Name	Uncharacterized protein; cDNA FLJ59144, highly similar to Tetraspanin-7 [Source:UniProtKB/TrEMBL;Acc:B4E171]
rs5963441	X	TM4SF2	UniProtKB Gene Name	Uncharacterized protein; cDNA FLJ59144, highly similar to Tetraspanin-7 [Source:UniProtKB/TrEMBL;Acc:B4E171]
rs143689118	X	TM4SF2	UniProtKB Gene Name	Uncharacterized protein; cDNA FLJ59144, highly similar to Tetraspanin-7 [Source:UniProtKB/TrEMBL;Acc:B4E171]
rs966492	X	TM4SF2	UniProtKB Gene Name	Uncharacterized protein; cDNA FLJ59144, highly similar to Tetraspanin-7 [Source:UniProtKB/TrEMBL;Acc:B4E171]

SNP	Chromosome	gene name	gene source	description
rs12855903	X	TM4SF2	UniProtKB Gene Name	Uncharacterized protein; cDNA FLJ59144, highly similar to Tetraspanin-7 [Source:UniProtKB/TrEMBL;Acc:B4E171]
rs5963038	X	TM4SF2	UniProtKB Gene Name	Uncharacterized protein; cDNA FLJ59144, highly similar to Tetraspanin-7 [Source:UniProtKB/TrEMBL;Acc:B4E171]
rs146052971	X	TM4SF2	UniProtKB Gene Name	Uncharacterized protein; cDNA FLJ59144, highly similar to Tetraspanin-7 [Source:UniProtKB/TrEMBL;Acc:B4E171]
rs35911641	X	TM4SF2	UniProtKB Gene Name	Uncharacterized protein; cDNA FLJ59144, highly similar to Tetraspanin-7 [Source:UniProtKB/TrEMBL;Acc:B4E171]
rs1022451	X	TM4SF2	UniProtKB Gene Name	Uncharacterized protein; cDNA FLJ59144, highly similar to Tetraspanin-7 [Source:UniProtKB/TrEMBL;Acc:B4E171]
rs5963487	X	AF241726.4;TM4SF2	Clone-based (Vega) gene;UniProtKB Gene Name	;Uncharacterized protein; cDNA FLJ59144, highly similar to Tetraspanin-7 [Source:UniProtKB/TrEMBL;Acc:B4E171]
rs4827105	X	TM4SF2	UniProtKB Gene Name	Uncharacterized protein; cDNA FLJ59144, highly similar to Tetraspanin-7 [Source:UniProtKB/TrEMBL;Acc:B4E171]
rs6610117	X	TM4SF2	UniProtKB Gene Name	Uncharacterized protein; cDNA FLJ59144, highly similar to Tetraspanin-7 [Source:UniProtKB/TrEMBL;Acc:B4E171]
rs12840299	X	TM4SF2	UniProtKB Gene Name	Uncharacterized protein; cDNA FLJ59144, highly similar to Tetraspanin-7 [Source:UniProtKB/TrEMBL;Acc:B4E171]
rs6520495	X	TM4SF2	UniProtKB Gene Name	Uncharacterized protein; cDNA FLJ59144, highly similar to Tetraspanin-7 [Source:UniProtKB/TrEMBL;Acc:B4E171]
rs34913508	X	TSPAN7;TM4SF2	HGNC Symbol;UniProtKB Gene Name	tetraspanin 7 [Source:HGNC Symbol;Acc:11854];Uncharacterized protein; cDNA FLJ59144, highly similar to Tetraspanin-7 [Source:UniProtKB/TrEMBL;Acc:B4E171]
rs7887127	X	TSPAN7;TM4SF2	HGNC Symbol;UniProtKB Gene Name	tetraspanin 7 [Source:HGNC Symbol;Acc:11854];Uncharacterized protein; cDNA FLJ59144, highly similar to Tetraspanin-7 [Source:UniProtKB/TrEMBL;Acc:B4E171]
rs62589248	X	TSPAN7;TM4SF2	HGNC Symbol;UniProtKB Gene Name	tetraspanin 7 [Source:HGNC Symbol;Acc:11854];Uncharacterized protein; cDNA FLJ59144, highly similar to Tetraspanin-7 [Source:UniProtKB/TrEMBL;Acc:B4E171]
rs58307409	X	TSPAN7;TM4SF2	HGNC Symbol;UniProtKB Gene Name	tetraspanin 7 [Source:HGNC Symbol;Acc:11854];Uncharacterized protein; cDNA FLJ59144, highly similar to Tetraspanin-7 [Source:UniProtKB/TrEMBL;Acc:B4E171]
rs56673674	X	TSPAN7;TM4SF2	HGNC Symbol;UniProtKB Gene Name	tetraspanin 7 [Source:HGNC Symbol;Acc:11854];Uncharacterized protein; cDNA FLJ59144, highly similar to Tetraspanin-7 [Source:UniProtKB/TrEMBL;Acc:B4E171]
rs4448427	X	TSPAN7;TM4SF2	HGNC Symbol;UniProtKB Gene Name	tetraspanin 7 [Source:HGNC Symbol;Acc:11854];Uncharacterized protein; cDNA FLJ59144, highly similar to Tetraspanin-7 [Source:UniProtKB/TrEMBL;Acc:B4E171]

SNP	Chromosome	gene name	gene source	description
rs5963523	X	TSPAN7;TM4SF2	HGNC Symbol;UniProtKB Gene Name	tetraspanin 7 [Source:HGNC Symbol;Acc:11854];Uncharacterized protein; cDNA FLJ59144, highly similar to Tetraspanin-7 [Source:UniProtKB/TrEMBL;Acc:B4E171]
rs73198496	X	TSPAN7;TM4SF2	HGNC Symbol;UniProtKB Gene Name	tetraspanin 7 [Source:HGNC Symbol;Acc:11854];Uncharacterized protein; cDNA FLJ59144, highly similar to Tetraspanin-7 [Source:UniProtKB/TrEMBL;Acc:B4E171]
rs12392962	X	TSPAN7;TM4SF2	HGNC Symbol;UniProtKB Gene Name	tetraspanin 7 [Source:HGNC Symbol;Acc:11854];Uncharacterized protein; cDNA FLJ59144, highly similar to Tetraspanin-7 [Source:UniProtKB/TrEMBL;Acc:B4E171]
rs10333	X	TSPAN7	HGNC Symbol	tetraspanin 7 [Source:HGNC Symbol;Acc:11854]
rs12839388				
rs11795872				
rs5917219				
rs970714				
rs144575916				
rs17313336				
rs73200485	X	RP4-646N3.1	Clone-based (Vega) gene	
rs198774	X	RP4-646N3.1	Clone-based (Vega) gene	
rs55963750	X	MID1IP1;MID1IP1-AS1	HGNC Symbol	MID1 interacting protein 1 [Source:HGNC Symbol;Acc:20715];MID1IP1 antisense RNA 1 [Source:HGNC Symbol;Acc:40932]
rs143008624	X	MID1IP1	HGNC Symbol	MID1 interacting protein 1 [Source:HGNC Symbol;Acc:20715]
rs198785				
rs73465507				
rs72623204				
rs10521415				
rs149340726				
rs138053843				
rs66653026				
rs199903				
rs12688830				
rs5917638				
rs199868				
rs199855				
rs199851				

SNP	Chromosome	gene name	gene source	description
rs73202406				
rs66466639				
rs56395632				
rs73202476				
rs73202482				
rs7891664				
rs12857099				
rs148899764				
rs12687852				
rs55690680				
rs4827118				
rs11796578				
rs73202488				
rs5917678				
rs139511904				
rs5917247				
rs5963577				
rs5963578				
rs909381				
rs5917702				
rs147441967				
rs111849103				
rs4827007				
rs146056122				
rs73205005				
rs5963595				
rs5917735				
rs11266241				
rs73205009				
rs73205013				
rs56827783				
rs5917744				
rs67285016				

SNP	Chromosome	gene name	gene source	description
rs17247674				
rs11266242				
rs5917279				
rs5917757				
rs140177545				
rs11266251				
rs6610244				
rs73205021	X	RP11-265P11.1	Clone-based (Vega) gene	
rs142062184	X	RP11-265P11.1	Clone-based (Vega) gene	
rs7880137	X	RP11-265P11.1	Clone-based (Vega) gene	
rs12840783	X	RP11-265P11.1	Clone-based (Vega) gene	
rs5963117	X	RP11-265P11.1	Clone-based (Vega) gene	
rs112906393	X	RP11-265P11.1	Clone-based (Vega) gene	
rs17313672	X	RP11-265P11.1	Clone-based (Vega) gene	
rs10126663	X	RP11-265P11.1	Clone-based (Vega) gene	
rs141255834	X	RP11-265P11.1	Clone-based (Vega) gene	
rs150604526	X	RP11-265P11.2	Clone-based (Vega) gene	
rs5917783	X	RP11-265P11.2	Clone-based (Vega) gene	
rs4827138	X	RP11-265P11.2	Clone-based (Vega) gene	
rs142492725	X	RP11-265P11.2	Clone-based (Vega) gene	
rs10521418	X	RP11-265P11.2	Clone-based (Vega) gene	
rs5917296	X	RP11-265P11.2	Clone-based (Vega) gene	
rs6610258				

SNP	Chromosome	gene name	gene source	description
rs12557180				
rs112451528				
rs62590129				
rs4827146	X	RP11-157D23.1	Clone-based (Vega) gene	
rs41298470	X	RP11-157D23.1	Clone-based (Vega) gene	
rs150910648	X	RP11-157D23.1	Clone-based (Vega) gene	
rs5917302	X	RP11-157D23.1	Clone-based (Vega) gene	
rs5917303				
rs55772618				
rs958398				
rs11796466	X	RP11-157D23.2	Clone-based (Vega) gene	
rs148349674	X	RP11-157D23.2	Clone-based (Vega) gene	
rs4827150	X	RP11-157D23.2	Clone-based (Vega) gene	
rs5917816				
rs6610271				
rs66651550				
rs17145393				
rs73206823				
rs5917820				
rs5963663				
rs17274776				
rs4589010				
rs17313880				
rs17313894				
rs12559272				
rs6610294				
rs66904200				
rs2317663				

SNP	Chromosome	gene name	gene source	description
rs5963671				
rs4827166				
rs206035				
rs4827018				
rs17313943				
rs206039				
rs138545851				
rs17274867				
rs73209122				
rs149719566				
rs207036				
rs138436947				
rs5963678				
rs584465				
rs150222				
rs4614139				
rs2919834				
rs2961423				
rs2919824				
rs12014400				
rs6610334				
rs2317890				
rs149400676				
rs2068740				
rs144783040				
rs62588984				
rs114454893				
rs5963691				
rs5963693				
rs4827023				
rs140911585	X	MIR1587	HGNC Symbol	microRNA 1587 [Source:HGNC Symbol;Acc:41596]
rs140302835	X	MIR1587	HGNC Symbol	microRNA 1587 [Source:HGNC Symbol;Acc:41596]
rs73191883	X	MIR1587	HGNC Symbol	microRNA 1587 [Source:HGNC Symbol;Acc:41596]

SNP	Chromosome	gene name	gene source	description
rs5963697	X	MIR1587	HGNC Symbol	microRNA 1587 [Source:HGNC Symbol;Acc:41596]
rs149603173				
rs3002414				
rs116708792				
rs10127068				
rs3008915				
rs73191888				
rs5917889				
rs12394720				
rs3002403				
rs3008926				
rs11795866				
rs5963701				
rs7885790				
rs5963706				
rs6651608				
rs3002397				
rs3008897				
rs3008899				
rs4146183				
rs12559823				
rs5963709				
rs12689787				
rs3002424				
rs141029539				
rs17314146				
rs17314153				
rs113001152				
rs5917335				
rs73465739				
rs12557637				
rs11266269				
rs5917905				

SNP	Chromosome	gene name	gene source	description
rs5917908				
rs4827030				
rs5917911				
rs73465762				
rs7054723				
rs12559699				
rs5917919				
rs17145638				
rs145158961				
rs5917922				
rs12689666				
rs7879155				
rs5917924				
rs5963722				
rs5963153	X	BCOR	HGNC Symbol	BCL6 corepressor [Source:HGNC Symbol;Acc:20893]
rs5963154	X	BCOR	HGNC Symbol	BCL6 corepressor [Source:HGNC Symbol;Acc:20893]
rs5963728	X	BCOR	HGNC Symbol	BCL6 corepressor [Source:HGNC Symbol;Acc:20893]
rs4630013	X	BCOR	HGNC Symbol	BCL6 corepressor [Source:HGNC Symbol;Acc:20893]
rs55700604	X	BCOR	HGNC Symbol	BCL6 corepressor [Source:HGNC Symbol;Acc:20893]
rs5963155	X	BCOR	HGNC Symbol	BCL6 corepressor [Source:HGNC Symbol;Acc:20893]
rs73194084	X	BCOR	HGNC Symbol	BCL6 corepressor [Source:HGNC Symbol;Acc:20893]
rs5917931	X	BCOR	HGNC Symbol	BCL6 corepressor [Source:HGNC Symbol;Acc:20893]
rs6520618	X	BCOR	HGNC Symbol	BCL6 corepressor [Source:HGNC Symbol;Acc:20893]
rs6610384	X	BCOR	HGNC Symbol	BCL6 corepressor [Source:HGNC Symbol;Acc:20893]
rs4076107	X	BCOR	HGNC Symbol	BCL6 corepressor [Source:HGNC Symbol;Acc:20893]
rs5963736	X	BCOR	HGNC Symbol	BCL6 corepressor [Source:HGNC Symbol;Acc:20893]
rs4827194	X	BCOR	HGNC Symbol	BCL6 corepressor [Source:HGNC Symbol;Acc:20893]
rs5963739	X	BCOR	HGNC Symbol	BCL6 corepressor [Source:HGNC Symbol;Acc:20893]
rs12841108	X	BCOR	HGNC Symbol	BCL6 corepressor [Source:HGNC Symbol;Acc:20893]
rs5963157	X	BCOR	HGNC Symbol	BCL6 corepressor [Source:HGNC Symbol;Acc:20893]
rs5963159	X	BCOR	HGNC Symbol	BCL6 corepressor [Source:HGNC Symbol;Acc:20893]
rs7063183	X	BCOR	HGNC Symbol	BCL6 corepressor [Source:HGNC Symbol;Acc:20893]
rs186754368	X	BCOR	HGNC Symbol	BCL6 corepressor [Source:HGNC Symbol;Acc:20893]

SNP	Chromosome	gene name	gene source	description
rs12859884	X	BCOR	HGNC Symbol	BCL6 corepressor [Source:HGNC Symbol;Acc:20893]
rs35811853	X	BCOR	HGNC Symbol	BCL6 corepressor [Source:HGNC Symbol;Acc:20893]
rs147830781	X	BCOR	HGNC Symbol	BCL6 corepressor [Source:HGNC Symbol;Acc:20893]
rs73194088				
rs56167307				
rs5963755				
rs5963756				
rs112986949				
rs5963764				
rs62585885				
rs55901985				
rs9785476				
rs3932236				
rs7055988				
rs4827201				
rs4827202				
rs113395359				
rs5963774	X	RP11-320G24.1	Clone-based (Vega) gene	
rs67816257	X	RP11-320G24.1	Clone-based (Vega) gene	
rs11266279	X	RP11-320G24.1	Clone-based (Vega) gene	
rs11796969	X	RP11-320G24.1	Clone-based (Vega) gene	
rs5963173				
rs869829				
rs5917964				
rs2961373				
rs73195815				
rs3008972				
rs2961405				
rs2961393				
rs7058033				

SNP	Chromosome	gene name	gene source	description
rs2961385				
rs2948463				
rs3008956				
rs2961402				
rs3008946				
rs72623232				
rs143083342				
rs73197912				
rs5917974				
rs59151655				
rs62584872				
rs17248626				
rs5917357				
rs2948495				
rs148812591				
rs112300017				
rs73197918				
rs73197921				
rs5917982				
rs7054029				
rs7066726				
rs7064994				
rs5917361				
rs5963181				
rs12392708	X	RP11-126D17.1	Clone-based (Vega) gene	
rs12687986				
rs4509477				
rs5917364				
rs5917990				
rs12557397				
rs6520655				
rs112797956				

SNP	Chromosome	gene name	gene source	description
rs142089149				
rs7061646				
rs6610420				
rs6609072				
rs61216765				
rs5918003				
rs4240044				
rs884563				
rs5918011				
rs150938106				
rs4323613				
rs56387342	X	CXorf38	HGNC Symbol	chromosome X open reading frame 38 [Source:HGNC Symbol;Acc:28589]
rs12558065	X	MED14	HGNC Symbol	mediator complex subunit 14 [Source:HGNC Symbol;Acc:2370]
rs5918029	X	MED14	HGNC Symbol	mediator complex subunit 14 [Source:HGNC Symbol;Acc:2370]
rs5917382	X	MED14	HGNC Symbol	mediator complex subunit 14 [Source:HGNC Symbol;Acc:2370]
rs6520683	X	MED14	HGNC Symbol	mediator complex subunit 14 [Source:HGNC Symbol;Acc:2370]
rs148191803	X	MED14	HGNC Symbol	mediator complex subunit 14 [Source:HGNC Symbol;Acc:2370]
rs4827229	X	MED14	HGNC Symbol	mediator complex subunit 14 [Source:HGNC Symbol;Acc:2370]
rs113770660				
rs67446705				
rs5963844				
rs6609094				
rs5917388				
rs112396107				
rs11795553				
rs140319336				
rs5918045				
rs57023905				
rs58102978				
rs7887906				
rs4827045				
rs138305671				
rs2168030				

SNP	Chromosome	gene name	gene source	description
rs73474824				
rs73201713				
rs7884878				
rs10127045				
rs35865200				
rs5963889				
rs4483318				
rs73476523				
rs61282543				
rs5917394				
rs5918068				
rs66512463				
rs5963904				
rs151183050				
rs4425907				
rs11266298				
rs6609114				
rs7062915				
rs5918084				
rs4827248				
rs1243791				
rs67697499				
rs1243779				
rs7060889				
rs6417860				
rs67917545				
rs1263915				
rs1150525				
rs35235436				
rs1937229	X	USP9X	HGNC Symbol	ubiquitin specific peptidase 9, X-linked [Source:HGNC Symbol;Acc:12632]
rs5918109	X	USP9X	HGNC Symbol	ubiquitin specific peptidase 9, X-linked [Source:HGNC Symbol;Acc:12632]
rs1150527	X	USP9X	HGNC Symbol	ubiquitin specific peptidase 9, X-linked [Source:HGNC Symbol;Acc:12632]
rs73203642	X	USP9X	HGNC Symbol	ubiquitin specific peptidase 9, X-linked [Source:HGNC Symbol;Acc:12632]

SNP	Chromosome	gene name	gene source	description
rs12689801	X	USP9X	HGNC Symbol	ubiquitin specific peptidase 9, X-linked [Source:HGNC Symbol;Acc:12632]
rs73203644	X	USP9X	HGNC Symbol	ubiquitin specific peptidase 9, X-linked [Source:HGNC Symbol;Acc:12632]
rs5918124	X	USP9X	HGNC Symbol	ubiquitin specific peptidase 9, X-linked [Source:HGNC Symbol;Acc:12632]
rs2302381	X	USP9X	HGNC Symbol	ubiquitin specific peptidase 9, X-linked [Source:HGNC Symbol;Acc:12632]
rs1150540	X	USP9X	HGNC Symbol	ubiquitin specific peptidase 9, X-linked [Source:HGNC Symbol;Acc:12632]
rs3788881	X	USP9X	HGNC Symbol	ubiquitin specific peptidase 9, X-linked [Source:HGNC Symbol;Acc:12632]
rs5918131	X	USP9X	HGNC Symbol	ubiquitin specific peptidase 9, X-linked [Source:HGNC Symbol;Acc:12632]
rs2284118	X	USP9X	HGNC Symbol	ubiquitin specific peptidase 9, X-linked [Source:HGNC Symbol;Acc:12632]
rs12857881	X	USP9X	HGNC Symbol	ubiquitin specific peptidase 9, X-linked [Source:HGNC Symbol;Acc:12632]
rs6520734	X	USP9X	HGNC Symbol	ubiquitin specific peptidase 9, X-linked [Source:HGNC Symbol;Acc:12632]
rs56033584	X	USP9X	HGNC Symbol	ubiquitin specific peptidase 9, X-linked [Source:HGNC Symbol;Acc:12632]
rs5963931	X	USP9X	HGNC Symbol	ubiquitin specific peptidase 9, X-linked [Source:HGNC Symbol;Acc:12632]
rs5917418	X	USP9X	HGNC Symbol	ubiquitin specific peptidase 9, X-linked [Source:HGNC Symbol;Acc:12632]
rs10463	X	USP9X	HGNC Symbol	ubiquitin specific peptidase 9, X-linked [Source:HGNC Symbol;Acc:12632]
rs41305227	X	USP9X	HGNC Symbol	ubiquitin specific peptidase 9, X-linked [Source:HGNC Symbol;Acc:12632]
rs113218539				
rs6609123				
rs1150537				
rs12390290				
rs6610530				
rs34956682				
rs5963946				
rs6610534				
rs147276859				
rs6610538				
rs6610541				
rs184347580	X	DDX3X	HGNC Symbol	DEAD (Asp-Glu-Ala-Asp) box helicase 3, X-linked [Source:HGNC Symbol;Acc:2745]
rs953114	X	DDX3X	HGNC Symbol	DEAD (Asp-Glu-Ala-Asp) box helicase 3, X-linked [Source:HGNC Symbol;Acc:2745]
rs12851342	X	DDX3X	HGNC Symbol	DEAD (Asp-Glu-Ala-Asp) box helicase 3, X-linked [Source:HGNC Symbol;Acc:2745]
rs56395484	X	DDX3X	HGNC Symbol	DEAD (Asp-Glu-Ala-Asp) box helicase 3, X-linked [Source:HGNC Symbol;Acc:2745]
rs2275944	X	DDX3X	HGNC Symbol	DEAD (Asp-Glu-Ala-Asp) box helicase 3, X-linked [Source:HGNC Symbol;Acc:2745]
rs72626411	X	DDX3X	HGNC Symbol	DEAD (Asp-Glu-Ala-Asp) box helicase 3, X-linked [Source:HGNC Symbol;Acc:2745]
rs72626414				

SNP	Chromosome	gene name	gene source	description
rs787092				
rs787089				
rs787084				
rs112780869				
rs1794648				
rs146173417				
rs12861886				
rs150255888				
rs5918155				
rs2056579				
rs2859013				
rs1794662				
rs2859018				
rs140733523				
rs3810732	X	NYX	HGNC Symbol	nyctalopin [Source:HGNC Symbol;Acc:8082]
rs3013116	X	NYX	HGNC Symbol	nyctalopin [Source:HGNC Symbol;Acc:8082]
rs2858997	X	NYX	HGNC Symbol	nyctalopin [Source:HGNC Symbol;Acc:8082]
rs873336	X	NYX	HGNC Symbol	nyctalopin [Source:HGNC Symbol;Acc:8082]
rs7051589				
rs2859006				
rs2807171				
rs5963996				
rs2859010				
rs5964004				
rs3203642	X	CASK	HGNC Symbol	calcium/calmodulin-dependent serine protein kinase (MAGUK family) [Source:HGNC Symbol;Acc:1497]
rs5918193	X	CASK	HGNC Symbol	calcium/calmodulin-dependent serine protein kinase (MAGUK family) [Source:HGNC Symbol;Acc:1497]
rs41310597	X	CASK	HGNC Symbol	calcium/calmodulin-dependent serine protein kinase (MAGUK family) [Source:HGNC Symbol;Acc:1497]
rs2998250	X	CASK	HGNC Symbol	calcium/calmodulin-dependent serine protein kinase (MAGUK family) [Source:HGNC Symbol;Acc:1497]
rs62587016	X	CASK	HGNC Symbol	calcium/calmodulin-dependent serine protein kinase (MAGUK family) [Source:HGNC Symbol;Acc:1497]

SNP	Chromosome	gene name	gene source	description
rs17315793	X	CASK	HGNC Symbol	calcium/calmodulin-dependent serine protein kinase (MAGUK family) [Source:HGNC Symbol;Acc:1497]
rs73193113	X	CASK	HGNC Symbol	calcium/calmodulin-dependent serine protein kinase (MAGUK family) [Source:HGNC Symbol;Acc:1497]
rs5918213	X	CASK	HGNC Symbol	calcium/calmodulin-dependent serine protein kinase (MAGUK family) [Source:HGNC Symbol;Acc:1497]
rs111732408	X	CASK	HGNC Symbol	calcium/calmodulin-dependent serine protein kinase (MAGUK family) [Source:HGNC Symbol;Acc:1497]
rs76068774	X	CASK	HGNC Symbol	calcium/calmodulin-dependent serine protein kinase (MAGUK family) [Source:HGNC Symbol;Acc:1497]
rs12851091	X	CASK	HGNC Symbol	calcium/calmodulin-dependent serine protein kinase (MAGUK family) [Source:HGNC Symbol;Acc:1497]
rs5917441	X	CASK	HGNC Symbol	calcium/calmodulin-dependent serine protein kinase (MAGUK family) [Source:HGNC Symbol;Acc:1497]
rs147839261	X	CASK	HGNC Symbol	calcium/calmodulin-dependent serine protein kinase (MAGUK family) [Source:HGNC Symbol;Acc:1497]
rs6609159	X	CASK	HGNC Symbol	calcium/calmodulin-dependent serine protein kinase (MAGUK family) [Source:HGNC Symbol;Acc:1497]
rs73193134	X	CASK	HGNC Symbol	calcium/calmodulin-dependent serine protein kinase (MAGUK family) [Source:HGNC Symbol;Acc:1497]
rs514729	X	CASK	HGNC Symbol	calcium/calmodulin-dependent serine protein kinase (MAGUK family) [Source:HGNC Symbol;Acc:1497]
rs62589167	X	CASK	HGNC Symbol	calcium/calmodulin-dependent serine protein kinase (MAGUK family) [Source:HGNC Symbol;Acc:1497]
rs34736295	X	CASK	HGNC Symbol	calcium/calmodulin-dependent serine protein kinase (MAGUK family) [Source:HGNC Symbol;Acc:1497]
rs17146134	X	CASK	HGNC Symbol	calcium/calmodulin-dependent serine protein kinase (MAGUK family) [Source:HGNC Symbol;Acc:1497]
rs112291337	X	CASK	HGNC Symbol	calcium/calmodulin-dependent serine protein kinase (MAGUK family) [Source:HGNC Symbol;Acc:1497]
rs5918245	X	CASK	HGNC Symbol	calcium/calmodulin-dependent serine protein kinase (MAGUK family) [Source:HGNC Symbol;Acc:1497]
rs73193141	X	CASK	HGNC Symbol	calcium/calmodulin-dependent serine protein kinase (MAGUK family) [Source:HGNC Symbol;Acc:1497]
rs12558921	X	CASK	HGNC Symbol	calcium/calmodulin-dependent serine protein kinase (MAGUK family) [Source:HGNC Symbol;Acc:1497]
rs5918264	X	CASK	HGNC Symbol	calcium/calmodulin-dependent serine protein kinase (MAGUK family) [Source:HGNC Symbol;Acc:1497]
rs17250058	X	CASK	HGNC Symbol	calcium/calmodulin-dependent serine protein kinase (MAGUK family) [Source:HGNC Symbol;Acc:1497]

SNP	Chromosome	gene name	gene source	description
rs1150380	X	CASK	HGNC Symbol	calcium/calmodulin-dependent serine protein kinase (MAGUK family) [Source:HGNC Symbol;Acc:1497]
rs5918265	X	CASK	HGNC Symbol	calcium/calmodulin-dependent serine protein kinase (MAGUK family) [Source:HGNC Symbol;Acc:1497]
rs73472547	X	CASK	HGNC Symbol	calcium/calmodulin-dependent serine protein kinase (MAGUK family) [Source:HGNC Symbol;Acc:1497]
rs5964065	X	CASK	HGNC Symbol	calcium/calmodulin-dependent serine protein kinase (MAGUK family) [Source:HGNC Symbol;Acc:1497]
rs5964067				
rs707440				
rs72626429				
rs5918272				
rs17250128				
rs72626430				
rs4827289				
rs1937118				
rs12557638				
rs73624970				
rs6520774				
rs1014784				
rs5917457				
rs138376213				
rs11796203				
rs72626441				
rs6609184				
rs5964077				
rs5918284				
rs5918286				
rs206064				
rs432284				
rs206051				
rs57502992				
rs55954619				
rs11796465				

SNP	Chromosome	gene name	gene source	description
rs2213601				
rs5917462				
rs5917466				
rs66475461				
rs5917468				
rs5918312				
rs62587418	X	RP1-154K9.2	Clone-based (Vega) gene	
rs73198601	X	RP1-154K9.2	Clone-based (Vega) gene	
rs2384947	X	RP1-154K9.2	Clone-based (Vega) gene	
rs2206146	X	RP1-154K9.2	Clone-based (Vega) gene	
rs17146230	X	RP1-154K9.2	Clone-based (Vega) gene	
rs714472	X	RP1-154K9.2	Clone-based (Vega) gene	
rs58316243	X	RP1-154K9.2	Clone-based (Vega) gene	
rs12849344	X	RP1-154K9.2	Clone-based (Vega) gene	
rs5918362	X	RP1-154K9.2	Clone-based (Vega) gene	
rs56274259	X	RP1-154K9.2	Clone-based (Vega) gene	
rs2213621	X	RP1-154K9.2	Clone-based (Vega) gene	
rs149320808	X	RP1-154K9.2	Clone-based (Vega) gene	
rs7066592	X	RP1-154K9.2	Clone-based (Vega) gene	
rs6610695	X	RP1-154K9.2	Clone-based (Vega) gene	
rs66914944	X	RP1-154K9.2	Clone-based (Vega) gene	
rs150834027	X	RP1-154K9.2	Clone-based (Vega) gene	

SNP	Chromosome	gene name	gene source	description
rs73200946	X	RP1-154K9.2	Clone-based (Vega) gene	
rs6610700	X	RP1-154K9.2	Clone-based (Vega) gene	
rs73200954	X	RP1-154K9.2	Clone-based (Vega) gene	
rs12833155	X	RP1-154K9.2	Clone-based (Vega) gene	
rs73208564	X	RP1-154K9.2	Clone-based (Vega) gene	
rs17146289	X	RP1-154K9.2	Clone-based (Vega) gene	
rs112794456	X	RP1-154K9.2	Clone-based (Vega) gene	
rs7886351	X	RP1-154K9.2	Clone-based (Vega) gene	
rs62588235	X	RP1-154K9.2	Clone-based (Vega) gene	
rs138493039				
rs73208570				
rs17214716				
rs141256388				
rs3921116				
rs12353585				
rs11266342				
rs139132298				
rs11796774				
rs2411001				
rs12851446				
rs5918409				
rs5964217				
rs73208579				
rs2157386				
rs146896335				
rs73208585				
rs5950918				

SNP	Chromosome	gene name	gene source	description
rs139414034				
rs2213606				
rs5991571				
rs17146360				
rs5951015				
rs67401067				
rs450472				
rs205845				
rs5950925				
rs73210281				
rs1112491				
rs10126264				
rs1396305				
rs12856241				
rs183043010				
rs73210289				
rs5991424				
rs5950938				
rs5991558				
rs147544363				
rs5950942				
rs5991446				
rs5991596				
rs73210301				
rs73631279				
rs73212004				
rs73212011				
rs112463230				
rs1983646				
rs5950972				
rs1033411				
rs73212025				

SNP	Chromosome	gene name	gene source	description
rs5950895	X	RP3-326I13.1	Clone-based (Vega) gene	
rs140826571				
rs62591795				
rs73208984				
rs73208986				
rs5991647				
rs5951009				
rs5950903				
rs113177008				
rs145269866				
rs142822380				
rs4986534				
rs5991663				
rs5991477				
rs7063048				
rs5951038				
rs5951051				
rs5906154				
rs5906276				
rs3788862	X	MAOA	HGNC Symbol	monoamine oxidase A [Source:HGNC Symbol;Acc:6833]
rs142677545	X	MAOA	HGNC Symbol	monoamine oxidase A [Source:HGNC Symbol;Acc:6833]
rs147023114	X	MAOA	HGNC Symbol	monoamine oxidase A [Source:HGNC Symbol;Acc:6833]
rs3027392	X	MAOA	HGNC Symbol	monoamine oxidase A [Source:HGNC Symbol;Acc:6833]
rs41303697	X	MAOA	HGNC Symbol	monoamine oxidase A [Source:HGNC Symbol;Acc:6833]
rs2179098	X	MAOA	HGNC Symbol	monoamine oxidase A [Source:HGNC Symbol;Acc:6833]
rs1800464	X	MAOA	HGNC Symbol	monoamine oxidase A [Source:HGNC Symbol;Acc:6833]
rs6323	X	MAOA	HGNC Symbol	monoamine oxidase A [Source:HGNC Symbol;Acc:6833]
rs2072743	X	MAOA	HGNC Symbol	monoamine oxidase A [Source:HGNC Symbol;Acc:6833]
rs5905418	X	MAOA	HGNC Symbol	monoamine oxidase A [Source:HGNC Symbol;Acc:6833]
rs1137070	X	MAOA	HGNC Symbol	monoamine oxidase A [Source:HGNC Symbol;Acc:6833]
rs3027409				
rs6609257				

SNP	Chromosome	gene name	gene source	description
rs73212713				
rs6324	X	MAOB	HGNC Symbol	monoamine oxidase B [Source:HGNC Symbol;Acc:6834]
rs1799836	X	MAOB	HGNC Symbol	monoamine oxidase B [Source:HGNC Symbol;Acc:6834]
rs3027450	X	MAOB	HGNC Symbol	monoamine oxidase B [Source:HGNC Symbol;Acc:6834]
rs3027452	X	MAOB	HGNC Symbol	monoamine oxidase B [Source:HGNC Symbol;Acc:6834]
rs72627377	X	MAOB	HGNC Symbol	monoamine oxidase B [Source:HGNC Symbol;Acc:6834]
rs1181252				
rs73212732				
rs3948768				
rs34387930				
rs209761				
rs5906278				
rs56017144	X	NDP;NDP-AS1	HGNC Symbol	Norrie disease (pseudoglioma) [Source:HGNC Symbol;Acc:7678];NDP antisense RNA 1 [Source:HGNC Symbol;Acc:40395]
rs2238972	X	NDP;NDP-AS1	HGNC Symbol	Norrie disease (pseudoglioma) [Source:HGNC Symbol;Acc:7678];NDP antisense RNA 1 [Source:HGNC Symbol;Acc:40395]
rs766117	X	NDP;NDP-AS1	HGNC Symbol	Norrie disease (pseudoglioma) [Source:HGNC Symbol;Acc:7678];NDP antisense RNA 1 [Source:HGNC Symbol;Acc:40395]
rs45501198	X	NDP;NDP-AS1	HGNC Symbol	Norrie disease (pseudoglioma) [Source:HGNC Symbol;Acc:7678];NDP antisense RNA 1 [Source:HGNC Symbol;Acc:40395]
rs5952976	X	NDP;NDP-AS1	HGNC Symbol	Norrie disease (pseudoglioma) [Source:HGNC Symbol;Acc:7678];NDP antisense RNA 1 [Source:HGNC Symbol;Acc:40395]
rs209766	X	NDP;NDP-AS1	HGNC Symbol	Norrie disease (pseudoglioma) [Source:HGNC Symbol;Acc:7678];NDP antisense RNA 1 [Source:HGNC Symbol;Acc:40395]
rs12839985	X	NDP;NDP-AS1	HGNC Symbol	Norrie disease (pseudoglioma) [Source:HGNC Symbol;Acc:7678];NDP antisense RNA 1 [Source:HGNC Symbol;Acc:40395]
rs147811246	X	NDP	HGNC Symbol	Norrie disease (pseudoglioma) [Source:HGNC Symbol;Acc:7678]
rs3761587				
rs5952416				
rs67933211				
rs73212757				
rs150326947				
rs5906426				
rs209767				
rs12556123				

SNP	Chromosome	gene name	gene source	description
rs55779415				
rs895867				
rs142601294				
rs287774				
rs73196113				
rs146766029				
rs4824671				
rs287772				
rs287770				
rs5905684				
rs4644408				
rs12014523				
rs287781				
rs5905703				
rs1335102				
rs2298065				
rs10521433	X	EFHC2	HGNC Symbol	EF-hand domain (C-terminal) containing 2 [Source:HGNC Symbol;Acc:26233]
rs6609283	X	EFHC2	HGNC Symbol	EF-hand domain (C-terminal) containing 2 [Source:HGNC Symbol;Acc:26233]
rs1181067	X	EFHC2	HGNC Symbol	EF-hand domain (C-terminal) containing 2 [Source:HGNC Symbol;Acc:26233]
rs34799018	X	EFHC2	HGNC Symbol	EF-hand domain (C-terminal) containing 2 [Source:HGNC Symbol;Acc:26233]
rs1181064	X	EFHC2	HGNC Symbol	EF-hand domain (C-terminal) containing 2 [Source:HGNC Symbol;Acc:26233]
rs73196195	X	EFHC2	HGNC Symbol	EF-hand domain (C-terminal) containing 2 [Source:HGNC Symbol;Acc:26233]
rs2208592	X	EFHC2	HGNC Symbol	EF-hand domain (C-terminal) containing 2 [Source:HGNC Symbol;Acc:26233]
rs147408131	X	EFHC2	HGNC Symbol	EF-hand domain (C-terminal) containing 2 [Source:HGNC Symbol;Acc:26233]
rs73483015	X	EFHC2	HGNC Symbol	EF-hand domain (C-terminal) containing 2 [Source:HGNC Symbol;Acc:26233]
rs73196201	X	EFHC2	HGNC Symbol	EF-hand domain (C-terminal) containing 2 [Source:HGNC Symbol;Acc:26233]
rs5953374	X	EFHC2	HGNC Symbol	EF-hand domain (C-terminal) containing 2 [Source:HGNC Symbol;Acc:26233]
rs149975561	X	EFHC2	HGNC Symbol	EF-hand domain (C-terminal) containing 2 [Source:HGNC Symbol;Acc:26233]
rs140925586	X	EFHC2	HGNC Symbol	EF-hand domain (C-terminal) containing 2 [Source:HGNC Symbol;Acc:26233]
rs66793041	X	EFHC2	HGNC Symbol	EF-hand domain (C-terminal) containing 2 [Source:HGNC Symbol;Acc:26233]
rs73628338	X	EFHC2	HGNC Symbol	EF-hand domain (C-terminal) containing 2 [Source:HGNC Symbol;Acc:26233]
rs61636783	X	EFHC2	HGNC Symbol	EF-hand domain (C-terminal) containing 2 [Source:HGNC Symbol;Acc:26233]
rs12014086	X	EFHC2	HGNC Symbol	EF-hand domain (C-terminal) containing 2 [Source:HGNC Symbol;Acc:26233]

SNP	Chromosome	gene name	gene source	description
rs5906917	X	EFHC2	HGNC Symbol	EF-hand domain (C-terminal) containing 2 [Source:HGNC Symbol;Acc:26233]
rs73628342	X	EFHC2	HGNC Symbol	EF-hand domain (C-terminal) containing 2 [Source:HGNC Symbol;Acc:26233]
rs17146919	X	EFHC2	HGNC Symbol	EF-hand domain (C-terminal) containing 2 [Source:HGNC Symbol;Acc:26233]
rs73198109	X	EFHC2	HGNC Symbol	EF-hand domain (C-terminal) containing 2 [Source:HGNC Symbol;Acc:26233]
rs5905799				
rs1562876				
rs5906945				
rs7889124				
rs5952572				
rs11798265				
rs6610918				
rs1451510				
rs73198136				
rs5953391				
rs17148382				
rs5905804				
rs12556969				
rs995355				
rs58152159				
rs5905817				
rs909658				
rs35243804				
rs11091272	X	RP4-551E13.2	Clone-based (Vega) gene	
rs5953415				
rs67697116				
rs12840599				
rs7057974				
rs12009034				
rs5953425				
rs5953432	X	FUNDC1	HGNC Symbol	FUN14 domain containing 1 [Source:HGNC Symbol;Acc:28746]
rs56076153	X	FUNDC1	HGNC Symbol	FUN14 domain containing 1 [Source:HGNC Symbol;Acc:28746]
rs6610953	X	FUNDC1	HGNC Symbol	FUN14 domain containing 1 [Source:HGNC Symbol;Acc:28746]

SNP	Chromosome	gene name	gene source	description
rs41309613	X	FUNDC1	HGNC Symbol	FUN14 domain containing 1 [Source:HGNC Symbol;Acc:28746]
rs60334020				
rs73633297				
rs5953452				
rs5953457				
rs5952595				
rs5907006				
rs5907007				
rs72627393				
rs59597370				
rs62591393				
rs6609313				
rs4824841				
rs4824843				
rs5907035				
rs12861071				
rs5952603				
rs112998199				
rs10126477				
rs66485769				
rs112917037				
rs68188385				
rs17300235				
rs4824517				
rs607764				
rs142255983				
rs113786337				
rs57542360				
rs5905875				
rs66509220				
rs12849327				
rs5952255				
rs17146993				

SNP	Chromosome	gene name	gene source	description
rs4824519				
rs60913891				
rs6520981				
rs5905899				
rs58972347				
rs73486952				
rs1045031	X	DUSP21	HGNC Symbol	dual specificity phosphatase 21 [Source:HGNC Symbol;Acc:20476]
rs12846943				
rs12393337	X	KDM6A	HGNC Symbol	lysine (K)-specific demethylase 6A [Source:HGNC Symbol;Acc:12637]
rs73200183	X	KDM6A	HGNC Symbol	lysine (K)-specific demethylase 6A [Source:HGNC Symbol;Acc:12637]
rs73488844	X	KDM6A	HGNC Symbol	lysine (K)-specific demethylase 6A [Source:HGNC Symbol;Acc:12637]
rs1172541	X	KDM6A	HGNC Symbol	lysine (K)-specific demethylase 6A [Source:HGNC Symbol;Acc:12637]
rs6611055	X	KDM6A	HGNC Symbol	lysine (K)-specific demethylase 6A [Source:HGNC Symbol;Acc:12637]
rs12559916	X	KDM6A	HGNC Symbol	lysine (K)-specific demethylase 6A [Source:HGNC Symbol;Acc:12637]
rs2230018	X	KDM6A	HGNC Symbol	lysine (K)-specific demethylase 6A [Source:HGNC Symbol;Acc:12637]
rs5952288	X	KDM6A	HGNC Symbol	lysine (K)-specific demethylase 6A [Source:HGNC Symbol;Acc:12637]
rs146337123				
rs6521004				
rs12687686	X	CXorf36	HGNC Symbol	chromosome X open reading frame 36 [Source:HGNC Symbol;Acc:25866]
rs7050908	X	CXorf36	HGNC Symbol	chromosome X open reading frame 36 [Source:HGNC Symbol;Acc:25866]
rs4452929	X	CXorf36	HGNC Symbol	chromosome X open reading frame 36 [Source:HGNC Symbol;Acc:25866]
rs4294309	X	CXorf36	HGNC Symbol	chromosome X open reading frame 36 [Source:HGNC Symbol;Acc:25866]
rs1132201	X	CXorf36;RP11-342D14.1	HGNC Symbol;Clone-based (Vega) gene	chromosome X open reading frame 36 [Source:HGNC Symbol;Acc:25866];
rs4335267	X	CXorf36;RP11-342D14.1	HGNC Symbol;Clone-based (Vega) gene	chromosome X open reading frame 36 [Source:HGNC Symbol;Acc:25866];
rs113500388	X	CXorf36;RP11-342D14.1	HGNC Symbol;Clone-based (Vega) gene	chromosome X open reading frame 36 [Source:HGNC Symbol;Acc:25866];
rs4239959	X	CXorf36;RP11-342D14.1	HGNC Symbol;Clone-based (Vega) gene	chromosome X open reading frame 36 [Source:HGNC Symbol;Acc:25866];
rs5905937	X	RP11-342D14.1	Clone-based (Vega) gene	

SNP	Chromosome	gene name	gene source	description
rs61400284	X	RP11-342D14.1	Clone-based (Vega) gene	
rs17148154	X	RP11-342D14.1	Clone-based (Vega) gene	
rs141133274	X	RP11-342D14.1	Clone-based (Vega) gene	
rs5905441	X	RP11-342D14.1	Clone-based (Vega) gene	
rs73208643	X	RP11-342D14.1	Clone-based (Vega) gene	
rs7052648	X	RP11-342D14.1	Clone-based (Vega) gene	
rs112633712	X	RP11-342D14.1	Clone-based (Vega) gene	
rs4446859	X	RP11-342D14.1	Clone-based (Vega) gene	
rs73210707	X	RP11-342D14.1	Clone-based (Vega) gene	
rs5905447	X	RP11-342D14.1	Clone-based (Vega) gene	
rs4481737	X	RP11-342D14.1	Clone-based (Vega) gene	
rs17261222				
rs147620253				
rs12396486				
rs5905996				
rs5906004				
rs113799864				
rs149858608				
rs5952756				
rs112531252				
rs10854928				
rs41312098	X	RP11-245M24.1	Clone-based (Vega) gene	
rs5952318	X	RP11-245M24.1	Clone-based (Vega) gene	
rs1536278	X	RP11-245M24.1	Clone-based (Vega) gene	

SNP	Chromosome	gene name	gene source	description
rs73210734	X	RP11-245M24.1	Clone-based (Vega) gene	
rs56759370	X	RP11-245M24.1	Clone-based (Vega) gene	
rs5906035	X	RP11-245M24.1	Clone-based (Vega) gene	
rs73210737	X	RP11-245M24.1	Clone-based (Vega) gene	
rs12837681				
rs72628925				
rs2353631				
rs5906041				
rs6609366				
rs62587270				
rs5906048				
rs2022502				
rs5905475				
rs1883675				
rs1883672				
rs1207316				
rs150553877				
rs2858204				
rs55984215				
rs56222302	X;HG1435_PATCH	RP6-99M1.2	Clone-based (Vega) gene	
rs17310797	X;HG1435_PATCH	RP6-99M1.2	Clone-based (Vega) gene	
rs2858058	X;HG1435_PATCH	RP6-99M1.2	Clone-based (Vega) gene	
rs7887062	X;HG1435_PATCH	RP6-99M1.2	Clone-based (Vega) gene	
rs112143425				
rs6609372				
rs113259563				
rs5906072				
rs58233894				

SNP	Chromosome	gene name	gene source	description
rs5906076				
rs4824561				
rs17300297				
rs6521042				
rs5906083				
rs2009184				
rs7058787				
rs59669225				
rs5952803				
rs5906093				
rs73212648				
rs143321538				
rs5952332				
rs17215229				
rs35857391				
rs5906109				
rs5906116				
rs1028362				
rs141622115				
rs5906120				
rs145916556				
rs73628218				
rs5906130				
rs5906131				
rs13440683				
rs5906133				
rs2148106				
rs73196055				
rs982150				
rs5906143				
rs5905517				
rs17300353				
rs1207304				

SNP	Chromosome	gene name	gene source	description
rs139328779				
rs1936653				
rs1207265				
rs1207264				
rs5906163				
rs5905526				
rs12687220				
rs79796220				
rs56074764				
rs73196069	X	ACTBP1	HGNC Symbol	actin, beta pseudogene 1 [Source:HGNC Symbol;Acc:134]
rs58450717				
rs1936651				
rs72628950				
rs5952854				
rs6521093				
rs851234				
rs10521444				
rs851223				
rs2209585				
rs59431030				
rs761200				
rs5906180				
rs5952865				
rs62590007				
rs5905536				
rs1325986				
rs12558451				
rs4824580				
rs5906201				
rs1409197				
rs6611244	X	KRBOX4	HGNC Symbol	KRAB box domain containing 4 [Source:HGNC Symbol;Acc:26007]
rs1100916	X	KRBOX4	HGNC Symbol	KRAB box domain containing 4 [Source:HGNC Symbol;Acc:26007]
rs149674363	X	KRBOX4	HGNC Symbol	KRAB box domain containing 4 [Source:HGNC Symbol;Acc:26007]

SNP	Chromosome	gene name	gene source	description
rs60762218	X	KRBOX4	HGNC Symbol	KRAB box domain containing 4 [Source:HGNC Symbol;Acc:26007]
rs62593969	X	ZNF674	HGNC Symbol	zinc finger protein 674 [Source:HGNC Symbol;Acc:17625]
rs7889429	X	ZNF674	HGNC Symbol	zinc finger protein 674 [Source:HGNC Symbol;Acc:17625]
rs73198088				
rs4824584				
rs6521128	X	CHST7	HGNC Symbol	carbohydrate (N-acetylglucosamine 6-O) sulfotransferase 7 [Source:HGNC Symbol;Acc:13817]
rs11796837	X	CHST7	HGNC Symbol	carbohydrate (N-acetylglucosamine 6-O) sulfotransferase 7 [Source:HGNC Symbol;Acc:13817]
rs735716	X	CHST7	HGNC Symbol	carbohydrate (N-acetylglucosamine 6-O) sulfotransferase 7 [Source:HGNC Symbol;Acc:13817]
rs732316				
rs6640				
rs12688544	X	SLC9A7	HGNC Symbol	solute carrier family 9, subfamily A (NHE7, cation proton antiporter 7), member 7 [Source:HGNC Symbol;Acc:17123]
rs73200258	X	SLC9A7	HGNC Symbol	solute carrier family 9, subfamily A (NHE7, cation proton antiporter 7), member 7 [Source:HGNC Symbol;Acc:17123]
rs3208940	X	SLC9A7	HGNC Symbol	solute carrier family 9, subfamily A (NHE7, cation proton antiporter 7), member 7 [Source:HGNC Symbol;Acc:17123]
rs6611279	X	SLC9A7	HGNC Symbol	solute carrier family 9, subfamily A (NHE7, cation proton antiporter 7), member 7 [Source:HGNC Symbol;Acc:17123]
rs73200264	X	SLC9A7	HGNC Symbol	solute carrier family 9, subfamily A (NHE7, cation proton antiporter 7), member 7 [Source:HGNC Symbol;Acc:17123]
rs909662	X	SLC9A7	HGNC Symbol	solute carrier family 9, subfamily A (NHE7, cation proton antiporter 7), member 7 [Source:HGNC Symbol;Acc:17123]
rs73200266	X	SLC9A7	HGNC Symbol	solute carrier family 9, subfamily A (NHE7, cation proton antiporter 7), member 7 [Source:HGNC Symbol;Acc:17123]
rs11797818	X	SLC9A7	HGNC Symbol	solute carrier family 9, subfamily A (NHE7, cation proton antiporter 7), member 7 [Source:HGNC Symbol;Acc:17123]
rs28445915	X	SLC9A7	HGNC Symbol	solute carrier family 9, subfamily A (NHE7, cation proton antiporter 7), member 7 [Source:HGNC Symbol;Acc:17123]
rs6521138	X	SLC9A7;YBX1P8	HGNC Symbol	solute carrier family 9, subfamily A (NHE7, cation proton antiporter 7), member 7 [Source:HGNC Symbol;Acc:17123];Y box binding protein 1 pseudogene 8 [Source:HGNC Symbol;Acc:42429]
rs5906255	X	SLC9A7	HGNC Symbol	solute carrier family 9, subfamily A (NHE7, cation proton antiporter 7), member 7 [Source:HGNC Symbol;Acc:17123]
rs4824410	X	SLC9A7	HGNC Symbol	solute carrier family 9, subfamily A (NHE7, cation proton antiporter 7), member 7 [Source:HGNC Symbol;Acc:17123]
rs5905564	X	SLC9A7	HGNC Symbol	solute carrier family 9, subfamily A (NHE7, cation proton antiporter 7), member 7 [Source:HGNC Symbol;Acc:17123]
rs996126				
rs148555208				

SNP	Chromosome	gene name	gene source	description
rs6609437				
rs73478366				
rs5952966	X;HG29_PATCH	RP2	HGNC Symbol	retinitis pigmentosa 2 (X-linked recessive) [Source:HGNC Symbol;Acc:10274]
rs1805147	X;HG29_PATCH	RP2	HGNC Symbol	retinitis pigmentosa 2 (X-linked recessive) [Source:HGNC Symbol;Acc:10274]
rs55893393				
rs5906288				
rs12560179	X	JADE3	HGNC Symbol	jade family PHD finger 3 [Source:HGNC Symbol;Acc:22982]
rs4567179	X	JADE3	HGNC Symbol	jade family PHD finger 3 [Source:HGNC Symbol;Acc:22982]
rs2187789	X	JADE3	HGNC Symbol	jade family PHD finger 3 [Source:HGNC Symbol;Acc:22982]
rs138989995	X	JADE3	HGNC Symbol	jade family PHD finger 3 [Source:HGNC Symbol;Acc:22982]
rs3126139	X	JADE3	HGNC Symbol	jade family PHD finger 3 [Source:HGNC Symbol;Acc:22982]
rs138651080	X	JADE3	HGNC Symbol	jade family PHD finger 3 [Source:HGNC Symbol;Acc:22982]
rs73201913	X	JADE3	HGNC Symbol	jade family PHD finger 3 [Source:HGNC Symbol;Acc:22982]
rs149673021	X	JADE3	HGNC Symbol	jade family PHD finger 3 [Source:HGNC Symbol;Acc:22982]
rs59621587	X	JADE3	HGNC Symbol	jade family PHD finger 3 [Source:HGNC Symbol;Acc:22982]
rs6725	X	JADE3	HGNC Symbol	jade family PHD finger 3 [Source:HGNC Symbol;Acc:22982]
rs6521175				
rs5952995				
rs12846518				
rs143304641				
rs4503212	X;HG29_PATCH	RGN	HGNC Symbol	regucalcin [Source:HGNC Symbol;Acc:9989]
rs7882477	X	RGN	HGNC Symbol	regucalcin [Source:HGNC Symbol;Acc:9989]
rs62591340				
rs5906330				
rs4824419				
rs73201990				
rs12852223	X;HG29_PATCH	RBM10	HGNC Symbol	RNA binding motif protein 10 [Source:HGNC Symbol;Acc:9896]
rs79693964	X;HG29_PATCH	RBM10	HGNC Symbol	RNA binding motif protein 10 [Source:HGNC Symbol;Acc:9896]
rs2070169	X	UBA1	HGNC Symbol	ubiquitin-like modifier activating enzyme 1 [Source:HGNC Symbol;Acc:12469]
rs145546830	X	CDK16	HGNC Symbol	cyclin-dependent kinase 16 [Source:HGNC Symbol;Acc:8749]
rs17550472	X	CDK16	HGNC Symbol	cyclin-dependent kinase 16 [Source:HGNC Symbol;Acc:8749]
rs6417923	X	CDK16	HGNC Symbol	cyclin-dependent kinase 16 [Source:HGNC Symbol;Acc:8749]
rs41305231	X	CDK16	HGNC Symbol	cyclin-dependent kinase 16 [Source:HGNC Symbol;Acc:8749]

SNP	Chromosome	gene name	gene source	description
rs17327529	X	USP11	HGNC Symbol	ubiquitin specific peptidase 11 [Source:HGNC Symbol;Acc:12609]
rs17327536	X	USP11	HGNC Symbol	ubiquitin specific peptidase 11 [Source:HGNC Symbol;Acc:12609]
rs17261658				
rs5906367				
rs3005644				
rs4824604				
rs12559649				
rs139127466				
rs57854640				
rs5953025				
rs2474323				
rs6611365				
rs12013699				
rs55836029				
rs2064596				
rs12846558				
rs743046				
rs146489693				
rs5906381				
rs62594693				
rs11796948				
rs2474314				
rs7063875				
rs6520263	X	ZNF157	HGNC Symbol	zinc finger protein 157 [Source:HGNC Symbol;Acc:12942]
rs13440834	X	ZNF157	HGNC Symbol	zinc finger protein 157 [Source:HGNC Symbol;Acc:12942]
rs4498683				
rs73204096				
rs2498170	X	ZNF41	HGNC Symbol	zinc finger protein 41 [Source:HGNC Symbol;Acc:13107]
rs17147624	X	ZNF41	HGNC Symbol	zinc finger protein 41 [Source:HGNC Symbol;Acc:13107]
rs2498488	X	ZNF41	HGNC Symbol	zinc finger protein 41 [Source:HGNC Symbol;Acc:13107]
rs4327971	X	ZNF41	HGNC Symbol	zinc finger protein 41 [Source:HGNC Symbol;Acc:13107]
rs5953045	X	ZNF41	HGNC Symbol	zinc finger protein 41 [Source:HGNC Symbol;Acc:13107]
rs150321593	X	ZNF41	HGNC Symbol	zinc finger protein 41 [Source:HGNC Symbol;Acc:13107]

SNP	Chromosome	gene name	gene source	description
rs2187803				
rs60174983				
rs140727532				
rs3748516				
rs73206108				
rs7891301				
rs2313072				
rs5905611				
rs56014229	X	ARAF	HGNC Symbol	v-raf murine sarcoma 3611 viral oncogene homolog [Source:HGNC Symbol;Acc:646]
rs2071777	X	ARAF	HGNC Symbol	v-raf murine sarcoma 3611 viral oncogene homolog [Source:HGNC Symbol;Acc:646]
rs2858769	X	ARAF	HGNC Symbol	v-raf murine sarcoma 3611 viral oncogene homolog [Source:HGNC Symbol;Acc:646]
rs34576985	X	SYN1	HGNC Symbol	synapsin I [Source:HGNC Symbol;Acc:11494]
rs55990337	X	SYN1;TIMP1	HGNC Symbol	synapsin I [Source:HGNC Symbol;Acc:11494];TIMP metallopeptidase inhibitor 1 [Source:HGNC Symbol;Acc:11820]
rs4898	X	SYN1;TIMP1	HGNC Symbol	synapsin I [Source:HGNC Symbol;Acc:11494];TIMP metallopeptidase inhibitor 1 [Source:HGNC Symbol;Acc:11820]
rs6520279	X	SYN1	HGNC Symbol	synapsin I [Source:HGNC Symbol;Acc:11494]
rs5906435	X	SYN1	HGNC Symbol	synapsin I [Source:HGNC Symbol;Acc:11494]
rs12559303	X	SYN1	HGNC Symbol	synapsin I [Source:HGNC Symbol;Acc:11494]
rs73206134	X	SYN1	HGNC Symbol	synapsin I [Source:HGNC Symbol;Acc:11494]
rs5953066	X	SYN1	HGNC Symbol	synapsin I [Source:HGNC Symbol;Acc:11494]
rs1142636	X	SYN1	HGNC Symbol	synapsin I [Source:HGNC Symbol;Acc:11494]
rs4824624	X	SYN1	HGNC Symbol	synapsin I [Source:HGNC Symbol;Acc:11494]
rs4824628				
rs8177084				
rs1048118	X	CFP	HGNC Symbol	complement factor properdin [Source:HGNC Symbol;Acc:8864]
rs7060246	X	CFP	HGNC Symbol	complement factor properdin [Source:HGNC Symbol;Acc:8864]
rs1998837	X	ELK1	HGNC Symbol	ELK1, member of ETS oncogene family [Source:HGNC Symbol;Acc:3321]
rs5953077				
rs35107876				
rs7051207				
rs1590561				
rs17332415	X	CXXC1P1	HGNC Symbol	CXXC finger protein 1 pseudogene 1 [Source:HGNC Symbol;Acc:27864]
rs6609568	X	CXXC1P1	HGNC Symbol	CXXC finger protein 1 pseudogene 1 [Source:HGNC Symbol;Acc:27864]

SNP	Chromosome	gene name	gene source	description
rs5906466	X	CXXC1P1	HGNC Symbol	CXXC finger protein 1 pseudogene 1 [Source:HGNC Symbol;Acc:27864]
rs73206154	X	CXXC1P1	HGNC Symbol	CXXC finger protein 1 pseudogene 1 [Source:HGNC Symbol;Acc:27864]
rs5953093	X	CXXC1P1	HGNC Symbol	CXXC finger protein 1 pseudogene 1 [Source:HGNC Symbol;Acc:27864]
rs78168288				
rs5905625				
rs5952451				
rs2506769	X;HG1436_HG1432_PATCH	RP3-393P12.1	Clone-based (Vega) gene	
rs2022365	X;HG1436_HG1432_PATCH	ZNF81	HGNC Symbol	zinc finger protein 81 [Source:HGNC Symbol;Acc:13156]
rs183846665	X;HG1436_HG1432_PATCH	ZNF81	HGNC Symbol	zinc finger protein 81 [Source:HGNC Symbol;Acc:13156]
rs11091213	X	ZNF81	HGNC Symbol	zinc finger protein 81 [Source:HGNC Symbol;Acc:13156]
rs7064485	X	ZNF81	HGNC Symbol	zinc finger protein 81 [Source:HGNC Symbol;Acc:13156]
rs9887125	X	ZNF81	HGNC Symbol	zinc finger protein 81 [Source:HGNC Symbol;Acc:13156]
rs5906506	X;HG1436_HG1432_PATCH	ZNF81	HGNC Symbol	zinc finger protein 81 [Source:HGNC Symbol;Acc:13156]
rs149527962	X;HG1436_HG1432_PATCH	ZNF81	HGNC Symbol	zinc finger protein 81 [Source:HGNC Symbol;Acc:13156]
rs45588937	X;HG1436_HG1432_PATCH	ZNF81	HGNC Symbol	zinc finger protein 81 [Source:HGNC Symbol;Acc:13156]
rs5952463	X;HG1436_HG1432_PATCH	ZNF182;ZNF81	HGNC Symbol	zinc finger protein 182 [Source:HGNC Symbol;Acc:13001];zinc finger protein 81 [Source:HGNC Symbol;Acc:13156]
rs536366	X;HG1436_HG1432_PATCH	ZNF630	HGNC Symbol	zinc finger protein 630 [Source:HGNC Symbol;Acc:28855]
rs541367	X;HG1436_HG1432_PATCH	ZNF630	HGNC Symbol	zinc finger protein 630 [Source:HGNC Symbol;Acc:28855]
rs507395	X;HG1436_HG1432_PATCH	ZNF630	HGNC Symbol	zinc finger protein 630 [Source:HGNC Symbol;Acc:28855]
rs560972	X;HG1436_HG1432_PATCH	ZNF630	HGNC Symbol	zinc finger protein 630 [Source:HGNC Symbol;Acc:28855]
rs472255	X;HG1436_HG1432_PATCH	ZNF630	HGNC Symbol	zinc finger protein 630 [Source:HGNC Symbol;Acc:28855]
rs5905647	X;HG1436_HG1432_PATCH	ZNF630	HGNC Symbol	zinc finger protein 630 [Source:HGNC Symbol;Acc:28855]
rs492351	X;HG1436_HG1432_PATCH	ZNF630	HGNC Symbol	zinc finger protein 630 [Source:HGNC Symbol;Acc:28855]

SNP	Chromosome	gene name	gene source	description
rs41297342	X;HG1436_HG1432_PATCH	ZNF630;ZNF630-AS1	HGNC Symbol	zinc finger protein 630 [Source:HGNC Symbol;Acc:28855];ZNF630 antisense RNA 1 [Source:HGNC Symbol;Acc:41215]
rs491610	X;HG1436_HG1432_PATCH	ZNF630;ZNF630-AS1	HGNC Symbol	zinc finger protein 630 [Source:HGNC Symbol;Acc:28855];ZNF630 antisense RNA 1 [Source:HGNC Symbol;Acc:41215]
rs7050994				
rs35962780				
rs560236				
rs73207853				
rs5906541				
rs77265236				
rs5953181	X;HG1436_HG1432_PATCH	SSXP3	HGNC Symbol	SSX family pseudogene 3 [Source:HGNC Symbol;Acc:30640]
rs6608777				
rs5905662				
rs7056162				
rs140029421	X;HG1436_HG1432_PATCH	SSX1	HGNC Symbol	synovial sarcoma, X breakpoint 1 [Source:HGNC Symbol;Acc:11335]
rs55839103	X;HG1436_HG1432_PATCH	SSX1	HGNC Symbol	synovial sarcoma, X breakpoint 1 [Source:HGNC Symbol;Acc:11335]
rs7060353				
rs5953215				
rs7061151				
rs55861983	X;HG1436_HG1432_PATCH	SSX9	HGNC Symbol	synovial sarcoma, X breakpoint 9 [Source:HGNC Symbol;Acc:19655]
rs4598385	X;HG1436_HG1432_PATCH	SSX9	HGNC Symbol	synovial sarcoma, X breakpoint 9 [Source:HGNC Symbol;Acc:19655]
rs72619006				
rs4824704				
rs12057122				
rs5905683	X;HG1436_HG1432_PATCH	SSX3	HGNC Symbol	synovial sarcoma, X breakpoint 3 [Source:HGNC Symbol;Acc:11337]
rs2005947	X;HG1436_HG1432_PATCH	AF196972.3	Clone-based (Vega) gene	
rs2977591				
rs56349136				

SNP	Chromosome	gene name	gene source	description
rs17281188	X;HG1436_HG1432_PATCH	SLC38A5	HGNC Symbol;UniProtKB Gene Name	solute carrier family 38, member 5 [Source:HGNC Symbol;Acc:18070];Sodium-coupled neutral amino acid transporter 5 [Source:UniProtKB/TrEMBL;Acc:C9JNK4]
rs5906671	X;HG1436_HG1432_PATCH	SLC38A5	HGNC Symbol;UniProtKB Gene Name	solute carrier family 38, member 5 [Source:HGNC Symbol;Acc:18070];Sodium-coupled neutral amino acid transporter 5 [Source:UniProtKB/TrEMBL;Acc:C9JNK4]
rs73209722				
rs17250918	X	FTSJ1	HGNC Symbol	FtsJ RNA methyltransferase homolog 1 (E. coli) [Source:HGNC Symbol;Acc:13254]
rs5905692	X;HG1436_HG1432_PATCH	FTSJ1	HGNC Symbol	FtsJ RNA methyltransferase homolog 1 (E. coli) [Source:HGNC Symbol;Acc:13254]
rs5953242				
rs6608809				
rs111771187	X;HG1436_HG1432_PATCH	PORCN	HGNC Symbol	porcupine homolog (Drosophila) [Source:HGNC Symbol;Acc:17652]
rs41312163	X;HG1436_HG1432_PATCH	TBC1D25	HGNC Symbol	TBC1 domain family, member 25 [Source:HGNC Symbol;Acc:8092]
rs11795697	X;HG1436_HG1432_PATCH	TBC1D25	HGNC Symbol	TBC1 domain family, member 25 [Source:HGNC Symbol;Acc:8092]
rs2293948	X;HG1436_HG1432_PATCH	TBC1D25	HGNC Symbol	TBC1 domain family, member 25 [Source:HGNC Symbol;Acc:8092]
rs149387926				
rs235843	X;HG1436_HG1432_PATCH	WDR13	HGNC Symbol	WD repeat domain 13 [Source:HGNC Symbol;Acc:14352]
rs62600294				
rs56845814				
rs235820				
rs58214541				
rs112617119	X;HG1436_HG1432_PATCH	VN1R110P	HGNC Symbol	vomeronasal 1 receptor 110 pseudogene [Source:HGNC Symbol;Acc:37438]
rs57924938				
rs12559480				
rs111297033				
rs72619028	X;HG1436_HG1432_PATCH	SUV39H1	HGNC Symbol	suppressor of variegation 3-9 homolog 1 (Drosophila) [Source:HGNC Symbol;Acc:11479]
rs3761543	X;HG1436_HG1432_PATCH	SUV39H1	HGNC Symbol	suppressor of variegation 3-9 homolog 1 (Drosophila) [Source:HGNC Symbol;Acc:11479]

SNP	Chromosome	gene name	gene source	description
rs3373	X;HG1436_HG1432_PATCH	SUV39H1;AF196970.3	HGNC Symbol;Clone-based (Vega) gene	suppressor of variegation 3-9 homolog 1 (Drosophila) [Source:HGNC Symbol;Acc:11479];
rs113332558	X;HG1436_HG1432_PATCH	AF196970.3	Clone-based (Vega) gene	
rs55903338				
rs5906705				
rs5906707	X;HG1436_HG1432_PATCH	GLOD5	HGNC Symbol	glyoxalase domain containing 5 [Source:HGNC Symbol;Acc:33358]
rs200192031	X;HG1436_HG1432_PATCH	GLOD5	HGNC Symbol	glyoxalase domain containing 5 [Source:HGNC Symbol;Acc:33358]
rs5906710				
rs17281209				
rs2075840	X;HG1436_HG1432_PATCH	HDAC6	HGNC Symbol	histone deacetylase 6 [Source:HGNC Symbol;Acc:14064]
rs61735967	X;HG1436_HG1432_PATCH	HDAC6	HGNC Symbol	histone deacetylase 6 [Source:HGNC Symbol;Acc:14064]
rs201845777	X;HG1436_HG1432_PATCH	HDAC6	HGNC Symbol	histone deacetylase 6 [Source:HGNC Symbol;Acc:14064]
rs151130423	X;HG1436_HG1432_PATCH	HDAC6	HGNC Symbol	histone deacetylase 6 [Source:HGNC Symbol;Acc:14064]
rs149923021				
rs55721510	X;HG1436_HG1432_PATCH	TIMM17B	HGNC Symbol	translocase of inner mitochondrial membrane 17 homolog B (yeast) [Source:HGNC Symbol;Acc:17310]
rs717193	X;HG1436_HG1432_PATCH	SLC35A2	HGNC Symbol	solute carrier family 35 (UDP-galactose transporter), member A2 [Source:HGNC Symbol;Acc:11022]
rs55719932	X;HG1436_HG1432_PATCH	SLC35A2	HGNC Symbol	solute carrier family 35 (UDP-galactose transporter), member A2 [Source:HGNC Symbol;Acc:11022]
rs3027514	X;HG1436_HG1432_PATCH	PIM2	HGNC Symbol	pim-2 oncogene [Source:HGNC Symbol;Acc:8987]
rs189130449				
rs3027476	X;HG1436_HG1432_PATCH	GRIPAP1;AC233294.1	HGNC Symbol;Clone-based (Ensembl) gene	GRIP1 associated protein 1 [Source:HGNC Symbol;Acc:18706];
rs56350738	X;HG1436_HG1432_PATCH	GRIPAP1;AC233294.1	HGNC Symbol;Clone-based (Ensembl) gene	GRIP1 associated protein 1 [Source:HGNC Symbol;Acc:18706];

SNP	Chromosome	gene name	gene source	description
rs28446716	X;HG1436_HG1432_PATCH	GRIPAP1;AC233294.1	HGNC Symbol;Clone-based (Ensembl) gene	GRIP1 associated protein 1 [Source:HGNC Symbol;Acc:18706];
rs3027474				
rs3027471	X	TFE3	HGNC Symbol	transcription factor binding to IGHM enhancer 3 [Source:HGNC Symbol;Acc:11752]
rs4824458				
rs56087424	X;HG1436_HG1432_PATCH	CCDC120	HGNC Symbol;UniProtKB Gene Name	coiled-coil domain containing 120 [Source:HGNC Symbol;Acc:28910];JM11 protein, isoform CRA_a; cDNA FLJ76847, highly similar to Homo sapiens JM11 protein (JM11), mRNA; cDNA, FLJ79486, weakly similar to FERM domain-containing protein 4B [Source:UniProtKB/TrEMBL;Acc:A8K5Q5]
rs144149884				
rs12843494	X	MAGIX	HGNC Symbol	MAGI family member, X-linked [Source:HGNC Symbol;Acc:30006]
rs12557970	X;HG1436_HG1432_PATCH	PRICKLE3	HGNC Symbol;UniProtKB Gene Name	prickle homolog 3 (Drosophila) [Source:HGNC Symbol;Acc:6645];Prickle-like protein 3 [Source:UniProtKB/TrEMBL;Acc:F5H4N2]
rs4824745	X	PRICKLE3	HGNC Symbol	prickle homolog 3 (Drosophila) [Source:HGNC Symbol;Acc:6645]
rs3810680				
rs5905722	X;HG1436_HG1432_PATCH	SYP	HGNC Symbol	synaptophysin [Source:HGNC Symbol;Acc:11506]
rs33910054	X;HG1436_HG1432_PATCH	CACNA1F	HGNC Symbol	calcium channel, voltage-dependent, L type, alpha 1F subunit [Source:HGNC Symbol;Acc:1393]
rs2071316	X;HG1436_HG1432_PATCH	CACNA1F	HGNC Symbol	calcium channel, voltage-dependent, L type, alpha 1F subunit [Source:HGNC Symbol;Acc:1393]
rs2235127	X;HG1436_HG1432_PATCH	CACNA1F	HGNC Symbol	calcium channel, voltage-dependent, L type, alpha 1F subunit [Source:HGNC Symbol;Acc:1393]
rs34162630	X;HG1436_HG1432_PATCH	CACNA1F	HGNC Symbol	calcium channel, voltage-dependent, L type, alpha 1F subunit [Source:HGNC Symbol;Acc:1393]
rs145961306	X;HG1436_HG1432_PATCH	CCDC22	HGNC Symbol	coiled-coil domain containing 22 [Source:HGNC Symbol;Acc:28909]
rs2294020	X;HG1436_HG1432_PATCH	CCDC22	HGNC Symbol	coiled-coil domain containing 22 [Source:HGNC Symbol;Acc:28909]
rs2232367	X;HG1436_HG1432_PATCH	FOXP3	HGNC Symbol	forkhead box P3 [Source:HGNC Symbol;Acc:6106]
rs3761547	X;HG1436_HG1432_PATCH	FOXP3	HGNC Symbol	forkhead box P3 [Source:HGNC Symbol;Acc:6106]
rs2904021				
rs4824755	X	GAGE10	HGNC Symbol	G antigen 10 [Source:HGNC Symbol;Acc:30968]
rs11795807	X	GAGE10	HGNC Symbol	G antigen 10 [Source:HGNC Symbol;Acc:30968]

SNP	Chromosome	gene name	gene source	description
rs5906782	X	GAGE10	HGNC Symbol	G antigen 10 [Source:HGNC Symbol;Acc:30968]
rs148793590	X;HG1436_HG1432_PATCH	GAGE1	HGNC Symbol	G antigen 1 [Source:HGNC Symbol;Acc:4098]
rs5906844	X;HG1436_HG1432_PATCH	GAGE1	HGNC Symbol	G antigen 1 [Source:HGNC Symbol;Acc:4098]
rs5906845	X;HG1436_HG1432_PATCH	GAGE1	HGNC Symbol	G antigen 1 [Source:HGNC Symbol;Acc:4098]
rs41305753	X;HG1436_HG1432_PATCH	GAGE1	HGNC Symbol	G antigen 1 [Source:HGNC Symbol;Acc:4098]
rs6520435	X;HG1436_HG1432_PATCH	GAGE1	HGNC Symbol	G antigen 1 [Source:HGNC Symbol;Acc:4098]
rs62602681				
rs6417816				
rs148107284				
rs5953333				
rs35699735				
rs7884658				
rs147376247				
rs5906865				
rs1882984				
rs4824801				
rs34620314				
rs17174031				
rs5906888	X	USP27X-AS1	HGNC Symbol	USP27X antisense RNA 1 (head to head) [Source:HGNC Symbol;Acc:27249]
rs149256				
rs151259	X;HG1436_HG1432_PATCH	CLCN5	HGNC Symbol	chloride channel, voltage-sensitive 5 [Source:HGNC Symbol;Acc:2023]
rs10521460	X;HG1436_HG1432_PATCH	CLCN5	HGNC Symbol	chloride channel, voltage-sensitive 5 [Source:HGNC Symbol;Acc:2023]
rs179824	X;HG1436_HG1432_PATCH	CLCN5	HGNC Symbol	chloride channel, voltage-sensitive 5 [Source:HGNC Symbol;Acc:2023]
rs179830	X;HG1436_HG1432_PATCH	CLCN5	HGNC Symbol	chloride channel, voltage-sensitive 5 [Source:HGNC Symbol;Acc:2023]
rs4824819	X;HG1436_HG1432_PATCH	CLCN5	HGNC Symbol	chloride channel, voltage-sensitive 5 [Source:HGNC Symbol;Acc:2023]
rs147570790	X;HG1436_HG1432_PATCH	CLCN5	HGNC Symbol	chloride channel, voltage-sensitive 5 [Source:HGNC Symbol;Acc:2023]

SNP	Chromosome	gene name	gene source	description
rs56041343	X;HG1436_HG1432_PATCH	CLCN5	HGNC Symbol	chloride channel, voltage-sensitive 5 [Source:HGNC Symbol;Acc:2023]
rs140913229	X;HG1436_HG1432_PATCH	CLCN5	HGNC Symbol	chloride channel, voltage-sensitive 5 [Source:HGNC Symbol;Acc:2023]
rs4824497				
rs17328195	X;HG1436_HG1432_PATCH	AKAP4	HGNC Symbol	A kinase (PRKA) anchor protein 4 [Source:HGNC Symbol;Acc:374]
rs5915255	X;HG1433_PATCH	CCNB3	HGNC Symbol	cyclin B3 [Source:HGNC Symbol;Acc:18709]
rs73213612	X;HG1433_PATCH	CCNB3	HGNC Symbol	cyclin B3 [Source:HGNC Symbol;Acc:18709]
rs73213614	X;HG1433_PATCH	CCNB3	HGNC Symbol	cyclin B3 [Source:HGNC Symbol;Acc:18709]
rs5915296	X;HG1433_PATCH	CCNB3	HGNC Symbol	cyclin B3 [Source:HGNC Symbol;Acc:18709]
rs140009992	X;HG1433_PATCH	CCNB3	HGNC Symbol	cyclin B3 [Source:HGNC Symbol;Acc:18709]
rs4406560				
rs1129435	X	DGKK	HGNC Symbol	diacylglycerol kinase, kappa [Source:HGNC Symbol;Acc:32395]
rs4074320	X;HG1433_PATCH	DGKK	HGNC Symbol;UniProtKB Gene Name	diacylglycerol kinase, kappa [Source:HGNC Symbol;Acc:32395];Homo sapiens diacylglycerol kinase, kappa (DGKK), mRNA. [Source:RefSeq mRNA;Acc:NM_001013742]
rs5961179	X;HG1433_PATCH	DGKK	HGNC Symbol;UniProtKB Gene Name	diacylglycerol kinase, kappa [Source:HGNC Symbol;Acc:32395];Homo sapiens diacylglycerol kinase, kappa (DGKK), mRNA. [Source:RefSeq mRNA;Acc:NM_001013742]
rs17328222	X;HG1433_PATCH	DGKK	HGNC Symbol;UniProtKB Gene Name	diacylglycerol kinase, kappa [Source:HGNC Symbol;Acc:32395];Homo sapiens diacylglycerol kinase, kappa (DGKK), mRNA. [Source:RefSeq mRNA;Acc:NM_001013742]
rs4143304	X;HG1433_PATCH	DGKK	HGNC Symbol;UniProtKB Gene Name	diacylglycerol kinase, kappa [Source:HGNC Symbol;Acc:32395];Homo sapiens diacylglycerol kinase, kappa (DGKK), mRNA. [Source:RefSeq mRNA;Acc:NM_001013742]
rs4826630	X;HG1433_PATCH	DGKK	HGNC Symbol;UniProtKB Gene Name	diacylglycerol kinase, kappa [Source:HGNC Symbol;Acc:32395];Homo sapiens diacylglycerol kinase, kappa (DGKK), mRNA. [Source:RefSeq mRNA;Acc:NM_001013742]
rs1934179	X;HG1433_PATCH	DGKK	HGNC Symbol;UniProtKB Gene Name	diacylglycerol kinase, kappa [Source:HGNC Symbol;Acc:32395];Homo sapiens diacylglycerol kinase, kappa (DGKK), mRNA. [Source:RefSeq mRNA;Acc:NM_001013742]
rs73213621	X;HG1433_PATCH	DGKK	HGNC Symbol;UniProtKB Gene Name	diacylglycerol kinase, kappa [Source:HGNC Symbol;Acc:32395];Homo sapiens diacylglycerol kinase, kappa (DGKK), mRNA. [Source:RefSeq mRNA;Acc:NM_001013742]

SNP	Chromosome	gene name	gene source	description
rs111530092				
rs5961190				
rs73213626				
rs7889048				
rs150372708				
rs17328298				
rs4826638				
rs911090				
rs17281496	X;HG1433_PATCH	SHROOM4	HGNC Symbol	shroom family member 4 [Source:HGNC Symbol;Acc:29215]
rs28362303	X;HG1433_PATCH	SHROOM4	HGNC Symbol	shroom family member 4 [Source:HGNC Symbol;Acc:29215]
rs5915279	X;HG1433_PATCH	SHROOM4	HGNC Symbol	shroom family member 4 [Source:HGNC Symbol;Acc:29215]
rs5915280	X;HG1433_PATCH	SHROOM4	HGNC Symbol	shroom family member 4 [Source:HGNC Symbol;Acc:29215]
rs3747282	X;HG1433_PATCH	SHROOM4	HGNC Symbol	shroom family member 4 [Source:HGNC Symbol;Acc:29215]
rs7066803	X;HG1433_PATCH	SHROOM4	HGNC Symbol	shroom family member 4 [Source:HGNC Symbol;Acc:29215]
rs5915291	X;HG1433_PATCH	SHROOM4	HGNC Symbol	shroom family member 4 [Source:HGNC Symbol;Acc:29215]
rs7058894	X;HG1433_PATCH	SHROOM4	HGNC Symbol	shroom family member 4 [Source:HGNC Symbol;Acc:29215]
rs12010533	X;HG1433_PATCH	SHROOM4	HGNC Symbol	shroom family member 4 [Source:HGNC Symbol;Acc:29215]
rs55660620	X;HG1433_PATCH	SHROOM4	HGNC Symbol	shroom family member 4 [Source:HGNC Symbol;Acc:29215]
rs9887134	X;HG1433_PATCH	SHROOM4	HGNC Symbol	shroom family member 4 [Source:HGNC Symbol;Acc:29215]
rs73199931	X;HG1433_PATCH	SHROOM4	HGNC Symbol	shroom family member 4 [Source:HGNC Symbol;Acc:29215]
rs73199935	X;HG1433_PATCH	SHROOM4	HGNC Symbol	shroom family member 4 [Source:HGNC Symbol;Acc:29215]
rs73199936	X;HG1433_PATCH	SHROOM4	HGNC Symbol	shroom family member 4 [Source:HGNC Symbol;Acc:29215]
rs137977824	X;HG1433_PATCH	SHROOM4	HGNC Symbol	shroom family member 4 [Source:HGNC Symbol;Acc:29215]

SNP	Chromosome	gene name	gene source	description
rs6614579				
rs146933587				
rs2382650				
rs112296322				
rs2211222				
rs5915342				
rs952422				
rs17249566				
rs112296970				
rs3897937	X;HG1433_PATCH	BMP15	HGNC Symbol	bone morphogenetic protein 15 [Source:HGNC Symbol;Acc:1068]
rs73488027	X;HG1433_PATCH	BMP15	HGNC Symbol	bone morphogenetic protein 15 [Source:HGNC Symbol;Acc:1068]
rs56294496				
rs112422691				
rs1418020				
rs28595006				
rs5915383				
rs12559498				
rs73199998				
rs4826623				
rs5915395				
rs5915237				
rs12850774				
rs2382900	X;HG1433_PATCH	RP11-104D21.2	Clone-based (Vega) gene	
rs4486337	X	RP11-104D21.3	Clone-based (Vega) gene	
rs73492286	X	RP11-104D21.3	Clone-based (Vega) gene	
rs6418032				
rs73202116				
rs73202119				
rs6614658				
rs5987459				

SNP	Chromosome	gene name	gene source	description
rs1151696				
rs55751353				
rs908864				
rs5945601				
rs1144828				
rs1144829				
rs73202137				
rs12833678				
rs73202144	X;HG1433_PATCH	RP11-348F1.3	Clone-based (Vega) gene	
rs146569811	X;HG1433_PATCH	RP11-348F1.2	Clone-based (Vega) gene	
rs2134677	X;HG1433_PATCH	RP11-348F1.2	Clone-based (Vega) gene	
rs73202155	X;HG1433_PATCH	RP11-348F1.2	Clone-based (Vega) gene	
rs5945604	X;HG1433_PATCH	RP11-348F1.2	Clone-based (Vega) gene	
rs62596010	X;HG1433_PATCH	RP11-348F1.2	Clone-based (Vega) gene	
rs5987395				
rs1327301				
rs5945572				
rs5945619				
rs1891702	X;HG1433_PATCH	RP11-56H2.2	Clone-based (Vega) gene	
rs5945637				
rs1110404				
rs1936037				
rs17003307				
rs5945652				
rs5945654				
rs56342212				
rs12558898				
rs113546594				

SNP	Chromosome	gene name	gene source	description
rs5951102				
rs73204551				
rs111396373				
rs150767800				
rs6614327	X;HG1433_PATCH	MAGED1	HGNC Symbol	melanoma antigen family D, 1 [Source:HGNC Symbol;Acc:6813]
rs139658451	X;HG1433_PATCH	MAGED1	HGNC Symbol	melanoma antigen family D, 1 [Source:HGNC Symbol;Acc:6813]
rs112841851	X;HG1433_PATCH	MAGED1	HGNC Symbol	melanoma antigen family D, 1 [Source:HGNC Symbol;Acc:6813]
rs3199687	X	MAGED1	HGNC Symbol	melanoma antigen family D, 1 [Source:HGNC Symbol;Acc:6813]
rs5991756				
rs4131729				
rs141061448				
rs4129866				
rs4986573				
rs113025824				
rs12846126				
rs145117130				
rs62597512				
rs145814295				
rs141219156				
rs111821352				
rs12394834				
rs11795843				
rs4893622				
rs7049569				
rs190928294				
rs56329235				
rs73206431				
rs5986164				
rs145485164				
rs62597211				
rs5943723				

SNP	Chromosome	gene name	gene source	description
rs2806837	X	SPANXN5	HGNC Symbol	SPANX family, member N5 [Source:HGNC Symbol;Acc:33178]
rs2806838	X;HG1433_PATCH	SPANXN5	HGNC Symbol	SPANX family, member N5 [Source:HGNC Symbol;Acc:33178]
rs2807014				
rs73206445				
rs111932898				
rs17002009				
rs4986585	X	FAM156A	HGNC Symbol	family with sequence similarity 156, member A [Source:HGNC Symbol;Acc:30114]
rs111950356				
rs5951170				
rs55804907				
rs6643593				
rs5951155				
rs73208426				
rs73208442				
rs13440965				
rs73634276	X;HG1433_PATCH	GPR173	HGNC Symbol	G protein-coupled receptor 173 [Source:HGNC Symbol;Acc:18186]
rs55850908	X;HG1433_PATCH	GPR173	HGNC Symbol	G protein-coupled receptor 173 [Source:HGNC Symbol;Acc:18186]
rs139768820	X;HG1433_PATCH	GPR173;RP1-290F12.3	HGNC Symbol;Clone-based (Vega) gene	G protein-coupled receptor 173 [Source:HGNC Symbol;Acc:18186];
rs17850567	X;HG1433_PATCH	GPR173	HGNC Symbol	G protein-coupled receptor 173 [Source:HGNC Symbol;Acc:18186]
rs7060542	X;HG1433_PATCH	GPR173	HGNC Symbol	G protein-coupled receptor 173 [Source:HGNC Symbol;Acc:18186]
rs79703928	X;HG1433_PATCH	TSPYL2	HGNC Symbol	TSPY-like 2 [Source:HGNC Symbol;Acc:24358]
rs5933603	X;HG1433_PATCH	LINC01155	HGNC Symbol	long intergenic non-protein coding RNA 1155 [Source:HGNC Symbol;Acc:49510]
rs4830344	X;HG1433_PATCH	LINC01155	HGNC Symbol	long intergenic non-protein coding RNA 1155 [Source:HGNC Symbol;Acc:49510]
rs73208462	X;HG1433_PATCH	LINC01155	HGNC Symbol	long intergenic non-protein coding RNA 1155 [Source:HGNC Symbol;Acc:49510]
rs4532742	X;HG1433_PATCH	KDM5C	HGNC Symbol	lysine (K)-specific demethylase 5C [Source:HGNC Symbol;Acc:11114]

SNP	Chromosome	gene name	gene source	description
rs5978144	X;HG1433_PATCH	KDM5C	HGNC Symbol	lysine (K)-specific demethylase 5C [Source:HGNC Symbol;Acc:11114]
rs41308616	X;HG1433_PATCH	KDM5C	HGNC Symbol	lysine (K)-specific demethylase 5C [Source:HGNC Symbol;Acc:11114]
rs3810737				
rs12841970				
rs73492138	X;HG1433_PATCH	IQSEC2	HGNC Symbol	IQ motif and Sec7 domain 2 [Source:HGNC Symbol;Acc:29059]
rs5933536	X;HG1433_PATCH	IQSEC2	HGNC Symbol	IQ motif and Sec7 domain 2 [Source:HGNC Symbol;Acc:29059]
rs12013178	X;HG1433_PATCH	IQSEC2	HGNC Symbol	IQ motif and Sec7 domain 2 [Source:HGNC Symbol;Acc:29059]
rs4830363	X;HG1433_PATCH	IQSEC2	HGNC Symbol	IQ motif and Sec7 domain 2 [Source:HGNC Symbol;Acc:29059]
rs2315862	X	IQSEC2	HGNC Symbol	IQ motif and Sec7 domain 2 [Source:HGNC Symbol;Acc:29059]
rs17276442	X;HG1433_PATCH	IQSEC2	HGNC Symbol	IQ motif and Sec7 domain 2 [Source:HGNC Symbol;Acc:29059]
rs7050017	X;HG1433_PATCH	IQSEC2	HGNC Symbol	IQ motif and Sec7 domain 2 [Source:HGNC Symbol;Acc:29059]
rs17316052	X;HG1433_PATCH	IQSEC2	HGNC Symbol	IQ motif and Sec7 domain 2 [Source:HGNC Symbol;Acc:29059]
rs2149783	X;HG1433_PATCH	IQSEC2	HGNC Symbol	IQ motif and Sec7 domain 2 [Source:HGNC Symbol;Acc:29059]
rs880441	X;HG1433_PATCH	IQSEC2	HGNC Symbol	IQ motif and Sec7 domain 2 [Source:HGNC Symbol;Acc:29059]
rs1547218	X;HG1433_PATCH	IQSEC2	HGNC Symbol	IQ motif and Sec7 domain 2 [Source:HGNC Symbol;Acc:29059]
rs5933539				
rs5978156				
rs41304790	X;HG1433_PATCH	SMC1A	HGNC Symbol	structural maintenance of chromosomes 1A [Source:HGNC Symbol;Acc:11111]
rs1264011	X;HG1433_PATCH	SMC1A	HGNC Symbol	structural maintenance of chromosomes 1A [Source:HGNC Symbol;Acc:11111]
rs4830346	X;HG1433_PATCH	RIBC1	HGNC Symbol	RIB43A domain with coiled-coils 1 [Source:HGNC Symbol;Acc:26537]
rs1264013	X;HG1433_PATCH	RIBC1	HGNC Symbol	RIB43A domain with coiled-coils 1 [Source:HGNC Symbol;Acc:26537]
rs1264014				
rs1264018				

SNP	Chromosome	gene name	gene source	description
rs57928918				
rs142548369				
rs73210426				
rs45513301	X;HG1433_PATCH	HUWE1	HGNC Symbol;UniProtKB Gene Name	HECT, UBA and WWE domain containing 1, E3 ubiquitin protein ligase [Source:HGNC Symbol;Acc:30892];E3 ubiquitin-protein ligase HUWE1; HECT, UBA and WWE domain containing 1 [Source:UniProtKB/TrEMBL;Acc:Q5H963]
rs111723336	X;HG1433_PATCH	HUWE1	HGNC Symbol;UniProtKB Gene Name	HECT, UBA and WWE domain containing 1, E3 ubiquitin protein ligase [Source:HGNC Symbol;Acc:30892];E3 ubiquitin-protein ligase HUWE1; HECT, UBA and WWE domain containing 1 [Source:UniProtKB/TrEMBL;Acc:Q5H963]
rs17276588	X;HG1433_PATCH	HUWE1	HGNC Symbol;UniProtKB Gene Name	HECT, UBA and WWE domain containing 1, E3 ubiquitin protein ligase [Source:HGNC Symbol;Acc:30892];E3 ubiquitin-protein ligase HUWE1; HECT, UBA and WWE domain containing 1 [Source:UniProtKB/TrEMBL;Acc:Q5H963]
rs12840724	X;HG1433_PATCH	HUWE1	HGNC Symbol;UniProtKB Gene Name	HECT, UBA and WWE domain containing 1, E3 ubiquitin protein ligase [Source:HGNC Symbol;Acc:30892];E3 ubiquitin-protein ligase HUWE1; HECT, UBA and WWE domain containing 1 [Source:UniProtKB/TrEMBL;Acc:Q5H963]
rs62617499	X;HG1433_PATCH	HUWE1	HGNC Symbol;UniProtKB Gene Name	HECT, UBA and WWE domain containing 1, E3 ubiquitin protein ligase [Source:HGNC Symbol;Acc:30892];E3 ubiquitin-protein ligase HUWE1; HECT, UBA and WWE domain containing 1 [Source:UniProtKB/TrEMBL;Acc:Q5H963]
rs145409781	X;HG1433_PATCH	HUWE1	HGNC Symbol;UniProtKB Gene Name	HECT, UBA and WWE domain containing 1, E3 ubiquitin protein ligase [Source:HGNC Symbol;Acc:30892];E3 ubiquitin-protein ligase HUWE1; HECT, UBA and WWE domain containing 1 [Source:UniProtKB/TrEMBL;Acc:Q5H963]
rs6638360	X;HG1433_PATCH	HUWE1	HGNC Symbol;UniProtKB Gene Name	HECT, UBA and WWE domain containing 1, E3 ubiquitin protein ligase [Source:HGNC Symbol;Acc:30892];E3 ubiquitin-protein ligase HUWE1; HECT, UBA and WWE domain containing 1 [Source:UniProtKB/TrEMBL;Acc:Q5H963]
rs1858002	X;HG1433_PATCH	HUWE1	HGNC Symbol;UniProtKB Gene Name	HECT, UBA and WWE domain containing 1, E3 ubiquitin protein ligase [Source:HGNC Symbol;Acc:30892];E3 ubiquitin-protein ligase HUWE1; HECT, UBA and WWE domain containing 1 [Source:UniProtKB/TrEMBL;Acc:Q5H963]
rs41307640	X;HG1433_PATCH	HUWE1	HGNC Symbol;UniProtKB Gene Name	HECT, UBA and WWE domain containing 1, E3 ubiquitin protein ligase [Source:HGNC Symbol;Acc:30892];E3 ubiquitin-protein ligase HUWE1; HECT, UBA and WWE domain containing 1 [Source:UniProtKB/TrEMBL;Acc:Q5H963]
rs73210437	X;HG1433_PATCH	HUWE1	HGNC Symbol;UniProtKB Gene Name	HECT, UBA and WWE domain containing 1, E3 ubiquitin protein ligase [Source:HGNC Symbol;Acc:30892];E3 ubiquitin-protein ligase HUWE1; HECT, UBA and WWE domain containing 1 [Source:UniProtKB/TrEMBL;Acc:Q5H963]
rs138963374				
rs12853099				
rs6638366				
rs12842443				
rs56769397				
rs12380898				

SNP	Chromosome	gene name	gene source	description
rs9779962				
rs9803280				
rs11091317				
rs12690009				
rs5961051				
rs12556165				
rs6614240				
rs140267847				
rs5961108				
rs7877755				
rs137857730				
rs57648118				
rs28687633				
rs55699394	X;HG1433_PATCH	PHF8	HGNC Symbol	PHD finger protein 8 [Source:HGNC Symbol;Acc:20672]
rs112576131	X;HG1433_PATCH	PHF8	HGNC Symbol	PHD finger protein 8 [Source:HGNC Symbol;Acc:20672]
rs148215758	X;HG1433_PATCH	PHF8	HGNC Symbol	PHD finger protein 8 [Source:HGNC Symbol;Acc:20672]
rs146948401	X;HG1433_PATCH	PHF8	HGNC Symbol	PHD finger protein 8 [Source:HGNC Symbol;Acc:20672]
rs139494182	X;HG1433_PATCH	PHF8	HGNC Symbol	PHD finger protein 8 [Source:HGNC Symbol;Acc:20672]
rs17002477	X;HG1433_PATCH	PHF8	HGNC Symbol	PHD finger protein 8 [Source:HGNC Symbol;Acc:20672]
rs5960612	X;HG1433_PATCH	PHF8	HGNC Symbol	PHD finger protein 8 [Source:HGNC Symbol;Acc:20672]
rs68148941	X;HG1433_PATCH	PHF8	HGNC Symbol	PHD finger protein 8 [Source:HGNC Symbol;Acc:20672]
rs142143634				
rs45552536	X;HG1433_PATCH	FAM120C	HGNC Symbol	family with sequence similarity 120C [Source:HGNC Symbol;Acc:16949]
rs113782816	X;HG1433_PATCH	FAM120C	HGNC Symbol	family with sequence similarity 120C [Source:HGNC Symbol;Acc:16949]
rs41304786	X;HG1433_PATCH	FAM120C	HGNC Symbol	family with sequence similarity 120C [Source:HGNC Symbol;Acc:16949]

SNP	Chromosome	gene name	gene source	description
rs145731815	X;HG1433_PATCH	FAM120C	HGNC Symbol	family with sequence similarity 120C [Source:HGNC Symbol;Acc:16949]
rs149595825	X;HG1433_PATCH	FAM120C	HGNC Symbol	family with sequence similarity 120C [Source:HGNC Symbol;Acc:16949]
rs111339334	X;HG1433_PATCH	WNK3	HGNC Symbol	WNK lysine deficient protein kinase 3 [Source:HGNC Symbol;Acc:14543]
rs55998917	X;HG1433_PATCH	WNK3	HGNC Symbol	WNK lysine deficient protein kinase 3 [Source:HGNC Symbol;Acc:14543]
rs139433785	X;HG1433_PATCH	WNK3	HGNC Symbol	WNK lysine deficient protein kinase 3 [Source:HGNC Symbol;Acc:14543]
rs3021278	X;HG1433_PATCH	WNK3	HGNC Symbol	WNK lysine deficient protein kinase 3 [Source:HGNC Symbol;Acc:14543]
rs3021280	X;HG1433_PATCH	WNK3	HGNC Symbol	WNK lysine deficient protein kinase 3 [Source:HGNC Symbol;Acc:14543]
rs66888684				
rs5961056				
rs34953337				
rs113324008				
rs17002370				
rs7890290				
rs721003				
rs5961064				
rs6521768	X	FGD1	HGNC Symbol	FYVE, RhoGEF and PH domain containing 1 [Source:HGNC Symbol;Acc:3663]
rs12015001				
rs73212528	X	GNL3L	HGNC Symbol	guanine nucleotide binding protein-like 3 (nucleolar)-like [Source:HGNC Symbol;Acc:25553]
rs6612164	X	GNL3L	HGNC Symbol	guanine nucleotide binding protein-like 3 (nucleolar)-like [Source:HGNC Symbol;Acc:25553]
rs62618081	X	GNL3L	HGNC Symbol	guanine nucleotide binding protein-like 3 (nucleolar)-like [Source:HGNC Symbol;Acc:25553]
rs148325530				
rs28853084				
rs5914147				
rs137938875				
rs34217659				
rs12389474				
rs35287488				
rs5914148				

SNP	Chromosome	gene name	gene source	description
rs141052341				
rs5915165				
rs12558522				
rs12387987				
rs73212560	X	ITIH6	HGNC Symbol	inter-alpha-trypsin inhibitor heavy chain family, member 6 [Source:HGNC Symbol;Acc:28907]
rs17316505	X	ITIH6	HGNC Symbol	inter-alpha-trypsin inhibitor heavy chain family, member 6 [Source:HGNC Symbol;Acc:28907]
rs62615624	X	ITIH6	HGNC Symbol	inter-alpha-trypsin inhibitor heavy chain family, member 6 [Source:HGNC Symbol;Acc:28907]
rs1043031	X	MAGED2	HGNC Symbol	melanoma antigen family D, 2 [Source:HGNC Symbol;Acc:16353]
rs1043044	X	MAGED2	HGNC Symbol	melanoma antigen family D, 2 [Source:HGNC Symbol;Acc:16353]
rs141138279				
rs6611381				
rs12848349				
rs986776				
rs17250653				
rs45448595	X	TRO	HGNC Symbol	trophinin [Source:HGNC Symbol;Acc:12326]
rs2273138	X	TRO	HGNC Symbol	trophinin [Source:HGNC Symbol;Acc:12326]
rs73214613	X	PFKFB1	HGNC Symbol	6-phosphofructo-2-kinase/fructose-2,6-biphosphatase 1 [Source:HGNC Symbol;Acc:8872]
rs28382694	X	APEX2	HGNC Symbol	APEX nuclease (apurinic/apyrimidinic endonuclease) 2 [Source:HGNC Symbol;Acc:17889]
rs45468097	X	ALAS2	HGNC Symbol	aminolevulinate, delta-, synthase 2 [Source:HGNC Symbol;Acc:397]
rs17250674	X	ALAS2	HGNC Symbol	aminolevulinate, delta-, synthase 2 [Source:HGNC Symbol;Acc:397]
rs45479691	X	ALAS2	HGNC Symbol	aminolevulinate, delta-, synthase 2 [Source:HGNC Symbol;Acc:397]
rs73214618				
rs954958				
rs5914251				
rs5913791	X	FAM104B	HGNC Symbol	family with sequence similarity 104, member B [Source:HGNC Symbol;Acc:25085]
rs5960402	X	MTRNR2L10	HGNC Symbol	MT-RNR2-like 10 [Source:HGNC Symbol;Acc:37167]
rs10521478	X	MTRNR2L10	HGNC Symbol	MT-RNR2-like 10 [Source:HGNC Symbol;Acc:37167]
rs5914272	X	PAGE5	HGNC Symbol	P antigen family, member 5 (prostate associated) [Source:HGNC Symbol;Acc:29992]
rs5914273	X	PAGE5	HGNC Symbol	P antigen family, member 5 (prostate associated) [Source:HGNC Symbol;Acc:29992]
rs5913807	X	PAGE3	HGNC Symbol	P antigen family, member 3 (prostate associated) [Source:HGNC Symbol;Acc:4110]
rs4826381	X	PAGE3	HGNC Symbol	P antigen family, member 3 (prostate associated) [Source:HGNC Symbol;Acc:4110]
rs35528118				

SNP	Chromosome	gene name	gene source	description
rs2375091	X	RP11-382F24.2;RP11-382F24.1	Clone-based (Vega) gene	
rs138216499				
rs1927307				
rs5914315				
rs7892090				
rs4826280				
rs57620007				
rs17250872				
rs11545211	X	MAGEH1	HGNC Symbol	melanoma antigen family H, 1 [Source:HGNC Symbol;Acc:24092]
rs3126254				
rs28446189				
rs3126255	X	USP51	HGNC Symbol	ubiquitin specific peptidase 51 [Source:HGNC Symbol;Acc:23086]
rs111374245				
rs10521479				
rs3126241				
rs2375465	X	FOXR2	HGNC Symbol	forkhead box R2 [Source:HGNC Symbol;Acc:30469]
rs149700928				
rs146106389				
rs5913856	X	RRAGB	HGNC Symbol	Ras-related GTP binding B [Source:HGNC Symbol;Acc:19901]
rs1007153	X	RRAGB	HGNC Symbol	Ras-related GTP binding B [Source:HGNC Symbol;Acc:19901]
rs139472360				
rs138565687				
rs5914476				
rs6521315				
rs73202599				
rs10855058	X	RP13-188A5.1	Clone-based (Vega) gene	
rs6417935	X	RP13-188A5.1	Clone-based (Vega) gene	
rs74800947	X	RP13-188A5.1	Clone-based (Vega) gene	
rs17251083	X	RP13-188A5.1	Clone-based (Vega) gene	

SNP	Chromosome	gene name	gene source	description
rs7885685	X	RP13-188A5.1	Clone-based (Vega) gene	
rs73204713	X	RP13-188A5.1	Clone-based (Vega) gene	
rs2104871				
rs12842703				
rs7887731				
rs17251126				
rs6612472				
rs4826461				
rs73206583	X	KLF8	HGNC Symbol	Kruppel-like factor 8 [Source:HGNC Symbol;Acc:6351]
rs145719980	X	KLF8	HGNC Symbol	Kruppel-like factor 8 [Source:HGNC Symbol;Acc:6351]
rs3922927	X	KLF8	HGNC Symbol	Kruppel-like factor 8 [Source:HGNC Symbol;Acc:6351]
rs11797159				
rs6521388				
rs7876062				
rs147005610				
rs5913935				
rs73206595				
rs1332731				
rs73208812				
rs112771446				
rs62611929				
rs73208820				
rs73210843				
rs1336048				
rs1571970				
rs721963				
rs142753470				
rs7057007				
rs145692111				
rs73210857				
rs6521472				
rs766912				

SNP	Chromosome	gene name	gene source	description
rs6612589				
rs4364764				
rs140457400				
rs5914806				
rs5914812				
rs5914815				
rs5960820				
rs5960823				
rs56363232				
rs5960832				
rs12858633				
rs5914035	X	SPIN3	HGNC Symbol	spindlin family, member 3 [Source:HGNC Symbol;Acc:27272]
rs912956	X	SPIN3	HGNC Symbol	spindlin family, member 3 [Source:HGNC Symbol;Acc:27272]
rs73216480				
rs5914052				
rs5960927				
rs75278260				
rs139470886				
rs6611612	X	FAAH2	HGNC Symbol	fatty acid amide hydrolase 2 [Source:HGNC Symbol;Acc:26440]
rs1897320	X	FAAH2	HGNC Symbol	fatty acid amide hydrolase 2 [Source:HGNC Symbol;Acc:26440]
rs148544750	X	FAAH2	HGNC Symbol	fatty acid amide hydrolase 2 [Source:HGNC Symbol;Acc:26440]
rs73224076	X	FAAH2	HGNC Symbol	fatty acid amide hydrolase 2 [Source:HGNC Symbol;Acc:26440]
rs2060113	X	FAAH2	HGNC Symbol	fatty acid amide hydrolase 2 [Source:HGNC Symbol;Acc:26440]
rs1594503	X	FAAH2	HGNC Symbol	fatty acid amide hydrolase 2 [Source:HGNC Symbol;Acc:26440]
rs1048358	X	FAAH2	HGNC Symbol	fatty acid amide hydrolase 2 [Source:HGNC Symbol;Acc:26440]
rs148950097				
rs1997715	X	ZXDB	HGNC Symbol	zinc finger, X-linked, duplicated B [Source:HGNC Symbol;Acc:13199]
rs141604862				
rs73226027				
rs62598121				
rs17251419				
rs73226044				
rs62598159				

SNP	Chromosome	gene name	gene source	description
rs73226048				
rs55950555				
rs73209409				
rs73209413				
rs56355347				
rs5989004				
rs4625204				
rs139914909				
rs2942863				
rs62596709				
rs7474182				
rs2942875				
rs73520509				
rs4384157				
rs113183112				
rs111413275				
rs149045274				
rs66513141				
rs112064215				
rs62596823				
rs60576970	X	RP11-3D23.1	Clone-based (Vega) gene	
rs6418403				
rs140914450	X	MTND1P31	HGNC Symbol	MT-ND1 pseudogene 31 [Source:HGNC Symbol;Acc:42080]
rs7886200				
rs11093962				
rs73223245				
rs10218222				
rs62597967				
rs141219727				
rs62597976				
rs17842839				
rs17251426				

SNP	Chromosome	gene name	gene source	description
rs17251433				
rs111360463				
rs139675312				
rs72623427				
rs56329621				
rs1221064				
rs73225260	X;HG1437_PATCH	RP11-357C3.3	Clone-based (Vega) gene	
rs7882672				
rs78475991	X;HG1437_PATCH	ARHGEF9	HGNC Symbol	Cdc42 guanine nucleotide exchange factor (GEF) 9 [Source:HGNC Symbol;Acc:14561]
rs139113137	X;HG1437_PATCH	ARHGEF9	HGNC Symbol	Cdc42 guanine nucleotide exchange factor (GEF) 9 [Source:HGNC Symbol;Acc:14561]
rs56259331	X;HG1437_PATCH	ARHGEF9	HGNC Symbol	Cdc42 guanine nucleotide exchange factor (GEF) 9 [Source:HGNC Symbol;Acc:14561]
rs5964688	X;HG1437_PATCH	ARHGEF9	HGNC Symbol	Cdc42 guanine nucleotide exchange factor (GEF) 9 [Source:HGNC Symbol;Acc:14561]
rs140385304	X;HG1437_PATCH	ARHGEF9	HGNC Symbol	Cdc42 guanine nucleotide exchange factor (GEF) 9 [Source:HGNC Symbol;Acc:14561]
rs73225269	X;HG1437_PATCH	ARHGEF9	HGNC Symbol	Cdc42 guanine nucleotide exchange factor (GEF) 9 [Source:HGNC Symbol;Acc:14561]
rs969630				
rs17301101				
rs35611706				
rs113655342				
rs73227114				
rs113279342				
rs144255958				
rs73227127				
rs62611780				
rs5964740	X	AMER1	HGNC Symbol	APC membrane recruitment protein 1 [Source:HGNC Symbol;Acc:26837]
rs73227160	X	MTMR8	HGNC Symbol	myotubularin related protein 8 [Source:HGNC Symbol;Acc:16825]
rs12560201	X	MTMR8	HGNC Symbol	myotubularin related protein 8 [Source:HGNC Symbol;Acc:16825]
rs1296019	X	MTMR8	HGNC Symbol	myotubularin related protein 8 [Source:HGNC Symbol;Acc:16825]
rs4462043				

SNP	Chromosome	gene name	gene source	description
rs146112277				
rs150501108				
rs12557468				
rs73211275				
rs17311245				
rs73211282				
rs1094421				
rs140289662				
rs73211289				
rs5918888				
rs113082631				
rs143612993				
rs5964866				
rs17216169				
rs1547338				
rs17301297	X	ZC4H2	HGNC Symbol	zinc finger, C4H2 domain containing [Source:HGNC Symbol;Acc:24931]
rs17216225	X	ZC4H2	HGNC Symbol	zinc finger, C4H2 domain containing [Source:HGNC Symbol;Acc:24931]
rs150192797	X	ZC4H2	HGNC Symbol	zinc finger, C4H2 domain containing [Source:HGNC Symbol;Acc:24931]
rs144558257				
rs150305441				
rs7064929				
rs62610373				
rs6624142	X	RP11-231N9.1	Clone-based (Vega) gene	
rs112478629				
rs35543298				
rs5964963				
rs45588031	X	MSN	HGNC Symbol	moesin [Source:HGNC Symbol;Acc:7373]
rs73213355				
rs12851290				
rs5965019	X	NANOGP9	HGNC Symbol	Nanog homeobox pseudogene 9 [Source:HGNC Symbol;Acc:23107]
rs144202550				
rs17216505				

SNP	Chromosome	gene name	gene source	description
rs142959525				
rs17249650				
rs62610407				
rs140819855				
rs3848896				
rs1152311				
rs7056244				
rs140989980				
rs1044165	X	VSIG4	HGNC Symbol	V-set and immunoglobulin domain containing 4 [Source:HGNC Symbol;Acc:17032]
rs17315645	X	VSIG4	HGNC Symbol	V-set and immunoglobulin domain containing 4 [Source:HGNC Symbol;Acc:17032]
rs9887348	X	VSIG4	HGNC Symbol	V-set and immunoglobulin domain containing 4 [Source:HGNC Symbol;Acc:17032]
rs678475				
rs17216533				
rs5918586				
rs11795802				
rs5919015	X	HEPH	HGNC Symbol	hephaestin [Source:HGNC Symbol;Acc:4866]
rs35835670	X	HEPH	HGNC Symbol	hephaestin [Source:HGNC Symbol;Acc:4866]
rs17216603	X	HEPH	HGNC Symbol	hephaestin [Source:HGNC Symbol;Acc:4866]
rs1264215	X	HEPH	HGNC Symbol	hephaestin [Source:HGNC Symbol;Acc:4866]
rs111873599	X	HEPH	HGNC Symbol	hephaestin [Source:HGNC Symbol;Acc:4866]
rs806607	X	HEPH	HGNC Symbol	hephaestin [Source:HGNC Symbol;Acc:4866]
rs809363	X	HEPH	HGNC Symbol	hephaestin [Source:HGNC Symbol;Acc:4866]
rs1090752	X	HEPH	HGNC Symbol	hephaestin [Source:HGNC Symbol;Acc:4866]
rs12690403				
rs150082860				
rs5919049				
rs11799032				
rs145867342				
rs112069404				
rs6624911				
rs5965182				
rs56373032				
rs1926338				

SNP	Chromosome	gene name	gene source	description
rs79798752				
rs5965192				
rs5919092				
rs62612637				
rs140229083				
rs137906178				
rs5919120				
rs12556663				
rs113989766				
rs73219599				
rs145957578				
rs5919159				
rs1385699	X	EDA2R	HGNC Symbol	ectodysplasin A2 receptor [Source:HGNC Symbol;Acc:17756]
rs148686312	X	EDA2R	HGNC Symbol	ectodysplasin A2 receptor [Source:HGNC Symbol;Acc:17756]
rs111727221				
rs73221553				
rs73221556				
rs775366				
rs111424077				
rs73221587				
rs111430255				
rs140516259				
rs149483793				
rs73223708				
rs145017238				
rs4240053				
rs5919235				
rs112712390				
rs59003621				
rs471205				
rs112360029				
rs1606095				
rs73632187				

SNP	Chromosome	gene name	gene source	description
rs4827528				
rs5919274				
rs17216820				
rs5919275				
rs149242619				
rs73225832				
rs34191540				
rs5919340				
rs12558842				
rs6624290				
rs17301965				
rs6625163				
rs142169094				
rs2497938				
rs73227823				
rs34665307				
rs5918750				
rs62604342				
rs112576028				
rs12557549				
rs962458				
rs7888856				
rs148292912	X	AR	HGNC Symbol	androgen receptor [Source:HGNC Symbol;Acc:644]
rs2361634	X	AR	HGNC Symbol	androgen receptor [Source:HGNC Symbol;Acc:644]
rs6624304	X	AR	HGNC Symbol	androgen receptor [Source:HGNC Symbol;Acc:644]
rs113044988	X	AR	HGNC Symbol	androgen receptor [Source:HGNC Symbol;Acc:644]
rs73227899				
rs139678949				
rs5919427				
rs5919432				
rs4022202				
rs142188276				
rs61098894				

SNP	Chromosome	gene name	gene source	description
rs60750995				
rs73212804				
rs5965476				
rs146376239				
rs145131650				
rs17302236				
rs4562482				
rs62604532				
rs2765951				
rs7888212	X	OPHN1	HGNC Symbol	oligophrenin 1 [Source:HGNC Symbol;Acc:8148]
rs5918807	X	OPHN1	HGNC Symbol	oligophrenin 1 [Source:HGNC Symbol;Acc:8148]
rs143483183	X	OPHN1	HGNC Symbol	oligophrenin 1 [Source:HGNC Symbol;Acc:8148]
rs12558557	X	OPHN1	HGNC Symbol	oligophrenin 1 [Source:HGNC Symbol;Acc:8148]
rs5965497	X	OPHN1	HGNC Symbol	oligophrenin 1 [Source:HGNC Symbol;Acc:8148]
rs7061504	X	OPHN1	HGNC Symbol	oligophrenin 1 [Source:HGNC Symbol;Acc:8148]
rs62607069	X	OPHN1	HGNC Symbol	oligophrenin 1 [Source:HGNC Symbol;Acc:8148]
rs79383505	X	OPHN1	HGNC Symbol	oligophrenin 1 [Source:HGNC Symbol;Acc:8148]
rs62607098	X	OPHN1	HGNC Symbol	oligophrenin 1 [Source:HGNC Symbol;Acc:8148]
rs73212857	X	OPHN1	HGNC Symbol	oligophrenin 1 [Source:HGNC Symbol;Acc:8148]
rs12836444	X	OPHN1	HGNC Symbol	oligophrenin 1 [Source:HGNC Symbol;Acc:8148]
rs145403307	X	OPHN1	HGNC Symbol	oligophrenin 1 [Source:HGNC Symbol;Acc:8148]
rs111365012	X	OPHN1	HGNC Symbol	oligophrenin 1 [Source:HGNC Symbol;Acc:8148]
rs2768811	X	OPHN1	HGNC Symbol	oligophrenin 1 [Source:HGNC Symbol;Acc:8148]
rs6625282	X	OPHN1	HGNC Symbol	oligophrenin 1 [Source:HGNC Symbol;Acc:8148]
rs73212868	X	OPHN1	HGNC Symbol	oligophrenin 1 [Source:HGNC Symbol;Acc:8148]
rs2225124	X	OPHN1	HGNC Symbol	oligophrenin 1 [Source:HGNC Symbol;Acc:8148]
rs5965519	X	OPHN1	HGNC Symbol	oligophrenin 1 [Source:HGNC Symbol;Acc:8148]
rs5965529	X	OPHN1	HGNC Symbol	oligophrenin 1 [Source:HGNC Symbol;Acc:8148]
rs73212878	X	OPHN1	HGNC Symbol	oligophrenin 1 [Source:HGNC Symbol;Acc:8148]
rs140488081	X	OPHN1	HGNC Symbol	oligophrenin 1 [Source:HGNC Symbol;Acc:8148]
rs7053742	X	OPHN1	HGNC Symbol	oligophrenin 1 [Source:HGNC Symbol;Acc:8148]
rs150294060	X	OPHN1	HGNC Symbol	oligophrenin 1 [Source:HGNC Symbol;Acc:8148]
rs5918825	X	OPHN1	HGNC Symbol	oligophrenin 1 [Source:HGNC Symbol;Acc:8148]

SNP	Chromosome	gene name	gene source	description
rs11796608	X	OPHN1	HGNC Symbol	oligophrenin 1 [Source:HGNC Symbol;Acc:8148]
rs73539208	X	OPHN1	HGNC Symbol	oligophrenin 1 [Source:HGNC Symbol;Acc:8148]
rs7062425	X	OPHN1	HGNC Symbol	oligophrenin 1 [Source:HGNC Symbol;Acc:8148]
rs73212884	X	OPHN1	HGNC Symbol	oligophrenin 1 [Source:HGNC Symbol;Acc:8148]
rs5965561	X	OPHN1	HGNC Symbol	oligophrenin 1 [Source:HGNC Symbol;Acc:8148]
rs6625312	X	OPHN1	HGNC Symbol	oligophrenin 1 [Source:HGNC Symbol;Acc:8148]
rs5919550	X	OPHN1	HGNC Symbol	oligophrenin 1 [Source:HGNC Symbol;Acc:8148]
rs150651949	X	OPHN1	HGNC Symbol	oligophrenin 1 [Source:HGNC Symbol;Acc:8148]
rs139761338	X	OPHN1	HGNC Symbol	oligophrenin 1 [Source:HGNC Symbol;Acc:8148]
rs151229760	X	OPHN1	HGNC Symbol	oligophrenin 1 [Source:HGNC Symbol;Acc:8148]
rs5919559	X	OPHN1	HGNC Symbol	oligophrenin 1 [Source:HGNC Symbol;Acc:8148]
rs41303733	X	OPHN1	HGNC Symbol	oligophrenin 1 [Source:HGNC Symbol;Acc:8148]
rs5919571				
rs5919577				
rs140506878				
rs7055305	X	YIPF6	HGNC Symbol	Yip1 domain family, member 6 [Source:HGNC Symbol;Acc:28304]
rs6525241	X	YIPF6	HGNC Symbol	Yip1 domain family, member 6 [Source:HGNC Symbol;Acc:28304]
rs45508695	X	YIPF6	HGNC Symbol	Yip1 domain family, member 6 [Source:HGNC Symbol;Acc:28304]
rs5964684				
rs4240815				
rs7886577				
rs35900152				
rs12010994				
rs3935804				
rs4609344				
rs7886230	X	STARD8	HGNC Symbol	StAR-related lipid transfer (START) domain containing 8 [Source:HGNC Symbol;Acc:19161]
rs112479767	X	STARD8	HGNC Symbol	StAR-related lipid transfer (START) domain containing 8 [Source:HGNC Symbol;Acc:19161]
rs5936716	X	STARD8	HGNC Symbol	StAR-related lipid transfer (START) domain containing 8 [Source:HGNC Symbol;Acc:19161]
rs6625359	X	STARD8	HGNC Symbol	StAR-related lipid transfer (START) domain containing 8 [Source:HGNC Symbol;Acc:19161]
rs60325541	X	STARD8	HGNC Symbol	StAR-related lipid transfer (START) domain containing 8 [Source:HGNC Symbol;Acc:19161]
rs12841264	X	STARD8	HGNC Symbol	StAR-related lipid transfer (START) domain containing 8 [Source:HGNC Symbol;Acc:19161]
rs62604444	X	STARD8	HGNC Symbol	StAR-related lipid transfer (START) domain containing 8 [Source:HGNC Symbol;Acc:19161]
rs4844101	X	STARD8	HGNC Symbol	StAR-related lipid transfer (START) domain containing 8 [Source:HGNC Symbol;Acc:19161]

SNP	Chromosome	gene name	gene source	description
rs6525255	X	STARD8	HGNC Symbol	StAR-related lipid transfer (START) domain containing 8 [Source:HGNC Symbol;Acc:19161]
rs56008802	X	STARD8	HGNC Symbol	StAR-related lipid transfer (START) domain containing 8 [Source:HGNC Symbol;Acc:19161]
rs62604446	X	STARD8	HGNC Symbol	StAR-related lipid transfer (START) domain containing 8 [Source:HGNC Symbol;Acc:19161]
rs41310627	X	STARD8	HGNC Symbol	StAR-related lipid transfer (START) domain containing 8 [Source:HGNC Symbol;Acc:19161]
rs55962426	X	STARD8	HGNC Symbol	StAR-related lipid transfer (START) domain containing 8 [Source:HGNC Symbol;Acc:19161]
rs55927581	X	STARD8	HGNC Symbol	StAR-related lipid transfer (START) domain containing 8 [Source:HGNC Symbol;Acc:19161]
rs7064080				
rs56915326				
rs73215084				
rs5980922				
rs73215085				
rs5936559				
rs792956				
rs66998265				
rs5936920				
rs73218749				
rs7065977				
rs10482102				
rs7056165				
rs66947109				
rs55698573				
rs705896				
rs12014040				
rs142053660				
rs877817	X	EFNB1	HGNC Symbol	ephrin-B1 [Source:HGNC Symbol;Acc:3226]
rs12013955	X	EFNB1	HGNC Symbol	ephrin-B1 [Source:HGNC Symbol;Acc:3226]
rs241396				
rs73218776				
rs1343286				
rs665542				
rs241391				
rs241390				
rs35488161				

SNP	Chromosome	gene name	gene source	description
rs5937118				
rs241750				
rs56317970				
rs73543624				
rs73543630				
rs73218784				
rs73218785				
rs61170274				
rs141091404				
rs1277994				
rs5936649				
rs59093237				
rs1277951				
rs73218793				
rs1277966				
rs56894983				
rs1277971				
rs4844160				
rs1777640				
rs113960727				
rs4844317				
rs73220615				
rs73220627				
rs183231451				
rs73220656				
rs2666149				
rs73220658				
rs6624381				
rs73220661				
rs17302605				
rs73220663				
rs57400154				
rs1926348				

SNP	Chromosome	gene name	gene source	description
rs7066879				
rs5981162				
rs11796961				
rs5981165				
rs1926345				
rs73528831				
rs5981167				
rs16990817				
rs5980792				
rs73528851				
rs5981172				
rs5936657				
rs73222542				
rs6624382				
rs6625402				
rs5936660				
rs5937159				
rs17217584				
rs11539157	X	PJA1	HGNC Symbol	praja ring finger 1, E3 ubiquitin protein ligase [Source:HGNC Symbol;Acc:16648]
rs5937160	X	PJA1	HGNC Symbol	praja ring finger 1, E3 ubiquitin protein ligase [Source:HGNC Symbol;Acc:16648]
rs3761646	X	PJA1	HGNC Symbol	praja ring finger 1, E3 ubiquitin protein ligase [Source:HGNC Symbol;Acc:16648]
rs7067170	X	PJA1	HGNC Symbol	praja ring finger 1, E3 ubiquitin protein ligase [Source:HGNC Symbol;Acc:16648]
rs4844165	X	PJA1	HGNC Symbol	praja ring finger 1, E3 ubiquitin protein ligase [Source:HGNC Symbol;Acc:16648]
rs17302682				
rs5937162				
rs4596804				
rs2148344	X	LINC00269	HGNC Symbol	long intergenic non-protein coding RNA 269 [Source:HGNC Symbol;Acc:26586]
rs56230427	X	LINC00269	HGNC Symbol	long intergenic non-protein coding RNA 269 [Source:HGNC Symbol;Acc:26586]
rs10856072	X	LINC00269	HGNC Symbol	long intergenic non-protein coding RNA 269 [Source:HGNC Symbol;Acc:26586]
rs1926350	X	LINC00269	HGNC Symbol	long intergenic non-protein coding RNA 269 [Source:HGNC Symbol;Acc:26586]
rs5937175	X	LINC00269	HGNC Symbol	long intergenic non-protein coding RNA 269 [Source:HGNC Symbol;Acc:26586]
rs6624391	X	LINC00269	HGNC Symbol	long intergenic non-protein coding RNA 269 [Source:HGNC Symbol;Acc:26586]
rs5937178	X	LINC00269	HGNC Symbol	long intergenic non-protein coding RNA 269 [Source:HGNC Symbol;Acc:26586]

SNP	Chromosome	gene name	gene source	description
rs6625416				
rs150558194				
rs5980801				
rs5981189				
rs7062312				
rs3849265				
rs4844335				
rs5980803				
rs11796821				
rs5936673				
rs5936674				
rs1574658				
rs12397209				
rs5981196				
rs1317200				
rs12854725				
rs4844339				
rs73222592				
rs4844341				
rs5937202				
rs55737046				
rs1888634				
rs6625441				
rs5937206				
rs17302855				
rs56402033				
rs973513				
rs5937209				
rs73224411				
rs34025676				
rs12688507				
rs942670				
rs1555776				

SNP	Chromosome	gene name	gene source	description
rs4844173				
rs1408783				
rs73224425				
rs1536062				
rs5937242				
rs6625471	X	FAM155B	HGNC Symbol	family with sequence similarity 155, member B [Source:HGNC Symbol;Acc:30701]
rs4844359	X	FAM155B	HGNC Symbol	family with sequence similarity 155, member B [Source:HGNC Symbol;Acc:30701]
rs55679031	X	FAM155B	HGNC Symbol	family with sequence similarity 155, member B [Source:HGNC Symbol;Acc:30701]
rs5980820	X	FAM155B	HGNC Symbol	family with sequence similarity 155, member B [Source:HGNC Symbol;Acc:30701]
rs12116358	X	FAM155B	HGNC Symbol	family with sequence similarity 155, member B [Source:HGNC Symbol;Acc:30701]
rs41306119	X	FAM155B	HGNC Symbol	family with sequence similarity 155, member B [Source:HGNC Symbol;Acc:30701]
rs12844850				
rs6625478				
rs150230046				
rs4844364				
rs4844365				
rs5936698				
rs4844178				
rs4844096				
rs17216260	X	EDA	HGNC Symbol	ectodysplasin A [Source:HGNC Symbol;Acc:3157]
rs62605733	X	EDA	HGNC Symbol	ectodysplasin A [Source:HGNC Symbol;Acc:3157]
rs146934198	X	EDA	HGNC Symbol	ectodysplasin A [Source:HGNC Symbol;Acc:3157]
rs5936487	X	EDA	HGNC Symbol	ectodysplasin A [Source:HGNC Symbol;Acc:3157]
rs62604169	X	EDA	HGNC Symbol	ectodysplasin A [Source:HGNC Symbol;Acc:3157]
rs2804384	X	EDA	HGNC Symbol	ectodysplasin A [Source:HGNC Symbol;Acc:3157]
rs2520387	X	EDA	HGNC Symbol	ectodysplasin A [Source:HGNC Symbol;Acc:3157]
rs146840389	X	EDA	HGNC Symbol	ectodysplasin A [Source:HGNC Symbol;Acc:3157]
rs147121494	X	EDA	HGNC Symbol	ectodysplasin A [Source:HGNC Symbol;Acc:3157]
rs2804352	X	EDA	HGNC Symbol	ectodysplasin A [Source:HGNC Symbol;Acc:3157]
rs11094136	X	EDA	HGNC Symbol	ectodysplasin A [Source:HGNC Symbol;Acc:3157]
rs5936511	X	EDA	HGNC Symbol	ectodysplasin A [Source:HGNC Symbol;Acc:3157]
rs75063666	X	EDA	HGNC Symbol	ectodysplasin A [Source:HGNC Symbol;Acc:3157]
rs1202986	X	EDA	HGNC Symbol	ectodysplasin A [Source:HGNC Symbol;Acc:3157]

SNP	Chromosome	gene name	gene source	description
rs1203008	X	EDA	HGNC Symbol	ectodysplasin A [Source:HGNC Symbol;Acc:3157]
rs73226443	X	EDA	HGNC Symbol	ectodysplasin A [Source:HGNC Symbol;Acc:3157]
rs1938029	X	EDA	HGNC Symbol	ectodysplasin A [Source:HGNC Symbol;Acc:3157]
rs73226444	X	EDA	HGNC Symbol	ectodysplasin A [Source:HGNC Symbol;Acc:3157]
rs1938023	X	EDA	HGNC Symbol	ectodysplasin A [Source:HGNC Symbol;Acc:3157]
rs35407838	X	EDA	HGNC Symbol	ectodysplasin A [Source:HGNC Symbol;Acc:3157]
rs55879809	X	EDA	HGNC Symbol	ectodysplasin A [Source:HGNC Symbol;Acc:3157]
rs5936806	X	EDA	HGNC Symbol	ectodysplasin A [Source:HGNC Symbol;Acc:3157]
rs73226452	X	EDA	HGNC Symbol	ectodysplasin A [Source:HGNC Symbol;Acc:3157]
rs6625541	X	EDA	HGNC Symbol	ectodysplasin A [Source:HGNC Symbol;Acc:3157]
rs5936809	X	EDA	HGNC Symbol	ectodysplasin A [Source:HGNC Symbol;Acc:3157]
rs6625546	X	EDA	HGNC Symbol	ectodysplasin A [Source:HGNC Symbol;Acc:3157]
rs1359837	X	EDA	HGNC Symbol	ectodysplasin A [Source:HGNC Symbol;Acc:3157]
rs5936817	X	EDA	HGNC Symbol	ectodysplasin A [Source:HGNC Symbol;Acc:3157]
rs12849039	X	EDA	HGNC Symbol	ectodysplasin A [Source:HGNC Symbol;Acc:3157]
rs5936818	X	EDA	HGNC Symbol	ectodysplasin A [Source:HGNC Symbol;Acc:3157]
rs73226481	X	EDA	HGNC Symbol	ectodysplasin A [Source:HGNC Symbol;Acc:3157]
rs62604271	X	EDA	HGNC Symbol	ectodysplasin A [Source:HGNC Symbol;Acc:3157]
rs2296765	X	EDA	HGNC Symbol	ectodysplasin A [Source:HGNC Symbol;Acc:3157]
rs6625563	X	EDA	HGNC Symbol	ectodysplasin A [Source:HGNC Symbol;Acc:3157]
rs41310621	X	EDA	HGNC Symbol	ectodysplasin A [Source:HGNC Symbol;Acc:3157]
rs11795582	X	AWAT2	HGNC Symbol	acyl-CoA wax alcohol acyltransferase 2 [Source:HGNC Symbol;Acc:23251]
rs60550878	X	AWAT2	HGNC Symbol	acyl-CoA wax alcohol acyltransferase 2 [Source:HGNC Symbol;Acc:23251]
rs55991455	X	AWAT2	HGNC Symbol	acyl-CoA wax alcohol acyltransferase 2 [Source:HGNC Symbol;Acc:23251]
rs55933614				
rs62604281				
rs12007284				
rs73228223				
rs73532918				
rs5936833				
rs607614				
rs150656555				

SNP	Chromosome	gene name	gene source	description
rs77282445	X	RP11-351K23.3	Clone-based (Vega) gene	
rs150196945	X	MTND4P31	HGNC Symbol	MT-ND4 pseudogene 31 [Source:HGNC Symbol;Acc:42218]
rs72628851				
rs59856053	X	IGBP1	HGNC Symbol	immunoglobulin (CD79A) binding protein 1 [Source:HGNC Symbol;Acc:5461]
rs149043880	X	IGBP1	HGNC Symbol	immunoglobulin (CD79A) binding protein 1 [Source:HGNC Symbol;Acc:5461]
rs5936856	X	IGBP1	HGNC Symbol	immunoglobulin (CD79A) binding protein 1 [Source:HGNC Symbol;Acc:5461]
rs1199509	X	DGAT2L6	HGNC Symbol	diacylglycerol O-acyltransferase 2-like 6 [Source:HGNC Symbol;Acc:23250]
rs7060831	X	DGAT2L6	HGNC Symbol	diacylglycerol O-acyltransferase 2-like 6 [Source:HGNC Symbol;Acc:23250]
rs5980903	X	DGAT2L6	HGNC Symbol	diacylglycerol O-acyltransferase 2-like 6 [Source:HGNC Symbol;Acc:23250]
rs111714615	X	DGAT2L6	HGNC Symbol	diacylglycerol O-acyltransferase 2-like 6 [Source:HGNC Symbol;Acc:23250]
rs1199504	X	DGAT2L6	HGNC Symbol	diacylglycerol O-acyltransferase 2-like 6 [Source:HGNC Symbol;Acc:23250]
rs1209721	X	DGAT2L6	HGNC Symbol	diacylglycerol O-acyltransferase 2-like 6 [Source:HGNC Symbol;Acc:23250]
rs12689032	X	DGAT2L6	HGNC Symbol	diacylglycerol O-acyltransferase 2-like 6 [Source:HGNC Symbol;Acc:23250]
rs73214982	X	DGAT2L6	HGNC Symbol	diacylglycerol O-acyltransferase 2-like 6 [Source:HGNC Symbol;Acc:23250]
rs5980905	X	DGAT2L6	HGNC Symbol	diacylglycerol O-acyltransferase 2-like 6 [Source:HGNC Symbol;Acc:23250]
rs113548026				
rs56080916	X	AWAT1	HGNC Symbol	acyl-CoA wax alcohol acyltransferase 1 [Source:HGNC Symbol;Acc:23252]
rs1152197	X	AWAT1	HGNC Symbol	acyl-CoA wax alcohol acyltransferase 1 [Source:HGNC Symbol;Acc:23252]
rs28577193	X	AWAT1	HGNC Symbol	acyl-CoA wax alcohol acyltransferase 1 [Source:HGNC Symbol;Acc:23252]
rs4844218				
rs41310667	X	P2RY4	HGNC Symbol	pyrimidinergic receptor P2Y, G-protein coupled, 4 [Source:HGNC Symbol;Acc:8542]
rs56217451	X	P2RY4	HGNC Symbol	pyrimidinergic receptor P2Y, G-protein coupled, 4 [Source:HGNC Symbol;Acc:8542]
rs3829708	X	P2RY4	HGNC Symbol	pyrimidinergic receptor P2Y, G-protein coupled, 4 [Source:HGNC Symbol;Acc:8542]
rs3829709	X	P2RY4	HGNC Symbol	pyrimidinergic receptor P2Y, G-protein coupled, 4 [Source:HGNC Symbol;Acc:8542]
rs1152187	X	P2RY4	HGNC Symbol	pyrimidinergic receptor P2Y, G-protein coupled, 4 [Source:HGNC Symbol;Acc:8542]
rs1152185				
rs41303717	X	ARR3	HGNC Symbol	arrestin 3, retinal (X-arrestin) [Source:HGNC Symbol;Acc:710]
rs144350357	X	ARR3	HGNC Symbol	arrestin 3, retinal (X-arrestin) [Source:HGNC Symbol;Acc:710]
rs4844220	X	ARR3	HGNC Symbol	arrestin 3, retinal (X-arrestin) [Source:HGNC Symbol;Acc:710]
rs17301749	X	ARR3	HGNC Symbol	arrestin 3, retinal (X-arrestin) [Source:HGNC Symbol;Acc:710]
rs3818861	X	ARR3	HGNC Symbol	arrestin 3, retinal (X-arrestin) [Source:HGNC Symbol;Acc:710]
rs5936549	X	RAB41	HGNC Symbol	RAB41, member RAS oncogene family [Source:HGNC Symbol;Acc:18293]

SNP	Chromosome	gene name	gene source	description
rs1713746	X	RAB41	HGNC Symbol	RAB41, member RAS oncogene family [Source:HGNC Symbol;Acc:18293]
rs1199470	X	KIF4A;PDZD11	HGNC Symbol	kinesin family member 4A [Source:HGNC Symbol;Acc:13339];PDZ domain containing 11 [Source:HGNC Symbol;Acc:28034]
rs1199469	X	KIF4A	HGNC Symbol	kinesin family member 4A [Source:HGNC Symbol;Acc:13339]
rs113372544	X	KIF4A	HGNC Symbol	kinesin family member 4A [Source:HGNC Symbol;Acc:13339]
rs62607671	X	KIF4A	HGNC Symbol	kinesin family member 4A [Source:HGNC Symbol;Acc:13339]
rs145495213	X	KIF4A	HGNC Symbol	kinesin family member 4A [Source:HGNC Symbol;Acc:13339]
rs146484329	X	KIF4A	HGNC Symbol	kinesin family member 4A [Source:HGNC Symbol;Acc:13339]
rs41306123	X	KIF4A	HGNC Symbol	kinesin family member 4A [Source:HGNC Symbol;Acc:13339]
rs1199457	X	KIF4A	HGNC Symbol	kinesin family member 4A [Source:HGNC Symbol;Acc:13339]
rs1199429	X	KIF4A	HGNC Symbol	kinesin family member 4A [Source:HGNC Symbol;Acc:13339]
rs140902523	X	KIF4A	HGNC Symbol	kinesin family member 4A [Source:HGNC Symbol;Acc:13339]
rs6625606	X	KIF4A	HGNC Symbol	kinesin family member 4A [Source:HGNC Symbol;Acc:13339]
rs5936558	X	KIF4A	HGNC Symbol	kinesin family member 4A [Source:HGNC Symbol;Acc:13339]
rs2296542	X	GDPD2	HGNC Symbol	glycerophosphodiester phosphodiesterase domain containing 2 [Source:HGNC Symbol;Acc:25974]
rs41307254	X	GDPD2	HGNC Symbol	glycerophosphodiester phosphodiesterase domain containing 2 [Source:HGNC Symbol;Acc:25974]
rs73216714				
rs62609681	X	DLG3	HGNC Symbol	discs, large homolog 3 (Drosophila) [Source:HGNC Symbol;Acc:2902]
rs41306141	X	DLG3	HGNC Symbol	discs, large homolog 3 (Drosophila) [Source:HGNC Symbol;Acc:2902]
rs12850410	X	DLG3	HGNC Symbol	discs, large homolog 3 (Drosophila) [Source:HGNC Symbol;Acc:2902]
rs73216721	X	DLG3	HGNC Symbol	discs, large homolog 3 (Drosophila) [Source:HGNC Symbol;Acc:2902]
rs73216723	X	DLG3	HGNC Symbol	discs, large homolog 3 (Drosophila) [Source:HGNC Symbol;Acc:2902]
rs4844121	X	DLG3	HGNC Symbol	discs, large homolog 3 (Drosophila) [Source:HGNC Symbol;Acc:2902]
rs12843277	X	DLG3	HGNC Symbol	discs, large homolog 3 (Drosophila) [Source:HGNC Symbol;Acc:2902]
rs5980949	X	DLG3	HGNC Symbol	discs, large homolog 3 (Drosophila) [Source:HGNC Symbol;Acc:2902]
rs111499414	X	DLG3	HGNC Symbol	discs, large homolog 3 (Drosophila) [Source:HGNC Symbol;Acc:2902]
rs41303736	X	DLG3	HGNC Symbol	discs, large homolog 3 (Drosophila) [Source:HGNC Symbol;Acc:2902]
rs3828017	X	DLG3	HGNC Symbol	discs, large homolog 3 (Drosophila) [Source:HGNC Symbol;Acc:2902]
rs12014337				
rs6525372				
rs5936905				
rs1536250	X	TEX11	HGNC Symbol	testis expressed 11 [Source:HGNC Symbol;Acc:11733]
rs62608008	X	TEX11	HGNC Symbol	testis expressed 11 [Source:HGNC Symbol;Acc:11733]

SNP	Chromosome	gene name	gene source	description
rs16991177	X	TEX11	HGNC Symbol	testis expressed 11 [Source:HGNC Symbol;Acc:11733]
rs5936929	X	TEX11	HGNC Symbol	testis expressed 11 [Source:HGNC Symbol;Acc:11733]
rs73216776	X	TEX11	HGNC Symbol	testis expressed 11 [Source:HGNC Symbol;Acc:11733]
rs146012538	X	TEX11	HGNC Symbol	testis expressed 11 [Source:HGNC Symbol;Acc:11733]
rs73542414	X	TEX11	HGNC Symbol	testis expressed 11 [Source:HGNC Symbol;Acc:11733]
rs143110708	X	TEX11	HGNC Symbol	testis expressed 11 [Source:HGNC Symbol;Acc:11733]
rs56268567	X	TEX11	HGNC Symbol	testis expressed 11 [Source:HGNC Symbol;Acc:11733]
rs5980726	X	TEX11	HGNC Symbol	testis expressed 11 [Source:HGNC Symbol;Acc:11733]
rs4844247	X	TEX11	HGNC Symbol	testis expressed 11 [Source:HGNC Symbol;Acc:11733]
rs7886580	X	TEX11	HGNC Symbol	testis expressed 11 [Source:HGNC Symbol;Acc:11733]
rs6525401	X	TEX11	HGNC Symbol	testis expressed 11 [Source:HGNC Symbol;Acc:11733]
rs5936963	X	TEX11	HGNC Symbol	testis expressed 11 [Source:HGNC Symbol;Acc:11733]
rs6653305	X	TEX11	HGNC Symbol	testis expressed 11 [Source:HGNC Symbol;Acc:11733]
rs12557911	X	TEX11	HGNC Symbol	testis expressed 11 [Source:HGNC Symbol;Acc:11733]
rs5981010	X	TEX11	HGNC Symbol	testis expressed 11 [Source:HGNC Symbol;Acc:11733]
rs182202558	X	TEX11	HGNC Symbol	testis expressed 11 [Source:HGNC Symbol;Acc:11733]
rs6525428	X	TEX11	HGNC Symbol	testis expressed 11 [Source:HGNC Symbol;Acc:11733]
rs6624506	X	TEX11	HGNC Symbol	testis expressed 11 [Source:HGNC Symbol;Acc:11733]
rs73213092	X	TEX11	HGNC Symbol	testis expressed 11 [Source:HGNC Symbol;Acc:11733]
rs6525433	X	TEX11	HGNC Symbol	testis expressed 11 [Source:HGNC Symbol;Acc:11733]
rs5937008	X	TEX11	HGNC Symbol	testis expressed 11 [Source:HGNC Symbol;Acc:11733]
rs4844274	X	TEX11	HGNC Symbol	testis expressed 11 [Source:HGNC Symbol;Acc:11733]
rs4393069	X	TEX11	HGNC Symbol	testis expressed 11 [Source:HGNC Symbol;Acc:11733]
rs4457484	X	TEX11	HGNC Symbol	testis expressed 11 [Source:HGNC Symbol;Acc:11733]
rs4360450	X	SLC7A3	HGNC Symbol	solute carrier family 7 (cationic amino acid transporter, y+ system), member 3 [Source:HGNC Symbol;Acc:11061]
rs6525447	X	SLC7A3	HGNC Symbol	solute carrier family 7 (cationic amino acid transporter, y+ system), member 3 [Source:HGNC Symbol;Acc:11061]
rs59445423	X	SLC7A3	HGNC Symbol	solute carrier family 7 (cationic amino acid transporter, y+ system), member 3 [Source:HGNC Symbol;Acc:11061]
rs7055793				
rs5937030				
rs5936612				
rs139833295				

SNP	Chromosome	gene name	gene source	description
rs12688227				
rs73634849				
rs35014580				
rs56349504				
rs12851337	X	SNX12	HGNC Symbol	sorting nexin 12 [Source:HGNC Symbol;Acc:14976]
rs5981065				
rs4393070				
rs7472797				
rs55663223				
rs2075790	X	MED12	HGNC Symbol	mediator complex subunit 12 [Source:HGNC Symbol;Acc:11957]
rs5030619	X	MED12	HGNC Symbol	mediator complex subunit 12 [Source:HGNC Symbol;Acc:11957]
rs10521349	X	MED12	HGNC Symbol	mediator complex subunit 12 [Source:HGNC Symbol;Acc:11957]
rs4844285	X	NLGN3	HGNC Symbol	neuroligin 3 [Source:HGNC Symbol;Acc:14289]
rs12846068	X	NLGN3	HGNC Symbol	neuroligin 3 [Source:HGNC Symbol;Acc:14289]
rs67640617	X	NLGN3	HGNC Symbol	neuroligin 3 [Source:HGNC Symbol;Acc:14289]
rs2503131	X	NLGN3	HGNC Symbol	neuroligin 3 [Source:HGNC Symbol;Acc:14289]
rs73217041	X	NLGN3	HGNC Symbol	neuroligin 3 [Source:HGNC Symbol;Acc:14289]
rs7886134	X	NLGN3	HGNC Symbol	neuroligin 3 [Source:HGNC Symbol;Acc:14289]
rs7055734				
rs142435968				
rs66558969				
rs140709755				
rs6624539	X	RP5-1091N2.9	Clone-based (Vega) gene	
rs190825185	X	RP5-1091N2.9	Clone-based (Vega) gene	
rs6525481				
rs7058793				
rs6525483				
rs6625772	X	GJB1	HGNC Symbol	gap junction protein, beta 1, 32kDa [Source:HGNC Symbol;Acc:4283]
rs41475447	X	GJB1	HGNC Symbol	gap junction protein, beta 1, 32kDa [Source:HGNC Symbol;Acc:4283]
rs5980746	X	GJB1	HGNC Symbol	gap junction protein, beta 1, 32kDa [Source:HGNC Symbol;Acc:4283]
rs41310607	X	GJB1	HGNC Symbol	gap junction protein, beta 1, 32kDa [Source:HGNC Symbol;Acc:4283]
rs2235132				

SNP	Chromosome	gene name	gene source	description
rs752081				
rs2341629				
rs1803001	X	ZMYM3	HGNC Symbol	zinc finger, MYM-type 3 [Source:HGNC Symbol;Acc:13054]
rs5937076	X	ZMYM3	HGNC Symbol	zinc finger, MYM-type 3 [Source:HGNC Symbol;Acc:13054]
rs73217059	X	ITGB1BP2	HGNC Symbol	integrin beta 1 binding protein (melusin) 2 [Source:HGNC Symbol;Acc:6154]
rs6625777				
rs17311927				
rs73217060	X	TAF1	HGNC Symbol	TAF1 RNA polymerase II, TATA box binding protein (TBP)-associated factor, 250kDa [Source:HGNC Symbol;Acc:11535]
rs28382158	X	TAF1	HGNC Symbol	TAF1 RNA polymerase II, TATA box binding protein (TBP)-associated factor, 250kDa [Source:HGNC Symbol;Acc:11535]
rs4844148	X	TAF1	HGNC Symbol	TAF1 RNA polymerase II, TATA box binding protein (TBP)-associated factor, 250kDa [Source:HGNC Symbol;Acc:11535]
rs28382208	X	TAF1	HGNC Symbol	TAF1 RNA polymerase II, TATA box binding protein (TBP)-associated factor, 250kDa [Source:HGNC Symbol;Acc:11535]
rs5981119	X	TAF1	HGNC Symbol	TAF1 RNA polymerase II, TATA box binding protein (TBP)-associated factor, 250kDa [Source:HGNC Symbol;Acc:11535]
rs113203261	X	TAF1	HGNC Symbol	TAF1 RNA polymerase II, TATA box binding protein (TBP)-associated factor, 250kDa [Source:HGNC Symbol;Acc:11535]
rs17217228	X	TAF1	HGNC Symbol	TAF1 RNA polymerase II, TATA box binding protein (TBP)-associated factor, 250kDa [Source:HGNC Symbol;Acc:11535]
rs5937091	X	TAF1	HGNC Symbol	TAF1 RNA polymerase II, TATA box binding protein (TBP)-associated factor, 250kDa [Source:HGNC Symbol;Acc:11535]
rs192909158	X	OGT	HGNC Symbol	O-linked N-acetylglucosamine (GlcNAc) transferase [Source:HGNC Symbol;Acc:8127]
rs11796215	X	OGT	HGNC Symbol	O-linked N-acetylglucosamine (GlcNAc) transferase [Source:HGNC Symbol;Acc:8127]
rs3736670	X	OGT	HGNC Symbol	O-linked N-acetylglucosamine (GlcNAc) transferase [Source:HGNC Symbol;Acc:8127]
rs145367267	X	OGT	HGNC Symbol	O-linked N-acetylglucosamine (GlcNAc) transferase [Source:HGNC Symbol;Acc:8127]
rs6525489	X	OGT	HGNC Symbol	O-linked N-acetylglucosamine (GlcNAc) transferase [Source:HGNC Symbol;Acc:8127]
rs189919484	X	ACRC	HGNC Symbol	acidic repeat containing [Source:HGNC Symbol;Acc:15805]
rs149580659	X	ACRC	HGNC Symbol	acidic repeat containing [Source:HGNC Symbol;Acc:15805]
rs34963786	X	ACRC	HGNC Symbol	acidic repeat containing [Source:HGNC Symbol;Acc:15805]
rs6625807				
rs6624564				
rs4844299				
rs147969823				
rs17311996				

SNP	Chromosome	gene name	gene source	description
rs5937104				
rs941413				
rs11795584				
rs112869633				
rs6624567				
rs12007790				
rs60688552				
rs7891022				
rs138277585				
rs73217085				
rs4986604				
rs7050263				
rs73634891	X	NHSL2	HGNC Symbol	NHS-like 2 [Source:HGNC Symbol;Acc:33737]
rs4986619	X	NHSL2	HGNC Symbol	NHS-like 2 [Source:HGNC Symbol;Acc:33737]
rs73223121	X	NHSL2	HGNC Symbol	NHS-like 2 [Source:HGNC Symbol;Acc:33737]
rs72630023	X	NHSL2	HGNC Symbol	NHS-like 2 [Source:HGNC Symbol;Acc:33737]
rs55705032	X	NHSL2	HGNC Symbol	NHS-like 2 [Source:HGNC Symbol;Acc:33737]
rs36018136	X	NHSL2	HGNC Symbol	NHS-like 2 [Source:HGNC Symbol;Acc:33737]
rs113867753	X	NHSL2	HGNC Symbol	NHS-like 2 [Source:HGNC Symbol;Acc:33737]
rs138097931	X	NHSL2	HGNC Symbol	NHS-like 2 [Source:HGNC Symbol;Acc:33737]
rs5951199	X	NHSL2	HGNC Symbol	NHS-like 2 [Source:HGNC Symbol;Acc:33737]
rs191505562	X	NHSL2	HGNC Symbol	NHS-like 2 [Source:HGNC Symbol;Acc:33737]
rs12556061	X	NHSL2	HGNC Symbol	NHS-like 2 [Source:HGNC Symbol;Acc:33737]
rs10482163	X	NHSL2	HGNC Symbol	NHS-like 2 [Source:HGNC Symbol;Acc:33737]
rs59036635	X	NHSL2	HGNC Symbol	NHS-like 2 [Source:HGNC Symbol;Acc:33737]
rs62612130	X	NHSL2	HGNC Symbol	NHS-like 2 [Source:HGNC Symbol;Acc:33737]
rs115948179	X	NHSL2	HGNC Symbol	NHS-like 2 [Source:HGNC Symbol;Acc:33737]
rs6624592	X	NHSL2	HGNC Symbol	NHS-like 2 [Source:HGNC Symbol;Acc:33737]
rs6525568	X	NHSL2	HGNC Symbol	NHS-like 2 [Source:HGNC Symbol;Acc:33737]
rs12390630	X	NHSL2	HGNC Symbol	NHS-like 2 [Source:HGNC Symbol;Acc:33737]
rs6525571	X	NHSL2	HGNC Symbol	NHS-like 2 [Source:HGNC Symbol;Acc:33737]
rs73565816	X	NHSL2	HGNC Symbol	NHS-like 2 [Source:HGNC Symbol;Acc:33737]
rs7472613	X	NHSL2	HGNC Symbol	NHS-like 2 [Source:HGNC Symbol;Acc:33737]

SNP	Chromosome	gene name	gene source	description
rs11550742	X	NHSL2;RGAG4	HGNC Symbol	NHS-like 2 [Source:HGNC Symbol;Acc:33737];retrotransposon gag domain containing 4 [Source:HGNC Symbol;Acc:29430]
rs12393722	X	NHSL2;RGAG4	HGNC Symbol	NHS-like 2 [Source:HGNC Symbol;Acc:33737];retrotransposon gag domain containing 4 [Source:HGNC Symbol;Acc:29430]
rs6624595	X	NHSL2;RGAG4	HGNC Symbol	NHS-like 2 [Source:HGNC Symbol;Acc:33737];retrotransposon gag domain containing 4 [Source:HGNC Symbol;Acc:29430]
rs12390592	X	NHSL2;RP11-262D11.1	HGNC Symbol;Clone-based (Vega) gene	NHS-like 2 [Source:HGNC Symbol;Acc:33737];
rs7061150	X	NHSL2	HGNC Symbol	NHS-like 2 [Source:HGNC Symbol;Acc:33737]
rs148426364	X	NHSL2	HGNC Symbol	NHS-like 2 [Source:HGNC Symbol;Acc:33737]
rs7884806	X	RP11-262D11.2	Clone-based (Vega) gene	
rs7880917	X	RP11-262D11.2	Clone-based (Vega) gene	
rs6525585				
rs10284060				
rs6525589	X	PIN4	HGNC Symbol	protein (peptidylprolyl cis/trans isomerase) NIMA-interacting, 4 (parvulin) [Source:HGNC Symbol;Acc:8992]
rs7058353	X	PIN4	HGNC Symbol	protein (peptidylprolyl cis/trans isomerase) NIMA-interacting, 4 (parvulin) [Source:HGNC Symbol;Acc:8992]
rs141356725	X	PIN4	HGNC Symbol	protein (peptidylprolyl cis/trans isomerase) NIMA-interacting, 4 (parvulin) [Source:HGNC Symbol;Acc:8992]
rs7890052	X	PIN4	HGNC Symbol	protein (peptidylprolyl cis/trans isomerase) NIMA-interacting, 4 (parvulin) [Source:HGNC Symbol;Acc:8992]
rs11415	X	PIN4	HGNC Symbol	protein (peptidylprolyl cis/trans isomerase) NIMA-interacting, 4 (parvulin) [Source:HGNC Symbol;Acc:8992]
rs45448501	X	PIN4;ERCC6L	HGNC Symbol	protein (peptidylprolyl cis/trans isomerase) NIMA-interacting, 4 (parvulin) [Source:HGNC Symbol;Acc:8992];excision repair cross-complementing rodent repair deficiency, complementation group 6-like [Source:HGNC Symbol;Acc:20794]
rs12861442	X	PIN4;ERCC6L	HGNC Symbol	protein (peptidylprolyl cis/trans isomerase) NIMA-interacting, 4 (parvulin) [Source:HGNC Symbol;Acc:8992];excision repair cross-complementing rodent repair deficiency, complementation group 6-like [Source:HGNC Symbol;Acc:20794]
rs73225104	X	PIN4;ERCC6L	HGNC Symbol	protein (peptidylprolyl cis/trans isomerase) NIMA-interacting, 4 (parvulin) [Source:HGNC Symbol;Acc:8992];excision repair cross-complementing rodent repair deficiency, complementation group 6-like [Source:HGNC Symbol;Acc:20794]
rs41298504	X	PIN4;ERCC6L	HGNC Symbol	protein (peptidylprolyl cis/trans isomerase) NIMA-interacting, 4 (parvulin) [Source:HGNC Symbol;Acc:8992];excision repair cross-complementing rodent repair deficiency, complementation group 6-like [Source:HGNC Symbol;Acc:20794]

SNP	Chromosome	gene name	gene source	description
rs3012647	X	PIN4	HGNC Symbol	protein (peptidylprolyl cis/trans isomerase) NIMA-interacting, 4 (parvulin) [Source:HGNC Symbol;Acc:8992]
rs11798909	X	PIN4	HGNC Symbol	protein (peptidylprolyl cis/trans isomerase) NIMA-interacting, 4 (parvulin) [Source:HGNC Symbol;Acc:8992]
rs139452358	X	PIN4	HGNC Symbol	protein (peptidylprolyl cis/trans isomerase) NIMA-interacting, 4 (parvulin) [Source:HGNC Symbol;Acc:8992]
rs5958779	X	PIN4	HGNC Symbol	protein (peptidylprolyl cis/trans isomerase) NIMA-interacting, 4 (parvulin) [Source:HGNC Symbol;Acc:8992]
rs3012627	X	PIN4;CITED1	HGNC Symbol	protein (peptidylprolyl cis/trans isomerase) NIMA-interacting, 4 (parvulin) [Source:HGNC Symbol;Acc:8992];Cbp/p300-interacting transactivator, with Glu/Asp-rich carboxy-terminal domain, 1 [Source:HGNC Symbol;Acc:1986]
rs2984344	X	CITED1	HGNC Symbol	Cbp/p300-interacting transactivator, with Glu/Asp-rich carboxy-terminal domain, 1 [Source:HGNC Symbol;Acc:1986]
rs3012625				
rs664221				
rs646297				
rs585535				
rs7883438				
rs73554656	X;HG1438_PATCH	HDAC8	HGNC Symbol	histone deacetylase 8 [Source:HGNC Symbol;Acc:13315]
rs515992	X;HG1438_PATCH	HDAC8	HGNC Symbol	histone deacetylase 8 [Source:HGNC Symbol;Acc:13315]
rs73551091	X;HG1438_PATCH	HDAC8	HGNC Symbol	histone deacetylase 8 [Source:HGNC Symbol;Acc:13315]
rs78046994	X;HG1438_PATCH	HDAC8	HGNC Symbol	histone deacetylase 8 [Source:HGNC Symbol;Acc:13315]
rs73225129	X;HG1438_PATCH	HDAC8	HGNC Symbol	histone deacetylase 8 [Source:HGNC Symbol;Acc:13315]
rs966272	X;HG1438_PATCH	HDAC8	HGNC Symbol	histone deacetylase 8 [Source:HGNC Symbol;Acc:13315]
rs138494136	X;HG1438_PATCH	HDAC8	HGNC Symbol	histone deacetylase 8 [Source:HGNC Symbol;Acc:13315]
rs3012642	X	HDAC8	HGNC Symbol	histone deacetylase 8 [Source:HGNC Symbol;Acc:13315]
rs142016337	X;HG1438_PATCH	HDAC8	HGNC Symbol	histone deacetylase 8 [Source:HGNC Symbol;Acc:13315]
rs12007867	X	HDAC8	HGNC Symbol	histone deacetylase 8 [Source:HGNC Symbol;Acc:13315]
rs73218357	X;HG1438_PATCH	HDAC8	HGNC Symbol	histone deacetylase 8 [Source:HGNC Symbol;Acc:13315]

SNP	Chromosome	gene name	gene source	description
rs56153257	X;HG1438_PATCH	HDAC8	HGNC Symbol	histone deacetylase 8 [Source:HGNC Symbol;Acc:13315]
rs67625365	X;HG1438_PATCH	HDAC8	HGNC Symbol	histone deacetylase 8 [Source:HGNC Symbol;Acc:13315]
rs5912109	X;HG1438_PATCH	PHKA1	HGNC Symbol	phosphorylase kinase, alpha 1 (muscle) [Source:HGNC Symbol;Acc:8925]
rs150631317	X;HG1438_PATCH	PHKA1	HGNC Symbol	phosphorylase kinase, alpha 1 (muscle) [Source:HGNC Symbol;Acc:8925]
rs12688343	X;HG1438_PATCH	PHKA1	HGNC Symbol	phosphorylase kinase, alpha 1 (muscle) [Source:HGNC Symbol;Acc:8925]
rs62613008	X;HG1438_PATCH	PHKA1	HGNC Symbol	phosphorylase kinase, alpha 1 (muscle) [Source:HGNC Symbol;Acc:8925]
rs56047400	X;HG1438_PATCH	U3	RFAM	Small nucleolar RNA U3 [Source:RFAM;Acc:RF00012]
rs12014250				
rs137927439				
rs4623616				
rs4269698				
rs5937794				
rs139180358				
rs5937826				
rs12840698				
rs62613928				
rs5937309				
rs5937382				
rs56164817				
rs67056036				
rs144568122				
rs73222179				
rs2362988				
rs145879449				
rs239906	X	AC004074.4	Clone-based (Vega) gene	
rs239905				
rs150832600				
rs5937611				

SNP	Chromosome	gene name	gene source	description
rs6648197				
rs2428415				
rs239875				
rs6647371				
rs56287682				
rs7881565				
rs2075528	X	CDX4	HGNC Symbol	caudal type homeobox 4 [Source:HGNC Symbol;Acc:1808]
rs151310846	X	RPL7P53;MAP2K4P1	HGNC Symbol	ribosomal protein L7 pseudogene 53 [Source:HGNC Symbol;Acc:36831];mitogen-activated protein kinase kinase 4 pseudogene 1 [Source:HGNC Symbol;Acc:43837]
rs73229442	X	CHIC1	HGNC Symbol	cysteine-rich hydrophobic domain 1 [Source:HGNC Symbol;Acc:1934]
rs12559655	X	CHIC1	HGNC Symbol	cysteine-rich hydrophobic domain 1 [Source:HGNC Symbol;Acc:1934]
rs36079594				
rs1088599				
rs62610693				
rs5981565	X	TSIX	HGNC Symbol	TSIX transcript, XIST antisense RNA [Source:HGNC Symbol;Acc:12377]
rs41303699	X	XIST;TSIX	HGNC Symbol	X inactive specific transcript (non-protein coding) [Source:HGNC Symbol;Acc:12810];TSIX transcript, XIST antisense RNA [Source:HGNC Symbol;Acc:12377]
rs7888812	X	XIST	HGNC Symbol	X inactive specific transcript (non-protein coding) [Source:HGNC Symbol;Acc:12810]
rs6528	X	XIST	HGNC Symbol	X inactive specific transcript (non-protein coding) [Source:HGNC Symbol;Acc:12810]
rs195678				
rs7880327				
rs112063193	X	JPX;RP13-216E22.5	HGNC Symbol;Clone-based (Vega) gene	JPX transcript, XIST activator (non-protein coding) [Source:HGNC Symbol;Acc:37191];
rs142481296	X	JPX;FTX;RP13-216E22.5	HGNC Symbol;Clone-based (Vega) gene	JPX transcript, XIST activator (non-protein coding) [Source:HGNC Symbol;Acc:37191];FTX transcript, XIST regulator (non-protein coding) [Source:HGNC Symbol;Acc:37190];
rs659249	X	JPX;FTX	HGNC Symbol	JPX transcript, XIST activator (non-protein coding) [Source:HGNC Symbol;Acc:37191];FTX transcript, XIST regulator (non-protein coding) [Source:HGNC Symbol;Acc:37190]
rs11795520	X	FTX	HGNC Symbol	FTX transcript, XIST regulator (non-protein coding) [Source:HGNC Symbol;Acc:37190]
rs73214333	X	FTX	HGNC Symbol	FTX transcript, XIST regulator (non-protein coding) [Source:HGNC Symbol;Acc:37190]
rs6655556	X	RAB11FIP1P1;FTX;RP3-368A4.5	HGNC Symbol;Clone-based (Vega) gene	RAB11 family interacting protein 1 (class I) pseudogene 1 [Source:HGNC Symbol;Acc:42366];FTX transcript, XIST regulator (non-protein coding) [Source:HGNC Symbol;Acc:37190];

SNP	Chromosome	gene name	gene source	description
rs144570538	X	FTX;RP3-368A4.5	HGNC Symbol;Clone-based (Vega) gene	FTX transcript, XIST regulator (non-protein coding) [Source:HGNC Symbol;Acc:37190];
rs1341325	X	FTX;RP3-368A4.5	HGNC Symbol;Clone-based (Vega) gene	FTX transcript, XIST regulator (non-protein coding) [Source:HGNC Symbol;Acc:37190];
rs174194	X	FTX;RP3-368A4.5	HGNC Symbol;Clone-based (Vega) gene	FTX transcript, XIST regulator (non-protein coding) [Source:HGNC Symbol;Acc:37190];
rs17217843				
rs636078				
rs667313				
rs17312507				
rs147312656				
rs1634915				
rs473645				
rs6647476	X	SLC16A2	HGNC Symbol	solute carrier family 16, member 2 (thyroid hormone transporter) [Source:HGNC Symbol;Acc:10923]
rs5937813	X	SLC16A2	HGNC Symbol	solute carrier family 16, member 2 (thyroid hormone transporter) [Source:HGNC Symbol;Acc:10923]
rs12559757	X	SLC16A2	HGNC Symbol	solute carrier family 16, member 2 (thyroid hormone transporter) [Source:HGNC Symbol;Acc:10923]
rs72630714	X	SLC16A2	HGNC Symbol	solute carrier family 16, member 2 (thyroid hormone transporter) [Source:HGNC Symbol;Acc:10923]
rs1263181	X	SLC16A2	HGNC Symbol	solute carrier family 16, member 2 (thyroid hormone transporter) [Source:HGNC Symbol;Acc:10923]
rs12688747	X	SLC16A2	HGNC Symbol	solute carrier family 16, member 2 (thyroid hormone transporter) [Source:HGNC Symbol;Acc:10923]
rs112573145	X	SLC16A2	HGNC Symbol	solute carrier family 16, member 2 (thyroid hormone transporter) [Source:HGNC Symbol;Acc:10923]
rs60345806	X	SLC16A2	HGNC Symbol	solute carrier family 16, member 2 (thyroid hormone transporter) [Source:HGNC Symbol;Acc:10923]
rs5937837	X	SLC16A2	HGNC Symbol	solute carrier family 16, member 2 (thyroid hormone transporter) [Source:HGNC Symbol;Acc:10923]
rs148883744	X	SLC16A2	HGNC Symbol	solute carrier family 16, member 2 (thyroid hormone transporter) [Source:HGNC Symbol;Acc:10923]
rs144858450	X	SLC16A2	HGNC Symbol	solute carrier family 16, member 2 (thyroid hormone transporter) [Source:HGNC Symbol;Acc:10923]
rs73216270				
rs73216271				
rs5981649				
rs56336157				
rs5981297				
rs6647514				
rs16991800				
rs138135984				

SNP	Chromosome	gene name	gene source	description
rs41310631	X	KIAA2022	HGNC Symbol	KIAA2022 [Source:HGNC Symbol;Acc:29433]
rs73216276	X	KIAA2022	HGNC Symbol	KIAA2022 [Source:HGNC Symbol;Acc:29433]
rs145013494	X	KIAA2022	HGNC Symbol	KIAA2022 [Source:HGNC Symbol;Acc:29433]
rs62612360	X	KIAA2022	HGNC Symbol	KIAA2022 [Source:HGNC Symbol;Acc:29433]
rs113337777	X	KIAA2022	HGNC Symbol	KIAA2022 [Source:HGNC Symbol;Acc:29433]
rs62612362	X	KIAA2022	HGNC Symbol	KIAA2022 [Source:HGNC Symbol;Acc:29433]
rs73216285	X	KIAA2022	HGNC Symbol	KIAA2022 [Source:HGNC Symbol;Acc:29433]
rs73625833				
rs12687919				
rs2886720				
rs141003973				
rs5937937				
rs6647617	X	ABCB7	HGNC Symbol	ATP-binding cassette, sub-family B (MDR/TAP), member 7 [Source:HGNC Symbol;Acc:48]
rs1340990	X	ABCB7	HGNC Symbol	ATP-binding cassette, sub-family B (MDR/TAP), member 7 [Source:HGNC Symbol;Acc:48]
rs5937319	X	ABCB7	HGNC Symbol	ATP-binding cassette, sub-family B (MDR/TAP), member 7 [Source:HGNC Symbol;Acc:48]
rs5937939	X	ABCB7	HGNC Symbol	ATP-binding cassette, sub-family B (MDR/TAP), member 7 [Source:HGNC Symbol;Acc:48]
rs146861844	X	ABCB7	HGNC Symbol	ATP-binding cassette, sub-family B (MDR/TAP), member 7 [Source:HGNC Symbol;Acc:48]
rs41305387	X	ABCB7	HGNC Symbol	ATP-binding cassette, sub-family B (MDR/TAP), member 7 [Source:HGNC Symbol;Acc:48]
rs61323727	X	ABCB7	HGNC Symbol	ATP-binding cassette, sub-family B (MDR/TAP), member 7 [Source:HGNC Symbol;Acc:48]
rs5981777	X	ABCB7	HGNC Symbol	ATP-binding cassette, sub-family B (MDR/TAP), member 7 [Source:HGNC Symbol;Acc:48]
rs5937966				
rs73218183				
rs5981833	X	UPRT	HGNC Symbol	uracil phosphoribosyltransferase (FUR1) homolog (S. cerevisiae) [Source:HGNC Symbol;Acc:28334]
rs41307373	X	UPRT	HGNC Symbol	uracil phosphoribosyltransferase (FUR1) homolog (S. cerevisiae) [Source:HGNC Symbol;Acc:28334]
rs5937362				
rs5937366				
rs5937367				
rs5937370	X	ZDHHC15	HGNC Symbol	zinc finger, DHHC-type containing 15 [Source:HGNC Symbol;Acc:20342]
rs138848911	X	ZDHHC15	HGNC Symbol	zinc finger, DHHC-type containing 15 [Source:HGNC Symbol;Acc:20342]
rs56166350	X	ZDHHC15	HGNC Symbol	zinc finger, DHHC-type containing 15 [Source:HGNC Symbol;Acc:20342]
rs35921625	X	ZDHHC15	HGNC Symbol	zinc finger, DHHC-type containing 15 [Source:HGNC Symbol;Acc:20342]
rs113787696				
rs58191293				

SNP	Chromosome	gene name	gene source	description
rs73221932				
rs113228883				
rs111314283				
rs5981919				
rs11093567				
rs6647802				
rs5981937				
rs141835479				
rs11796957				
rs7057424				
rs147365180				
rs62622626				
rs5938296				
rs112296965				
rs73223970				
rs242468				
rs1304203				
rs72626714				
rs2213481				
rs6647912				
rs5981993				
rs187899581				
rs17218184				
rs5937496				
rs143133138				
rs73225938				
rs138831049				
rs112592248				
rs5982036				
rs1604733				
rs1503645				
rs113881521				
rs6647232				

SNP	Chromosome	gene name	gene source	description
rs12851854				
rs73231809				
rs111874075				
rs5982200				
rs4892684				
rs5982207				
rs144553307				
rs6607798				
rs73215619				
rs5938637				
rs73215634				
rs143815916				
rs143072286				
rs140583970				
rs66961403				
rs113877841				
rs112636706				
rs62612661				
rs5938746				
rs5938763				
rs5937661				
rs75016512				
rs976641				
rs113228998				
rs4369147				
rs35610395				
rs1375399				
rs1375398				
rs3099677				
rs3099716				
rs6607884				
rs5938937				
rs4892749				

SNP	Chromosome	gene name	gene source	description
rs111241532				
rs5981550				
rs35929441				
rs28637360				
rs28882668				
rs28653671				
rs73224149				
rs73224157				
rs763739	X;HG1426_PATCH	ATRX	HGNC Symbol	alpha thalassemia/mental retardation syndrome X-linked [Source:HGNC Symbol;Acc:886]
rs56394234	X;HG1426_PATCH	ATRX	HGNC Symbol	alpha thalassemia/mental retardation syndrome X-linked [Source:HGNC Symbol;Acc:886]
rs45459797	X;HG1426_PATCH	ATRX	HGNC Symbol	alpha thalassemia/mental retardation syndrome X-linked [Source:HGNC Symbol;Acc:886]
rs45464195	X;HG1426_PATCH	ATRX	HGNC Symbol	alpha thalassemia/mental retardation syndrome X-linked [Source:HGNC Symbol;Acc:886]
rs45460899	X;HG1426_PATCH	ATRX	HGNC Symbol	alpha thalassemia/mental retardation syndrome X-linked [Source:HGNC Symbol;Acc:886]
rs3088074	X;HG1426_PATCH	ATRX	HGNC Symbol	alpha thalassemia/mental retardation syndrome X-linked [Source:HGNC Symbol;Acc:886]
rs12395309	X;HG1426_PATCH	ATRX	HGNC Symbol	alpha thalassemia/mental retardation syndrome X-linked [Source:HGNC Symbol;Acc:886]
rs17303393	X;HG1426_PATCH	ATP7A	HGNC Symbol	ATPase, Cu++ transporting, alpha polypeptide [Source:HGNC Symbol;Acc:869]
rs62614930	X;HG1426_PATCH	ATP7A	HGNC Symbol	ATPase, Cu++ transporting, alpha polypeptide [Source:HGNC Symbol;Acc:869]
rs2227291	X;HG1426_PATCH	ATP7A	HGNC Symbol	ATPase, Cu++ transporting, alpha polypeptide [Source:HGNC Symbol;Acc:869]
rs374162669	X;HG1426_PATCH	ATP7A	HGNC Symbol	ATPase, Cu++ transporting, alpha polypeptide [Source:HGNC Symbol;Acc:869]
rs17218317				
rs36103418	X;HG1426_PATCH	PGK1	HGNC Symbol	phosphoglycerate kinase 1 [Source:HGNC Symbol;Acc:8896]
rs717689	X;HG1426_PATCH	PGK1	HGNC Symbol	phosphoglycerate kinase 1 [Source:HGNC Symbol;Acc:8896]
rs5959980				
rs139511170				
rs150555567	X	CYSLTR1	HGNC Symbol	cysteinyl leukotriene receptor 1 [Source:HGNC Symbol;Acc:17451]

SNP	Chromosome	gene name	gene source	description
rs73226142	X	CYSLTR1	HGNC Symbol	cysteinyl leukotriene receptor 1 [Source:HGNC Symbol;Acc:17451]
rs321074	X	CYSLTR1	HGNC Symbol	cysteinyl leukotriene receptor 1 [Source:HGNC Symbol;Acc:17451]
rs56176732	X	CYSLTR1	HGNC Symbol	cysteinyl leukotriene receptor 1 [Source:HGNC Symbol;Acc:17451]
rs34607276	X	CYSLTR1	HGNC Symbol	cysteinyl leukotriene receptor 1 [Source:HGNC Symbol;Acc:17451]
rs321006	X	CYSLTR1	HGNC Symbol	cysteinyl leukotriene receptor 1 [Source:HGNC Symbol;Acc:17451]
rs321023				
rs62612828				
rs321034				
rs138055888				
rs3943322				
rs73227631				
rs6521954				
rs111951461				
rs12393410				
rs5912622				
rs756384				
rs111385766				
rs5958836				
rs73491916				
rs5959165				
rs139495644				
rs5912631				
rs12836631				
rs62612903				
rs41306247	X	ZCCHC5	HGNC Symbol	zinc finger, CCHC domain containing 5 [Source:HGNC Symbol;Acc:22997]
rs4077512	X	ZCCHC5	HGNC Symbol	zinc finger, CCHC domain containing 5 [Source:HGNC Symbol;Acc:22997]
rs112125049				
rs12556323				
rs148260886				
rs113622338				
rs34658552	X	LPAR4	HGNC Symbol	lysophosphatidic acid receptor 4 [Source:HGNC Symbol;Acc:4478]
rs62614227	X	LPAR4	HGNC Symbol	lysophosphatidic acid receptor 4 [Source:HGNC Symbol;Acc:4478]
rs1140040	X	LPAR4	HGNC Symbol	lysophosphatidic acid receptor 4 [Source:HGNC Symbol;Acc:4478]

SNP	Chromosome	gene name	gene source	description
rs7890636				
rs57864574				
rs67468150				
rs56228754				
rs67686668				
rs148515202				
rs2412097				
rs12844521				
rs140086795				
rs527098	X	P2RY10	HGNC Symbol	purinergic receptor P2Y, G-protein coupled, 10 [Source:HGNC Symbol;Acc:19906]
rs6618868	X	P2RY10	HGNC Symbol	purinergic receptor P2Y, G-protein coupled, 10 [Source:HGNC Symbol;Acc:19906]
rs73231530				
rs57033802				
rs73231536				
rs145934807				
rs35796855				
rs150957066				
rs141751756				
rs5912731				
rs6619490				
rs5959255				
rs3827440	X	GPR174	HGNC Symbol	G protein-coupled receptor 174 [Source:HGNC Symbol;Acc:30245]
rs5912794				
rs5912234				
rs193142535				
rs73233212				
rs113354281				
rs5912844				
rs6619800				
rs5912854				
rs5959289				
rs114598022				
rs6615743				

SNP	Chromosome	gene name	gene source	description
rs5959303				
rs1736680				
rs1751094	X	ITM2A	HGNC Symbol	integral membrane protein 2A [Source:HGNC Symbol;Acc:6173]
rs73233295				
rs7886910				
rs142817953				
rs1474563				
rs17251845				
rs28421625				
rs149011784				
rs62606354				
rs4826102				
rs148878183				
rs7879177				
rs5912299				
rs5912970				
rs146033607				
rs59936200				
rs5959388				
rs2411754				
rs73235393				
rs5959397				
rs140969463				
rs1603379				
rs4826118				
rs73237443				
rs12839556				
rs188413882				
rs6620807				
rs2898803				
rs73237465				
rs1898819				
rs7064039				

SNP	Chromosome	gene name	gene source	description
rs3127134				
rs10081845				
rs5913115				
rs138305750				
rs5913116				
rs113109168				
rs5913147				
rs11266520				
rs59192467				
rs62598520				
rs6616332				
rs6523640				
rs73241427				
rs56909837	X	TBX22	HGNC Symbol	T-box 22 [Source:HGNC Symbol;Acc:11600]
rs34244923	X	TBX22	HGNC Symbol	T-box 22 [Source:HGNC Symbol;Acc:11600]
rs195276				
rs6621634				
rs73241443				
rs1008201				
rs112732910				
rs61631808				
rs589183				
rs12013328				
rs113375614				
rs149196343				
rs560665				
rs147576166				
rs393298				
rs73223413				
rs59321203				
rs2082350				
rs6652913				
rs4826173				

SNP	Chromosome	gene name	gene source	description
rs73223443				
rs4498691	X	CHMP1B2P	HGNC Symbol	charged multivesicular body protein 1B2, pseudogene [Source:HGNC Symbol;Acc:49380]
rs5913255	X	FAM46D	HGNC Symbol	family with sequence similarity 46, member D [Source:HGNC Symbol;Acc:28399]
rs67793634	X	FAM46D	HGNC Symbol	family with sequence similarity 46, member D [Source:HGNC Symbol;Acc:28399]
rs34189733	X	FAM46D	HGNC Symbol	family with sequence similarity 46, member D [Source:HGNC Symbol;Acc:28399]
rs73223496				
rs58836750				
rs73223502				
rs5959033				
rs7066655				
rs115562366				
rs12558413				
rs2175647				
rs3810676	X	BRWD3	HGNC Symbol	bromodomain and WD repeat domain containing 3 [Source:HGNC Symbol;Acc:17342]
rs41300175	X	BRWD3	HGNC Symbol	bromodomain and WD repeat domain containing 3 [Source:HGNC Symbol;Acc:17342]
rs112186701	X	BRWD3	HGNC Symbol	bromodomain and WD repeat domain containing 3 [Source:HGNC Symbol;Acc:17342]
rs143041103	X	BRWD3	HGNC Symbol	bromodomain and WD repeat domain containing 3 [Source:HGNC Symbol;Acc:17342]
rs3106407	X	BRWD3	HGNC Symbol	bromodomain and WD repeat domain containing 3 [Source:HGNC Symbol;Acc:17342]
rs12841491	X	BRWD3	HGNC Symbol	bromodomain and WD repeat domain containing 3 [Source:HGNC Symbol;Acc:17342]
rs145445891				
rs3123265				
rs3123266				
rs4826193				
rs6622379				
rs4317712				
rs7878159				
rs73227354				
rs73227355				
rs12013713				
rs7064573				
rs145679683				
rs11798879				
rs1166645				

SNP	Chromosome	gene name	gene source	description
rs1166683				
rs17328555				
rs112787998	X	HMGN5	HGNC Symbol	high mobility group nucleosome binding domain 5 [Source:HGNC Symbol;Acc:8013]
rs189501409	X	HMGN5	HGNC Symbol	high mobility group nucleosome binding domain 5 [Source:HGNC Symbol;Acc:8013]
rs2027279	X	SH3BGRL	HGNC Symbol	SH3 domain binding glutamic acid-rich protein like [Source:HGNC Symbol;Acc:10823]
rs17328569	X	SH3BGRL	HGNC Symbol	SH3 domain binding glutamic acid-rich protein like [Source:HGNC Symbol;Acc:10823]
rs12842228	X	SH3BGRL	HGNC Symbol	SH3 domain binding glutamic acid-rich protein like [Source:HGNC Symbol;Acc:10823]
rs1016393				
rs138123519				
rs113170149				
rs55861599				
rs56169132				
rs5959880				
rs185892836				
rs2444580	X	RP13-52K8.1	Clone-based (Vega) gene	
rs9780603				
rs12839057				
rs1563086				
rs148665457				
rs7057095				
rs56360532				
rs4526500				
rs5913557				
rs5959119	X	XXyac-YR12DB5.1	Clone-based (Vega) gene	
rs5912543				
rs6622535				
rs73233109				
rs6524130				
rs6622563				
rs143225829				
rs150179359				
rs6652992				

SNP	Chromosome	gene name	gene source	description
rs2516128				
rs4367716				
rs142819227				
rs5968184				
rs12846003				
rs506604				
rs67258221				
rs182382747				
rs145713609				
rs147166358				
rs12836422				
rs141990906				
rs73241350				
rs150250904				
rs12556404				
rs4828452				
rs35243821				
rs56139406				
rs2223215				
rs113149167				
rs139791236				
rs12836514				
rs6622635				
rs73224720				
rs73224721				
rs6616817				
rs6622648				
rs148881997				
rs1923010				
rs1321179				
rs35199902				
rs181757683				
rs2223347				

SNP	Chromosome	gene name	gene source	description
rs139006038				
rs145384325				
rs2223351				
rs5969722				
rs5922643				
rs5922652				
rs73510029				
rs5921922				
rs5967389				
rs139031358				
rs2317504				
rs7884462				
rs34104871				
rs56082706				
rs73226802				
rs113126610				
rs593848				
rs143879479				
rs1886886				
rs62617489				
rs7054313				
rs4828220				
rs6622726				
rs2103551				
rs192705018				
rs12688013				
rs138369113				
rs1410530				
rs12687893				
rs73230915				
rs150558137				
rs213464				
rs213443				

SNP	Chromosome	gene name	gene source	description
rs6622761				
rs56035532	X	RP3-326L13.2	Clone-based (Vega) gene	
rs12841496				
rs59117209	X	RP1-223D17.1	Clone-based (Vega) gene	
rs62615013				
rs5922826				
rs148046761				
rs5922838				
rs2506840				
rs5968205				
rs5967455				
rs825552				
rs62616381				
rs73230984				
rs4375148				
rs143954881				
rs5922005				
rs34762696				
rs17304335				
rs139572143				
rs113813840				
rs12858390				
rs73232922				
rs3747462				
rs6616890	X	RPS6KA6	HGNC Symbol	ribosomal protein S6 kinase, 90kDa, polypeptide 6 [Source:HGNC Symbol;Acc:10435]
rs5922911	X	RPS6KA6	HGNC Symbol	ribosomal protein S6 kinase, 90kDa, polypeptide 6 [Source:HGNC Symbol;Acc:10435]
rs9887469	X	RPS6KA6	HGNC Symbol	ribosomal protein S6 kinase, 90kDa, polypeptide 6 [Source:HGNC Symbol;Acc:10435]
rs140894900	X	RPS6KA6	HGNC Symbol	ribosomal protein S6 kinase, 90kDa, polypeptide 6 [Source:HGNC Symbol;Acc:10435]
rs5968267	X	RPS6KA6	HGNC Symbol	ribosomal protein S6 kinase, 90kDa, polypeptide 6 [Source:HGNC Symbol;Acc:10435]
rs62614184	X	RPS6KA6	HGNC Symbol	ribosomal protein S6 kinase, 90kDa, polypeptide 6 [Source:HGNC Symbol;Acc:10435]
rs73232992	X	RPS6KA6	HGNC Symbol	ribosomal protein S6 kinase, 90kDa, polypeptide 6 [Source:HGNC Symbol;Acc:10435]
rs186821727				

SNP	Chromosome	gene name	gene source	description
rs62614219				
rs5968284				
rs5922934				
rs144084720				
rs12688565				
rs5922038				
rs5003031				
rs144829573	X	HDX	HGNC Symbol	highly divergent homeobox [Source:HGNC Symbol;Acc:26411]
rs73237161	X	HDX	HGNC Symbol	highly divergent homeobox [Source:HGNC Symbol;Acc:26411]
rs73237162	X	HDX	HGNC Symbol	highly divergent homeobox [Source:HGNC Symbol;Acc:26411]
rs55719627	X	HDX	HGNC Symbol	highly divergent homeobox [Source:HGNC Symbol;Acc:26411]
rs151066610	X	HDX	HGNC Symbol	highly divergent homeobox [Source:HGNC Symbol;Acc:26411]
rs58322933	X	HDX	HGNC Symbol	highly divergent homeobox [Source:HGNC Symbol;Acc:26411]
rs5922051	X	HDX	HGNC Symbol	highly divergent homeobox [Source:HGNC Symbol;Acc:26411]
rs35161124	X	HDX	HGNC Symbol	highly divergent homeobox [Source:HGNC Symbol;Acc:26411]
rs146859394	X	HDX	HGNC Symbol	highly divergent homeobox [Source:HGNC Symbol;Acc:26411]
rs12856189				
rs73241192				
rs2341838				
rs1406988				
rs436276				
rs55634773				
rs62615887				
rs707675				
rs1831116				
rs139875497				
rs73226570				
rs1157463				
rs74738892				
rs5923117				
rs6616992				
rs5923132				
rs150673674				

SNP	Chromosome	gene name	gene source	description
rs67274104				
rs143957861				
rs12852750				
rs5923157				
rs150926528				
rs35639239				
rs12687497				
rs12687190				
rs62613857				
rs6623194				
rs66786391				
rs1517192				
rs72632502				
rs5922108				
rs5922114				
rs17324846				
rs5923199				
rs1573689	X	RP1-215K18.4	Clone-based (Vega) gene	
rs7062047				
rs3747422	X	APOOL	HGNC Symbol	apolipoprotein O-like [Source:HGNC Symbol;Acc:24009]
rs5968436	X	APOOL	HGNC Symbol	apolipoprotein O-like [Source:HGNC Symbol;Acc:24009]
rs4828319	X	APOOL	HGNC Symbol	apolipoprotein O-like [Source:HGNC Symbol;Acc:24009]
rs142184202	X	APOOL	HGNC Symbol	apolipoprotein O-like [Source:HGNC Symbol;Acc:24009]
rs10126146	X	SATL1	HGNC Symbol	spermidine/spermine N1-acetyl transferase-like 1 [Source:HGNC Symbol;Acc:27992]
rs12014708				
rs5968458				
rs16980121				
rs138160068				
rs1013639				
rs17252084				
rs73234630				
rs55972110				

SNP	Chromosome	gene name	gene source	description
rs1989862				
rs2188732				
rs363774	X	POF1B	HGNC Symbol	premature ovarian failure, 1B [Source:HGNC Symbol;Acc:13711]
rs7886971	X	POF1B	HGNC Symbol	premature ovarian failure, 1B [Source:HGNC Symbol;Acc:13711]
rs147552598	X	POF1B	HGNC Symbol	premature ovarian failure, 1B [Source:HGNC Symbol;Acc:13711]
rs363765	X	POF1B	HGNC Symbol	premature ovarian failure, 1B [Source:HGNC Symbol;Acc:13711]
rs5968480	X	POF1B	HGNC Symbol	premature ovarian failure, 1B [Source:HGNC Symbol;Acc:13711]
rs6524433				
rs7051722				
rs145322232				
rs6623287				
rs6617059				
rs5923297				
rs6524462				
rs7890011				
rs111342773				
rs147832437				
rs5968493				
rs6623339				
rs73236000				
rs6623340				
rs141719943				
rs75663308				
rs6524498				
rs150755043				
rs5968568				
rs73239767				
rs17252313				
rs6623424				
rs12556281				
rs5923356				
rs2064151				
rs5922188				

SNP	Chromosome	gene name	gene source	description
rs5968673				
rs5968690				
rs5968692				
rs7066183				
rs73224576				
rs12845781				
rs142542856				
rs147328763				
rs5968697				
rs5967637				
rs5967638				
rs1013031				
rs3790357	X	CHM	HGNC Symbol	choroideremia (Rab escort protein 1) [Source:HGNC Symbol;Acc:1940]
rs73224586	X	CHM	HGNC Symbol	choroideremia (Rab escort protein 1) [Source:HGNC Symbol;Acc:1940]
rs113031507	X	CHM	HGNC Symbol	choroideremia (Rab escort protein 1) [Source:HGNC Symbol;Acc:1940]
rs147207046	X	CHM	HGNC Symbol	choroideremia (Rab escort protein 1) [Source:HGNC Symbol;Acc:1940]
rs17252383	X	CHM	HGNC Symbol	choroideremia (Rab escort protein 1) [Source:HGNC Symbol;Acc:1940]
rs56169997	X	CHM	HGNC Symbol	choroideremia (Rab escort protein 1) [Source:HGNC Symbol;Acc:1940]
rs12006886	X	CHM	HGNC Symbol	choroideremia (Rab escort protein 1) [Source:HGNC Symbol;Acc:1940]
rs140237438	X	CHM	HGNC Symbol	choroideremia (Rab escort protein 1) [Source:HGNC Symbol;Acc:1940]
rs56205486	X	CHM	HGNC Symbol	choroideremia (Rab escort protein 1) [Source:HGNC Symbol;Acc:1940]
rs7883601	X	CHM	HGNC Symbol	choroideremia (Rab escort protein 1) [Source:HGNC Symbol;Acc:1940]
rs5922214				
rs5922215				
rs67423390				
rs34547958				
rs2746119				
rs5923487				
rs12688954	X	DACH2	HGNC Symbol	dachshund homolog 2 (Drosophila) [Source:HGNC Symbol;Acc:16814]
rs5922230	X	DACH2	HGNC Symbol	dachshund homolog 2 (Drosophila) [Source:HGNC Symbol;Acc:16814]
rs12391988	X	DACH2	HGNC Symbol	dachshund homolog 2 (Drosophila) [Source:HGNC Symbol;Acc:16814]
rs142681233	X	DACH2	HGNC Symbol	dachshund homolog 2 (Drosophila) [Source:HGNC Symbol;Acc:16814]
rs150875723	X	DACH2	HGNC Symbol	dachshund homolog 2 (Drosophila) [Source:HGNC Symbol;Acc:16814]

SNP	Chromosome	gene name	gene source	description
rs11320377	X	DACH2	HGNC Symbol	dachshund homolog 2 (Drosophila) [Source:HGNC Symbol;Acc:16814]
rs1397341	X	DACH2	HGNC Symbol	dachshund homolog 2 (Drosophila) [Source:HGNC Symbol;Acc:16814]
rs195035	X	DACH2	HGNC Symbol	dachshund homolog 2 (Drosophila) [Source:HGNC Symbol;Acc:16814]
rs148970661	X	DACH2	HGNC Symbol	dachshund homolog 2 (Drosophila) [Source:HGNC Symbol;Acc:16814]
rs60442265	X	DACH2	HGNC Symbol	dachshund homolog 2 (Drosophila) [Source:HGNC Symbol;Acc:16814]
rs17325410	X	DACH2	HGNC Symbol	dachshund homolog 2 (Drosophila) [Source:HGNC Symbol;Acc:16814]
rs6617219	X	DACH2	HGNC Symbol	dachshund homolog 2 (Drosophila) [Source:HGNC Symbol;Acc:16814]
rs7052641	X	DACH2	HGNC Symbol	dachshund homolog 2 (Drosophila) [Source:HGNC Symbol;Acc:16814]
rs5968893	X	DACH2	HGNC Symbol	dachshund homolog 2 (Drosophila) [Source:HGNC Symbol;Acc:16814]
rs111658775	X	DACH2	HGNC Symbol	dachshund homolog 2 (Drosophila) [Source:HGNC Symbol;Acc:16814]
rs5968915	X	DACH2	HGNC Symbol	dachshund homolog 2 (Drosophila) [Source:HGNC Symbol;Acc:16814]
rs2369004	X	DACH2	HGNC Symbol	dachshund homolog 2 (Drosophila) [Source:HGNC Symbol;Acc:16814]
rs5968920	X	DACH2	HGNC Symbol	dachshund homolog 2 (Drosophila) [Source:HGNC Symbol;Acc:16814]
rs7055735	X	DACH2	HGNC Symbol	dachshund homolog 2 (Drosophila) [Source:HGNC Symbol;Acc:16814]
rs5967730	X	DACH2	HGNC Symbol	dachshund homolog 2 (Drosophila) [Source:HGNC Symbol;Acc:16814]
rs5967731	X	DACH2	HGNC Symbol	dachshund homolog 2 (Drosophila) [Source:HGNC Symbol;Acc:16814]
rs67364357	X	DACH2	HGNC Symbol	dachshund homolog 2 (Drosophila) [Source:HGNC Symbol;Acc:16814]
rs2369065	X	DACH2	HGNC Symbol	dachshund homolog 2 (Drosophila) [Source:HGNC Symbol;Acc:16814]
rs139995104	X	DACH2	HGNC Symbol	dachshund homolog 2 (Drosophila) [Source:HGNC Symbol;Acc:16814]
rs62593268	X	DACH2	HGNC Symbol	dachshund homolog 2 (Drosophila) [Source:HGNC Symbol;Acc:16814]
rs1883333	X	DACH2	HGNC Symbol	dachshund homolog 2 (Drosophila) [Source:HGNC Symbol;Acc:16814]
rs67087003	X	DACH2	HGNC Symbol	dachshund homolog 2 (Drosophila) [Source:HGNC Symbol;Acc:16814]
rs1014975	X	DACH2	HGNC Symbol	dachshund homolog 2 (Drosophila) [Source:HGNC Symbol;Acc:16814]
rs73506306	X	DACH2	HGNC Symbol	dachshund homolog 2 (Drosophila) [Source:HGNC Symbol;Acc:16814]
rs4828151	X	DACH2	HGNC Symbol	dachshund homolog 2 (Drosophila) [Source:HGNC Symbol;Acc:16814]
rs6623735	X	DACH2	HGNC Symbol	dachshund homolog 2 (Drosophila) [Source:HGNC Symbol;Acc:16814]
rs17278808	X	DACH2	HGNC Symbol	dachshund homolog 2 (Drosophila) [Source:HGNC Symbol;Acc:16814]
rs67485015	X	DACH2	HGNC Symbol	dachshund homolog 2 (Drosophila) [Source:HGNC Symbol;Acc:16814]
rs139146397	X	DACH2	HGNC Symbol	dachshund homolog 2 (Drosophila) [Source:HGNC Symbol;Acc:16814]
rs5923592	X	DACH2	HGNC Symbol	dachshund homolog 2 (Drosophila) [Source:HGNC Symbol;Acc:16814]
rs80343814	X	DACH2	HGNC Symbol	dachshund homolog 2 (Drosophila) [Source:HGNC Symbol;Acc:16814]
rs1291769	X	DACH2	HGNC Symbol	dachshund homolog 2 (Drosophila) [Source:HGNC Symbol;Acc:16814]
rs12835041	X	DACH2	HGNC Symbol	dachshund homolog 2 (Drosophila) [Source:HGNC Symbol;Acc:16814]

SNP	Chromosome	gene name	gene source	description
rs144189313	X	DACH2	HGNC Symbol	dachshund homolog 2 (Drosophila) [Source:HGNC Symbol;Acc:16814]
rs5923636	X	DACH2	HGNC Symbol	dachshund homolog 2 (Drosophila) [Source:HGNC Symbol;Acc:16814]
rs151141427	X	DACH2;RP11-345E19.2	HGNC Symbol;Clone-based (Vega) gene	dachshund homolog 2 (Drosophila) [Source:HGNC Symbol;Acc:16814];
rs12852660	X	DACH2	HGNC Symbol	dachshund homolog 2 (Drosophila) [Source:HGNC Symbol;Acc:16814]
rs148234349	X	DACH2	HGNC Symbol	dachshund homolog 2 (Drosophila) [Source:HGNC Symbol;Acc:16814]
rs41300149	X	DACH2	HGNC Symbol	dachshund homolog 2 (Drosophila) [Source:HGNC Symbol;Acc:16814]
rs6617268				
rs149544817				
rs1113139				
rs1592297				
rs188843537				
rs2710057				
rs17252844				
rs2858684				
rs2213468				
rs5923710				
rs73230769				
rs6524718				
rs62598026				
rs2858709				
rs2746357				
rs2746376				
rs58812623				
rs2172554				
rs5922371				
rs1586957				
rs6617312				
rs111343425				
rs5922379				
rs6617332				
rs6623949				
rs73511059				

SNP	Chromosome	gene name	gene source	description
rs12009000				
rs17252964				
rs62596237				
rs112926275				
rs5922413				
rs5923933				
rs73235604				
rs12388156				
rs12847597				
rs534326				
rs143911714				
rs503848				
rs5967814				
rs518512				
rs34128578				
rs73239826				
rs34480774				
rs147253858				
rs4828171				
rs73239865				
rs6617363				
rs7064578				
rs6624017				
rs73242134				
rs73631521				
rs6624027				
rs72633188				
rs16980838				
rs145946067				
rs4582701				
rs72633191				
rs2213724				
rs144971764				

SNP	Chromosome	gene name	gene source	description
rs2887578				
rs2157410				
rs2369886				
rs4828178				
rs5004685				
rs7064044				
rs6521933				
rs36122044				
rs9792821	X	KLHL4	HGNC Symbol	kelch-like family member 4 [Source:HGNC Symbol;Acc:6355]
rs5969235	X	KLHL4	HGNC Symbol	kelch-like family member 4 [Source:HGNC Symbol;Acc:6355]
rs112304795	X	KLHL4	HGNC Symbol	kelch-like family member 4 [Source:HGNC Symbol;Acc:6355]
rs4631605	X	KLHL4	HGNC Symbol	kelch-like family member 4 [Source:HGNC Symbol;Acc:6355]
rs12391207	X	KLHL4	HGNC Symbol	kelch-like family member 4 [Source:HGNC Symbol;Acc:6355]
rs6614687	X	KLHL4	HGNC Symbol	kelch-like family member 4 [Source:HGNC Symbol;Acc:6355]
rs5967855	X	KLHL4	HGNC Symbol	kelch-like family member 4 [Source:HGNC Symbol;Acc:6355]
rs62608267	X	KLHL4	HGNC Symbol	kelch-like family member 4 [Source:HGNC Symbol;Acc:6355]
rs143149456	X	KLHL4	HGNC Symbol	kelch-like family member 4 [Source:HGNC Symbol;Acc:6355]
rs62608283	X	KLHL4	HGNC Symbol	kelch-like family member 4 [Source:HGNC Symbol;Acc:6355]
rs2273050	X	KLHL4	HGNC Symbol	kelch-like family member 4 [Source:HGNC Symbol;Acc:6355]
rs222070	X	KLHL4	HGNC Symbol	kelch-like family member 4 [Source:HGNC Symbol;Acc:6355]
rs41304056	X	KLHL4	HGNC Symbol	kelch-like family member 4 [Source:HGNC Symbol;Acc:6355]
rs222085	X	KLHL4	HGNC Symbol	kelch-like family member 4 [Source:HGNC Symbol;Acc:6355]
rs72634520	X	KLHL4	HGNC Symbol	kelch-like family member 4 [Source:HGNC Symbol;Acc:6355]
rs11797132	X	KLHL4	HGNC Symbol	kelch-like family member 4 [Source:HGNC Symbol;Acc:6355]
rs222113	X	KLHL4	HGNC Symbol	kelch-like family member 4 [Source:HGNC Symbol;Acc:6355]
rs72634522	X	KLHL4	HGNC Symbol	kelch-like family member 4 [Source:HGNC Symbol;Acc:6355]
rs11092977				
rs55652025				
rs62608198				
rs62608200				
rs1321406				
rs5969287				
rs7065261				

SNP	Chromosome	gene name	gene source	description
rs73241623				
rs5924101				
rs5924107				
rs11796164				
rs73241638				
rs2370013				
rs7891751				
rs2476108				
rs5969333				
rs17253453				
rs2507106				
rs73232261				
rs7063284				
rs56332612				
rs111443168				
rs5924181				
rs5969411				
rs12388812				
rs139097899				
rs17253529				
rs2616723				
rs148528205				
rs142987502				
rs17319482				
rs7888330				
rs73248791				
rs5924210				
rs112168772				
rs58306354				
rs2370427				
rs5969508				
rs11093017				
rs5924294				

SNP	Chromosome	gene name	gene source	description
rs5924297				
rs73518947				
rs73250424				
rs5924435				
rs5969627				
rs146056334				
rs5924440				
rs5924449				
rs1815943				
rs4304477				
rs113466951				
rs11093075				
rs6617604				
rs137863336				
rs149841386				
rs7392254				
rs6522050				
rs73234128				
rs111730264				
rs73234137				
rs6617634				
rs73234138				
rs73499150				
rs148425390				
rs7063211				
rs6617644				
rs6617649				
rs12397840				
rs12393476				
rs6617674				
rs56185299				
rs6522113				
rs12689167				

SNP	Chromosome	gene name	gene source	description
rs73504440				
rs6617709				
rs140030517				
rs56321986				
rs62607943				
rs7052636				
rs6614816				
rs5942377				
rs2206684				
rs67721811				
rs2400252				
rs5942473				
rs35490351				
rs6617733				
rs10521385				
rs5941440				
rs62593864				
rs147186933				
rs55950283				
rs146364790				
rs12557390				
rs59757321				
rs17319825				
rs7889435				
rs73249326				
rs62593867				
rs5940915	X	CPXCR1	HGNC Symbol	CPX chromosome region, candidate 1 [Source:HGNC Symbol;Acc:2332]
rs5984611	X	CPXCR1	HGNC Symbol	CPX chromosome region, candidate 1 [Source:HGNC Symbol;Acc:2332]
rs5984613	X	CPXCR1	HGNC Symbol	CPX chromosome region, candidate 1 [Source:HGNC Symbol;Acc:2332]
rs1883401				
rs2370632				
rs10481946				
rs145441590				

SNP	Chromosome	gene name	gene source	description
rs4893265				
rs139929471				
rs11796364				
rs73251241				
rs6617767				
rs3128273				
rs847475				
rs62594965				
rs17319893				
rs847492				
rs12014129				
rs1586912				
rs12558311				
rs1493010				
rs641388				
rs5942344				
rs572922				
rs7890086				
rs1493007				
rs35490131				
rs4893359				
rs73238213				
rs1540303				
rs72634595				
rs5942368				
rs5942376				
rs73238247				
rs73240118				
rs5985117				
rs111528594				
rs73241980				
rs2209054				
rs5942441				

SNP	Chromosome	gene name	gene source	description
rs35037740				
rs73636407				
rs73244272				
rs1209777				
rs2558769				
rs141440393				
rs5942497				
rs6617912				
rs150089861				
rs6614939				
rs142307315				
rs145795784				
rs172778				
rs149733815				
rs223804				
rs149838623				
rs6618035				
rs66665587				
rs402199				
rs115679507				
rs223677				
rs387294				
rs434868				
rs223753				
rs5941247				
rs223761				
rs140129122				
rs2984667				
rs113715166				
rs115250393				
rs3014770				
rs62594581				
rs3995213				

SNP	Chromosome	gene name	gene source	description
rs73251336				
rs6618160				
rs145703686				
rs144891797				
rs116682661				
rs2257384				
rs2771548				
rs2771588				
rs149379834				
rs62591985				
rs6618185				
rs147544673				
rs151000882				
rs62592016				
rs72617885				
rs2496803				
rs112899483				
rs4549936				
rs5940774				
rs5941436				
rs73234426				
rs73234428				
rs5983987				
rs73234430				
rs5940779				
rs5940783				
rs73234443				
rs2038454				
rs17245585				
rs5941456				
rs55739139				
rs2220360				
rs5940790				

SNP	Chromosome	gene name	gene source	description
rs62592897				
rs2752723				
rs150460010				
rs150957703				
rs2752517				
rs2752299				
rs143466181				
rs4061219				
rs6615117				
rs78382382				
rs141119441				
rs2574447				
rs5941547				
rs3968404				
rs6615141				
rs391199				
rs62594517				
rs28369439				
rs62594540				
rs138061630				
rs11796770				
rs4061559				
rs113330848				
rs73250402				
rs1236613				
rs1236603				
rs149981677				
rs6618432				
rs149580255				
rs139787891				
rs73252413				
rs180701990				
rs2564351				

SNP	Chromosome	gene name	gene source	description
rs113818596				
rs2755745				
rs138119510				
rs143345852				
rs2564233				
rs142417474				
rs7060219				
rs12556544				
rs2755940				
rs2755459				
rs34860881				
rs2755485				
rs2755505				
rs4893139				
rs5941627				
rs111642401				
rs2755589				
rs146610483				
rs5941640				
rs4062116				
rs189187884				
rs144998798				
rs73252451				
rs7066897				
rs62594547				
rs73525181				
rs2756761				
rs145883568				
rs116688350				
rs4893140				
rs138526144				
rs2566049				
rs34273331				

SNP	Chromosome	gene name	gene source	description
rs2481333				
rs143210868				
rs150897648				
rs2481225				
rs6618530				
rs148309153				
rs2774607				
rs143931662				
rs73252473				
rs1898147				
rs185534988				
rs75801529				
rs5941722				
rs5941725				
rs142101606				
rs5941744				
rs62594204				
rs115778147				
rs60573937				
rs35059915				
rs73252488				
rs149913459				
rs149038678				
rs3014505				
rs2917311				
rs59243949				
rs113388918				
rs12853340				
rs72605109				
rs58718607				
rs5941795				
rs5940928				
rs142289898				

SNP	Chromosome	gene name	gene source	description
rs6618575				
rs12840601				
rs142277070				
rs5941856				
rs12387371				
rs142101482				
rs3865905				
rs140058178				
rs7062156				
rs2750246				
rs6652597				
rs77431323				
rs17320245				
rs150979612				
rs5984703				
rs624287				
rs633266				
rs55674632				
rs474622	X	RP13-212L9.1	Clone-based (Vega) gene	
rs513756				
rs525869				
rs73242509				
rs5984097				
rs34684654				
rs11093286				
rs4263932				
rs12556741				
rs5941910	X	PABPC5-AS1	HGNC Symbol	PABPC5 antisense RNA 1 [Source:HGNC Symbol;Acc:31845]
rs12836163	X	PABPC5-AS1	HGNC Symbol	PABPC5 antisense RNA 1 [Source:HGNC Symbol;Acc:31845]
rs5941927				
rs3096995				
rs5984760				

SNP	Chromosome	gene name	gene source	description
rs73242549				
rs12558661				
rs7062118				
rs1986115				
rs3126694				
rs5940990				
rs5940995				
rs138579599				
rs5984108				
rs3100550				
rs73630142				
rs72553276				
rs4521931				
rs5984785				
rs12557563				
rs73242583				
rs5984802				
rs74910046				
rs12116013				
rs4081567				
rs113211372				
rs75153625				
rs141744529				
rs2563419	X	PCDH11X	HGNC Symbol	protocadherin 11 X-linked [Source:HGNC Symbol;Acc:8656]
rs140696510				
rs146750733	X	PCDH11X	HGNC Symbol	protocadherin 11 X-linked [Source:HGNC Symbol;Acc:8656]
rs5942091	X	PCDH11X	HGNC Symbol	protocadherin 11 X-linked [Source:HGNC Symbol;Acc:8656]
rs62607801	X	PCDH11X	HGNC Symbol	protocadherin 11 X-linked [Source:HGNC Symbol;Acc:8656]
rs2525362	X	PCDH11X	HGNC Symbol	protocadherin 11 X-linked [Source:HGNC Symbol;Acc:8656]
rs4893294	X	PCDH11X	HGNC Symbol	protocadherin 11 X-linked [Source:HGNC Symbol;Acc:8656]
rs35071466	X	PCDH11X	HGNC Symbol	protocadherin 11 X-linked [Source:HGNC Symbol;Acc:8656]
rs7881217	X	PCDH11X	HGNC Symbol	protocadherin 11 X-linked [Source:HGNC Symbol;Acc:8656]
rs115408001				

SNP	Chromosome	gene name	gene source	description
rs1294451	X	PCDH11X	HGNC Symbol	protocadherin 11 X-linked [Source:HGNC Symbol;Acc:8656]
rs139814170				
rs182347084				
rs2759963	X	PCDH11X	HGNC Symbol	protocadherin 11 X-linked [Source:HGNC Symbol;Acc:8656]
rs6618858	X	PCDH11X	HGNC Symbol	protocadherin 11 X-linked [Source:HGNC Symbol;Acc:8656]
rs2573905	X	PCDH11X	HGNC Symbol	protocadherin 11 X-linked [Source:HGNC Symbol;Acc:8656]
rs74766983				
rs6618889	X	PCDH11X	HGNC Symbol	protocadherin 11 X-linked [Source:HGNC Symbol;Acc:8656]
rs7881327	X	PCDH11X	HGNC Symbol	protocadherin 11 X-linked [Source:HGNC Symbol;Acc:8656]
rs5942150	X	PCDH11X	HGNC Symbol	protocadherin 11 X-linked [Source:HGNC Symbol;Acc:8656]
rs34636359	X	PCDH11X	HGNC Symbol	protocadherin 11 X-linked [Source:HGNC Symbol;Acc:8656]
rs6618925	X	PCDH11X	HGNC Symbol	protocadherin 11 X-linked [Source:HGNC Symbol;Acc:8656]
rs5942187	X	PCDH11X	HGNC Symbol	protocadherin 11 X-linked [Source:HGNC Symbol;Acc:8656]
rs67310656	X	PCDH11X	HGNC Symbol	protocadherin 11 X-linked [Source:HGNC Symbol;Acc:8656]
rs10126983	X	PCDH11X	HGNC Symbol	protocadherin 11 X-linked [Source:HGNC Symbol;Acc:8656]
rs73245239	X	PCDH11X	HGNC Symbol	protocadherin 11 X-linked [Source:HGNC Symbol;Acc:8656]
rs138837079	X	PCDH11X	HGNC Symbol	protocadherin 11 X-linked [Source:HGNC Symbol;Acc:8656]
rs419619	X	PCDH11X	HGNC Symbol	protocadherin 11 X-linked [Source:HGNC Symbol;Acc:8656]
rs62598534	X	PCDH11X	HGNC Symbol	protocadherin 11 X-linked [Source:HGNC Symbol;Acc:8656]
rs147493192	X	PCDH11X	HGNC Symbol	protocadherin 11 X-linked [Source:HGNC Symbol;Acc:8656]
rs416075	X	PCDH11X	HGNC Symbol	protocadherin 11 X-linked [Source:HGNC Symbol;Acc:8656]
rs394756	X	PCDH11X	HGNC Symbol	protocadherin 11 X-linked [Source:HGNC Symbol;Acc:8656]
rs420721	X	PCDH11X	HGNC Symbol	protocadherin 11 X-linked [Source:HGNC Symbol;Acc:8656]
rs2755351	X	PCDH11X	HGNC Symbol	protocadherin 11 X-linked [Source:HGNC Symbol;Acc:8656]
rs2755353	X	PCDH11X	HGNC Symbol	protocadherin 11 X-linked [Source:HGNC Symbol;Acc:8656]
rs2522702	X	PCDH11X	HGNC Symbol	protocadherin 11 X-linked [Source:HGNC Symbol;Acc:8656]
rs7050013	X	PCDH11X	HGNC Symbol	protocadherin 11 X-linked [Source:HGNC Symbol;Acc:8656]
rs6618977	X	PCDH11X	HGNC Symbol	protocadherin 11 X-linked [Source:HGNC Symbol;Acc:8656]
rs182635933				
rs2573768	X	PCDH11X	HGNC Symbol	protocadherin 11 X-linked [Source:HGNC Symbol;Acc:8656]
rs2754930	X	PCDH11X	HGNC Symbol	protocadherin 11 X-linked [Source:HGNC Symbol;Acc:8656]
rs2984109	X	PCDH11X	HGNC Symbol	protocadherin 11 X-linked [Source:HGNC Symbol;Acc:8656]
rs2905672	X	PCDH11X	HGNC Symbol	protocadherin 11 X-linked [Source:HGNC Symbol;Acc:8656]

SNP	Chromosome	gene name	gene source	description
rs2754943				
rs147066277				
rs62596929	X	PCDH11X	HGNC Symbol	protocadherin 11 X-linked [Source:HGNC Symbol;Acc:8656]
rs2579004	X	PCDH11X	HGNC Symbol	protocadherin 11 X-linked [Source:HGNC Symbol;Acc:8656]
rs4020641	X	PCDH11X	HGNC Symbol	protocadherin 11 X-linked [Source:HGNC Symbol;Acc:8656]
rs141262908	X	PCDH11X	HGNC Symbol	protocadherin 11 X-linked [Source:HGNC Symbol;Acc:8656]
rs2755009	X	PCDH11X	HGNC Symbol	protocadherin 11 X-linked [Source:HGNC Symbol;Acc:8656]
rs4020637	X	PCDH11X	HGNC Symbol	protocadherin 11 X-linked [Source:HGNC Symbol;Acc:8656]
rs2578894				
rs75725248	X	PCDH11X	HGNC Symbol	protocadherin 11 X-linked [Source:HGNC Symbol;Acc:8656]
rs2556754	X	PCDH11X	HGNC Symbol	protocadherin 11 X-linked [Source:HGNC Symbol;Acc:8656]
rs5941089	X	PCDH11X	HGNC Symbol	protocadherin 11 X-linked [Source:HGNC Symbol;Acc:8656]
rs6615414	X	PCDH11X	HGNC Symbol	protocadherin 11 X-linked [Source:HGNC Symbol;Acc:8656]
rs145134624	X	PCDH11X	HGNC Symbol	protocadherin 11 X-linked [Source:HGNC Symbol;Acc:8656]
rs5941094	X	PCDH11X	HGNC Symbol	protocadherin 11 X-linked [Source:HGNC Symbol;Acc:8656]
rs184868100				
rs4893324	X	PCDH11X	HGNC Symbol	protocadherin 11 X-linked [Source:HGNC Symbol;Acc:8656]
rs138232719				
rs4020593	X	PCDH11X	HGNC Symbol	protocadherin 11 X-linked [Source:HGNC Symbol;Acc:8656]
rs192593398	X	PCDH11X	HGNC Symbol	protocadherin 11 X-linked [Source:HGNC Symbol;Acc:8656]
rs5984196	X	PCDH11X	HGNC Symbol	protocadherin 11 X-linked [Source:HGNC Symbol;Acc:8656]
rs3952482	X	PCDH11X	HGNC Symbol	protocadherin 11 X-linked [Source:HGNC Symbol;Acc:8656]
rs5985007	X	PCDH11X	HGNC Symbol	protocadherin 11 X-linked [Source:HGNC Symbol;Acc:8656]
rs7050375	X	PCDH11X	HGNC Symbol	protocadherin 11 X-linked [Source:HGNC Symbol;Acc:8656]
rs72608342	X	PCDH11X	HGNC Symbol	protocadherin 11 X-linked [Source:HGNC Symbol;Acc:8656]
rs79889385				
rs72608365				
rs2556983				
rs112142727				
rs3853040				
rs112602170				
rs62597414	X	RP13-258O15.1	Clone-based (Vega) gene	

SNP	Chromosome	gene name	gene source	description
rs2565376				
rs5985020				
rs112646833				
rs2751164				
rs2565570				
rs146303632				
rs4020239				
rs62598604				
rs142496506				
rs2506336				
rs2506326				
rs73247387				
rs62598626				
rs2499197				
rs2499186				
rs112141449				
rs34432535				
rs12010872				
rs2500760				
rs62598648				
rs2915078				
rs11795413				
rs111812394				
rs35572792				
rs6522586				
rs139805638				
rs35450030				
rs3853045				
rs785789				
rs137980088				
rs1854090				
rs113107407				
rs141853816				

SNP	Chromosome	gene name	gene source	description
rs36038905				
rs12841376				
rs785748				
rs73249532				
rs785754				
rs1578337				
rs55951278				
rs1198725				
rs5940044				
rs1198717				
rs1198718				
rs4892955				
rs6522621				
rs1402077				
rs1850987				
rs73249545				
rs7888166				
rs10283975				
rs7888584				
rs7887979				
rs5940148				
rs5940151				
rs17314027	X	RP3-455H14.1	Clone-based (Vega) gene	
rs2311603				
rs62598042				
rs35855911				
rs5940199				
rs73249594				
rs6615532				
rs56319256				
rs5940234				
rs7065953				

SNP	Chromosome	gene name	gene source	description
rs6619355				
rs1886990				
rs1326177				
rs1326176				
rs73252241				
rs5940304				
rs73252243				
rs73536296				
rs1045686	X	NAP1L3	HGNC Symbol	nucleosome assembly protein 1-like 3 [Source:HGNC Symbol;Acc:7639]
rs10126690	X	FAM133A	HGNC Symbol	family with sequence similarity 133, member A [Source:HGNC Symbol;Acc:26748]
rs4893043	X	FAM133A	HGNC Symbol	family with sequence similarity 133, member A [Source:HGNC Symbol;Acc:26748]
rs7891218	X	FAM133A	HGNC Symbol	family with sequence similarity 133, member A [Source:HGNC Symbol;Acc:26748]
rs10127399	X	FAM133A	HGNC Symbol	family with sequence similarity 133, member A [Source:HGNC Symbol;Acc:26748]
rs5940328				
rs73252252				
rs16983153				
rs1207446	X	RP1-60G11.1	Clone-based (Vega) gene	
rs73538015				
rs5939740				
rs62597059				
rs1207457				
rs5983260				
rs146788659				
rs6615562				
rs62595173				
rs1779393				
rs67824586				
rs7059961				
rs1797011				
rs62595187				
rs73635281				
rs5939789				

SNP	Chromosome	gene name	gene source	description
rs62595208				
rs5939805				
rs5939542				
rs4892973				
rs73254374				
rs5939835				
rs143279811				
rs5939837				
rs7883900				
rs5939553				
rs111391526				
rs1341936				
rs5939558				
rs5939859				
rs6619476				
rs5983168				
rs1416241				
rs146862255				
rs77873602				
rs5939876				
rs5939886				
rs5983342				
rs5983172				
rs17249431				
rs57380027				
rs56049550				
rs5939580				
rs57566357				
rs59128563				
rs56211197				
rs73254400				
rs111581267				
rs808777				

SNP	Chromosome	gene name	gene source	description
rs213675				
rs213678				
rs213707				
rs213721				
rs6615595				
rs12007582				
rs10745112				
rs6522763				
rs986952				
rs1458873				
rs73629712				
rs60540399				
rs2609513				
rs112212266				
rs2609526				
rs62595441				
rs2609529				
rs148269511				
rs5950165				
rs115438025				
rs2782744				
rs114786886				
rs2782730				
rs2782724				
rs2799879				
rs5949644				
rs6619549				
rs2862214				
rs6619554				
rs150481748				
rs73256544				
rs73256547				
rs72610589				

SNP	Chromosome	gene name	gene source	description
rs185837010				
rs72610593				
rs180892109				
rs111979499				
rs66864266				
rs6619612				
rs62597577				
rs148867584				
rs3130077				
rs17337207				
rs5949500				
rs4350137				
rs6615654				
rs12857888				
rs7880483				
rs4969705				
rs5990694				
rs57795725				
rs4969708				
rs4529644				
rs7065846				
rs60757162				
rs12115901				
rs73258520				
rs142234175				
rs6619694				
rs12557044				
rs73258533				
rs5949507				
rs6619708				
rs2800670				
rs5950127				
rs111314151				

SNP	Chromosome	gene name	gene source	description
rs62600659				
rs75617198				
rs2800668				
rs57689461				
rs5950163				
rs2354292				
rs139986284				
rs7061674				
rs5950204				
rs3131418				
rs6619774				
rs56132106				
rs5990771				
rs2028561				
rs73243027				
rs3131391				
rs73243095				
rs17328752				
rs145787710				
rs5990087				
rs146153752				
rs5949296				
rs3015161				
rs73245110				
rs2317958				
rs140281333				
rs12860169				
rs5990366				
rs150957528				
rs4469663				
rs73245118				
rs5949599				
rs111335229				

SNP	Chromosome	gene name	gene source	description
rs111834131				
rs62600378				
rs4142818				
rs6619889				
rs1573255				
rs10126465				
rs5949324				
rs5990142				
rs61612305				
rs5949646				
rs62600417				
rs5990410				
rs5949654				
rs5990411				
rs5949660				
rs5949661				
rs5990417				
rs5949328				
rs73245199				
rs6522944				
rs66627368				
rs146263478				
rs5949676				
rs73635766				
rs75009304				
rs12559642				
rs144215478				
rs5949343				
rs72612525				
rs6619936				
rs73247124				
rs5990155				
rs5949349				

SNP	Chromosome	gene name	gene source	description
rs72612532				
rs73247135				
rs2805995				
rs2317472				
rs1590366				
rs2806020				
rs2476579				
rs5949759				
rs62600809				
rs58491501				
rs5949365				
rs942275				
rs12832483				
rs5949366				
rs2147053				
rs2743683				
rs142097098				
rs12856143				
rs17337541				
rs2808765				
rs111460176				
rs73247163				
rs5949377				
rs12852940				
rs146552642				
rs17328926				
rs1737302				
rs1206664				
rs1150286				
rs4969585				
rs1933408				
rs2803384				
rs17333535				

SNP	Chromosome	gene name	gene source	description
rs141222176				
rs7881274				
rs73247183				
rs7886045				
rs73247184				
rs6615806				
rs12011697				
rs7055508				
rs5949848				
rs5949410				
rs113911348				
rs5949868				
rs4463614				
rs57343194				
rs149444039				
rs58936690				
rs4969675				
rs5949878				
rs5949879				
rs4969678				
rs5949884				
rs17282205				
rs17337770				
rs5949890				
rs4639693				
rs12837907				
rs66616254				
rs5949895				
rs5949901				
rs6620098				
rs58048126				
rs4370708				
rs5949428				

SNP	Chromosome	gene name	gene source	description
rs5949434				
rs5949950				
rs5903056				
rs73249102				
rs5949445				
rs190961953	X	DIAPH2	HGNC Symbol	diaphanous-related formin 2 [Source:HGNC Symbol;Acc:2877]
rs7058735	X	DIAPH2	HGNC Symbol	diaphanous-related formin 2 [Source:HGNC Symbol;Acc:2877]
rs150518592	X	DIAPH2	HGNC Symbol	diaphanous-related formin 2 [Source:HGNC Symbol;Acc:2877]
rs6620152	X	DIAPH2	HGNC Symbol	diaphanous-related formin 2 [Source:HGNC Symbol;Acc:2877]
rs20363	X	DIAPH2	HGNC Symbol	diaphanous-related formin 2 [Source:HGNC Symbol;Acc:2877]
rs442950	X	DIAPH2	HGNC Symbol	diaphanous-related formin 2 [Source:HGNC Symbol;Acc:2877]
rs73250737	X	DIAPH2	HGNC Symbol	diaphanous-related formin 2 [Source:HGNC Symbol;Acc:2877]
rs409454	X	DIAPH2	HGNC Symbol	diaphanous-related formin 2 [Source:HGNC Symbol;Acc:2877]
rs417698	X	DIAPH2	HGNC Symbol	diaphanous-related formin 2 [Source:HGNC Symbol;Acc:2877]
rs413169	X	DIAPH2	HGNC Symbol	diaphanous-related formin 2 [Source:HGNC Symbol;Acc:2877]
rs233688	X	DIAPH2	HGNC Symbol	diaphanous-related formin 2 [Source:HGNC Symbol;Acc:2877]
rs17256465	X	DIAPH2;RPA4	HGNC Symbol	diaphanous-related formin 2 [Source:HGNC Symbol;Acc:2877];replication protein A4, 30kDa [Source:HGNC Symbol;Acc:30305]
rs2642219	X	DIAPH2;RPA4	HGNC Symbol	diaphanous-related formin 2 [Source:HGNC Symbol;Acc:2877];replication protein A4, 30kDa [Source:HGNC Symbol;Acc:30305]
rs2642218	X	DIAPH2;RPA4	HGNC Symbol	diaphanous-related formin 2 [Source:HGNC Symbol;Acc:2877];replication protein A4, 30kDa [Source:HGNC Symbol;Acc:30305]
rs707287	X	DIAPH2	HGNC Symbol	diaphanous-related formin 2 [Source:HGNC Symbol;Acc:2877]
rs829285	X	DIAPH2	HGNC Symbol	diaphanous-related formin 2 [Source:HGNC Symbol;Acc:2877]
rs181520373	X	DIAPH2	HGNC Symbol	diaphanous-related formin 2 [Source:HGNC Symbol;Acc:2877]
rs73250766	X	DIAPH2	HGNC Symbol	diaphanous-related formin 2 [Source:HGNC Symbol;Acc:2877]
rs41310689	X	DIAPH2	HGNC Symbol	diaphanous-related formin 2 [Source:HGNC Symbol;Acc:2877]
rs5920990	X	DIAPH2	HGNC Symbol	diaphanous-related formin 2 [Source:HGNC Symbol;Acc:2877]
rs5966836	X	DIAPH2	HGNC Symbol	diaphanous-related formin 2 [Source:HGNC Symbol;Acc:2877]
rs148935786	X	DIAPH2	HGNC Symbol	diaphanous-related formin 2 [Source:HGNC Symbol;Acc:2877]
rs5921097	X	DIAPH2	HGNC Symbol	diaphanous-related formin 2 [Source:HGNC Symbol;Acc:2877]
rs1040449	X	DIAPH2	HGNC Symbol	diaphanous-related formin 2 [Source:HGNC Symbol;Acc:2877]
rs35708312	X	DIAPH2	HGNC Symbol	diaphanous-related formin 2 [Source:HGNC Symbol;Acc:2877]
rs112195754	X	DIAPH2	HGNC Symbol	diaphanous-related formin 2 [Source:HGNC Symbol;Acc:2877]

SNP	Chromosome	gene name	gene source	description
rs11796386	X	DIAPH2	HGNC Symbol	diaphanous-related formin 2 [Source:HGNC Symbol;Acc:2877]
rs5920720	X	DIAPH2	HGNC Symbol	diaphanous-related formin 2 [Source:HGNC Symbol;Acc:2877]
rs661342	X	DIAPH2	HGNC Symbol	diaphanous-related formin 2 [Source:HGNC Symbol;Acc:2877]
rs144559575	X	DIAPH2	HGNC Symbol	diaphanous-related formin 2 [Source:HGNC Symbol;Acc:2877]
rs148673965	X	DIAPH2	HGNC Symbol	diaphanous-related formin 2 [Source:HGNC Symbol;Acc:2877]
rs5921341	X	DIAPH2	HGNC Symbol	diaphanous-related formin 2 [Source:HGNC Symbol;Acc:2877]
rs600455	X	DIAPH2	HGNC Symbol	diaphanous-related formin 2 [Source:HGNC Symbol;Acc:2877]
rs926407	X	DIAPH2	HGNC Symbol	diaphanous-related formin 2 [Source:HGNC Symbol;Acc:2877]
rs73250785	X	DIAPH2	HGNC Symbol	diaphanous-related formin 2 [Source:HGNC Symbol;Acc:2877]
rs5921403	X	DIAPH2	HGNC Symbol	diaphanous-related formin 2 [Source:HGNC Symbol;Acc:2877]
rs12857090	X	DIAPH2	HGNC Symbol	diaphanous-related formin 2 [Source:HGNC Symbol;Acc:2877]
rs12394769	X	DIAPH2	HGNC Symbol	diaphanous-related formin 2 [Source:HGNC Symbol;Acc:2877]
rs148639505	X	DIAPH2	HGNC Symbol	diaphanous-related formin 2 [Source:HGNC Symbol;Acc:2877]
rs5920815	X	DIAPH2	HGNC Symbol	diaphanous-related formin 2 [Source:HGNC Symbol;Acc:2877]
rs73250794	X	DIAPH2	HGNC Symbol	diaphanous-related formin 2 [Source:HGNC Symbol;Acc:2877]
rs143432964	X	DIAPH2	HGNC Symbol	diaphanous-related formin 2 [Source:HGNC Symbol;Acc:2877]
rs11796806	X	DIAPH2	HGNC Symbol	diaphanous-related formin 2 [Source:HGNC Symbol;Acc:2877]
rs73258349	X	DIAPH2	HGNC Symbol	diaphanous-related formin 2 [Source:HGNC Symbol;Acc:2877]
rs35408243	X	DIAPH2	HGNC Symbol	diaphanous-related formin 2 [Source:HGNC Symbol;Acc:2877]
rs5921803	X	DIAPH2	HGNC Symbol	diaphanous-related formin 2 [Source:HGNC Symbol;Acc:2877]
rs12008065	X	DIAPH2;DIAPH2-AS1	HGNC Symbol	diaphanous-related formin 2 [Source:HGNC Symbol;Acc:2877];DIAPH2 antisense RNA 1 [Source:HGNC Symbol;Acc:16972]
rs142948510	X	DIAPH2;DIAPH2-AS1	HGNC Symbol	diaphanous-related formin 2 [Source:HGNC Symbol;Acc:2877];DIAPH2 antisense RNA 1 [Source:HGNC Symbol;Acc:16972]
rs11798175	X	DIAPH2;DIAPH2-AS1	HGNC Symbol	diaphanous-related formin 2 [Source:HGNC Symbol;Acc:2877];DIAPH2 antisense RNA 1 [Source:HGNC Symbol;Acc:16972]
rs12847298	X	DIAPH2;DIAPH2-AS1	HGNC Symbol	diaphanous-related formin 2 [Source:HGNC Symbol;Acc:2877];DIAPH2 antisense RNA 1 [Source:HGNC Symbol;Acc:16972]
rs73554324	X	DIAPH2;DIAPH2-AS1	HGNC Symbol	diaphanous-related formin 2 [Source:HGNC Symbol;Acc:2877];DIAPH2 antisense RNA 1 [Source:HGNC Symbol;Acc:16972]
rs4828088	X	DIAPH2;DIAPH2-AS1	HGNC Symbol	diaphanous-related formin 2 [Source:HGNC Symbol;Acc:2877];DIAPH2 antisense RNA 1 [Source:HGNC Symbol;Acc:16972]
rs5967324	X	DIAPH2;DIAPH2-AS1	HGNC Symbol	diaphanous-related formin 2 [Source:HGNC Symbol;Acc:2877];DIAPH2 antisense RNA 1 [Source:HGNC Symbol;Acc:16972]
rs62594827	X	DIAPH2;DIAPH2-AS1	HGNC Symbol	diaphanous-related formin 2 [Source:HGNC Symbol;Acc:2877];DIAPH2 antisense RNA 1 [Source:HGNC Symbol;Acc:16972]

SNP	Chromosome	gene name	gene source	description
rs146858325	X	DIAPH2;DIAPH2-AS1	HGNC Symbol	diaphanous-related formin 2 [Source:HGNC Symbol;Acc:2877];DIAPH2 antisense RNA 1 [Source:HGNC Symbol;Acc:16972]
rs17282304	X	DIAPH2;DIAPH2-AS1	HGNC Symbol	diaphanous-related formin 2 [Source:HGNC Symbol;Acc:2877];DIAPH2 antisense RNA 1 [Source:HGNC Symbol;Acc:16972]
rs5967328	X	DIAPH2;DIAPH2-AS1	HGNC Symbol	diaphanous-related formin 2 [Source:HGNC Symbol;Acc:2877];DIAPH2 antisense RNA 1 [Source:HGNC Symbol;Acc:16972]
rs6620311	X	DIAPH2;DIAPH2-AS1	HGNC Symbol	diaphanous-related formin 2 [Source:HGNC Symbol;Acc:2877];DIAPH2 antisense RNA 1 [Source:HGNC Symbol;Acc:16972]
rs363759	X	DIAPH2;DIAPH2-AS1	HGNC Symbol	diaphanous-related formin 2 [Source:HGNC Symbol;Acc:2877];DIAPH2 antisense RNA 1 [Source:HGNC Symbol;Acc:16972]
rs111472373	X	DIAPH2-AS1	HGNC Symbol	DIAPH2 antisense RNA 1 [Source:HGNC Symbol;Acc:16972]
rs5967338	X	DIAPH2-AS1	HGNC Symbol	DIAPH2 antisense RNA 1 [Source:HGNC Symbol;Acc:16972]
rs12862340	X	DIAPH2-AS1	HGNC Symbol	DIAPH2 antisense RNA 1 [Source:HGNC Symbol;Acc:16972]
rs138140181	X	DIAPH2-AS1	HGNC Symbol	DIAPH2 antisense RNA 1 [Source:HGNC Symbol;Acc:16972]
rs73258371				
rs60098214				
rs60646996				
rs5967343				
rs73258373				
rs6620349				
rs6620351				
rs79369846				
rs2497889				
rs4281241				
rs142111827				
rs6620366				
rs10481982				
rs1383651				
rs1021072				
rs5967373				
rs5920955				
rs5920960				
rs318187				
rs318185				
rs318181				

SNP	Chromosome	gene name	gene source	description
rs145349078				
rs318177				
rs318173				
rs318171				
rs17003793				
rs400586				
rs11092162				
rs318138				
rs318132				
rs4827909	X	ERVWE2	Clone-based (Vega) gene	
rs17333695	X	ERVWE2	Clone-based (Vega) gene	
rs2379168				
rs170320				
rs318167				
rs149568341				
rs6523129				
rs139241665				
rs17329162				
rs17282380				
rs73260463				
rs5920614	X	RP13-130D24.1	Clone-based (Vega) gene	
rs147576701				
rs5920984				
rs1453333				
rs73550297				
rs73243611				
rs5966581				
rs4827818				
rs143460286				
rs5921026				
rs12835601				

SNP	Chromosome	gene name	gene source	description
rs6620461				
rs151067868				
rs6620471				
rs147394583				
rs946301				
rs6615988				
rs5920657	X	EEF1A1P15	HGNC Symbol	eukaryotic translation elongation factor 1 alpha 1 pseudogene 15 [Source:HGNC Symbol;Acc:3198]
rs5921084				
rs1890447				
rs5920659				
rs73243639				
rs5921103				
rs113294980				
rs1933800				
rs5921106				
rs5921108				
rs184613753				
rs5966855				
rs55906722				
rs17322555				
rs4145194				
rs142426830				
rs6616015				
rs1935438				
rs143042025				
rs4827947				
rs6620556				
rs7888925				
rs7062726				
rs17322597				
rs4827948				
rs241852				
rs17282025				

SNP	Chromosome	gene name	gene source	description
rs5921201				
rs140580619				
rs17256765				
rs1983897				
rs6620598				
rs2498898				
rs5920707				
rs5966900				
rs5966901				
rs2473218				
rs16982961				
rs7889840				
rs4827953				
rs112879811				
rs16982964				
rs73246404				
rs112088729				
rs5920719				
rs17322758				
rs5921285				
rs73246412				
rs2707952				
rs5920732				
rs996341				
rs5966950				
rs1024199				
rs143443060				
rs73246418				
rs767772				
rs140984023				
rs112247079				
rs111819846				
rs149003052				

SNP	Chromosome	gene name	gene source	description
rs12557850	X	XRCC6P5	HGNC Symbol	X-ray repair complementing defective repair in Chinese hamster cells 6 pseudogene 5 [Source:HGNC Symbol;Acc:45187]
rs5966968	X	XRCC6P5	HGNC Symbol	X-ray repair complementing defective repair in Chinese hamster cells 6 pseudogene 5 [Source:HGNC Symbol;Acc:45187]
rs6620700	X	XRCC6P5	HGNC Symbol	X-ray repair complementing defective repair in Chinese hamster cells 6 pseudogene 5 [Source:HGNC Symbol;Acc:45187]
rs6616087	X	XRCC6P5	HGNC Symbol	X-ray repair complementing defective repair in Chinese hamster cells 6 pseudogene 5 [Source:HGNC Symbol;Acc:45187]
rs12841845	X	XRCC6P5	HGNC Symbol	X-ray repair complementing defective repair in Chinese hamster cells 6 pseudogene 5 [Source:HGNC Symbol;Acc:45187]
rs56173628	X	XRCC6P5	HGNC Symbol	X-ray repair complementing defective repair in Chinese hamster cells 6 pseudogene 5 [Source:HGNC Symbol;Acc:45187]
rs149843107	X	XRCC6P5	HGNC Symbol	X-ray repair complementing defective repair in Chinese hamster cells 6 pseudogene 5 [Source:HGNC Symbol;Acc:45187]
rs12687843	X	XRCC6P5	HGNC Symbol	X-ray repair complementing defective repair in Chinese hamster cells 6 pseudogene 5 [Source:HGNC Symbol;Acc:45187]
rs35209009	X	XRCC6P5	HGNC Symbol	X-ray repair complementing defective repair in Chinese hamster cells 6 pseudogene 5 [Source:HGNC Symbol;Acc:45187]
rs138121507	X	XRCC6P5	HGNC Symbol	X-ray repair complementing defective repair in Chinese hamster cells 6 pseudogene 5 [Source:HGNC Symbol;Acc:45187]
rs17322793	X	XRCC6P5	HGNC Symbol	X-ray repair complementing defective repair in Chinese hamster cells 6 pseudogene 5 [Source:HGNC Symbol;Acc:45187]
rs112892503	X	XRCC6P5	HGNC Symbol	X-ray repair complementing defective repair in Chinese hamster cells 6 pseudogene 5 [Source:HGNC Symbol;Acc:45187]
rs141182486				
rs1343474				
rs62614761				
rs6620737				
rs5921379				
rs4501730				
rs5921392				
rs17322905				
rs5920778				
rs7053289				
rs73543943				
rs73557954				
rs62610479				

SNP	Chromosome	gene name	gene source	description
rs5921443				
rs5967045				
rs5921455				
rs61484236				
rs5920794				
rs5921463				
rs73246490				
rs7062296				
rs7064414				
rs12864057				
rs73246501				
rs73248405				
rs17257104				
rs5967083				
rs1832751				
rs56003729				
rs6620832				
rs4828001				
rs1341079				
rs1341080				
rs17328938				
rs5921531				
rs17282282				
rs1986391	X	PCDH19	HGNC Symbol	protocadherin 19 [Source:HGNC Symbol;Acc:14270]
rs12690257	X	PCDH19	HGNC Symbol	protocadherin 19 [Source:HGNC Symbol;Acc:14270]
rs5920818	X	PCDH19	HGNC Symbol	protocadherin 19 [Source:HGNC Symbol;Acc:14270]
rs11797294	X	PCDH19	HGNC Symbol	protocadherin 19 [Source:HGNC Symbol;Acc:14270]
rs5920819	X	PCDH19	HGNC Symbol	protocadherin 19 [Source:HGNC Symbol;Acc:14270]
rs28545751	X	PCDH19	HGNC Symbol	protocadherin 19 [Source:HGNC Symbol;Acc:14270]
rs7885942	X	PCDH19	HGNC Symbol	protocadherin 19 [Source:HGNC Symbol;Acc:14270]
rs55805304	X	PCDH19	HGNC Symbol	protocadherin 19 [Source:HGNC Symbol;Acc:14270]
rs17282303	X	PCDH19	HGNC Symbol	protocadherin 19 [Source:HGNC Symbol;Acc:14270]
rs1953337	X	PCDH19	HGNC Symbol	protocadherin 19 [Source:HGNC Symbol;Acc:14270]

SNP	Chromosome	gene name	gene source	description
rs41300169	X	PCDH19	HGNC Symbol	protocadherin 19 [Source:HGNC Symbol;Acc:14270]
rs7055859				
rs62600479				
rs5967134				
rs73248490				
rs4991657				
rs16983501				
rs72615552				
rs5967145				
rs34429223				
rs11092266				
rs16983508				
rs720403				
rs12557224				
rs5921594				
rs6616166				
rs58071178				
rs7059391				
rs873275				
rs1540914	X	TNMD	HGNC Symbol	tenomodulin [Source:HGNC Symbol;Acc:17757]
rs5921613	X	TNMD	HGNC Symbol	tenomodulin [Source:HGNC Symbol;Acc:17757]
rs7060407	X	TNMD	HGNC Symbol	tenomodulin [Source:HGNC Symbol;Acc:17757]
rs11092277	X	TNMD	HGNC Symbol	tenomodulin [Source:HGNC Symbol;Acc:17757]
rs56957755	X	TNMD	HGNC Symbol	tenomodulin [Source:HGNC Symbol;Acc:17757]
rs2073162	X	TNMD	HGNC Symbol	tenomodulin [Source:HGNC Symbol;Acc:17757]
rs4828038	X	TNMD	HGNC Symbol	tenomodulin [Source:HGNC Symbol;Acc:17757]
rs6616168				
rs1204386				
rs1204387				
rs1204394				
rs1204396				
rs1802288	X	TSPAN6	HGNC Symbol	tetraspanin 6 [Source:HGNC Symbol;Acc:11858]
rs6523390				

SNP	Chromosome	gene name	gene source	description
rs1343213	X	SRPX2	HGNC Symbol	sushi-repeat containing protein, X-linked 2 [Source:HGNC Symbol;Acc:30668]
rs5967161	X	SRPX2	HGNC Symbol	sushi-repeat containing protein, X-linked 2 [Source:HGNC Symbol;Acc:30668]
rs1204411	X	SRPX2	HGNC Symbol	sushi-repeat containing protein, X-linked 2 [Source:HGNC Symbol;Acc:30668]
rs143874356	X	SRPX2	HGNC Symbol	sushi-repeat containing protein, X-linked 2 [Source:HGNC Symbol;Acc:30668]
rs1419017	X	SRPX2	HGNC Symbol	sushi-repeat containing protein, X-linked 2 [Source:HGNC Symbol;Acc:30668]
rs113945055	X	SRPX2	HGNC Symbol	sushi-repeat containing protein, X-linked 2 [Source:HGNC Symbol;Acc:30668]
rs112915821				
rs143469082	X	SYTL4	HGNC Symbol	synaptotagmin-like 4 [Source:HGNC Symbol;Acc:15588]
rs6620911	X	SYTL4	HGNC Symbol	synaptotagmin-like 4 [Source:HGNC Symbol;Acc:15588]
rs17174169	X	SYTL4	HGNC Symbol	synaptotagmin-like 4 [Source:HGNC Symbol;Acc:15588]
rs5967170	X	SYTL4	HGNC Symbol	synaptotagmin-like 4 [Source:HGNC Symbol;Acc:15588]
rs73557582	X	SYTL4	HGNC Symbol	synaptotagmin-like 4 [Source:HGNC Symbol;Acc:15588]
rs113537287	X	SYTL4	HGNC Symbol	synaptotagmin-like 4 [Source:HGNC Symbol;Acc:15588]
rs11798365				
rs73250616				
rs2154370				
rs11092279				
rs1794471				
rs3213559	X	CSTF2	HGNC Symbol	cleavage stimulation factor, 3' pre-RNA, subunit 2, 64kDa [Source:HGNC Symbol;Acc:2484]
rs5920863	X	CSTF2	HGNC Symbol	cleavage stimulation factor, 3' pre-RNA, subunit 2, 64kDa [Source:HGNC Symbol;Acc:2484]
rs150725322	X	CSTF2	HGNC Symbol	cleavage stimulation factor, 3' pre-RNA, subunit 2, 64kDa [Source:HGNC Symbol;Acc:2484]
rs34688635	X	NOX1	HGNC Symbol	NADPH oxidase 1 [Source:HGNC Symbol;Acc:7889]
rs4828068	X	NOX1	HGNC Symbol	NADPH oxidase 1 [Source:HGNC Symbol;Acc:7889]
rs73561355	X	NOX1;HNRNPA1P26	HGNC Symbol	NADPH oxidase 1 [Source:HGNC Symbol;Acc:7889];heterogeneous nuclear ribonucleoprotein A1 pseudogene 26 [Source:HGNC Symbol;Acc:39544]
rs16983650	X	NOX1	HGNC Symbol	NADPH oxidase 1 [Source:HGNC Symbol;Acc:7889]
rs4827881				
rs6620949				
rs10126566				
rs17257460				
rs5967207				
rs17323346				
rs5966722				

SNP	Chromosome	gene name	gene source	description
rs5921687				
rs5921690				
rs7128	X	LL0XNC01-131B10.2	Clone-based (Vega) gene	
rs5920872				
rs1204418	X	XKRX	HGNC Symbol	XK, Kell blood group complex subunit-related, X-linked [Source:HGNC Symbol;Acc:29845]
rs12842818				
rs17282641				
rs7882094				
rs60097212	X	ARL13A	HGNC Symbol	ADP-ribosylation factor-like 13A [Source:HGNC Symbol;Acc:31709]
rs147105321	X	ARL13A	HGNC Symbol	ADP-ribosylation factor-like 13A [Source:HGNC Symbol;Acc:31709]
rs5921701	X	ARL13A	HGNC Symbol	ADP-ribosylation factor-like 13A [Source:HGNC Symbol;Acc:31709]
rs17257516	X	ARL13A	HGNC Symbol	ADP-ribosylation factor-like 13A [Source:HGNC Symbol;Acc:31709]
rs41307262	X	ARL13A	HGNC Symbol	ADP-ribosylation factor-like 13A [Source:HGNC Symbol;Acc:31709]
rs3934462	X	ARL13A	HGNC Symbol	ADP-ribosylation factor-like 13A [Source:HGNC Symbol;Acc:31709]
rs7885599	X	TRMT2B	HGNC Symbol	tRNA methyltransferase 2 homolog B (S. cerevisiae) [Source:HGNC Symbol;Acc:25748]
rs139231094	X	TRMT2B	HGNC Symbol	tRNA methyltransferase 2 homolog B (S. cerevisiae) [Source:HGNC Symbol;Acc:25748]
rs56176072	X	TRMT2B	HGNC Symbol	tRNA methyltransferase 2 homolog B (S. cerevisiae) [Source:HGNC Symbol;Acc:25748]
rs5921710	X	TRMT2B	HGNC Symbol	tRNA methyltransferase 2 homolog B (S. cerevisiae) [Source:HGNC Symbol;Acc:25748]
rs5921712	X	TRMT2B;TRMT2B-AS1	HGNC Symbol	tRNA methyltransferase 2 homolog B (S. cerevisiae) [Source:HGNC Symbol;Acc:25748];TRMT2B antisense RNA 1 [Source:HGNC Symbol;Acc:41116]
rs111917350	X	TRMT2B;TRMT2B-AS1	HGNC Symbol	tRNA methyltransferase 2 homolog B (S. cerevisiae) [Source:HGNC Symbol;Acc:25748];TRMT2B antisense RNA 1 [Source:HGNC Symbol;Acc:41116]
rs17257536	X	TRMT2B-AS1	HGNC Symbol	TRMT2B antisense RNA 1 [Source:HGNC Symbol;Acc:41116]
rs113487380	X	TRMT2B-AS1	HGNC Symbol	TRMT2B antisense RNA 1 [Source:HGNC Symbol;Acc:41116]
rs35451003	X	TRMT2B-AS1	HGNC Symbol	TRMT2B antisense RNA 1 [Source:HGNC Symbol;Acc:41116]
rs12156914	X	TRMT2B-AS1	HGNC Symbol	TRMT2B antisense RNA 1 [Source:HGNC Symbol;Acc:41116]
rs143759617	X	TMEM35;TRMT2B-AS1	HGNC Symbol	transmembrane protein 35 [Source:HGNC Symbol;Acc:25864];TRMT2B antisense RNA 1 [Source:HGNC Symbol;Acc:41116]
rs41304050	X	TMEM35;TRMT2B-AS1	HGNC Symbol	transmembrane protein 35 [Source:HGNC Symbol;Acc:25864];TRMT2B antisense RNA 1 [Source:HGNC Symbol;Acc:41116]
rs34994578	X	TMEM35	HGNC Symbol	transmembrane protein 35 [Source:HGNC Symbol;Acc:25864]
rs60742597	X	CENPI	HGNC Symbol	centromere protein I [Source:HGNC Symbol;Acc:3968]
rs112343581	X	CENPI	HGNC Symbol	centromere protein I [Source:HGNC Symbol;Acc:3968]
rs144142416				

SNP	Chromosome	gene name	gene source	description
rs111477175				
rs5921747				
rs56253313				
rs145211237	X	DRP2	HGNC Symbol	dystrophin related protein 2 [Source:HGNC Symbol;Acc:3032]
rs34013624	X	DRP2	HGNC Symbol	dystrophin related protein 2 [Source:HGNC Symbol;Acc:3032]
rs7066252	X	DRP2	HGNC Symbol	dystrophin related protein 2 [Source:HGNC Symbol;Acc:3032]
rs5921756	X	DRP2	HGNC Symbol	dystrophin related protein 2 [Source:HGNC Symbol;Acc:3032]
rs17257634	X	DRP2	HGNC Symbol	dystrophin related protein 2 [Source:HGNC Symbol;Acc:3032]
rs2238993	X	DRP2	HGNC Symbol	dystrophin related protein 2 [Source:HGNC Symbol;Acc:3032]
rs56230120	X	DRP2	HGNC Symbol	dystrophin related protein 2 [Source:HGNC Symbol;Acc:3032]
rs876152				
rs73250679	X	TAF7L	HGNC Symbol	TAF7-like RNA polymerase II, TATA box binding protein (TBP)-associated factor, 50kDa [Source:HGNC Symbol;Acc:11548]
rs35899692	X	TAF7L	HGNC Symbol	TAF7-like RNA polymerase II, TATA box binding protein (TBP)-associated factor, 50kDa [Source:HGNC Symbol;Acc:11548]
rs140510490	X	TAF7L	HGNC Symbol	TAF7-like RNA polymerase II, TATA box binding protein (TBP)-associated factor, 50kDa [Source:HGNC Symbol;Acc:11548]
rs6621026	X	TAF7L	HGNC Symbol	TAF7-like RNA polymerase II, TATA box binding protein (TBP)-associated factor, 50kDa [Source:HGNC Symbol;Acc:11548]
rs2180271	X	TAF7L	HGNC Symbol	TAF7-like RNA polymerase II, TATA box binding protein (TBP)-associated factor, 50kDa [Source:HGNC Symbol;Acc:11548]
rs79990723				
rs151101436				
rs6621038				
rs5991981				
rs58400762				
rs73250680				
rs3027657				
rs72615576				
rs41309506	X	TIMM8A	HGNC Symbol	translocase of inner mitochondrial membrane 8 homolog A (yeast) [Source:HGNC Symbol;Acc:11817]
rs1057403	X;HG1439_PATCH	BTK	HGNC Symbol	Bruton agammaglobulinemia tyrosine kinase [Source:HGNC Symbol;Acc:1133]
rs1135363	X	BTK	HGNC Symbol	Bruton agammaglobulinemia tyrosine kinase [Source:HGNC Symbol;Acc:1133]
rs2855259	X;HG1439_PATCH	BTK	HGNC Symbol	Bruton agammaglobulinemia tyrosine kinase [Source:HGNC Symbol;Acc:1133]

SNP	Chromosome	gene name	gene source	description
rs7053244	X	BTK	HGNC Symbol	Bruton agammaglobulinemia tyrosine kinase [Source:HGNC Symbol;Acc:1133]
rs73250681	X;HG1439_PATCH	BTK	HGNC Symbol	Bruton agammaglobulinemia tyrosine kinase [Source:HGNC Symbol;Acc:1133]
rs3027627	X;HG1439_PATCH	BTK	HGNC Symbol	Bruton agammaglobulinemia tyrosine kinase [Source:HGNC Symbol;Acc:1133]
rs2239462	X;HG1439_PATCH	BTK	HGNC Symbol	Bruton agammaglobulinemia tyrosine kinase [Source:HGNC Symbol;Acc:1133]
rs3027607	X;HG1439_PATCH	BTK	HGNC Symbol	Bruton agammaglobulinemia tyrosine kinase [Source:HGNC Symbol;Acc:1133]
rs2239460	X	BTK	HGNC Symbol	Bruton agammaglobulinemia tyrosine kinase [Source:HGNC Symbol;Acc:1133]
rs36222103				
rs3027594	X;HG1439_PATCH	RPL36A;RPL36A-HNRNPH2	HGNC Symbol	ribosomal protein L36a [Source:HGNC Symbol;Acc:10359];RPL36A-HNRNPH2 readthrough [Source:HGNC Symbol;Acc:48349]
rs2515901	X	RPL36A-HNRNPH2	HGNC Symbol	RPL36A-HNRNPH2 readthrough [Source:HGNC Symbol;Acc:48349]
rs2071397	X;HG1439_PATCH	GLA;RPL36A-HNRNPH2	HGNC Symbol	galactosidase, alpha [Source:HGNC Symbol;Acc:4296];RPL36A-HNRNPH2 readthrough [Source:HGNC Symbol;Acc:48349]
rs2071225	X;HG1439_PATCH	GLA;RPL36A-HNRNPH2	HGNC Symbol	galactosidase, alpha [Source:HGNC Symbol;Acc:4296];RPL36A-HNRNPH2 readthrough [Source:HGNC Symbol;Acc:48349]
rs3027585	X;HG1439_PATCH	GLA;RPL36A-HNRNPH2	HGNC Symbol	galactosidase, alpha [Source:HGNC Symbol;Acc:4296];RPL36A-HNRNPH2 readthrough [Source:HGNC Symbol;Acc:48349]
rs3027584	X;HG1439_PATCH	RPL36A-HNRNPH2	HGNC Symbol	RPL36A-HNRNPH2 readthrough [Source:HGNC Symbol;Acc:48349]
rs3027578	X;HG1439_PATCH	HNRNPH2;RPL36A-HNRNPH2	HGNC Symbol	heterogeneous nuclear ribonucleoprotein H2 (H') [Source:HGNC Symbol;Acc:5042];RPL36A-HNRNPH2 readthrough [Source:HGNC Symbol;Acc:48349]
rs5991904	X;HG1439_PATCH	ARMCX4	HGNC Symbol	armadillo repeat containing, X-linked 4 [Source:HGNC Symbol;Acc:28615]
rs139143003	X;HG1439_PATCH	ARMCX4	HGNC Symbol	armadillo repeat containing, X-linked 4 [Source:HGNC Symbol;Acc:28615]
rs2078204	X;HG1439_PATCH	ARMCX4	HGNC Symbol	armadillo repeat containing, X-linked 4 [Source:HGNC Symbol;Acc:28615]
rs144262560	X;HG1439_PATCH	ARMCX4	HGNC Symbol	armadillo repeat containing, X-linked 4 [Source:HGNC Symbol;Acc:28615]
rs3027567	X;HG1439_PATCH	ARMCX4;RP1-164F3.8	HGNC Symbol;Clone-based (Vega) gene	armadillo repeat containing, X-linked 4 [Source:HGNC Symbol;Acc:28615];
rs3027566	X;HG1439_PATCH	ARMCX4	HGNC Symbol	armadillo repeat containing, X-linked 4 [Source:HGNC Symbol;Acc:28615]
rs4388621	X	ARMCX4	HGNC Symbol	armadillo repeat containing, X-linked 4 [Source:HGNC Symbol;Acc:28615]

SNP	Chromosome	gene name	gene source	description
rs113402053	X;HG1439_PATCH	ARMCX4	HGNC Symbol	armadillo repeat containing, X-linked 4 [Source:HGNC Symbol;Acc:28615]
rs188385227	X;HG1439_PATCH	ARMCX4	HGNC Symbol	armadillo repeat containing, X-linked 4 [Source:HGNC Symbol;Acc:28615]
rs3827421	X;HG1439_PATCH	ARMCX4	HGNC Symbol	armadillo repeat containing, X-linked 4 [Source:HGNC Symbol;Acc:28615]
rs2253062	X;HG1439_PATCH	ARMCX4	HGNC Symbol	armadillo repeat containing, X-linked 4 [Source:HGNC Symbol;Acc:28615]
rs963618	X;HG1439_PATCH	ARMCX4	HGNC Symbol	armadillo repeat containing, X-linked 4 [Source:HGNC Symbol;Acc:28615]
rs5951332	X;HG1439_PATCH	ARMCX4	HGNC Symbol	armadillo repeat containing, X-linked 4 [Source:HGNC Symbol;Acc:28615]
rs5951333	X;HG1439_PATCH	ARMCX4	HGNC Symbol	armadillo repeat containing, X-linked 4 [Source:HGNC Symbol;Acc:28615]
rs61736018	X;HG1439_PATCH	ARMCX4	HGNC Symbol	armadillo repeat containing, X-linked 4 [Source:HGNC Symbol;Acc:28615]
rs3174476	X;HG1439_PATCH	ARMCX4	HGNC Symbol	armadillo repeat containing, X-linked 4 [Source:HGNC Symbol;Acc:28615]
rs73250691	X;HG1439_PATCH	ARMCX4	HGNC Symbol	armadillo repeat containing, X-linked 4 [Source:HGNC Symbol;Acc:28615]
rs7060868	X	ARMCX4	HGNC Symbol	armadillo repeat containing, X-linked 4 [Source:HGNC Symbol;Acc:28615]
rs7060491	X	ARMCX4	HGNC Symbol	armadillo repeat containing, X-linked 4 [Source:HGNC Symbol;Acc:28615]
rs147107169	X;HG1439_PATCH	ARMCX4	HGNC Symbol	armadillo repeat containing, X-linked 4 [Source:HGNC Symbol;Acc:28615]
rs73564767				
rs142632461	X;HG1439_PATCH	RP3-514P16.1	Clone-based (Vega) gene	
rs6616255	X;HG1439_PATCH	ARMCX1	HGNC Symbol	armadillo repeat containing, X-linked 1 [Source:HGNC Symbol;Acc:18073]
rs1044275	X;HG1439_PATCH	ARMCX1	HGNC Symbol	armadillo repeat containing, X-linked 1 [Source:HGNC Symbol;Acc:18073]
rs149192215				
rs5951353				
rs62601876				
rs73566834				
rs62601880				
rs6995	X;HG1439_PATCH	ARMCX3	HGNC Symbol	armadillo repeat containing, X-linked 3 [Source:HGNC Symbol;Acc:24065]

SNP	Chromosome	gene name	gene source	description
rs2858168				
rs62601908				
rs3850315	X;HG1439_PATCH	ARMCX2	HGNC Symbol	armadillo repeat containing, X-linked 2 [Source:HGNC Symbol;Acc:16869]
rs16984406				
rs6621174				
rs12687779				
rs6621219	X	NXF5	HGNC Symbol	nuclear RNA export factor 5 [Source:HGNC Symbol;Acc:8075]
rs5986869				
rs17282855	X	ZMAT1	HGNC Symbol	zinc finger, matrin-type 1 [Source:HGNC Symbol;Acc:29377]
rs41305429	X	ZMAT1	HGNC Symbol	zinc finger, matrin-type 1 [Source:HGNC Symbol;Acc:29377]
rs5944883	X	ZMAT1	HGNC Symbol	zinc finger, matrin-type 1 [Source:HGNC Symbol;Acc:29377]
rs149021462	X	ZMAT1	HGNC Symbol	zinc finger, matrin-type 1 [Source:HGNC Symbol;Acc:29377]
rs148989446				
rs76058877				
rs151244117				
rs5944838				
rs59364010				
rs60325959				
rs5944857				
rs144385349				
rs4898340				
rs185988086	X	NXF2B;TCP11X2	HGNC Symbol	nuclear RNA export factor 2B [Source:HGNC Symbol;Acc:23984];t-complex 11 family, X-linked 2 [Source:HGNC Symbol;Acc:48335]
rs73254183				
rs6616394				
rs62600513				
rs5987614	X	NXF4	HGNC Symbol	nuclear RNA export factor 4 pseudogene [Source:HGNC Symbol;Acc:8074]
rs147068824	X	NXF4	HGNC Symbol	nuclear RNA export factor 4 pseudogene [Source:HGNC Symbol;Acc:8074]
rs5945891	X	NXF4	HGNC Symbol	nuclear RNA export factor 4 pseudogene [Source:HGNC Symbol;Acc:8074]
rs5945893	X	NXF4	HGNC Symbol	nuclear RNA export factor 4 pseudogene [Source:HGNC Symbol;Acc:8074]
rs35168882	X	ARMCX5;RP4-769N13.7;RP4-769N13.6	HGNC Symbol;Clone-based (Vega) gene	armadillo repeat containing, X-linked 5 [Source:HGNC Symbol;Acc:25772];

SNP	Chromosome	gene name	gene source	description
rs17340210	X	RP4-769N13.6	Clone-based (Vega) gene	
rs5987626	X	RP4-769N13.2;RP4-769N13.6	Clone-based (Vega) gene	
rs12391731	X	BHLHB9	HGNC Symbol	basic helix-loop-helix domain containing, class B, 9 [Source:HGNC Symbol;Acc:29353]
rs2179675				
rs17284970	X	LINC00630;MTND1P32	HGNC Symbol	long intergenic non-protein coding RNA 630 [Source:HGNC Symbol;Acc:44263];MT-ND1 pseudogene 32 [Source:HGNC Symbol;Acc:42081]
rs138047395	X	LINC00630	HGNC Symbol	long intergenic non-protein coding RNA 630 [Source:HGNC Symbol;Acc:44263]
rs960109	X	LL0XNC01-237H1.2	Clone-based (Vega) gene	
rs73256121				
rs725570				
rs138218657				
rs73256131				
rs5945924				
rs11092424				
rs73256137				
rs143849888				
rs6616440				
rs111398273	X	NXF3	HGNC Symbol	nuclear RNA export factor 3 [Source:HGNC Symbol;Acc:8073]
rs62591059				
rs17285025				
rs5987510				
rs978853				
rs6621621				
rs35902347				
rs41311733	X	BEX4	HGNC Symbol	brain expressed, X-linked 4 [Source:HGNC Symbol;Acc:25475]
rs6621632				
rs5987713				
rs61558955				
rs12838044				
rs12391568				
rs2743711				

SNP	Chromosome	gene name	gene source	description
rs11092437				
rs6616462				
rs150346899				
rs12839772				
rs4140428				
rs62591314				
rs1180891				
rs28529596				
rs12836455				
rs78248230				
rs150540333				
rs12012342				
rs11545818	X	TCEAL4	HGNC Symbol	transcription elongation factor A (SII)-like 4 [Source:HGNC Symbol;Acc:26121]
rs150700864				
rs4907840				
rs6616493				
rs874	X	MORF4L2	HGNC Symbol	mortality factor 4 like 2 [Source:HGNC Symbol;Acc:16849]
rs115898710	X	MORF4L2	HGNC Symbol	mortality factor 4 like 2 [Source:HGNC Symbol;Acc:16849]
rs1543384				
rs5945801				
rs5987572	X	GLRA4	HGNC Symbol	glycine receptor, alpha 4 [Source:HGNC Symbol;Acc:31715]
rs75607306	X	GLRA4	HGNC Symbol	glycine receptor, alpha 4 [Source:HGNC Symbol;Acc:31715]
rs5945807	X	GLRA4	HGNC Symbol	glycine receptor, alpha 4 [Source:HGNC Symbol;Acc:31715]
rs4907817	X	GLRA4	HGNC Symbol	glycine receptor, alpha 4 [Source:HGNC Symbol;Acc:31715]
rs5945699				
rs1029315				
rs483812				
rs139779446				
rs743901				
rs588849				
rs5987579				
rs11796658	X	PLP1	HGNC Symbol	proteolipid protein 1 [Source:HGNC Symbol;Acc:9086]
rs521895	X	PLP1	HGNC Symbol	proteolipid protein 1 [Source:HGNC Symbol;Acc:9086]

SNP	Chromosome	gene name	gene source	description
rs17003884	X	PLP1	HGNC Symbol	proteolipid protein 1 [Source:HGNC Symbol;Acc:9086]
rs1126707	X	PLP1	HGNC Symbol	proteolipid protein 1 [Source:HGNC Symbol;Acc:9086]
rs10521502				
rs111915380				
rs5945830				
rs1343157				
rs7067173				
rs178310				
rs62594675	X	TMSB15B	HGNC Symbol	thymosin beta 15B [Source:HGNC Symbol;Acc:28612]
rs4907851	X;HG1441_PATCH	TMSB15B;LL0XNC01-116E7.4	HGNC Symbol;Clone-based (Vega) gene	thymosin beta 15B [Source:HGNC Symbol;Acc:28612];
rs2385014	X	TMSB15B	HGNC Symbol	thymosin beta 15B [Source:HGNC Symbol;Acc:28612]
rs148270795	X	TMSB15B	HGNC Symbol	thymosin beta 15B [Source:HGNC Symbol;Acc:28612]
rs71203799				
rs507089				
rs553509	X;HG1441_PATCH	H2BFWT	HGNC Symbol	H2B histone family, member W, testis-specific [Source:HGNC Symbol;Acc:27252]
rs141626622				
rs34367407				
rs178116				
rs5916738	X;HG1441_PATCH	FAM199X	HGNC Symbol	family with sequence similarity 199, X-linked [Source:HGNC Symbol;Acc:25195]
rs17332064	X	FAM199X	HGNC Symbol	family with sequence similarity 199, X-linked [Source:HGNC Symbol;Acc:25195]
rs15464	X	FAM199X	HGNC Symbol	family with sequence similarity 199, X-linked [Source:HGNC Symbol;Acc:25195]
rs41305431	X	ESX1	HGNC Symbol	ESX homeobox 1 [Source:HGNC Symbol;Acc:14865]
rs66532214				
rs142462119				
rs5962705				
rs34071875				
rs17342782				
rs5917031				
rs17285326				
rs72616866				

SNP	Chromosome	gene name	gene source	description
rs12837467				
rs6616530				
rs5962428				
rs1172043				
rs112217849	X	IL1RAPL2	HGNC Symbol	interleukin 1 receptor accessory protein-like 2 [Source:HGNC Symbol;Acc:5997]
rs58548809	X	IL1RAPL2	HGNC Symbol	interleukin 1 receptor accessory protein-like 2 [Source:HGNC Symbol;Acc:5997]
rs6621854	X	IL1RAPL2	HGNC Symbol	interleukin 1 receptor accessory protein-like 2 [Source:HGNC Symbol;Acc:5997]
rs138552152	X	IL1RAPL2	HGNC Symbol	interleukin 1 receptor accessory protein-like 2 [Source:HGNC Symbol;Acc:5997]
rs73243896	X	IL1RAPL2	HGNC Symbol	interleukin 1 receptor accessory protein-like 2 [Source:HGNC Symbol;Acc:5997]
rs112962026	X	IL1RAPL2	HGNC Symbol	interleukin 1 receptor accessory protein-like 2 [Source:HGNC Symbol;Acc:5997]
rs73245703	X	IL1RAPL2	HGNC Symbol	interleukin 1 receptor accessory protein-like 2 [Source:HGNC Symbol;Acc:5997]
rs73245705	X	IL1RAPL2	HGNC Symbol	interleukin 1 receptor accessory protein-like 2 [Source:HGNC Symbol;Acc:5997]
rs144961385	X	IL1RAPL2	HGNC Symbol	interleukin 1 receptor accessory protein-like 2 [Source:HGNC Symbol;Acc:5997]
rs145914158	X	IL1RAPL2	HGNC Symbol	interleukin 1 receptor accessory protein-like 2 [Source:HGNC Symbol;Acc:5997]
rs141124309	X	IL1RAPL2	HGNC Symbol	interleukin 1 receptor accessory protein-like 2 [Source:HGNC Symbol;Acc:5997]
rs6621874	X	IL1RAPL2	HGNC Symbol	interleukin 1 receptor accessory protein-like 2 [Source:HGNC Symbol;Acc:5997]
rs73245719	X	IL1RAPL2	HGNC Symbol	interleukin 1 receptor accessory protein-like 2 [Source:HGNC Symbol;Acc:5997]
rs1343409	X	IL1RAPL2	HGNC Symbol	interleukin 1 receptor accessory protein-like 2 [Source:HGNC Symbol;Acc:5997]
rs5962999	X	IL1RAPL2	HGNC Symbol	interleukin 1 receptor accessory protein-like 2 [Source:HGNC Symbol;Acc:5997]
rs4515681	X	IL1RAPL2	HGNC Symbol	interleukin 1 receptor accessory protein-like 2 [Source:HGNC Symbol;Acc:5997]
rs6652393	X	IL1RAPL2	HGNC Symbol	interleukin 1 receptor accessory protein-like 2 [Source:HGNC Symbol;Acc:5997]
rs67067821	X	IL1RAPL2	HGNC Symbol	interleukin 1 receptor accessory protein-like 2 [Source:HGNC Symbol;Acc:5997]
rs2392623	X	IL1RAPL2	HGNC Symbol	interleukin 1 receptor accessory protein-like 2 [Source:HGNC Symbol;Acc:5997]
rs143214500	X	IL1RAPL2	HGNC Symbol	interleukin 1 receptor accessory protein-like 2 [Source:HGNC Symbol;Acc:5997]
rs5916724	X	IL1RAPL2	HGNC Symbol	interleukin 1 receptor accessory protein-like 2 [Source:HGNC Symbol;Acc:5997]
rs5916725	X	IL1RAPL2	HGNC Symbol	interleukin 1 receptor accessory protein-like 2 [Source:HGNC Symbol;Acc:5997]
rs142058815	X	IL1RAPL2	HGNC Symbol	interleukin 1 receptor accessory protein-like 2 [Source:HGNC Symbol;Acc:5997]
rs17332218	X	IL1RAPL2	HGNC Symbol	interleukin 1 receptor accessory protein-like 2 [Source:HGNC Symbol;Acc:5997]
rs73245758	X	IL1RAPL2	HGNC Symbol	interleukin 1 receptor accessory protein-like 2 [Source:HGNC Symbol;Acc:5997]
rs141365747	X;HG375_PATCH	IL1RAPL2	HGNC Symbol	interleukin 1 receptor accessory protein-like 2 [Source:HGNC Symbol;Acc:5997]
rs35882917	X;HG375_PATCH	IL1RAPL2	HGNC Symbol	interleukin 1 receptor accessory protein-like 2 [Source:HGNC Symbol;Acc:5997]

SNP	Chromosome	gene name	gene source	description
rs5916731	X;HG375_PATCH	IL1RAPL2	HGNC Symbol	interleukin 1 receptor accessory protein-like 2 [Source:HGNC Symbol;Acc:5997]
rs16984652	X;HG375_PATCH	IL1RAPL2	HGNC Symbol	interleukin 1 receptor accessory protein-like 2 [Source:HGNC Symbol;Acc:5997]
rs6616577	X;HG375_PATCH	IL1RAPL2	HGNC Symbol	interleukin 1 receptor accessory protein-like 2 [Source:HGNC Symbol;Acc:5997]
rs7063177	X	IL1RAPL2	HGNC Symbol	interleukin 1 receptor accessory protein-like 2 [Source:HGNC Symbol;Acc:5997]
rs17285500	X	IL1RAPL2	HGNC Symbol	interleukin 1 receptor accessory protein-like 2 [Source:HGNC Symbol;Acc:5997]
rs73245768	X	IL1RAPL2	HGNC Symbol	interleukin 1 receptor accessory protein-like 2 [Source:HGNC Symbol;Acc:5997]
rs1741710	X	IL1RAPL2	HGNC Symbol	interleukin 1 receptor accessory protein-like 2 [Source:HGNC Symbol;Acc:5997]
rs5916893	X	IL1RAPL2	HGNC Symbol	interleukin 1 receptor accessory protein-like 2 [Source:HGNC Symbol;Acc:5997]
rs150571119	X	IL1RAPL2	HGNC Symbol	interleukin 1 receptor accessory protein-like 2 [Source:HGNC Symbol;Acc:5997]
rs980736	X	IL1RAPL2	HGNC Symbol	interleukin 1 receptor accessory protein-like 2 [Source:HGNC Symbol;Acc:5997]
rs17332274	X	IL1RAPL2	HGNC Symbol	interleukin 1 receptor accessory protein-like 2 [Source:HGNC Symbol;Acc:5997]
rs17003895	X	IL1RAPL2	HGNC Symbol	interleukin 1 receptor accessory protein-like 2 [Source:HGNC Symbol;Acc:5997]
rs5916914	X	IL1RAPL2	HGNC Symbol	interleukin 1 receptor accessory protein-like 2 [Source:HGNC Symbol;Acc:5997]
rs72616902	X	IL1RAPL2	HGNC Symbol	interleukin 1 receptor accessory protein-like 2 [Source:HGNC Symbol;Acc:5997]
rs113217613	X	IL1RAPL2	HGNC Symbol	interleukin 1 receptor accessory protein-like 2 [Source:HGNC Symbol;Acc:5997]
rs73245784	X	IL1RAPL2	HGNC Symbol	interleukin 1 receptor accessory protein-like 2 [Source:HGNC Symbol;Acc:5997]
rs5962292	X	IL1RAPL2	HGNC Symbol	interleukin 1 receptor accessory protein-like 2 [Source:HGNC Symbol;Acc:5997]
rs5916932	X	IL1RAPL2	HGNC Symbol	interleukin 1 receptor accessory protein-like 2 [Source:HGNC Symbol;Acc:5997]
rs5962556	X	IL1RAPL2	HGNC Symbol	interleukin 1 receptor accessory protein-like 2 [Source:HGNC Symbol;Acc:5997]
rs150891050	X	IL1RAPL2	HGNC Symbol	interleukin 1 receptor accessory protein-like 2 [Source:HGNC Symbol;Acc:5997]
rs5916936	X	IL1RAPL2	HGNC Symbol	interleukin 1 receptor accessory protein-like 2 [Source:HGNC Symbol;Acc:5997]
rs5916941	X	IL1RAPL2	HGNC Symbol	interleukin 1 receptor accessory protein-like 2 [Source:HGNC Symbol;Acc:5997]
rs34434748	X	IL1RAPL2	HGNC Symbol	interleukin 1 receptor accessory protein-like 2 [Source:HGNC Symbol;Acc:5997]
rs141183767	X	IL1RAPL2	HGNC Symbol	interleukin 1 receptor accessory protein-like 2 [Source:HGNC Symbol;Acc:5997]
rs12557027	X	IL1RAPL2	HGNC Symbol	interleukin 1 receptor accessory protein-like 2 [Source:HGNC Symbol;Acc:5997]
rs59495607	X	IL1RAPL2	HGNC Symbol	interleukin 1 receptor accessory protein-like 2 [Source:HGNC Symbol;Acc:5997]
rs73245800	X	IL1RAPL2	HGNC Symbol	interleukin 1 receptor accessory protein-like 2 [Source:HGNC Symbol;Acc:5997]
rs3764765	X	IL1RAPL2	HGNC Symbol	interleukin 1 receptor accessory protein-like 2 [Source:HGNC Symbol;Acc:5997]
rs5962298	X	IL1RAPL2	HGNC Symbol	interleukin 1 receptor accessory protein-like 2 [Source:HGNC Symbol;Acc:5997]
rs17332335				
rs79313072	X	NRK	HGNC Symbol	Nik related kinase [Source:HGNC Symbol;Acc:25391]

SNP	Chromosome	gene name	gene source	description
rs73247907	X	NRK	HGNC Symbol	Nik related kinase [Source:HGNC Symbol;Acc:25391]
rs17285577	X	NRK	HGNC Symbol	Nik related kinase [Source:HGNC Symbol;Acc:25391]
rs17332342	X	NRK	HGNC Symbol	Nik related kinase [Source:HGNC Symbol;Acc:25391]
rs209373	X	NRK	HGNC Symbol	Nik related kinase [Source:HGNC Symbol;Acc:25391]
rs209095	X	NRK	HGNC Symbol	Nik related kinase [Source:HGNC Symbol;Acc:25391]
rs209109	X	NRK	HGNC Symbol	Nik related kinase [Source:HGNC Symbol;Acc:25391]
rs10521507				
rs209119				
rs11795816				
rs5916749				
rs1804495	X	SERPINA7	HGNC Symbol	serpin peptidase inhibitor, clade A (alpha-1 antiproteinase, antitrypsin), member 7 [Source:HGNC Symbol;Acc:11583]
rs148070011				
rs5916966				
rs5916970				
rs72618332				
rs12006621				
rs376289				
rs6622044				
rs199528480	X	MUM1L1	HGNC Symbol	melanoma associated antigen (mutated) 1-like 1 [Source:HGNC Symbol;Acc:26583]
rs17332376				
rs5962323				
rs379742				
rs5916757				
rs10521508				
rs143816237				
rs145343117				
rs62603149				
rs141805325				
rs5917007				
rs5917009				
rs73247947	X	CXorf57	HGNC Symbol	chromosome X open reading frame 57 [Source:HGNC Symbol;Acc:25486]
rs6622104	X	CXorf57	HGNC Symbol	chromosome X open reading frame 57 [Source:HGNC Symbol;Acc:25486]

SNP	Chromosome	gene name	gene source	description
rs62603156	X	CXorf57	HGNC Symbol	chromosome X open reading frame 57 [Source:HGNC Symbol;Acc:25486]
rs73247954	X	RNF128	HGNC Symbol	ring finger protein 128, E3 ubiquitin protein ligase [Source:HGNC Symbol;Acc:21153]
rs2880013	X	RNF128	HGNC Symbol	ring finger protein 128, E3 ubiquitin protein ligase [Source:HGNC Symbol;Acc:21153]
rs1937303	X	MORC4;TBC1D8B	HGNC Symbol	MORC family CW-type zinc finger 4 [Source:HGNC Symbol;Acc:23485];TBC1 domain family, member 8B (with GRAM domain) [Source:HGNC Symbol;Acc:24715]
rs34044365	X	MORC4;TBC1D8B	HGNC Symbol	MORC family CW-type zinc finger 4 [Source:HGNC Symbol;Acc:23485];TBC1 domain family, member 8B (with GRAM domain) [Source:HGNC Symbol;Acc:24715]
rs2880012	X	MORC4;TBC1D8B	HGNC Symbol	MORC family CW-type zinc finger 4 [Source:HGNC Symbol;Acc:23485];TBC1 domain family, member 8B (with GRAM domain) [Source:HGNC Symbol;Acc:24715]
rs6622123	X	MORC4	HGNC Symbol	MORC family CW-type zinc finger 4 [Source:HGNC Symbol;Acc:23485]
rs6622126	X	MORC4	HGNC Symbol	MORC family CW-type zinc finger 4 [Source:HGNC Symbol;Acc:23485]
rs17253753	X	MORC4	HGNC Symbol	MORC family CW-type zinc finger 4 [Source:HGNC Symbol;Acc:23485]
rs41304048	X	MORC4	HGNC Symbol	MORC family CW-type zinc finger 4 [Source:HGNC Symbol;Acc:23485]
rs17326228	X	MORC4	HGNC Symbol	MORC family CW-type zinc finger 4 [Source:HGNC Symbol;Acc:23485]
rs12688220				
rs35560232				
rs147303751				
rs1285715	X	RBM41	HGNC Symbol	RNA binding motif protein 41 [Source:HGNC Symbol;Acc:25617]
rs144283335	X	NUP62CL	HGNC Symbol	nucleoporin 62kDa C-terminal like [Source:HGNC Symbol;Acc:25960]
rs1298577	X	NUP62CL	HGNC Symbol	nucleoporin 62kDa C-terminal like [Source:HGNC Symbol;Acc:25960]
rs151170437	X	NUP62CL	HGNC Symbol	nucleoporin 62kDa C-terminal like [Source:HGNC Symbol;Acc:25960]
rs6622173	X	NUP62CL	HGNC Symbol	nucleoporin 62kDa C-terminal like [Source:HGNC Symbol;Acc:25960]
rs1294085	X	PIH1D3	HGNC Symbol	PIH1 domain containing 3 [Source:HGNC Symbol;Acc:28570]
rs12857658				
rs58958250				
rs142746817				
rs2051790				
rs17254061				
rs5962828				
rs12559122				
rs6622225				
rs149729335				
rs143917804				
rs17320032				

SNP	Chromosome	gene name	gene source	description
rs5917052				
rs5916784				
rs6622233				
rs35814730				
rs1012633				
rs73249850	X	FRMPD3	HGNC Symbol	FERM and PDZ domain containing 3 [Source:HGNC Symbol;Acc:29382]
rs41304066	X	FRMPD3	HGNC Symbol	FERM and PDZ domain containing 3 [Source:HGNC Symbol;Acc:29382]
rs112067194				
rs9887704				
rs11092598				
rs11798115				
rs5916791				
rs17254207	X	TSC22D3	HGNC Symbol	TSC22 domain family, member 3 [Source:HGNC Symbol;Acc:3051]
rs6523976	X	TSC22D3	HGNC Symbol	TSC22 domain family, member 3 [Source:HGNC Symbol;Acc:3051]
rs12850825	X	TSC22D3	HGNC Symbol	TSC22 domain family, member 3 [Source:HGNC Symbol;Acc:3051]
rs5917070	X	NCBP2L	HGNC Symbol	nuclear cap binding protein subunit 2-like [Source:HGNC Symbol;Acc:31795]
rs5962885	X	NCBP2L	HGNC Symbol	nuclear cap binding protein subunit 2-like [Source:HGNC Symbol;Acc:31795]
rs73249873				
rs5917071				
rs59053226				
rs12852580	X	MID2	HGNC Symbol	midline 2 [Source:HGNC Symbol;Acc:7096]
rs11092600	X	MID2	HGNC Symbol	midline 2 [Source:HGNC Symbol;Acc:7096]
rs17254228	X	MID2	HGNC Symbol	midline 2 [Source:HGNC Symbol;Acc:7096]
rs17320095	X	MID2	HGNC Symbol	midline 2 [Source:HGNC Symbol;Acc:7096]
rs5917075	X	MID2	HGNC Symbol	midline 2 [Source:HGNC Symbol;Acc:7096]
rs12849510	X	MID2;RP6-191P20.4	HGNC Symbol;Clone-based (Vega) gene	midline 2 [Source:HGNC Symbol;Acc:7096];
rs139478894				
rs5962423				
rs141633839				
rs41300872	X	TEX13B	HGNC Symbol	testis expressed 13B [Source:HGNC Symbol;Acc:11736]
rs73251754				
rs807227				

SNP	Chromosome	gene name	gene source	description
rs1581754				
rs73251770				
rs12860693	X	VSIG1	HGNC Symbol	V-set and immunoglobulin domain containing 1 [Source:HGNC Symbol;Acc:28675]
rs17254305	X	VSIG1	HGNC Symbol	V-set and immunoglobulin domain containing 1 [Source:HGNC Symbol;Acc:28675]
rs807178	X	PSMD10	HGNC Symbol	proteasome (prosome, macropain) 26S subunit, non-ATPase, 10 [Source:HGNC Symbol;Acc:9555]
rs2064238	X	ATG4A	HGNC Symbol	autophagy related 4A, cysteine peptidase [Source:HGNC Symbol;Acc:16489]
rs17254349	X	ATG4A	HGNC Symbol	autophagy related 4A, cysteine peptidase [Source:HGNC Symbol;Acc:16489]
rs7061076	X	ATG4A;COL4A6	HGNC Symbol	autophagy related 4A, cysteine peptidase [Source:HGNC Symbol;Acc:16489];collagen, type IV, alpha 6 [Source:HGNC Symbol;Acc:2208]
rs5973822	X	ATG4A;COL4A6	HGNC Symbol	autophagy related 4A, cysteine peptidase [Source:HGNC Symbol;Acc:16489];collagen, type IV, alpha 6 [Source:HGNC Symbol;Acc:2208]
rs5973850	X	COL4A6	HGNC Symbol	collagen, type IV, alpha 6 [Source:HGNC Symbol;Acc:2208]
rs1042071	X	COL4A6	HGNC Symbol	collagen, type IV, alpha 6 [Source:HGNC Symbol;Acc:2208]
rs73251796	X	COL4A6	HGNC Symbol	collagen, type IV, alpha 6 [Source:HGNC Symbol;Acc:2208]
rs7050251	X	COL4A6	HGNC Symbol	collagen, type IV, alpha 6 [Source:HGNC Symbol;Acc:2208]
rs34466065	X	COL4A6	HGNC Symbol	collagen, type IV, alpha 6 [Source:HGNC Symbol;Acc:2208]
rs2295912	X	COL4A6	HGNC Symbol	collagen, type IV, alpha 6 [Source:HGNC Symbol;Acc:2208]
rs5929105	X	COL4A6	HGNC Symbol	collagen, type IV, alpha 6 [Source:HGNC Symbol;Acc:2208]
rs75532576	X	COL4A6	HGNC Symbol	collagen, type IV, alpha 6 [Source:HGNC Symbol;Acc:2208]
rs1042065	X	COL4A6	HGNC Symbol	collagen, type IV, alpha 6 [Source:HGNC Symbol;Acc:2208]
rs62601414	X	COL4A6	HGNC Symbol	collagen, type IV, alpha 6 [Source:HGNC Symbol;Acc:2208]
rs1266744	X	COL4A6	HGNC Symbol	collagen, type IV, alpha 6 [Source:HGNC Symbol;Acc:2208]
rs1266748	X	COL4A6	HGNC Symbol	collagen, type IV, alpha 6 [Source:HGNC Symbol;Acc:2208]
rs73524811	X	COL4A6	HGNC Symbol	collagen, type IV, alpha 6 [Source:HGNC Symbol;Acc:2208]
rs112441293	X	COL4A6	HGNC Symbol	collagen, type IV, alpha 6 [Source:HGNC Symbol;Acc:2208]
rs5973828	X	COL4A6	HGNC Symbol	collagen, type IV, alpha 6 [Source:HGNC Symbol;Acc:2208]
rs62601457	X	COL4A6	HGNC Symbol	collagen, type IV, alpha 6 [Source:HGNC Symbol;Acc:2208]
rs112557865	X	COL4A6	HGNC Symbol	collagen, type IV, alpha 6 [Source:HGNC Symbol;Acc:2208]
rs5929126	X	COL4A5	HGNC Symbol	collagen, type IV, alpha 5 [Source:HGNC Symbol;Acc:2207]
rs17254489	X	COL4A5	HGNC Symbol	collagen, type IV, alpha 5 [Source:HGNC Symbol;Acc:2207]
rs12007702	X	COL4A5	HGNC Symbol	collagen, type IV, alpha 5 [Source:HGNC Symbol;Acc:2207]
rs73253687	X	COL4A5	HGNC Symbol	collagen, type IV, alpha 5 [Source:HGNC Symbol;Acc:2207]
rs2143442	X	COL4A5	HGNC Symbol	collagen, type IV, alpha 5 [Source:HGNC Symbol;Acc:2207]
rs28627520	X	COL4A5	HGNC Symbol	collagen, type IV, alpha 5 [Source:HGNC Symbol;Acc:2207]

SNP	Chromosome	gene name	gene source	description
rs34077552	X	COL4A5	HGNC Symbol	collagen, type IV, alpha 5 [Source:HGNC Symbol;Acc:2207]
rs2273051	X	COL4A5	HGNC Symbol	collagen, type IV, alpha 5 [Source:HGNC Symbol;Acc:2207]
rs149872004				
rs17280280	X	RP6-24A23.7	Clone-based (Vega) gene	
rs41307415	X	IRS4	HGNC Symbol	insulin receptor substrate 4 [Source:HGNC Symbol;Acc:6128]
rs1801162	X	IRS4;RP6-24A23.3	HGNC Symbol;Clone-based (Vega) gene	insulin receptor substrate 4 [Source:HGNC Symbol;Acc:6128];
rs2073115	X	IRS4;RP6-24A23.3	HGNC Symbol;Clone-based (Vega) gene	insulin receptor substrate 4 [Source:HGNC Symbol;Acc:6128];
rs17320415				
rs73255611				
rs28770540				
rs1455401				
rs73255702				
rs1455393				
rs4614172				
rs5985275				
rs5942930				
rs5942970				
rs5985510				
rs148749091				
rs7061716				
rs5942734				
rs73261736				
rs5985681				
rs68013729				
rs34476096				
rs5943261				
rs5942780				
rs6643211				
rs2348450				

SNP	Chromosome	gene name	gene source	description
rs4893424				
rs62604912				
rs10521523				
rs10494606				
rs504284				
rs617417				
rs12833418				
rs2473637				
rs59771578				
rs656150	X	GUCY2F	HGNC Symbol	guanylate cyclase 2F, retinal [Source:HGNC Symbol;Acc:4691]
rs556111	X	GUCY2F	HGNC Symbol	guanylate cyclase 2F, retinal [Source:HGNC Symbol;Acc:4691]
rs2480507	X	GUCY2F	HGNC Symbol	guanylate cyclase 2F, retinal [Source:HGNC Symbol;Acc:4691]
rs17254566	X	GUCY2F	HGNC Symbol	guanylate cyclase 2F, retinal [Source:HGNC Symbol;Acc:4691]
rs73248157	X	GUCY2F	HGNC Symbol	guanylate cyclase 2F, retinal [Source:HGNC Symbol;Acc:4691]
rs658454	X	GUCY2F	HGNC Symbol	guanylate cyclase 2F, retinal [Source:HGNC Symbol;Acc:4691]
rs73248159	X	GUCY2F	HGNC Symbol	guanylate cyclase 2F, retinal [Source:HGNC Symbol;Acc:4691]
rs12008095	X	GUCY2F	HGNC Symbol	guanylate cyclase 2F, retinal [Source:HGNC Symbol;Acc:4691]
rs145057416				
rs73248163				
rs12156749				
rs5943381	X	NXT2	HGNC Symbol	nuclear transport factor 2-like export factor 2 [Source:HGNC Symbol;Acc:18151]
rs5943382	X	NXT2	HGNC Symbol	nuclear transport factor 2-like export factor 2 [Source:HGNC Symbol;Acc:18151]
rs1321289				
rs73532282				
rs73248167				
rs41307405	X	ACSL4	HGNC Symbol	acyl-CoA synthetase long-chain family member 4 [Source:HGNC Symbol;Acc:3571]
rs5985760	X	ACSL4	HGNC Symbol	acyl-CoA synthetase long-chain family member 4 [Source:HGNC Symbol;Acc:3571]
rs35077907	X	ACSL4	HGNC Symbol	acyl-CoA synthetase long-chain family member 4 [Source:HGNC Symbol;Acc:3571]
rs5985403	X	ACSL4	HGNC Symbol	acyl-CoA synthetase long-chain family member 4 [Source:HGNC Symbol;Acc:3571]
rs4893537	X	ACSL4	HGNC Symbol	acyl-CoA synthetase long-chain family member 4 [Source:HGNC Symbol;Acc:3571]
rs139736475				
rs62595869				
rs5942837				

SNP	Chromosome	gene name	gene source	description
rs55663048				
rs17320513				
rs5943442				
rs55860654				
rs5943443				
rs5985413				
rs12848534				
rs4893428				
rs62595952				
rs2188746				
rs5942860				
rs10218397				
rs10218162				
rs35852062				
rs143162402				
rs7054614				
rs2475827				
rs1012731				
rs2208812				
rs140091224				
rs2499416				
rs150035258				
rs73250210				
rs17320694	X	TMEM164	HGNC Symbol	transmembrane protein 164 [Source:HGNC Symbol;Acc:26217]
rs4893446	X	TMEM164	HGNC Symbol	transmembrane protein 164 [Source:HGNC Symbol;Acc:26217]
rs5985267	X	TMEM164	HGNC Symbol	transmembrane protein 164 [Source:HGNC Symbol;Acc:26217]
rs5942880	X	TMEM164	HGNC Symbol	transmembrane protein 164 [Source:HGNC Symbol;Acc:26217]
rs5942620	X	TMEM164	HGNC Symbol	transmembrane protein 164 [Source:HGNC Symbol;Acc:26217]
rs73250285	X	TMEM164	HGNC Symbol	transmembrane protein 164 [Source:HGNC Symbol;Acc:26217]
rs57228476	X	TMEM164	HGNC Symbol	transmembrane protein 164 [Source:HGNC Symbol;Acc:26217]
rs5942629	X	TMEM164	HGNC Symbol	transmembrane protein 164 [Source:HGNC Symbol;Acc:26217]
rs141273936	X	TMEM164	HGNC Symbol	transmembrane protein 164 [Source:HGNC Symbol;Acc:26217]
rs5942902	X	TMEM164	HGNC Symbol	transmembrane protein 164 [Source:HGNC Symbol;Acc:26217]

SNP	Chromosome	gene name	gene source	description
rs2795995	X	TMEM164	HGNC Symbol	transmembrane protein 164 [Source:HGNC Symbol;Acc:26217]
rs5942630				
rs5942906	X	AMMECR1	HGNC Symbol	Alport syndrome, mental retardation, midface hypoplasia and elliptocytosis chromosomal region gene 1 [Source:HGNC Symbol;Acc:467]
rs62595768	X	AMMECR1	HGNC Symbol	Alport syndrome, mental retardation, midface hypoplasia and elliptocytosis chromosomal region gene 1 [Source:HGNC Symbol;Acc:467]
rs17254838	X	AMMECR1	HGNC Symbol	Alport syndrome, mental retardation, midface hypoplasia and elliptocytosis chromosomal region gene 1 [Source:HGNC Symbol;Acc:467]
rs5942909	X	AMMECR1	HGNC Symbol	Alport syndrome, mental retardation, midface hypoplasia and elliptocytosis chromosomal region gene 1 [Source:HGNC Symbol;Acc:467]
rs36106944	X	AMMECR1	HGNC Symbol	Alport syndrome, mental retardation, midface hypoplasia and elliptocytosis chromosomal region gene 1 [Source:HGNC Symbol;Acc:467]
rs73250295	X	AMMECR1	HGNC Symbol	Alport syndrome, mental retardation, midface hypoplasia and elliptocytosis chromosomal region gene 1 [Source:HGNC Symbol;Acc:467]
rs2294504	X	AMMECR1	HGNC Symbol	Alport syndrome, mental retardation, midface hypoplasia and elliptocytosis chromosomal region gene 1 [Source:HGNC Symbol;Acc:467]
rs41309508	X	AMMECR1	HGNC Symbol	Alport syndrome, mental retardation, midface hypoplasia and elliptocytosis chromosomal region gene 1 [Source:HGNC Symbol;Acc:467]
rs41482749	X	AMMECR1;RGAG1	HGNC Symbol	Alport syndrome, mental retardation, midface hypoplasia and elliptocytosis chromosomal region gene 1 [Source:HGNC Symbol;Acc:467];retrotransposon gag domain containing 1 [Source:HGNC Symbol;Acc:29245]
rs146915924	X	AMMECR1;RGAG1	HGNC Symbol	Alport syndrome, mental retardation, midface hypoplasia and elliptocytosis chromosomal region gene 1 [Source:HGNC Symbol;Acc:467];retrotransposon gag domain containing 1 [Source:HGNC Symbol;Acc:29245]
rs140663280	X	AMMECR1;RGAG1	HGNC Symbol	Alport syndrome, mental retardation, midface hypoplasia and elliptocytosis chromosomal region gene 1 [Source:HGNC Symbol;Acc:467];retrotransposon gag domain containing 1 [Source:HGNC Symbol;Acc:29245]
rs73251804	X	AMMECR1;RGAG1	HGNC Symbol	Alport syndrome, mental retardation, midface hypoplasia and elliptocytosis chromosomal region gene 1 [Source:HGNC Symbol;Acc:467];retrotransposon gag domain containing 1 [Source:HGNC Symbol;Acc:29245]
rs5942920	X	AMMECR1;RGAG1	HGNC Symbol	Alport syndrome, mental retardation, midface hypoplasia and elliptocytosis chromosomal region gene 1 [Source:HGNC Symbol;Acc:467];retrotransposon gag domain containing 1 [Source:HGNC Symbol;Acc:29245]
rs768312	X	AMMECR1;RGAG1	HGNC Symbol	Alport syndrome, mental retardation, midface hypoplasia and elliptocytosis chromosomal region gene 1 [Source:HGNC Symbol;Acc:467];retrotransposon gag domain containing 1 [Source:HGNC Symbol;Acc:29245]
rs10521528	X	RGAG1	HGNC Symbol	retrotransposon gag domain containing 1 [Source:HGNC Symbol;Acc:29245]
rs41304450	X	RGAG1	HGNC Symbol	retrotransposon gag domain containing 1 [Source:HGNC Symbol;Acc:29245]
rs2073787	X	RGAG1	HGNC Symbol	retrotransposon gag domain containing 1 [Source:HGNC Symbol;Acc:29245]

SNP	Chromosome	gene name	gene source	description
rs41306249	X	RGAG1	HGNC Symbol	retrotransposon gag domain containing 1 [Source:HGNC Symbol;Acc:29245]
rs2073785	X	RGAG1	HGNC Symbol	retrotransposon gag domain containing 1 [Source:HGNC Symbol;Acc:29245]
rs5942931	X	RGAG1	HGNC Symbol	retrotransposon gag domain containing 1 [Source:HGNC Symbol;Acc:29245]
rs5985472				
rs146735686				
rs5942641				
rs12842402				
rs12013156				
rs1573036				
rs73251825				
rs5942656				
rs2206972				
rs73260103	X	CHRDL1	HGNC Symbol	chordin-like 1 [Source:HGNC Symbol;Acc:29861]
rs5943053	X	CHRDL1	HGNC Symbol	chordin-like 1 [Source:HGNC Symbol;Acc:29861]
rs5943057	X	CHRDL1	HGNC Symbol	chordin-like 1 [Source:HGNC Symbol;Acc:29861]
rs113730370	X	CHRDL1	HGNC Symbol	chordin-like 1 [Source:HGNC Symbol;Acc:29861]
rs197023	X	CHRDL1	HGNC Symbol	chordin-like 1 [Source:HGNC Symbol;Acc:29861]
rs73260109	X	CHRDL1	HGNC Symbol	chordin-like 1 [Source:HGNC Symbol;Acc:29861]
rs73260110	X	CHRDL1	HGNC Symbol	chordin-like 1 [Source:HGNC Symbol;Acc:29861]
rs34538785	X	CHRDL1	HGNC Symbol	chordin-like 1 [Source:HGNC Symbol;Acc:29861]
rs138136448				
rs148902394				
rs73260118				
rs55676345				
rs5943072				
rs34792745				
rs5943087				
rs73264367	X	PAK3	HGNC Symbol	p21 protein (Cdc42/Rac)-activated kinase 3 [Source:HGNC Symbol;Acc:8592]
rs145510914	X	PAK3	HGNC Symbol	p21 protein (Cdc42/Rac)-activated kinase 3 [Source:HGNC Symbol;Acc:8592]
rs55889661	X	PAK3	HGNC Symbol	p21 protein (Cdc42/Rac)-activated kinase 3 [Source:HGNC Symbol;Acc:8592]
rs5942712	X	PAK3	HGNC Symbol	p21 protein (Cdc42/Rac)-activated kinase 3 [Source:HGNC Symbol;Acc:8592]
rs5943126	X	PAK3	HGNC Symbol	p21 protein (Cdc42/Rac)-activated kinase 3 [Source:HGNC Symbol;Acc:8592]
rs111904771	X	PAK3	HGNC Symbol	p21 protein (Cdc42/Rac)-activated kinase 3 [Source:HGNC Symbol;Acc:8592]

SNP	Chromosome	gene name	gene source	description
rs17327273	X	PAK3	HGNC Symbol	p21 protein (Cdc42/Rac)-activated kinase 3 [Source:HGNC Symbol;Acc:8592]
rs1159650	X	PAK3	HGNC Symbol	p21 protein (Cdc42/Rac)-activated kinase 3 [Source:HGNC Symbol;Acc:8592]
rs10521531	X	PAK3	HGNC Symbol	p21 protein (Cdc42/Rac)-activated kinase 3 [Source:HGNC Symbol;Acc:8592]
rs57204200	X	PAK3	HGNC Symbol	p21 protein (Cdc42/Rac)-activated kinase 3 [Source:HGNC Symbol;Acc:8592]
rs2208019	X	PAK3	HGNC Symbol	p21 protein (Cdc42/Rac)-activated kinase 3 [Source:HGNC Symbol;Acc:8592]
rs73545213	X	PAK3	HGNC Symbol	p21 protein (Cdc42/Rac)-activated kinase 3 [Source:HGNC Symbol;Acc:8592]
rs5985320	X	PAK3	HGNC Symbol	p21 protein (Cdc42/Rac)-activated kinase 3 [Source:HGNC Symbol;Acc:8592]
rs73264385				
rs6642884	X	CAPN6	HGNC Symbol	calpain 6 [Source:HGNC Symbol;Acc:1483]
rs17882616	X	CAPN6	HGNC Symbol	calpain 6 [Source:HGNC Symbol;Acc:1483]
rs17885163	X	CAPN6	HGNC Symbol	calpain 6 [Source:HGNC Symbol;Acc:1483]
rs17883801	X	CAPN6	HGNC Symbol	calpain 6 [Source:HGNC Symbol;Acc:1483]
rs7882753				
rs73264386				
rs41300894	X	DCX	HGNC Symbol	doublecortin [Source:HGNC Symbol;Acc:2714]
rs144933556	X	DCX	HGNC Symbol	doublecortin [Source:HGNC Symbol;Acc:2714]
rs5985330	X	DCX	HGNC Symbol	doublecortin [Source:HGNC Symbol;Acc:2714]
rs11152658	X	DCX	HGNC Symbol	doublecortin [Source:HGNC Symbol;Acc:2714]
rs34843608	X	DCX	HGNC Symbol	doublecortin [Source:HGNC Symbol;Acc:2714]
rs138274835	X	DCX	HGNC Symbol	doublecortin [Source:HGNC Symbol;Acc:2714]
rs112274912	X	DCX	HGNC Symbol	doublecortin [Source:HGNC Symbol;Acc:2714]
rs5943146	X	DCX	HGNC Symbol	doublecortin [Source:HGNC Symbol;Acc:2714]
rs7056011	X	DCX	HGNC Symbol	doublecortin [Source:HGNC Symbol;Acc:2714]
rs7052287				
rs6642906				
rs16986503				
rs62613862				
rs17307447				
rs73549358				
rs73264395				
rs112561539				
rs144268644	X	LINC00890	HGNC Symbol	long intergenic non-protein coding RNA 890 [Source:HGNC Symbol;Acc:48576]
rs73266306				

SNP	Chromosome	gene name	gene source	description
rs2495805				
rs5943189				
rs16986618	X	ALG13	HGNC Symbol	ALG13, UDP-N-acetylglucosaminyltransferase subunit [Source:HGNC Symbol;Acc:30881]
rs17307593	X	ALG13	HGNC Symbol	ALG13, UDP-N-acetylglucosaminyltransferase subunit [Source:HGNC Symbol;Acc:30881]
rs11795603	X	TRPC5	HGNC Symbol	transient receptor potential cation channel, subfamily C, member 5 [Source:HGNC Symbol;Acc:12337]
rs34984575	X	TRPC5	HGNC Symbol	transient receptor potential cation channel, subfamily C, member 5 [Source:HGNC Symbol;Acc:12337]
rs3027743	X	TRPC5	HGNC Symbol	transient receptor potential cation channel, subfamily C, member 5 [Source:HGNC Symbol;Acc:12337]
rs73266317	X	TRPC5	HGNC Symbol	transient receptor potential cation channel, subfamily C, member 5 [Source:HGNC Symbol;Acc:12337]
rs10521536	X	TRPC5;TRPC5OS	HGNC Symbol	transient receptor potential cation channel, subfamily C, member 5 [Source:HGNC Symbol;Acc:12337];TRPC5 opposite strand [Source:HGNC Symbol;Acc:40593]
rs116763278	X	TRPC5;TRPC5OS	HGNC Symbol	transient receptor potential cation channel, subfamily C, member 5 [Source:HGNC Symbol;Acc:12337];TRPC5 opposite strand [Source:HGNC Symbol;Acc:40593]
rs16986679	X	TRPC5;TRPC5OS	HGNC Symbol	transient receptor potential cation channel, subfamily C, member 5 [Source:HGNC Symbol;Acc:12337];TRPC5 opposite strand [Source:HGNC Symbol;Acc:40593]
rs1966366	X	TRPC5	HGNC Symbol	transient receptor potential cation channel, subfamily C, member 5 [Source:HGNC Symbol;Acc:12337]
rs917290	X	TRPC5	HGNC Symbol	transient receptor potential cation channel, subfamily C, member 5 [Source:HGNC Symbol;Acc:12337]
rs12556646	X	TRPC5	HGNC Symbol	transient receptor potential cation channel, subfamily C, member 5 [Source:HGNC Symbol;Acc:12337]
rs4893414	X	TRPC5	HGNC Symbol	transient receptor potential cation channel, subfamily C, member 5 [Source:HGNC Symbol;Acc:12337]
rs7050529	X	TRPC5	HGNC Symbol	transient receptor potential cation channel, subfamily C, member 5 [Source:HGNC Symbol;Acc:12337]
rs4893416	X	TRPC5	HGNC Symbol	transient receptor potential cation channel, subfamily C, member 5 [Source:HGNC Symbol;Acc:12337]
rs17222608	X	TRPC5	HGNC Symbol	transient receptor potential cation channel, subfamily C, member 5 [Source:HGNC Symbol;Acc:12337]
rs2238999	X	TRPC5	HGNC Symbol	transient receptor potential cation channel, subfamily C, member 5 [Source:HGNC Symbol;Acc:12337]
rs5943223	X	TRPC5	HGNC Symbol	transient receptor potential cation channel, subfamily C, member 5 [Source:HGNC Symbol;Acc:12337]
rs57856726	X	TRPC5	HGNC Symbol	transient receptor potential cation channel, subfamily C, member 5 [Source:HGNC Symbol;Acc:12337]

SNP	Chromosome	gene name	gene source	description
rs3944092	X	TRPC5	HGNC Symbol	transient receptor potential cation channel, subfamily C, member 5 [Source:HGNC Symbol;Acc:12337]
rs7876872	X	TRPC5	HGNC Symbol	transient receptor potential cation channel, subfamily C, member 5 [Source:HGNC Symbol;Acc:12337]
rs140629422	X	TRPC5	HGNC Symbol	transient receptor potential cation channel, subfamily C, member 5 [Source:HGNC Symbol;Acc:12337]
rs12844670	X	TRPC5	HGNC Symbol	transient receptor potential cation channel, subfamily C, member 5 [Source:HGNC Symbol;Acc:12337]
rs73266330	X	TRPC5	HGNC Symbol	transient receptor potential cation channel, subfamily C, member 5 [Source:HGNC Symbol;Acc:12337]
rs73548118	X	TRPC5	HGNC Symbol	transient receptor potential cation channel, subfamily C, member 5 [Source:HGNC Symbol;Acc:12337]
rs7056863	X	TRPC5	HGNC Symbol	transient receptor potential cation channel, subfamily C, member 5 [Source:HGNC Symbol;Acc:12337]
rs73266331	X	TRPC5	HGNC Symbol	transient receptor potential cation channel, subfamily C, member 5 [Source:HGNC Symbol;Acc:12337]
rs73548178	X	TRPC5	HGNC Symbol	transient receptor potential cation channel, subfamily C, member 5 [Source:HGNC Symbol;Acc:12337]
rs57917942	X	TRPC5	HGNC Symbol	transient receptor potential cation channel, subfamily C, member 5 [Source:HGNC Symbol;Acc:12337]
rs138415038	X	TRPC5	HGNC Symbol	transient receptor potential cation channel, subfamily C, member 5 [Source:HGNC Symbol;Acc:12337]
rs6642976	X	TRPC5	HGNC Symbol	transient receptor potential cation channel, subfamily C, member 5 [Source:HGNC Symbol;Acc:12337]
rs5942765	X	TRPC5	HGNC Symbol	transient receptor potential cation channel, subfamily C, member 5 [Source:HGNC Symbol;Acc:12337]
rs5985364	X	TRPC5	HGNC Symbol	transient receptor potential cation channel, subfamily C, member 5 [Source:HGNC Symbol;Acc:12337]
rs73266342	X	TRPC5	HGNC Symbol	transient receptor potential cation channel, subfamily C, member 5 [Source:HGNC Symbol;Acc:12337]
rs17307746	X	TRPC5	HGNC Symbol	transient receptor potential cation channel, subfamily C, member 5 [Source:HGNC Symbol;Acc:12337]
rs151297580	X	TRPC5	HGNC Symbol	transient receptor potential cation channel, subfamily C, member 5 [Source:HGNC Symbol;Acc:12337]
rs6642982	X	TRPC5	HGNC Symbol	transient receptor potential cation channel, subfamily C, member 5 [Source:HGNC Symbol;Acc:12337]
rs3027693	X	TRPC5	HGNC Symbol	transient receptor potential cation channel, subfamily C, member 5 [Source:HGNC Symbol;Acc:12337]
rs73550175				
rs5985678				

SNP	Chromosome	gene name	gene source	description
rs73219764				
rs5942768				
rs5943238				
rs5943248				
rs7885066				
rs35403336				
rs73219774				
rs17307753				
rs2744440				
rs2887277				
rs73219779				
rs2368547				
rs2223470				
rs2744443				
rs2887278				
rs137933515				
rs2744446				
rs12010464				
rs1122647				
rs73219801				
rs5982518				
rs12013647				
rs111617304				
rs5982530				
rs5982532				
rs5982533				
rs16986963				
rs73221822				
rs12396827				
rs72619742				
rs6568039				
rs7888862				
rs58406516				

SNP	Chromosome	gene name	gene source	description
rs7880948				
rs7472981				
rs6568050	X	ZCCHC16	HGNC Symbol	zinc finger, CCHC domain containing 16 [Source:HGNC Symbol;Acc:25214]
rs7474140	X	ZCCHC16	HGNC Symbol	zinc finger, CCHC domain containing 16 [Source:HGNC Symbol;Acc:25214]
rs7053563	X	ZCCHC16	HGNC Symbol	zinc finger, CCHC domain containing 16 [Source:HGNC Symbol;Acc:25214]
rs10127108				
rs12398702				
rs67057973				
rs13441011				
rs113638537				
rs11152721				
rs17307949				
rs67830770				
rs10521551				
rs62612495				
rs73549303				
rs7062817				
rs6655188				
rs5973902				
rs2027947				
rs1160001				
rs929170	X	LHFPL1	HGNC Symbol	lipoma HMGIC fusion partner-like 1 [Source:HGNC Symbol;Acc:6587]
rs4829491	X	LHFPL1	HGNC Symbol	lipoma HMGIC fusion partner-like 1 [Source:HGNC Symbol;Acc:6587]
rs17223001	X	LHFPL1	HGNC Symbol	lipoma HMGIC fusion partner-like 1 [Source:HGNC Symbol;Acc:6587]
rs7050419	X	LHFPL1	HGNC Symbol	lipoma HMGIC fusion partner-like 1 [Source:HGNC Symbol;Acc:6587]
rs74654552	X	LHFPL1	HGNC Symbol	lipoma HMGIC fusion partner-like 1 [Source:HGNC Symbol;Acc:6587]
rs113170941	X	LHFPL1	HGNC Symbol	lipoma HMGIC fusion partner-like 1 [Source:HGNC Symbol;Acc:6587]
rs12687789	X	LHFPL1	HGNC Symbol	lipoma HMGIC fusion partner-like 1 [Source:HGNC Symbol;Acc:6587]
rs7064462	X	LHFPL1	HGNC Symbol	lipoma HMGIC fusion partner-like 1 [Source:HGNC Symbol;Acc:6587]
rs73221878	X	LHFPL1	HGNC Symbol	lipoma HMGIC fusion partner-like 1 [Source:HGNC Symbol;Acc:6587]
rs5929220	X	LHFPL1	HGNC Symbol	lipoma HMGIC fusion partner-like 1 [Source:HGNC Symbol;Acc:6587]
rs73536931				
rs138049801				

SNP	Chromosome	gene name	gene source	description
rs17223043				
rs17223050				
rs682448				
rs5973961				
rs2286064	X	AMOT	HGNC Symbol	angiomotin [Source:HGNC Symbol;Acc:17810]
rs41307407	X	AMOT	HGNC Symbol	angiomotin [Source:HGNC Symbol;Acc:17810]
rs5974282	X	AMOT	HGNC Symbol	angiomotin [Source:HGNC Symbol;Acc:17810]
rs604591	X	AMOT	HGNC Symbol	angiomotin [Source:HGNC Symbol;Acc:17810]
rs138393566	X	AMOT	HGNC Symbol	angiomotin [Source:HGNC Symbol;Acc:17810]
rs73221885	X	AMOT	HGNC Symbol	angiomotin [Source:HGNC Symbol;Acc:17810]
rs1024514	X	AMOT	HGNC Symbol	angiomotin [Source:HGNC Symbol;Acc:17810]
rs687956	X	AMOT	HGNC Symbol	angiomotin [Source:HGNC Symbol;Acc:17810]
rs595613				
rs669085				
rs646398				
rs9988292				
rs4829460				
rs35585982				
rs150675937				
rs146306060				
rs12007560				
rs5929497				
rs11796036				
rs16987193				
rs5973989				
rs3116884				
rs17308201				
rs112064956				
rs5929251				
rs6643175				
rs3125955				
rs60201198				
rs6642581				

SNP	Chromosome	gene name	gene source	description
rs2887506				
rs2206156				
rs73213486				
rs6643214				
rs5929263				
rs5929582				
rs6642602				
rs113320049				
rs113961218				
rs2143078				
rs73215411				
rs73215412				
rs3953025				
rs2369731				
rs112930425				
rs2369737				
rs6642625				
rs5929328				
rs2023560				
rs5929340				
rs2207304	X;HG1434_PATCH	RP5-964N17.1	Clone-based (Vega) gene	
rs148059565	X;HG1434_PATCH	RP5-964N17.1	Clone-based (Vega) gene	
rs17308368	X;HG1434_PATCH	RP5-964N17.1	Clone-based (Vega) gene	
rs5929362	X;HG1434_PATCH	RP5-964N17.1	Clone-based (Vega) gene	
rs5929169	X;HG1434_PATCH	RP5-964N17.1	Clone-based (Vega) gene	
rs142274380	X;HG1434_PATCH	RP5-964N17.1	Clone-based (Vega) gene	
rs140254789	X;HG1434_PATCH	RP5-964N17.1	Clone-based (Vega) gene	
rs5973905	X;HG1434_PATCH	RP5-964N17.1	Clone-based (Vega) gene	

SNP	Chromosome	gene name	gene source	description
rs5974103	X;HG1434_PATCH	RP5-964N17.1	Clone-based (Vega) gene	
rs5929190	X;HG1434_PATCH	RP5-964N17.1	Clone-based (Vega) gene	
rs146872238	X;HG1434_PATCH	RP5-964N17.1	Clone-based (Vega) gene	
rs17223372	X;HG1434_PATCH	RP5-964N17.1	Clone-based (Vega) gene	
rs6642650	X;HG1434_PATCH	RP5-964N17.1	Clone-based (Vega) gene	
rs34870939	X;HG1434_PATCH	RP5-964N17.1	Clone-based (Vega) gene	
rs3007202	X;HG1434_PATCH	RP5-964N17.1	Clone-based (Vega) gene	
rs73215455	X;HG1434_PATCH	RP5-964N17.1	Clone-based (Vega) gene	
rs3007199	X;HG1434_PATCH	RP5-964N17.1	Clone-based (Vega) gene	
rs56222686	X;HG1434_PATCH	RP5-964N17.1	Clone-based (Vega) gene	
rs7892025	X;HG1434_PATCH	RP5-964N17.1	Clone-based (Vega) gene	
rs5929418	X;HG1434_PATCH	RP5-964N17.1	Clone-based (Vega) gene	
rs2188502	X;HG1434_PATCH	RP5-964N17.1	Clone-based (Vega) gene	
rs73636163	X;HG1434_PATCH	RP5-964N17.1	Clone-based (Vega) gene	
rs3007172	X;HG1434_PATCH	RP5-964N17.1	Clone-based (Vega) gene	
rs2905403	X;HG1434_PATCH	RP5-964N17.1	Clone-based (Vega) gene	
rs3007186	X;HG1434_PATCH	RP5-964N17.1	Clone-based (Vega) gene	
rs2905396	X;HG1434_PATCH	RP5-964N17.1	Clone-based (Vega) gene	
rs7888133	X;HG1434_PATCH	RP5-964N17.1	Clone-based (Vega) gene	
rs72619782	X;HG1434_PATCH	RP5-964N17.1	Clone-based (Vega) gene	

SNP	Chromosome	gene name	gene source	description
rs17223394	X;HG1434_PATCH	RP5-964N17.1	Clone-based (Vega) gene	
rs34379821				
rs56296825				
rs6568218				
rs6642665	X;HG1434_PATCH	RN7SL93P	HGNC Symbol	RNA, 7SL, cytoplasmic 93, pseudogene [Source:HGNC Symbol;Acc:46109]
rs72619784				
rs12842945				
rs7058109				
rs56149954				
rs17004084				
rs5974216				
rs61153238				
rs2369982				
rs5974224				
rs1468409				
rs72619793				
rs5929429				
rs73636193				
rs10521569				
rs5929430				
rs5929431				
rs62594128				
rs142502442				
rs7055199				
rs12852981				
rs7057106				
rs5974243				
rs7053578				
rs143555798				
rs5929434				
rs16987404				
rs12841340				

SNP	Chromosome	gene name	gene source	description
rs5929198				
rs5973957				
rs17270382				
rs4829453				
rs143901299				
rs9698788				
rs73219446				
rs11152814				
rs73221281				
rs6421114				
rs12689510				
rs149254743				
rs5945977				
rs73578436				
rs60467311				
rs5988062				
rs73222911				
rs5987798				
rs12687525				
rs73222915				
rs5988152				
rs5988159	HG1462_PATCH;X	CTD-2230M5.3	Clone-based (Vega) gene	
rs12845557				
rs139945738				
rs142784345				
rs6655322				
rs5946131				
rs12557575				
rs1914866				
rs7889622				
rs1474122				
rs1401414				

SNP	Chromosome	gene name	gene source	description
rs12560109				
rs62595506				
rs3813928				
rs3813929				
rs518147	X;HG1462_PATCH	HTR2C	HGNC Symbol	5-hydroxytryptamine (serotonin) receptor 2C, G protein-coupled [Source:HGNC Symbol;Acc:5295]
rs539748	X;HG1462_PATCH	HTR2C	HGNC Symbol	5-hydroxytryptamine (serotonin) receptor 2C, G protein-coupled [Source:HGNC Symbol;Acc:5295]
rs12846241	X;HG1462_PATCH	HTR2C	HGNC Symbol	5-hydroxytryptamine (serotonin) receptor 2C, G protein-coupled [Source:HGNC Symbol;Acc:5295]
rs73222943	X;HG1462_PATCH	HTR2C	HGNC Symbol	5-hydroxytryptamine (serotonin) receptor 2C, G protein-coupled [Source:HGNC Symbol;Acc:5295]
rs73222944	X;HG1462_PATCH	HTR2C	HGNC Symbol	5-hydroxytryptamine (serotonin) receptor 2C, G protein-coupled [Source:HGNC Symbol;Acc:5295]
rs4911805	X;HG1462_PATCH	HTR2C	HGNC Symbol	5-hydroxytryptamine (serotonin) receptor 2C, G protein-coupled [Source:HGNC Symbol;Acc:5295]
rs146699879	X;HG1462_PATCH	HTR2C	HGNC Symbol	5-hydroxytryptamine (serotonin) receptor 2C, G protein-coupled [Source:HGNC Symbol;Acc:5295]
rs12855702	X;HG1462_PATCH	HTR2C	HGNC Symbol	5-hydroxytryptamine (serotonin) receptor 2C, G protein-coupled [Source:HGNC Symbol;Acc:5295]
rs6318	X;HG1462_PATCH	HTR2C	HGNC Symbol	5-hydroxytryptamine (serotonin) receptor 2C, G protein-coupled [Source:HGNC Symbol;Acc:5295]
rs2497538	X;HG1462_PATCH	HTR2C	HGNC Symbol	5-hydroxytryptamine (serotonin) receptor 2C, G protein-coupled [Source:HGNC Symbol;Acc:5295]
rs4338289	X;HG1462_PATCH	HTR2C	HGNC Symbol	5-hydroxytryptamine (serotonin) receptor 2C, G protein-coupled [Source:HGNC Symbol;Acc:5295]
rs5946164	X;HG1462_PATCH	HTR2C	HGNC Symbol	5-hydroxytryptamine (serotonin) receptor 2C, G protein-coupled [Source:HGNC Symbol;Acc:5295]
rs6644090	X;HG1462_PATCH	HTR2C	HGNC Symbol	5-hydroxytryptamine (serotonin) receptor 2C, G protein-coupled [Source:HGNC Symbol;Acc:5295]
rs5946009	X;HG1462_PATCH	HTR2C	HGNC Symbol	5-hydroxytryptamine (serotonin) receptor 2C, G protein-coupled [Source:HGNC Symbol;Acc:5295]
rs10875535	X;HG1462_PATCH	HTR2C	HGNC Symbol	5-hydroxytryptamine (serotonin) receptor 2C, G protein-coupled [Source:HGNC Symbol;Acc:5295]
rs1414334	X;HG1462_PATCH	HTR2C	HGNC Symbol	5-hydroxytryptamine (serotonin) receptor 2C, G protein-coupled [Source:HGNC Symbol;Acc:5295]
rs1360851	X;HG1462_PATCH	HTR2C	HGNC Symbol	5-hydroxytryptamine (serotonin) receptor 2C, G protein-coupled [Source:HGNC Symbol;Acc:5295]
rs73222967				

SNP	Chromosome	gene name	gene source	description
rs73222974				
rs62594921				
rs1537755				
rs56159328				
rs638376				
rs41300273	X	IL13RA2	HGNC Symbol	interleukin 13 receptor, alpha 2 [Source:HGNC Symbol;Acc:5975]
rs5946039	X;HG1462_PATCH	IL13RA2	HGNC Symbol	interleukin 13 receptor, alpha 2 [Source:HGNC Symbol;Acc:5975]
rs4911886				
rs10482478				
rs72620919				
rs73222991				
rs11167454				
rs17095098				
rs142704646				
rs11541651	X;HG1462_PATCH	LRCH2	HGNC Symbol	leucine-rich repeats and calponin homology (CH) domain containing 2 [Source:HGNC Symbol;Acc:29292]
rs73222998	X;HG1462_PATCH	LRCH2	HGNC Symbol	leucine-rich repeats and calponin homology (CH) domain containing 2 [Source:HGNC Symbol;Acc:29292]
rs12844807	X;HG1462_PATCH	LRCH2	HGNC Symbol	leucine-rich repeats and calponin homology (CH) domain containing 2 [Source:HGNC Symbol;Acc:29292]
rs17326506	X;HG1462_PATCH	LRCH2	HGNC Symbol	leucine-rich repeats and calponin homology (CH) domain containing 2 [Source:HGNC Symbol;Acc:29292]
rs12216983	X	LRCH2	HGNC Symbol	leucine-rich repeats and calponin homology (CH) domain containing 2 [Source:HGNC Symbol;Acc:29292]
rs56337951	X	LRCH2	HGNC Symbol	leucine-rich repeats and calponin homology (CH) domain containing 2 [Source:HGNC Symbol;Acc:29292]
rs6644208	X	LRCH2	HGNC Symbol	leucine-rich repeats and calponin homology (CH) domain containing 2 [Source:HGNC Symbol;Acc:29292]
rs62601525	X;HG1462_PATCH	LRCH2;RBMXL3	HGNC Symbol	leucine-rich repeats and calponin homology (CH) domain containing 2 [Source:HGNC Symbol;Acc:29292];RNA binding motif protein, X-linked-like 3 [Source:HGNC Symbol;Acc:26859]
rs12009026	X;HG1462_PATCH	LRCH2;RBMXL3	HGNC Symbol	leucine-rich repeats and calponin homology (CH) domain containing 2 [Source:HGNC Symbol;Acc:29292];RNA binding motif protein, X-linked-like 3 [Source:HGNC Symbol;Acc:26859]
rs62601529	X;HG1462_PATCH	LRCH2;RBMXL3	HGNC Symbol	leucine-rich repeats and calponin homology (CH) domain containing 2 [Source:HGNC Symbol;Acc:29292];RNA binding motif protein, X-linked-like 3 [Source:HGNC Symbol;Acc:26859]
rs62601530	X;HG1462_PATCH	LRCH2;RBMXL3	HGNC Symbol	leucine-rich repeats and calponin homology (CH) domain containing 2 [Source:HGNC Symbol;Acc:29292];RNA binding motif protein, X-linked-like 3 [Source:HGNC Symbol;Acc:26859]

SNP	Chromosome	gene name	gene source	description
rs12857270	X;HG1462_PATCH	LRCH2;RBMXL3	HGNC Symbol	leucine-rich repeats and calponin homology (CH) domain containing 2 [Source:HGNC Symbol;Acc:29292];RNA binding motif protein, X-linked-like 3 [Source:HGNC Symbol;Acc:26859]
rs80194951	X;HG1462_PATCH	LRCH2;RBMXL3	HGNC Symbol	leucine-rich repeats and calponin homology (CH) domain containing 2 [Source:HGNC Symbol;Acc:29292];RNA binding motif protein, X-linked-like 3 [Source:HGNC Symbol;Acc:26859]
rs11795689	X;HG1462_PATCH	LRCH2;RBMXL3	HGNC Symbol	leucine-rich repeats and calponin homology (CH) domain containing 2 [Source:HGNC Symbol;Acc:29292];RNA binding motif protein, X-linked-like 3 [Source:HGNC Symbol;Acc:26859]
rs17326513	X;HG1462_PATCH	LRCH2	HGNC Symbol	leucine-rich repeats and calponin homology (CH) domain containing 2 [Source:HGNC Symbol;Acc:29292]
rs2064652				
rs6644218				
rs2232733	X;HG1462_PATCH	LUZP4	HGNC Symbol	leucine zipper protein 4 [Source:HGNC Symbol;Acc:24971]
rs6643956	X;HG1462_PATCH	LUZP4	HGNC Symbol	leucine zipper protein 4 [Source:HGNC Symbol;Acc:24971]
rs41304490	X;HG1462_PATCH	LUZP4	HGNC Symbol	leucine zipper protein 4 [Source:HGNC Symbol;Acc:24971]
rs140549729				
rs11796486				
rs6413658				
rs11167465				
rs56093212				
rs73580023				
rs112243830				
rs11798970				
rs73571739				
rs147303012				
rs4911821				
rs5987898				
rs73224821				
rs12353866				
rs2398579				
rs144171652				
rs112561107				
rs7887273				
rs139091357				

SNP	Chromosome	gene name	gene source	description
rs7880226				
rs2522148				
rs2522161				
rs5946078	X;HG1462_PATCH	RP1-93I3.1	Clone-based (Vega) gene	
rs5945976	X;HG1462_PATCH	RP1-93I3.1	Clone-based (Vega) gene	
rs2843594	X;HG1462_PATCH	RP1-93I3.1	Clone-based (Vega) gene	
rs28540355				
rs5987927	X;HG1462_PATCH	RP1-93I3.1	Clone-based (Vega) gene	
rs17326695	X;HG1462_PATCH	RP1-93I3.1	Clone-based (Vega) gene	
rs12556546	X;HG1462_PATCH	RP1-93I3.1	Clone-based (Vega) gene	
rs3813931	X;HG1462_PATCH	RP1-93I3.1	Clone-based (Vega) gene	
rs2522179	X;HG1462_PATCH	PLS3	HGNC Symbol	plastin 3 [Source:HGNC Symbol;Acc:9091]
rs17326716	X;HG1462_PATCH	PLS3	HGNC Symbol	plastin 3 [Source:HGNC Symbol;Acc:9091]
rs12847396	X;HG1462_PATCH	PLS3	HGNC Symbol	plastin 3 [Source:HGNC Symbol;Acc:9091]
rs5987938	X;HG1462_PATCH	PLS3	HGNC Symbol	plastin 3 [Source:HGNC Symbol;Acc:9091]
rs150739150	X;HG1462_PATCH	PLS3	HGNC Symbol	plastin 3 [Source:HGNC Symbol;Acc:9091]
rs5987946	X;HG1462_PATCH	PLS3	HGNC Symbol	plastin 3 [Source:HGNC Symbol;Acc:9091]
rs5987947	X;HG1462_PATCH	PLS3	HGNC Symbol	plastin 3 [Source:HGNC Symbol;Acc:9091]
rs5987956	X;HG1462_PATCH	PLS3	HGNC Symbol	plastin 3 [Source:HGNC Symbol;Acc:9091]
rs2843611	X;HG1462_PATCH	PLS3	HGNC Symbol	plastin 3 [Source:HGNC Symbol;Acc:9091]
rs2108099	X;HG1462_PATCH	PLS3	HGNC Symbol	plastin 3 [Source:HGNC Symbol;Acc:9091]
rs12396000				

SNP	Chromosome	gene name	gene source	description
rs5987981				
rs12012154				
rs5945981				
rs12836051				
rs1859672				
rs7057674				
rs6579481				
rs73224841				
rs73224844				
rs151144195				
rs5988003				
rs7065513				
rs138672942	X;HG1463_PATCH	RP1-241P17.1	Clone-based (Vega) gene	
rs5988009				
rs62603974				
rs9778352				
rs12012155				
rs138185049				
rs9724449				
rs11091036				
rs75140750				
rs61638747				
rs143003447	X;HG1463_PATCH	RP11-761E20.1	Clone-based (Vega) gene	
rs59871887	X;HG1463_PATCH	RP11-761E20.1	Clone-based (Vega) gene	
rs5991107	X;HG1463_PATCH	RP11-761E20.1	Clone-based (Vega) gene	
rs5991047	X;HG1463_PATCH	RP11-761E20.1	Clone-based (Vega) gene	
rs4417000				
rs149648251				
rs7065954				
rs61180261				

SNP	Chromosome	gene name	gene source	description
rs142491398				
rs7889653				
rs5991133				
rs67515883				
rs6608539				
rs12847037				
rs73224879				
rs148007873				
rs12007405				
rs73224882				
rs73224883				
rs5950534				
rs148759028				
rs149397942				
rs1914714				
rs12156686				
rs12156673				
rs5950474				
rs1403543	X;HG1463_PATCH	AGTR2	HGNC Symbol	angiotensin II receptor, type 2 [Source:HGNC Symbol;Acc:338]
rs5194	X;HG1463_PATCH	AGTR2	HGNC Symbol	angiotensin II receptor, type 2 [Source:HGNC Symbol;Acc:338]
rs17231478	X	AGTR2	HGNC Symbol	angiotensin II receptor, type 2 [Source:HGNC Symbol;Acc:338]
rs5905246				
rs12835631				
rs150223216				
rs5905360				
rs11798303				
rs5905222				
rs5952236				
rs2208263				
rs5905231				
rs111766629				
rs5905240				

SNP	Chromosome	gene name	gene source	description
rs144119236				
rs6608623				
rs5905162				
rs12836652				
rs113946157				
rs6520222				
rs73226835				
rs5952084				
rs5905270				
rs5952090				
rs5952091				
rs12843107				
rs7879792				
rs17308697				
rs5905283				
rs12720076	X;HG1463_PATCH	SLC6A14	HGNC Symbol;UniProtKB Gene Name	solute carrier family 6 (amino acid transporter), member 14 [Source:HGNC Symbol;Acc:11047];
rs5905176	X;HG1463_PATCH	SLC6A14	HGNC Symbol;UniProtKB Gene Name	solute carrier family 6 (amino acid transporter), member 14 [Source:HGNC Symbol;Acc:11047];
rs4824324	X;HG1463_PATCH	SLC6A14	HGNC Symbol;UniProtKB Gene Name	solute carrier family 6 (amino acid transporter), member 14 [Source:HGNC Symbol;Acc:11047];
rs5952101	X;HG1463_PATCH	SLC6A14	HGNC Symbol;UniProtKB Gene Name	solute carrier family 6 (amino acid transporter), member 14 [Source:HGNC Symbol;Acc:11047];
rs17308739	X;HG1463_PATCH	SLC6A14	HGNC Symbol;UniProtKB Gene Name	solute carrier family 6 (amino acid transporter), member 14 [Source:HGNC Symbol;Acc:11047];
rs10521578	X;HG1463_PATCH	SLC6A14	HGNC Symbol;UniProtKB Gene Name	solute carrier family 6 (amino acid transporter), member 14 [Source:HGNC Symbol;Acc:11047];
rs5905178	X;HG1463_PATCH	SLC6A14	HGNC Symbol;UniProtKB Gene Name	solute carrier family 6 (amino acid transporter), member 14 [Source:HGNC Symbol;Acc:11047];
rs5905289	X	SLC6A14	HGNC Symbol	solute carrier family 6 (amino acid transporter), member 14 [Source:HGNC Symbol;Acc:11047]

SNP	Chromosome	gene name	gene source	description
rs6608651				
rs12390279				
rs2001124				
rs5905184				
rs1116786				
rs143426418				
rs5905314				
rs6608682				
rs6608690				
rs5905349				
rs12398711				
rs73213288				
rs60685549				
rs78660760				
rs5912025				
rs55645743				
rs1876296	X;HG1490_PATCH	RP11-232D9.3	Clone-based (Vega) gene	
rs68080618				
rs12687590				
rs5910361				
rs73215321				
rs62603537				
rs151123577				
rs12862103				
rs4512571				
rs62606183				
rs34480403				
rs73215333				
rs145822297				
rs144606078				
rs113359878				
rs6646380				

SNP	Chromosome	gene name	gene source	description
rs62605259				
rs73215343				
rs112424139				
rs55685404				
rs5957120				
rs4431762				
rs144864189				
rs6603593				
rs73219385				
rs6646677				
rs4534289				
rs73219392				
rs12556201				
rs143295616				
rs73219396				
rs73219397				
rs4422935				
rs149191032				
rs72605256				
rs72605259				
rs56318080				
rs62607107				
rs12689840				
rs12057130				
rs2840754				
rs6646802				
rs2078802				
rs2012066				
rs73211214				
rs886604				
rs500845				
rs2840706				
rs111688205				

SNP	Chromosome	gene name	gene source	description
rs2526815				
rs2526818				
rs5958177				
rs7884616				
rs2335744				
rs113778386				
rs6646819				
rs5911671				
rs35251144				
rs5958328				
rs2190287				
rs17317262				
rs73217404				
rs6646884				
rs6646885				
rs6646887				
rs12009172				
rs11260501				
rs5911853				
rs6646934				
rs4825918				
rs73636904				
rs2040962				
rs7054252				
rs6645774				
rs4353017				
rs2335763				
rs5911925				
rs112814066				
rs6646985				
rs5958709				
rs5912022				
rs62597488				

SNP	Chromosome	gene name	gene source	description
rs73223720				
rs5912029				
rs62597491				
rs146226451				
rs2336654				
rs2878761				
rs6645355				
rs5909489				
rs7058127				
rs2192283				
rs12008294				
rs5910238				
rs11797990				
rs10521581				
rs5909504				
rs2336400				
rs12838274				
rs73215036				
rs73215038				
rs2041642				
rs113300909				
rs17317813				
rs6645954				
rs765697				
rs73215054				
rs6645963				
rs1890387				
rs983225				
rs5956802				
rs5955983				
rs67316363				
rs5909510				
rs73215063				

SNP	Chromosome	gene name	gene source	description
rs5956807				
rs62609195				
rs5910266	X	KLHL13	HGNC Symbol	kelch-like family member 13 [Source:HGNC Symbol;Acc:22931]
rs6645994	X	KLHL13	HGNC Symbol	kelch-like family member 13 [Source:HGNC Symbol;Acc:22931]
rs6645995	X	KLHL13	HGNC Symbol	kelch-like family member 13 [Source:HGNC Symbol;Acc:22931]
rs2430212	X	KLHL13	HGNC Symbol	kelch-like family member 13 [Source:HGNC Symbol;Acc:22931]
rs2430207	X	KLHL13	HGNC Symbol	kelch-like family member 13 [Source:HGNC Symbol;Acc:22931]
rs6646018	X	KLHL13	HGNC Symbol	kelch-like family member 13 [Source:HGNC Symbol;Acc:22931]
rs144780203	X	KLHL13	HGNC Symbol	kelch-like family member 13 [Source:HGNC Symbol;Acc:22931]
rs3886411	X	KLHL13	HGNC Symbol	kelch-like family member 13 [Source:HGNC Symbol;Acc:22931]
rs55968218	X	KLHL13	HGNC Symbol	kelch-like family member 13 [Source:HGNC Symbol;Acc:22931]
rs12556108	X	KLHL13	HGNC Symbol	kelch-like family member 13 [Source:HGNC Symbol;Acc:22931]
rs6646098				
rs1230805				
rs12011685				
rs1230807				
rs1230820				
rs73588814				
rs2802918				
rs2539941				
rs2682987				
rs12557784				
rs1937425				
rs1954617				
rs1218135				
rs1218131				
rs1218126				
rs1218125				
rs5956024				
rs6645462				
rs5909528				
rs1218117				
rs6603379				

SNP	Chromosome	gene name	gene source	description
rs1218095				
rs72607617				
rs59564164				
rs17243490				
rs62608298				
rs1218138				
rs73216861				
rs1781973				
rs6646160				
rs145415682				
rs1716785	X	WDR44	HGNC Symbol	WD repeat domain 44 [Source:HGNC Symbol;Acc:30512]
rs4292883	X	WDR44	HGNC Symbol	WD repeat domain 44 [Source:HGNC Symbol;Acc:30512]
rs17318100	X	WDR44	HGNC Symbol	WD repeat domain 44 [Source:HGNC Symbol;Acc:30512]
rs17271416	X	WDR44	HGNC Symbol	WD repeat domain 44 [Source:HGNC Symbol;Acc:30512]
rs4825378	X	WDR44	HGNC Symbol	WD repeat domain 44 [Source:HGNC Symbol;Acc:30512]
rs35261442				
rs17331635				
rs112983517				
rs1294799				
rs73216875				
rs1294787				
rs1294784				
rs142569781				
rs12558710	X	DOCK11	HGNC Symbol	dedicator of cytokinesis 11 [Source:HGNC Symbol;Acc:23483]
rs1294822	X	DOCK11	HGNC Symbol	dedicator of cytokinesis 11 [Source:HGNC Symbol;Acc:23483]
rs7052630	X	DOCK11	HGNC Symbol	dedicator of cytokinesis 11 [Source:HGNC Symbol;Acc:23483]
rs6646228	X	DOCK11	HGNC Symbol	dedicator of cytokinesis 11 [Source:HGNC Symbol;Acc:23483]
rs5909543	X	DOCK11	HGNC Symbol	dedicator of cytokinesis 11 [Source:HGNC Symbol;Acc:23483]
rs62609950	X	DOCK11	HGNC Symbol	dedicator of cytokinesis 11 [Source:HGNC Symbol;Acc:23483]
rs5910373	X	DOCK11	HGNC Symbol	dedicator of cytokinesis 11 [Source:HGNC Symbol;Acc:23483]
rs17326744	X	DOCK11	HGNC Symbol	dedicator of cytokinesis 11 [Source:HGNC Symbol;Acc:23483]
rs1294781	X	DOCK11	HGNC Symbol	dedicator of cytokinesis 11 [Source:HGNC Symbol;Acc:23483]
rs1294826	X	DOCK11	HGNC Symbol	dedicator of cytokinesis 11 [Source:HGNC Symbol;Acc:23483]

SNP	Chromosome	gene name	gene source	description
rs5910376	X	DOCK11	HGNC Symbol	dedicator of cytokinesis 11 [Source:HGNC Symbol;Acc:23483]
rs2286977	X	DOCK11	HGNC Symbol	dedicator of cytokinesis 11 [Source:HGNC Symbol;Acc:23483]
rs1294809	X	DOCK11	HGNC Symbol	dedicator of cytokinesis 11 [Source:HGNC Symbol;Acc:23483]
rs140942232	X	DOCK11	HGNC Symbol	dedicator of cytokinesis 11 [Source:HGNC Symbol;Acc:23483]
rs4825597	X	DOCK11	HGNC Symbol	dedicator of cytokinesis 11 [Source:HGNC Symbol;Acc:23483]
rs73228281	X	DOCK11	HGNC Symbol	dedicator of cytokinesis 11 [Source:HGNC Symbol;Acc:23483]
rs6645516	X	DOCK11	HGNC Symbol	dedicator of cytokinesis 11 [Source:HGNC Symbol;Acc:23483]
rs4825598	X	DOCK11	HGNC Symbol	dedicator of cytokinesis 11 [Source:HGNC Symbol;Acc:23483]
rs148179857	X	DOCK11	HGNC Symbol	dedicator of cytokinesis 11 [Source:HGNC Symbol;Acc:23483]
rs2379118	X	DOCK11	HGNC Symbol	dedicator of cytokinesis 11 [Source:HGNC Symbol;Acc:23483]
rs17260901				
rs73228287				
rs5957044				
rs4825600				
rs2254498				
rs2495620	X	IL13RA1	HGNC Symbol	interleukin 13 receptor, alpha 1 [Source:HGNC Symbol;Acc:5974]
rs5910413	X	IL13RA1	HGNC Symbol	interleukin 13 receptor, alpha 1 [Source:HGNC Symbol;Acc:5974]
rs2495623	X	IL13RA1	HGNC Symbol	interleukin 13 receptor, alpha 1 [Source:HGNC Symbol;Acc:5974]
rs62608691	X	IL13RA1;RP13-128O4.3	HGNC Symbol;Clone-based (Vega) gene	interleukin 13 receptor, alpha 1 [Source:HGNC Symbol;Acc:5974];
rs2489879				
rs2430200				
rs759147				
rs74699409				
rs5957063				
rs5910423				
rs261720	X	RP4-562J12.2	Clone-based (Vega) gene	
rs261692	X	RP4-562J12.2	Clone-based (Vega) gene	
rs17260928	X	RP4-562J12.2	Clone-based (Vega) gene	
rs17335839	X	RP4-562J12.2	Clone-based (Vega) gene	

SNP	Chromosome	gene name	gene source	description
rs448670				
rs5910439				
rs150718333				
rs140376824				
rs73213121				
rs5910461				
rs3813933	X	LONRF3	HGNC Symbol	LON peptidase N-terminal domain and ring finger 3 [Source:HGNC Symbol;Acc:21152]
rs58462205	X	LONRF3	HGNC Symbol	LON peptidase N-terminal domain and ring finger 3 [Source:HGNC Symbol;Acc:21152]
rs5909579	X	LONRF3	HGNC Symbol	LON peptidase N-terminal domain and ring finger 3 [Source:HGNC Symbol;Acc:21152]
rs62599018	X	LONRF3	HGNC Symbol	LON peptidase N-terminal domain and ring finger 3 [Source:HGNC Symbol;Acc:21152]
rs1455862	X	LONRF3	HGNC Symbol	LON peptidase N-terminal domain and ring finger 3 [Source:HGNC Symbol;Acc:21152]
rs5910481	X	LONRF3	HGNC Symbol	LON peptidase N-terminal domain and ring finger 3 [Source:HGNC Symbol;Acc:21152]
rs34187529	X	LONRF3	HGNC Symbol	LON peptidase N-terminal domain and ring finger 3 [Source:HGNC Symbol;Acc:21152]
rs17326920				
rs7472817				
rs58142779				
rs3747383	X	KIAA1210	HGNC Symbol	KIAA1210 [Source:HGNC Symbol;Acc:29218]
rs12558675	X	KIAA1210	HGNC Symbol	KIAA1210 [Source:HGNC Symbol;Acc:29218]
rs2305570	X	KIAA1210	HGNC Symbol	KIAA1210 [Source:HGNC Symbol;Acc:29218]
rs3813932	X	KIAA1210	HGNC Symbol	KIAA1210 [Source:HGNC Symbol;Acc:29218]
rs3761592	X	KIAA1210	HGNC Symbol	KIAA1210 [Source:HGNC Symbol;Acc:29218]
rs7876554	X	KIAA1210	HGNC Symbol	KIAA1210 [Source:HGNC Symbol;Acc:29218]
rs7877969	X	KIAA1210	HGNC Symbol	KIAA1210 [Source:HGNC Symbol;Acc:29218]
rs148521556	X	KIAA1210	HGNC Symbol	KIAA1210 [Source:HGNC Symbol;Acc:29218]
rs73592500	X	KIAA1210	HGNC Symbol	KIAA1210 [Source:HGNC Symbol;Acc:29218]
rs5957117	X	KIAA1210	HGNC Symbol	KIAA1210 [Source:HGNC Symbol;Acc:29218]
rs5909596	X	KIAA1210	HGNC Symbol	KIAA1210 [Source:HGNC Symbol;Acc:29218]
rs10218155				
rs6603484				
rs150422858				
rs1806510				
rs73213151				
rs5910544				

SNP	Chromosome	gene name	gene source	description
rs2428744				
rs2428754				
rs5909609				
rs41294876				
rs11546862	X	PGRMC1	HGNC Symbol	progesterone receptor membrane component 1 [Source:HGNC Symbol;Acc:16090]
rs12353619				
rs28461382	X	RP5-1139I1.1	Clone-based (Vega) gene	
rs12688801	X	RP5-1139I1.1	Clone-based (Vega) gene	
rs150069730				
rs67644421				
rs4825636				
rs17326976				
rs5956118	X	RP5-1139I1.2	Clone-based (Vega) gene	
rs12688715	X	RP5-1139I1.2	Clone-based (Vega) gene	
rs5957154	X	RP5-1139I1.2	Clone-based (Vega) gene	
rs5909617	X	RP5-1139I1.2	Clone-based (Vega) gene	
rs217930	X	RP5-1139I1.2	Clone-based (Vega) gene	
rs67943868	X	RP5-1139I1.2	Clone-based (Vega) gene	
rs139378972	X	RP5-1139I1.2	Clone-based (Vega) gene	
rs58866784				
rs73213183				
rs217977	X	SLC25A43	HGNC Symbol	solute carrier family 25, member 43 [Source:HGNC Symbol;Acc:30557]
rs3848869	X	SLC25A43	HGNC Symbol	solute carrier family 25, member 43 [Source:HGNC Symbol;Acc:30557]
rs57802345	X	SLC25A43	HGNC Symbol	solute carrier family 25, member 43 [Source:HGNC Symbol;Acc:30557]
rs151283069	X	SLC25A43	HGNC Symbol	solute carrier family 25, member 43 [Source:HGNC Symbol;Acc:30557]
rs16274	X	SLC25A43	HGNC Symbol	solute carrier family 25, member 43 [Source:HGNC Symbol;Acc:30557]
rs17261103	X	SLC25A43	HGNC Symbol	solute carrier family 25, member 43 [Source:HGNC Symbol;Acc:30557]

SNP	Chromosome	gene name	gene source	description
rs12842133	X	SLC25A43	HGNC Symbol	solute carrier family 25, member 43 [Source:HGNC Symbol;Acc:30557]
rs217994	X	SLC25A43	HGNC Symbol	solute carrier family 25, member 43 [Source:HGNC Symbol;Acc:30557]
rs11798653	X	SLC25A43	HGNC Symbol	solute carrier family 25, member 43 [Source:HGNC Symbol;Acc:30557]
rs5910578	X	SLC25A43	HGNC Symbol	solute carrier family 25, member 43 [Source:HGNC Symbol;Acc:30557]
rs62599429	X	SLC25A43	HGNC Symbol	solute carrier family 25, member 43 [Source:HGNC Symbol;Acc:30557]
rs446246	X	SLC25A43	HGNC Symbol	solute carrier family 25, member 43 [Source:HGNC Symbol;Acc:30557]
rs3810755	X	SLC25A43	HGNC Symbol	solute carrier family 25, member 43 [Source:HGNC Symbol;Acc:30557]
rs3747455	X	SLC25A43	HGNC Symbol	solute carrier family 25, member 43 [Source:HGNC Symbol;Acc:30557]
rs10907045	X	SLC25A43	HGNC Symbol	solute carrier family 25, member 43 [Source:HGNC Symbol;Acc:30557]
rs12390	X	SLC25A5	HGNC Symbol	solute carrier family 25 (mitochondrial carrier; adenine nucleotide translocator), member 5 [Source:HGNC Symbol;Acc:10991]
rs5910592				
rs35099031				
rs5909628				
rs4825643				
rs6655438				
rs12559132				
rs16995547				
rs45537740	X	CXorf56	HGNC Symbol	chromosome X open reading frame 56 [Source:HGNC Symbol;Acc:26239]
rs143248031	X	CXorf56	HGNC Symbol	chromosome X open reading frame 56 [Source:HGNC Symbol;Acc:26239]
rs5957186	X	CXorf56	HGNC Symbol	chromosome X open reading frame 56 [Source:HGNC Symbol;Acc:26239]
rs5956133				
rs73214921	X	UBE2A	HGNC Symbol	ubiquitin-conjugating enzyme E2A [Source:HGNC Symbol;Acc:12472]
rs7879933	X	UBE2A	HGNC Symbol	ubiquitin-conjugating enzyme E2A [Source:HGNC Symbol;Acc:12472]
rs73214923				
rs2018358				
rs16995570	X	NKRF	HGNC Symbol	NFKB repressing factor [Source:HGNC Symbol;Acc:19374]
rs802802				
rs305157				
rs3848877	X	SEPT6	HGNC Symbol	septin 6 [Source:HGNC Symbol;Acc:15848]
rs12839202	X	SEPT6	HGNC Symbol	septin 6 [Source:HGNC Symbol;Acc:15848]
rs41311358	X	SEPT6	HGNC Symbol	septin 6 [Source:HGNC Symbol;Acc:15848]
rs9780607	X	SEPT6	HGNC Symbol	septin 6 [Source:HGNC Symbol;Acc:15848]

SNP	Chromosome	gene name	gene source	description
rs11797680	X	SEPT6	HGNC Symbol	septin 6 [Source:HGNC Symbol;Acc:15848]
rs5909648	X	SEPT6	HGNC Symbol	septin 6 [Source:HGNC Symbol;Acc:15848]
rs6645586	X	SEPT6	HGNC Symbol	septin 6 [Source:HGNC Symbol;Acc:15848]
rs6603539	X	SEPT6	HGNC Symbol	septin 6 [Source:HGNC Symbol;Acc:15848]
rs148792291	X	SEPT6	HGNC Symbol	septin 6 [Source:HGNC Symbol;Acc:15848]
rs73637876	X	SEPT6	HGNC Symbol	septin 6 [Source:HGNC Symbol;Acc:15848]
rs3747450	X	SEPT6	HGNC Symbol	septin 6 [Source:HGNC Symbol;Acc:15848]
rs4991683	X	SEPT6	HGNC Symbol	septin 6 [Source:HGNC Symbol;Acc:15848]
rs72607660	X	SEPT6	HGNC Symbol	septin 6 [Source:HGNC Symbol;Acc:15848]
rs12832146	X	SEPT6	HGNC Symbol	septin 6 [Source:HGNC Symbol;Acc:15848]
rs28687759	X	SEPT6	HGNC Symbol	septin 6 [Source:HGNC Symbol;Acc:15848]
rs4825663	X	SEPT6	HGNC Symbol	septin 6 [Source:HGNC Symbol;Acc:15848]
rs3848879				
rs77806999				
rs7891792				
rs17327018				
rs6646466				
rs56275017				
rs17327032				
rs2528711				
rs34102597				
rs73608359				
rs7880087				
rs2859473				
rs60580595				
rs5909654				
rs2782213				
rs6655442				
rs34867331				
rs2782215				
rs2782218				
rs2528727				
rs5910653				

SNP	Chromosome	gene name	gene source	description
rs2782224	X	SOWAHD	HGNC Symbol	sosondowah ankyrin repeat domain family member D [Source:HGNC Symbol;Acc:32960]
rs2782225	X	SOWAHD	HGNC Symbol	sosondowah ankyrin repeat domain family member D [Source:HGNC Symbol;Acc:32960]
rs2782228				
rs2782248				
rs34130449				
rs17261236	X	UPF3B	HGNC Symbol	UPF3 regulator of nonsense transcripts homolog B (yeast) [Source:HGNC Symbol;Acc:20439]
rs144417011				
rs1858934				
rs142871346	X	RNF113A	HGNC Symbol	ring finger protein 113A [Source:HGNC Symbol;Acc:12974]
rs1800823	X	RNF113A;NDUFA1	HGNC Symbol	ring finger protein 113A [Source:HGNC Symbol;Acc:12974];NADH dehydrogenase (ubiquinone) 1 alpha subcomplex, 1, 7.5kDa [Source:HGNC Symbol;Acc:7683]
rs708463	X	RNF113A;NDUFA1	HGNC Symbol	ring finger protein 113A [Source:HGNC Symbol;Acc:12974];NADH dehydrogenase (ubiquinone) 1 alpha subcomplex, 1, 7.5kDa [Source:HGNC Symbol;Acc:7683]
rs1541341	X	NDUFA1	HGNC Symbol	NADH dehydrogenase (ubiquinone) 1 alpha subcomplex, 1, 7.5kDa [Source:HGNC Symbol;Acc:7683]
rs2285553	X	NDUFA1	HGNC Symbol	NADH dehydrogenase (ubiquinone) 1 alpha subcomplex, 1, 7.5kDa [Source:HGNC Symbol;Acc:7683]
rs2496185				
rs2428237	X	AKAP14	HGNC Symbol	A kinase (PRKA) anchor protein 14 [Source:HGNC Symbol;Acc:24061]
rs36100454	X	AKAP14	HGNC Symbol	A kinase (PRKA) anchor protein 14 [Source:HGNC Symbol;Acc:24061]
rs56128042	X	AKAP14	HGNC Symbol	A kinase (PRKA) anchor protein 14 [Source:HGNC Symbol;Acc:24061]
rs964680	X	AKAP14	HGNC Symbol	A kinase (PRKA) anchor protein 14 [Source:HGNC Symbol;Acc:24061]
rs7879094	X	NKAP	HGNC Symbol	NFKB activating protein [Source:HGNC Symbol;Acc:29873]
rs5909669				
rs1468422				
rs7877396	X;HG1442_PATCH	GS1-421I3.2	Clone-based (Vega) gene	
rs140385058				
rs11260221				
rs6645613				
rs5910746				
rs151313265				
rs4825696	X	NKAPP1;RP4-755D9.1	HGNC Symbol;Clone-based (Vega) gene	NFKB activating protein pseudogene 1 [Source:HGNC Symbol;Acc:26706];

SNP	Chromosome	gene name	gene source	description
rs7059689	X	NKAPP1;RP4-755D9.1	HGNC Symbol;Clone-based (Vega) gene	NFKB activating protein pseudogene 1 [Source:HGNC Symbol;Acc:26706];
rs6645622	X;HG1442_PATCH	NKAPP1;RP4-755D9.1	HGNC Symbol;Clone-based (Vega) gene	NFKB activating protein pseudogene 1 [Source:HGNC Symbol;Acc:26706];
rs5910768	X;HG1442_PATCH	NKAPP1;RP4-755D9.1	HGNC Symbol;Clone-based (Vega) gene	NFKB activating protein pseudogene 1 [Source:HGNC Symbol;Acc:26706];
rs5956194	X	NKAPP1	HGNC Symbol	NFKB activating protein pseudogene 1 [Source:HGNC Symbol;Acc:26706]
rs73617820	X	NKAPP1	HGNC Symbol	NFKB activating protein pseudogene 1 [Source:HGNC Symbol;Acc:26706]
rs1016299	X	NKAPP1	HGNC Symbol	NFKB activating protein pseudogene 1 [Source:HGNC Symbol;Acc:26706]
rs5957336	X	NKAPP1	HGNC Symbol	NFKB activating protein pseudogene 1 [Source:HGNC Symbol;Acc:26706]
rs5910801	X	NKAPP1	HGNC Symbol	NFKB activating protein pseudogene 1 [Source:HGNC Symbol;Acc:26706]
rs59843861	X	NKAPP1	HGNC Symbol	NFKB activating protein pseudogene 1 [Source:HGNC Symbol;Acc:26706]
rs146325556	X	NKAPP1	HGNC Symbol	NFKB activating protein pseudogene 1 [Source:HGNC Symbol;Acc:26706]
rs73219111	X	NKAPP1	HGNC Symbol	NFKB activating protein pseudogene 1 [Source:HGNC Symbol;Acc:26706]
rs138070693				
rs7882480	X;HG1442_PATCH	ZBTB33	HGNC Symbol	zinc finger and BTB domain containing 33 [Source:HGNC Symbol;Acc:16682]
rs146524049	X;HG1442_PATCH	ZBTB33	HGNC Symbol	zinc finger and BTB domain containing 33 [Source:HGNC Symbol;Acc:16682]
rs5909691	X;HG1442_PATCH	TMEM255A	HGNC Symbol	transmembrane protein 255A [Source:HGNC Symbol;Acc:26086]
rs73219115	X;HG1442_PATCH	TMEM255A	HGNC Symbol	transmembrane protein 255A [Source:HGNC Symbol;Acc:26086]
rs41300936	X;HG1442_PATCH	TMEM255A	HGNC Symbol	transmembrane protein 255A [Source:HGNC Symbol;Acc:26086]
rs5957348	X;HG1442_PATCH	TMEM255A	HGNC Symbol	transmembrane protein 255A [Source:HGNC Symbol;Acc:26086]
rs45453700	X;HG1442_PATCH	TMEM255A	HGNC Symbol	transmembrane protein 255A [Source:HGNC Symbol;Acc:26086]
rs144401691	X;HG1442_PATCH	TMEM255A	HGNC Symbol	transmembrane protein 255A [Source:HGNC Symbol;Acc:26086]
rs73219119	X;HG1442_PATCH	TMEM255A	HGNC Symbol	transmembrane protein 255A [Source:HGNC Symbol;Acc:26086]
rs5909698	X;HG1442_PATCH	TMEM255A	HGNC Symbol	transmembrane protein 255A [Source:HGNC Symbol;Acc:26086]

SNP	Chromosome	gene name	gene source	description
rs73219121				
rs11797873				
rs5909701				
rs146532799				
rs11797418				
rs34592278				
rs62614206				
rs6646667				
rs3810748				
rs35769930	X	ATP1B4	HGNC Symbol	ATPase, Na+/K+ transporting, beta 4 polypeptide [Source:HGNC Symbol;Acc:808]
rs3788941	X	ATP1B4	HGNC Symbol	ATPase, Na+/K+ transporting, beta 4 polypeptide [Source:HGNC Symbol;Acc:808]
rs12839997	X	ATP1B4	HGNC Symbol	ATPase, Na+/K+ transporting, beta 4 polypeptide [Source:HGNC Symbol;Acc:808]
rs2192257				
rs1476923				
rs5910867				
rs66917610				
rs5957369				
rs11379511				
rs113279313				
rs2748				
rs56158197	X	LAMP2	HGNC Symbol	lysosomal-associated membrane protein 2 [Source:HGNC Symbol;Acc:6501]
rs41300191	X	LAMP2	HGNC Symbol	lysosomal-associated membrane protein 2 [Source:HGNC Symbol;Acc:6501]
rs42886	X	LAMP2	HGNC Symbol	lysosomal-associated membrane protein 2 [Source:HGNC Symbol;Acc:6501]
rs5910887	X	LAMP2	HGNC Symbol	lysosomal-associated membrane protein 2 [Source:HGNC Symbol;Acc:6501]
rs73219144	X	LAMP2	HGNC Symbol	lysosomal-associated membrane protein 2 [Source:HGNC Symbol;Acc:6501]
rs42895	X	LAMP2	HGNC Symbol	lysosomal-associated membrane protein 2 [Source:HGNC Symbol;Acc:6501]
rs12097	X	LAMP2	HGNC Symbol	lysosomal-associated membrane protein 2 [Source:HGNC Symbol;Acc:6501]
rs42897	X	LAMP2	HGNC Symbol	lysosomal-associated membrane protein 2 [Source:HGNC Symbol;Acc:6501]
rs11797604	X	LAMP2	HGNC Symbol	lysosomal-associated membrane protein 2 [Source:HGNC Symbol;Acc:6501]
rs42903				
rs62616185				
rs5909724				
rs12688345	X	CUL4B	HGNC Symbol	cullin 4B [Source:HGNC Symbol;Acc:2555]

SNP	Chromosome	gene name	gene source	description
rs56241821	X	CUL4B	HGNC Symbol	cullin 4B [Source:HGNC Symbol;Acc:2555]
rs2285550	X	CUL4B	HGNC Symbol	cullin 4B [Source:HGNC Symbol;Acc:2555]
rs62616188	X	CUL4B	HGNC Symbol	cullin 4B [Source:HGNC Symbol;Acc:2555]
rs12839503	X	CUL4B	HGNC Symbol	cullin 4B [Source:HGNC Symbol;Acc:2555]
rs112414111	X	CUL4B	HGNC Symbol	cullin 4B [Source:HGNC Symbol;Acc:2555]
rs45524842	X	CUL4B	HGNC Symbol	cullin 4B [Source:HGNC Symbol;Acc:2555]
rs17261565				
rs59200282				
rs146492852				
rs142004998	X	C1GALT1C1	HGNC Symbol	C1GALT1-specific chaperone 1 [Source:HGNC Symbol;Acc:24338]
rs45557031	X	C1GALT1C1	HGNC Symbol	C1GALT1-specific chaperone 1 [Source:HGNC Symbol;Acc:24338]
rs62613282	X	C1GALT1C1	HGNC Symbol	C1GALT1-specific chaperone 1 [Source:HGNC Symbol;Acc:24338]
rs73219171				
rs6603644				
rs139742301				
rs2196262				
rs59327191				
rs4825426				
rs5910981				
rs73639322				
rs2117501				
rs1972809				
rs2196260				
rs61129998				
rs5956244				
rs6646733				
rs6646738				
rs5909764				
rs56178909				
rs16995989				
rs5911010				
rs7057602				
rs5956251				

SNP	Chromosome	gene name	gene source	description
rs5957485				
rs138897258				
rs1978413				
rs6422487				
rs12559481				
rs12841685				
rs10521692				
rs73221009				
rs7064323				
rs4478705				
rs17282869				
rs5911050				
rs142422382				
rs5909778				
rs5911060				
rs5909779				
rs9697983	X	GLUD2	HGNC Symbol	glutamate dehydrogenase 2 [Source:HGNC Symbol;Acc:4336]
rs9698227				
rs17324016				
rs7051454				
rs60587586				
rs73221033				
rs189740973				
rs17258154				
rs13440470				
rs55725409				
rs5909792				
rs141841969				
rs2214044				
rs5909799				
rs6648784				
rs12842226				
rs73221068				

SNP	Chromosome	gene name	gene source	description
rs144911663				
rs5909807				
rs5957583				
rs73221074				
rs4325031				
rs5911113				
rs4142568				
rs12839589				
rs4825758				
rs35337637				
rs5957646				
rs145993778				
rs5957653				
rs73221092				
rs6649232				
rs7881649				
rs112132659				
rs11394866				
rs799002				
rs73223111				
rs2140257				
rs6649366				
rs633959				
rs73623462				
rs5957763				
rs2110461				
rs149535880				
rs7889784				
rs138669752				
rs147100961				
rs55848603				
rs147770850				
rs12007413				

SNP	Chromosome	gene name	gene source	description
rs73630968				
rs5956354				
rs2429659				
rs111323526				
rs17258545				
rs1931662				
rs17324456				
rs2483884				
rs2483880				
rs5957831				
rs5911183				
rs73633184				
rs4825778				
rs62601244				
rs2185658				
rs5909848				
rs2794543				
rs5957861				
rs5911247				
rs12849106				
rs151214516				
rs5909870				
rs5956385				
rs151048332				
rs1386920				
rs73227209				
rs16996558				
rs2801231				
rs73227224				
rs61170050				
rs10047006				
rs75093386				
rs73227232				

SNP	Chromosome	gene name	gene source	description
rs1166804				
rs73227237				
rs113170519				
rs148318031				
rs12011731				
rs5956424				
rs34235951				
rs2622946				
rs4825789				
rs1600714				
rs5957981				
rs10521708				
rs1481170				
rs2448888				
rs1481169				
rs5956458				
rs2495661				
rs5956460				
rs2495662				
rs55709132				
rs73227252				
rs2622953				
rs2840638				
rs11260370				
rs2199957				
rs2840627				
rs6648770				
rs6648406				
rs73227258				
rs5958027				
rs2840660				
rs2840658				
rs6648411				

SNP	Chromosome	gene name	gene source	description
rs147198307				
rs4825798				
rs72609453				
rs113165252				
rs73227261				
rs73227262				
rs2603608				
rs10126345				
rs113261051				
rs12559201				
rs146012502				
rs139781087				
rs73227269				
rs111403785				
rs1454663				
rs1378093				
rs5909897				
rs34911253				
rs1378083				
rs111443644				
rs4574971				
rs5909899				
rs4523407				
rs62593857				
rs151197960				
rs35464043				
rs5909908				
rs113252806				
rs148015407				
rs28545591				
rs6648836				
rs5958090				
rs73227286				

SNP	Chromosome	gene name	gene source	description
rs4509468				
rs5911423				
rs6655749				
rs6648877				
rs4372129				
rs4559365				
rs72609475				
rs73227290				
rs73227291				
rs4553047				
rs7052129				
rs5958129				
rs57360260				
rs1361627				
rs17324939				
rs5956496				
rs5956497				
rs6648473				
rs3013024				
rs4560923				
rs6648917				
rs7065151				
rs766843				
rs1293546				
rs5911475				
rs12690234				
rs1151720				
rs5909942				
rs1496800				
rs73229242				
rs1293473				
rs17259204				
rs76726056				

SNP	Chromosome	gene name	gene source	description
rs146925938				
rs5911528				
rs4825832				
rs72609487				
rs4145466				
rs11798709				
rs5958194				
rs6648978				
rs12389178				
rs3761555				
rs3761554				
rs12007747	X	GRIA3	HGNC Symbol	glutamate receptor, ionotropic, AMPA 3 [Source:HGNC Symbol;Acc:4573]
rs5909973	X	GRIA3	HGNC Symbol	glutamate receptor, ionotropic, AMPA 3 [Source:HGNC Symbol;Acc:4573]
rs4825836	X	GRIA3	HGNC Symbol	glutamate receptor, ionotropic, AMPA 3 [Source:HGNC Symbol;Acc:4573]
rs56385541	X	GRIA3	HGNC Symbol	glutamate receptor, ionotropic, AMPA 3 [Source:HGNC Symbol;Acc:4573]
rs17259295	X	GRIA3	HGNC Symbol	glutamate receptor, ionotropic, AMPA 3 [Source:HGNC Symbol;Acc:4573]
rs5911547	X	GRIA3	HGNC Symbol	glutamate receptor, ionotropic, AMPA 3 [Source:HGNC Symbol;Acc:4573]
rs5909974	X	GRIA3	HGNC Symbol	glutamate receptor, ionotropic, AMPA 3 [Source:HGNC Symbol;Acc:4573]
rs762993	X	GRIA3	HGNC Symbol	glutamate receptor, ionotropic, AMPA 3 [Source:HGNC Symbol;Acc:4573]
rs6655763	X	GRIA3	HGNC Symbol	glutamate receptor, ionotropic, AMPA 3 [Source:HGNC Symbol;Acc:4573]
rs4825841	X	GRIA3	HGNC Symbol	glutamate receptor, ionotropic, AMPA 3 [Source:HGNC Symbol;Acc:4573]
rs2157292	X	GRIA3	HGNC Symbol	glutamate receptor, ionotropic, AMPA 3 [Source:HGNC Symbol;Acc:4573]
rs5911552	X	GRIA3	HGNC Symbol	glutamate receptor, ionotropic, AMPA 3 [Source:HGNC Symbol;Acc:4573]
rs10521715	X	GRIA3	HGNC Symbol	glutamate receptor, ionotropic, AMPA 3 [Source:HGNC Symbol;Acc:4573]
rs142397190	X	GRIA3	HGNC Symbol	glutamate receptor, ionotropic, AMPA 3 [Source:HGNC Symbol;Acc:4573]
rs5958198	X	GRIA3	HGNC Symbol	glutamate receptor, ionotropic, AMPA 3 [Source:HGNC Symbol;Acc:4573]
rs5911554	X	GRIA3	HGNC Symbol	glutamate receptor, ionotropic, AMPA 3 [Source:HGNC Symbol;Acc:4573]
rs5911556	X	GRIA3	HGNC Symbol	glutamate receptor, ionotropic, AMPA 3 [Source:HGNC Symbol;Acc:4573]
rs139421110	X	GRIA3	HGNC Symbol	glutamate receptor, ionotropic, AMPA 3 [Source:HGNC Symbol;Acc:4573]
rs4825473	X	GRIA3	HGNC Symbol	glutamate receptor, ionotropic, AMPA 3 [Source:HGNC Symbol;Acc:4573]
rs4532718	X	GRIA3	HGNC Symbol	glutamate receptor, ionotropic, AMPA 3 [Source:HGNC Symbol;Acc:4573]
rs55755823	X	GRIA3	HGNC Symbol	glutamate receptor, ionotropic, AMPA 3 [Source:HGNC Symbol;Acc:4573]
rs5956535	X	GRIA3	HGNC Symbol	glutamate receptor, ionotropic, AMPA 3 [Source:HGNC Symbol;Acc:4573]

SNP	Chromosome	gene name	gene source	description
rs149925251	X	GRIA3	HGNC Symbol	glutamate receptor, ionotropic, AMPA 3 [Source:HGNC Symbol;Acc:4573]
rs6608076	X	GRIA3	HGNC Symbol	glutamate receptor, ionotropic, AMPA 3 [Source:HGNC Symbol;Acc:4573]
rs4825476	X	GRIA3	HGNC Symbol	glutamate receptor, ionotropic, AMPA 3 [Source:HGNC Symbol;Acc:4573]
rs5911586	X	GRIA3	HGNC Symbol	glutamate receptor, ionotropic, AMPA 3 [Source:HGNC Symbol;Acc:4573]
rs77757444	X	GRIA3	HGNC Symbol	glutamate receptor, ionotropic, AMPA 3 [Source:HGNC Symbol;Acc:4573]
rs151163057	X	GRIA3	HGNC Symbol	glutamate receptor, ionotropic, AMPA 3 [Source:HGNC Symbol;Acc:4573]
rs73229283	X	GRIA3	HGNC Symbol	glutamate receptor, ionotropic, AMPA 3 [Source:HGNC Symbol;Acc:4573]
rs62594108	X	GRIA3	HGNC Symbol	glutamate receptor, ionotropic, AMPA 3 [Source:HGNC Symbol;Acc:4573]
rs73229284	X	GRIA3	HGNC Symbol	glutamate receptor, ionotropic, AMPA 3 [Source:HGNC Symbol;Acc:4573]
rs4825856	X	GRIA3	HGNC Symbol	glutamate receptor, ionotropic, AMPA 3 [Source:HGNC Symbol;Acc:4573]
rs17325282	X	GRIA3	HGNC Symbol	glutamate receptor, ionotropic, AMPA 3 [Source:HGNC Symbol;Acc:4573]
rs7052053	X	GRIA3	HGNC Symbol	glutamate receptor, ionotropic, AMPA 3 [Source:HGNC Symbol;Acc:4573]
rs5910001	X	GRIA3	HGNC Symbol	glutamate receptor, ionotropic, AMPA 3 [Source:HGNC Symbol;Acc:4573]
rs11260429	X	GRIA3	HGNC Symbol	glutamate receptor, ionotropic, AMPA 3 [Source:HGNC Symbol;Acc:4573]
rs5910002	X	GRIA3	HGNC Symbol	glutamate receptor, ionotropic, AMPA 3 [Source:HGNC Symbol;Acc:4573]
rs2511034	X	GRIA3	HGNC Symbol	glutamate receptor, ionotropic, AMPA 3 [Source:HGNC Symbol;Acc:4573]
rs73229292	X	GRIA3	HGNC Symbol	glutamate receptor, ionotropic, AMPA 3 [Source:HGNC Symbol;Acc:4573]
rs612595	X	GRIA3	HGNC Symbol	glutamate receptor, ionotropic, AMPA 3 [Source:HGNC Symbol;Acc:4573]
rs502434	X	GRIA3	HGNC Symbol	glutamate receptor, ionotropic, AMPA 3 [Source:HGNC Symbol;Acc:4573]
rs526716	X	GRIA3	HGNC Symbol	glutamate receptor, ionotropic, AMPA 3 [Source:HGNC Symbol;Acc:4573]
rs524654	X	GRIA3	HGNC Symbol	glutamate receptor, ionotropic, AMPA 3 [Source:HGNC Symbol;Acc:4573]
rs551166	X	GRIA3	HGNC Symbol	glutamate receptor, ionotropic, AMPA 3 [Source:HGNC Symbol;Acc:4573]
rs611524	X	GRIA3	HGNC Symbol	glutamate receptor, ionotropic, AMPA 3 [Source:HGNC Symbol;Acc:4573]
rs10521721	X	GRIA3	HGNC Symbol	glutamate receptor, ionotropic, AMPA 3 [Source:HGNC Symbol;Acc:4573]
rs5911622	X	GRIA3	HGNC Symbol	glutamate receptor, ionotropic, AMPA 3 [Source:HGNC Symbol;Acc:4573]
rs6608087	X	GRIA3	HGNC Symbol	glutamate receptor, ionotropic, AMPA 3 [Source:HGNC Symbol;Acc:4573]
rs62594148	X	GRIA3	HGNC Symbol	glutamate receptor, ionotropic, AMPA 3 [Source:HGNC Symbol;Acc:4573]
rs503118	X	GRIA3	HGNC Symbol	glutamate receptor, ionotropic, AMPA 3 [Source:HGNC Symbol;Acc:4573]
rs5910005	X	GRIA3	HGNC Symbol	glutamate receptor, ionotropic, AMPA 3 [Source:HGNC Symbol;Acc:4573]
rs670021	X	GRIA3	HGNC Symbol	glutamate receptor, ionotropic, AMPA 3 [Source:HGNC Symbol;Acc:4573]
rs6649020	X	GRIA3	HGNC Symbol	glutamate receptor, ionotropic, AMPA 3 [Source:HGNC Symbol;Acc:4573]
rs113746553	X	GRIA3	HGNC Symbol	glutamate receptor, ionotropic, AMPA 3 [Source:HGNC Symbol;Acc:4573]
rs4825483				

SNP	Chromosome	gene name	gene source	description
rs4474149				
rs35551694				
rs144033913				
rs5911645				
rs4825486				
rs5910012				
rs12855173				
rs17325366				
rs2744309				
rs5911665				
rs73231113				
rs17259484				
rs56233537				
rs45460491	X	THOC2	HGNC Symbol	THO complex 2 [Source:HGNC Symbol;Acc:19073]
rs11796027	X	THOC2	HGNC Symbol	THO complex 2 [Source:HGNC Symbol;Acc:19073]
rs12841446	X	THOC2	HGNC Symbol	THO complex 2 [Source:HGNC Symbol;Acc:19073]
rs140825546	X	THOC2	HGNC Symbol	THO complex 2 [Source:HGNC Symbol;Acc:19073]
rs5956566	X	THOC2	HGNC Symbol	THO complex 2 [Source:HGNC Symbol;Acc:19073]
rs5911687	X	THOC2	HGNC Symbol	THO complex 2 [Source:HGNC Symbol;Acc:19073]
rs5911690	X	THOC2	HGNC Symbol	THO complex 2 [Source:HGNC Symbol;Acc:19073]
rs6649069	X	THOC2	HGNC Symbol	THO complex 2 [Source:HGNC Symbol;Acc:19073]
rs2982137				
rs2982139				
rs36053352				
rs35506394				
rs3005858				
rs5910038				
rs583				
rs2982144				
rs5958298				
rs56302102				
rs62604518				
rs3021259				

SNP	Chromosome	gene name	gene source	description
rs3005870				
rs3005871				
rs3005882				
rs3124045				
rs7878701				
rs3021238				
rs11260451				
rs3005851				
rs67456859				
rs145781515				
rs10907134	X	XIAP	HGNC Symbol	X-linked inhibitor of apoptosis [Source:HGNC Symbol;Acc:592]
rs34656663	X	XIAP	HGNC Symbol	X-linked inhibitor of apoptosis [Source:HGNC Symbol;Acc:592]
rs5958319	X	XIAP	HGNC Symbol	X-linked inhibitor of apoptosis [Source:HGNC Symbol;Acc:592]
rs7878541	X	XIAP	HGNC Symbol	X-linked inhibitor of apoptosis [Source:HGNC Symbol;Acc:592]
rs5956583	X	XIAP	HGNC Symbol	X-linked inhibitor of apoptosis [Source:HGNC Symbol;Acc:592]
rs12397329	X	XIAP	HGNC Symbol	X-linked inhibitor of apoptosis [Source:HGNC Symbol;Acc:592]
rs2269178	X	XIAP	HGNC Symbol	X-linked inhibitor of apoptosis [Source:HGNC Symbol;Acc:592]
rs9856	X	XIAP	HGNC Symbol	X-linked inhibitor of apoptosis [Source:HGNC Symbol;Acc:592]
rs73212905				
rs139078417				
rs68006212				
rs7889440				
rs4825497	X	STAG2	HGNC Symbol	stromal antigen 2 [Source:HGNC Symbol;Acc:11355]
rs199426	X	STAG2	HGNC Symbol	stromal antigen 2 [Source:HGNC Symbol;Acc:11355]
rs150298926	X	STAG2	HGNC Symbol	stromal antigen 2 [Source:HGNC Symbol;Acc:11355]
rs76876596	X	STAG2	HGNC Symbol	stromal antigen 2 [Source:HGNC Symbol;Acc:11355]
rs5911744	X	STAG2	HGNC Symbol	stromal antigen 2 [Source:HGNC Symbol;Acc:11355]
rs35941521	X	STAG2	HGNC Symbol	stromal antigen 2 [Source:HGNC Symbol;Acc:11355]
rs148298548	X	STAG2	HGNC Symbol	stromal antigen 2 [Source:HGNC Symbol;Acc:11355]
rs17330721	X	STAG2	HGNC Symbol	stromal antigen 2 [Source:HGNC Symbol;Acc:11355]
rs5958390	X	STAG2	HGNC Symbol	stromal antigen 2 [Source:HGNC Symbol;Acc:11355]
rs12844741	X	STAG2	HGNC Symbol	stromal antigen 2 [Source:HGNC Symbol;Acc:11355]
rs5956618	X	STAG2	HGNC Symbol	stromal antigen 2 [Source:HGNC Symbol;Acc:11355]

SNP	Chromosome	gene name	gene source	description
rs2801355	X	STAG2	HGNC Symbol	stromal antigen 2 [Source:HGNC Symbol;Acc:11355]
rs72610628	X	STAG2	HGNC Symbol	stromal antigen 2 [Source:HGNC Symbol;Acc:11355]
rs1279778	X	STAG2	HGNC Symbol	stromal antigen 2 [Source:HGNC Symbol;Acc:11355]
rs35971159	X	STAG2	HGNC Symbol	stromal antigen 2 [Source:HGNC Symbol;Acc:11355]
rs5911787	X	STAG2	HGNC Symbol	stromal antigen 2 [Source:HGNC Symbol;Acc:11355]
rs4825889	X	STAG2	HGNC Symbol	stromal antigen 2 [Source:HGNC Symbol;Acc:11355]
rs1781104	X	STAG2	HGNC Symbol	stromal antigen 2 [Source:HGNC Symbol;Acc:11355]
rs1798135	X	STAG2	HGNC Symbol	stromal antigen 2 [Source:HGNC Symbol;Acc:11355]
rs1279795	X	STAG2	HGNC Symbol	stromal antigen 2 [Source:HGNC Symbol;Acc:11355]
rs12558732	X	STAG2	HGNC Symbol	stromal antigen 2 [Source:HGNC Symbol;Acc:11355]
rs73212967	X	STAG2	HGNC Symbol	stromal antigen 2 [Source:HGNC Symbol;Acc:11355]
rs73212971	X	STAG2	HGNC Symbol	stromal antigen 2 [Source:HGNC Symbol;Acc:11355]
rs1279812	X	STAG2	HGNC Symbol	stromal antigen 2 [Source:HGNC Symbol;Acc:11355]
rs12687398	X	STAG2	HGNC Symbol	stromal antigen 2 [Source:HGNC Symbol;Acc:11355]
rs6608166	X	STAG2	HGNC Symbol	stromal antigen 2 [Source:HGNC Symbol;Acc:11355]
rs58807433	X	STAG2	HGNC Symbol	stromal antigen 2 [Source:HGNC Symbol;Acc:11355]
rs17330749	X	STAG2	HGNC Symbol	stromal antigen 2 [Source:HGNC Symbol;Acc:11355]
rs7057068	X	STAG2	HGNC Symbol	stromal antigen 2 [Source:HGNC Symbol;Acc:11355]
rs5958427	X	STAG2	HGNC Symbol	stromal antigen 2 [Source:HGNC Symbol;Acc:11355]
rs5910074	X	STAG2	HGNC Symbol	stromal antigen 2 [Source:HGNC Symbol;Acc:11355]
rs6649181	X	STAG2	HGNC Symbol	stromal antigen 2 [Source:HGNC Symbol;Acc:11355]
rs723538	X	STAG2	HGNC Symbol	stromal antigen 2 [Source:HGNC Symbol;Acc:11355]
rs7889198	X	STAG2	HGNC Symbol	stromal antigen 2 [Source:HGNC Symbol;Acc:11355]
rs1279822	X	STAG2	HGNC Symbol	stromal antigen 2 [Source:HGNC Symbol;Acc:11355]
rs6423132	X	STAG2	HGNC Symbol	stromal antigen 2 [Source:HGNC Symbol;Acc:11355]
rs12841499	X	STAG2	HGNC Symbol	stromal antigen 2 [Source:HGNC Symbol;Acc:11355]
rs1279819	X	STAG2	HGNC Symbol	stromal antigen 2 [Source:HGNC Symbol;Acc:11355]
rs1279818	X	STAG2	HGNC Symbol	stromal antigen 2 [Source:HGNC Symbol;Acc:11355]
rs1279817	X	STAG2	HGNC Symbol	stromal antigen 2 [Source:HGNC Symbol;Acc:11355]
rs6608181	X	STAG2	HGNC Symbol	stromal antigen 2 [Source:HGNC Symbol;Acc:11355]
rs73557901	X	STAG2	HGNC Symbol	stromal antigen 2 [Source:HGNC Symbol;Acc:11355]
rs66919192	X	STAG2	HGNC Symbol	stromal antigen 2 [Source:HGNC Symbol;Acc:11355]
rs5911806	X	STAG2	HGNC Symbol	stromal antigen 2 [Source:HGNC Symbol;Acc:11355]

SNP	Chromosome	gene name	gene source	description
rs1937190	X	STAG2	HGNC Symbol	stromal antigen 2 [Source:HGNC Symbol;Acc:11355]
rs151184635	X	STAG2	HGNC Symbol	stromal antigen 2 [Source:HGNC Symbol;Acc:11355]
rs2356408	X	STAG2	HGNC Symbol	stromal antigen 2 [Source:HGNC Symbol;Acc:11355]
rs1292209	X	STAG2	HGNC Symbol	stromal antigen 2 [Source:HGNC Symbol;Acc:11355]
rs62604220	X	STAG2	HGNC Symbol	stromal antigen 2 [Source:HGNC Symbol;Acc:11355]
rs62604221	X	STAG2	HGNC Symbol	stromal antigen 2 [Source:HGNC Symbol;Acc:11355]
rs5910079	X	STAG2	HGNC Symbol	stromal antigen 2 [Source:HGNC Symbol;Acc:11355]
rs2357205	X	STAG2	HGNC Symbol	stromal antigen 2 [Source:HGNC Symbol;Acc:11355]
rs5911814	X	STAG2	HGNC Symbol	stromal antigen 2 [Source:HGNC Symbol;Acc:11355]
rs9633189	X	STAG2;SH2D1A	HGNC Symbol	stromal antigen 2 [Source:HGNC Symbol;Acc:11355];SH2 domain containing 1A [Source:HGNC Symbol;Acc:10820]
rs5958470	X	STAG2;SH2D1A	HGNC Symbol	stromal antigen 2 [Source:HGNC Symbol;Acc:11355];SH2 domain containing 1A [Source:HGNC Symbol;Acc:10820]
rs148923326	X	STAG2;SH2D1A	HGNC Symbol	stromal antigen 2 [Source:HGNC Symbol;Acc:11355];SH2 domain containing 1A [Source:HGNC Symbol;Acc:10820]
rs73212989	X	STAG2;SH2D1A	HGNC Symbol	stromal antigen 2 [Source:HGNC Symbol;Acc:11355];SH2 domain containing 1A [Source:HGNC Symbol;Acc:10820]
rs2239481	X	STAG2;SH2D1A	HGNC Symbol	stromal antigen 2 [Source:HGNC Symbol;Acc:11355];SH2 domain containing 1A [Source:HGNC Symbol;Acc:10820]
rs62604250	X	STAG2;SH2D1A	HGNC Symbol	stromal antigen 2 [Source:HGNC Symbol;Acc:11355];SH2 domain containing 1A [Source:HGNC Symbol;Acc:10820]
rs73212990	X	STAG2;SH2D1A	HGNC Symbol	stromal antigen 2 [Source:HGNC Symbol;Acc:11355];SH2 domain containing 1A [Source:HGNC Symbol;Acc:10820]
rs2206018	X	STAG2	HGNC Symbol	stromal antigen 2 [Source:HGNC Symbol;Acc:11355]
rs10284179	X	STAG2	HGNC Symbol	stromal antigen 2 [Source:HGNC Symbol;Acc:11355]
rs6648597	X	TENM1;STAG2	HGNC Symbol	teneurin transmembrane protein 1 [Source:HGNC Symbol;Acc:8117];stromal antigen 2 [Source:HGNC Symbol;Acc:11355]
rs56659142	X	TENM1;STAG2	HGNC Symbol	teneurin transmembrane protein 1 [Source:HGNC Symbol;Acc:8117];stromal antigen 2 [Source:HGNC Symbol;Acc:11355]
rs144335593	X	TENM1;STAG2	HGNC Symbol	teneurin transmembrane protein 1 [Source:HGNC Symbol;Acc:8117];stromal antigen 2 [Source:HGNC Symbol;Acc:11355]
rs17325478	X	TENM1;STAG2	HGNC Symbol	teneurin transmembrane protein 1 [Source:HGNC Symbol;Acc:8117];stromal antigen 2 [Source:HGNC Symbol;Acc:11355]
rs6649213	X	TENM1;STAG2	HGNC Symbol	teneurin transmembrane protein 1 [Source:HGNC Symbol;Acc:8117];stromal antigen 2 [Source:HGNC Symbol;Acc:11355]
rs6649214	X	TENM1;STAG2	HGNC Symbol	teneurin transmembrane protein 1 [Source:HGNC Symbol;Acc:8117];stromal antigen 2 [Source:HGNC Symbol;Acc:11355]

SNP	Chromosome	gene name	gene source	description
rs2076165	X	TENM1;STAG2	HGNC Symbol	teneurin transmembrane protein 1 [Source:HGNC Symbol;Acc:8117];stromal antigen 2 [Source:HGNC Symbol;Acc:11355]
rs2357252	X	TENM1;STAG2	HGNC Symbol	teneurin transmembrane protein 1 [Source:HGNC Symbol;Acc:8117];stromal antigen 2 [Source:HGNC Symbol;Acc:11355]
rs2239478	X	TENM1;STAG2	HGNC Symbol	teneurin transmembrane protein 1 [Source:HGNC Symbol;Acc:8117];stromal antigen 2 [Source:HGNC Symbol;Acc:11355]
rs7055785	X	TENM1;STAG2	HGNC Symbol	teneurin transmembrane protein 1 [Source:HGNC Symbol;Acc:8117];stromal antigen 2 [Source:HGNC Symbol;Acc:11355]
rs960869	X	TENM1;STAG2	HGNC Symbol	teneurin transmembrane protein 1 [Source:HGNC Symbol;Acc:8117];stromal antigen 2 [Source:HGNC Symbol;Acc:11355]
rs5911832	X	TENM1;STAG2	HGNC Symbol	teneurin transmembrane protein 1 [Source:HGNC Symbol;Acc:8117];stromal antigen 2 [Source:HGNC Symbol;Acc:11355]
rs5911833	X	TENM1;STAG2	HGNC Symbol	teneurin transmembrane protein 1 [Source:HGNC Symbol;Acc:8117];stromal antigen 2 [Source:HGNC Symbol;Acc:11355]
rs12007060	X	TENM1;STAG2	HGNC Symbol	teneurin transmembrane protein 1 [Source:HGNC Symbol;Acc:8117];stromal antigen 2 [Source:HGNC Symbol;Acc:11355]
rs2239475	X	TENM1;STAG2	HGNC Symbol	teneurin transmembrane protein 1 [Source:HGNC Symbol;Acc:8117];stromal antigen 2 [Source:HGNC Symbol;Acc:11355]
rs12013090	X	TENM1;STAG2	HGNC Symbol	teneurin transmembrane protein 1 [Source:HGNC Symbol;Acc:8117];stromal antigen 2 [Source:HGNC Symbol;Acc:11355]
rs2076164	X	TENM1;STAG2	HGNC Symbol	teneurin transmembrane protein 1 [Source:HGNC Symbol;Acc:8117];stromal antigen 2 [Source:HGNC Symbol;Acc:11355]
rs2072886	X	TENM1;STAG2	HGNC Symbol	teneurin transmembrane protein 1 [Source:HGNC Symbol;Acc:8117];stromal antigen 2 [Source:HGNC Symbol;Acc:11355]
rs2294414	X	TENM1;STAG2	HGNC Symbol	teneurin transmembrane protein 1 [Source:HGNC Symbol;Acc:8117];stromal antigen 2 [Source:HGNC Symbol;Acc:11355]
rs5958487	X	TENM1	HGNC Symbol	teneurin transmembrane protein 1 [Source:HGNC Symbol;Acc:8117]
rs7889643	X	TENM1	HGNC Symbol	teneurin transmembrane protein 1 [Source:HGNC Symbol;Acc:8117]
rs5911855	X	TENM1	HGNC Symbol	teneurin transmembrane protein 1 [Source:HGNC Symbol;Acc:8117]
rs7058630	X	TENM1	HGNC Symbol	teneurin transmembrane protein 1 [Source:HGNC Symbol;Acc:8117]
rs909774	X	TENM1	HGNC Symbol	teneurin transmembrane protein 1 [Source:HGNC Symbol;Acc:8117]
rs56108083	X	TENM1	HGNC Symbol	teneurin transmembrane protein 1 [Source:HGNC Symbol;Acc:8117]
rs5956658	X	TENM1	HGNC Symbol	teneurin transmembrane protein 1 [Source:HGNC Symbol;Acc:8117]
rs5958493	X	TENM1	HGNC Symbol	teneurin transmembrane protein 1 [Source:HGNC Symbol;Acc:8117]
rs111472283	X	TENM1	HGNC Symbol	teneurin transmembrane protein 1 [Source:HGNC Symbol;Acc:8117]
rs5958495	X	TENM1	HGNC Symbol	teneurin transmembrane protein 1 [Source:HGNC Symbol;Acc:8117]
rs2266907	X	TENM1	HGNC Symbol	teneurin transmembrane protein 1 [Source:HGNC Symbol;Acc:8117]
rs7885450	X	TENM1	HGNC Symbol	teneurin transmembrane protein 1 [Source:HGNC Symbol;Acc:8117]

SNP	Chromosome	gene name	gene source	description
rs2266904	X	TENM1	HGNC Symbol	teneurin transmembrane protein 1 [Source:HGNC Symbol;Acc:8117]
rs41312783	X	TENM1	HGNC Symbol	teneurin transmembrane protein 1 [Source:HGNC Symbol;Acc:8117]
rs12393597	X	TENM1	HGNC Symbol	teneurin transmembrane protein 1 [Source:HGNC Symbol;Acc:8117]
rs17330825	X	TENM1	HGNC Symbol	teneurin transmembrane protein 1 [Source:HGNC Symbol;Acc:8117]
rs16305	X	TENM1	HGNC Symbol	teneurin transmembrane protein 1 [Source:HGNC Symbol;Acc:8117]
rs12011051	X	TENM1	HGNC Symbol	teneurin transmembrane protein 1 [Source:HGNC Symbol;Acc:8117]
rs16301	X	TENM1	HGNC Symbol	teneurin transmembrane protein 1 [Source:HGNC Symbol;Acc:8117]
rs6649251	X	TENM1	HGNC Symbol	teneurin transmembrane protein 1 [Source:HGNC Symbol;Acc:8117]
rs2283764	X	TENM1	HGNC Symbol	teneurin transmembrane protein 1 [Source:HGNC Symbol;Acc:8117]
rs13328496	X	TENM1	HGNC Symbol	teneurin transmembrane protein 1 [Source:HGNC Symbol;Acc:8117]
rs115272341	X	TENM1	HGNC Symbol	teneurin transmembrane protein 1 [Source:HGNC Symbol;Acc:8117]
rs73215115	X	TENM1	HGNC Symbol	teneurin transmembrane protein 1 [Source:HGNC Symbol;Acc:8117]
rs12390320	X	TENM1	HGNC Symbol	teneurin transmembrane protein 1 [Source:HGNC Symbol;Acc:8117]
rs5958513	X	TENM1	HGNC Symbol	teneurin transmembrane protein 1 [Source:HGNC Symbol;Acc:8117]
rs113906589	X	TENM1	HGNC Symbol	teneurin transmembrane protein 1 [Source:HGNC Symbol;Acc:8117]
rs12006619	X	TENM1	HGNC Symbol	teneurin transmembrane protein 1 [Source:HGNC Symbol;Acc:8117]
rs2858445	X	TENM1	HGNC Symbol	teneurin transmembrane protein 1 [Source:HGNC Symbol;Acc:8117]
rs3788776	X	TENM1	HGNC Symbol	teneurin transmembrane protein 1 [Source:HGNC Symbol;Acc:8117]
rs2843520	X	TENM1	HGNC Symbol	teneurin transmembrane protein 1 [Source:HGNC Symbol;Acc:8117]
rs2858443	X	TENM1	HGNC Symbol	teneurin transmembrane protein 1 [Source:HGNC Symbol;Acc:8117]
rs2858417	X	TENM1	HGNC Symbol	teneurin transmembrane protein 1 [Source:HGNC Symbol;Acc:8117]
rs2843512	X	TENM1	HGNC Symbol	teneurin transmembrane protein 1 [Source:HGNC Symbol;Acc:8117]
rs5956666	X	TENM1	HGNC Symbol	teneurin transmembrane protein 1 [Source:HGNC Symbol;Acc:8117]
rs2249084	X	TENM1	HGNC Symbol	teneurin transmembrane protein 1 [Source:HGNC Symbol;Acc:8117]
rs73215124	X	TENM1	HGNC Symbol	teneurin transmembrane protein 1 [Source:HGNC Symbol;Acc:8117]
rs5958542	X	TENM1	HGNC Symbol	teneurin transmembrane protein 1 [Source:HGNC Symbol;Acc:8117]
rs73558352	X	TENM1	HGNC Symbol	teneurin transmembrane protein 1 [Source:HGNC Symbol;Acc:8117]
rs7884675	X	TENM1	HGNC Symbol	teneurin transmembrane protein 1 [Source:HGNC Symbol;Acc:8117]
rs73215127	X	TENM1	HGNC Symbol	teneurin transmembrane protein 1 [Source:HGNC Symbol;Acc:8117]
rs73215129	X	TENM1	HGNC Symbol	teneurin transmembrane protein 1 [Source:HGNC Symbol;Acc:8117]
rs17325547	X	TENM1	HGNC Symbol	teneurin transmembrane protein 1 [Source:HGNC Symbol;Acc:8117]
rs2213591	X	TENM1	HGNC Symbol	teneurin transmembrane protein 1 [Source:HGNC Symbol;Acc:8117]
rs5956674	X	TENM1	HGNC Symbol	teneurin transmembrane protein 1 [Source:HGNC Symbol;Acc:8117]

SNP	Chromosome	gene name	gene source	description
rs5958557	X	TENM1	HGNC Symbol	teneurin transmembrane protein 1 [Source:HGNC Symbol;Acc:8117]
rs73215133	X	TENM1	HGNC Symbol	teneurin transmembrane protein 1 [Source:HGNC Symbol;Acc:8117]
rs2206237	X	TENM1	HGNC Symbol	teneurin transmembrane protein 1 [Source:HGNC Symbol;Acc:8117]
rs3859908	X	TENM1	HGNC Symbol	teneurin transmembrane protein 1 [Source:HGNC Symbol;Acc:8117]
rs147213913	X	TENM1	HGNC Symbol	teneurin transmembrane protein 1 [Source:HGNC Symbol;Acc:8117]
rs73215142	X	TENM1	HGNC Symbol	teneurin transmembrane protein 1 [Source:HGNC Symbol;Acc:8117]
rs73215144	X	TENM1	HGNC Symbol	teneurin transmembrane protein 1 [Source:HGNC Symbol;Acc:8117]
rs1569565	X	TENM1	HGNC Symbol	teneurin transmembrane protein 1 [Source:HGNC Symbol;Acc:8117]
rs6655801	X	TENM1	HGNC Symbol	teneurin transmembrane protein 1 [Source:HGNC Symbol;Acc:8117]
rs62604295	X	TENM1	HGNC Symbol	teneurin transmembrane protein 1 [Source:HGNC Symbol;Acc:8117]
rs112619548	X	TENM1	HGNC Symbol	teneurin transmembrane protein 1 [Source:HGNC Symbol;Acc:8117]
rs6655802	X	TENM1	HGNC Symbol	teneurin transmembrane protein 1 [Source:HGNC Symbol;Acc:8117]
rs2223460	X	TENM1	HGNC Symbol	teneurin transmembrane protein 1 [Source:HGNC Symbol;Acc:8117]
rs5958588	X	TENM1	HGNC Symbol	teneurin transmembrane protein 1 [Source:HGNC Symbol;Acc:8117]
rs73215148	X	TENM1	HGNC Symbol	teneurin transmembrane protein 1 [Source:HGNC Symbol;Acc:8117]
rs62604297	X	TENM1	HGNC Symbol	teneurin transmembrane protein 1 [Source:HGNC Symbol;Acc:8117]
rs73215153	X	TENM1	HGNC Symbol	teneurin transmembrane protein 1 [Source:HGNC Symbol;Acc:8117]
rs5911908	X	TENM1	HGNC Symbol	teneurin transmembrane protein 1 [Source:HGNC Symbol;Acc:8117]
rs73215156	X	TENM1	HGNC Symbol	teneurin transmembrane protein 1 [Source:HGNC Symbol;Acc:8117]
rs148260483	X	TENM1	HGNC Symbol	teneurin transmembrane protein 1 [Source:HGNC Symbol;Acc:8117]
rs6649298	X	TENM1	HGNC Symbol	teneurin transmembrane protein 1 [Source:HGNC Symbol;Acc:8117]
rs5911917	X	TENM1	HGNC Symbol	teneurin transmembrane protein 1 [Source:HGNC Symbol;Acc:8117]
rs73215170	X	TENM1	HGNC Symbol	teneurin transmembrane protein 1 [Source:HGNC Symbol;Acc:8117]
rs12842370	X	TENM1	HGNC Symbol	teneurin transmembrane protein 1 [Source:HGNC Symbol;Acc:8117]
rs150455251	X	TENM1	HGNC Symbol	teneurin transmembrane protein 1 [Source:HGNC Symbol;Acc:8117]
rs5911931				
rs75703158				
rs5911935				
rs2032493				
rs17325575				
rs5958652				
rs7887647				
rs5910128				

SNP	Chromosome	gene name	gene source	description
rs5910134				
rs2227146				
rs1894576				
rs1894574				
rs28564405				
rs1005379				
rs17259715				
rs147922501				
rs16998322				
rs2213484				
rs16998333				
rs28599959				
rs5911985				
rs2213486				
rs2213488				
rs17330937				
rs5958696				
rs111475138				
rs17325624				
rs73216133				
rs5912002				
rs66831211				
rs72610661				
rs762966				
rs5912013				
rs6649336				
rs28370505				
rs7881188				
rs6649796				
rs6649797				
rs3924466				
rs62613098				
rs3126329				

SNP	Chromosome	gene name	gene source	description
rs3135237				
rs56292951				
rs2016462				
rs2078600				
rs73216144				
rs3135286				
rs72610676				
rs3126111				
rs3101163				
rs56063902				
rs3126101				
rs28578072				
rs2219480				
rs146818456				
rs3101137				
rs3126295				
rs1903090				
rs4830000				
rs148075998				
rs73216164				
rs1017715				
rs144267858				
rs5931896				
rs66986349				
rs16998481				
rs28369463				
rs28626864				
rs212253				
rs1580221				
rs6637317				
rs212266				
rs5976698				
rs177021				

SNP	Chromosome	gene name	gene source	description
rs5932271				
rs5932316				
rs140035259				
rs5976774				
rs5930250				
rs5932415				
rs2858278				
rs6649827				
rs79658279				
rs141577400				
rs6649824				
rs3128747				
rs56340315				
rs5930345				
rs6649791				
rs147568247				
rs17265994				
rs62611739				
rs73214455				
rs57230270				
rs73214463				
rs6649801				
rs5932823				
rs5932947				
rs6637878				
rs12853532				
rs7882893				
rs582694				
rs209624				
rs209623				
rs1305003				
rs209658				
rs139880202				

SNP	Chromosome	gene name	gene source	description
rs62614261				
rs7056175				
rs5977814				
rs7066275				
rs2878431				
rs73548852				
rs143123657				
rs7053815				
rs111687805				
rs12009868				
rs5930648				
rs7882213				
rs5978043				
rs55999191				
rs5978056				
rs2839957				
rs5933485				
rs6529663				
rs12835638				
rs5930675				
rs184301362				
rs112644739				
rs1290578				
rs845322				
rs5930842				
rs5975669				
rs1418125				
rs6635264				
rs146355436				
rs73557182				
rs73219998				
rs73559207				
rs5929773				

SNP	Chromosome	gene name	gene source	description
rs4489435				
rs73221707				
rs17303386				
rs5931143				
rs5931152				
rs4829890				
rs143008718				
rs73632039				
rs1984384				
rs2515986				
rs5976003				
rs73221718				
rs111732512				
rs4518776				
rs5974724				
rs4645676				
rs4497100				
rs16998832				
rs141910145				
rs5976232				
rs12009075				
rs17218338				
rs73223603				
rs5929983				
rs113487906				
rs6635844				
rs12845376				
rs62609661				
rs5930016				
rs7890915				
rs7474176				
rs5974852				
rs5931715	X	CXorf64	HGNC Symbol	chromosome X open reading frame 64 [Source:HGNC Symbol;Acc:34498]

SNP	Chromosome	gene name	gene source	description
rs12835991	X	CXorf64	HGNC Symbol	chromosome X open reading frame 64 [Source:HGNC Symbol;Acc:34498]
rs4289953	X	CXorf64	HGNC Symbol	chromosome X open reading frame 64 [Source:HGNC Symbol;Acc:34498]
rs2269777	X	CXorf64	HGNC Symbol	chromosome X open reading frame 64 [Source:HGNC Symbol;Acc:34498]
rs5976427				
rs5976429				
rs3131365				
rs73216557				
rs5976441				
rs73216567				
rs146916410				
rs4378102				
rs4830018				
rs12013554				
rs17261707				
rs5931796				
rs5931806				
rs144084862				
rs145731737				
rs11798246				
rs146014476				
rs201641				
rs141765152				
rs139203184				
rs12833994				
rs2097341				
rs148420459				
rs5931936				
rs16992509				
rs12832529				
rs6634600				
rs73216923				
rs5974912				
rs139081411				

SNP	Chromosome	gene name	gene source	description
rs5930140				
rs11096160				
rs35833461				
rs57519058				
rs5976634				
rs226578				
rs12559365				
rs140027627				
rs226528				
rs6637326				
rs5974972				
rs73236598				
rs78282377				
rs12839649				
rs6637381				
rs5976740				
rs6634654				
rs732182	X	ACTRT1	HGNC Symbol	actin-related protein T1 [Source:HGNC Symbol;Acc:24027]
rs61732204	X	ACTRT1	HGNC Symbol	actin-related protein T1 [Source:HGNC Symbol;Acc:24027]
rs73238923				
rs2858111				
rs62598278				
rs17218701				
rs145182934				
rs6637420				
rs11796826				
rs192146851				
rs73557654				
rs147044484	X	RP1-30E17.2	Clone-based (Vega) gene	
rs240583	X	RP1-30E17.2	Clone-based (Vega) gene	
rs5975024	X	RP1-30E17.2	Clone-based (Vega) gene	

SNP	Chromosome	gene name	gene source	description
rs5976811	X	RP1-30E17.2	Clone-based (Vega) gene	
rs111291419	X	RP1-30E17.2	Clone-based (Vega) gene	
rs5932430	X	RP1-30E17.2	Clone-based (Vega) gene	
rs144080138	X	RP1-30E17.2	Clone-based (Vega) gene	
rs7055882	X	RP1-30E17.2	Clone-based (Vega) gene	
rs149298622	X	RP1-30E17.2	Clone-based (Vega) gene	
rs112163949	X	RP1-30E17.2	Clone-based (Vega) gene	
rs11796949	X	RP1-30E17.2	Clone-based (Vega) gene	
rs58886746	X	RP1-30E17.2	Clone-based (Vega) gene	
rs16999538	X	RP1-30E17.2	Clone-based (Vega) gene	
rs55839915	X	RP1-30E17.2	Clone-based (Vega) gene	
rs62601020				
rs5976834				
rs5932462				
rs5976837				
rs2027843				
rs7056693				
rs5932467				
rs111500731				
rs62598905				
rs1034430				
rs12859839				
rs12851376				
rs56305467				
rs2105888				
rs5930274				

SNP	Chromosome	gene name	gene source	description
rs6637473				
rs150828057				
rs5976878				
rs149011695				
rs5932489				
rs73223318				
rs4829683				
rs653				
rs916208				
rs4355963				
rs5932520				
rs7880219				
rs4830101				
rs73555931				
rs73223342				
rs190847911				
rs12559502				
rs55803536				
rs5975066				
rs5930301				
rs11796064				
rs5932549				
rs1204090				
rs5932555				
rs6418922				
rs112966317				
rs12688007				
rs1572971				
rs11096220				
rs4830111				
rs6529327				
rs5976952				
rs73223367				

SNP	Chromosome	gene name	gene source	description
rs149719035				
rs145339824				
rs73223375				
rs41435452				
rs5930313				
rs73223381				
rs5975081				
rs2213619				
rs737159				
rs139578712				
rs5977001				
rs12850132				
rs16318				
rs1997631				
rs17304482				
rs73225513				
rs2223197				
rs34232029				
rs5932597				
rs73225520				
rs5930326				
rs141453892				
rs6637562				
rs2364347				
rs10521754				
rs1923842				
rs6637566				
rs55727078				
rs7064427				
rs6529359				
rs143776808				
rs1474609				
rs12010865				

SNP	Chromosome	gene name	gene source	description
rs149639464				
rs2206011				
rs1324149				
rs1324151	X	SMARCA1	HGNC Symbol	SWI/SNF related, matrix associated, actin dependent regulator of chromatin, subfamily a, member 1 [Source:HGNC Symbol;Acc:11097]
rs12850442	X	SMARCA1	HGNC Symbol	SWI/SNF related, matrix associated, actin dependent regulator of chromatin, subfamily a, member 1 [Source:HGNC Symbol;Acc:11097]
rs6529385	X	SMARCA1	HGNC Symbol	SWI/SNF related, matrix associated, actin dependent regulator of chromatin, subfamily a, member 1 [Source:HGNC Symbol;Acc:11097]
rs5932632	X	SMARCA1	HGNC Symbol	SWI/SNF related, matrix associated, actin dependent regulator of chromatin, subfamily a, member 1 [Source:HGNC Symbol;Acc:11097]
rs3118104	X	SMARCA1	HGNC Symbol	SWI/SNF related, matrix associated, actin dependent regulator of chromatin, subfamily a, member 1 [Source:HGNC Symbol;Acc:11097]
rs3131274				
rs143145263				
rs3131278				
rs5930347				
rs138464761	X	OCRL	HGNC Symbol	oculocerebrorenal syndrome of Lowe [Source:HGNC Symbol;Acc:8108]
rs5977104	X	OCRL	HGNC Symbol	oculocerebrorenal syndrome of Lowe [Source:HGNC Symbol;Acc:8108]
rs17304790	X	OCRL	HGNC Symbol	oculocerebrorenal syndrome of Lowe [Source:HGNC Symbol;Acc:8108]
rs5977112	X	OCRL	HGNC Symbol	oculocerebrorenal syndrome of Lowe [Source:HGNC Symbol;Acc:8108]
rs2362937	X	OCRL	HGNC Symbol	oculocerebrorenal syndrome of Lowe [Source:HGNC Symbol;Acc:8108]
rs2179205				
rs5977121				
rs3116753				
rs3115759	X	APLN	HGNC Symbol	apelin [Source:HGNC Symbol;Acc:16665]
rs2235308	X	APLN	HGNC Symbol	apelin [Source:HGNC Symbol;Acc:16665]
rs3116744				
rs62608329				
rs12156961				
rs1997670				
rs6637643				
rs909655				
rs5932662				
rs4829706				

SNP	Chromosome	gene name	gene source	description
rs5932667				
rs56102455	X	XPNPEP2	HGNC Symbol	X-prolyl aminopeptidase (aminopeptidase P) 2, membrane-bound [Source:HGNC Symbol;Acc:12823]
rs5975145	X	XPNPEP2	HGNC Symbol	X-prolyl aminopeptidase (aminopeptidase P) 2, membrane-bound [Source:HGNC Symbol;Acc:12823]
rs5932672	X	XPNPEP2	HGNC Symbol	X-prolyl aminopeptidase (aminopeptidase P) 2, membrane-bound [Source:HGNC Symbol;Acc:12823]
rs3747343	X	XPNPEP2	HGNC Symbol	X-prolyl aminopeptidase (aminopeptidase P) 2, membrane-bound [Source:HGNC Symbol;Acc:12823]
rs4830167	X	XPNPEP2	HGNC Symbol	X-prolyl aminopeptidase (aminopeptidase P) 2, membrane-bound [Source:HGNC Symbol;Acc:12823]
rs41311662	X	XPNPEP2	HGNC Symbol	X-prolyl aminopeptidase (aminopeptidase P) 2, membrane-bound [Source:HGNC Symbol;Acc:12823]
rs1771601	X	XPNPEP2	HGNC Symbol	X-prolyl aminopeptidase (aminopeptidase P) 2, membrane-bound [Source:HGNC Symbol;Acc:12823]
rs859584	X	XPNPEP2	HGNC Symbol	X-prolyl aminopeptidase (aminopeptidase P) 2, membrane-bound [Source:HGNC Symbol;Acc:12823]
rs11795787	X	XPNPEP2	HGNC Symbol	X-prolyl aminopeptidase (aminopeptidase P) 2, membrane-bound [Source:HGNC Symbol;Acc:12823]
rs859585	X	XPNPEP2	HGNC Symbol	X-prolyl aminopeptidase (aminopeptidase P) 2, membrane-bound [Source:HGNC Symbol;Acc:12823]
rs12007179				
rs60006060				
rs6637657				
rs11798976	X	SASH3	HGNC Symbol	SAM and SH3 domain containing 3 [Source:HGNC Symbol;Acc:15975]
rs859602	X	SASH3	HGNC Symbol	SAM and SH3 domain containing 3 [Source:HGNC Symbol;Acc:15975]
rs5932684	X	SASH3	HGNC Symbol	SAM and SH3 domain containing 3 [Source:HGNC Symbol;Acc:15975]
rs41300283	X	SASH3;RP4-753P9.3	HGNC Symbol;Clone-based (Vega) gene	SAM and SH3 domain containing 3 [Source:HGNC Symbol;Acc:15975];
rs5903790				
rs11990	X	ZDHHC9	HGNC Symbol	zinc finger, DHHC-type containing 9 [Source:HGNC Symbol;Acc:18475]
rs3747340	X	ZDHHC9	HGNC Symbol	zinc finger, DHHC-type containing 9 [Source:HGNC Symbol;Acc:18475]
rs45522933	X	ZDHHC9	HGNC Symbol	zinc finger, DHHC-type containing 9 [Source:HGNC Symbol;Acc:18475]
rs3810699				
rs148001993				
rs145301495				

SNP	Chromosome	gene name	gene source	description
rs138782780				
rs7890054	X	RP4-537K23.4	Clone-based (Vega) gene	
rs5977166	X	RP4-537K23.4	Clone-based (Vega) gene	
rs112072518	X	RP4-537K23.4	Clone-based (Vega) gene	
rs73225590	X	UTP14A;RP4-537K23.4	HGNC Symbol;Clone-based (Vega) gene	UTP14, U3 small nucleolar ribonucleoprotein, homolog A (yeast) [Source:HGNC Symbol;Acc:10665];
rs2281278	X	UTP14A;RP4-537K23.4	HGNC Symbol;Clone-based (Vega) gene	UTP14, U3 small nucleolar ribonucleoprotein, homolog A (yeast) [Source:HGNC Symbol;Acc:10665];
rs4830171	X	RP4-537K23.4	Clone-based (Vega) gene	
rs73633938				
rs73633939				
rs5977184				
rs113865958				
rs146852038	X	BCORL1	HGNC Symbol	BCL6 corepressor-like 1 [Source:HGNC Symbol;Acc:25657]
rs143325572	X	BCORL1	HGNC Symbol	BCL6 corepressor-like 1 [Source:HGNC Symbol;Acc:25657]
rs5932715	X	BCORL1	HGNC Symbol	BCL6 corepressor-like 1 [Source:HGNC Symbol;Acc:25657]
rs73225593	X	BCORL1	HGNC Symbol	BCL6 corepressor-like 1 [Source:HGNC Symbol;Acc:25657]
rs3788848	X	ELF4	HGNC Symbol	E74-like factor 4 (ets domain transcription factor) [Source:HGNC Symbol;Acc:3319]
rs5932717	X	ELF4	HGNC Symbol	E74-like factor 4 (ets domain transcription factor) [Source:HGNC Symbol;Acc:3319]
rs7050694	X	ELF4	HGNC Symbol	E74-like factor 4 (ets domain transcription factor) [Source:HGNC Symbol;Acc:3319]
rs3848963	X	ELF4	HGNC Symbol	E74-like factor 4 (ets domain transcription factor) [Source:HGNC Symbol;Acc:3319]
rs210009	X	ELF4	HGNC Symbol	E74-like factor 4 (ets domain transcription factor) [Source:HGNC Symbol;Acc:3319]
rs209988				
rs73225598				
rs73225599	X	AIFM1	HGNC Symbol	apoptosis-inducing factor, mitochondrion-associated, 1 [Source:HGNC Symbol;Acc:8768]
rs12559753	X	AIFM1	HGNC Symbol	apoptosis-inducing factor, mitochondrion-associated, 1 [Source:HGNC Symbol;Acc:8768]
rs209555				
rs5932726				
rs41302144	X	ZNF280C	HGNC Symbol	zinc finger protein 280C [Source:HGNC Symbol;Acc:25955]
rs5930385	X	ZNF280C	HGNC Symbol	zinc finger protein 280C [Source:HGNC Symbol;Acc:25955]

SNP	Chromosome	gene name	gene source	description
rs673667	X	ZNF280C	HGNC Symbol	zinc finger protein 280C [Source:HGNC Symbol;Acc:25955]
rs9969910				
rs62601165				
rs5930396				
rs2239483	X	SLC25A14	HGNC Symbol	solute carrier family 25 (mitochondrial carrier, brain), member 14 [Source:HGNC Symbol;Acc:10984]
rs1010978	X	SLC25A14	HGNC Symbol	solute carrier family 25 (mitochondrial carrier, brain), member 14 [Source:HGNC Symbol;Acc:10984]
rs59626676	X	SLC25A14	HGNC Symbol	solute carrier family 25 (mitochondrial carrier, brain), member 14 [Source:HGNC Symbol;Acc:10984]
rs2235800	X	SLC25A14	HGNC Symbol	solute carrier family 25 (mitochondrial carrier, brain), member 14 [Source:HGNC Symbol;Acc:10984]
rs5977250	X	SLC25A14	HGNC Symbol	solute carrier family 25 (mitochondrial carrier, brain), member 14 [Source:HGNC Symbol;Acc:10984]
rs151331408				
rs144741515	X	RBMX2	HGNC Symbol	RNA binding motif protein, X-linked 2 [Source:HGNC Symbol;Acc:24282]
rs2294956	X	RBMX2	HGNC Symbol	RNA binding motif protein, X-linked 2 [Source:HGNC Symbol;Acc:24282]
rs17268265				
rs7880254				
rs147833569				
rs17305432				
rs5932770	X	RP1-274L7.1	Clone-based (Vega) gene	
rs5977301	X	RP1-274L7.1	Clone-based (Vega) gene	
rs16980163	X	RP1-274L7.1	Clone-based (Vega) gene	
rs41301495	X	FAM45B;RP1-274L7.1	HGNC Symbol;Clone-based (Vega) gene	family with sequence similarity 45, member B (pseudogene) [Source:HGNC Symbol;Acc:30886];
rs7056257	X	RP1-274L7.1	Clone-based (Vega) gene	
rs12559307	X	RP1-274L7.1	Clone-based (Vega) gene	
rs73635201				
rs3007758				
rs142984078				
rs5977322				
rs73229064				
rs5975223	X	ENOX2	HGNC Symbol	ecto-NOX disulfide-thiol exchanger 2 [Source:HGNC Symbol;Acc:2259]

SNP	Chromosome	gene name	gene source	description
rs34943384	X	ENOX2	HGNC Symbol	ecto-NOX disulfide-thiol exchanger 2 [Source:HGNC Symbol;Acc:2259]
rs73229071	X	ENOX2	HGNC Symbol	ecto-NOX disulfide-thiol exchanger 2 [Source:HGNC Symbol;Acc:2259]
rs140886270	X	ENOX2	HGNC Symbol	ecto-NOX disulfide-thiol exchanger 2 [Source:HGNC Symbol;Acc:2259]
rs5975231	X	ENOX2	HGNC Symbol	ecto-NOX disulfide-thiol exchanger 2 [Source:HGNC Symbol;Acc:2259]
rs5932817	X	ENOX2	HGNC Symbol	ecto-NOX disulfide-thiol exchanger 2 [Source:HGNC Symbol;Acc:2259]
rs140427927	X	ENOX2	HGNC Symbol	ecto-NOX disulfide-thiol exchanger 2 [Source:HGNC Symbol;Acc:2259]
rs6529451	X	ENOX2	HGNC Symbol	ecto-NOX disulfide-thiol exchanger 2 [Source:HGNC Symbol;Acc:2259]
rs2864894	X	ENOX2	HGNC Symbol	ecto-NOX disulfide-thiol exchanger 2 [Source:HGNC Symbol;Acc:2259]
rs5930430				
rs17316199				
rs112184180				
rs78831073	X	RP1-23K20.2	Clone-based (Vega) gene	
rs138591016	X	RP1-23K20.2	Clone-based (Vega) gene	
rs141782995	X	RP1-23K20.2	Clone-based (Vega) gene	
rs73229083	X	RP1-23K20.2	Clone-based (Vega) gene	
rs5977398	X	RP1-23K20.2	Clone-based (Vega) gene	
rs62603944	X	RP1-23K20.2	Clone-based (Vega) gene	
rs10521762	X	RP1-23K20.2	Clone-based (Vega) gene	
rs6637789	X	RP1-23K20.2	Clone-based (Vega) gene	
rs5977409	X	ARHGAP36	HGNC Symbol	Rho GTPase activating protein 36 [Source:HGNC Symbol;Acc:26388]
rs4506388	X	ARHGAP36	HGNC Symbol	Rho GTPase activating protein 36 [Source:HGNC Symbol;Acc:26388]
rs60028314	X	ARHGAP36	HGNC Symbol	Rho GTPase activating protein 36 [Source:HGNC Symbol;Acc:26388]
rs10521763	X	ARHGAP36	HGNC Symbol	Rho GTPase activating protein 36 [Source:HGNC Symbol;Acc:26388]
rs3788	X	ARHGAP36	HGNC Symbol	Rho GTPase activating protein 36 [Source:HGNC Symbol;Acc:26388]
rs7883161				
rs4829723				
rs5932848				
rs73233155				

SNP	Chromosome	gene name	gene source	description
rs73233157				
rs5932855				
rs5932857				
rs17316366				
rs56675839				
rs58301334				
rs6634815				
rs6634819				
rs6637824				
rs73635985	X	IGSF1	HGNC Symbol	immunoglobulin superfamily, member 1 [Source:HGNC Symbol;Acc:5948]
rs4830219	X	IGSF1	HGNC Symbol	immunoglobulin superfamily, member 1 [Source:HGNC Symbol;Acc:5948]
rs4524963	X	IGSF1	HGNC Symbol	immunoglobulin superfamily, member 1 [Source:HGNC Symbol;Acc:5948]
rs4830220	X	IGSF1	HGNC Symbol	immunoglobulin superfamily, member 1 [Source:HGNC Symbol;Acc:5948]
rs139140137	X	IGSF1	HGNC Symbol	immunoglobulin superfamily, member 1 [Source:HGNC Symbol;Acc:5948]
rs73568436	X	IGSF1	HGNC Symbol	immunoglobulin superfamily, member 1 [Source:HGNC Symbol;Acc:5948]
rs56799010	X	IGSF1	HGNC Symbol	immunoglobulin superfamily, member 1 [Source:HGNC Symbol;Acc:5948]
rs5932883	X	IGSF1	HGNC Symbol	immunoglobulin superfamily, member 1 [Source:HGNC Symbol;Acc:5948]
rs5932884	X	IGSF1	HGNC Symbol	immunoglobulin superfamily, member 1 [Source:HGNC Symbol;Acc:5948]
rs73233186	X	IGSF1	HGNC Symbol	immunoglobulin superfamily, member 1 [Source:HGNC Symbol;Acc:5948]
rs146342487	X	IGSF1;OR5AW1P	HGNC Symbol	immunoglobulin superfamily, member 1 [Source:HGNC Symbol;Acc:5948];olfactory receptor, family 5, subfamily AW, member 1 pseudogene [Source:HGNC Symbol;Acc:15406]
rs67579737	X	IGSF1	HGNC Symbol	immunoglobulin superfamily, member 1 [Source:HGNC Symbol;Acc:5948]
rs144250919	X	IGSF1	HGNC Symbol	immunoglobulin superfamily, member 1 [Source:HGNC Symbol;Acc:5948]
rs67582293	X	IGSF1	HGNC Symbol	immunoglobulin superfamily, member 1 [Source:HGNC Symbol;Acc:5948]
rs5977475	X	IGSF1	HGNC Symbol	immunoglobulin superfamily, member 1 [Source:HGNC Symbol;Acc:5948]
rs147405550	X	IGSF1	HGNC Symbol	immunoglobulin superfamily, member 1 [Source:HGNC Symbol;Acc:5948]
rs62616574				
rs2208697				
rs150333093				
rs73235176				
rs653398				
rs5932929				
rs499030	X	OR13H1	HGNC Symbol	olfactory receptor, family 13, subfamily H, member 1 [Source:HGNC Symbol;Acc:14755]

SNP	Chromosome	gene name	gene source	description
rs17316625	X	OR13H1	HGNC Symbol	olfactory receptor, family 13, subfamily H, member 1 [Source:HGNC Symbol;Acc:14755]
rs655415	X	OR13H1	HGNC Symbol	olfactory receptor, family 13, subfamily H, member 1 [Source:HGNC Symbol;Acc:14755]
rs112266689				
rs707252				
rs6637876				
rs76143819				
rs859864				
rs859867				
rs55961428				
rs143119152				
rs112638577				
rs6634838				
rs5977530				
rs58736069				
rs5977533				
rs142893763	X	RP11-453F18__B.1	Clone-based (Vega) gene	
rs872840	X	RP11-453F18__B.1	Clone-based (Vega) gene	
rs111362277	X	RP11-453F18__B.1	Clone-based (Vega) gene	
rs111892598	X	RP11-453F18__B.1	Clone-based (Vega) gene	
rs5930506	X	RP11-453F18__B.1	Clone-based (Vega) gene	
rs4830231	X	RP11-453F18__B.1	Clone-based (Vega) gene	
rs5903823	X	RP11-453F18__B.1	Clone-based (Vega) gene	
rs6637904	X	RP11-453F18__B.1	Clone-based (Vega) gene	
rs143962491				
rs5977568				
rs5975301				
rs5930521				
rs73638546				

SNP	Chromosome	gene name	gene source	description
rs73565957				
rs5977585				
rs17316792				
rs73237328				
rs202746				
rs113919685				
rs5930534				
rs113706382				
rs2180237				
rs12847569				
rs62619090	X	MST4	UniProtKB Gene Name	Serine/threonine-protein kinase MST4 [Source:UniProtKB/Swiss-Prot;Acc:Q9P289]
rs4142509	X	MST4	UniProtKB Gene Name	Serine/threonine-protein kinase MST4 [Source:UniProtKB/Swiss-Prot;Acc:Q9P289]
rs148067211	X	MST4	UniProtKB Gene Name	Serine/threonine-protein kinase MST4 [Source:UniProtKB/Swiss-Prot;Acc:Q9P289]
rs58519314	X	MST4	UniProtKB Gene Name	Serine/threonine-protein kinase MST4 [Source:UniProtKB/Swiss-Prot;Acc:Q9P289]
rs17250957	X	MST4	UniProtKB Gene Name	Serine/threonine-protein kinase MST4 [Source:UniProtKB/Swiss-Prot;Acc:Q9P289]
rs12858569	X	MST4	UniProtKB Gene Name	Serine/threonine-protein kinase MST4 [Source:UniProtKB/Swiss-Prot;Acc:Q9P289]
rs45471491	X	MST4	UniProtKB Gene Name	Serine/threonine-protein kinase MST4 [Source:UniProtKB/Swiss-Prot;Acc:Q9P289]
rs5977623	X	FRMD7	HGNC Symbol	FERM domain containing 7 [Source:HGNC Symbol;Acc:8079]
rs6637934	X	FRMD7	HGNC Symbol	FERM domain containing 7 [Source:HGNC Symbol;Acc:8079]
rs5977625	X	FRMD7	HGNC Symbol	FERM domain containing 7 [Source:HGNC Symbol;Acc:8079]
rs139820962	X	FRMD7	HGNC Symbol	FERM domain containing 7 [Source:HGNC Symbol;Acc:8079]
rs17000937	X	FRMD7	HGNC Symbol	FERM domain containing 7 [Source:HGNC Symbol;Acc:8079]
rs6637941	X	FRMD7	HGNC Symbol	FERM domain containing 7 [Source:HGNC Symbol;Acc:8079]
rs62619094	X	FRMD7	HGNC Symbol	FERM domain containing 7 [Source:HGNC Symbol;Acc:8079]
rs5933080	X	FRMD7	HGNC Symbol	FERM domain containing 7 [Source:HGNC Symbol;Acc:8079]
rs5933109				
rs143853322	X	RAP2C	HGNC Symbol	RAP2C, member of RAS oncogene family [Source:HGNC Symbol;Acc:21165]
rs41304510	X	RAP2C	HGNC Symbol	RAP2C, member of RAS oncogene family [Source:HGNC Symbol;Acc:21165]

SNP	Chromosome	gene name	gene source	description
rs5977658	X	RAP2C	HGNC Symbol	RAP2C, member of RAS oncogene family [Source:HGNC Symbol;Acc:21165]
rs5930561	X	RAP2C-AS1	HGNC Symbol	RAP2C antisense RNA 1 [Source:HGNC Symbol;Acc:40957]
rs12843746	X	RAP2C-AS1	HGNC Symbol	RAP2C antisense RNA 1 [Source:HGNC Symbol;Acc:40957]
rs144412136	X	RAP2C-AS1	HGNC Symbol	RAP2C antisense RNA 1 [Source:HGNC Symbol;Acc:40957]
rs6637988	X	RAP2C-AS1	HGNC Symbol	RAP2C antisense RNA 1 [Source:HGNC Symbol;Acc:40957]
rs56220643	X	RAP2C-AS1	HGNC Symbol	RAP2C antisense RNA 1 [Source:HGNC Symbol;Acc:40957]
rs62617185	X	MBNL3	HGNC Symbol	muscleblind-like splicing regulator 3 [Source:HGNC Symbol;Acc:20564]
rs149935657				
rs73237367				
rs2583496				
rs2089871				
rs12558802	X	HS6ST2	HGNC Symbol	heparan sulfate 6-O-sulfotransferase 2 [Source:HGNC Symbol;Acc:19133]
rs17324091	X	HS6ST2	HGNC Symbol	heparan sulfate 6-O-sulfotransferase 2 [Source:HGNC Symbol;Acc:19133]
rs243439	X	HS6ST2	HGNC Symbol	heparan sulfate 6-O-sulfotransferase 2 [Source:HGNC Symbol;Acc:19133]
rs5930575	X	HS6ST2	HGNC Symbol	heparan sulfate 6-O-sulfotransferase 2 [Source:HGNC Symbol;Acc:19133]
rs139228627	X	HS6ST2	HGNC Symbol	heparan sulfate 6-O-sulfotransferase 2 [Source:HGNC Symbol;Acc:19133]
rs17380	X	HS6ST2	HGNC Symbol	heparan sulfate 6-O-sulfotransferase 2 [Source:HGNC Symbol;Acc:19133]
rs17251211	X	HS6ST2	HGNC Symbol	heparan sulfate 6-O-sulfotransferase 2 [Source:HGNC Symbol;Acc:19133]
rs111316374	X	HS6ST2	HGNC Symbol	heparan sulfate 6-O-sulfotransferase 2 [Source:HGNC Symbol;Acc:19133]
rs5977737	X	HS6ST2	HGNC Symbol	heparan sulfate 6-O-sulfotransferase 2 [Source:HGNC Symbol;Acc:19133]
rs113417453	X	HS6ST2	HGNC Symbol	heparan sulfate 6-O-sulfotransferase 2 [Source:HGNC Symbol;Acc:19133]
rs73638579	X	HS6ST2	HGNC Symbol	heparan sulfate 6-O-sulfotransferase 2 [Source:HGNC Symbol;Acc:19133]
rs5977760	X	HS6ST2	HGNC Symbol	heparan sulfate 6-O-sulfotransferase 2 [Source:HGNC Symbol;Acc:19133]
rs12840953	X	HS6ST2	HGNC Symbol	heparan sulfate 6-O-sulfotransferase 2 [Source:HGNC Symbol;Acc:19133]
rs144346774	X	HS6ST2	HGNC Symbol	heparan sulfate 6-O-sulfotransferase 2 [Source:HGNC Symbol;Acc:19133]
rs113350037	X	HS6ST2	HGNC Symbol	heparan sulfate 6-O-sulfotransferase 2 [Source:HGNC Symbol;Acc:19133]
rs5977769	X	HS6ST2	HGNC Symbol	heparan sulfate 6-O-sulfotransferase 2 [Source:HGNC Symbol;Acc:19133]
rs57066725	X	HS6ST2	HGNC Symbol	heparan sulfate 6-O-sulfotransferase 2 [Source:HGNC Symbol;Acc:19133]
rs34694310	X	HS6ST2	HGNC Symbol	heparan sulfate 6-O-sulfotransferase 2 [Source:HGNC Symbol;Acc:19133]
rs140041122	X	HS6ST2	HGNC Symbol	heparan sulfate 6-O-sulfotransferase 2 [Source:HGNC Symbol;Acc:19133]
rs146450227				
rs2872900				
rs17317259				

SNP	Chromosome	gene name	gene source	description
rs6638047				
rs41299088	X	USP26	HGNC Symbol	ubiquitin specific peptidase 26 [Source:HGNC Symbol;Acc:13485]
rs35397110	X	USP26	HGNC Symbol	ubiquitin specific peptidase 26 [Source:HGNC Symbol;Acc:13485]
rs41304540	X	USP26	HGNC Symbol	ubiquitin specific peptidase 26 [Source:HGNC Symbol;Acc:13485]
rs61741870	X	USP26	HGNC Symbol	ubiquitin specific peptidase 26 [Source:HGNC Symbol;Acc:13485]
rs1503894	X	USP26	HGNC Symbol	ubiquitin specific peptidase 26 [Source:HGNC Symbol;Acc:13485]
rs173343	X	USP26	HGNC Symbol	ubiquitin specific peptidase 26 [Source:HGNC Symbol;Acc:13485]
rs17305684				
rs112373309				
rs56188189	X	RP3-358H7.1	Clone-based (Vega) gene	
rs5933276				
rs11798041				
rs181447				
rs242160				
rs73239366				
rs6634921				
rs11796817				
rs5977837				
rs7892070				
rs1048369	X	GPC4	HGNC Symbol	glypican 4 [Source:HGNC Symbol;Acc:4452]
rs1129980	X	GPC4	HGNC Symbol	glypican 4 [Source:HGNC Symbol;Acc:4452]
rs2266806	X	GPC4	HGNC Symbol	glypican 4 [Source:HGNC Symbol;Acc:4452]
rs139420537	X	GPC4	HGNC Symbol	glypican 4 [Source:HGNC Symbol;Acc:4452]
rs112064319	X	GPC4	HGNC Symbol	glypican 4 [Source:HGNC Symbol;Acc:4452]
rs73239387	X	GPC4	HGNC Symbol	glypican 4 [Source:HGNC Symbol;Acc:4452]
rs41490249				
rs17000449				
rs35307515				
rs5933308				
rs57061145				
rs111954829				
rs12844160				

SNP	Chromosome	gene name	gene source	description
rs56249937	X	GPC3	HGNC Symbol	glypican 3 [Source:HGNC Symbol;Acc:4451]
rs17317371	X	GPC3	HGNC Symbol	glypican 3 [Source:HGNC Symbol;Acc:4451]
rs17277770	X	GPC3	HGNC Symbol	glypican 3 [Source:HGNC Symbol;Acc:4451]
rs17251489	X	GPC3	HGNC Symbol	glypican 3 [Source:HGNC Symbol;Acc:4451]
rs12862989	X	GPC3	HGNC Symbol	glypican 3 [Source:HGNC Symbol;Acc:4451]
rs879941	X	GPC3	HGNC Symbol	glypican 3 [Source:HGNC Symbol;Acc:4451]
rs17251516	X	GPC3	HGNC Symbol	glypican 3 [Source:HGNC Symbol;Acc:4451]
rs3831708	X	GPC3	HGNC Symbol	glypican 3 [Source:HGNC Symbol;Acc:4451]
rs73239402	X	GPC3	HGNC Symbol	glypican 3 [Source:HGNC Symbol;Acc:4451]
rs73241304	X	GPC3	HGNC Symbol	glypican 3 [Source:HGNC Symbol;Acc:4451]
rs12558298	X	GPC3	HGNC Symbol	glypican 3 [Source:HGNC Symbol;Acc:4451]
rs764519	X	GPC3	HGNC Symbol	glypican 3 [Source:HGNC Symbol;Acc:4451]
rs4829762	X	GPC3	HGNC Symbol	glypican 3 [Source:HGNC Symbol;Acc:4451]
rs7060636	X	GPC3	HGNC Symbol	glypican 3 [Source:HGNC Symbol;Acc:4451]
rs12860457	X	GPC3	HGNC Symbol	glypican 3 [Source:HGNC Symbol;Acc:4451]
rs2267511	X	GPC3	HGNC Symbol	glypican 3 [Source:HGNC Symbol;Acc:4451]
rs2267516	X	GPC3	HGNC Symbol	glypican 3 [Source:HGNC Symbol;Acc:4451]
rs73632100	X	GPC3	HGNC Symbol	glypican 3 [Source:HGNC Symbol;Acc:4451]
rs11797521	X	GPC3	HGNC Symbol	glypican 3 [Source:HGNC Symbol;Acc:4451]
rs147826244	X	GPC3	HGNC Symbol	glypican 3 [Source:HGNC Symbol;Acc:4451]
rs5977928	X	GPC3	HGNC Symbol	glypican 3 [Source:HGNC Symbol;Acc:4451]
rs144385017	X	GPC3	HGNC Symbol	glypican 3 [Source:HGNC Symbol;Acc:4451]
rs6654278	X	GPC3	HGNC Symbol	glypican 3 [Source:HGNC Symbol;Acc:4451]
rs73241312	X	GPC3	HGNC Symbol	glypican 3 [Source:HGNC Symbol;Acc:4451]
rs138697029	X	AF003529.2	Clone-based (Vega) gene	
rs17324524				
rs149561060				
rs11796475				
rs11798675				
rs6638162				
rs73241317				
rs61344208				

SNP	Chromosome	gene name	gene source	description
rs55971319				
rs62599382				
rs5977968				
rs62599383				
rs138454883				
rs12850804				
rs5977998				
rs59386608				
rs112967805				
rs5933405				
rs73241337	X	PHF6	HGNC Symbol	PHD finger protein 6 [Source:HGNC Symbol;Acc:18145]
rs6529626	X	PHF6	HGNC Symbol	PHD finger protein 6 [Source:HGNC Symbol;Acc:18145]
rs5933410	X	PHF6	HGNC Symbol	PHD finger protein 6 [Source:HGNC Symbol;Acc:18145]
rs4830285	X	PHF6	HGNC Symbol	PHD finger protein 6 [Source:HGNC Symbol;Acc:18145]
rs6638230	X	PHF6	HGNC Symbol	PHD finger protein 6 [Source:HGNC Symbol;Acc:18145]
rs2097778				
rs12558462				
rs4829768				
rs17885363	X	HPRT1	HGNC Symbol	hypoxanthine phosphoribosyltransferase 1 [Source:HGNC Symbol;Acc:5157]
rs73241339	X	HPRT1	HGNC Symbol	hypoxanthine phosphoribosyltransferase 1 [Source:HGNC Symbol;Acc:5157]
rs2428561				
rs757308				
rs5933419				
rs2503984	X	MIR503HG	HGNC Symbol	MIR503 host gene (non-protein coding) [Source:HGNC Symbol;Acc:28258]
rs62599410	X	LINC00629	HGNC Symbol	long intergenic non-protein coding RNA 629 [Source:HGNC Symbol;Acc:44262]
rs5933425	X	PLAC1	HGNC Symbol	placenta-specific 1 [Source:HGNC Symbol;Acc:9044]
rs73224744	X	PLAC1	HGNC Symbol	placenta-specific 1 [Source:HGNC Symbol;Acc:9044]
rs4830296	X	PLAC1	HGNC Symbol	placenta-specific 1 [Source:HGNC Symbol;Acc:9044]
rs17317793	X	PLAC1	HGNC Symbol	placenta-specific 1 [Source:HGNC Symbol;Acc:9044]
rs6635005	X	PLAC1	HGNC Symbol	placenta-specific 1 [Source:HGNC Symbol;Acc:9044]
rs5933433	X	PLAC1	HGNC Symbol	placenta-specific 1 [Source:HGNC Symbol;Acc:9044]
rs4345730	X	PLAC1;RP11-308B5.2	HGNC Symbol;Clone-based (Vega) gene	placenta-specific 1 [Source:HGNC Symbol;Acc:9044];

SNP	Chromosome	gene name	gene source	description
rs41300299	X	PLAC1;RP11-308B5.2	HGNC Symbol;Clone-based (Vega) gene	placenta-specific 1 [Source:HGNC Symbol;Acc:9044];
rs35026167	X	PLAC1	HGNC Symbol	placenta-specific 1 [Source:HGNC Symbol;Acc:9044]
rs12557773	X	PLAC1	HGNC Symbol	placenta-specific 1 [Source:HGNC Symbol;Acc:9044]
rs5930654	X	PLAC1	HGNC Symbol	placenta-specific 1 [Source:HGNC Symbol;Acc:9044]
rs41300910	X	PLAC1	HGNC Symbol	placenta-specific 1 [Source:HGNC Symbol;Acc:9044]
rs56164326	X	PLAC1	HGNC Symbol	placenta-specific 1 [Source:HGNC Symbol;Acc:9044]
rs11096402	X	PLAC1	HGNC Symbol	placenta-specific 1 [Source:HGNC Symbol;Acc:9044]
rs5978037	X	PLAC1	HGNC Symbol	placenta-specific 1 [Source:HGNC Symbol;Acc:9044]
rs7881280	X	PLAC1	HGNC Symbol	placenta-specific 1 [Source:HGNC Symbol;Acc:9044]
rs7056552	X	PLAC1	HGNC Symbol	placenta-specific 1 [Source:HGNC Symbol;Acc:9044]
rs12858878	X	PLAC1	HGNC Symbol	placenta-specific 1 [Source:HGNC Symbol;Acc:9044]
rs4529619	X	PLAC1	HGNC Symbol	placenta-specific 1 [Source:HGNC Symbol;Acc:9044]
rs66764879	X	PLAC1	HGNC Symbol	placenta-specific 1 [Source:HGNC Symbol;Acc:9044]
rs17317849	X	FAM122B	HGNC Symbol	family with sequence similarity 122B [Source:HGNC Symbol;Acc:30490]
rs147290565	X	FAM122C	HGNC Symbol	family with sequence similarity 122C [Source:HGNC Symbol;Acc:25202]
rs12556133	X	FAM122C	HGNC Symbol	family with sequence similarity 122C [Source:HGNC Symbol;Acc:25202]
rs45447592	X	FAM122C	HGNC Symbol	family with sequence similarity 122C [Source:HGNC Symbol;Acc:25202]
rs7058561	X	FAM122C	HGNC Symbol	family with sequence similarity 122C [Source:HGNC Symbol;Acc:25202]
rs5975480	X	FAM122C	HGNC Symbol	family with sequence similarity 122C [Source:HGNC Symbol;Acc:25202]
rs5930665				
rs73224765				
rs35503740				
rs5978053				
rs56973001				
rs6529647	X	MOSPD1	HGNC Symbol	motile sperm domain containing 1 [Source:HGNC Symbol;Acc:25235]
rs1984391	X	MOSPD1	HGNC Symbol	motile sperm domain containing 1 [Source:HGNC Symbol;Acc:25235]
rs5978059	X	MOSPD1	HGNC Symbol	motile sperm domain containing 1 [Source:HGNC Symbol;Acc:25235]
rs12863494	X	MOSPD1	HGNC Symbol	motile sperm domain containing 1 [Source:HGNC Symbol;Acc:25235]
rs5978060	X	MOSPD1	HGNC Symbol	motile sperm domain containing 1 [Source:HGNC Symbol;Acc:25235]
rs4830306				
rs6638305				
rs5978064				

SNP	Chromosome	gene name	gene source	description
rs72615445				
rs5930669				
rs137986605				
rs4830314				
rs7892173				
rs4829770	X	RP3-473B4.3	Clone-based (Vega) gene	
rs5933484				
rs17278226				
rs2097402				
rs2498771				
rs12856424				
rs146074260				
rs151222539				
rs144063645	X	RP11-85L21.4	Clone-based (Vega) gene	
rs12849383	X	RP11-85L21.4	Clone-based (Vega) gene	
rs5933501	X;HG1443_HG1444_PATCH	RP11-85L21.4	Clone-based (Vega) gene	
rs149875145	X;HG1443_HG1444_PATCH	RP11-85L21.4	Clone-based (Vega) gene	
rs150781289	X	CT55	HGNC Symbol	cancer/testis antigen 55 [Source:HGNC Symbol;Acc:26047]
rs6635037	X	CT55	HGNC Symbol	cancer/testis antigen 55 [Source:HGNC Symbol;Acc:26047]
rs7882782				
rs5978106	X;HG1443_HG1444_PATCH	RP13-210D15.1	Clone-based (Vega) gene	
rs1129095	X;HG1443_HG1444_PATCH	ZNF75D	HGNC Symbol	zinc finger protein 75D [Source:HGNC Symbol;Acc:13145]
rs1129093	X;HG1443_HG1444_PATCH	ZNF75D	HGNC Symbol	zinc finger protein 75D [Source:HGNC Symbol;Acc:13145]
rs62599926	X;HG1443_HG1444_PATCH	ZNF75D	HGNC Symbol	zinc finger protein 75D [Source:HGNC Symbol;Acc:13145]
rs5929652	X;HG1443_HG1444_PATCH	ZNF75D	HGNC Symbol	zinc finger protein 75D [Source:HGNC Symbol;Acc:13145]
rs146228655	X;HG1443_HG1444_PATCH	ZNF75D	HGNC Symbol	zinc finger protein 75D [Source:HGNC Symbol;Acc:13145]

SNP	Chromosome	gene name	gene source	description
rs12837419	X;HG1443_HG1444_PATCH	ZNF75D	HGNC Symbol	zinc finger protein 75D [Source:HGNC Symbol;Acc:13145]
rs1419828	X;HG1443_HG1444_PATCH	ZNF75D	HGNC Symbol	zinc finger protein 75D [Source:HGNC Symbol;Acc:13145]
rs45461393	X;HG1443_HG1444_PATCH	ZNF449	HGNC Symbol	zinc finger protein 449 [Source:HGNC Symbol;Acc:21039]
rs147968032				
rs5975507				
rs138744218				
rs2531377				
rs5930699				
rs5975524				
rs5930723				
rs150584613				
rs17283962				
rs137874043				
rs5974532				
rs5930725				
rs5929661	X;HG1443_HG1444_PATCH	DDX26B	HGNC Symbol	DEAD/H (Asp-Glu-Ala-Asp/His) box polypeptide 26B [Source:HGNC Symbol;Acc:27334]
rs73224797	X;HG1443_HG1444_PATCH	DDX26B	HGNC Symbol	DEAD/H (Asp-Glu-Ala-Asp/His) box polypeptide 26B [Source:HGNC Symbol;Acc:27334]
rs17330752	X;HG1443_HG1444_PATCH	DDX26B	HGNC Symbol	DEAD/H (Asp-Glu-Ala-Asp/His) box polypeptide 26B [Source:HGNC Symbol;Acc:27334]
rs148519767	X	DDX26B	HGNC Symbol	DEAD/H (Asp-Glu-Ala-Asp/His) box polypeptide 26B [Source:HGNC Symbol;Acc:27334]
rs2298302	X	DDX26B	HGNC Symbol	DEAD/H (Asp-Glu-Ala-Asp/His) box polypeptide 26B [Source:HGNC Symbol;Acc:27334]
rs41312590	X	DDX26B	HGNC Symbol	DEAD/H (Asp-Glu-Ala-Asp/His) box polypeptide 26B [Source:HGNC Symbol;Acc:27334]
rs11797927				
rs5930777				
rs12859638				
rs138460865				
rs5974540				
rs180796572				
rs139701979	X	RP11-432N13.4	Clone-based (Vega) gene	

SNP	Chromosome	gene name	gene source	description
rs2501483				
rs28507277				
rs6635091				
rs139976494				
rs2446132				
rs7474320	X;HG1443_HG1444_PATCH	SAGE1	HGNC Symbol	sarcoma antigen 1 [Source:HGNC Symbol;Acc:30369]
rs41301507				
rs4829799	X;HG1443_HG1444_PATCH	SAGE1	HGNC Symbol	sarcoma antigen 1 [Source:HGNC Symbol;Acc:30369]
rs41302146	X;HG1443_HG1444_PATCH	SAGE1	HGNC Symbol	sarcoma antigen 1 [Source:HGNC Symbol;Acc:30369]
rs28766587				
rs28523472				
rs140864304				
rs5930820				
rs61311022				
rs55962438				
rs6635201	X;HG1443_HG1444_PATCH	MMGT1	HGNC Symbol	membrane magnesium transporter 1 [Source:HGNC Symbol;Acc:28100]
rs56165875				
rs5930843	X;HG1443_HG1444_PATCH	SLC9A6	HGNC Symbol	solute carrier family 9, subfamily A (NHE6, cation proton antiporter 6), member 6 [Source:HGNC Symbol;Acc:11079]
rs73226741	X;HG1443_HG1444_PATCH	SLC9A6	HGNC Symbol	solute carrier family 9, subfamily A (NHE6, cation proton antiporter 6), member 6 [Source:HGNC Symbol;Acc:11079]
rs1048463	X;HG1443_HG1444_PATCH	SLC9A6	HGNC Symbol	solute carrier family 9, subfamily A (NHE6, cation proton antiporter 6), member 6 [Source:HGNC Symbol;Acc:11079]
rs55915620				
rs5930846				
rs28475536				
rs6528335				
rs5975665				
rs142180666				
rs5975675				
rs140614175				

SNP	Chromosome	gene name	gene source	description
rs73240340				
rs7471253				
rs145613446				
rs5975687				
rs7057230				
rs190586548				
rs72615473	X	FHL1	HGNC Symbol	four and a half LIM domains 1 [Source:HGNC Symbol;Acc:3702]
rs5930890	X	FHL1	HGNC Symbol	four and a half LIM domains 1 [Source:HGNC Symbol;Acc:3702]
rs3753173	X	FHL1	HGNC Symbol	four and a half LIM domains 1 [Source:HGNC Symbol;Acc:3702]
rs140163950	X	FHL1	HGNC Symbol	four and a half LIM domains 1 [Source:HGNC Symbol;Acc:3702]
rs5975695	X	FHL1	HGNC Symbol	four and a half LIM domains 1 [Source:HGNC Symbol;Acc:3702]
rs2024704	X	FHL1	HGNC Symbol	four and a half LIM domains 1 [Source:HGNC Symbol;Acc:3702]
rs9018	X	FHL1	HGNC Symbol	four and a half LIM domains 1 [Source:HGNC Symbol;Acc:3702]
rs2273221	X	MAP7D3	HGNC Symbol	MAP7 domain containing 3 [Source:HGNC Symbol;Acc:25742]
rs1055497	X	MAP7D3	HGNC Symbol	MAP7 domain containing 3 [Source:HGNC Symbol;Acc:25742]
rs5930917				
rs137878609				
rs2073236	X	RP11-535K18.2	Clone-based (Vega) gene	
rs79114823	X	GPR112	HGNC Symbol	G protein-coupled receptor 112 [Source:HGNC Symbol;Acc:18992]
rs4829829	X	GPR112	HGNC Symbol	G protein-coupled receptor 112 [Source:HGNC Symbol;Acc:18992]
rs5930931	X	GPR112	HGNC Symbol	G protein-coupled receptor 112 [Source:HGNC Symbol;Acc:18992]
rs4829830	X	GPR112	HGNC Symbol	G protein-coupled receptor 112 [Source:HGNC Symbol;Acc:18992]
rs877761	X	GPR112	HGNC Symbol	G protein-coupled receptor 112 [Source:HGNC Symbol;Acc:18992]
rs912002	X	GPR112	HGNC Symbol	G protein-coupled receptor 112 [Source:HGNC Symbol;Acc:18992]
rs111880833	X	GPR112	HGNC Symbol	G protein-coupled receptor 112 [Source:HGNC Symbol;Acc:18992]
rs5930932	X	GPR112	HGNC Symbol	G protein-coupled receptor 112 [Source:HGNC Symbol;Acc:18992]
rs5930933	X	GPR112	HGNC Symbol	G protein-coupled receptor 112 [Source:HGNC Symbol;Acc:18992]
rs716148	X	GPR112	HGNC Symbol	G protein-coupled receptor 112 [Source:HGNC Symbol;Acc:18992]
rs56053841	X	GPR112	HGNC Symbol	G protein-coupled receptor 112 [Source:HGNC Symbol;Acc:18992]
rs1329546	X	GPR112	HGNC Symbol	G protein-coupled receptor 112 [Source:HGNC Symbol;Acc:18992]
rs5974595	X	GPR112	HGNC Symbol	G protein-coupled receptor 112 [Source:HGNC Symbol;Acc:18992]
rs11797633	X	GPR112	HGNC Symbol	G protein-coupled receptor 112 [Source:HGNC Symbol;Acc:18992]

SNP	Chromosome	gene name	gene source	description
rs4829596	X	GPR112	HGNC Symbol	G protein-coupled receptor 112 [Source:HGNC Symbol;Acc:18992]
rs5930942	X	GPR112	HGNC Symbol	G protein-coupled receptor 112 [Source:HGNC Symbol;Acc:18992]
rs62606002	X	GPR112	HGNC Symbol	G protein-coupled receptor 112 [Source:HGNC Symbol;Acc:18992]
rs17284053	X	GPR112	HGNC Symbol	G protein-coupled receptor 112 [Source:HGNC Symbol;Acc:18992]
rs1329549	X	GPR112	HGNC Symbol	G protein-coupled receptor 112 [Source:HGNC Symbol;Acc:18992]
rs3027827				
rs72615477				
rs3027849	X	HTATSF1	HGNC Symbol	HIV-1 Tat specific factor 1 [Source:HGNC Symbol;Acc:5276]
rs17002661				
rs5975742				
rs3027859	X	VGLL1	HGNC Symbol	vestigial like 1 (Drosophila) [Source:HGNC Symbol;Acc:20985]
rs5975745	X	VGLL1	HGNC Symbol	vestigial like 1 (Drosophila) [Source:HGNC Symbol;Acc:20985]
rs67400413	X	VGLL1	HGNC Symbol	vestigial like 1 (Drosophila) [Source:HGNC Symbol;Acc:20985]
rs17330843	X	VGLL1	HGNC Symbol	vestigial like 1 (Drosophila) [Source:HGNC Symbol;Acc:20985]
rs9119	X	VGLL1	HGNC Symbol	vestigial like 1 (Drosophila) [Source:HGNC Symbol;Acc:20985]
rs2427870				
rs2518884				
rs2518903				
rs2157739				
rs5930970				
rs975379	X	LINC00892	HGNC Symbol	long intergenic non-protein coding RNA 892 [Source:HGNC Symbol;Acc:48578]
rs3092949				
rs1126535	X	CD40LG	HGNC Symbol	CD40 ligand [Source:HGNC Symbol;Acc:11935]
rs5930973	X	CD40LG	HGNC Symbol	CD40 ligand [Source:HGNC Symbol;Acc:11935]
rs3092935	X	CD40LG	HGNC Symbol	CD40 ligand [Source:HGNC Symbol;Acc:11935]
rs12559068	X	ARHGEF6	HGNC Symbol	Rac/Cdc42 guanine nucleotide exchange factor (GEF) 6 [Source:HGNC Symbol;Acc:685]
rs55683638	X	ARHGEF6	HGNC Symbol	Rac/Cdc42 guanine nucleotide exchange factor (GEF) 6 [Source:HGNC Symbol;Acc:685]
rs138962037	X	ARHGEF6	HGNC Symbol	Rac/Cdc42 guanine nucleotide exchange factor (GEF) 6 [Source:HGNC Symbol;Acc:685]
rs616718	X	ARHGEF6	HGNC Symbol	Rac/Cdc42 guanine nucleotide exchange factor (GEF) 6 [Source:HGNC Symbol;Acc:685]
rs661426	X	ARHGEF6	HGNC Symbol	Rac/Cdc42 guanine nucleotide exchange factor (GEF) 6 [Source:HGNC Symbol;Acc:685]
rs139890432	X	ARHGEF6	HGNC Symbol	Rac/Cdc42 guanine nucleotide exchange factor (GEF) 6 [Source:HGNC Symbol;Acc:685]
rs12557857				
rs6635326				

SNP	Chromosome	gene name	gene source	description
rs73566916				
rs11798667				
rs73228721				
rs1382533	X	RP11-1114A5.5;RP11-1114A5.4	Clone-based (Vega) gene	
rs17341651	X	RBMX	HGNC Symbol	RNA binding motif protein, X-linked [Source:HGNC Symbol;Acc:9910]
rs146925246				
rs33997936	X	RP11-308D16.4	Clone-based (Vega) gene	
rs5929779	X	RP11-308D16.4	Clone-based (Vega) gene	
rs6633858	X	RP11-308D16.4;RP11-308D16.2	Clone-based (Vega) gene	
rs1190739	X	RP11-308D16.4;RP11-308D16.2	Clone-based (Vega) gene	
rs6635362	X	RP11-308D16.4;RP11-308D16.2	Clone-based (Vega) gene	
rs144729234	X	RP11-308D16.4;RP11-308D16.2	Clone-based (Vega) gene	
rs151028451	X	RP11-308D16.4;RP11-308D16.2	Clone-based (Vega) gene	
rs5931046	X	GPR101	HGNC Symbol	G protein-coupled receptor 101 [Source:HGNC Symbol;Acc:14963]
rs1190736	X	GPR101	HGNC Symbol	G protein-coupled receptor 101 [Source:HGNC Symbol;Acc:14963]
rs5931049				
rs1190734				
rs6633864				
rs7876405				
rs73567961				
rs5929791				
rs5975862				
rs6635369				
rs11797672				
rs5975867				
rs5975868				
rs2151083				
rs10521782				

SNP	Chromosome	gene name	gene source	description
rs5931073				
rs73228739				
rs189470876				
rs5931086				
rs143493958				
rs140367231				
rs5931089				
rs62603370				
rs4829864				
rs57757254				
rs12841694				
rs4263914				
rs6635411				
rs6528444				
rs5929816				
rs11095691				
rs73230519				
rs4269687				
rs79317274				
rs2743903				
rs1024169				
rs143991751				
rs2743914				
rs113485657				
rs1206233				
rs6635441				
rs1206249				
rs12844697				
rs1206223				
rs143037980				
rs55750653				
rs67551497				
rs56230740				

SNP	Chromosome	gene name	gene source	description
rs6635445				
rs2859251				
rs5931172	X	RP1-137H15.2	Clone-based (Vega) gene	
rs143269743	X	RP1-137H15.2	Clone-based (Vega) gene	
rs184632873				
rs7055630	X	ZIC3	HGNC Symbol	Zic family member 3 [Source:HGNC Symbol;Acc:12874]
rs5931176				
rs146887905				
rs4240110	X	ZFYVE9P1	HGNC Symbol	zinc finger, FYVE domain containing 9 pseudogene 1 [Source:HGNC Symbol;Acc:33359]
rs73230559				
rs11798189				
rs17284319				
rs73232778				
rs12688369				
rs7886303				
rs12013075				
rs1408308				
rs143921182				
rs860124				
rs73232795				
rs12851076				
rs2886699				
rs4829622				
rs5931236				
rs5931238				
rs12688840				
rs17331170				
rs5931270				
rs113214614				
rs5976027				
rs4829924				
rs34166214				

SNP	Chromosome	gene name	gene source	description
rs1366934				
rs73234709				
rs17331198				
rs35389436				
rs7056509				
rs452961				
rs5929883				
rs12837096				
rs1594214				
rs34207960				
rs5976047				
rs145042369				
rs4829934				
rs1929364				
rs187214				
rs6635599				
rs73236854				
rs59794052				
rs6635622				
rs1994451				
rs5931380				
rs1891095				
rs7052068				
rs5931395				
rs12843397				
rs1487918				
rs6635684				
rs5931431				
rs73633965				
rs73236875				
rs6654348				
rs138231452				
rs5931457				

SNP	Chromosome	gene name	gene source	description
rs5976162				
rs5931463				
rs2185130				
rs17284572	X	FGF13	HGNC Symbol	fibroblast growth factor 13 [Source:HGNC Symbol;Acc:3670]
rs7052771	X	FGF13	HGNC Symbol	fibroblast growth factor 13 [Source:HGNC Symbol;Acc:3670]
rs147018514	X	FGF13	HGNC Symbol	fibroblast growth factor 13 [Source:HGNC Symbol;Acc:3670]
rs2267628	X	FGF13	HGNC Symbol	fibroblast growth factor 13 [Source:HGNC Symbol;Acc:3670]
rs657	X	FGF13	HGNC Symbol	fibroblast growth factor 13 [Source:HGNC Symbol;Acc:3670]
rs72616246	X	FGF13	HGNC Symbol	fibroblast growth factor 13 [Source:HGNC Symbol;Acc:3670]
rs12557991	X	FGF13	HGNC Symbol	fibroblast growth factor 13 [Source:HGNC Symbol;Acc:3670]
rs17510088	X	FGF13	HGNC Symbol	fibroblast growth factor 13 [Source:HGNC Symbol;Acc:3670]
rs17538809	X	FGF13	HGNC Symbol	fibroblast growth factor 13 [Source:HGNC Symbol;Acc:3670]
rs6528564	X	FGF13	HGNC Symbol	fibroblast growth factor 13 [Source:HGNC Symbol;Acc:3670]
rs2017465	X	FGF13	HGNC Symbol	fibroblast growth factor 13 [Source:HGNC Symbol;Acc:3670]
rs73241070	X	FGF13	HGNC Symbol	fibroblast growth factor 13 [Source:HGNC Symbol;Acc:3670]
rs5929947	X	FGF13	HGNC Symbol	fibroblast growth factor 13 [Source:HGNC Symbol;Acc:3670]
rs5976197	X	FGF13	HGNC Symbol	fibroblast growth factor 13 [Source:HGNC Symbol;Acc:3670]
rs144050803	X	FGF13	HGNC Symbol	fibroblast growth factor 13 [Source:HGNC Symbol;Acc:3670]
rs140131209	X	FGF13	HGNC Symbol	fibroblast growth factor 13 [Source:HGNC Symbol;Acc:3670]
rs34079655	X	FGF13	HGNC Symbol	fibroblast growth factor 13 [Source:HGNC Symbol;Acc:3670]
rs12838463	X	FGF13	HGNC Symbol	fibroblast growth factor 13 [Source:HGNC Symbol;Acc:3670]
rs73241079	X	FGF13	HGNC Symbol	fibroblast growth factor 13 [Source:HGNC Symbol;Acc:3670]
rs7060413	X	FGF13	HGNC Symbol	fibroblast growth factor 13 [Source:HGNC Symbol;Acc:3670]
rs148753164	X	FGF13	HGNC Symbol	fibroblast growth factor 13 [Source:HGNC Symbol;Acc:3670]
rs6654356	X	FGF13	HGNC Symbol	fibroblast growth factor 13 [Source:HGNC Symbol;Acc:3670]
rs56111210	X	FGF13	HGNC Symbol	fibroblast growth factor 13 [Source:HGNC Symbol;Acc:3670]
rs507988	X	FGF13	HGNC Symbol	fibroblast growth factor 13 [Source:HGNC Symbol;Acc:3670]
rs556414	X	FGF13	HGNC Symbol	fibroblast growth factor 13 [Source:HGNC Symbol;Acc:3670]
rs17331373	X	FGF13	HGNC Symbol	fibroblast growth factor 13 [Source:HGNC Symbol;Acc:3670]
rs141792138	X	FGF13	HGNC Symbol	fibroblast growth factor 13 [Source:HGNC Symbol;Acc:3670]
rs619373	X	FGF13	HGNC Symbol	fibroblast growth factor 13 [Source:HGNC Symbol;Acc:3670]
rs479265	X	FGF13	HGNC Symbol	fibroblast growth factor 13 [Source:HGNC Symbol;Acc:3670]
rs540958	X	FGF13	HGNC Symbol	fibroblast growth factor 13 [Source:HGNC Symbol;Acc:3670]

SNP	Chromosome	gene name	gene source	description
rs4829636	X	FGF13	HGNC Symbol	fibroblast growth factor 13 [Source:HGNC Symbol;Acc:3670]
rs2206128	X	FGF13	HGNC Symbol	fibroblast growth factor 13 [Source:HGNC Symbol;Acc:3670]
rs971470	X	FGF13	HGNC Symbol	fibroblast growth factor 13 [Source:HGNC Symbol;Acc:3670]
rs72616259	X	FGF13	HGNC Symbol	fibroblast growth factor 13 [Source:HGNC Symbol;Acc:3670]
rs73243305	X	FGF13	HGNC Symbol	fibroblast growth factor 13 [Source:HGNC Symbol;Acc:3670]
rs512877	X	FGF13	HGNC Symbol	fibroblast growth factor 13 [Source:HGNC Symbol;Acc:3670]
rs10521792	X	FGF13	HGNC Symbol	fibroblast growth factor 13 [Source:HGNC Symbol;Acc:3670]
rs62602205	X	FGF13	HGNC Symbol	fibroblast growth factor 13 [Source:HGNC Symbol;Acc:3670]
rs5931564	X	FGF13	HGNC Symbol	fibroblast growth factor 13 [Source:HGNC Symbol;Acc:3670]
rs144218712	X	FGF13	HGNC Symbol	fibroblast growth factor 13 [Source:HGNC Symbol;Acc:3670]
rs73243310	X	FGF13	HGNC Symbol	fibroblast growth factor 13 [Source:HGNC Symbol;Acc:3670]
rs5976272	X	FGF13	HGNC Symbol	fibroblast growth factor 13 [Source:HGNC Symbol;Acc:3670]
rs5976273	X	FGF13	HGNC Symbol	fibroblast growth factor 13 [Source:HGNC Symbol;Acc:3670]
rs72616266	X	FGF13	HGNC Symbol	fibroblast growth factor 13 [Source:HGNC Symbol;Acc:3670]
rs7882266	X	FGF13	HGNC Symbol	fibroblast growth factor 13 [Source:HGNC Symbol;Acc:3670]
rs62603213	X	FGF13	HGNC Symbol	fibroblast growth factor 13 [Source:HGNC Symbol;Acc:3670]
rs12687894	X	FGF13	HGNC Symbol	fibroblast growth factor 13 [Source:HGNC Symbol;Acc:3670]
rs12387343	X	FGF13	HGNC Symbol	fibroblast growth factor 13 [Source:HGNC Symbol;Acc:3670]
rs73243312	X	FGF13	HGNC Symbol	fibroblast growth factor 13 [Source:HGNC Symbol;Acc:3670]
rs5931572	X	FGF13	HGNC Symbol	fibroblast growth factor 13 [Source:HGNC Symbol;Acc:3670]
rs5976289	X	FGF13	HGNC Symbol	fibroblast growth factor 13 [Source:HGNC Symbol;Acc:3670]
rs2227059	X	FGF13	HGNC Symbol	fibroblast growth factor 13 [Source:HGNC Symbol;Acc:3670]
rs10521795	X	FGF13	HGNC Symbol	fibroblast growth factor 13 [Source:HGNC Symbol;Acc:3670]
rs55913587	X	FGF13	HGNC Symbol	fibroblast growth factor 13 [Source:HGNC Symbol;Acc:3670]
rs7880571	X	FGF13	HGNC Symbol	fibroblast growth factor 13 [Source:HGNC Symbol;Acc:3670]
rs73243317	X	FGF13	HGNC Symbol	fibroblast growth factor 13 [Source:HGNC Symbol;Acc:3670]
rs9988241	X	FGF13	HGNC Symbol	fibroblast growth factor 13 [Source:HGNC Symbol;Acc:3670]
rs10127052	X	FGF13	HGNC Symbol	fibroblast growth factor 13 [Source:HGNC Symbol;Acc:3670]
rs12847459	X	FGF13	HGNC Symbol	fibroblast growth factor 13 [Source:HGNC Symbol;Acc:3670]
rs73634010	X	FGF13	HGNC Symbol	fibroblast growth factor 13 [Source:HGNC Symbol;Acc:3670]
rs1016933	X	FGF13	HGNC Symbol	fibroblast growth factor 13 [Source:HGNC Symbol;Acc:3670]
rs73634017	X	FGF13	HGNC Symbol	fibroblast growth factor 13 [Source:HGNC Symbol;Acc:3670]
rs57429691	X	FGF13	HGNC Symbol	fibroblast growth factor 13 [Source:HGNC Symbol;Acc:3670]

SNP	Chromosome	gene name	gene source	description
rs149854453	X	FGF13	HGNC Symbol	fibroblast growth factor 13 [Source:HGNC Symbol;Acc:3670]
rs12556103				
rs12836794				
rs5974809				
rs6634047				
rs66700094				
rs73243324				
rs606				
rs11798734				
rs73243327				
rs145457335				
rs12842104				
rs56196628				
rs374508				
rs62602891				
rs6528613				
rs4829971				
rs73243337				
rs17284726				
rs10521798				
rs6635815				
rs11797591				
rs35812450				
rs5976357				
rs3971914				
rs4829974				
rs41380647				
rs17002106				
rs67764332				
rs13440893				
rs35893373				
rs1268027				
rs6528615				

SNP	Chromosome	gene name	gene source	description
rs6634066				
rs5931636				
rs12557491				
rs56085107				
rs62611477				
rs58895352				
rs140150717				
rs6634076				
rs145011638				
rs4829994				
rs146513145				
rs5931666				
rs7055668				
rs3817939	X	F9	HGNC Symbol	coagulation factor IX [Source:HGNC Symbol;Acc:3551]
rs371000	X	F9	HGNC Symbol	coagulation factor IX [Source:HGNC Symbol;Acc:3551]
rs392959	X	F9	HGNC Symbol	coagulation factor IX [Source:HGNC Symbol;Acc:3551]
rs4149756	X	F9	HGNC Symbol	coagulation factor IX [Source:HGNC Symbol;Acc:3551]
rs374988	X	F9	HGNC Symbol	coagulation factor IX [Source:HGNC Symbol;Acc:3551]
rs6048	X	F9	HGNC Symbol	coagulation factor IX [Source:HGNC Symbol;Acc:3551]
rs4149759	X	F9	HGNC Symbol	coagulation factor IX [Source:HGNC Symbol;Acc:3551]
rs4149730	X	F9	HGNC Symbol	coagulation factor IX [Source:HGNC Symbol;Acc:3551]
rs434447				
rs17340148				
rs4825206				
rs5908942				
rs1051619	X	MCF2	HGNC Symbol	MCF.2 cell line derived transforming sequence [Source:HGNC Symbol;Acc:6940]
rs61751333	X	MCF2	HGNC Symbol	MCF.2 cell line derived transforming sequence [Source:HGNC Symbol;Acc:6940]
rs10521800	X	MCF2	HGNC Symbol	MCF.2 cell line derived transforming sequence [Source:HGNC Symbol;Acc:6940]
rs5907604	X	MCF2	HGNC Symbol	MCF.2 cell line derived transforming sequence [Source:HGNC Symbol;Acc:6940]
rs12013923	X	MCF2	HGNC Symbol	MCF.2 cell line derived transforming sequence [Source:HGNC Symbol;Acc:6940]
rs10521801	X	MCF2	HGNC Symbol	MCF.2 cell line derived transforming sequence [Source:HGNC Symbol;Acc:6940]
rs12556003	X	MCF2	HGNC Symbol	MCF.2 cell line derived transforming sequence [Source:HGNC Symbol;Acc:6940]
rs978818	X	MCF2	HGNC Symbol	MCF.2 cell line derived transforming sequence [Source:HGNC Symbol;Acc:6940]

SNP	Chromosome	gene name	gene source	description
rs2772185	X	MCF2	HGNC Symbol	MCF.2 cell line derived transforming sequence [Source:HGNC Symbol;Acc:6940]
rs2805899				
rs7061584	X	ATP11C	HGNC Symbol	ATPase, class VI, type 11C [Source:HGNC Symbol;Acc:13554]
rs12687833	X	ATP11C	HGNC Symbol	ATPase, class VI, type 11C [Source:HGNC Symbol;Acc:13554]
rs45465799	X	ATP11C	HGNC Symbol	ATPase, class VI, type 11C [Source:HGNC Symbol;Acc:13554]
rs62609091	X	ATP11C	HGNC Symbol	ATPase, class VI, type 11C [Source:HGNC Symbol;Acc:13554]
rs2485724	X	ATP11C;RNU6ATAC23P	HGNC Symbol	ATPase, class VI, type 11C [Source:HGNC Symbol;Acc:13554];RNA, U6atac small nuclear 23, pseudogene [Source:HGNC Symbol;Acc:46922]
rs4631627	X	ATP11C	HGNC Symbol	ATPase, class VI, type 11C [Source:HGNC Symbol;Acc:13554]
rs17256782				
rs5955139	X	CXorf66	HGNC Symbol	chromosome X open reading frame 66 [Source:HGNC Symbol;Acc:33743]
rs73245304				
rs6634105				
rs5953915				
rs6635942				
rs5955267				
rs4354481				
rs73585731				
rs6418824				
rs2496271				
rs2496269				
rs12156768				
rs5955368				
rs5907577				
rs1886366				
rs73230727				
rs5909021				
rs1541375				
rs507077				
rs4825222				
rs73230738				
rs6636011				
rs17322765				

SNP	Chromosome	gene name	gene source	description
rs149060233				
rs440228				
rs402015				
rs63012860				
rs5907587				
rs5955417				
rs6636024				
rs73230751				
rs17322779				
rs5955456				
rs12558530				
rs62609015				
rs144305610				
rs7061468				
rs147682400				
rs67171357				
rs56219564				
rs5909058				
rs73230760				
rs6634164				
rs2340412				
rs5955502				
rs911093				
rs35418001				
rs5907591				
rs5907593				
rs62609040				
rs203635				
rs203644				
rs1407833				
rs203647				
rs72617934				
rs35661031				

SNP	Chromosome	gene name	gene source	description
rs59469630				
rs67401583				
rs5907596				
rs73580439				
rs6418831				
rs60556419				
rs17322856				
rs2340447				
rs6528723				
rs6636082				
rs5954046				
rs6528729				
rs143006078				
rs6528734				
rs12688185				
rs7876656				
rs5954056				
rs112528577				
rs4824847				
rs11796840				
rs73234912				
rs6636093				
rs5954069	X	XXyac-YR29IB3.1	Clone-based (Vega) gene	
rs5954070	X	XXyac-YR29IB3.1	Clone-based (Vega) gene	
rs6636100				
rs4824848				
rs6528750				
rs5954073				
rs5954074				
rs73234918				
rs72617941				
rs5907616	X	LINC00632	HGNC Symbol	long intergenic non-protein coding RNA 632 [Source:HGNC Symbol;Acc:27865]

SNP	Chromosome	gene name	gene source	description
rs41304542	X	LINC00632	HGNC Symbol	long intergenic non-protein coding RNA 632 [Source:HGNC Symbol;Acc:27865]
rs73590124	X	LINC00632	HGNC Symbol	long intergenic non-protein coding RNA 632 [Source:HGNC Symbol;Acc:27865]
rs72617942	X	LINC00632	HGNC Symbol	long intergenic non-protein coding RNA 632 [Source:HGNC Symbol;Acc:27865]
rs12842403	X	LINC00632	HGNC Symbol	long intergenic non-protein coding RNA 632 [Source:HGNC Symbol;Acc:27865]
rs73590145	X	LINC00632	HGNC Symbol	long intergenic non-protein coding RNA 632 [Source:HGNC Symbol;Acc:27865]
rs17002480	X	LINC00632	HGNC Symbol	long intergenic non-protein coding RNA 632 [Source:HGNC Symbol;Acc:27865]
rs2281414	X	LINC00632	HGNC Symbol	long intergenic non-protein coding RNA 632 [Source:HGNC Symbol;Acc:27865]
rs56239823	X	LINC00632	HGNC Symbol	long intergenic non-protein coding RNA 632 [Source:HGNC Symbol;Acc:27865]
rs6636110	X	LINC00632	HGNC Symbol	long intergenic non-protein coding RNA 632 [Source:HGNC Symbol;Acc:27865]
rs5954087	X	LINC00632	HGNC Symbol	long intergenic non-protein coding RNA 632 [Source:HGNC Symbol;Acc:27865]
rs6636113	X	LINC00632	HGNC Symbol	long intergenic non-protein coding RNA 632 [Source:HGNC Symbol;Acc:27865]
rs6636114	X	LINC00632	HGNC Symbol	long intergenic non-protein coding RNA 632 [Source:HGNC Symbol;Acc:27865]
rs62618215	X	LINC00632	HGNC Symbol	long intergenic non-protein coding RNA 632 [Source:HGNC Symbol;Acc:27865]
rs6634194	X	LINC00632	HGNC Symbol	long intergenic non-protein coding RNA 632 [Source:HGNC Symbol;Acc:27865]
rs59262713	X	LINC00632	HGNC Symbol	long intergenic non-protein coding RNA 632 [Source:HGNC Symbol;Acc:27865]
rs5907635	X	LINC00632	HGNC Symbol	long intergenic non-protein coding RNA 632 [Source:HGNC Symbol;Acc:27865]
rs2180065	X	LINC00632	HGNC Symbol	long intergenic non-protein coding RNA 632 [Source:HGNC Symbol;Acc:27865]
rs5907638	X	LINC00632	HGNC Symbol	long intergenic non-protein coding RNA 632 [Source:HGNC Symbol;Acc:27865]
rs6528754				
rs7885095				
rs5907055				
rs41299075	X	CDR1	HGNC Symbol	cerebellar degeneration-related protein 1, 34kDa [Source:HGNC Symbol;Acc:1798]
rs5954093				
rs12013481				
rs142456265				
rs72617949				
rs5907066				
rs12556960				
rs57435667				
rs7886564				
rs56010449				
rs41381447				
rs2873231				

SNP	Chromosome	gene name	gene source	description
rs5907074				
rs1417426				
rs17002513				
rs1934831				
rs2144283				
rs5907078				
rs7879822				
rs1417427				
rs5954118				
rs6528768				
rs4824853				
rs5954121				
rs5907702				
rs6636129				
rs17002540				
rs7882637				
rs112153932				
rs62620091				
rs41327547				
rs7065549				
rs954054				
rs6636137				
rs6528776				
rs6636138				
rs7888189				
rs5907086				
rs5907721				
rs5907722				
rs112509294				
rs11095843				
rs73586564				
rs138364887				
rs6528780				

SNP	Chromosome	gene name	gene source	description
rs5907726				
rs12008676				
rs5907727				
rs12557289				
rs1908319				
rs5907733				
rs5907091				
rs11095846				
rs12557149				
rs7886651				
rs12387620				
rs5907736	X;HG1453_PATCH	RP11-298A8.2	Clone-based (Vega) gene	
rs73236639	X;HG1453_PATCH	RP11-298A8.2	Clone-based (Vega) gene	
rs9969924	X;HG1453_PATCH	RP11-298A8.2	Clone-based (Vega) gene	
rs6636151	X;HG1453_PATCH	RP11-298A8.2	Clone-based (Vega) gene	
rs12014462	X;HG1453_PATCH	RP11-298A8.2	Clone-based (Vega) gene	
rs769291	X;HG1453_PATCH	RP11-298A8.2	Clone-based (Vega) gene	
rs73236648	X;HG1453_PATCH	RP11-298A8.2	Clone-based (Vega) gene	
rs12689457	X;HG1453_PATCH	RP11-298A8.2	Clone-based (Vega) gene	
rs6634214	X;HG1453_PATCH	RP11-298A8.2	Clone-based (Vega) gene	
rs73236651	X;HG1453_PATCH	RP11-298A8.2	Clone-based (Vega) gene	
rs73588426	X;HG1453_PATCH	RP11-298A8.2	Clone-based (Vega) gene	
rs1004425	X;HG1453_PATCH	RP11-298A8.2	Clone-based (Vega) gene	
rs7059419	X;HG1453_PATCH	RP11-298A8.2	Clone-based (Vega) gene	

SNP	Chromosome	gene name	gene source	description
rs11798510	X;HG1453_PATCH	RP11-298A8.2	Clone-based (Vega) gene	
rs1565844	X;HG1453_PATCH	RP11-298A8.2	Clone-based (Vega) gene	
rs6636165	X;HG1453_PATCH	RP11-298A8.2	Clone-based (Vega) gene	
rs5953546	X;HG1453_PATCH	RP11-298A8.2	Clone-based (Vega) gene	
rs17323017	X;HG1453_PATCH	RP11-298A8.2	Clone-based (Vega) gene	
rs1018818				
rs12007771				
rs5953564				
rs4824991				
rs67180776				
rs12393198				
rs6636233				
rs5954226				
rs142932231				
rs5954246				
rs73594160				
rs12843245				
rs56271894				
rs5953581				
rs5907859				
rs139639813				
rs845164				
rs845165				
rs16979074				
rs5953587				
rs845186				
rs1493189				
rs7878438				
rs5954267				
rs5954272				

SNP	Chromosome	gene name	gene source	description
rs2029673				
rs5907131				
rs4825002				
rs6528829				
rs12557948				
rs844969				
rs844971				
rs146681286				
rs844965				
rs844956				
rs926809				
rs6636278				
rs2864953				
rs5907910				
rs6636295				
rs6528851				
rs5907150				
rs5953602				
rs6636315				
rs5907931				
rs17282467				
rs6636332				
rs4824873				
rs6528868				
rs5907949				
rs6528869				
rs7063314				
rs7879609				
rs5907955				
rs7891457	X	SPANXA2-OT1	HGNC Symbol	SPANXA2 overlapping transcript 1 (non-protein coding) [Source:HGNC Symbol;Acc:31683]
rs2239835	X	RP1-171K16.5;SPANXA2-OT1	Clone-based (Vega) gene;HGNC Symbol	;SPANXA2 overlapping transcript 1 (non-protein coding) [Source:HGNC Symbol;Acc:31683]
rs6636421	X	SPANXA2-OT1	HGNC Symbol	SPANXA2 overlapping transcript 1 (non-protein coding) [Source:HGNC Symbol;Acc:31683]

SNP	Chromosome	gene name	gene source	description
rs7060030	X	SPANXA2-OT1	HGNC Symbol	SPANXA2 overlapping transcript 1 (non-protein coding) [Source:HGNC Symbol;Acc:31683]
rs11796588	X	SPANXA2-OT1	HGNC Symbol	SPANXA2 overlapping transcript 1 (non-protein coding) [Source:HGNC Symbol;Acc:31683]
rs5954387				
rs6634314				
rs59092255				
rs6636440				
rs12390429				
rs144568404				
rs5908013				
rs73635182				
rs7054385				
rs5954414				
rs595				
rs73238509				
rs5908024				
rs7061536				
rs5907175				
rs16979855				
rs12862702				
rs6418866				
rs142555383				
rs5908045				
rs62614109				
rs12009454				
rs5908051				
rs146831141				
rs139782656				
rs55668399				
rs1476361				
rs5954446				
rs9887407	X	MAGEC3	HGNC Symbol	melanoma antigen family C, 3 [Source:HGNC Symbol;Acc:23798]
rs115454657	X	MAGEC3	HGNC Symbol	melanoma antigen family C, 3 [Source:HGNC Symbol;Acc:23798]
rs73238531	X	MAGEC3	HGNC Symbol	melanoma antigen family C, 3 [Source:HGNC Symbol;Acc:23798]

SNP	Chromosome	gene name	gene source	description
rs73238533	X	MAGEC3	HGNC Symbol	melanoma antigen family C, 3 [Source:HGNC Symbol;Acc:23798]
rs1119116	X	MAGEC3	HGNC Symbol	melanoma antigen family C, 3 [Source:HGNC Symbol;Acc:23798]
rs5908060	X	MAGEC3	HGNC Symbol	melanoma antigen family C, 3 [Source:HGNC Symbol;Acc:23798]
rs1120947	X	MAGEC3	HGNC Symbol	melanoma antigen family C, 3 [Source:HGNC Symbol;Acc:23798]
rs5908063	X	MAGEC3	HGNC Symbol	melanoma antigen family C, 3 [Source:HGNC Symbol;Acc:23798]
rs12833353	X	MAGEC3	HGNC Symbol	melanoma antigen family C, 3 [Source:HGNC Symbol;Acc:23798]
rs62611961	X	MAGEC3	HGNC Symbol	melanoma antigen family C, 3 [Source:HGNC Symbol;Acc:23798]
rs13440525	X	MAGEC3	HGNC Symbol	melanoma antigen family C, 3 [Source:HGNC Symbol;Acc:23798]
rs5907191	X	MAGEC3	HGNC Symbol	melanoma antigen family C, 3 [Source:HGNC Symbol;Acc:23798]
rs2868576	X	MAGEC3	HGNC Symbol	melanoma antigen family C, 3 [Source:HGNC Symbol;Acc:23798]
rs73577986	X	MAGEC3	HGNC Symbol	melanoma antigen family C, 3 [Source:HGNC Symbol;Acc:23798]
rs73577990	X	MAGEC3	HGNC Symbol	melanoma antigen family C, 3 [Source:HGNC Symbol;Acc:23798]
rs11095909	X	MAGEC3	HGNC Symbol	melanoma antigen family C, 3 [Source:HGNC Symbol;Acc:23798]
rs61348924	X	MAGEC3	HGNC Symbol	melanoma antigen family C, 3 [Source:HGNC Symbol;Acc:23798]
rs73577998	X	MAGEC3	HGNC Symbol	melanoma antigen family C, 3 [Source:HGNC Symbol;Acc:23798]
rs5908065	X	MAGEC3	HGNC Symbol	melanoma antigen family C, 3 [Source:HGNC Symbol;Acc:23798]
rs176024	X	MAGEC3	HGNC Symbol	melanoma antigen family C, 3 [Source:HGNC Symbol;Acc:23798]
rs176025	X	MAGEC3	HGNC Symbol	melanoma antigen family C, 3 [Source:HGNC Symbol;Acc:23798]
rs176026	X	MAGEC3	HGNC Symbol	melanoma antigen family C, 3 [Source:HGNC Symbol;Acc:23798]
rs176029				
rs55763214				
rs4553040				
rs16980287				
rs176034				
rs41299076	X	MAGEC1	HGNC Symbol	melanoma antigen family C, 1 [Source:HGNC Symbol;Acc:6812]
rs41300301	X	MAGEC1	HGNC Symbol	melanoma antigen family C, 1 [Source:HGNC Symbol;Acc:6812]
rs176053				
rs17323542				
rs5907195				
rs6634336				
rs10465401				
rs67371067				
rs17323583				

SNP	Chromosome	gene name	gene source	description
rs6636538				
rs5908078				
rs5954469				
rs17257697				
rs5954472				
rs12687094				
rs4825045				
rs73238557				
rs6634348				
rs12833422				
rs5953652				
rs1534907				
rs2223313				
rs60015258				
rs11795547				
rs111329029				
rs12013120				
rs5908092				
rs73590652				
rs72617990				
rs72617991				
rs73238571				
rs4503214				
rs2144095				
rs5954512				
rs73238580				
rs73594697				
rs59918747				
rs5908126				
rs2179313				
rs72617996				
rs72617997				
rs6528958				

SNP	Chromosome	gene name	gene source	description
rs5908145				
rs5954519				
rs12012883				
rs12012067				
rs2206118				
rs73238592				
rs73238593				
rs5908162				
rs17413				
rs1555058				
rs12009112				
rs5907239				
rs62613599				
rs7065189				
rs11095946				
rs5908188	X	RP3-406C18.1	Clone-based (Vega) gene	
rs73582953	X	RP3-406C18.1	Clone-based (Vega) gene	
rs5908192				
rs56355606				
rs5908197				
rs7053223				
rs5954551				
rs17323862				
rs5908206	X	RP3-406C18.2	Clone-based (Vega) gene	
rs4825094				
rs12710619				
rs28567523				
rs5954557				
rs5908213				
rs5907255				
rs56254936				

SNP	Chromosome	gene name	gene source	description
rs114249495				
rs3765272	X	MAGEC2	HGNC Symbol	melanoma antigen family C, 2 [Source:HGNC Symbol;Acc:13574]
rs2233058				
rs5953684				
rs9887437				
rs145608549				
rs5908226				
rs12390344				
rs62613621				
rs11796500				
rs12390872				
rs7060449				
rs112846100				
rs5907262				
rs56346761				
rs764198				
rs4824895				
rs5908239				
rs5953699				
rs7063947				
rs10521816				
rs5908264				
rs57233941				
rs5907274				
rs5908266				
rs5908275				
rs3135497				
rs5908277				
rs5954610				
rs67123115				
rs11798760				
rs5908284				
rs17258245				

SNP	Chromosome	gene name	gene source	description
rs16978831				
rs5953705				
rs17324149				
rs73240699				
rs146030802				
rs1535162				
rs1476385				
rs7060609				
rs6529005				
rs5953706				
rs5954661				
rs145699557				
rs2207810				
rs4825126				
rs5954669				
rs73242420				
rs16993896				
rs4824906				
rs3736714				
rs7050149				
rs5954679				
rs75109052				
rs2223512				
rs5954681				
rs6636708				
rs146573698				
rs5908339				
rs5908351				
rs142323002				
rs5908353				
rs73229712				
rs34930800				
rs59225271				

SNP	Chromosome	gene name	gene source	description
rs56695249				
rs717631				
rs11095994				
rs200282680				
rs7059455				
rs5954720				
rs5908359				
rs5908361				
rs73599503				
rs5953735				
rs5953736				
rs5954735				
rs5953738				
rs4452931				
rs5954737				
rs6636738				
rs12832121				
rs149254217				
rs6636756				
rs6636765				
rs12011192				
rs5954781				
rs12848504				
rs79359302				
rs113116494				
rs34185978				
rs7054922				
rs73594601				
rs4825135				
rs5954791				
rs76033124				
rs17258580				
rs113212226				

SNP	Chromosome	gene name	gene source	description
rs4535867				
rs35171115				
rs5907337				
rs73229744				
rs4545220				
rs150653006				
rs150982418				
rs138880414				
rs62602401				
rs148799459				
rs73229751				
rs151042990				
rs7878871				
rs142148286				
rs4446860				
rs12009282				
rs5908423				
rs12848812				
rs73229758				
rs2206216				
rs6529070				
rs7880233				
rs6636842				
rs5908436				
rs2092291				
rs7883119				
rs7884237				
rs5954882				
rs2143041				
rs991071				
rs991256				
rs34954325				
rs3765268				

SNP	Chromosome	gene name	gene source	description
rs62600824				
rs2223452				
rs5908445				
rs35506718				
rs5907365				
rs1569562				
rs5908454				
rs62600831				
rs73554123				
rs5954922				
rs1475239				
rs5908480				
rs143200018				
rs5953800				
rs28653045				
rs5908502				
rs4825145				
rs12014401				
rs4548313				
rs5908517				
rs62600572				
rs5908519				
rs6634456				
rs17329976				
rs144161693				
rs2224815				
rs5908524				
rs12840560				
rs12556170				
rs12007888				
rs5908531				
rs5908533				
rs139466070				

SNP	Chromosome	gene name	gene source	description
rs5907387				
rs59534199				
rs5908537				
rs5954947				
rs58124247				
rs6529095				
rs2865226				
rs35739866				
rs12556731				
rs1005317				
rs73558816				
rs5954955				
rs11797727				
rs5908568				
rs2005623				
rs149692940				
rs5908583				
rs73575404				
rs139194674				
rs6529115				
rs80209015				
rs5907408				
rs5908622				
rs5953823				
rs5954988				
rs113432185				
rs5907412				
rs7062935				
rs148362448				
rs148477370	X;HG1458_PATCH	GS1-256O22.5	Clone-based (Vega) gene	
rs5907416	X;HG1458_PATCH	GS1-256O22.5	Clone-based (Vega) gene	

SNP	Chromosome	gene name	gene source	description
rs12012987	X;HG1458_PATCH	GS1-256O22.5	Clone-based (Vega) gene	
rs5908642	X;HG1458_PATCH	GS1-256O22.5	Clone-based (Vega) gene	
rs67474692	X;HG1458_PATCH	GS1-256O22.5	Clone-based (Vega) gene	
rs5955000	X;HG1458_PATCH	PGBD4P6;GS1-256O22.5	HGNC Symbol;Clone-based (Vega) gene	piggyBac transposable element derived 4 pseudogene 6 [Source:HGNC Symbol;Acc:44071];
rs5907420	X;HG1458_PATCH	GS1-256O22.5	Clone-based (Vega) gene	
rs728395	X;HG1458_PATCH	GS1-256O22.5	Clone-based (Vega) gene	
rs4825158	X;HG1458_PATCH	GS1-256O22.5	Clone-based (Vega) gene	
rs5908648	X;HG1458_PATCH	GS1-256O22.5	Clone-based (Vega) gene	
rs112587684	X;HG1458_PATCH	GS1-256O22.5	Clone-based (Vega) gene	
rs6529132	X;HG1458_PATCH	GS1-256O22.5	Clone-based (Vega) gene	
rs12558189	X;HG1458_PATCH	GS1-256O22.5	Clone-based (Vega) gene	
rs59759626	X;HG1458_PATCH	GS1-256O22.5	Clone-based (Vega) gene	
rs4598387	X;HG1458_PATCH	GS1-256O22.5	Clone-based (Vega) gene	
rs5908660	X;HG1458_PATCH	GS1-256O22.5	Clone-based (Vega) gene	
rs6634478	X;HG1458_PATCH	GS1-256O22.5	Clone-based (Vega) gene	
rs5908665	X;HG1458_PATCH	GS1-256O22.5	Clone-based (Vega) gene	
rs7886052	X;HG1458_PATCH	GS1-256O22.5	Clone-based (Vega) gene	
rs5907427	X;HG1458_PATCH	GS1-256O22.5	Clone-based (Vega) gene	
rs35352030	X;HG1458_PATCH	GS1-256O22.5	Clone-based (Vega) gene	

SNP	Chromosome	gene name	gene source	description
rs5953842	X;HG1458_PATCH	GS1-256O22.5	Clone-based (Vega) gene	
rs5955035	X;HG1458_PATCH	GS1-256O22.5	Clone-based (Vega) gene	
rs138975666	X;HG1458_PATCH	GS1-256O22.5	Clone-based (Vega) gene	
rs67469816	X;HG1458_PATCH	GS1-256O22.5	Clone-based (Vega) gene	
rs11798895	X;HG1458_PATCH	GS1-256O22.5	Clone-based (Vega) gene	
rs73235664	X;HG1458_PATCH	GS1-256O22.5	Clone-based (Vega) gene	
rs6634495	X;HG1458_PATCH	GS1-256O22.5	Clone-based (Vega) gene	
rs16979361	X	GS1-256O22.5	Clone-based (Vega) gene	
rs6654212	X	SPANXN3;GS1-256O22.5	HGNC Symbol;Clone-based (Vega) gene	SPANX family, member N3 [Source:HGNC Symbol;Acc:33176];
rs6529146	X	SPANXN3;GS1-256O22.5	HGNC Symbol;Clone-based (Vega) gene	SPANX family, member N3 [Source:HGNC Symbol;Acc:33176];
rs144925264	X	SPANXN3;GS1-256O22.5	HGNC Symbol;Clone-based (Vega) gene	SPANX family, member N3 [Source:HGNC Symbol;Acc:33176];
rs5955068				
rs2865818	X	GS1-256O22.1	Clone-based (Vega) gene	
rs73235693				
rs59500411				
rs2207578				
rs742998				
rs7051503				
rs5907437				
rs4825169				
rs5908698				
rs5908714				
rs34493995				

SNP	Chromosome	gene name	gene source	description
rs75201119				
rs5908728				
rs16979468				
rs993478	X	SLITRK4	HGNC Symbol	SLIT and NTRK-like family, member 4 [Source:HGNC Symbol;Acc:23502]
rs12156770	X;HG1458_PATCH	SLITRK4	HGNC Symbol;UniProtKB Gene Name	SLIT and NTRK-like family, member 4 [Source:HGNC Symbol;Acc:23502];
rs34811796				
rs11798334				
rs851082				
rs851076				
rs851075				
rs144651034				
rs12860639				
rs34446555				
rs4825172				
rs17259195				
rs5908748				
rs12687823				
rs73242099				
rs5907463	X	SPANXN2	HGNC Symbol	SPANX family, member N2 [Source:HGNC Symbol;Acc:33175]
rs6637085				
rs5955106				
rs5953865				
rs5907474				
rs73244008				
rs73244014	X;HG1458_PATCH	RP3-526F5.2	Clone-based (Vega) gene	
rs4633171	X;HG1458_PATCH	RP3-526F5.2	Clone-based (Vega) gene	
rs12013944	X;HG1458_PATCH	RP3-526F5.2	Clone-based (Vega) gene	
rs112038888	X;HG1458_PATCH	RP3-526F5.2	Clone-based (Vega) gene	

SNP	Chromosome	gene name	gene source	description
rs4307462	X;HG1458_PATCH	RP3-526F5.2	Clone-based (Vega) gene	
rs28361330	X;HG1458_PATCH	RP3-526F5.2	Clone-based (Vega) gene	
rs5908810	X;HG1458_PATCH	RP3-526F5.2	Clone-based (Vega) gene	
rs78984626	X;HG1458_PATCH	RP3-526F5.2	Clone-based (Vega) gene	
rs12157034				
rs5907499				
rs5908823				
rs17259358				
rs73244019				
rs237494				
rs237505				
rs237520	X	UBE2NL	HGNC Symbol	ubiquitin-conjugating enzyme E2N-like [Source:HGNC Symbol;Acc:31710]
rs138291813				
rs28541728				
rs6637101				
rs5908832				
rs114126177				
rs6529189				
rs35909982				
rs5908840				
rs9699308				
rs5955168				
rs12382468				
rs5908863				
rs12383919				
rs138433991				
rs12387545				
rs62609462				
rs2500149				
rs2473419				

SNP	Chromosome	gene name	gene source	description
rs62609463				
rs139700644				
rs144217035				
rs141018304				
rs142486214				
rs6634569				
rs843025				
rs140300511				
rs186579469				
rs73248226				
rs150115643				
rs151334818				
rs859940				
rs2746564				
rs6649724				
rs859927				
rs7056059				
rs5908964				
rs73232108				
rs4590570				
rs977953				
rs859909				
rs5955327				
rs56185601				
rs6637217				
rs56989033				
rs2024607				
rs148296148				
rs28523847				
rs144487515				
rs58700419				
rs11497363				
rs11497364				

SNP	Chromosome	gene name	gene source	description
rs28445198				
rs28605715				
rs9699290				
rs28546204				
rs6649631				
rs149667230				
rs5945513				
rs5987334				
rs16995302				
rs16995391				
rs17309667				
rs113562832				
rs139178994				
rs17243736				
rs112858201				
rs73232180				
rs16995431				
rs5987323				
rs12857448				
rs62598725				
rs5945466				
rs62598750				
rs12860325				
rs73588037				
rs5987373				
rs5987372				
rs9781142				
rs765082				
rs5945484				
rs112915389				
rs2815683				
rs2815679				
rs12863927				

SNP	Chromosome	gene name	gene source	description
rs5966040				
rs9698796				
rs2742600				
rs73233705				
rs12559795				
rs12559109				
rs2742590				
rs1883120				
rs57919653				
rs6653372				
rs12013685				
rs62598755				
rs5919922				
rs34443517				
rs7049661				
rs4430214				
rs73233724				
rs5919645				
rs11797855				
rs2382931				
rs2382932				
rs7049249				
rs62597384				
rs73584878				
rs191398726				
rs143640008				
rs57341603				
rs10482174				
rs11094435				
rs1736865				
rs138747076				
rs5920329				
rs7876075				

SNP	Chromosome	gene name	gene source	description
rs35542182				
rs73233734				
rs67922130				
rs186982583				
rs6627018				
rs7062052				
rs5920352				
rs6525897				
rs17318718				
rs73233767				
rs5920369				
rs73233780				
rs73233792				
rs17310108				
rs16995236				
rs17318896				
rs5920415				
rs1930679				
rs55646380				
rs914820				
rs67687429				
rs6627057				
rs73235510				
rs11094456				
rs4515687				
rs7887503				
rs12558085				
rs1325103				
rs151005952				
rs1980775				
rs471551				
rs62600221				
rs566103				

SNP	Chromosome	gene name	gene source	description
rs55675431				
rs9988299				
rs112864694				
rs477202				
rs5920445				
rs150777394				
rs4827773				
rs479943				
rs545922				
rs5919803				
rs34366602				
rs535546				
rs57580842				
rs78807285				
rs5966452				
rs5920458				
rs7885395				
rs6626331				
rs5919810				
rs1458749				
rs12688576				
rs2384961				
rs9792699				
rs17244302				
rs41416052				
rs6525936				
rs5919817				
rs62597893				
rs633136				
rs6627094				
rs5919819				
rs1151979				
rs5920480				

SNP	Chromosome	gene name	gene source	description
rs55634273				
rs1974266				
rs5965847				
rs141737930				
rs5966501				
rs5920501				
rs17244441				
rs150362051				
rs11094472				
rs5966506				
rs1454679				
rs12848856				
rs5919839				
rs144384750				
rs5920520				
rs2891670				
rs6653349				
rs1174079				
rs72608971				
rs5920548				
rs5966520				
rs143116064				
rs12687323				
rs1174138				
rs1174135				
rs112220934				
rs7876275				
rs1143920				
rs1781502				
rs1618046				
rs1648730				
rs1727454				
rs5920597				

SNP	Chromosome	gene name	gene source	description
rs7357841				
rs112620162				
rs1174503				
rs140591033				
rs12557016				
rs57205492				
rs3865789				
rs142416631				
rs6626396				
rs73237918				
rs12689869				
rs6626400				
rs1022645				
rs62600299				
rs6626407				
rs147750368				
rs113482952				
rs62600302				
rs2207824				
rs2842670				
rs2748627				
rs138367545				
rs5919609				
rs2180219				
rs146458110				
rs2842675				
rs17272727				
rs142778097				
rs74712822				
rs4827655				
rs5919904				
rs1009773				
rs2748579				

SNP	Chromosome	gene name	gene source	description
rs6626437				
rs2748581				
rs7052650				
rs12557206				
rs56322118	X;HG1459_PATCH	SLITRK2	HGNC Symbol	SLIT and NTRK-like family, member 2 [Source:HGNC Symbol;Acc:13449]
rs3810704	X;HG1459_PATCH	SLITRK2	HGNC Symbol	SLIT and NTRK-like family, member 2 [Source:HGNC Symbol;Acc:13449]
rs55810646	X	SLITRK2	HGNC Symbol	SLIT and NTRK-like family, member 2 [Source:HGNC Symbol;Acc:13449]
rs2748588	X;HG1459_PATCH	SLITRK2	HGNC Symbol	SLIT and NTRK-like family, member 2 [Source:HGNC Symbol;Acc:13449]
rs5919909	X;HG1459_PATCH	TMEM257	HGNC Symbol	transmembrane protein 257 [Source:HGNC Symbol;Acc:2562]
rs2057211				
rs7063240				
rs147425194				
rs34089154				
rs12558701				
rs12556597				
rs5919919				
rs75010330				
rs148407294				
rs7050019				
rs6626452				
rs17311032				
rs5919927				
rs4291408				
rs4483305				
rs55876683				
rs143853548				
rs62598980				
rs5919941				
rs10521856				
rs2405859				

SNP	Chromosome	gene name	gene source	description
rs905089				
rs73237956				
rs5919957				
rs60936992				
rs56789000				
rs66471700				
rs5919633				
rs5919975				
rs73237975				
rs73237976				
rs1339597				
rs5965660	X;HG1459_PATCH	MIR888	HGNC Symbol	microRNA 888 [Source:HGNC Symbol;Acc:33648]
rs73584968				
rs5919986				
rs73237985				
rs73237986				
rs7064276				
rs36115511				
rs1856568				
rs78921565				
rs73637349				
rs7054873				
rs12710603				
rs73237998				
rs5965685				
rs73237999				
rs7061770				
rs5920017				
rs4300131				
rs5920027				
rs7060618				
rs7887203				

SNP	Chromosome	gene name	gene source	description
rs5966065				
rs12850809				
rs5965691				
rs6626082				
rs73240007				
rs5965703				
rs73240008				
rs16993604				
rs113232391				
rs111358462				
rs17245530				
rs73240009				
rs6626484				
rs2891792				
rs7050911				
rs4259541				
rs73240024				
rs5920062				
rs1934238				
rs17311508				
rs9306717				
rs1998004				
rs5920077				
rs112816911				
rs6626095				
rs4302984				
rs5966154				
rs17311536				
rs6626099				
rs73240034				
rs112561283				
rs6626519				
rs73240036				

SNP	Chromosome	gene name	gene source	description
rs7066221				
rs55751118				
rs7884523				
rs55982135				
rs35038499				
rs232707				
rs61093572				
rs6626537				
rs5920112				
rs67294682				
rs73240048				
rs78953490				
rs143706656				
rs12007117				
rs2077381				
rs5919699				
rs62608625				
rs7877832				
rs7064861				
rs150882550				
rs5920185				
rs147999535				
rs5966233				
rs2891838				
rs5920214				
rs5920218				
rs2207928				
rs4827618				
rs73240082				
rs5919714				
rs12855563				
rs145482947				
rs7051053				

SNP	Chromosome	gene name	gene source	description
rs111601632				
rs2213728				
rs1159881				
rs73240096				
rs62609486				
rs381365				
rs415969				
rs407379				
rs138790123				
rs73244318				
rs419696				
rs451559				
rs73244326				
rs394346				
rs142666096				
rs12845924				
rs62606761				
rs72611215				
rs73637385				
rs5920283				
rs72611218				
rs7063697				
rs5951898				
rs1415127				
rs1072149				
rs6626795				
rs6626192				
rs6626802				
rs62609279				
rs73244362				
rs5951853				
rs5951875	HG1459_PATCH;X	RP1-73A14.2	Clone-based (Vega) gene	

SNP	Chromosome	gene name	gene source	description
rs5904872				
rs6626205				
rs6626213				
rs73605022				
rs73244365				
rs5904916				
rs73640009				
rs5951768				
rs5951903				
rs5951905				
rs5951906				
rs4824278				
rs6626224				
rs66774298				
rs12839843				
rs5904936				
rs12011838				
rs5951924				
rs12007404				
rs41462848				
rs73599931				
rs5904699				
rs147889091				
rs16994035				
rs5904714				
rs12559851				
rs2392722				
rs7060854				
rs5951952				
rs113748654				
rs5904726				
rs6626232				
rs111830525				

SNP	Chromosome	gene name	gene source	description
rs61560773				
rs5905028				
rs2504169				
rs76666504				
rs12115908				
rs16994071				
rs73243487				
rs55932702				
rs11796968				
rs144803799				
rs77164493				
rs73243495				
rs66503535				
rs73243501				
rs73245504				
rs6653403				
rs7881344				
rs12847288				
rs111831187				
rs516931				
rs73245531				
rs12854961				
rs689417				
rs73245541				
rs614640				
rs1359160				
rs642359				
rs138095962				
rs58532472				
rs6626903				
rs2719867				
rs2719873				
rs73613016				

SNP	Chromosome	gene name	gene source	description
rs55730544				
rs5904741				
rs73245572				
rs2719835				
rs12846857				
rs6626255				
rs6626911				
rs966844				
rs61536918				
rs2954104				
rs73245578				
rs5952010				
rs7063331				
rs2761605				
rs6626268				
rs2742897				
rs2021952				
rs2742901				
rs2719848				
rs112908590				
rs1323747				
rs5905096				
rs112260972				
rs2761613				
rs5905099				
rs12014784				
rs2742911				
rs2761620				
rs145693887				
rs17312638				
rs12397290				
rs144861489				
rs5905103				

SNP	Chromosome	gene name	gene source	description
rs5905111				
rs5951824				
rs111338947				
rs5904764				
rs11094430				
rs11797123				
rs73245601				
rs73602793				
rs6626935				
rs73604734				
rs1861579				
rs111900525				
rs5905141				
rs6626938				
rs5905147				
rs45631658				
rs5904796				
rs2197710				
rs62608983				
rs12556135				
rs1805420	X;HG1459_PATCH	FMR1-AS1	HGNC Symbol	FMR1 antisense RNA 1 [Source:HGNC Symbol;Acc:39081]
rs1805423	X;HG1459_PATCH	FMR1	HGNC Symbol	fragile X mental retardation 1 [Source:HGNC Symbol;Acc:3775]
rs25708	X;HG1459_PATCH	FMR1	HGNC Symbol	fragile X mental retardation 1 [Source:HGNC Symbol;Acc:3775]
rs29282	X;HG1459_PATCH	FMR1	HGNC Symbol	fragile X mental retardation 1 [Source:HGNC Symbol;Acc:3775]
rs6626284				
rs5904817				
rs5904818				
rs5951863				
rs6626288				
rs6525868	X	FMR1NB	HGNC Symbol	fragile X mental retardation 1 neighbor [Source:HGNC Symbol;Acc:26372]

SNP	Chromosome	gene name	gene source	description
rs112178075	X;HG1459_PATCH	FMR1NB	HGNC Symbol	fragile X mental retardation 1 neighbor [Source:HGNC Symbol;Acc:26372]
rs12006703	X	FMR1NB	HGNC Symbol	fragile X mental retardation 1 neighbor [Source:HGNC Symbol;Acc:26372]
rs2392877	X	FMR1NB	HGNC Symbol	fragile X mental retardation 1 neighbor [Source:HGNC Symbol;Acc:26372]
rs7889692	X	FMR1NB	HGNC Symbol	fragile X mental retardation 1 neighbor [Source:HGNC Symbol;Acc:26372]
rs764631	X;HG1459_PATCH	FMR1NB	HGNC Symbol	fragile X mental retardation 1 neighbor [Source:HGNC Symbol;Acc:26372]
rs73247527	X;HG1459_PATCH	FMR1NB	HGNC Symbol	fragile X mental retardation 1 neighbor [Source:HGNC Symbol;Acc:26372]
rs7063607	X;HG1459_PATCH	FMR1NB	HGNC Symbol	fragile X mental retardation 1 neighbor [Source:HGNC Symbol;Acc:26372]
rs5904835	X;HG1459_PATCH	FMR1NB	HGNC Symbol	fragile X mental retardation 1 neighbor [Source:HGNC Symbol;Acc:26372]
rs57731657				
rs6626982				
rs215117				
rs73247561				
rs112511344				
rs6626990				
rs62609030				
rs6525877				
rs5904873				
rs6525885				
rs7888459				
rs11798727				
rs5904675				
rs56350552				
rs5904889				
rs2392921				
rs5936310				
rs7058946				
rs5936384				
rs144265803				
rs140033857				
rs73247587				

SNP	Chromosome	gene name	gene source	description
rs5936441				
rs16994511				
rs56021264				
rs2159767				
rs2109881				
rs5936201				
rs1582301				
rs1582302				
rs111354451				
rs6641219				
rs140843538				
rs7054951				
rs9887368				
rs16994562				
rs150468904				
rs5936355				
rs5980366				
rs4844050				
rs5980520				
rs5980526				
rs17317968				
rs12857182				
rs16994583				
rs113300734				
rs12839703				
rs1978081				
rs150181861				
rs3762240	X;HG1459_PATCH	AC002368.4	Clone-based (Vega) gene	
rs73249419	X;HG1459_PATCH	AFF2	HGNC Symbol	AF4/FMR2 family, member 2 [Source:HGNC Symbol;Acc:3776]
rs1547932	X;HG1459_PATCH	AFF2	HGNC Symbol	AF4/FMR2 family, member 2 [Source:HGNC Symbol;Acc:3776]

SNP	Chromosome	gene name	gene source	description
rs7058978	X;HG1459_PATCH	AFF2	HGNC Symbol	AF4/FMR2 family, member 2 [Source:HGNC Symbol;Acc:3776]
rs12010803	X;HG1459_PATCH	AFF2	HGNC Symbol	AF4/FMR2 family, member 2 [Source:HGNC Symbol;Acc:3776]
rs73249423	X;HG1459_PATCH	AFF2	HGNC Symbol	AF4/FMR2 family, member 2 [Source:HGNC Symbol;Acc:3776]
rs5936216	X;HG1459_PATCH	AFF2	HGNC Symbol	AF4/FMR2 family, member 2 [Source:HGNC Symbol;Acc:3776]
rs1541337	X;HG1459_PATCH	AFF2	HGNC Symbol	AF4/FMR2 family, member 2 [Source:HGNC Symbol;Acc:3776]
rs139044699	X;HG1459_PATCH	AFF2	HGNC Symbol	AF4/FMR2 family, member 2 [Source:HGNC Symbol;Acc:3776]
rs73249429	X;HG1459_PATCH	AFF2	HGNC Symbol	AF4/FMR2 family, member 2 [Source:HGNC Symbol;Acc:3776]
rs7064371	X;HG1459_PATCH	AFF2	HGNC Symbol	AF4/FMR2 family, member 2 [Source:HGNC Symbol;Acc:3776]
rs149666323	X;HG1459_PATCH	AFF2	HGNC Symbol	AF4/FMR2 family, member 2 [Source:HGNC Symbol;Acc:3776]
rs739488	X;HG1459_PATCH	AFF2	HGNC Symbol	AF4/FMR2 family, member 2 [Source:HGNC Symbol;Acc:3776]
rs241124	X	AFF2	HGNC Symbol	AF4/FMR2 family, member 2 [Source:HGNC Symbol;Acc:3776]
rs241127	X;HG1459_PATCH	AFF2	HGNC Symbol	AF4/FMR2 family, member 2 [Source:HGNC Symbol;Acc:3776]
rs5936421	X;HG1459_PATCH	AFF2	HGNC Symbol	AF4/FMR2 family, member 2 [Source:HGNC Symbol;Acc:3776]
rs241082	X;HG1459_PATCH	AFF2	HGNC Symbol	AF4/FMR2 family, member 2 [Source:HGNC Symbol;Acc:3776]
rs241084	X;HG1459_PATCH	AFF2	HGNC Symbol	AF4/FMR2 family, member 2 [Source:HGNC Symbol;Acc:3776]
rs73249437	X;HG1459_PATCH	AFF2	HGNC Symbol	AF4/FMR2 family, member 2 [Source:HGNC Symbol;Acc:3776]
rs1858885	X;HG1459_PATCH	AFF2	HGNC Symbol	AF4/FMR2 family, member 2 [Source:HGNC Symbol;Acc:3776]
rs5980584	X;HG1459_PATCH	AFF2	HGNC Symbol	AF4/FMR2 family, member 2 [Source:HGNC Symbol;Acc:3776]
rs241132	X;HG1459_PATCH	AFF2	HGNC Symbol	AF4/FMR2 family, member 2 [Source:HGNC Symbol;Acc:3776]
rs4844071	X;HG1459_PATCH	AFF2	HGNC Symbol	AF4/FMR2 family, member 2 [Source:HGNC Symbol;Acc:3776]

SNP	Chromosome	gene name	gene source	description
rs145828422	X;HG1459_PATCH	AFF2	HGNC Symbol	AF4/FMR2 family, member 2 [Source:HGNC Symbol;Acc:3776]
rs723445	X;HG1459_PATCH	AFF2	HGNC Symbol	AF4/FMR2 family, member 2 [Source:HGNC Symbol;Acc:3776]
rs4844072	X;HG1459_PATCH	AFF2	HGNC Symbol	AF4/FMR2 family, member 2 [Source:HGNC Symbol;Acc:3776]
rs17252278	X;HG1459_PATCH	AFF2	HGNC Symbol	AF4/FMR2 family, member 2 [Source:HGNC Symbol;Acc:3776]
rs3790344	X;HG1459_PATCH	AFF2	HGNC Symbol	AF4/FMR2 family, member 2 [Source:HGNC Symbol;Acc:3776]
rs5936439	X;HG1459_PATCH	AFF2	HGNC Symbol	AF4/FMR2 family, member 2 [Source:HGNC Symbol;Acc:3776]
rs4844074	X;HG1459_PATCH	AFF2	HGNC Symbol	AF4/FMR2 family, member 2 [Source:HGNC Symbol;Acc:3776]
rs12556938	X;HG1459_PATCH	AFF2	HGNC Symbol	AF4/FMR2 family, member 2 [Source:HGNC Symbol;Acc:3776]
rs138415595	X;HG1459_PATCH	AFF2	HGNC Symbol	AF4/FMR2 family, member 2 [Source:HGNC Symbol;Acc:3776]
rs150709713	X;HG1459_PATCH	AFF2	HGNC Symbol	AF4/FMR2 family, member 2 [Source:HGNC Symbol;Acc:3776]
rs5980603	X;HG1459_PATCH	AFF2	HGNC Symbol	AF4/FMR2 family, member 2 [Source:HGNC Symbol;Acc:3776]
rs34463921	X	AFF2	HGNC Symbol	AF4/FMR2 family, member 2 [Source:HGNC Symbol;Acc:3776]
rs1867024	X;HG1459_PATCH	AFF2	HGNC Symbol	AF4/FMR2 family, member 2 [Source:HGNC Symbol;Acc:3776]
rs1265414	X;HG1459_PATCH	AFF2	HGNC Symbol	AF4/FMR2 family, member 2 [Source:HGNC Symbol;Acc:3776]
rs12842782	X;HG1459_PATCH	AFF2	HGNC Symbol	AF4/FMR2 family, member 2 [Source:HGNC Symbol;Acc:3776]
rs12848014	X	AFF2	HGNC Symbol	AF4/FMR2 family, member 2 [Source:HGNC Symbol;Acc:3776]
rs145732787	X;HG1459_PATCH	AFF2	HGNC Symbol	AF4/FMR2 family, member 2 [Source:HGNC Symbol;Acc:3776]
rs55711809	X;HG1459_PATCH	AFF2	HGNC Symbol	AF4/FMR2 family, member 2 [Source:HGNC Symbol;Acc:3776]
rs6655003	X;HG1459_PATCH	AFF2	HGNC Symbol	AF4/FMR2 family, member 2 [Source:HGNC Symbol;Acc:3776]
rs5936463	X;HG1459_PATCH	AFF2	HGNC Symbol	AF4/FMR2 family, member 2 [Source:HGNC Symbol;Acc:3776]
rs4844078	X;HG1459_PATCH	AFF2	HGNC Symbol	AF4/FMR2 family, member 2 [Source:HGNC Symbol;Acc:3776]

SNP	Chromosome	gene name	gene source	description
rs6641482	X	AFF2	HGNC Symbol	AF4/FMR2 family, member 2 [Source:HGNC Symbol;Acc:3776]
rs12011478				
rs6641505				
rs112100352				
rs2188626				
rs12556508				
rs73249456				
rs148101966				
rs5936241				
rs16995043				
rs10127314				
rs17252537				
rs5980419				
rs112402266				
rs41326247				
rs138074222				
rs2066893				
rs732572				
rs5980427				
rs41373847				
rs4844009				
rs10521881				
rs12859656				
rs145808308				
rs56344234				
rs1882730				
rs140405544				
rs1120209				
rs17371				
rs1329431				
rs985081				
rs148748554				
rs10482431				

SNP	Chromosome	gene name	gene source	description
rs67580407				
rs4843985				
rs73240470				
rs2066883				
rs1329432				
rs4843986	X;HG1459_PATCH	IDS	HGNC Symbol;UniProtKB Gene Name	iduronate 2-sulfatase [Source:HGNC Symbol;Acc:5389];Iduronate 2-sulfatase (Hunter syndrome), isoform CRA_e; Iduronate 2-sulfatase 14 kDa chain; cDNA FLJ42669 fis, clone BRAMY2022168, highly similar to IDURONATE 2-SULFATASE [Source:UniProtKB/TrEMBL;Acc:B3KWA1]
rs1141608	X;HG1459_PATCH	IDS	HGNC Symbol;UniProtKB Gene Name	iduronate 2-sulfatase [Source:HGNC Symbol;Acc:5389];Iduronate 2-sulfatase (Hunter syndrome), isoform CRA_e; Iduronate 2-sulfatase 14 kDa chain; cDNA FLJ42669 fis, clone BRAMY2022168, highly similar to IDURONATE 2-SULFATASE [Source:UniProtKB/TrEMBL;Acc:B3KWA1]
rs605115	X;HG1459_PATCH	IDS;AF011889.2	HGNC Symbol;Clone-based (Vega) gene;UniProtKB Gene Name	iduronate 2-sulfatase [Source:HGNC Symbol;Acc:5389];;Iduronate 2-sulfatase (Hunter syndrome), isoform CRA_e; Iduronate 2-sulfatase 14 kDa chain; cDNA FLJ42669 fis, clone BRAMY2022168, highly similar to IDURONATE 2-SULFATASE [Source:UniProtKB/TrEMBL;Acc:B3KWA1]
rs1999128	X;HG1459_PATCH	IDS;AF011889.2	HGNC Symbol;Clone-based (Vega) gene;UniProtKB Gene Name	iduronate 2-sulfatase [Source:HGNC Symbol;Acc:5389];;Iduronate 2-sulfatase (Hunter syndrome), isoform CRA_e; Iduronate 2-sulfatase 14 kDa chain; cDNA FLJ42669 fis, clone BRAMY2022168, highly similar to IDURONATE 2-SULFATASE [Source:UniProtKB/TrEMBL;Acc:B3KWA1]
rs1074248	X;HG1459_PATCH	IDS;LINC00893	HGNC Symbol;UniProtKB Gene Name	iduronate 2-sulfatase [Source:HGNC Symbol;Acc:5389];Iduronate 2-sulfatase (Hunter syndrome), isoform CRA_e; Iduronate 2-sulfatase 14 kDa chain; cDNA FLJ42669 fis, clone BRAMY2022168, highly similar to IDURONATE 2-SULFATASE [Source:UniProtKB/TrEMBL;Acc:B3KWA1];long intergenic non-protein coding RNA 893 [Source:HGNC Symbol;Acc:44265]
rs583839				
rs5741912				
rs7052815				
rs66829792	X;HG1459_PATCH	TMEM185A;FAM11A	HGNC Symbol;UniProtKB Gene Name	transmembrane protein 185A [Source:HGNC Symbol;Acc:17125];Family with sequence similarity 11, member A, isoform CRA_a; cDNA FLJ46855 fis, clone UTERU3010029, highly similar to Protein FAM11A [Source:UniProtKB/TrEMBL;Acc:B3KY49]
rs687632				
rs4844037				
rs139079774				
rs5980493				
rs12556082				
rs62612570	X;HG1459_PATCH	MAGEA11	HGNC Symbol	melanoma antigen family A, 11 [Source:HGNC Symbol;Acc:6798]

SNP	Chromosome	gene name	gene source	description
rs185512115				
rs381321				
rs12012092				
rs5983899				
rs73244660				
rs5983903				
rs140720249				
rs7878544				
rs5983916	X;HG1459_PATCH	MAGEA8	HGNC Symbol	melanoma antigen family A, 8 [Source:HGNC Symbol;Acc:6806]
rs56288522	X;HG1459_PATCH	LINC00894	HGNC Symbol	long intergenic non-protein coding RNA 894 [Source:HGNC Symbol;Acc:48579]
rs10856256	X;HG1459_PATCH	LINC00894	HGNC Symbol	long intergenic non-protein coding RNA 894 [Source:HGNC Symbol;Acc:48579]
rs144116872	X;HG1459_PATCH	LINC00894	HGNC Symbol	long intergenic non-protein coding RNA 894 [Source:HGNC Symbol;Acc:48579]
rs151052430	X;HG1459_PATCH	LINC00894	HGNC Symbol	long intergenic non-protein coding RNA 894 [Source:HGNC Symbol;Acc:48579]
rs62611265	X;HG1459_PATCH	LINC00894	HGNC Symbol	long intergenic non-protein coding RNA 894 [Source:HGNC Symbol;Acc:48579]
rs7391189	X;HG1459_PATCH	LINC00894	HGNC Symbol	long intergenic non-protein coding RNA 894 [Source:HGNC Symbol;Acc:48579]
rs140078291	X;HG1459_PATCH	LINC00894	HGNC Symbol	long intergenic non-protein coding RNA 894 [Source:HGNC Symbol;Acc:48579]
rs6649483	X;HG1459_PATCH	LINC00894	HGNC Symbol	long intergenic non-protein coding RNA 894 [Source:HGNC Symbol;Acc:48579]
rs4111082	X;HG1459_PATCH	LINC00894	HGNC Symbol	long intergenic non-protein coding RNA 894 [Source:HGNC Symbol;Acc:48579]
rs3900055	X;HG1459_PATCH	LINC00894	HGNC Symbol	long intergenic non-protein coding RNA 894 [Source:HGNC Symbol;Acc:48579]
rs73638243	X;HG1459_PATCH	LINC00894	HGNC Symbol	long intergenic non-protein coding RNA 894 [Source:HGNC Symbol;Acc:48579]
rs9698338	X;HG1459_PATCH	LINC00894	HGNC Symbol	long intergenic non-protein coding RNA 894 [Source:HGNC Symbol;Acc:48579]
rs9724045	X;HG1459_PATCH	LINC00894	HGNC Symbol	long intergenic non-protein coding RNA 894 [Source:HGNC Symbol;Acc:48579]
rs10856246	X;HG1459_PATCH	LINC00894	HGNC Symbol	long intergenic non-protein coding RNA 894 [Source:HGNC Symbol;Acc:48579]

SNP	Chromosome	gene name	gene source	description
rs12399776	X;HG1459_PATCH	LINC00894	HGNC Symbol	long intergenic non-protein coding RNA 894 [Source:HGNC Symbol;Acc:48579]
rs55903510				
rs9780782				
rs9698926				
rs56066523				
rs56027404				
rs9306747				
rs9781523				
rs9284560				
rs12858909				
rs145507149				
rs5969816				
rs148407991				
rs143785399				
rs5924811				
rs12557733				
rs17318763				
rs5970467				
rs5925421				
rs4828780				
rs12010944				
rs73246815				
rs7054236				
rs591456				
rs17318777				
rs34964358				
rs147270834				
rs572781				
rs72612713	X	MAMLD1	HGNC Symbol	mastermind-like domain containing 1 [Source:HGNC Symbol;Acc:2568]
rs12839256	X	MAMLD1	HGNC Symbol	mastermind-like domain containing 1 [Source:HGNC Symbol;Acc:2568]
rs5925537	X	MAMLD1	HGNC Symbol	mastermind-like domain containing 1 [Source:HGNC Symbol;Acc:2568]
rs693913	X	MAMLD1	HGNC Symbol	mastermind-like domain containing 1 [Source:HGNC Symbol;Acc:2568]

SNP	Chromosome	gene name	gene source	description
rs73246818	X	MAMLD1	HGNC Symbol	mastermind-like domain containing 1 [Source:HGNC Symbol;Acc:2568]
rs12559488	X	MAMLD1	HGNC Symbol	mastermind-like domain containing 1 [Source:HGNC Symbol;Acc:2568]
rs1983610	X	MAMLD1	HGNC Symbol	mastermind-like domain containing 1 [Source:HGNC Symbol;Acc:2568]
rs12013750	X	MAMLD1	HGNC Symbol	mastermind-like domain containing 1 [Source:HGNC Symbol;Acc:2568]
rs5970576	X	MAMLD1	HGNC Symbol	mastermind-like domain containing 1 [Source:HGNC Symbol;Acc:2568]
rs4828563	X	MAMLD1	HGNC Symbol	mastermind-like domain containing 1 [Source:HGNC Symbol;Acc:2568]
rs73246819	X	MAMLD1	HGNC Symbol	mastermind-like domain containing 1 [Source:HGNC Symbol;Acc:2568]
rs12389317	X	MAMLD1	HGNC Symbol	mastermind-like domain containing 1 [Source:HGNC Symbol;Acc:2568]
rs150042509	X	MAMLD1	HGNC Symbol	mastermind-like domain containing 1 [Source:HGNC Symbol;Acc:2568]
rs5925007	X	MAMLD1	HGNC Symbol	mastermind-like domain containing 1 [Source:HGNC Symbol;Acc:2568]
rs60645255	X	MAMLD1	HGNC Symbol	mastermind-like domain containing 1 [Source:HGNC Symbol;Acc:2568]
rs611711	X	MAMLD1	HGNC Symbol	mastermind-like domain containing 1 [Source:HGNC Symbol;Acc:2568]
rs2266826	X	MAMLD1	HGNC Symbol	mastermind-like domain containing 1 [Source:HGNC Symbol;Acc:2568]
rs2266827	X	MAMLD1	HGNC Symbol	mastermind-like domain containing 1 [Source:HGNC Symbol;Acc:2568]
rs2266831	X	MAMLD1	HGNC Symbol	mastermind-like domain containing 1 [Source:HGNC Symbol;Acc:2568]
rs3788806	X	MAMLD1	HGNC Symbol	mastermind-like domain containing 1 [Source:HGNC Symbol;Acc:2568]
rs17252936	X	MAMLD1	HGNC Symbol	mastermind-like domain containing 1 [Source:HGNC Symbol;Acc:2568]
rs2283740	X	MAMLD1	HGNC Symbol	mastermind-like domain containing 1 [Source:HGNC Symbol;Acc:2568]
rs41313406	X	MAMLD1	HGNC Symbol	mastermind-like domain containing 1 [Source:HGNC Symbol;Acc:2568]
rs73250532	X	MAMLD1	HGNC Symbol	mastermind-like domain containing 1 [Source:HGNC Symbol;Acc:2568]
rs2073043	X	MAMLD1	HGNC Symbol	mastermind-like domain containing 1 [Source:HGNC Symbol;Acc:2568]
rs5925148	X	MAMLD1	HGNC Symbol	mastermind-like domain containing 1 [Source:HGNC Symbol;Acc:2568]
rs10127356	X	MAMLD1	HGNC Symbol	mastermind-like domain containing 1 [Source:HGNC Symbol;Acc:2568]
rs6627581	X	MAMLD1	HGNC Symbol	mastermind-like domain containing 1 [Source:HGNC Symbol;Acc:2568]
rs2283742	X	MAMLD1	HGNC Symbol	mastermind-like domain containing 1 [Source:HGNC Symbol;Acc:2568]
rs1209028	X	MAMLD1	HGNC Symbol	mastermind-like domain containing 1 [Source:HGNC Symbol;Acc:2568]
rs2266836	X	MAMLD1	HGNC Symbol	mastermind-like domain containing 1 [Source:HGNC Symbol;Acc:2568]
rs73250538	X	MAMLD1	HGNC Symbol	mastermind-like domain containing 1 [Source:HGNC Symbol;Acc:2568]
rs2266838	X	MAMLD1	HGNC Symbol	mastermind-like domain containing 1 [Source:HGNC Symbol;Acc:2568]
rs655381	X	MAMLD1	HGNC Symbol	mastermind-like domain containing 1 [Source:HGNC Symbol;Acc:2568]
rs143635208	X	MAMLD1	HGNC Symbol	mastermind-like domain containing 1 [Source:HGNC Symbol;Acc:2568]
rs587511	X	MAMLD1	HGNC Symbol	mastermind-like domain containing 1 [Source:HGNC Symbol;Acc:2568]
rs598334	X	MAMLD1	HGNC Symbol	mastermind-like domain containing 1 [Source:HGNC Symbol;Acc:2568]

SNP	Chromosome	gene name	gene source	description
rs497220	X	MAMLD1	HGNC Symbol	mastermind-like domain containing 1 [Source:HGNC Symbol;Acc:2568]
rs73250543	X	MAMLD1	HGNC Symbol	mastermind-like domain containing 1 [Source:HGNC Symbol;Acc:2568]
rs567517	X	MAMLD1	HGNC Symbol	mastermind-like domain containing 1 [Source:HGNC Symbol;Acc:2568]
rs12007216				
rs112396737				
rs498608				
rs6627643				
rs56348703				
rs5925221				
rs12559994				
rs7065239				
rs73618825				
rs73250551				
rs138625285				
rs12557844				
rs55900771	X;HG1459_PATCH	MTM1	HGNC Symbol	myotubularin 1 [Source:HGNC Symbol;Acc:7448]
rs1515963	X;HG1459_PATCH	MTM1	HGNC Symbol	myotubularin 1 [Source:HGNC Symbol;Acc:7448]
rs73250555	X;HG1459_PATCH	MTM1	HGNC Symbol	myotubularin 1 [Source:HGNC Symbol;Acc:7448]
rs5924834	X;HG1459_PATCH	MTM1	HGNC Symbol	myotubularin 1 [Source:HGNC Symbol;Acc:7448]
rs5925388	X;HG1459_PATCH	MTM1	HGNC Symbol	myotubularin 1 [Source:HGNC Symbol;Acc:7448]
rs57102007	X;HG1459_PATCH	MTM1	HGNC Symbol	myotubularin 1 [Source:HGNC Symbol;Acc:7448]
rs149701385	X;HG1459_PATCH	MTM1	HGNC Symbol	myotubularin 1 [Source:HGNC Symbol;Acc:7448]
rs55714664	X;HG1459_PATCH	MTM1	HGNC Symbol	myotubularin 1 [Source:HGNC Symbol;Acc:7448]
rs5925408	X;HG1459_PATCH	MTMR1	HGNC Symbol	myotubularin related protein 1 [Source:HGNC Symbol;Acc:7449]
rs1882710	X;HG1459_PATCH	MTMR1	HGNC Symbol	myotubularin related protein 1 [Source:HGNC Symbol;Acc:7449]
rs16996714	X;HG1459_PATCH	MTMR1	HGNC Symbol	myotubularin related protein 1 [Source:HGNC Symbol;Acc:7449]

SNP	Chromosome	gene name	gene source	description
rs5925418	X;HG1459_PATCH	MTMR1	HGNC Symbol	myotubularin related protein 1 [Source:HGNC Symbol;Acc:7449]
rs73250569	X;HG1459_PATCH	MTMR1	HGNC Symbol	myotubularin related protein 1 [Source:HGNC Symbol;Acc:7449]
rs6627801	X;HG1459_PATCH	MTMR1	HGNC Symbol	myotubularin related protein 1 [Source:HGNC Symbol;Acc:7449]
rs6627803	X;HG1459_PATCH	MTMR1	HGNC Symbol	myotubularin related protein 1 [Source:HGNC Symbol;Acc:7449]
rs78489095	X;HG1459_PATCH	MTMR1	HGNC Symbol	myotubularin related protein 1 [Source:HGNC Symbol;Acc:7449]
rs5925420	X;HG1459_PATCH	MTMR1	HGNC Symbol	myotubularin related protein 1 [Source:HGNC Symbol;Acc:7449]
rs16995747	X;HG1459_PATCH	MTMR1	HGNC Symbol	myotubularin related protein 1 [Source:HGNC Symbol;Acc:7449]
rs6877	X;HG1459_PATCH	CD99L2	HGNC Symbol	CD99 molecule-like 2 [Source:HGNC Symbol;Acc:18237]
rs9866	X;HG1459_PATCH	CD99L2	HGNC Symbol	CD99 molecule-like 2 [Source:HGNC Symbol;Acc:18237]
rs5970474	X;HG1459_PATCH	CD99L2	HGNC Symbol	CD99 molecule-like 2 [Source:HGNC Symbol;Acc:18237]
rs73624526	X;HG1459_PATCH	CD99L2	HGNC Symbol	CD99 molecule-like 2 [Source:HGNC Symbol;Acc:18237]
rs73250576	X;HG1459_PATCH	CD99L2	HGNC Symbol	CD99 molecule-like 2 [Source:HGNC Symbol;Acc:18237]
rs5924843	X;HG1459_PATCH	CD99L2	HGNC Symbol	CD99 molecule-like 2 [Source:HGNC Symbol;Acc:18237]
rs5925428	X;HG1459_PATCH	CD99L2	HGNC Symbol	CD99 molecule-like 2 [Source:HGNC Symbol;Acc:18237]
rs73250577	X;HG1459_PATCH	CD99L2	HGNC Symbol	CD99 molecule-like 2 [Source:HGNC Symbol;Acc:18237]
rs146241156	X;HG1459_PATCH	CD99L2	HGNC Symbol	CD99 molecule-like 2 [Source:HGNC Symbol;Acc:18237]
rs73609545	X;HG1459_PATCH	CD99L2	HGNC Symbol	CD99 molecule-like 2 [Source:HGNC Symbol;Acc:18237]
rs149334428	X;HG1459_PATCH	CD99L2	HGNC Symbol	CD99 molecule-like 2 [Source:HGNC Symbol;Acc:18237]
rs7887176	X	CD99L2	HGNC Symbol	CD99 molecule-like 2 [Source:HGNC Symbol;Acc:18237]
rs6653501	X;HG1459_PATCH	CD99L2	HGNC Symbol	CD99 molecule-like 2 [Source:HGNC Symbol;Acc:18237]

SNP	Chromosome	gene name	gene source	description
rs66596395	X;HG1459_PATCH	CD99L2	HGNC Symbol	CD99 molecule-like 2 [Source:HGNC Symbol;Acc:18237]
rs4240082	X;HG1459_PATCH	CD99L2	HGNC Symbol	CD99 molecule-like 2 [Source:HGNC Symbol;Acc:18237]
rs11798706	X	CD99L2	HGNC Symbol	CD99 molecule-like 2 [Source:HGNC Symbol;Acc:18237]
rs142220165	X;HG1459_PATCH	CD99L2	HGNC Symbol	CD99 molecule-like 2 [Source:HGNC Symbol;Acc:18237]
rs149345052	X;HG1459_PATCH	CD99L2	HGNC Symbol	CD99 molecule-like 2 [Source:HGNC Symbol;Acc:18237]
rs7877936				
rs72612736				
rs7056697				
rs7064262				
rs12389338				
rs34908843				
rs41304966	X;HG1459_PATCH	AF003626.1	Clone-based (Vega) gene	
rs10521890				
rs139541466				
rs237378				
rs7879970				
rs2128990				
rs5970498				
rs4828786				
rs2369404				
rs237390				
rs5924847				
rs7888762				
rs6627335				
rs1483959				
rs148304180				
rs74540486				
rs909084				
rs118722				
rs909086				

SNP	Chromosome	gene name	gene source	description
rs12836796				
rs6627831				
rs4828614				
rs633698				
rs7063866				
rs73232310				
rs237397				
rs7057942				
rs146582646				
rs237402	X;HG1459_PATCH	HMGB3	HGNC Symbol	high mobility group box 3 [Source:HGNC Symbol;Acc:5004]
rs6627833	X;HG1459_PATCH	RPL19P21	HGNC Symbol	ribosomal protein L19 pseudogene 21 [Source:HGNC Symbol;Acc:35868]
rs5970515				
rs5925465				
rs922952				
rs12863717				
rs5924858				
rs7876150				
rs4601520				
rs73237665				
rs3866985				
rs5924863				
rs5925496				
rs5970527				
rs5970528				
rs5925505				
rs17253404				
rs7879350				
rs6627353				
rs236734				
rs59831991				
rs12171762				
rs552922				

SNP	Chromosome	gene name	gene source	description
rs7881137				
rs5925522				
rs73237686				
rs5925529				
rs73237691				
rs632006				
rs12009129				
rs55871513				
rs5924886				
rs630675				
rs180488				
rs6627871				
rs180491				
rs180495				
rs180497				
rs5970547				
rs644345				
rs547043				
rs529386				
rs3827434				
rs5969987	X	GPR50-AS1	HGNC Symbol	GPR50 antisense RNA 1 [Source:HGNC Symbol;Acc:40259]
rs79178579	X	GPR50	HGNC Symbol	G protein-coupled receptor 50 [Source:HGNC Symbol;Acc:4506]
rs66603953	X	GPR50	HGNC Symbol	G protein-coupled receptor 50 [Source:HGNC Symbol;Acc:4506]
rs1202872	X	GPR50	HGNC Symbol	G protein-coupled receptor 50 [Source:HGNC Symbol;Acc:4506]
rs35089505	X	GPR50	HGNC Symbol	G protein-coupled receptor 50 [Source:HGNC Symbol;Acc:4506]
rs1202873	X	GPR50	HGNC Symbol	G protein-coupled receptor 50 [Source:HGNC Symbol;Acc:4506]
rs199797606	X	GPR50	HGNC Symbol	G protein-coupled receptor 50 [Source:HGNC Symbol;Acc:4506]
rs200787393	X	GPR50	HGNC Symbol	G protein-coupled receptor 50 [Source:HGNC Symbol;Acc:4506]
rs561077	X	GPR50	HGNC Symbol	G protein-coupled receptor 50 [Source:HGNC Symbol;Acc:4506]
rs13440581	X	GPR50	HGNC Symbol	G protein-coupled receptor 50 [Source:HGNC Symbol;Acc:4506]
rs565689				
rs481878				
rs543515				

SNP	Chromosome	gene name	gene source	description
rs5970555				
rs73239603				
rs144901787				
rs6627884				
rs7055317				
rs73239616				
rs5925540				
rs58548000	X	AF013593.1	Clone-based (Vega) gene	
rs141837155				
rs1202948				
rs17253481				
rs5924891				
rs1202913				
rs1202919				
rs17326172				
rs78972460				
rs7064079				
rs1202944				
rs6525970				
rs5970001				
rs73239639				
rs1937215				
rs4477207				
rs12010578				
rs871478				
rs73239640				
rs4828625				
rs5924897				
rs112055233				
rs4121203				
rs17319496				
rs17319503				

SNP	Chromosome	gene name	gene source	description
rs73619410				
rs4409582				
rs11798111				
rs112841813				
rs55685397				
rs12388271				
rs62609813				
rs2369717				
rs73621026				
rs176448				
rs7067140	X	VMA21	HGNC Symbol	VMA21 vacuolar H+-ATPase homolog (S. cerevisiae) [Source:HGNC Symbol;Acc:22082]
rs5924934				
rs5924633				
rs73239653				
rs139887191				
rs7876668				
rs2142017				
rs55710241				
rs111675510				
rs4017709				
rs5970066				
rs28580038				
rs5970069				
rs5924959	X	PASD1	HGNC Symbol	PAS domain containing 1 [Source:HGNC Symbol;Acc:20686]
rs5924646	X	PASD1	HGNC Symbol	PAS domain containing 1 [Source:HGNC Symbol;Acc:20686]
rs138618576	X	PASD1	HGNC Symbol	PAS domain containing 1 [Source:HGNC Symbol;Acc:20686]
rs58448897	X	PASD1	HGNC Symbol	PAS domain containing 1 [Source:HGNC Symbol;Acc:20686]
rs56035326	X	PASD1	HGNC Symbol	PAS domain containing 1 [Source:HGNC Symbol;Acc:20686]
rs56222103	X	PASD1;RP11-45D17.1	HGNC Symbol;Clone-based (Vega) gene	PAS domain containing 1 [Source:HGNC Symbol;Acc:20686];
rs5924977	X	PASD1;RP11-45D17.1	HGNC Symbol;Clone-based (Vega) gene	PAS domain containing 1 [Source:HGNC Symbol;Acc:20686];

SNP	Chromosome	gene name	gene source	description
rs5924978	X	PASD1;RP11-45D17.1	HGNC Symbol;Clone-based (Vega) gene	PAS domain containing 1 [Source:HGNC Symbol;Acc:20686];
rs5924979	X	PASD1;RP11-45D17.1	HGNC Symbol;Clone-based (Vega) gene	PAS domain containing 1 [Source:HGNC Symbol;Acc:20686];
rs12859750	X	PASD1;RP11-45D17.1	HGNC Symbol;Clone-based (Vega) gene	PAS domain containing 1 [Source:HGNC Symbol;Acc:20686];
rs7880403	X	PASD1;RP11-45D17.1	HGNC Symbol;Clone-based (Vega) gene	PAS domain containing 1 [Source:HGNC Symbol;Acc:20686];
rs12392387	X	PASD1;RP11-45D17.1	HGNC Symbol;Clone-based (Vega) gene	PAS domain containing 1 [Source:HGNC Symbol;Acc:20686];
rs73239682	X	PASD1;RP11-45D17.1	HGNC Symbol;Clone-based (Vega) gene	PAS domain containing 1 [Source:HGNC Symbol;Acc:20686];
rs10482212	X	PASD1;RP11-45D17.1	HGNC Symbol;Clone-based (Vega) gene	PAS domain containing 1 [Source:HGNC Symbol;Acc:20686];
rs6627174	X	PASD1	HGNC Symbol	PAS domain containing 1 [Source:HGNC Symbol;Acc:20686]
rs201718557	X	PASD1	HGNC Symbol	PAS domain containing 1 [Source:HGNC Symbol;Acc:20686]
rs41312626	X	PASD1	HGNC Symbol	PAS domain containing 1 [Source:HGNC Symbol;Acc:20686]
rs41299110	X	PASD1	HGNC Symbol	PAS domain containing 1 [Source:HGNC Symbol;Acc:20686]
rs4553055	X	PASD1	HGNC Symbol	PAS domain containing 1 [Source:HGNC Symbol;Acc:20686]
rs7878232				
rs4330820				
rs7471911				
rs5925006				
rs73239697				
rs58968875	X	PRRG3	HGNC Symbol	proline rich Gla (G-carboxyglutamic acid) 3 (transmembrane) [Source:HGNC Symbol;Acc:30798]
rs6627445	X	PRRG3	HGNC Symbol	proline rich Gla (G-carboxyglutamic acid) 3 (transmembrane) [Source:HGNC Symbol;Acc:30798]
rs5970097	X	PRRG3	HGNC Symbol	proline rich Gla (G-carboxyglutamic acid) 3 (transmembrane) [Source:HGNC Symbol;Acc:30798]
rs7052313	X	PRRG3	HGNC Symbol	proline rich Gla (G-carboxyglutamic acid) 3 (transmembrane) [Source:HGNC Symbol;Acc:30798]
rs41302168	X	PRRG3	HGNC Symbol	proline rich Gla (G-carboxyglutamic acid) 3 (transmembrane) [Source:HGNC Symbol;Acc:30798]
rs4323608	X	PRRG3	HGNC Symbol	proline rich Gla (G-carboxyglutamic acid) 3 (transmembrane) [Source:HGNC Symbol;Acc:30798]

SNP	Chromosome	gene name	gene source	description
rs4507912	X	PRRG3	HGNC Symbol	proline rich Gla (G-carboxyglutamic acid) 3 (transmembrane) [Source:HGNC Symbol;Acc:30798]
rs10521893	X	PRRG3	HGNC Symbol	proline rich Gla (G-carboxyglutamic acid) 3 (transmembrane) [Source:HGNC Symbol;Acc:30798]
rs6627449				
rs12839964				
rs1554914				
rs12854809				
rs3810715	X	FATE1	HGNC Symbol	fetal and adult testis expressed 1 [Source:HGNC Symbol;Acc:24683]
rs3810714	X	FATE1	HGNC Symbol	fetal and adult testis expressed 1 [Source:HGNC Symbol;Acc:24683]
rs873158	X	FATE1	HGNC Symbol	fetal and adult testis expressed 1 [Source:HGNC Symbol;Acc:24683]
rs73241808	X	FATE1	HGNC Symbol	fetal and adult testis expressed 1 [Source:HGNC Symbol;Acc:24683]
rs56766712	X	FATE1	HGNC Symbol	fetal and adult testis expressed 1 [Source:HGNC Symbol;Acc:24683]
rs13047	X	FATE1	HGNC Symbol	fetal and adult testis expressed 1 [Source:HGNC Symbol;Acc:24683]
rs11094513				
rs10521894				
rs741725				
rs73628420				
rs12008689				
rs5925018				
rs35350051	X	CNGA2	HGNC Symbol	cyclic nucleotide gated channel alpha 2 [Source:HGNC Symbol;Acc:2149]
rs12013494	X	CNGA2	HGNC Symbol	cyclic nucleotide gated channel alpha 2 [Source:HGNC Symbol;Acc:2149]
rs41289500	X	CNGA2	HGNC Symbol	cyclic nucleotide gated channel alpha 2 [Source:HGNC Symbol;Acc:2149]
rs714147	X	CNGA2	HGNC Symbol	cyclic nucleotide gated channel alpha 2 [Source:HGNC Symbol;Acc:2149]
rs7061975				
rs112523892				
rs741726				
rs5925027				
rs62610776				
rs4828647				
rs5925032				
rs55633091				
rs741727				
rs5970106				
rs62610783				

SNP	Chromosome	gene name	gene source	description
rs6627187				
rs12844305				
rs34042532				
rs73241820				
rs5970117				
rs7054854				
rs73640131				
rs5970118				
rs720378				
rs5925043				
rs113627993				
rs12558894				
rs5925050				
rs12836522				
rs73241828				
rs16996176				
rs142174885				
rs5925054				
rs7050422				
rs5969828				
rs5925062				
rs73241832				
rs144238359				
rs41529149				
rs6526041				
rs2205549				
rs12843815				
rs12387812				
rs6627483				
rs2335174				
rs5970148				
rs112646972				
rs5970151				

SNP	Chromosome	gene name	gene source	description
rs12832440	X	RP11-366F6.2	Clone-based (Vega) gene	
rs7879168	X	RP11-366F6.2	Clone-based (Vega) gene	
rs1047246	X	MAGEA4	HGNC Symbol	melanoma antigen family A, 4 [Source:HGNC Symbol;Acc:6802]
rs61746135	X	MAGEA4	HGNC Symbol	melanoma antigen family A, 4 [Source:HGNC Symbol;Acc:6802]
rs113954542				
rs6627501				
rs149642212				
rs45439991	X	GABRE	HGNC Symbol	gamma-aminobutyric acid (GABA) A receptor, epsilon [Source:HGNC Symbol;Acc:4085]
rs2256882	X	GABRE	HGNC Symbol	gamma-aminobutyric acid (GABA) A receptor, epsilon [Source:HGNC Symbol;Acc:4085]
rs1894367	X	GABRE	HGNC Symbol	gamma-aminobutyric acid (GABA) A receptor, epsilon [Source:HGNC Symbol;Acc:4085]
rs73241843	X	GABRE	HGNC Symbol	gamma-aminobutyric acid (GABA) A receptor, epsilon [Source:HGNC Symbol;Acc:4085]
rs2266855	X	GABRE	HGNC Symbol	gamma-aminobutyric acid (GABA) A receptor, epsilon [Source:HGNC Symbol;Acc:4085]
rs1139916	X	GABRE	HGNC Symbol	gamma-aminobutyric acid (GABA) A receptor, epsilon [Source:HGNC Symbol;Acc:4085]
rs2266857	X	GABRE	HGNC Symbol	gamma-aminobutyric acid (GABA) A receptor, epsilon [Source:HGNC Symbol;Acc:4085]
rs2266859	X	GABRE	HGNC Symbol	gamma-aminobutyric acid (GABA) A receptor, epsilon [Source:HGNC Symbol;Acc:4085]
rs5925077	X	GABRE	HGNC Symbol	gamma-aminobutyric acid (GABA) A receptor, epsilon [Source:HGNC Symbol;Acc:4085]
rs5924705				
rs2051529				
rs73241845				
rs145754054				
rs4537453				
rs73241852				
rs5970181				
rs5925105				
rs141283761				
rs5970194				
rs12557421				
rs61688207				
rs56053914				
rs4093545				
rs60441555				
rs58674506				

SNP	Chromosome	gene name	gene source	description
rs2172223				
rs2471382				
rs5970214				
rs726153				
rs210567				
rs2020030	X	MAGEA5;RP11-1007I13.4	HGNC Symbol;Clone-based (Vega) gene	melanoma antigen family A, 5 [Source:HGNC Symbol;Acc:6803];
rs17320283	X	RP11-329E24.6	Clone-based (Vega) gene	
rs73241870	X	RP11-329E24.6	Clone-based (Vega) gene	
rs145701879	X	GABRA3;RP11-329E24.6	HGNC Symbol;Clone-based (Vega) gene	gamma-aminobutyric acid (GABA) A receptor, alpha 3 [Source:HGNC Symbol;Acc:4077];
rs17320297	X	GABRA3;RP11-329E24.6	HGNC Symbol;Clone-based (Vega) gene	gamma-aminobutyric acid (GABA) A receptor, alpha 3 [Source:HGNC Symbol;Acc:4077];
rs6627545	X	GABRA3	HGNC Symbol	gamma-aminobutyric acid (GABA) A receptor, alpha 3 [Source:HGNC Symbol;Acc:4077]
rs17280085	X	GABRA3	HGNC Symbol	gamma-aminobutyric acid (GABA) A receptor, alpha 3 [Source:HGNC Symbol;Acc:4077]
rs62610383	X	GABRA3	HGNC Symbol	gamma-aminobutyric acid (GABA) A receptor, alpha 3 [Source:HGNC Symbol;Acc:4077]
rs994423	X	GABRA3	HGNC Symbol	gamma-aminobutyric acid (GABA) A receptor, alpha 3 [Source:HGNC Symbol;Acc:4077]
rs75662293	X	GABRA3	HGNC Symbol	gamma-aminobutyric acid (GABA) A receptor, alpha 3 [Source:HGNC Symbol;Acc:4077]
rs5970244	X	GABRA3	HGNC Symbol	gamma-aminobutyric acid (GABA) A receptor, alpha 3 [Source:HGNC Symbol;Acc:4077]
rs145959749	X	GABRA3	HGNC Symbol	gamma-aminobutyric acid (GABA) A receptor, alpha 3 [Source:HGNC Symbol;Acc:4077]
rs73241877	X	GABRA3	HGNC Symbol	gamma-aminobutyric acid (GABA) A receptor, alpha 3 [Source:HGNC Symbol;Acc:4077]
rs5924733	X	GABRA3	HGNC Symbol	gamma-aminobutyric acid (GABA) A receptor, alpha 3 [Source:HGNC Symbol;Acc:4077]
rs149700433	X	GABRA3	HGNC Symbol	gamma-aminobutyric acid (GABA) A receptor, alpha 3 [Source:HGNC Symbol;Acc:4077]
rs1492293	X	GABRA3	HGNC Symbol	gamma-aminobutyric acid (GABA) A receptor, alpha 3 [Source:HGNC Symbol;Acc:4077]
rs55747036	X	GABRA3	HGNC Symbol	gamma-aminobutyric acid (GABA) A receptor, alpha 3 [Source:HGNC Symbol;Acc:4077]
rs138169663	X	GABRA3	HGNC Symbol	gamma-aminobutyric acid (GABA) A receptor, alpha 3 [Source:HGNC Symbol;Acc:4077]
rs5970291	X	GABRA3	HGNC Symbol	gamma-aminobutyric acid (GABA) A receptor, alpha 3 [Source:HGNC Symbol;Acc:4077]
rs113259906	X	GABRA3	HGNC Symbol	gamma-aminobutyric acid (GABA) A receptor, alpha 3 [Source:HGNC Symbol;Acc:4077]
rs6526104	X	GABRA3	HGNC Symbol	gamma-aminobutyric acid (GABA) A receptor, alpha 3 [Source:HGNC Symbol;Acc:4077]
rs5970304	X	GABRA3	HGNC Symbol	gamma-aminobutyric acid (GABA) A receptor, alpha 3 [Source:HGNC Symbol;Acc:4077]

SNP	Chromosome	gene name	gene source	description
rs1109839	X	GABRA3	HGNC Symbol	gamma-aminobutyric acid (GABA) A receptor, alpha 3 [Source:HGNC Symbol;Acc:4077]
rs2092013	X	GABRA3	HGNC Symbol	gamma-aminobutyric acid (GABA) A receptor, alpha 3 [Source:HGNC Symbol;Acc:4077]
rs9306739	X	GABRA3	HGNC Symbol	gamma-aminobutyric acid (GABA) A receptor, alpha 3 [Source:HGNC Symbol;Acc:4077]
rs6627237	X	GABRA3	HGNC Symbol	gamma-aminobutyric acid (GABA) A receptor, alpha 3 [Source:HGNC Symbol;Acc:4077]
rs34210022				
rs73241891				
rs73241893				
rs10856240				
rs17326876				
rs5969901				
rs146310368				
rs5970335				
rs62610449				
rs7886003				
rs73628576				
rs5925188				
rs73244112				
rs41313408	X;HG1497_PATCH	GABRQ;AC244102.1	HGNC Symbol;Clone-based (Ensembl) gene	gamma-aminobutyric acid (GABA) A receptor, theta [Source:HGNC Symbol;Acc:14454];
rs3810651	X;HG1497_PATCH	GABRQ;AC244102.1	HGNC Symbol;Clone-based (Ensembl) gene	gamma-aminobutyric acid (GABA) A receptor, theta [Source:HGNC Symbol;Acc:14454];
rs5924754				
rs4828707				
rs60077659				
rs2515837	X;HG1497_PATCH	CSAG1	HGNC Symbol	chondrosarcoma associated gene 1 [Source:HGNC Symbol;Acc:24294]
rs2515839	X;HG1497_PATCH	CSAG1	HGNC Symbol	chondrosarcoma associated gene 1 [Source:HGNC Symbol;Acc:24294]
rs2515848	X;HG1497_PATCH	CSAG1	HGNC Symbol	chondrosarcoma associated gene 1 [Source:HGNC Symbol;Acc:24294]
rs56330201				
rs12687892				

SNP	Chromosome	gene name	gene source	description
rs5925233				
rs6526129				
rs6627683				
rs5970386				
rs17320422				
rs3827416	X;HG1497_PATCH	NSDHL	HGNC Symbol	NAD(P) dependent steroid dehydrogenase-like [Source:HGNC Symbol;Acc:13398]
rs6526131	X;HG1497_PATCH	NSDHL	HGNC Symbol	NAD(P) dependent steroid dehydrogenase-like [Source:HGNC Symbol;Acc:13398]
rs17320429	X;HG1497_PATCH	NSDHL	HGNC Symbol	NAD(P) dependent steroid dehydrogenase-like [Source:HGNC Symbol;Acc:13398]
rs12845949	X;HG1497_PATCH	NSDHL	HGNC Symbol	NAD(P) dependent steroid dehydrogenase-like [Source:HGNC Symbol;Acc:13398]
rs11797556	X;HG1497_PATCH	NSDHL	HGNC Symbol	NAD(P) dependent steroid dehydrogenase-like [Source:HGNC Symbol;Acc:13398]
rs56344265	X;HG1497_PATCH	NSDHL	HGNC Symbol	NAD(P) dependent steroid dehydrogenase-like [Source:HGNC Symbol;Acc:13398]
rs12558405	X;HG1497_PATCH	NSDHL	HGNC Symbol	NAD(P) dependent steroid dehydrogenase-like [Source:HGNC Symbol;Acc:13398]
rs6627696				
rs6526138				
rs17327065				
rs57372578				
rs73244132				
rs2301191				
rs12012932				
rs5925245				
rs6526141	X;HG1497_PATCH	ZNF185	HGNC Symbol	zinc finger protein 185 (LIM domain) [Source:HGNC Symbol;Acc:12976]
rs6526142	X;HG1497_PATCH	ZNF185	HGNC Symbol	zinc finger protein 185 (LIM domain) [Source:HGNC Symbol;Acc:12976]
rs736654	X;HG1497_PATCH	ZNF185	HGNC Symbol	zinc finger protein 185 (LIM domain) [Source:HGNC Symbol;Acc:12976]
rs113528928	X;HG1497_PATCH	ZNF185	HGNC Symbol	zinc finger protein 185 (LIM domain) [Source:HGNC Symbol;Acc:12976]
rs56268527	X;HG1497_PATCH	ZNF185	HGNC Symbol	zinc finger protein 185 (LIM domain) [Source:HGNC Symbol;Acc:12976]

SNP	Chromosome	gene name	gene source	description
rs6418510	X;HG1497_PATCH	ZNF185	HGNC Symbol	zinc finger protein 185 (LIM domain) [Source:HGNC Symbol;Acc:12976]
rs6653491	X;HG1497_PATCH	ZNF185	HGNC Symbol	zinc finger protein 185 (LIM domain) [Source:HGNC Symbol;Acc:12976]
rs5969924	X;HG1497_PATCH	ZNF185	HGNC Symbol	zinc finger protein 185 (LIM domain) [Source:HGNC Symbol;Acc:12976]
rs62593810	X;HG1497_PATCH	ZNF185	HGNC Symbol	zinc finger protein 185 (LIM domain) [Source:HGNC Symbol;Acc:12976]
rs2071262	X;HG1497_PATCH	ZNF185	HGNC Symbol	zinc finger protein 185 (LIM domain) [Source:HGNC Symbol;Acc:12976]
rs62593813	X;HG1497_PATCH	ZNF185	HGNC Symbol	zinc finger protein 185 (LIM domain) [Source:HGNC Symbol;Acc:12976]
rs5970400	X;HG1497_PATCH	ZNF185	HGNC Symbol	zinc finger protein 185 (LIM domain) [Source:HGNC Symbol;Acc:12976]
rs5925266	X;HG1497_PATCH	ZNF185	HGNC Symbol	zinc finger protein 185 (LIM domain) [Source:HGNC Symbol;Acc:12976]
rs11582<br>rs5925274<br>rs56066727<br>rs34913928	X;HG1497_PATCH	ZNF185	HGNC Symbol	zinc finger protein 185 (LIM domain) [Source:HGNC Symbol;Acc:12976]
rs56350208	X;HG1497_PATCH	PNMA5	HGNC Symbol	paraneoplastic Ma antigen family member 5 [Source:HGNC Symbol;Acc:18743]
rs146175766	X;HG1497_PATCH	PNMA5	HGNC Symbol	paraneoplastic Ma antigen family member 5 [Source:HGNC Symbol;Acc:18743]
rs76024752<br>rs5970407<br>rs1034376<br>rs7051108<br>rs1023434<br>rs112545839<br>rs75065001<br>rs5925321	X;HG1497_PATCH	PNMA5	HGNC Symbol	paraneoplastic Ma antigen family member 5 [Source:HGNC Symbol;Acc:18743]
rs6526155	X;HG1497_PATCH	PNMA3;AC243428.1	HGNC Symbol;Clone-based (Ensembl) gene	paraneoplastic Ma antigen 3 [Source:HGNC Symbol;Acc:18742];

SNP	Chromosome	gene name	gene source	description
rs5970424	X;HG1497_PATCH	PNMA3;AC243428.1	HGNC Symbol;Clone-based (Ensembl) gene	paraneoplastic Ma antigen 3 [Source:HGNC Symbol;Acc:18742];
rs1045069	X	PNMA3	HGNC Symbol	paraneoplastic Ma antigen 3 [Source:HGNC Symbol;Acc:18742]
rs62593668				
rs73245844				
rs5969947				
rs76731387				
rs2008144	X;HG1497_PATCH	MAGEA1	HGNC Symbol;UniProtKB Gene Name	melanoma antigen family A, 1 (directs expression of antigen MZ2-E) [Source:HGNC Symbol;Acc:6796];Melanoma-associated antigen 1 [Source:UniProtKB/Swiss-Prot;Acc:P43355]
rs2233044	X;HG1497_PATCH	MAGEA1	HGNC Symbol;UniProtKB Gene Name	melanoma antigen family A, 1 (directs expression of antigen MZ2-E) [Source:HGNC Symbol;Acc:6796];Melanoma-associated antigen 1 [Source:UniProtKB/Swiss-Prot;Acc:P43355]
rs12556259				
rs5925342				
rs59726447				
rs150769228				
rs58707270				
rs6627772				
rs61007290				
rs73245852				
rs5970440				
rs11094626				
rs148062047				
rs112937928	X;HG1497_PATCH	ZNF275	HGNC Symbol	zinc finger protein 275 [Source:HGNC Symbol;Acc:13069]
rs3213466	X;HG1497_PATCH	ZNF275	HGNC Symbol	zinc finger protein 275 [Source:HGNC Symbol;Acc:13069]
rs143775675	X;HG1497_PATCH	ZNF275	HGNC Symbol	zinc finger protein 275 [Source:HGNC Symbol;Acc:13069]
rs62596388				
rs66716612				
rs72616432				
rs12015993				

SNP	Chromosome	gene name	gene source	description
rs12400823				
rs9782761				
rs5987197				
rs12014659				
rs12689162				
rs58991246				
rs5945320				
rs5986916	X;HG1497_PATCH	ZFP92	HGNC Symbol	ZFP92 zinc finger protein [Source:HGNC Symbol;Acc:12865]
rs73632757				
rs73245880				
rs928952				
rs5987163				
rs7059650				
rs58070852	X;HG1497_PATCH	TREX2;HAUS7	HGNC Symbol	three prime repair exonuclease 2 [Source:HGNC Symbol;Acc:12270];HAUS augmin-like complex, subunit 7 [Source:HGNC Symbol;Acc:32979]
rs41311378	X	TREX2;HAUS7	HGNC Symbol	three prime repair exonuclease 2 [Source:HGNC Symbol;Acc:12270];HAUS augmin-like complex, subunit 7 [Source:HGNC Symbol;Acc:32979]
rs5945389	X;HG1497_PATCH	TREX2;HAUS7	HGNC Symbol	three prime repair exonuclease 2 [Source:HGNC Symbol;Acc:12270];HAUS augmin-like complex, subunit 7 [Source:HGNC Symbol;Acc:32979]
rs762736	X;HG1497_PATCH	TREX2;HAUS7	HGNC Symbol	three prime repair exonuclease 2 [Source:HGNC Symbol;Acc:12270];HAUS augmin-like complex, subunit 7 [Source:HGNC Symbol;Acc:32979]
rs2032436	X;HG1497_PATCH	TREX2;HAUS7	HGNC Symbol	three prime repair exonuclease 2 [Source:HGNC Symbol;Acc:12270];HAUS augmin-like complex, subunit 7 [Source:HGNC Symbol;Acc:32979]
rs12687112	X;HG1497_PATCH	HAUS7	HGNC Symbol	HAUS augmin-like complex, subunit 7 [Source:HGNC Symbol;Acc:32979]
rs3020973	X;HG1497_PATCH	HAUS7	HGNC Symbol	HAUS augmin-like complex, subunit 7 [Source:HGNC Symbol;Acc:32979]
rs147237990	X;HG1497_PATCH	HAUS7	HGNC Symbol	HAUS augmin-like complex, subunit 7 [Source:HGNC Symbol;Acc:32979]
rs3020975	X;HG1497_PATCH	HAUS7	HGNC Symbol	HAUS augmin-like complex, subunit 7 [Source:HGNC Symbol;Acc:32979]
rs11796997	X;HG1497_PATCH	HAUS7	HGNC Symbol	HAUS augmin-like complex, subunit 7 [Source:HGNC Symbol;Acc:32979]
rs7055188	X;HG1497_PATCH	BGN	HGNC Symbol	biglycan [Source:HGNC Symbol;Acc:1044]
rs2980051	X;HG1497_PATCH	BGN	HGNC Symbol	biglycan [Source:HGNC Symbol;Acc:1044]

SNP	Chromosome	gene name	gene source	description
rs4833	X;HG1497_PATCH	BGN	HGNC Symbol	biglycan [Source:HGNC Symbol;Acc:1044]
rs1126499	X;HG1497_PATCH	BGN	HGNC Symbol	biglycan [Source:HGNC Symbol;Acc:1044]
rs2070933	X;HG1497_PATCH	BGN	HGNC Symbol	biglycan [Source:HGNC Symbol;Acc:1044]
rs5945215	X;HG1497_PATCH	BGN	HGNC Symbol	biglycan [Source:HGNC Symbol;Acc:1044]
rs2980060				
rs2980075	X;HG1497_PATCH	ATP2B3	HGNC Symbol	ATPase, Ca++ transporting, plasma membrane 3 [Source:HGNC Symbol;Acc:816]
rs3020942	X;HG1497_PATCH	ATP2B3	HGNC Symbol	ATPase, Ca++ transporting, plasma membrane 3 [Source:HGNC Symbol;Acc:816]
rs2285034	X;HG1497_PATCH	ATP2B3	HGNC Symbol	ATPase, Ca++ transporting, plasma membrane 3 [Source:HGNC Symbol;Acc:816]
rs73245900	X;HG1497_PATCH	ATP2B3	HGNC Symbol	ATPase, Ca++ transporting, plasma membrane 3 [Source:HGNC Symbol;Acc:816]
rs2269415	X;HG1497_PATCH	ATP2B3	HGNC Symbol	ATPase, Ca++ transporting, plasma membrane 3 [Source:HGNC Symbol;Acc:816]
rs5945144	X;HG1497_PATCH	ATP2B3	HGNC Symbol	ATPase, Ca++ transporting, plasma membrane 3 [Source:HGNC Symbol;Acc:816]
rs4898419	X;HG1497_PATCH	ATP2B3	HGNC Symbol	ATPase, Ca++ transporting, plasma membrane 3 [Source:HGNC Symbol;Acc:816]
rs6643626	X;HG1497_PATCH	ATP2B3	HGNC Symbol	ATPase, Ca++ transporting, plasma membrane 3 [Source:HGNC Symbol;Acc:816]
rs4413960	X;HG1497_PATCH	ATP2B3	HGNC Symbol	ATPase, Ca++ transporting, plasma membrane 3 [Source:HGNC Symbol;Acc:816]
rs12835731	X;HG1497_PATCH	ATP2B3	HGNC Symbol	ATPase, Ca++ transporting, plasma membrane 3 [Source:HGNC Symbol;Acc:816]
rs146584290	X;HG1497_PATCH	ATP2B3	HGNC Symbol	ATPase, Ca++ transporting, plasma membrane 3 [Source:HGNC Symbol;Acc:816]
rs3817718	X;HG1497_PATCH	FAM58A	HGNC Symbol;UniProtKB Gene Name	family with sequence similarity 58, member A [Source:HGNC Symbol;Acc:28434];Cyclin-related protein FAM58A [Source:UniProtKB/Swiss-Prot;Acc:Q8N1B3]
rs12010175	X;HG1497_PATCH	FAM58A	HGNC Symbol;UniProtKB Gene Name	family with sequence similarity 58, member A [Source:HGNC Symbol;Acc:28434];Cyclin-related protein FAM58A [Source:UniProtKB/Swiss-Prot;Acc:Q8N1B3]
rs138958346	X;HG1497_PATCH	RP11-66N11.8	Clone-based (Vega) gene	

SNP	Chromosome	gene name	gene source	description
rs137887354	X;HG1497_PATCH	RP11-66N11.8	Clone-based (Vega) gene	
rs12835386	X;HG1497_PATCH	RP11-66N11.8	Clone-based (Vega) gene	
rs12860855				
rs5987111				
rs12852467				
rs17091307				
rs5945326				
rs7053640				
rs191356512				
rs5945330				
rs6571284				
rs6643636				
rs7881637				
rs5987125				
rs59599529				
rs5945337				
rs12848840	X;HG1497_PATCH	PNCK	HGNC Symbol	pregnancy up-regulated nonubiquitous CaM kinase [Source:HGNC Symbol;Acc:13415]
rs5987128	X;HG1497_PATCH	PNCK	HGNC Symbol	pregnancy up-regulated nonubiquitous CaM kinase [Source:HGNC Symbol;Acc:13415]
rs6643760	X;HG1497_PATCH	PNCK	HGNC Symbol	pregnancy up-regulated nonubiquitous CaM kinase [Source:HGNC Symbol;Acc:13415]
rs2071028	X;HG1497_PATCH	SLC6A8	HGNC Symbol	solute carrier family 6 (neurotransmitter transporter), member 8 [Source:HGNC Symbol;Acc:11055]
rs4148030	X	ABCD1	HGNC Symbol	ATP-binding cassette, sub-family D (ALD), member 1 [Source:HGNC Symbol;Acc:61]
rs80276708	X;HG1497_PATCH	ABCD1	HGNC Symbol	ATP-binding cassette, sub-family D (ALD), member 1 [Source:HGNC Symbol;Acc:61]
rs139176795	X;HG1497_PATCH	ABCD1	HGNC Symbol	ATP-binding cassette, sub-family D (ALD), member 1 [Source:HGNC Symbol;Acc:61]
rs11156606	X;HG1497_PATCH	ABCD1;U52111.14	HGNC Symbol;Clone-based (Vega) gene	ATP-binding cassette, sub-family D (ALD), member 1 [Source:HGNC Symbol;Acc:61];
rs2266876	X;HG1497_PATCH	ABCD1;U52111.14	HGNC Symbol;Clone-based (Vega) gene	ATP-binding cassette, sub-family D (ALD), member 1 [Source:HGNC Symbol;Acc:61];

SNP	Chromosome	gene name	gene source	description
rs6643785	X	U52111.14	Clone-based (Vega) gene	
rs4898437	X;HG1497_PATCH	U52111.14	Clone-based (Vega) gene	
rs4898439	X;HG1497_PATCH	U52111.14	Clone-based (Vega) gene	
rs2266879	X;HG1497_PATCH	PLXNB3	HGNC Symbol	plexin B3 [Source:HGNC Symbol;Acc:9105]
rs6643790	X;HG1497_PATCH	PLXNB3	HGNC Symbol	plexin B3 [Source:HGNC Symbol;Acc:9105]
rs2266882	X;HG1497_PATCH	PLXNB3	HGNC Symbol	plexin B3 [Source:HGNC Symbol;Acc:9105]
rs5987155	X;HG1497_PATCH	PLXNB3	HGNC Symbol	plexin B3 [Source:HGNC Symbol;Acc:9105]
rs146832392	X;HG1497_PATCH	SRPK3;PLXNB3	HGNC Symbol	SRSF protein kinase 3 [Source:HGNC Symbol;Acc:11402];plexin B3 [Source:HGNC Symbol;Acc:9105]
rs762651	X;HG1497_PATCH	SRPK3	HGNC Symbol	SRSF protein kinase 3 [Source:HGNC Symbol;Acc:11402]
rs1981439	X;HG1497_PATCH	SRPK3	HGNC Symbol	SRSF protein kinase 3 [Source:HGNC Symbol;Acc:11402]
rs55812039	X;HG1497_PATCH	SRPK3	HGNC Symbol	SRSF protein kinase 3 [Source:HGNC Symbol;Acc:11402]
rs1802092	X;HG1497_PATCH	SRPK3	HGNC Symbol	SRSF protein kinase 3 [Source:HGNC Symbol;Acc:11402]
rs17431	X;HG1497_PATCH	IDH3G	HGNC Symbol	isocitrate dehydrogenase 3 (NAD+) gamma [Source:HGNC Symbol;Acc:5386]
rs2283752	X;HG1497_PATCH	IDH3G	HGNC Symbol	isocitrate dehydrogenase 3 (NAD+) gamma [Source:HGNC Symbol;Acc:5386]
rs5945165	X;HG1497_PATCH	SSR4	HGNC Symbol	signal sequence receptor, delta [Source:HGNC Symbol;Acc:11326]
rs4898447				
rs2269367	X;HG1497_PATCH	PDZD4	HGNC Symbol	PDZ domain containing 4 [Source:HGNC Symbol;Acc:21167]
rs3747309	X;HG1497_PATCH	PDZD4	HGNC Symbol	PDZ domain containing 4 [Source:HGNC Symbol;Acc:21167]
rs142879053				
rs12394846				
rs60849638				
rs5987171				

SNP	Chromosome	gene name	gene source	description
rs4646265	X;HG1497_PATCH	L1CAM	HGNC Symbol	L1 cell adhesion molecule [Source:HGNC Symbol;Acc:6470]
rs149814383	X;HG1497_PATCH	L1CAM	HGNC Symbol	L1 cell adhesion molecule [Source:HGNC Symbol;Acc:6470]
rs4243542	X;HG1497_PATCH	L1CAM	HGNC Symbol	L1 cell adhesion molecule [Source:HGNC Symbol;Acc:6470]
rs4646263	X;HG1497_PATCH	L1CAM	HGNC Symbol	L1 cell adhesion molecule [Source:HGNC Symbol;Acc:6470]
rs142085600	X;HG1497_PATCH	LCA10;L1CAM	UniProtKB Gene Name;HGNC Symbol	Putative lung carcinoma-associated protein 10 [Source:UniProtKB/Swiss-Prot;Acc:Q71F78];L1 cell adhesion molecule [Source:HGNC Symbol;Acc:6470]
rs12380966	X;HG1497_PATCH	L1CAM	HGNC Symbol	L1 cell adhesion molecule [Source:HGNC Symbol;Acc:6470]
rs12559136	X;HG1497_PATCH	AVPR2;L1CAM	HGNC Symbol	arginine vasopressin receptor 2 [Source:HGNC Symbol;Acc:897];L1 cell adhesion molecule [Source:HGNC Symbol;Acc:6470]
rs3761529	X;HG1497_PATCH	AVPR2;L1CAM	HGNC Symbol	arginine vasopressin receptor 2 [Source:HGNC Symbol;Acc:897];L1 cell adhesion molecule [Source:HGNC Symbol;Acc:6470]
rs3761527	X;HG1497_PATCH	AVPR2;L1CAM	HGNC Symbol	arginine vasopressin receptor 2 [Source:HGNC Symbol;Acc:897];L1 cell adhesion molecule [Source:HGNC Symbol;Acc:6470]
rs4898372	X;HG1497_PATCH	AVPR2;L1CAM	HGNC Symbol	arginine vasopressin receptor 2 [Source:HGNC Symbol;Acc:897];L1 cell adhesion molecule [Source:HGNC Symbol;Acc:6470]
rs5201	X;HG1497_PATCH	AVPR2;L1CAM	HGNC Symbol	arginine vasopressin receptor 2 [Source:HGNC Symbol;Acc:897];L1 cell adhesion molecule [Source:HGNC Symbol;Acc:6470]
rs5202	X	AVPR2;L1CAM	HGNC Symbol	arginine vasopressin receptor 2 [Source:HGNC Symbol;Acc:897];L1 cell adhesion molecule [Source:HGNC Symbol;Acc:6470]
rs2070099	X;HG1497_PATCH	ARHGAP4	HGNC Symbol	Rho GTPase activating protein 4 [Source:HGNC Symbol;Acc:674]
rs2070097	X;HG1497_PATCH	ARHGAP4	HGNC Symbol	Rho GTPase activating protein 4 [Source:HGNC Symbol;Acc:674]
rs2071128	X;HG1497_PATCH	ARHGAP4;NAA10	HGNC Symbol	Rho GTPase activating protein 4 [Source:HGNC Symbol;Acc:674];N(alpha)-acetyltransferase 10, NatA catalytic subunit [Source:HGNC Symbol;Acc:18704]
rs2071130	X;HG1497_PATCH	ARHGAP4;NAA10	HGNC Symbol	Rho GTPase activating protein 4 [Source:HGNC Symbol;Acc:674];N(alpha)-acetyltransferase 10, NatA catalytic subunit [Source:HGNC Symbol;Acc:18704]
rs2156929	X;HG1497_PATCH	RENBP	HGNC Symbol	renin binding protein [Source:HGNC Symbol;Acc:9959]
rs3027875	X	HCFC1	HGNC Symbol	host cell factor C1 (VP16-accessory protein) [Source:HGNC Symbol;Acc:4839]
rs2071133	X;HG1497_PATCH	HCFC1	HGNC Symbol	host cell factor C1 (VP16-accessory protein) [Source:HGNC Symbol;Acc:4839]
rs1051152	X;HG1497_PATCH	HCFC1	HGNC Symbol	host cell factor C1 (VP16-accessory protein) [Source:HGNC Symbol;Acc:4839]

SNP	Chromosome	gene name	gene source	description
rs2071134	X;HG1497_PATCH	HCFC1	HGNC Symbol	host cell factor C1 (VP16-accessory protein) [Source:HGNC Symbol;Acc:4839]
rs73247640	X;HG1497_PATCH	HCFC1	HGNC Symbol	host cell factor C1 (VP16-accessory protein) [Source:HGNC Symbol;Acc:4839]
rs2266890	X;HG1497_PATCH	TMEM187	HGNC Symbol	transmembrane protein 187 [Source:HGNC Symbol;Acc:13705]
rs6571303	X;HG1497_PATCH	TMEM187	HGNC Symbol	transmembrane protein 187 [Source:HGNC Symbol;Acc:13705]
rs13397	X;HG1497_PATCH	TMEM187	HGNC Symbol	transmembrane protein 187 [Source:HGNC Symbol;Acc:13705]
rs11465839	X;HG1497_PATCH	IRAK1	HGNC Symbol	interleukin-1 receptor-associated kinase 1 [Source:HGNC Symbol;Acc:6112]
rs1059702	X;HG1497_PATCH	IRAK1	HGNC Symbol	interleukin-1 receptor-associated kinase 1 [Source:HGNC Symbol;Acc:6112]
rs3027915	X;HG1497_PATCH	MECP2	HGNC Symbol	methyl CpG binding protein 2 (Rett syndrome) [Source:HGNC Symbol;Acc:6990]
rs2734647	X;HG1497_PATCH	MECP2	HGNC Symbol	methyl CpG binding protein 2 (Rett syndrome) [Source:HGNC Symbol;Acc:6990]
rs3027924	X;HG1497_PATCH	MECP2	HGNC Symbol	methyl CpG binding protein 2 (Rett syndrome) [Source:HGNC Symbol;Acc:6990]
rs3027928	X;HG1497_PATCH	MECP2	HGNC Symbol	methyl CpG binding protein 2 (Rett syndrome) [Source:HGNC Symbol;Acc:6990]
rs17435	X;HG1497_PATCH	MECP2	HGNC Symbol	methyl CpG binding protein 2 (Rett syndrome) [Source:HGNC Symbol;Acc:6990]
rs1734787	X;HG1497_PATCH	MECP2	HGNC Symbol	methyl CpG binding protein 2 (Rett syndrome) [Source:HGNC Symbol;Acc:6990]
rs1734791	X	MECP2	HGNC Symbol	methyl CpG binding protein 2 (Rett syndrome) [Source:HGNC Symbol;Acc:6990]
rs5945175	X;HG1497_PATCH	MECP2	HGNC Symbol	methyl CpG binding protein 2 (Rett syndrome) [Source:HGNC Symbol;Acc:6990]
rs147650189				
rs1316038				
rs6655245				
rs7884181				
rs5987215				
rs1573656				
rs10126322	X;HG1497_PATCH	TKTL1	HGNC Symbol	transketolase-like 1 [Source:HGNC Symbol;Acc:11835]
rs17281251	X;HG1497_PATCH	TKTL1	HGNC Symbol	transketolase-like 1 [Source:HGNC Symbol;Acc:11835]

SNP	Chromosome	gene name	gene source	description
rs5986969	X;HG1497_PATCH	TKTL1	HGNC Symbol	transketolase-like 1 [Source:HGNC Symbol;Acc:11835]
rs766420	X;HG1497_PATCH	TKTL1	HGNC Symbol	transketolase-like 1 [Source:HGNC Symbol;Acc:11835]
rs2872817	X;HG1497_PATCH	TKTL1	HGNC Symbol	transketolase-like 1 [Source:HGNC Symbol;Acc:11835]
rs2239469	X;HG1497_PATCH	FLNA	HGNC Symbol	filamin A, alpha [Source:HGNC Symbol;Acc:3754]
rs36051194	X;HG1497_PATCH	FLNA	HGNC Symbol	filamin A, alpha [Source:HGNC Symbol;Acc:3754]
rs146923904	X;HG1497_PATCH	DNASE1L1;RPL10	HGNC Symbol	deoxyribonuclease I-like 1 [Source:HGNC Symbol;Acc:2957];ribosomal protein L10 [Source:HGNC Symbol;Acc:10298]
rs34952165	X;HG1497_PATCH	DNASE1L1;RPL10	HGNC Symbol	deoxyribonuclease I-like 1 [Source:HGNC Symbol;Acc:2957];ribosomal protein L10 [Source:HGNC Symbol;Acc:10298]
rs1130929	X;HG1497_PATCH	DNASE1L1;RPL10	HGNC Symbol	deoxyribonuclease I-like 1 [Source:HGNC Symbol;Acc:2957];ribosomal protein L10 [Source:HGNC Symbol;Acc:10298]
rs743549				
rs28497482	X	ATP6AP1	HGNC Symbol	ATPase, H+ transporting, lysosomal accessory protein 1 [Source:HGNC Symbol;Acc:868]
rs4898495	X	ATP6AP1	HGNC Symbol	ATPase, H+ transporting, lysosomal accessory protein 1 [Source:HGNC Symbol;Acc:868]
rs762514	X;HG1497_PATCH	FAM50A	HGNC Symbol	family with sequence similarity 50, member A [Source:HGNC Symbol;Acc:18786]
rs35285799	X;HG1497_PATCH	PLXNA3	HGNC Symbol	plexin A3 [Source:HGNC Symbol;Acc:9101]
rs5945429	X;HG1497_PATCH	PLXNA3	HGNC Symbol	plexin A3 [Source:HGNC Symbol;Acc:9101]
rs5945430	X;HG1497_PATCH	PLXNA3	HGNC Symbol	plexin A3 [Source:HGNC Symbol;Acc:9101]
rs60361517	X;HG1497_PATCH	PLXNA3	HGNC Symbol	plexin A3 [Source:HGNC Symbol;Acc:9101]
rs5987266	X;HG1497_PATCH	PLXNA3	HGNC Symbol	plexin A3 [Source:HGNC Symbol;Acc:9101]
rs6567	X;HG1497_PATCH	LAGE3	HGNC Symbol;UniProtKB Gene Name	L antigen family, member 3 [Source:HGNC Symbol;Acc:26058];L antigen family member 3 [Source:UniProtKB/TrEMBL;Acc:B0S8I7]
rs5945432				
rs7057286	X;HG1497_PATCH	UBL4A	HGNC Symbol	ubiquitin-like 4A [Source:HGNC Symbol;Acc:12505]
rs12392447	HG1497_PATCH;X	RN7SL697P	HGNC Symbol	RNA, 7SL, cytoplasmic 697, pseudogene [Source:HGNC Symbol;Acc:46713]

SNP	Chromosome	gene name	gene source	description
rs5986987	X;HG1497_PATCH	FAM3A	HGNC Symbol;UniProtKB Gene Name	family with sequence similarity 3, member A [Source:HGNC Symbol;Acc:13749];
rs73247656				
rs2230037	X;HG1497_PATCH	G6PD	HGNC Symbol	glucose-6-phosphate dehydrogenase [Source:HGNC Symbol;Acc:4057]
rs743544	X;HG1497_PATCH	G6PD	HGNC Symbol	glucose-6-phosphate dehydrogenase [Source:HGNC Symbol;Acc:4057]
rs62617845	X;HG1497_PATCH	G6PD	HGNC Symbol	glucose-6-phosphate dehydrogenase [Source:HGNC Symbol;Acc:4057]
rs150358028	X;HG1497_PATCH	IKBKG	HGNC Symbol;UniProtKB Gene Name	inhibitor of kappa light polypeptide gene enhancer in B-cells, kinase gamma [Source:HGNC Symbol;Acc:5961];Inhibitor of kappa light polypeptide gene enhancer in B-cells, kinase gamma, isoform CRA_b [Source:UniProtKB/TrEMBL;Acc:D3DWY0]
rs5986877				
rs4326559	X;HG1497_PATCH	CTAG2	HGNC Symbol	cancer/testis antigen 2 [Source:HGNC Symbol;Acc:2492]
rs3813455	X;HG1497_PATCH	GAB3	HGNC Symbol	GRB2-associated binding protein 3 [Source:HGNC Symbol;Acc:17515]
rs5945108	X;HG1497_PATCH	GAB3	HGNC Symbol	GRB2-associated binding protein 3 [Source:HGNC Symbol;Acc:17515]
rs4431759	X;HG1497_PATCH	GAB3	HGNC Symbol	GRB2-associated binding protein 3 [Source:HGNC Symbol;Acc:17515]
rs112527470	X;HG1497_PATCH	GAB3	HGNC Symbol	GRB2-associated binding protein 3 [Source:HGNC Symbol;Acc:17515]
rs6643615	X	GAB3	HGNC Symbol	GRB2-associated binding protein 3 [Source:HGNC Symbol;Acc:17515]
rs17281349	X	GAB3	HGNC Symbol	GRB2-associated binding protein 3 [Source:HGNC Symbol;Acc:17515]
rs2664170	X;HG1497_PATCH	GAB3	HGNC Symbol	GRB2-associated binding protein 3 [Source:HGNC Symbol;Acc:17515]
rs1127051	X;HG1497_PATCH	DKC1	HGNC Symbol	dyskeratosis congenita 1, dyskerin [Source:HGNC Symbol;Acc:2890]
rs41299134	X	DKC1	HGNC Symbol	dyskeratosis congenita 1, dyskerin [Source:HGNC Symbol;Acc:2890]
rs112027316	X;HG1497_PATCH	MPP1	HGNC Symbol	membrane protein, palmitoylated 1, 55kDa [Source:HGNC Symbol;Acc:7219]
rs1848762	X	MPP1	HGNC Symbol	membrane protein, palmitoylated 1, 55kDa [Source:HGNC Symbol;Acc:7219]
rs6643620	X;HG1497_PATCH	SMIM9	HGNC Symbol	small integral membrane protein 9 [Source:HGNC Symbol;Acc:41915]
rs1050705	X;HG1497_PATCH	F8	HGNC Symbol	coagulation factor VIII, procoagulant component [Source:HGNC Symbol;Acc:3546]
rs28370229	X	F8	HGNC Symbol	coagulation factor VIII, procoagulant component [Source:HGNC Symbol;Acc:3546]

SNP	Chromosome	gene name	gene source	description
rs28814617	X;HG1497_PATCH	F8	HGNC Symbol	coagulation factor VIII, procoagulant component [Source:HGNC Symbol;Acc:3546]
rs28370241	X	F8	HGNC Symbol	coagulation factor VIII, procoagulant component [Source:HGNC Symbol;Acc:3546]
rs28370214	X;HG1497_PATCH	F8	HGNC Symbol	coagulation factor VIII, procoagulant component [Source:HGNC Symbol;Acc:3546]
rs1800292	X;HG1497_PATCH	F8	HGNC Symbol	coagulation factor VIII, procoagulant component [Source:HGNC Symbol;Acc:3546]
rs1800291	X;HG1497_PATCH	F8	HGNC Symbol	coagulation factor VIII, procoagulant component [Source:HGNC Symbol;Acc:3546]
rs17281398	X;HG1497_PATCH	F8	HGNC Symbol	coagulation factor VIII, procoagulant component [Source:HGNC Symbol;Acc:3546]
rs4898406	X;HG1497_PATCH	FUNDC2	HGNC Symbol	FUN14 domain containing 2 [Source:HGNC Symbol;Acc:24925]
rs62619847	X;HG1497_PATCH	FUNDC2	HGNC Symbol	FUN14 domain containing 2 [Source:HGNC Symbol;Acc:24925]
rs5945286	X;HG1497_PATCH	BRCC3;MTCP1	HGNC Symbol	BRCA1/BRCA2-containing complex, subunit 3 [Source:HGNC Symbol;Acc:24185];mature T-cell proliferation 1 [Source:HGNC Symbol;Acc:7423]
rs895745	X;HG1497_PATCH	BRCC3;MTCP1	HGNC Symbol	BRCA1/BRCA2-containing complex, subunit 3 [Source:HGNC Symbol;Acc:24185];mature T-cell proliferation 1 [Source:HGNC Symbol;Acc:7423]
rs113038688				
rs4893072	X;HG1497_PATCH	VBP1	HGNC Symbol	von Hippel-Lindau binding protein 1 [Source:HGNC Symbol;Acc:12662]
rs150901018	X;HG1497_PATCH	VBP1	HGNC Symbol	von Hippel-Lindau binding protein 1 [Source:HGNC Symbol;Acc:12662]
rs475025				
rs41311710				
rs5983697	X	RAB39B	HGNC Symbol	RAB39B, member RAS oncogene family [Source:HGNC Symbol;Acc:16499]
rs140657165				
rs559165	X;HG1497_PATCH	CLIC2	HGNC Symbol	chloride intracellular channel 2 [Source:HGNC Symbol;Acc:2063]
rs113313431	X;HG1497_PATCH	CLIC2	HGNC Symbol	chloride intracellular channel 2 [Source:HGNC Symbol;Acc:2063]
rs28826658				
rs28790888				
rs28448813				
rs28592896				
rs62618044	X;HG1497_PATCH	TMLHE;TMLHE-AS1	HGNC Symbol	trimethyllysine hydroxylase, epsilon [Source:HGNC Symbol;Acc:18308];TMLHE antisense RNA 1 [Source:HGNC Symbol;Acc:44261]

SNP	Chromosome	gene name	gene source	description
rs7064354	X	TMLHE;TMLHE-AS1	HGNC Symbol	trimethyllysine hydroxylase, epsilon [Source:HGNC Symbol;Acc:18308];TMLHE antisense RNA 1 [Source:HGNC Symbol;Acc:44261]
rs5940536	X;HG1497_PATCH	TMLHE;TMLHE-AS1	HGNC Symbol	trimethyllysine hydroxylase, epsilon [Source:HGNC Symbol;Acc:18308];TMLHE antisense RNA 1 [Source:HGNC Symbol;Acc:44261]
rs5940404	X;HG1497_PATCH	TMLHE	HGNC Symbol	trimethyllysine hydroxylase, epsilon [Source:HGNC Symbol;Acc:18308]
rs561841	X;HG1497_PATCH	TMLHE	HGNC Symbol	trimethyllysine hydroxylase, epsilon [Source:HGNC Symbol;Acc:18308]
rs144293808	X;HG1497_PATCH	TMLHE	HGNC Symbol	trimethyllysine hydroxylase, epsilon [Source:HGNC Symbol;Acc:18308]
rs601290	X;HG1497_PATCH	TMLHE	HGNC Symbol	trimethyllysine hydroxylase, epsilon [Source:HGNC Symbol;Acc:18308]
rs473491				
rs5940560				
rs112437556				
rs306932				
rs306908				
rs3909635				
rs306894				
rs142918084				
rs306890				
rs73249628				
rs73249629				
rs9650962				
rs57915757				
rs781716				
rs17149475				
rs145347644	X	SPRY3	HGNC Symbol	sprouty homolog 3 (Drosophila) [Source:HGNC Symbol;Acc:11271]
rs306885	X	SPRY3	HGNC Symbol	sprouty homolog 3 (Drosophila) [Source:HGNC Symbol;Acc:11271]
rs306886	X	SPRY3	HGNC Symbol	sprouty homolog 3 (Drosophila) [Source:HGNC Symbol;Acc:11271]
rs6642320	X	SPRY3	HGNC Symbol	sprouty homolog 3 (Drosophila) [Source:HGNC Symbol;Acc:11271]
rs306887	X	SPRY3	HGNC Symbol	sprouty homolog 3 (Drosophila) [Source:HGNC Symbol;Acc:11271]
rs306888	X	SPRY3	HGNC Symbol	sprouty homolog 3 (Drosophila) [Source:HGNC Symbol;Acc:11271]
rs306889	X	SPRY3	HGNC Symbol	sprouty homolog 3 (Drosophila) [Source:HGNC Symbol;Acc:11271]
rs17653586	X	SPRY3	HGNC Symbol	sprouty homolog 3 (Drosophila) [Source:HGNC Symbol;Acc:11271]

SNP	Chromosome	gene name	gene source	description
rs55857040	X	SPRY3	HGNC Symbol	sprouty homolog 3 (Drosophila) [Source:HGNC Symbol;Acc:11271]
rs700447	X	SPRY3	HGNC Symbol	sprouty homolog 3 (Drosophila) [Source:HGNC Symbol;Acc:11271]
rs700449	X	SPRY3	HGNC Symbol	sprouty homolog 3 (Drosophila) [Source:HGNC Symbol;Acc:11271]
rs28729587	X	SPRY3	HGNC Symbol	sprouty homolog 3 (Drosophila) [Source:HGNC Symbol;Acc:11271]
rs77442791	X	SPRY3	HGNC Symbol	sprouty homolog 3 (Drosophila) [Source:HGNC Symbol;Acc:11271]
rs700455	X	SPRY3	HGNC Symbol	sprouty homolog 3 (Drosophila) [Source:HGNC Symbol;Acc:11271]
rs306873	X	SPRY3	HGNC Symbol	sprouty homolog 3 (Drosophila) [Source:HGNC Symbol;Acc:11271]
rs781714				
rs28425172				
rs700462				
rs802480				
rs28884873				
rs73234550	X	AMDP1	HGNC Symbol	adenosylmethionine decarboxylase pseudogene 1 [Source:HGNC Symbol;Acc:460]
rs35519384				
rs6567777				
rs5940599				
rs1969622				
rs73234571				
rs34445725				
rs115603343				
rs5940618				
rs56009695	X	VAMP7	HGNC Symbol	vesicle-associated membrane protein 7 [Source:HGNC Symbol;Acc:11486]
rs5983826	X	VAMP7	HGNC Symbol	vesicle-associated membrane protein 7 [Source:HGNC Symbol;Acc:11486]
rs11547143	X	VAMP7	HGNC Symbol	vesicle-associated membrane protein 7 [Source:HGNC Symbol;Acc:11486]
rs73237015				
rs75303099				
rs5940638				
rs2205602				
rs5940657				
rs73237067				
rs3093457	X	IL9R	HGNC Symbol	interleukin 9 receptor [Source:HGNC Symbol;Acc:6030]
rs1883079	X	IL9R	HGNC Symbol	interleukin 9 receptor [Source:HGNC Symbol;Acc:6030]
rs3093535	X	IL9R	HGNC Symbol	interleukin 9 receptor [Source:HGNC Symbol;Acc:6030]

SNP	Chromosome	gene name	gene source	description
rs3093493	X	IL9R	HGNC Symbol	interleukin 9 receptor [Source:HGNC Symbol;Acc:6030]
rs34557243				
rs28494123				
rs28590175				
rs28491545				
rs73174453				
rs6423165				
rs6649864				
rs6649860	X	LL0YNC03-29C1.1	Clone-based (Vega) gene	
rs55815554	X	LL0YNC03-29C1.1	Clone-based (Vega) gene	
rs6649919				
rs6649917				
rs55771472				
rs56328755				
rs34304242				
rs6644964				
rs55770312				
rs6644961				
rs34264278				
rs60672098				
rs28429757				
rs73174474				
rs28669107	X	PLCXD1	HGNC Symbol	phosphatidylinositol-specific phospholipase C, X domain containing 1 [Source:HGNC Symbol;Acc:23148]
rs9785927	X	PLCXD1	HGNC Symbol	phosphatidylinositol-specific phospholipase C, X domain containing 1 [Source:HGNC Symbol;Acc:23148]
rs6644970	X	PLCXD1	HGNC Symbol	phosphatidylinositol-specific phospholipase C, X domain containing 1 [Source:HGNC Symbol;Acc:23148]
rs28510852	X	PLCXD1	HGNC Symbol	phosphatidylinositol-specific phospholipase C, X domain containing 1 [Source:HGNC Symbol;Acc:23148]
rs3936200	X	PLCXD1	HGNC Symbol	phosphatidylinositol-specific phospholipase C, X domain containing 1 [Source:HGNC Symbol;Acc:23148]
rs11556996	X	PLCXD1	HGNC Symbol	phosphatidylinositol-specific phospholipase C, X domain containing 1 [Source:HGNC Symbol;Acc:23148]

SNP	Chromosome	gene name	gene source	description
rs7892580	X	PLCXD1	HGNC Symbol	phosphatidylinositol-specific phospholipase C, X domain containing 1 [Source:HGNC Symbol;Acc:23148]
rs28661974	X	PLCXD1	HGNC Symbol	phosphatidylinositol-specific phospholipase C, X domain containing 1 [Source:HGNC Symbol;Acc:23148]
rs28681152	X	PLCXD1	HGNC Symbol	phosphatidylinositol-specific phospholipase C, X domain containing 1 [Source:HGNC Symbol;Acc:23148]
rs28605266	X	PLCXD1	HGNC Symbol	phosphatidylinositol-specific phospholipase C, X domain containing 1 [Source:HGNC Symbol;Acc:23148]
rs28670196	X	PLCXD1	HGNC Symbol	phosphatidylinositol-specific phospholipase C, X domain containing 1 [Source:HGNC Symbol;Acc:23148]
rs112920828	X	PLCXD1	HGNC Symbol	phosphatidylinositol-specific phospholipase C, X domain containing 1 [Source:HGNC Symbol;Acc:23148]
rs12401270	X	PLCXD1	HGNC Symbol	phosphatidylinositol-specific phospholipase C, X domain containing 1 [Source:HGNC Symbol;Acc:23148]
rs11019	X	PLCXD1	HGNC Symbol	phosphatidylinositol-specific phospholipase C, X domain containing 1 [Source:HGNC Symbol;Acc:23148]
rs28736870	X	GTPBP6	HGNC Symbol	GTP binding protein 6 (putative) [Source:HGNC Symbol;Acc:30189]
rs1140801	X	GTPBP6	HGNC Symbol	GTP binding protein 6 (putative) [Source:HGNC Symbol;Acc:30189]
rs28590518	X	GTPBP6	HGNC Symbol	GTP binding protein 6 (putative) [Source:HGNC Symbol;Acc:30189]
rs1140798	X	GTPBP6	HGNC Symbol	GTP binding protein 6 (putative) [Source:HGNC Symbol;Acc:30189]
rs7499265	X	GTPBP6	HGNC Symbol	GTP binding protein 6 (putative) [Source:HGNC Symbol;Acc:30189]
rs7066272				
rs5948800				
rs28678320				
rs5948797				
rs35167897				
rs35769534				
rs6644990				
rs6603194				
rs1133530	X	PPP2R3B	HGNC Symbol	protein phosphatase 2, regulatory subunit B'', beta [Source:HGNC Symbol;Acc:13417]
rs5989642	X	PPP2R3B	HGNC Symbol	protein phosphatase 2, regulatory subunit B'', beta [Source:HGNC Symbol;Acc:13417]
rs6645102	X	PPP2R3B	HGNC Symbol	protein phosphatase 2, regulatory subunit B'', beta [Source:HGNC Symbol;Acc:13417]
rs2738382	X	PPP2R3B	HGNC Symbol	protein phosphatase 2, regulatory subunit B'', beta [Source:HGNC Symbol;Acc:13417]
rs1133520				
rs2738376				
rs2738373				

SNP	Chromosome	gene name	gene source	description
rs2738360	X	PPP2R3B	HGNC Symbol	protein phosphatase 2, regulatory subunit B'', beta [Source:HGNC Symbol;Acc:13417]
rs2738348	X	PPP2R3B	HGNC Symbol	protein phosphatase 2, regulatory subunit B'', beta [Source:HGNC Symbol;Acc:13417]
rs2738402	X	PPP2R3B	HGNC Symbol	protein phosphatase 2, regulatory subunit B'', beta [Source:HGNC Symbol;Acc:13417]
rs62581489	X	PPP2R3B	HGNC Symbol	protein phosphatase 2, regulatory subunit B'', beta [Source:HGNC Symbol;Acc:13417]
rs2738397	X	PPP2R3B	HGNC Symbol	protein phosphatase 2, regulatory subunit B'', beta [Source:HGNC Symbol;Acc:13417]
rs5987291	X	PPP2R3B	HGNC Symbol	protein phosphatase 2, regulatory subunit B'', beta [Source:HGNC Symbol;Acc:13417]
rs2738319	X	PPP2R3B	HGNC Symbol	protein phosphatase 2, regulatory subunit B'', beta [Source:HGNC Symbol;Acc:13417]
rs6603251	X	PPP2R3B	HGNC Symbol	protein phosphatase 2, regulatory subunit B'', beta [Source:HGNC Symbol;Acc:13417]
rs3813594	X	PPP2R3B	HGNC Symbol	protein phosphatase 2, regulatory subunit B'', beta [Source:HGNC Symbol;Acc:13417]
rs3813593	X	PPP2R3B	HGNC Symbol	protein phosphatase 2, regulatory subunit B'', beta [Source:HGNC Symbol;Acc:13417]
rs17855192	X	PPP2R3B	HGNC Symbol	protein phosphatase 2, regulatory subunit B'', beta [Source:HGNC Symbol;Acc:13417]
rs6645211	X	PPP2R3B	HGNC Symbol	protein phosphatase 2, regulatory subunit B'', beta [Source:HGNC Symbol;Acc:13417]
rs76354719	X	PPP2R3B	HGNC Symbol	protein phosphatase 2, regulatory subunit B'', beta [Source:HGNC Symbol;Acc:13417]
rs4606239	X	PPP2R3B	HGNC Symbol	protein phosphatase 2, regulatory subunit B'', beta [Source:HGNC Symbol;Acc:13417]
rs5987282	X	PPP2R3B	HGNC Symbol	protein phosphatase 2, regulatory subunit B'', beta [Source:HGNC Symbol;Acc:13417]
rs5987274	X	PPP2R3B	HGNC Symbol	protein phosphatase 2, regulatory subunit B'', beta [Source:HGNC Symbol;Acc:13417]
rs73178055	X	PPP2R3B	HGNC Symbol	protein phosphatase 2, regulatory subunit B'', beta [Source:HGNC Symbol;Acc:13417]
rs5987296	X	PPP2R3B	HGNC Symbol	protein phosphatase 2, regulatory subunit B'', beta [Source:HGNC Symbol;Acc:13417]
rs61101135	X	PPP2R3B	HGNC Symbol	protein phosphatase 2, regulatory subunit B'', beta [Source:HGNC Symbol;Acc:13417]
rs73178070				
rs5950778				
rs28507737				
rs7055427				
rs5950673				
rs28566663				
rs28813147				
rs73178080				
rs5991320				
rs4986487				
rs28448638				
rs59940139				
rs28510287				
rs5991325				

SNP	Chromosome	gene name	gene source	description
rs112244883				
rs62585683				
rs34995254				
rs6645044				
rs5950818				
rs4072327				
rs5950819				
rs28479146				
rs57476114				
rs5950824				
rs5950688				
rs113615733				
rs112252601				
rs73617757				
rs5950840				
rs5950841				
rs190252931				
rs28698389				
rs6603179				
rs7473382				
rs5950843				
rs6603185				
rs62586569				
rs59099943				
rs28689799				
rs5991346				
rs5991348				
rs5991186				
rs140665715				
rs5991355				
rs67324389				
rs59962159				
rs111948374				

SNP	Chromosome	gene name	gene source	description
rs28680530				
rs5950849				
rs35167287				
rs28575158				
rs113150534				
rs35513154				
rs28684706				
rs71206124				
rs28599889				
rs56178950				
rs55971604				
rs28540149				
rs6422430				
rs7883029				
rs36020405				
rs73182018				
rs6645078				
rs28491143				
rs5991205				
rs5950699				
rs61080188				
rs5991211				
rs7053464				
rs7057146				
rs5991135				
rs5950701				
rs5991214				
rs28720723				
rs5950704				
rs73182038				
rs5950631				
rs6645103				
rs6645104				

SNP	Chromosome	gene name	gene source	description
rs5991138				
rs113715884				
rs73182046				
rs3935966				
rs78122608				
rs7891378				
rs28473635				
rs67365957				
rs7877381				
rs7472720				
rs67995411				
rs28362121				
rs7063444				
rs73623146				
rs35129074				
rs73623157				
rs66503718				
rs28373840				
rs117038960				
rs5950734	X	FABP5P13	HGNC Symbol	fatty acid binding protein 5 pseudogene 13 [Source:HGNC Symbol;Acc:38717]
rs5950646				
rs73182085				
rs73182093				
rs5950738				
rs28495413				
rs28393688				
rs66600054				
rs5991266				
rs66797270				
rs5991271				
rs111649363				
rs6645155				
rs5950649				

SNP	Chromosome	gene name	gene source	description
rs66609228				
rs5950757				
rs5950764				
rs5950656				
rs5950657				
rs73183946				
rs28428562				
rs7064175				
rs73183957				
rs5950662				
rs28621912				
rs6645165				
rs6645166				
rs73183971				
rs73183972				
rs73183979				
rs73183980				
rs191682679				
rs28502832				
rs73185948				
rs7063613				
rs6422435				
rs5950787				
rs73626522				
rs73626529				
rs187754046				
rs56224925				
rs6645020				
rs5991299				
rs28399836				
rs6645183				
rs6645028				
rs73185993				

SNP	Chromosome	gene name	gene source	description
rs5950790				
rs73616744				
rs6645195				
rs73187956				
rs5950801				
rs6645197				
rs56108581				
rs28491446				
rs28404660				
rs112380992				
rs28754585				
rs28698797				
rs35443706				
rs147389484				
rs28591982				
rs17719268				
rs28639722				
rs17148923				
rs28371904				
rs35099437	X	SHOX	HGNC Symbol	short stature homeobox [Source:HGNC Symbol;Acc:10853]
rs28603143	X	SHOX	HGNC Symbol	short stature homeobox [Source:HGNC Symbol;Acc:10853]
rs28485701	X	SHOX	HGNC Symbol	short stature homeobox [Source:HGNC Symbol;Acc:10853]
rs2239401	X	SHOX	HGNC Symbol	short stature homeobox [Source:HGNC Symbol;Acc:10853]
rs28659914	X	SHOX	HGNC Symbol	short stature homeobox [Source:HGNC Symbol;Acc:10853]
rs28482156	X	SHOX	HGNC Symbol	short stature homeobox [Source:HGNC Symbol;Acc:10853]
rs28451299	X	SHOX	HGNC Symbol	short stature homeobox [Source:HGNC Symbol;Acc:10853]
rs73607261	X	SHOX	HGNC Symbol	short stature homeobox [Source:HGNC Symbol;Acc:10853]
rs28628562	X	SHOX	HGNC Symbol	short stature homeobox [Source:HGNC Symbol;Acc:10853]
rs28430941	X	SHOX	HGNC Symbol	short stature homeobox [Source:HGNC Symbol;Acc:10853]
rs55647352	X	SHOX	HGNC Symbol	short stature homeobox [Source:HGNC Symbol;Acc:10853]
rs28367488	X	SHOX	HGNC Symbol	short stature homeobox [Source:HGNC Symbol;Acc:10853]
rs35507574	X	SHOX	HGNC Symbol	short stature homeobox [Source:HGNC Symbol;Acc:10853]
rs73190324	X	SHOX	HGNC Symbol	short stature homeobox [Source:HGNC Symbol;Acc:10853]

SNP	Chromosome	gene name	gene source	description
rs28664665	X	SHOX	HGNC Symbol	short stature homeobox [Source:HGNC Symbol;Acc:10853]
rs28408302	X	SHOX	HGNC Symbol	short stature homeobox [Source:HGNC Symbol;Acc:10853]
rs28579720	X	SHOX	HGNC Symbol	short stature homeobox [Source:HGNC Symbol;Acc:10853]
rs28459931	X	SHOX	HGNC Symbol	short stature homeobox [Source:HGNC Symbol;Acc:10853]
rs28362085	X	SHOX	HGNC Symbol	short stature homeobox [Source:HGNC Symbol;Acc:10853]
rs28479710	X	SHOX	HGNC Symbol	short stature homeobox [Source:HGNC Symbol;Acc:10853]
rs60077725	X	SHOX	HGNC Symbol	short stature homeobox [Source:HGNC Symbol;Acc:10853]
rs28474687	X	SHOX	HGNC Symbol	short stature homeobox [Source:HGNC Symbol;Acc:10853]
rs28656423				
rs28393031				
rs17148899				
rs17148884				
rs17148877				
rs28430507				
rs28568451				
rs34607933				
rs7052507				
rs7056080				
rs34124310				
rs4480287				
rs28667393				
rs28557182				
rs113944050				
rs55819855				
rs113858926				
rs4911923				
rs28590541				
rs111758114				
rs5988292				
rs5988473				
rs35246118				
rs7049429				
rs5946591				

SNP	Chromosome	gene name	gene source	description
rs7877972				
rs5946592				
rs5946606				
rs4472693				
rs6579694				
rs2037897				
rs5988600				
rs193144068				
rs4911918				
rs5946417				
rs6644553				
rs28673435				
rs28444181				
rs6644324				
rs4911956				
rs4911958				
rs28468600				
rs5988645				
rs67702862				
rs4911959				
rs5946733				
rs74892495				
rs5988649				
rs6644569				
rs62587521				
rs34141906				
rs5946743				
rs2027987				
rs3933418				
rs5988349				
rs28401629				
rs28425572				
rs4489488				

SNP	Chromosome	gene name	gene source	description
rs67021337				
rs28580397				
rs73176343				
rs28396647				
rs73176362				
rs28644170				
rs4300160				
rs5946460				
rs73176377				
rs59125168				
rs4911919				
rs73616287				
rs111593165				
rs5988357				
rs73178307				
rs6644242				
rs4911921				
rs28595610				
rs79970350				
rs73178314				
rs5988260				
rs5988367				
rs5988370				
rs55873209				
rs73178333				
rs7884122				
rs73178337				
rs28420575				
rs6655359				
rs28752004				
rs28393539				
rs59623055				
rs7891265				

SNP	Chromosome	gene name	gene source	description
rs5988392				
rs7889909				
rs4911903				
rs4376659				
rs4603065				
rs73618693				
rs28650169				
rs62587243				
rs28564284				
rs34775104				
rs5988413				
rs7892777				
rs4517284				
rs7059189				
rs5988277				
rs7057720				
rs73178389				
rs73178390				
rs73180407				
rs17537409				
rs6644366				
rs5988279				
rs5946506				
rs6579619				
rs28690766				
rs5988437				
rs17148729				
rs35841281				
rs5988441				
rs5946520				
rs5946521				
rs5946332				
rs5988285				

SNP	Chromosome	gene name	gene source	description
rs6644389				
rs117966248				
rs5946333				
rs5946526				
rs6579631				
rs34187660				
rs7067102				
rs5946536				
rs7052079				
rs5946343				
rs111644144				
rs28780986				
rs5946570				
rs5946353				
rs7885174				
rs5988301				
rs73182370				
rs73182371				
rs150791575				
rs5946581				
rs5946362				
rs58647086				
rs5946584				
rs62585318				
rs5946585				
rs5946586				
rs5946587				
rs17149048				
rs28513639				
rs67825803				
rs5988311				
rs56361554				
rs73184146				

SNP	Chromosome	gene name	gene source	description
rs17460280				
rs117712983				
rs28579639				
rs4078358				
rs73184154				
rs5988315				
rs4078363				
rs17148605				
rs6579656				
rs17539189				
rs6579665				
rs6579672				
rs5988544				
rs7053445				
rs5946608				
rs6579684				
rs5946611				
rs73188058	X;HG480_HG481_ PATCH	RP11-309M23.1	Clone-based (Vega) gene	
rs112303788	X;HG480_HG481_ PATCH	RP11-309M23.1	Clone-based (Vega) gene	
rs5946622	X;HG480_HG481_ PATCH	RP11-309M23.1	Clone-based (Vega) gene	
rs7878830				
rs7067043				
rs5946378				
rs62603456				
rs5023015				
rs73189936				
rs73189938				
rs60244200				
rs7062015				
rs4131911				
rs56055076				

SNP	Chromosome	gene name	gene source	description
rs73625403				
rs28558589				
rs66460782				
rs5988574				
rs28520535				
rs4129148				
rs5946648				
rs5946649				
rs7880794				
rs4468089				
rs149037989				
rs5946660				
rs5946663				
rs73191912				
rs28591455				
rs73191918				
rs5988594				
rs28450278				
rs5988602				
rs145774377				
rs5988607				
rs34836497				
rs5946398				
rs6644316				
rs55789586				
rs6644317				
rs56398889				
rs4567225				
rs73191962				
rs28456356				
rs5026762				
rs73191974				
rs4243991				

SNP	Chromosome	gene name	gene source	description
rs73174204				
rs73611692				
rs5988341				
rs5988627				
rs73611699				
rs66923305				
rs34696711				
rs28669114				
rs35433883				
rs62604753				
rs28377393				
rs68168005				
rs28565195				
rs28593564				
rs28687369				
rs28609570				
rs28708421				
rs111248768				
rs66501566				
rs28558823				
rs66679897				
rs113146239				
rs73614126				
rs36029157				
rs73174270				
rs34453751				
rs66788080				
rs35811834				
rs62605876				
rs67734326				
rs34745620				
rs114538020				
rs34720230				

SNP	Chromosome	gene name	gene source	description
rs62605887				
rs66513155				
rs62605890				
rs67501076				
rs67147760				
rs67039327				
rs191897026				
rs73175705				
rs73175706				
rs35094631				
rs62605897				
rs73616107				
rs34921821				
rs3923196	X	CRLF2	HGNC Symbol	cytokine receptor-like factor 2 [Source:HGNC Symbol;Acc:14281]
rs3923197	X	CRLF2	HGNC Symbol	cytokine receptor-like factor 2 [Source:HGNC Symbol;Acc:14281]
rs28719175	X	CRLF2	HGNC Symbol	cytokine receptor-like factor 2 [Source:HGNC Symbol;Acc:14281]
rs73616116				
rs34944168				
rs34455248				
rs28757823				
rs28649190				
rs28460331				
rs73175748				
rs28630695				
rs73175756				
rs28574627				
rs73618020	X	CSF2RA	HGNC Symbol	colony stimulating factor 2 receptor, alpha, low-affinity (granulocyte-macrophage) [Source:HGNC Symbol;Acc:2435]
rs28404117	X	CSF2RA	HGNC Symbol	colony stimulating factor 2 receptor, alpha, low-affinity (granulocyte-macrophage) [Source:HGNC Symbol;Acc:2435]
rs28838006	X	CSF2RA	HGNC Symbol	colony stimulating factor 2 receptor, alpha, low-affinity (granulocyte-macrophage) [Source:HGNC Symbol;Acc:2435]
rs28469584	X	CSF2RA	HGNC Symbol	colony stimulating factor 2 receptor, alpha, low-affinity (granulocyte-macrophage) [Source:HGNC Symbol;Acc:2435]

SNP	Chromosome	gene name	gene source	description
rs28535804	X	CSF2RA	HGNC Symbol	colony stimulating factor 2 receptor, alpha, low-affinity (granulocyte-macrophage) [Source:HGNC Symbol;Acc:2435]
rs28482952	X	CSF2RA	HGNC Symbol	colony stimulating factor 2 receptor, alpha, low-affinity (granulocyte-macrophage) [Source:HGNC Symbol;Acc:2435]
rs112263774	X	CSF2RA	HGNC Symbol	colony stimulating factor 2 receptor, alpha, low-affinity (granulocyte-macrophage) [Source:HGNC Symbol;Acc:2435]
rs28584401	X	CSF2RA	HGNC Symbol	colony stimulating factor 2 receptor, alpha, low-affinity (granulocyte-macrophage) [Source:HGNC Symbol;Acc:2435]
rs67006588	X	CSF2RA	HGNC Symbol	colony stimulating factor 2 receptor, alpha, low-affinity (granulocyte-macrophage) [Source:HGNC Symbol;Acc:2435]
rs28578497	X	CSF2RA	HGNC Symbol	colony stimulating factor 2 receptor, alpha, low-affinity (granulocyte-macrophage) [Source:HGNC Symbol;Acc:2435]
rs28716068	X	CSF2RA	HGNC Symbol	colony stimulating factor 2 receptor, alpha, low-affinity (granulocyte-macrophage) [Source:HGNC Symbol;Acc:2435]
rs28762968	X	CSF2RA	HGNC Symbol	colony stimulating factor 2 receptor, alpha, low-affinity (granulocyte-macrophage) [Source:HGNC Symbol;Acc:2435]
rs28377023	X	CSF2RA	HGNC Symbol	colony stimulating factor 2 receptor, alpha, low-affinity (granulocyte-macrophage) [Source:HGNC Symbol;Acc:2435]
rs148100327	X	CSF2RA	HGNC Symbol	colony stimulating factor 2 receptor, alpha, low-affinity (granulocyte-macrophage) [Source:HGNC Symbol;Acc:2435]
rs28460440	X	CSF2RA	HGNC Symbol	colony stimulating factor 2 receptor, alpha, low-affinity (granulocyte-macrophage) [Source:HGNC Symbol;Acc:2435]
rs28580169	X	CSF2RA	HGNC Symbol	colony stimulating factor 2 receptor, alpha, low-affinity (granulocyte-macrophage) [Source:HGNC Symbol;Acc:2435]
rs34875894	X	CSF2RA	HGNC Symbol	colony stimulating factor 2 receptor, alpha, low-affinity (granulocyte-macrophage) [Source:HGNC Symbol;Acc:2435]
rs28721949	X	CSF2RA	HGNC Symbol	colony stimulating factor 2 receptor, alpha, low-affinity (granulocyte-macrophage) [Source:HGNC Symbol;Acc:2435]
rs28736568	X	CSF2RA	HGNC Symbol	colony stimulating factor 2 receptor, alpha, low-affinity (granulocyte-macrophage) [Source:HGNC Symbol;Acc:2435]
rs28722602	X	CSF2RA	HGNC Symbol	colony stimulating factor 2 receptor, alpha, low-affinity (granulocyte-macrophage) [Source:HGNC Symbol;Acc:2435]
rs28489582	X	CSF2RA	HGNC Symbol	colony stimulating factor 2 receptor, alpha, low-affinity (granulocyte-macrophage) [Source:HGNC Symbol;Acc:2435]
rs28469184				
rs28498070				
rs35099787				
rs112148277				
rs73177393				

SNP	Chromosome	gene name	gene source	description
rs35012795				
rs67839578				
rs73177402				
rs73178903				
rs73624872				
rs6422987	X	IL3RA	HGNC Symbol	interleukin 3 receptor, alpha (low affinity) [Source:HGNC Symbol;Acc:6012]
rs7471052	X	IL3RA	HGNC Symbol	interleukin 3 receptor, alpha (low affinity) [Source:HGNC Symbol;Acc:6012]
rs17884091	X	IL3RA	HGNC Symbol	interleukin 3 receptor, alpha (low affinity) [Source:HGNC Symbol;Acc:6012]
rs7056192	X	IL3RA	HGNC Symbol	interleukin 3 receptor, alpha (low affinity) [Source:HGNC Symbol;Acc:6012]
rs17879881	X	IL3RA	HGNC Symbol	interleukin 3 receptor, alpha (low affinity) [Source:HGNC Symbol;Acc:6012]
rs6647005	X	IL3RA	HGNC Symbol	interleukin 3 receptor, alpha (low affinity) [Source:HGNC Symbol;Acc:6012]
rs61303955	X	IL3RA	HGNC Symbol	interleukin 3 receptor, alpha (low affinity) [Source:HGNC Symbol;Acc:6012]
rs28619190	X	IL3RA	HGNC Symbol	interleukin 3 receptor, alpha (low affinity) [Source:HGNC Symbol;Acc:6012]
rs7391575	X	IL3RA	HGNC Symbol	interleukin 3 receptor, alpha (low affinity) [Source:HGNC Symbol;Acc:6012]
rs17884006	X	IL3RA	HGNC Symbol	interleukin 3 receptor, alpha (low affinity) [Source:HGNC Symbol;Acc:6012]
rs17881232	X	IL3RA	HGNC Symbol	interleukin 3 receptor, alpha (low affinity) [Source:HGNC Symbol;Acc:6012]
rs17879004	X	IL3RA	HGNC Symbol	interleukin 3 receptor, alpha (low affinity) [Source:HGNC Symbol;Acc:6012]
rs73178951	X	IL3RA	HGNC Symbol	interleukin 3 receptor, alpha (low affinity) [Source:HGNC Symbol;Acc:6012]
rs7391312	X	IL3RA	HGNC Symbol	interleukin 3 receptor, alpha (low affinity) [Source:HGNC Symbol;Acc:6012]
rs34573150	X	IL3RA	HGNC Symbol	interleukin 3 receptor, alpha (low affinity) [Source:HGNC Symbol;Acc:6012]
rs17883082	X	IL3RA	HGNC Symbol	interleukin 3 receptor, alpha (low affinity) [Source:HGNC Symbol;Acc:6012]
rs6645269	X	IL3RA	HGNC Symbol	interleukin 3 receptor, alpha (low affinity) [Source:HGNC Symbol;Acc:6012]
rs6645279	X	IL3RA	HGNC Symbol	interleukin 3 receptor, alpha (low affinity) [Source:HGNC Symbol;Acc:6012]
rs17883366	X	IL3RA	HGNC Symbol	interleukin 3 receptor, alpha (low affinity) [Source:HGNC Symbol;Acc:6012]
rs6644896				
rs28540518				
rs4933151	X	SLC25A6	HGNC Symbol	solute carrier family 25 (mitochondrial carrier; adenine nucleotide translocator), member 6 [Source:HGNC Symbol;Acc:10992]
rs58680380	X	SLC25A6	HGNC Symbol	solute carrier family 25 (mitochondrial carrier; adenine nucleotide translocator), member 6 [Source:HGNC Symbol;Acc:10992]
rs6644952	X	SLC25A6	HGNC Symbol	solute carrier family 25 (mitochondrial carrier; adenine nucleotide translocator), member 6 [Source:HGNC Symbol;Acc:10992]
rs200654415	X	SLC25A6	HGNC Symbol	solute carrier family 25 (mitochondrial carrier; adenine nucleotide translocator), member 6 [Source:HGNC Symbol;Acc:10992]

SNP	Chromosome	gene name	gene source	description
rs7205	X	SLC25A6	HGNC Symbol	solute carrier family 25 (mitochondrial carrier; adenine nucleotide translocator), member 6 [Source:HGNC Symbol;Acc:10992]
rs14005	X	SLC25A6	HGNC Symbol	solute carrier family 25 (mitochondrial carrier; adenine nucleotide translocator), member 6 [Source:HGNC Symbol;Acc:10992]
rs113486847	X	LINC00106	HGNC Symbol	long intergenic non-protein coding RNA 106 [Source:HGNC Symbol;Acc:31843]
rs4933054	X	LINC00106	HGNC Symbol	long intergenic non-protein coding RNA 106 [Source:HGNC Symbol;Acc:31843]
rs112670469	X	LINC00106	HGNC Symbol	long intergenic non-protein coding RNA 106 [Source:HGNC Symbol;Acc:31843]
rs113370738				
rs28453965	X	ASMTL;ASMTL-AS1	HGNC Symbol	acetylserotonin O-methyltransferase-like [Source:HGNC Symbol;Acc:751];ASMTL antisense RNA 1 [Source:HGNC Symbol;Acc:25811]
rs1127297	X	ASMTL;ASMTL-AS1	HGNC Symbol	acetylserotonin O-methyltransferase-like [Source:HGNC Symbol;Acc:751];ASMTL antisense RNA 1 [Source:HGNC Symbol;Acc:25811]
rs79968027	X	ASMTL;ASMTL-AS1	HGNC Symbol	acetylserotonin O-methyltransferase-like [Source:HGNC Symbol;Acc:751];ASMTL antisense RNA 1 [Source:HGNC Symbol;Acc:25811]
rs5949005	X	ASMTL;ASMTL-AS1	HGNC Symbol	acetylserotonin O-methyltransferase-like [Source:HGNC Symbol;Acc:751];ASMTL antisense RNA 1 [Source:HGNC Symbol;Acc:25811]
rs5025696	X	ASMTL	HGNC Symbol	acetylserotonin O-methyltransferase-like [Source:HGNC Symbol;Acc:751]
rs6645289	X	ASMTL	HGNC Symbol	acetylserotonin O-methyltransferase-like [Source:HGNC Symbol;Acc:751]
rs6644873	X	ASMTL	HGNC Symbol	acetylserotonin O-methyltransferase-like [Source:HGNC Symbol;Acc:751]
rs11553052	X	ASMTL	HGNC Symbol	acetylserotonin O-methyltransferase-like [Source:HGNC Symbol;Acc:751]
rs112133773	X	ASMTL	HGNC Symbol	acetylserotonin O-methyltransferase-like [Source:HGNC Symbol;Acc:751]
rs35466875	X	ASMTL	HGNC Symbol	acetylserotonin O-methyltransferase-like [Source:HGNC Symbol;Acc:751]
rs28727547	X	ASMTL	HGNC Symbol	acetylserotonin O-methyltransferase-like [Source:HGNC Symbol;Acc:751]
rs5949069	X	ASMTL	HGNC Symbol	acetylserotonin O-methyltransferase-like [Source:HGNC Symbol;Acc:751]
rs28528112	X	ASMTL	HGNC Symbol	acetylserotonin O-methyltransferase-like [Source:HGNC Symbol;Acc:751]
rs5948863	X	ASMTL	HGNC Symbol	acetylserotonin O-methyltransferase-like [Source:HGNC Symbol;Acc:751]
rs5989920	X	ASMTL	HGNC Symbol	acetylserotonin O-methyltransferase-like [Source:HGNC Symbol;Acc:751]
rs5989937	X	ASMTL	HGNC Symbol	acetylserotonin O-methyltransferase-like [Source:HGNC Symbol;Acc:751]
rs144051705	X	ASMTL	HGNC Symbol	acetylserotonin O-methyltransferase-like [Source:HGNC Symbol;Acc:751]
rs45465401	X	ASMTL	HGNC Symbol	acetylserotonin O-methyltransferase-like [Source:HGNC Symbol;Acc:751]
rs6644940	X	ASMTL	HGNC Symbol	acetylserotonin O-methyltransferase-like [Source:HGNC Symbol;Acc:751]
rs28880629	X	ASMTL	HGNC Symbol	acetylserotonin O-methyltransferase-like [Source:HGNC Symbol;Acc:751]
rs28537349	X	ASMTL	HGNC Symbol	acetylserotonin O-methyltransferase-like [Source:HGNC Symbol;Acc:751]
rs28674639	X	ASMTL	HGNC Symbol	acetylserotonin O-methyltransferase-like [Source:HGNC Symbol;Acc:751]
rs56200635	X	ASMTL	HGNC Symbol	acetylserotonin O-methyltransferase-like [Source:HGNC Symbol;Acc:751]

SNP	Chromosome	gene name	gene source	description
rs6644946	X	ASMTL	HGNC Symbol	acetylserotonin O-methyltransferase-like [Source:HGNC Symbol;Acc:751]
rs6655422	X	ASMTL	HGNC Symbol	acetylserotonin O-methyltransferase-like [Source:HGNC Symbol;Acc:751]
rs73182962				
rs57525642				
rs28609053	X	P2RY8	HGNC Symbol	purinergic receptor P2Y, G-protein coupled, 8 [Source:HGNC Symbol;Acc:15524]
rs4933152	X	P2RY8	HGNC Symbol	purinergic receptor P2Y, G-protein coupled, 8 [Source:HGNC Symbol;Acc:15524]
rs73182974	X	P2RY8	HGNC Symbol	purinergic receptor P2Y, G-protein coupled, 8 [Source:HGNC Symbol;Acc:15524]
rs4548373	X	P2RY8	HGNC Symbol	purinergic receptor P2Y, G-protein coupled, 8 [Source:HGNC Symbol;Acc:15524]
rs35544304	X	P2RY8	HGNC Symbol	purinergic receptor P2Y, G-protein coupled, 8 [Source:HGNC Symbol;Acc:15524]
rs28578016	X	P2RY8	HGNC Symbol	purinergic receptor P2Y, G-protein coupled, 8 [Source:HGNC Symbol;Acc:15524]
rs28450615	X	P2RY8	HGNC Symbol	purinergic receptor P2Y, G-protein coupled, 8 [Source:HGNC Symbol;Acc:15524]
rs28684380	X	P2RY8	HGNC Symbol	purinergic receptor P2Y, G-protein coupled, 8 [Source:HGNC Symbol;Acc:15524]
rs28391357	X	P2RY8	HGNC Symbol	purinergic receptor P2Y, G-protein coupled, 8 [Source:HGNC Symbol;Acc:15524]
rs5949204	X	P2RY8	HGNC Symbol	purinergic receptor P2Y, G-protein coupled, 8 [Source:HGNC Symbol;Acc:15524]
rs28685231	X	P2RY8	HGNC Symbol	purinergic receptor P2Y, G-protein coupled, 8 [Source:HGNC Symbol;Acc:15524]
rs34487068	X	P2RY8	HGNC Symbol	purinergic receptor P2Y, G-protein coupled, 8 [Source:HGNC Symbol;Acc:15524]
rs28485295	X	P2RY8	HGNC Symbol	purinergic receptor P2Y, G-protein coupled, 8 [Source:HGNC Symbol;Acc:15524]
rs56392639	X	P2RY8	HGNC Symbol	purinergic receptor P2Y, G-protein coupled, 8 [Source:HGNC Symbol;Acc:15524]
rs6644711	X	P2RY8	HGNC Symbol	purinergic receptor P2Y, G-protein coupled, 8 [Source:HGNC Symbol;Acc:15524]
rs6645234	X	P2RY8	HGNC Symbol	purinergic receptor P2Y, G-protein coupled, 8 [Source:HGNC Symbol;Acc:15524]
rs5989754	X	P2RY8	HGNC Symbol	purinergic receptor P2Y, G-protein coupled, 8 [Source:HGNC Symbol;Acc:15524]
rs6644603	X	P2RY8	HGNC Symbol	purinergic receptor P2Y, G-protein coupled, 8 [Source:HGNC Symbol;Acc:15524]
rs5989761	X	P2RY8	HGNC Symbol	purinergic receptor P2Y, G-protein coupled, 8 [Source:HGNC Symbol;Acc:15524]
rs5989764	X	P2RY8	HGNC Symbol	purinergic receptor P2Y, G-protein coupled, 8 [Source:HGNC Symbol;Acc:15524]
rs66513751	X	P2RY8	HGNC Symbol	purinergic receptor P2Y, G-protein coupled, 8 [Source:HGNC Symbol;Acc:15524]
rs73184979	X	P2RY8	HGNC Symbol	purinergic receptor P2Y, G-protein coupled, 8 [Source:HGNC Symbol;Acc:15524]
rs28649312	X	P2RY8	HGNC Symbol	purinergic receptor P2Y, G-protein coupled, 8 [Source:HGNC Symbol;Acc:15524]
rs6645262	X	P2RY8	HGNC Symbol	purinergic receptor P2Y, G-protein coupled, 8 [Source:HGNC Symbol;Acc:15524]
rs5948925	X	P2RY8	HGNC Symbol	purinergic receptor P2Y, G-protein coupled, 8 [Source:HGNC Symbol;Acc:15524]
rs28505216	X	P2RY8	HGNC Symbol	purinergic receptor P2Y, G-protein coupled, 8 [Source:HGNC Symbol;Acc:15524]
rs73186906	X	P2RY8	HGNC Symbol	purinergic receptor P2Y, G-protein coupled, 8 [Source:HGNC Symbol;Acc:15524]
rs4076017	X	P2RY8	HGNC Symbol	purinergic receptor P2Y, G-protein coupled, 8 [Source:HGNC Symbol;Acc:15524]
rs62603050	X	P2RY8	HGNC Symbol	purinergic receptor P2Y, G-protein coupled, 8 [Source:HGNC Symbol;Acc:15524]

SNP	Chromosome	gene name	gene source	description
rs5948931	X	P2RY8	HGNC Symbol	purinergic receptor P2Y, G-protein coupled, 8 [Source:HGNC Symbol;Acc:15524]
rs4933085	X	P2RY8	HGNC Symbol	purinergic receptor P2Y, G-protein coupled, 8 [Source:HGNC Symbol;Acc:15524]
rs73186931	X	P2RY8	HGNC Symbol	purinergic receptor P2Y, G-protein coupled, 8 [Source:HGNC Symbol;Acc:15524]
rs73186934	X	P2RY8	HGNC Symbol	purinergic receptor P2Y, G-protein coupled, 8 [Source:HGNC Symbol;Acc:15524]
rs6644727	X	P2RY8	HGNC Symbol	purinergic receptor P2Y, G-protein coupled, 8 [Source:HGNC Symbol;Acc:15524]
rs73186942	X	P2RY8	HGNC Symbol	purinergic receptor P2Y, G-protein coupled, 8 [Source:HGNC Symbol;Acc:15524]
rs5948934	X	P2RY8	HGNC Symbol	purinergic receptor P2Y, G-protein coupled, 8 [Source:HGNC Symbol;Acc:15524]
rs28619240	X	P2RY8	HGNC Symbol	purinergic receptor P2Y, G-protein coupled, 8 [Source:HGNC Symbol;Acc:15524]
rs73186974	X	P2RY8	HGNC Symbol	purinergic receptor P2Y, G-protein coupled, 8 [Source:HGNC Symbol;Acc:15524]
rs28636482				
rs7391970				
rs5948961				
rs111997635				
rs58232928				
rs140336138				
rs5742040				
rs6588783				
rs28470496				
rs55685301				
rs5948964				
rs5948950				
rs62593889				
rs5948969				
rs112524240				
rs62593893				
rs6644615				
rs5948978				
rs4498736				
rs66967817				
rs111666237				
rs6644619				
rs7471229				
rs56827686				

SNP	Chromosome	gene name	gene source	description
rs6588799				
rs4317751				
rs28490591				
rs6644621	X	AKAP17A	HGNC Symbol	A kinase (PRKA) anchor protein 17A [Source:HGNC Symbol;Acc:18783]
rs56336628	X	AKAP17A	HGNC Symbol	A kinase (PRKA) anchor protein 17A [Source:HGNC Symbol;Acc:18783]
rs6644765	X	AKAP17A	HGNC Symbol	A kinase (PRKA) anchor protein 17A [Source:HGNC Symbol;Acc:18783]
rs28513563	X	AKAP17A	HGNC Symbol	A kinase (PRKA) anchor protein 17A [Source:HGNC Symbol;Acc:18783]
rs35214874	X	AKAP17A	HGNC Symbol	A kinase (PRKA) anchor protein 17A [Source:HGNC Symbol;Acc:18783]
rs28729076				
rs28610575				
rs5989833				
rs57666302				
rs73190852				
rs73190858				
rs62593283				
rs28696100				
rs28550202				
rs62593289				
rs6644635	X	ASMT	HGNC Symbol	acetylserotonin O-methyltransferase [Source:HGNC Symbol;Acc:750]
rs5989853	X	ASMT	HGNC Symbol	acetylserotonin O-methyltransferase [Source:HGNC Symbol;Acc:750]
rs73174016	X	ASMT	HGNC Symbol	acetylserotonin O-methyltransferase [Source:HGNC Symbol;Acc:750]
rs7061961	X	ASMT	HGNC Symbol	acetylserotonin O-methyltransferase [Source:HGNC Symbol;Acc:750]
rs57468917	X	ASMT	HGNC Symbol	acetylserotonin O-methyltransferase [Source:HGNC Symbol;Acc:750]
rs6588802	X	ASMT	HGNC Symbol	acetylserotonin O-methyltransferase [Source:HGNC Symbol;Acc:750]
rs7883695	X	ASMT	HGNC Symbol	acetylserotonin O-methyltransferase [Source:HGNC Symbol;Acc:750]
rs62593301	X	ASMT	HGNC Symbol	acetylserotonin O-methyltransferase [Source:HGNC Symbol;Acc:750]
rs7063283	X	ASMT	HGNC Symbol	acetylserotonin O-methyltransferase [Source:HGNC Symbol;Acc:750]
rs34397308	X	ASMT	HGNC Symbol	acetylserotonin O-methyltransferase [Source:HGNC Symbol;Acc:750]
rs6588807	X	ASMT	HGNC Symbol	acetylserotonin O-methyltransferase [Source:HGNC Symbol;Acc:750]
rs28675287	X	ASMT	HGNC Symbol	acetylserotonin O-methyltransferase [Source:HGNC Symbol;Acc:750]
rs56045909	X	ASMT	HGNC Symbol	acetylserotonin O-methyltransferase [Source:HGNC Symbol;Acc:750]
rs28827247	X	ASMT	HGNC Symbol	acetylserotonin O-methyltransferase [Source:HGNC Symbol;Acc:750]
rs7881098	X	ASMT	HGNC Symbol	acetylserotonin O-methyltransferase [Source:HGNC Symbol;Acc:750]

SNP	Chromosome	gene name	gene source	description
rs4639690	X	ASMT	HGNC Symbol	acetylserotonin O-methyltransferase [Source:HGNC Symbol;Acc:750]
rs4933063	X	ASMT	HGNC Symbol	acetylserotonin O-methyltransferase [Source:HGNC Symbol;Acc:750]
rs4529639				
rs7889728				
rs5989843				
rs7391672				
rs6588814				
rs57244318				
rs5949036				
rs5989690				
rs6588825				
rs73174093				
rs6644652				
rs5989869				
rs5989872				
rs28897149				
rs5948853				
rs7882667				
rs73175307				
rs28455505				
rs7891786				
rs73175326				
rs5948856				
rs6644661				
rs73175330				
rs5989880				
rs6644825				
rs73175343				
rs5949070				
rs5989883				
rs5948860				
rs181665650				
rs4428822				

SNP	Chromosome	gene name	gene source	description
rs5949087				
rs55884326				
rs28446038				
rs1470876				
rs1470875				
rs2167932				
rs2884952				
rs6644848				
rs34864168				
rs4933111				
rs5989916				
rs6644678	X	RP13-297E16.4	Clone-based (Vega) gene	
rs34611407	X	RP13-297E16.4	Clone-based (Vega) gene	
rs34924107	X	RP13-297E16.4	Clone-based (Vega) gene	
rs4933116	X	RP13-297E16.4	Clone-based (Vega) gene	
rs66593223	X	RP13-297E16.4	Clone-based (Vega) gene	
rs7880453	X	RP13-297E16.4	Clone-based (Vega) gene	
rs78371955	X	RP13-297E16.4	Clone-based (Vega) gene	
rs5989926	X	RP13-297E16.4	Clone-based (Vega) gene	
rs7892423	X	RP13-297E16.4	Clone-based (Vega) gene	
rs34849634	X	RP13-297E16.4	Clone-based (Vega) gene	
rs141521666	X	RP13-297E16.4	Clone-based (Vega) gene	
rs62596464	X	RP13-297E16.4	Clone-based (Vega) gene	
rs28646004				
rs1563120				

SNP	Chromosome	gene name	gene source	description
rs5948876				
rs71210249				
rs28402118				
rs4933068				
rs73177562				
rs4933131				
rs28626767				
rs58532553	X	RP13-297E16.5	Clone-based (Vega) gene	
rs28536665				
rs6644681				
rs5949128				
rs6644683				
rs5948884				
rs5989945				
rs4933076				
rs116968265				
rs28633868				
rs28375293				
rs28451978				
rs73177597				
rs3851031				
rs5989957				
rs5949147				
rs2360263				
rs67003311				
rs28461211				
rs34727095				
rs36176126				
rs73628381				
rs73179622				
rs7051519				
rs73629861				

SNP	Chromosome	gene name	gene source	description
rs6644687				
rs6588867				
rs71208051				
rs7892807				
rs59809471				
rs73629883				
rs2360259				
rs7878782				
rs5949181				
rs5989985				
rs5989732				
rs28584802				
rs5949189				
rs5949190				
rs73179667				
rs73179672				
rs5948902				
rs28623232				
rs116865723				
rs5989995				
rs7054570				
rs5949196				
rs6588877				
rs6588882				
rs6588883				
rs6588884				
rs6644915				
rs6644919				
rs73181537				
rs6588891				
rs6644695				
rs73181549				
rs73181553				

SNP	Chromosome	gene name	gene source	description
rs73623873				
rs28758502				
rs28405703				
rs113378368				
rs7883640				
rs62593245				
rs62593247				
rs73623879				
rs6588902				
rs67997355				
rs28425743				
rs76982438				
rs28660670				
rs7876414				
rs28494187				
rs28463581				
rs28397008				
rs28831750				
rs28405659				
rs56096431				
rs28589468				
rs28558833				
rs28584259				
rs73623901				
rs113523951				
rs7064310				
rs144720797				
rs6588906				
rs6644937				
rs28600363				
rs28491781				
rs6421534				
rs73183434				

SNP	Chromosome	gene name	gene source	description
rs6588911				
rs6421536				
rs73183441				
rs68044411				
rs66462858				
rs35600819				
rs34637935	X	DHRSX	HGNC Symbol	dehydrogenase/reductase (SDR family) X-linked [Source:HGNC Symbol;Acc:18399]
rs12010	X	DHRSX	HGNC Symbol	dehydrogenase/reductase (SDR family) X-linked [Source:HGNC Symbol;Acc:18399]
rs3210910	X	DHRSX	HGNC Symbol	dehydrogenase/reductase (SDR family) X-linked [Source:HGNC Symbol;Acc:18399]
rs34553691	X	DHRSX	HGNC Symbol	dehydrogenase/reductase (SDR family) X-linked [Source:HGNC Symbol;Acc:18399]
rs186641692	X	DHRSX	HGNC Symbol	dehydrogenase/reductase (SDR family) X-linked [Source:HGNC Symbol;Acc:18399]
rs1127915	X	DHRSX	HGNC Symbol	dehydrogenase/reductase (SDR family) X-linked [Source:HGNC Symbol;Acc:18399]
rs73183497	X	DHRSX	HGNC Symbol	dehydrogenase/reductase (SDR family) X-linked [Source:HGNC Symbol;Acc:18399]
rs35805291	X	DHRSX	HGNC Symbol	dehydrogenase/reductase (SDR family) X-linked [Source:HGNC Symbol;Acc:18399]
rs35731332	X	DHRSX	HGNC Symbol	dehydrogenase/reductase (SDR family) X-linked [Source:HGNC Symbol;Acc:18399]
rs62595504	X	DHRSX	HGNC Symbol	dehydrogenase/reductase (SDR family) X-linked [Source:HGNC Symbol;Acc:18399]
rs67146428	X	DHRSX	HGNC Symbol	dehydrogenase/reductase (SDR family) X-linked [Source:HGNC Symbol;Acc:18399]
rs73185750	X	DHRSX	HGNC Symbol	dehydrogenase/reductase (SDR family) X-linked [Source:HGNC Symbol;Acc:18399]
rs139542133	X	DHRSX	HGNC Symbol	dehydrogenase/reductase (SDR family) X-linked [Source:HGNC Symbol;Acc:18399]
rs73185753	X	DHRSX	HGNC Symbol	dehydrogenase/reductase (SDR family) X-linked [Source:HGNC Symbol;Acc:18399]
rs112514645	X	DHRSX	HGNC Symbol	dehydrogenase/reductase (SDR family) X-linked [Source:HGNC Symbol;Acc:18399]
rs62595533	X	DHRSX	HGNC Symbol	dehydrogenase/reductase (SDR family) X-linked [Source:HGNC Symbol;Acc:18399]
rs189837987	X	DHRSX	HGNC Symbol	dehydrogenase/reductase (SDR family) X-linked [Source:HGNC Symbol;Acc:18399]
rs73628284	X	DHRSX	HGNC Symbol	dehydrogenase/reductase (SDR family) X-linked [Source:HGNC Symbol;Acc:18399]
rs184373694	X	DHRSX	HGNC Symbol	dehydrogenase/reductase (SDR family) X-linked [Source:HGNC Symbol;Acc:18399]
rs143362134	X	DHRSX	HGNC Symbol	dehydrogenase/reductase (SDR family) X-linked [Source:HGNC Symbol;Acc:18399]
rs73628289	X	DHRSX	HGNC Symbol	dehydrogenase/reductase (SDR family) X-linked [Source:HGNC Symbol;Acc:18399]
rs34016541	X	DHRSX	HGNC Symbol	dehydrogenase/reductase (SDR family) X-linked [Source:HGNC Symbol;Acc:18399]
rs62593066	X	DHRSX	HGNC Symbol	dehydrogenase/reductase (SDR family) X-linked [Source:HGNC Symbol;Acc:18399]
rs35660934	X	DHRSX	HGNC Symbol	dehydrogenase/reductase (SDR family) X-linked [Source:HGNC Symbol;Acc:18399]
rs73628298	X	DHRSX	HGNC Symbol	dehydrogenase/reductase (SDR family) X-linked [Source:HGNC Symbol;Acc:18399]
rs73187640	X	DHRSX	HGNC Symbol	dehydrogenase/reductase (SDR family) X-linked [Source:HGNC Symbol;Acc:18399]
rs62583695	X	DHRSX	HGNC Symbol	dehydrogenase/reductase (SDR family) X-linked [Source:HGNC Symbol;Acc:18399]

SNP	Chromosome	gene name	gene source	description
rs62583697	X	DHRSX	HGNC Symbol	dehydrogenase/reductase (SDR family) X-linked [Source:HGNC Symbol;Acc:18399]
rs4083047	X	DHRSX	HGNC Symbol	dehydrogenase/reductase (SDR family) X-linked [Source:HGNC Symbol;Acc:18399]
rs62583722	X	DHRSX	HGNC Symbol	dehydrogenase/reductase (SDR family) X-linked [Source:HGNC Symbol;Acc:18399]
rs67152939	X	DHRSX	HGNC Symbol	dehydrogenase/reductase (SDR family) X-linked [Source:HGNC Symbol;Acc:18399]
rs34220838	X	DHRSX	HGNC Symbol	dehydrogenase/reductase (SDR family) X-linked [Source:HGNC Symbol;Acc:18399]
rs28629876	X	DHRSX	HGNC Symbol	dehydrogenase/reductase (SDR family) X-linked [Source:HGNC Symbol;Acc:18399]
rs9785588	X	DHRSX	HGNC Symbol	dehydrogenase/reductase (SDR family) X-linked [Source:HGNC Symbol;Acc:18399]
rs7063971	X	DHRSX	HGNC Symbol	dehydrogenase/reductase (SDR family) X-linked [Source:HGNC Symbol;Acc:18399]
rs73189683	X	DHRSX	HGNC Symbol	dehydrogenase/reductase (SDR family) X-linked [Source:HGNC Symbol;Acc:18399]
rs73189685	X	DHRSX	HGNC Symbol	dehydrogenase/reductase (SDR family) X-linked [Source:HGNC Symbol;Acc:18399]
rs6641687	X	DHRSX	HGNC Symbol	dehydrogenase/reductase (SDR family) X-linked [Source:HGNC Symbol;Acc:18399]
rs28670855	X	DHRSX	HGNC Symbol	dehydrogenase/reductase (SDR family) X-linked [Source:HGNC Symbol;Acc:18399]
rs56048312	X	DHRSX	HGNC Symbol	dehydrogenase/reductase (SDR family) X-linked [Source:HGNC Symbol;Acc:18399]
rs12855151	X	DHRSX	HGNC Symbol	dehydrogenase/reductase (SDR family) X-linked [Source:HGNC Symbol;Acc:18399]
rs6567519	X	DHRSX	HGNC Symbol	dehydrogenase/reductase (SDR family) X-linked [Source:HGNC Symbol;Acc:18399]
rs149212165	X	DHRSX	HGNC Symbol	dehydrogenase/reductase (SDR family) X-linked [Source:HGNC Symbol;Acc:18399]
rs57070520	X	DHRSX	HGNC Symbol	dehydrogenase/reductase (SDR family) X-linked [Source:HGNC Symbol;Acc:18399]
rs73191309	X	DHRSX	HGNC Symbol	dehydrogenase/reductase (SDR family) X-linked [Source:HGNC Symbol;Acc:18399]
rs7880206	X	DHRSX	HGNC Symbol	dehydrogenase/reductase (SDR family) X-linked [Source:HGNC Symbol;Acc:18399]
rs7063483	X	DHRSX	HGNC Symbol	dehydrogenase/reductase (SDR family) X-linked [Source:HGNC Symbol;Acc:18399]
rs28418610	X	DHRSX	HGNC Symbol	dehydrogenase/reductase (SDR family) X-linked [Source:HGNC Symbol;Acc:18399]
rs2317106	X	DHRSX	HGNC Symbol	dehydrogenase/reductase (SDR family) X-linked [Source:HGNC Symbol;Acc:18399]
rs9785591	X	DHRSX	HGNC Symbol	dehydrogenase/reductase (SDR family) X-linked [Source:HGNC Symbol;Acc:18399]
rs35472853	X	DHRSX	HGNC Symbol	dehydrogenase/reductase (SDR family) X-linked [Source:HGNC Symbol;Acc:18399]
rs7062927	X	DHRSX	HGNC Symbol	dehydrogenase/reductase (SDR family) X-linked [Source:HGNC Symbol;Acc:18399]
rs5939175	X	DHRSX	HGNC Symbol	dehydrogenase/reductase (SDR family) X-linked [Source:HGNC Symbol;Acc:18399]
rs28665237	X	DHRSX	HGNC Symbol	dehydrogenase/reductase (SDR family) X-linked [Source:HGNC Symbol;Acc:18399]
rs73191364	X	DHRSX	HGNC Symbol	dehydrogenase/reductase (SDR family) X-linked [Source:HGNC Symbol;Acc:18399]
rs6642077	X	DHRSX	HGNC Symbol	dehydrogenase/reductase (SDR family) X-linked [Source:HGNC Symbol;Acc:18399]
rs7879755	X	DHRSX	HGNC Symbol	dehydrogenase/reductase (SDR family) X-linked [Source:HGNC Symbol;Acc:18399]
rs73173708	X	DHRSX	HGNC Symbol	dehydrogenase/reductase (SDR family) X-linked [Source:HGNC Symbol;Acc:18399]
rs5939091	X	DHRSX	HGNC Symbol	dehydrogenase/reductase (SDR family) X-linked [Source:HGNC Symbol;Acc:18399]
rs57012236	X	DHRSX	HGNC Symbol	dehydrogenase/reductase (SDR family) X-linked [Source:HGNC Symbol;Acc:18399]

SNP	Chromosome	gene name	gene source	description
rs5939242	X	DHRSX	HGNC Symbol	dehydrogenase/reductase (SDR family) X-linked [Source:HGNC Symbol;Acc:18399]
rs73173783	X	DHRSX	HGNC Symbol	dehydrogenase/reductase (SDR family) X-linked [Source:HGNC Symbol;Acc:18399]
rs189203665	X	DHRSX	HGNC Symbol	dehydrogenase/reductase (SDR family) X-linked [Source:HGNC Symbol;Acc:18399]
rs112545028	X	DHRSX	HGNC Symbol	dehydrogenase/reductase (SDR family) X-linked [Source:HGNC Symbol;Acc:18399]
rs150589405	X	DHRSX	HGNC Symbol	dehydrogenase/reductase (SDR family) X-linked [Source:HGNC Symbol;Acc:18399]
rs114169872	X	DHRSX	HGNC Symbol	dehydrogenase/reductase (SDR family) X-linked [Source:HGNC Symbol;Acc:18399]
rs7054020	X	DHRSX	HGNC Symbol	dehydrogenase/reductase (SDR family) X-linked [Source:HGNC Symbol;Acc:18399]
rs5939127	X	DHRSX	HGNC Symbol	dehydrogenase/reductase (SDR family) X-linked [Source:HGNC Symbol;Acc:18399]
rs61148409	X	DHRSX	HGNC Symbol	dehydrogenase/reductase (SDR family) X-linked [Source:HGNC Symbol;Acc:18399]
rs6642167	X	DHRSX	HGNC Symbol	dehydrogenase/reductase (SDR family) X-linked [Source:HGNC Symbol;Acc:18399]
rs6642184	X	DHRSX	HGNC Symbol	dehydrogenase/reductase (SDR family) X-linked [Source:HGNC Symbol;Acc:18399]
rs4892905	X	DHRSX	HGNC Symbol	dehydrogenase/reductase (SDR family) X-linked [Source:HGNC Symbol;Acc:18399]
rs5982607	X	DHRSX	HGNC Symbol	dehydrogenase/reductase (SDR family) X-linked [Source:HGNC Symbol;Acc:18399]
rs7885495	X	DHRSX	HGNC Symbol	dehydrogenase/reductase (SDR family) X-linked [Source:HGNC Symbol;Acc:18399]
rs28645921	X	DHRSX	HGNC Symbol	dehydrogenase/reductase (SDR family) X-linked [Source:HGNC Symbol;Acc:18399]
rs55870101	X	DHRSX	HGNC Symbol	dehydrogenase/reductase (SDR family) X-linked [Source:HGNC Symbol;Acc:18399]
rs6641784	X	DHRSX	HGNC Symbol	dehydrogenase/reductase (SDR family) X-linked [Source:HGNC Symbol;Acc:18399]
rs17842869	X	DHRSX	HGNC Symbol	dehydrogenase/reductase (SDR family) X-linked [Source:HGNC Symbol;Acc:18399]
rs115599487	X	DHRSX	HGNC Symbol	dehydrogenase/reductase (SDR family) X-linked [Source:HGNC Symbol;Acc:18399]
rs6641809	X	DHRSX	HGNC Symbol	dehydrogenase/reductase (SDR family) X-linked [Source:HGNC Symbol;Acc:18399]
rs28712854	X	DHRSX	HGNC Symbol	dehydrogenase/reductase (SDR family) X-linked [Source:HGNC Symbol;Acc:18399]
rs5983086	X	DHRSX	HGNC Symbol	dehydrogenase/reductase (SDR family) X-linked [Source:HGNC Symbol;Acc:18399]
rs4489475	X	DHRSX	HGNC Symbol	dehydrogenase/reductase (SDR family) X-linked [Source:HGNC Symbol;Acc:18399]
rs28460787	X	DHRSX	HGNC Symbol	dehydrogenase/reductase (SDR family) X-linked [Source:HGNC Symbol;Acc:18399]
rs149645208	X	DHRSX	HGNC Symbol	dehydrogenase/reductase (SDR family) X-linked [Source:HGNC Symbol;Acc:18399]
rs1317657	X	DHRSX	HGNC Symbol	dehydrogenase/reductase (SDR family) X-linked [Source:HGNC Symbol;Acc:18399]
rs4892840	X	DHRSX	HGNC Symbol	dehydrogenase/reductase (SDR family) X-linked [Source:HGNC Symbol;Acc:18399]
rs7891627	X	DHRSX;ZBED1	HGNC Symbol	dehydrogenase/reductase (SDR family) X-linked [Source:HGNC Symbol;Acc:18399];zinc finger, BED-type containing 1 [Source:HGNC Symbol;Acc:447]
rs1044307	X	DHRSX;ZBED1	HGNC Symbol	dehydrogenase/reductase (SDR family) X-linked [Source:HGNC Symbol;Acc:18399];zinc finger, BED-type containing 1 [Source:HGNC Symbol;Acc:447]
rs6567595	X	DHRSX;ZBED1	HGNC Symbol	dehydrogenase/reductase (SDR family) X-linked [Source:HGNC Symbol;Acc:18399];zinc finger, BED-type containing 1 [Source:HGNC Symbol;Acc:447]

SNP	Chromosome	gene name	gene source	description
rs1062804	X	DHRSX;ZBED1	HGNC Symbol	dehydrogenase/reductase (SDR family) X-linked [Source:HGNC Symbol;Acc:18399];zinc finger, BED-type containing 1 [Source:HGNC Symbol;Acc:447]
rs17842876	X	DHRSX;ZBED1	HGNC Symbol	dehydrogenase/reductase (SDR family) X-linked [Source:HGNC Symbol;Acc:18399];zinc finger, BED-type containing 1 [Source:HGNC Symbol;Acc:447]
rs148140814	X	DHRSX;ZBED1	HGNC Symbol	dehydrogenase/reductase (SDR family) X-linked [Source:HGNC Symbol;Acc:18399];zinc finger, BED-type containing 1 [Source:HGNC Symbol;Acc:447]
rs3203783	X	DHRSX;ZBED1	HGNC Symbol	dehydrogenase/reductase (SDR family) X-linked [Source:HGNC Symbol;Acc:18399];zinc finger, BED-type containing 1 [Source:HGNC Symbol;Acc:447]
rs4892932	X	DHRSX;ZBED1	HGNC Symbol	dehydrogenase/reductase (SDR family) X-linked [Source:HGNC Symbol;Acc:18399];zinc finger, BED-type containing 1 [Source:HGNC Symbol;Acc:447]
rs5939179	X	DHRSX;ZBED1	HGNC Symbol	dehydrogenase/reductase (SDR family) X-linked [Source:HGNC Symbol;Acc:18399];zinc finger, BED-type containing 1 [Source:HGNC Symbol;Acc:447]
rs140302401	X	DHRSX;ZBED1	HGNC Symbol	dehydrogenase/reductase (SDR family) X-linked [Source:HGNC Symbol;Acc:18399];zinc finger, BED-type containing 1 [Source:HGNC Symbol;Acc:447]
rs5939497	X	DHRSX;ZBED1	HGNC Symbol	dehydrogenase/reductase (SDR family) X-linked [Source:HGNC Symbol;Acc:18399];zinc finger, BED-type containing 1 [Source:HGNC Symbol;Acc:447]
rs28448606	X	DHRSX;ZBED1	HGNC Symbol	dehydrogenase/reductase (SDR family) X-linked [Source:HGNC Symbol;Acc:18399];zinc finger, BED-type containing 1 [Source:HGNC Symbol;Acc:447]
rs2316860				
rs2316858				
rs5939200				
rs73182827				
rs73182829				
rs28496467				
rs5939074				
rs5982723				
rs5939210				
rs56151700				
rs6641927				
rs141259781				
rs73186679				
rs111713641				
rs28392935				
rs5939080				
rs5982733				
rs182135958				

SNP	Chromosome	gene name	gene source	description
rs5982748				
rs5939216				
rs951323				
rs6641952				
rs5982552				
rs17842894				
rs1997482				
rs4892816				
rs17842901				
rs35603448				
rs5939084				
rs4892859				
rs6567618				
rs5939233				
rs5939088				
rs5939089				
rs5982781				
rs7064867				
rs5939236				
rs28615082				
rs6641973	X	CD99P1	HGNC Symbol	CD99 molecule pseudogene 1 [Source:HGNC Symbol;Acc:7083]
rs115811462	X	CD99P1	HGNC Symbol	CD99 molecule pseudogene 1 [Source:HGNC Symbol;Acc:7083]
rs5982786	X	CD99P1;LINC00102	HGNC Symbol	CD99 molecule pseudogene 1 [Source:HGNC Symbol;Acc:7083];long intergenic non-protein coding RNA 102 [Source:HGNC Symbol;Acc:30470]
rs73183609	X	CD99P1;LINC00102	HGNC Symbol	CD99 molecule pseudogene 1 [Source:HGNC Symbol;Acc:7083];long intergenic non-protein coding RNA 102 [Source:HGNC Symbol;Acc:30470]
rs7061856	X	CD99P1;LINC00102	HGNC Symbol	CD99 molecule pseudogene 1 [Source:HGNC Symbol;Acc:7083];long intergenic non-protein coding RNA 102 [Source:HGNC Symbol;Acc:30470]
rs5939243	X	CD99P1	HGNC Symbol	CD99 molecule pseudogene 1 [Source:HGNC Symbol;Acc:7083]
rs2174468	X	CD99P1	HGNC Symbol	CD99 molecule pseudogene 1 [Source:HGNC Symbol;Acc:7083]
rs5939245	X	CD99P1	HGNC Symbol	CD99 molecule pseudogene 1 [Source:HGNC Symbol;Acc:7083]
rs6641976	X	CD99P1	HGNC Symbol	CD99 molecule pseudogene 1 [Source:HGNC Symbol;Acc:7083]
rs5939095	X	CD99P1	HGNC Symbol	CD99 molecule pseudogene 1 [Source:HGNC Symbol;Acc:7083]
rs56281454	X	CD99P1	HGNC Symbol	CD99 molecule pseudogene 1 [Source:HGNC Symbol;Acc:7083]

SNP	Chromosome	gene name	gene source	description
rs71207031	X	CD99P1	HGNC Symbol	CD99 molecule pseudogene 1 [Source:HGNC Symbol;Acc:7083]
rs6567632	X	CD99P1	HGNC Symbol	CD99 molecule pseudogene 1 [Source:HGNC Symbol;Acc:7083]
rs2271066	X	CD99P1	HGNC Symbol	CD99 molecule pseudogene 1 [Source:HGNC Symbol;Acc:7083]
rs145435329	X	CD99P1	HGNC Symbol	CD99 molecule pseudogene 1 [Source:HGNC Symbol;Acc:7083]
rs73189832	X	CD99P1	HGNC Symbol	CD99 molecule pseudogene 1 [Source:HGNC Symbol;Acc:7083]
rs17471918	X	CD99P1	HGNC Symbol	CD99 molecule pseudogene 1 [Source:HGNC Symbol;Acc:7083]
rs28636879	X	CD99P1	HGNC Symbol	CD99 molecule pseudogene 1 [Source:HGNC Symbol;Acc:7083]
rs721725	X	CD99P1	HGNC Symbol	CD99 molecule pseudogene 1 [Source:HGNC Symbol;Acc:7083]
rs36076314	X	CD99P1	HGNC Symbol	CD99 molecule pseudogene 1 [Source:HGNC Symbol;Acc:7083]
rs73189848	X	CD99P1	HGNC Symbol	CD99 molecule pseudogene 1 [Source:HGNC Symbol;Acc:7083]
rs5939276	X	CD99P1	HGNC Symbol	CD99 molecule pseudogene 1 [Source:HGNC Symbol;Acc:7083]
rs6641634	X	CD99P1	HGNC Symbol	CD99 molecule pseudogene 1 [Source:HGNC Symbol;Acc:7083]
rs28504362	X	CD99P1	HGNC Symbol	CD99 molecule pseudogene 1 [Source:HGNC Symbol;Acc:7083]
rs6567636	X	CD99P1	HGNC Symbol	CD99 molecule pseudogene 1 [Source:HGNC Symbol;Acc:7083]
rs28558759	X	CD99P1	HGNC Symbol	CD99 molecule pseudogene 1 [Source:HGNC Symbol;Acc:7083]
rs59170330	X	CD99P1	HGNC Symbol	CD99 molecule pseudogene 1 [Source:HGNC Symbol;Acc:7083]
rs5939282	X	CD99P1	HGNC Symbol	CD99 molecule pseudogene 1 [Source:HGNC Symbol;Acc:7083]
rs5939284	X	CD99P1	HGNC Symbol	CD99 molecule pseudogene 1 [Source:HGNC Symbol;Acc:7083]
rs4892877	X	CD99P1	HGNC Symbol	CD99 molecule pseudogene 1 [Source:HGNC Symbol;Acc:7083]
rs3813164				
rs28416483				
rs5982816				
rs55774824				
rs28694664				
rs111751926				
rs310139				
rs1419929				
rs7879113				
rs1700949				
rs28589836				
rs17808091				
rs310147				
rs17842914				

SNP	Chromosome	gene name	gene source	description
rs28523739				
rs28592721				
rs112337585				
rs28498577				
rs28649830				
rs28432965				
rs1700954				
rs5939299	X	CD99	HGNC Symbol	CD99 molecule [Source:HGNC Symbol;Acc:7082]
rs2236738	X	CD99	HGNC Symbol	CD99 molecule [Source:HGNC Symbol;Acc:7082]
rs167453	X	CD99	HGNC Symbol	CD99 molecule [Source:HGNC Symbol;Acc:7082]
rs184301	X	CD99	HGNC Symbol	CD99 molecule [Source:HGNC Symbol;Acc:7082]
rs144376982	X	CD99	HGNC Symbol	CD99 molecule [Source:HGNC Symbol;Acc:7082]
rs5982829	X	CD99	HGNC Symbol	CD99 molecule [Source:HGNC Symbol;Acc:7082]
rs28667678	X	CD99	HGNC Symbol	CD99 molecule [Source:HGNC Symbol;Acc:7082]
rs184886870	X	CD99	HGNC Symbol	CD99 molecule [Source:HGNC Symbol;Acc:7082]
rs5982839	X	CD99	HGNC Symbol	CD99 molecule [Source:HGNC Symbol;Acc:7082]
rs28635250	X	CD99	HGNC Symbol	CD99 molecule [Source:HGNC Symbol;Acc:7082]
rs1136447	X	CD99	HGNC Symbol	CD99 molecule [Source:HGNC Symbol;Acc:7082]
rs311072	X	CD99	HGNC Symbol	CD99 molecule [Source:HGNC Symbol;Acc:7082]
rs6641640	X	CD99	HGNC Symbol	CD99 molecule [Source:HGNC Symbol;Acc:7082]
rs5982579	X	CD99	HGNC Symbol	CD99 molecule [Source:HGNC Symbol;Acc:7082]
rs7049560	X	CD99	HGNC Symbol	CD99 molecule [Source:HGNC Symbol;Acc:7082]
rs189833710	X	CD99	HGNC Symbol	CD99 molecule [Source:HGNC Symbol;Acc:7082]
rs5939307	X	CD99	HGNC Symbol	CD99 molecule [Source:HGNC Symbol;Acc:7082]
rs5939113	X	CD99	HGNC Symbol	CD99 molecule [Source:HGNC Symbol;Acc:7082]
rs3828932	X	CD99	HGNC Symbol	CD99 molecule [Source:HGNC Symbol;Acc:7082]
rs17808254	X	CD99	HGNC Symbol	CD99 molecule [Source:HGNC Symbol;Acc:7082]
rs1136470	X	CD99	HGNC Symbol	CD99 molecule [Source:HGNC Symbol;Acc:7082]
rs112675992	X	CD99	HGNC Symbol	CD99 molecule [Source:HGNC Symbol;Acc:7082]
rs311088	X	CD99	HGNC Symbol	CD99 molecule [Source:HGNC Symbol;Acc:7082]
rs41311467	X	CD99	HGNC Symbol	CD99 molecule [Source:HGNC Symbol;Acc:7082]
rs311093	X	CD99	HGNC Symbol	CD99 molecule [Source:HGNC Symbol;Acc:7082]
rs311094	X	CD99	HGNC Symbol	CD99 molecule [Source:HGNC Symbol;Acc:7082]

SNP	Chromosome	gene name	gene source	description
rs35939958	X	CD99	HGNC Symbol	CD99 molecule [Source:HGNC Symbol;Acc:7082]
rs312250				
rs73188898				
rs311102				
rs67274880				
rs146821057				
rs5939313	X	XG	HGNC Symbol	Xg blood group [Source:HGNC Symbol;Acc:12806]
rs73190916	X	XG	HGNC Symbol	Xg blood group [Source:HGNC Symbol;Acc:12806]
rs28427303	X	XG	HGNC Symbol	Xg blood group [Source:HGNC Symbol;Acc:12806]
rs312232	X	XG	HGNC Symbol	Xg blood group [Source:HGNC Symbol;Acc:12806]
rs311115	X	XG	HGNC Symbol	Xg blood group [Source:HGNC Symbol;Acc:12806]
rs66607823	X	XG	HGNC Symbol	Xg blood group [Source:HGNC Symbol;Acc:12806]
rs311121	X	XG	HGNC Symbol	Xg blood group [Source:HGNC Symbol;Acc:12806]
rs311125	X	XG	HGNC Symbol	Xg blood group [Source:HGNC Symbol;Acc:12806]
rs73190930	X	XG	HGNC Symbol	Xg blood group [Source:HGNC Symbol;Acc:12806]
rs311128	X	XG	HGNC Symbol	Xg blood group [Source:HGNC Symbol;Acc:12806]
rs149038203	X	XG	HGNC Symbol	Xg blood group [Source:HGNC Symbol;Acc:12806]
rs2535447	X	XG	HGNC Symbol	Xg blood group [Source:HGNC Symbol;Acc:12806]
rs311142	X	XG	HGNC Symbol	Xg blood group [Source:HGNC Symbol;Acc:12806]
rs28758440	X	XG	HGNC Symbol	Xg blood group [Source:HGNC Symbol;Acc:12806]
rs311150	X	XG	HGNC Symbol	Xg blood group [Source:HGNC Symbol;Acc:12806]
rs35446509	X	XG	HGNC Symbol	Xg blood group [Source:HGNC Symbol;Acc:12806]
rs311157	X	XG	HGNC Symbol	Xg blood group [Source:HGNC Symbol;Acc:12806]
rs2259750	X	XG	HGNC Symbol	Xg blood group [Source:HGNC Symbol;Acc:12806]
rs2857319	X	XG	HGNC Symbol	Xg blood group [Source:HGNC Symbol;Acc:12806]
rs2857316	X	XG	HGNC Symbol	Xg blood group [Source:HGNC Symbol;Acc:12806]
rs2534635	X	XG	HGNC Symbol	Xg blood group [Source:HGNC Symbol;Acc:12806]
