# Supplementary material for: GWAS of Post-Orthodontic Aggressive External Apical Root Resorption Identified Multiple Putative Loci at X-Y Chromosomes
Source: J Pers Med. 2020 Oct 14;10(4):169. doi: 10.3390/jpm10040169 (PMC7712155; doi:10.3390/jpm10040169)
Supplement: Supplementary file 1 [file jpm-10-00169-s001.zip › SUPPL INFO FILE 5.pdf]

Supporting Information File 5 . SNPs marginally associated with aEARR [$p < .05$] at chromosomes 2, 4, 8, 12, 18, X and Y.

	SNP	OR	lower	upper	*p* -value	FDR	Chromosome	gene name	gene source	Description
Male (unadjusted)	rs61978642	10,2	1,04	100,47	0,02871442	0,894103771	X	ARSH	HGNC Symbol	arylsulfatase family, member H [Source:HGNC Symbol;Acc:32488]
	rs112655645	10,2	1,04	100,47	0,02871442	0,894103771	X	ARSF	HGNC Symbol	arylsulfatase F [Source:HGNC Symbol;Acc:721]
	rs4892924	0,37	0,17	0,76	0,00164205	0,894103771				
	rs5982670	0,38	0,18	0,8	0,00510629	0,894103771				
	rs1194776	1,9	1,26	2,88	0,00129717	0,894103771				
	rs12559033	2,35	1,07	5,16	0,03712002	0,894103771	X	CXorf28	HGNC Symbol	chromosome X open reading frame 28 [Source:HGNC Symbol;Acc:27336]
	rs73175543	0	0		0,00819276	0,894103771				
	rs1989994	0,28	0,06	1,22	0,04779993	0,894103771				
	rs11152524	0,47	0,22	1,02	0,0475075	0,894103771				
	rs150932516	1,59	1,09	2,33	0,02723687	0,894103771				

SNP	OR	lower	upper	p-value	FDR	Chromosome	gene name	gene source	Description
rs138239409	3,84	1,54	9,54	0,0045266	0,894103771	X	PRKX	HGNC Symbol	protein kinase, X-linked [Source:HGNC Symbol;Acc:9441]
rs6641804	4,24	1,61	11,17	0,00402187	0,894103771	X	PRKX	HGNC Symbol	protein kinase, X-linked [Source:HGNC Symbol;Acc:9441]
rs12839071	0,64	0,46	0,9	0,02056321	0,894103771	X	PRKX	HGNC Symbol	protein kinase, X-linked [Source:HGNC Symbol;Acc:9441]
rs17331167	2,71	1,29	5,68	0,00981945	0,894103771	X	PRKX	HGNC Symbol	protein kinase, X-linked [Source:HGNC Symbol;Acc:9441]
rs7880937	0,47	0,2	1,11	0,01923184	0,894103771	X	PRKX	HGNC Symbol	protein kinase, X-linked [Source:HGNC Symbol;Acc:9441]
rs6641847	1,69	1,12	2,55	0,00726552	0,894103771	X	PRKX	HGNC Symbol	protein kinase, X-linked [Source:HGNC Symbol;Acc:9441]
rs3876184	0,41	0,2	0,85	0,01293024	0,894103771				
rs111602303	3,44	1,5	7,89	0,00428938	0,894103771				
rs17313406	2,47	1,2	5,07	0,01561607	0,894103771				
rs28465437	1,46	1,05	2,03	0,01290273	0,894103771				
rs62575757	1,42	1,02	1,98	0,01685315	0,894103771				
rs5916684	0,61	0,43	0,86	0,00172978	0,894103771				
rs5915430	1,58	1,14	2,2	0,00379373	0,894103771	X	RP11-706O15.1	Clone-based (Vega)	HCG1981372, isoform CRA_c; Uncharacterized protein [Source:UniProtKB/TrEMBL;Acc:B1B108]
rs6640754	1,54	1,08	2,18	0,00810729	0,894103771				

SNP	OR	lower	upper	p-value	FDR	Chromosome	gene name	gene source	Description
rs73180343	2,48	1,29	4,79	0,00439102	0,894103771				
rs6638512	0,33	0,16	0,68	0,00163975	0,894103771				
rs5961309	2,27	1,12	4,58	0,02431396	0,894103771				
rs5916050	0,6	0,34	1,08	0,02944042	0,894103771				
rs73451282	3,09	1,06	9,04	0,04444686	0,894103771				
rs62588650	6,91	1,22	38,98	0,02347917	0,894103771				
rs55909210	4,93	1,62	15,04	0,00526808	0,894103771				
rs114443125	4,93	1,62	15,04	0,00526808	0,894103771				
rs6530010	0,7	0,47	1,04	0,02501985	0,894103771				
rs4830729	0,68	0,46	1,01	0,02122409	0,894103771				
rs5978827	0,72	0,48	1,09	0,04895456	0,894103771				
rs5978220	0,72	0,48	1,09	0,04895456	0,894103771	X	HDHD1	HGNC Symbol	haloacid dehalogenase-like hydrolase domain containing 1 [Source:HGNC Symbol;Acc:16818]
rs151122193	1,66	0,92	3	0,04457297	0,894103771				
rs6639942	0,43	0,19	0,98	0,03440819	0,894103771				

SNP	OR	lower	upper	p-value	FDR	Chromosome	gene name	gene source	Description
rs5933631	0,68	0,43	1,07	0,03113675	0,894103771				
rs5978854	0,59	0,33	1,06	0,02384999	0,894103771				
rs12008804	0,33	0,09	1,14	0,04790069	0,894103771	X	KAL1	HGNC Symbol	Kallmann syndrome 1 sequence [Source:HGNC Symbol;Acc:6211]
rs144420093	3,2	0,91	11,24	0,04244904	0,894103771				
rs1232116	1,42	1,02	1,97	0,01878773	0,894103771				
rs10521598	2,35	1,13	4,88	0,02424036	0,894103771				
rs1395790	0,41	0,16	1,03	0,04133245	0,894103771				
rs62581812	0	0		0,00467354	0,894103771	X	FAM9B	HGNC Symbol	family with sequence similarity 9, member B [Source:HGNC Symbol;Acc:18404]
rs1466984	0,68	0,46	0,99	0,01895637	0,894103771	X	FAM9B	HGNC Symbol	family with sequence similarity 9, member B [Source:HGNC Symbol;Acc:18404]
rs35912577	0	0		0,02417139	0,894103771	X	FAM9B	HGNC Symbol	family with sequence similarity 9, member B [Source:HGNC Symbol;Acc:18404]
rs73186314	0,62	0,42	0,92	0,03421389	0,894103771				
rs2405536	1,43	0,99	2,05	0,02948415	0,894103771				
rs5934617	0,28	0,08	0,97	0,02115554	0,894103771				
rs2040616	4,46	1,15	17,37	0,0330227	0,894103771	X	TBL1X	HGNC Symbol	transducin (beta)-like 1X-linked [Source:HGNC Symbol;Acc:11585]

SNP	OR	lower	upper	p-value	FDR	Chromosome	gene name	gene source	Description
rs146927570	1,88	1,13	3,11	0,01228084	0,894103771	X	SHROOM2	HGNC Symbol	shroom family member 2 [Source:HGNC Symbol;Acc:630]
rs2405942	0,71	0,46	1,1	0,04264399	0,894103771	X	SHROOM2	HGNC Symbol	shroom family member 2 [Source:HGNC Symbol;Acc:630]
rs79628572	0	0		0,04108943	0,894103771	X	SHROOM2	HGNC Symbol	shroom family member 2 [Source:HGNC Symbol;Acc:630]
rs5979272	0,63	0,42	0,94	0,03476604	0,894103771	X	CLCN4	HGNC Symbol	chloride channel, voltage-sensitive 4 [Source:HGNC Symbol;Acc:2022]
rs11795877	0,59	0,37	0,96	0,01080455	0,894103771	X	CLCN4	HGNC Symbol	chloride channel, voltage-sensitive 4 [Source:HGNC Symbol;Acc:2022]
rs5934812	2,01	1,04	3,9	0,03619587	0,894103771	X	CLCN4	HGNC Symbol	chloride channel, voltage-sensitive 4 [Source:HGNC Symbol;Acc:2022]
rs1012782	0,5	0,3	0,84	0,00141473	0,894103771	X	RP6-1O2.1	Clone-based (Vega)	
rs17327648	0,6	0,39	0,95	0,00859112	0,894103771	X	RP6-1O2.1	Clone-based (Vega)	
rs5979305	0,44	0,21	0,92	0,02317738	0,894103771				
rs2525070	0,39	0,16	0,93	0,02267493	0,894103771	X	MID1	HGNC Symbol	midline 1 (Opitz/BBB syndrome) [Source:HGNC Symbol;Acc:7095]
rs7878745	0,26	0,06	1,15	0,03733431	0,894103771	X	MID1	HGNC Symbol	midline 1 (Opitz/BBB syndrome) [Source:HGNC Symbol;Acc:7095]
rs6530417	0,25	0,06	1,09	0,02904938	0,894103771	X	RP11-120D5.1	Clone-based (Vega)	
rs12838825	1,45	1,01	2,07	0,03156042	0,894103771	X	FRMPD4	HGNC Symbol	FERM and PDZ domain containing 4 [Source:HGNC Symbol;Acc:29007]
rs5933977	0,61	0,41	0,9	0,00791854	0,894103771	X	FRMPD4	HGNC Symbol	FERM and PDZ domain containing 4 [Source:HGNC Symbol;Acc:29007]

SNP	OR	lower	upper	p-value	FDR	Chromosome	gene name	gene source	Description
rs5933996	0,7	0,5	0,98	0,01673196	0,894103771	X	FRMPD4	HGNC Symbol	FERM and PDZ domain containing 4 [Source:HGNC Symbol;Acc:29007]
rs55761104	1,99	1,1	3,58	0,01562548	0,894103771	X	FRMPD4	HGNC Symbol	FERM and PDZ domain containing 4 [Source:HGNC Symbol;Acc:29007]
rs5978532	0,18	0,02	1,41	0,03890189	0,894103771	X	FRMPD4	HGNC Symbol	FERM and PDZ domain containing 4 [Source:HGNC Symbol;Acc:29007]
rs6419058	1,55	1,1	2,18	0,02094895	0,894103771	X	FRMPD4	HGNC Symbol	FERM and PDZ domain containing 4 [Source:HGNC Symbol;Acc:29007]
rs149324165	4,26	1,24	14,64	0,02342047	0,894103771	X	FRMPD4	HGNC Symbol	FERM and PDZ domain containing 4 [Source:HGNC Symbol;Acc:29007]
rs73192460	0,72	0,48	1,07	0,03679253	0,894103771	X	FRMPD4	HGNC Symbol	FERM and PDZ domain containing 4 [Source:HGNC Symbol;Acc:29007]
rs5935361	1,47	1,05	2,06	0,02406427	0,894103771	X	FRMPD4	HGNC Symbol	FERM and PDZ domain containing 4 [Source:HGNC Symbol;Acc:29007]
rs12851640	0,64	0,42	0,98	0,03007978	0,894103771				
rs5979841	0,5	0,25	1	0,0461045	0,894103771				
rs17256262	7,19	1,73	29,97	0,00508055	0,894103771	X	GS1-600G8.5	Clone-based (Vega)	
rs4830840	1,49	1,06	2,1	0,03571261	0,894103771	X	GS1-600G8.5	Clone-based (Vega)	
rs5934088	0	0		0,04410144	0,894103771				
rs73192267	3,52	1,08	11,49	0,04144192	0,894103771	X	RP11-142G7.2	Clone-based (Vega)	
rs12689294	0,7	0,43	1,14	0,04547359	0,894103771				

SNP	OR	lower	upper	p-value	FDR	Chromosome	gene name	gene source	Description
rs113081319	0,4	0,09	1,74	0,033512	0,894103771				
rs12844566	1,44	1,03	2	0,01620565	0,894103771				
rs11095629	0,66	0,44	1	0,01446016	0,894103771	X	GPM6B	HGNC Symbol	glycoprotein M6B [Source:HGNC Symbol;Acc:4461]
rs7052395	0,65	0,42	0,99	0,01627293	0,894103771	X	GPM6B	HGNC Symbol	glycoprotein M6B [Source:HGNC Symbol;Acc:4461]
rs1139868	0,33	0,11	0,99	0,02840408	0,894103771	X	ASB9	HGNC Symbol	ankyrin repeat and SOCS box containing 9 [Source:HGNC Symbol;Acc:17184]
rs731197	0,33	0,11	0,99	0,02840408	0,894103771	X	ASB9	HGNC Symbol	ankyrin repeat and SOCS box containing 9 [Source:HGNC Symbol;Acc:17184]
rs5935944	1,54	1,11	2,15	0,00564311	0,894103771	X	ASB11	HGNC Symbol	ankyrin repeat and SOCS box containing 11 [Source:HGNC Symbol;Acc:17186]
rs2071178	1,44	1,03	2	0,01981481	0,894103771	X	PIR	HGNC Symbol	pirin (iron-binding nuclear protein) [Source:HGNC Symbol;Acc:30048]
rs8094	1,54	1,1	2,15	0,00714317	0,894103771	X	PIR	HGNC Symbol	pirin (iron-binding nuclear protein) [Source:HGNC Symbol;Acc:30048]
rs5935984	1,38	0,99	1,93	0,03483295	0,894103771	X	PIR;BMX	HGNC Symbol	pirin (iron-binding nuclear protein) [Source:HGNC Symbol;Acc:30048];BMX non-receptor tyrosine kinase [Source:HGNC Symbol;Acc:1079]
rs798166	1,4	0,98	2,01	0,03395786	0,894103771	X	AP1S2	HGNC Symbol	adaptor-related protein complex 1, sigma 2 subunit [Source:HGNC Symbol;Acc:560]
rs5980202	0,7	0,46	1,05	0,03357578	0,894103771				
rs62585413	1,68	0,93	3,03	0,04223255	0,894103771				
rs4355975	0,48	0,26	0,89	0,01542537	0,894103771				

SNP	OR	lower	upper	p-value	FDR	Chromosome	gene name	gene source	Description
rs66841637	1,58	1,01	2,48	0,03029533	0,894103771	X	CTPS2	HGNC Symbol	CTP synthase 2 [Source:HGNC Symbol;Acc:2520]
rs140173662	1,83	1,04	3,22	0,03193196	0,894103771	X	CTPS2	HGNC Symbol	CTP synthase 2 [Source:HGNC Symbol;Acc:2520]
rs5924607	1,48	1	2,2	0,02752799	0,894103771				
rs4528028	1,83	1,04	3,22	0,03193196	0,894103771				
rs4828523	1,59	1,06	2,38	0,01277572	0,894103771	X	REPS2	HGNC Symbol	RALBP1 associated Eps domain containing 2 [Source:HGNC Symbol;Acc:9963]
rs1365528	1,54	1,03	2,29	0,02453862	0,894103771	X	REPS2	HGNC Symbol	RALBP1 associated Eps domain containing 2 [Source:HGNC Symbol;Acc:9963]
rs12396700	1,59	1,06	2,38	0,01277572	0,894103771	X	REPS2	HGNC Symbol	RALBP1 associated Eps domain containing 2 [Source:HGNC Symbol;Acc:9963]
rs2382815	0,15	0,02	1,15	0,01679493	0,894103771				
rs7878986	0,17	0,02	1,32	0,03031022	0,894103771				
rs5950862	1,6	1,05	2,43	0,01751937	0,894103771				
rs150066059	5,69	1,91	16,96	0,00171584	0,894103771	X	RAI2	HGNC Symbol	retinoic acid induced 2 [Source:HGNC Symbol;Acc:9835]
rs139411880	4,93	1,62	15,04	0,00526808	0,894103771				
rs5909188	2,73	1,07	6,95	0,04046982	0,894103771	X	CDKL5	HGNC Symbol	cyclin-dependent kinase-like 5 [Source:HGNC Symbol;Acc:11411]
rs5955504	2,13	1,1	4,1	0,02344252	0,894103771	X	RS1	HGNC Symbol	retinoschisin 1 [Source:HGNC Symbol;Acc:10457]

SNP	OR	lower	upper	p-value	FDR	Chromosome	gene name	gene source	Description
rs7881721	0,43	0,21	0,9	0,0190025	0,894103771				
rs5990881	0,16	0,02	1,24	0,0229508	0,894103771				
rs5990821	5,69	1,91	16,96	0,00171584	0,894103771				
rs5990929	3,72	1,38	10	0,0108826	0,894103771				
rs1900730	3,72	1,38	10	0,0108826	0,894103771				
rs5951709	2,69	1,39	5,22	0,00334156	0,894103771	X	PHEX	HGNC Symbol	phosphate regulating endopeptidase homolog, X-linked [Source:HGNC Symbol;Acc:8918]
rs6633525	1,56	1,12	2,18	0,00465174	0,894103771	X	PHEX	HGNC Symbol	phosphate regulating endopeptidase homolog, X-linked [Source:HGNC Symbol;Acc:8918]
rs12396173	10,79	2,1	55,4	0,00177744	0,894103771	X	PHEX	HGNC Symbol	phosphate regulating endopeptidase homolog, X-linked [Source:HGNC Symbol;Acc:8918]
rs112585845	2,71	1,07	6,9	0,0418623	0,894103771	X	PHEX	HGNC Symbol	phosphate regulating endopeptidase homolog, X-linked [Source:HGNC Symbol;Acc:8918]
rs4824168	1,34	0,96	1,85	0,03731798	0,894103771	X	RP11-40F8.2	Clone-based (Vega)	
rs56196258	0,69	0,48	1	0,02055283	0,894103771	X	RP11-40F8.2	Clone-based (Vega)	
rs7057115	2,49	1,13	5,49	0,027461	0,894103771	X	RP11-494I9.2;RP11-40F8.2	Clone-based (Vega)	
rs67802966	0,26	0,1	0,71	0,00291432	0,894103771	X	RP11-494I9.2;RP11-40F8.2	Clone-based (Vega)	
rs12399184	0,48	0,23	0,98	0,03696824	0,894103771	X	RP11-494I9.2;RP11-40F8.2	Clone-based (Vega)	

SNP	OR	lower	upper	p-value	FDR	Chromosome	gene name	gene source	Description
rs61463999	0	0		0,00265632	0,894103771	X	RP11-40F8.2	Clone-based (Vega)	
rs6528188	1,32	0,95	1,84	0,04595843	0,894103771	X	RP11-40F8.2	Clone-based (Vega)	
rs34934530	0,7	0,5	0,99	0,01672751	0,894103771	X	RP11-40F8.2	Clone-based (Vega)	
rs66476440	1,47	1,02	2,12	0,02655157	0,894103771	X	RP11-40F8.2	Clone-based (Vega)	
rs11094865	0,39	0,19	0,81	0,00795434	0,894103771	X	RP11-40F8.2	Clone-based (Vega)	
rs142914756	0,33	0,13	0,83	0,00998298	0,894103771	X	RP11-40F8.2	Clone-based (Vega)	
rs67994587	1,52	1,05	2,2	0,03035465	0,894103771	X	RP11-40F8.2	Clone-based (Vega)	
rs67040061	1,56	1,07	2,28	0,01133395	0,894103771	X	RP11-40F8.2	Clone-based (Vega)	
rs12556171	1,36	0,97	1,92	0,04368987	0,894103771	X	RP11-40F8.2	Clone-based (Vega)	
rs5926265	0,34	0,16	0,74	0,00354356	0,894103771	X	RP11-40F8.2	Clone-based (Vega)	
rs6629602	4,43	1,14	17,25	0,03378488	0,894103771	X	RP11-40F8.2	Clone-based (Vega)	
rs5925793	1,38	0,98	1,93	0,03851945	0,894103771				
rs578606	0,31	0,07	1,34	0,0147159	0,894103771				
rs2665363	0,34	0,08	1,47	0,02394446	0,894103771				

SNP	OR	lower	upper	p-value	FDR	Chromosome	gene name	gene source	Description
rs34092038	4,26	1,24	14,64	0,02342047	0,894103771	X	RP13-314C10.5	Clone-based (Vega)	
rs58123806	3,12	1,07	9,1	0,04320988	0,894103771	X	RP13-314C10.5	Clone-based (Vega)	
rs73197327	5,85	1,34	25,48	0,01705025	0,894103771	X	KLHL15	HGNC Symbol	kelch-like family member 15 [Source:HGNC Symbol;Acc:29347]
rs5970824	1,55	1,09	2,19	0,01478661	0,894103771	X	KLHL15	HGNC Symbol	kelch-like family member 15 [Source:HGNC Symbol;Acc:29347]
rs73203501	4,21	1,34	13,22	0,01526178	0,894103771				
rs111323038	5,49	1,48	20,37	0,01073835	0,894103771				
rs113045766	5,96	1,85	19,21	0,00254745	0,894103771				
rs146194002	10,86	2,11	55,77	0,00172526	0,894103771				
rs12689847	2,2	1,19	4,08	0,00859538	0,894103771	X	PCYT1B	HGNC Symbol	phosphate cytidylyltransferase 1, choline, beta [Source:HGNC Symbol;Acc:8755]
rs11573406	6,91	1,22	38,98	0,02347917	0,894103771	X	POLA1	HGNC Symbol	polymerase (DNA directed), alpha 1, catalytic subunit [Source:HGNC Symbol;Acc:9173]
rs73209032	7,37	1,77	30,73	0,00460282	0,894103771				
rs959644	1,57	1,12	2,21	0,00766833	0,894103771				
rs73195057	0,67	0,47	0,96	0,01234883	0,894103771				
rs5986536	10,2	1,04	100,47	0,02871442	0,894103771				

SNP	OR	lower	upper	p-value	FDR	Chromosome	gene name	gene source	Description
rs67066022	0,17	0,02	1,32	0,03031022	0,894103771				
rs140120359	0	0		0,00833543	0,894103771				
rs148361973	4,36	1,12	16,95	0,03565503	0,894103771				
rs141815299	0,15	0,02	1,16	0,01733168	0,894103771				
rs73204844	8,84	1,66	47,15	0,00651971	0,894103771				
rs5926847	0,75	0,53	1,05	0,03666799	0,894103771	X	DCAF8L2	HGNC Symbol	DDB1 and CUL4 associated factor 8-like 2 [Source:HGNC Symbol;Acc:31811]
rs5971305	0,71	0,48	1,06	0,03734512	0,894103771				
rs1234509	0,52	0,27	1,03	0,0164849	0,894103771				
rs3848956	0,7	0,46	1,05	0,03357578	0,894103771				
rs6628345	0,68	0,43	1,07	0,03113675	0,894103771				
rs5985868	0,69	0,46	1,03	0,02188086	0,894103771				
rs4893551	1,57	1,11	2,22	0,02023158	0,894103771				
rs1384575	0	0		0,02466358	0,894103771	X	IL1RAPL1	HGNC Symbol	interleukin 1 receptor accessory protein-like 1 [Source:HGNC Symbol;Acc:5996]
rs140795074	1,67	0,92	3,01	0,04386164	0,894103771	X	IL1RAPL1	HGNC Symbol	interleukin 1 receptor accessory protein-like 1 [Source:HGNC Symbol;Acc:5996]

SNP	OR	lower	upper	p-value	FDR	Chromosome	gene name	gene source	Description
rs6628511	0,43	0,19	0,95	0,02753597	0,894103771				
rs5929084	0,64	0,44	0,93	0,03838643	0,894103771				
rs7062500	1,43	1	2,03	0,02382721	0,894103771				
rs60676446	0,45	0,24	0,84	0,00746476	0,894103771				
rs17282991	0,59	0,39	0,88	0,00292966	0,894103771				
rs5927576	0,72	0,49	1,05	0,04149395	0,894103771				
rs5927624	0,4	0,15	1,07	0,0484235	0,894103771				
rs12860337	0,43	0,18	1,02	0,04216059	0,894103771	X	TAB3	HGNC Symbol	TGF-beta activated kinase 1/MAP3K7 binding protein 3 [Source:HGNC Symbol;Acc:30681]
rs7067182	0,12	0,02	0,92	0,00549087	0,894103771	X	TAB3	HGNC Symbol	TGF-beta activated kinase 1/MAP3K7 binding protein 3 [Source:HGNC Symbol;Acc:30681]
rs62588738	1,48	1,06	2,07	0,02239326	0,894103771				
rs5927762	1,32	0,95	1,83	0,04496029	0,894103771	X	DMD	HGNC Symbol	dystrophin [Source:HGNC Symbol;Acc:2928]
rs1540705	0,47	0,22	1,02	0,04751768	0,894103771	X	DMD	HGNC Symbol	dystrophin [Source:HGNC Symbol;Acc:2928]
rs2646305	2,05	1,05	3,99	0,03571208	0,894103771	X	DMD	HGNC Symbol	dystrophin [Source:HGNC Symbol;Acc:2928]
rs5972472	4,36	1,12	16,95	0,03565503	0,894103771	X	DMD	HGNC Symbol	dystrophin [Source:HGNC Symbol;Acc:2928]

SNP	OR	lower	upper	p-value	FDR	Chromosome	gene name	gene source	Description
rs11095222	2,85	1,16	7,01	0,02603307	0,894103771	X	DMD	HGNC Symbol	dystrophin [Source:HGNC Symbol;Acc:2928]
rs5927938	0,73	0,51	1,04	0,03387844	0,894103771	X	DMD	HGNC Symbol	dystrophin [Source:HGNC Symbol;Acc:2928]
rs145272492	0	0		0,01815726	0,894103771	X	DMD	HGNC Symbol	dystrophin [Source:HGNC Symbol;Acc:2928]
rs7058785	0	0		0,04241486	0,894103771	X	DMD	HGNC Symbol	dystrophin [Source:HGNC Symbol;Acc:2928]
rs111826558	3,78	1,74	8,21	0,00095932	0,894103771	X	DMD	HGNC Symbol	dystrophin [Source:HGNC Symbol;Acc:2928]
rs1456727	2,46	1,18	5,13	0,01822609	0,894103771	X	DMD	HGNC Symbol	dystrophin [Source:HGNC Symbol;Acc:2928]
rs1456729	2,71	1,29	5,68	0,00981945	0,894103771	X	DMD	HGNC Symbol	dystrophin [Source:HGNC Symbol;Acc:2928]
rs1408983	2,99	1,33	6,74	0,00974687	0,894103771	X	DMD	HGNC Symbol	dystrophin [Source:HGNC Symbol;Acc:2928]
rs12007028	0,64	0,38	1,08	0,03957057	0,894103771	X	DMD	HGNC Symbol	dystrophin [Source:HGNC Symbol;Acc:2928]
rs331317	0,67	0,47	0,94	0,03177443	0,894103771	X	DMD	HGNC Symbol	dystrophin [Source:HGNC Symbol;Acc:2928]
rs5972699	2,11	1,08	4,1	0,02590232	0,894103771	X	DMD	HGNC Symbol	dystrophin [Source:HGNC Symbol;Acc:2928]
rs9887516	0,46	0,21	1	0,04167529	0,894103771	X	DMD	HGNC Symbol	dystrophin [Source:HGNC Symbol;Acc:2928]
rs5005249	1,43	1,01	2,02	0,04739478	0,894103771	X	DMD	HGNC Symbol	dystrophin [Source:HGNC Symbol;Acc:2928]
rs17338877	0,33	0,12	0,92	0,01313249	0,894103771	X	DMD	HGNC Symbol	dystrophin [Source:HGNC Symbol;Acc:2928]

SNP	OR	lower	upper	p-value	FDR	Chromosome	gene name	gene source	Description
rs67636309	0,3	0,09	1,03	0,03007327	0,894103771	X	DMD	HGNC Symbol	dystrophin [Source:HGNC Symbol;Acc:2928]
rs3006092	0,3	0,09	1,03	0,03007327	0,894103771				
rs17309863	0,38	0,09	1,63	0,03893986	0,894103771	X	RP11-545D19.1	Clone-based (Vega)	
rs5973200	0,65	0,42	1,03	0,02065981	0,894103771				
rs11095347	1,56	1,05	2,32	0,04850185	0,894103771				
rs17273427	1,37	0,99	1,91	0,02744584	0,894103771				
rs34913508	0,52	0,22	1,23	0,03002123	0,894103771	X	TSPAN7;TM4SF2	HGNC Symbol;UniProtKB	tetraspanin 7 [Source:HGNC Symbol;Acc:11854];Uncharacterized protein; cDNA FLJ59144, highly similar to Tetraspanin-7
rs11266242	0,7	0,43	1,14	0,04547359	0,894103771				
rs112906393	2,98	1,15	7,7	0,02828705	0,894103771	X	RP11-265P11.1	Clone-based (Vega)	
rs4827166	1,34	0,94	1,9	0,0472754	0,894103771				
rs138436947	0	0		0,04147239	0,894103771				
rs584465	0,48	0,25	0,94	0,02991992	0,894103771				
rs150222	0,48	0,25	0,93	0,0284521	0,894103771				
rs114454893	4,36	1,12	16,95	0,03565503	0,894103771				

SNP	OR	lower	upper	p-value	FDR	Chromosome	gene name	gene source	Description
rs140911585	5,36	1,44	19,87	0,01180561	0,894103771	X	MIR1587	HGNC Symbol	microRNA 1587 [Source:HGNC Symbol;Acc:41596]
rs17314146	0,36	0,12	1,07	0,04257126	0,894103771				
rs5917924	2,23	1,02	4,86	0,04922169	0,894103771				
rs5963756	0,33	0,09	1,14	0,04790069	0,894103771				
rs7054029	1,34	0,96	1,86	0,03983354	0,894103771				
rs140319336	2	1,13	3,52	0,03135707	0,894103771				
rs35865200	1,44	1	2,07	0,02836478	0,894103771				
rs5963904	1,34	0,96	1,87	0,03776676	0,894103771				
rs1150527	5,81	1,34	25,31	0,0174589	0,894103771	X	USP9X	HGNC Symbol	ubiquitin specific peptidase 9, X-linked [Source:HGNC Symbol;Acc:12632]
rs56033584	0,18	0,02	1,42	0,0399202	0,894103771	X	USP9X	HGNC Symbol	ubiquitin specific peptidase 9, X-linked [Source:HGNC Symbol;Acc:12632]
rs6610530	2,58	1,25	5,31	0,01160719	0,894103771				
rs150255888	17,79	2,02	156,37	0,00167723	0,894103771				
rs5918155	1,58	1,13	2,2	0,00705178	0,894103771				
rs2056579	1,33	0,95	1,87	0,04044656	0,894103771				

SNP	OR	lower	upper	p-value	FDR	Chromosome	gene name	gene source	Description
rs873336	0,65	0,38	1,1	0,04811707	0,894103771	X	NYX	HGNC Symbol	nyctalopin [Source:HGNC Symbol;Acc:8082]
rs12353585	2,37	1,05	5,33	0,04209693	0,894103771				
rs5950942	1,47	1,06	2,05	0,04449967	0,894103771				
rs150326947	6,91	1,22	38,98	0,02347917	0,894103771				
rs55779415	2	1,06	3,78	0,02351084	0,894103771				
rs6610953	0,35	0,12	1,04	0,03557384	0,894103771	X	FUNDC1	HGNC Symbol	FUN14 domain containing 1 [Source:HGNC Symbol;Acc:28746]
rs59597370	0,67	0,42	1,05	0,02495046	0,894103771				
rs12849327	1,56	1,06	2,3	0,01594242	0,894103771				
rs4335267	1,45	1,04	2,01	0,0277404	0,894103771	X	CXorf36;RP11-342D14.1	HGNC Symbol;Clone-based	chromosome X open reading frame 36 [Source:HGNC Symbol;Acc:25866];
rs41312098	10,13	1,03	99,81	0,02916878	0,894103771	X	RP11-245M24.1	Clone-based (Vega)	
rs5952318	2,1	1,09	4,08	0,02727411	0,894103771	X	RP11-245M24.1	Clone-based (Vega)	
rs1536278	2,02	1,05	3,92	0,03621985	0,894103771	X	RP11-245M24.1	Clone-based (Vega)	
rs143321538	2,01	0,99	4,08	0,03636517	0,894103771				
rs35857391	2,25	1,14	4,44	0,01308185	0,894103771				

SNP	OR	lower	upper	p-value	FDR	Chromosome	gene name	gene source	Description
rs4567179	4,38	1,36	14,14	0,00315229	0,894103771	X	JADE3	HGNC Symbol	jade family PHD finger 3 [Source:HGNC Symbol;Acc:22982]
rs5906330	0,74	0,51	1,07	0,04358106	0,894103771				
rs12852223	0,65	0,45	0,95	0,00998146	0,894103771	X;HG29_PATCH	RBM10	HGNC Symbol	RNA binding motif protein 10 [Source:HGNC Symbol;Acc:9896]
rs6417923	0,74	0,5	1,08	0,04980501	0,894103771	X	CDK16	HGNC Symbol	cyclin-dependent kinase 16 [Source:HGNC Symbol;Acc:8749]
rs7063875	1,44	1,03	2	0,01981481	0,894103771				
rs1142636	1,98	1,02	3,87	0,04541145	0,894103771	X	SYN1	HGNC Symbol	synapsin I [Source:HGNC Symbol;Acc:11494]
rs4824624	2,07	1,05	4,08	0,03783415	0,894103771	X	SYN1	HGNC Symbol	synapsin I [Source:HGNC Symbol;Acc:11494]
rs1998837	1,42	1,01	2	0,0281941	0,894103771	X	ELK1	HGNC Symbol	ELK1, member of ETS oncogene family [Source:HGNC Symbol;Acc:3321]
rs77265236	3,2	0,91	11,24	0,04244904	0,894103771				
rs55839103	0,75	0,53	1,04	0,0381072	0,894103771	X;HG1436_HG1432_PATCH	SSX1	HGNC Symbol	synovial sarcoma, X breakpoint 1 [Source:HGNC Symbol;Acc:11335]
rs41305753	10,2	1,04	100,47	0,02871442	0,894103771	X;HG1436_HG1432_PATCH	GAGE1	HGNC Symbol	G antigen 1 [Source:HGNC Symbol;Acc:4098]
rs5915296	0,35	0,13	0,96	0,0254094	0,894103771	X;HG1433_PATCH	CCNB3	HGNC Symbol	cyclin B3 [Source:HGNC Symbol;Acc:18709]
rs73202119	0	0		0,04097006	0,894103771				
rs10521479	0,54	0,3	0,97	0,00947109	0,894103771				

SNP	OR	lower	upper	p-value	FDR	Chromosome	gene name	gene source	Description
rs149700928	0,68	0,46	1,01	0,04588981	0,894103771				
rs5913856	0,69	0,47	1,03	0,02517591	0,894103771	X	RRAGB	HGNC Symbol	Ras-related GTP binding B [Source:HGNC Symbol;Acc:19901]
rs10855058	0,71	0,49	1,03	0,03033634	0,894103771	X	RP13-188A5.1	Clone-based (Vega)	
rs6417935	0,71	0,46	1,1	0,04264399	0,894103771	X	RP13-188A5.1	Clone-based (Vega)	
rs6521388	0,66	0,45	0,98	0,01734351	0,894103771				
rs73224076	0,18	0,02	1,41	0,03890189	0,894103771	X	FAAH2	HGNC Symbol	fatty acid amide hydrolase 2 [Source:HGNC Symbol;Acc:26440]
rs142169094	1,54	1,05	2,24	0,01623667	0,894103771				
rs146376239	0,39	0,16	0,93	0,02267493	0,894103771				
rs2765951	0,36	0,17	0,78	0,00573231	0,894103771				
rs7888212	0,35	0,16	0,75	0,00451809	0,894103771	X	OPHN1	HGNC Symbol	oligophrenin 1 [Source:HGNC Symbol;Acc:8148]
rs143483183	4,26	1,24	14,64	0,02342047	0,894103771	X	OPHN1	HGNC Symbol	oligophrenin 1 [Source:HGNC Symbol;Acc:8148]
rs5965497	0,43	0,22	0,86	0,01390102	0,894103771	X	OPHN1	HGNC Symbol	oligophrenin 1 [Source:HGNC Symbol;Acc:8148]
rs73212857	0,59	0,39	0,9	0,02095812	0,894103771	X	OPHN1	HGNC Symbol	oligophrenin 1 [Source:HGNC Symbol;Acc:8148]
rs12836444	0,43	0,21	0,9	0,0190025	0,894103771	X	OPHN1	HGNC Symbol	oligophrenin 1 [Source:HGNC Symbol;Acc:8148]

SNP	OR	lower	upper	p-value	FDR	Chromosome	gene name	gene source	Description
rs150294060	1,78	1,08	2,91	0,01547537	0,894103771	X	OPHN1	HGNC Symbol	oligophrenin 1 [Source:HGNC Symbol;Acc:8148]
rs5918825	0,36	0,12	1,08	0,04436136	0,894103771	X	OPHN1	HGNC Symbol	oligophrenin 1 [Source:HGNC Symbol;Acc:8148]
rs5965561	0,3	0,09	1,03	0,03007327	0,894103771	X	OPHN1	HGNC Symbol	oligophrenin 1 [Source:HGNC Symbol;Acc:8148]
rs705896	3,64	1,82	7,27	0,00014713	0,522979691				
rs241396	2,19	1,13	4,22	0,01913367	0,894103771				
rs665542	1,32	0,95	1,83	0,04496029	0,894103771				
rs241750	1,36	0,97	1,89	0,03159044	0,894103771				
rs17302682	0,69	0,49	0,97	0,03112357	0,894103771				
rs7062312	1,45	1,01	2,07	0,03156042	0,894103771				
rs147121494	1,72	1,08	2,74	0,01610734	0,894103771	X	EDA	HGNC Symbol	ectodysplasin A [Source:HGNC Symbol;Acc:3157]
rs2296765	0,69	0,49	0,99	0,01466387	0,894103771	X	EDA	HGNC Symbol	ectodysplasin A [Source:HGNC Symbol;Acc:3157]
rs55933614	0,41	0,2	0,87	0,01523623	0,894103771				
rs62604281	0,4	0,19	0,83	0,01074924	0,894103771				
rs607614	0,68	0,45	1,02	0,02262885	0,894103771				

SNP	OR	lower	upper	p-value	FDR	Chromosome	gene name	gene source	Description
rs150656555	0	0		0,04108943	0,894103771				
rs113548026	1,49	1,03	2,15	0,03768272	0,894103771				
rs1152185	0,53	0,22	1,25	0,04074698	0,894103771				
rs73216714	1,51	1,06	2,16	0,01204403	0,894103771				
rs111499414	2	1,06	3,78	0,02351084	0,894103771	X	DLG3	HGNC Symbol	discs, large homolog 3 (Drosophila) [Source:HGNC Symbol;Acc:2902]
rs73216776	0,71	0,46	1,1	0,04264399	0,894103771	X	TEX11	HGNC Symbol	testis expressed 11 [Source:HGNC Symbol;Acc:11733]
rs7472797	10,43	1,06	102,79	0,02724783	0,894103771				
rs142435968	10,2	1,04	100,47	0,02871442	0,894103771				
rs145367267	0,16	0,02	1,24	0,0229508	0,894103771	X	OGT	HGNC Symbol	O-linked N-acetylglucosamine (GlcNAc) transferase [Source:HGNC Symbol;Acc:8127]
rs78046994	0,22	0,05	0,98	0,01739418	0,894103771	X;HG1438_PATCH	HDAC8	HGNC Symbol	histone deacetylase 8 [Source:HGNC Symbol;Acc:13315]
rs5912109	0,33	0,09	1,14	0,04790069	0,894103771	X;HG1438_PATCH	PHKA1	HGNC Symbol	phosphorylase kinase, alpha 1 (muscle) [Source:HGNC Symbol;Acc:8925]
rs112573145	1,62	1,05	2,5	0,01898734	0,894103771	X	SLC16A2	HGNC Symbol	solute carrier family 16, member 2 (thyroid hormone transporter) [Source:HGNC Symbol;Acc:10923]
rs5912970	1,37	0,97	1,95	0,03973954	0,894103771				
rs4826173	1,33	0,96	1,85	0,04013506	0,894103771				

SNP	OR	lower	upper	p-value	FDR	Chromosome	gene name	gene source	Description
rs115562366	2,61	1,08	6,35	0,03899027	0,894103771				
rs3106407	1,35	0,96	1,9	0,03936713	0,894103771	X	BRWD3	HGNC Symbol	bromodomain and WD repeat domain containing 3 [Source:HGNC Symbol;Acc:17342]
rs3123266	4,26	1,24	14,64	0,02342047	0,894103771				
rs147166358	2,64	1,26	5,53	0,00640729	0,894103771				
rs35199902	3,12	1,07	9,1	0,04320988	0,894103771				
rs7884462	2,52	1,11	5,7	0,03087061	0,894103771				
rs62617489	1,4	0,97	2,01	0,04750118	0,894103771				
rs56035532	1,59	0,99	2,57	0,03012712	0,894103771	X	RP3-326L13.2	Clone-based (Vega)	
rs12841496	1,57	1,13	2,19	0,00695441	0,894103771				
rs148046761	1,84	1,11	3,05	0,0202046	0,894103771				
rs9887469	0	0		0,04108943	0,894103771	X	RPS6KA6	HGNC Symbol	ribosomal protein S6 kinase, 90kDa, polypeptide 6 [Source:HGNC Symbol;Acc:10435]
rs5922934	1,49	1,07	2,07	0,01810041	0,894103771				
rs12688565	1,54	1,1	2,16	0,00521413	0,894103771				
rs6623194	2,71	1,17	6,27	0,01575995	0,894103771				

SNP	OR	lower	upper	p-value	FDR	Chromosome	gene name	gene source	Description
rs7062047	0,47	0,22	1,02	0,04751768	0,894103771				
rs10126146	0,47	0,22	1,02	0,04751768	0,894103771	X	SATL1	HGNC Symbol	spermidine/spermine N1-acetyl transferase-like 1 [Source:HGNC Symbol;Acc:27992]
rs5968458	0,39	0,18	0,81	0,00859314	0,894103771				
rs16980121	0,17	0,02	1,32	0,03031022	0,894103771				
rs17252084	3,2	1,41	7,28	0,00656128	0,894103771				
rs73239767	0,37	0,14	1,02	0,03569948	0,894103771				
rs5923356	1,97	1,02	3,8	0,04177761	0,894103771				
rs2887578	0,69	0,49	0,99	0,015187	0,894103771				
rs4828178	2,15	1,08	4,27	0,03166774	0,894103771				
rs7064044	0,71	0,5	1,01	0,02402702	0,894103771				
rs5969235	1,38	0,99	1,94	0,02535542	0,894103771	X	KLHL4	HGNC Symbol	kelch-like family member 4 [Source:HGNC Symbol;Acc:6355]
rs1321406	0,61	0,34	1,1	0,0369719	0,894103771				
rs2370013	0	0		0,04410144	0,894103771				
rs5969333	0,47	0,27	0,81	0,00380087	0,894103771				

SNP	OR	lower	upper	p-value	FDR	Chromosome	gene name	gene source	Description
rs2507106	0	0		0,04108943	0,894103771				
rs7063284	0,21	0,06	0,72	0,00304584	0,894103771				
rs5924181	0	0		0,01431291	0,894103771				
rs7052636	0,43	0,19	0,98	0,03440819	0,894103771				
rs35490131	2,23	1,15	4,33	0,01859376	0,894103771				
rs1540303	2,18	1,12	4,26	0,02279402	0,894103771				
rs151000882	0,31	0,07	1,34	0,0147159	0,894103771				
rs112899483	0,16	0,02	1,24	0,0229508	0,894103771				
rs142289898	1,56	1,05	2,32	0,04850185	0,894103771				
rs5984703	1,5	1,01	2,23	0,02633105	0,894103771				
rs513756	0,68	0,47	0,98	0,03035078	0,894103771				
rs73242509	1,6	1,13	2,26	0,0118861	0,894103771				
rs138579599	1,87	1,27	2,74	0,00310137	0,894103771				
rs4020637	0,61	0,39	0,96	0,02252869	0,894103771	X	PCDH11X	HGNC Symbol	protocadherin 11 X-linked [Source:HGNC Symbol;Acc:8656]

SNP	OR	lower	upper	p-value	FDR	Chromosome	gene name	gene source	Description
rs2578894	0,64	0,43	0,94	0,0177704	0,894103771				
rs137980088	1,39	0,97	1,98	0,03564744	0,894103771				
rs12841376	0,13	0,02	0,97	0,00734726	0,894103771				
rs785748	0,16	0,02	1,24	0,0229508	0,894103771				
rs785754	0,16	0,02	1,24	0,0229508	0,894103771				
rs1198717	0,15	0,02	1,16	0,01733168	0,894103771				
rs1198718	0,15	0,02	1,16	0,01733168	0,894103771				
rs1045686	1,97	1,01	3,83	0,04713332	0,894103771	X	NAP1L3	HGNC Symbol	nucleosome assembly protein 1-like 3 [Source:HGNC Symbol;Acc:7639]
rs10126690	2,03	1,04	3,95	0,03819647	0,894103771	X	FAM133A	HGNC Symbol	family with sequence similarity 133, member A [Source:HGNC Symbol;Acc:26748]
rs7891218	2,71	1,29	5,68	0,00981945	0,894103771	X	FAM133A	HGNC Symbol	family with sequence similarity 133, member A [Source:HGNC Symbol;Acc:26748]
rs5983168	2,47	1,2	5,07	0,01561607	0,894103771				
rs114786886	1,99	1,05	3,77	0,02409196	0,894103771				
rs4969708	0,33	0,09	1,14	0,04790069	0,894103771				
rs60757162	4,26	1,24	14,64	0,02342047	0,894103771				

SNP	OR	lower	upper	p-value	FDR	Chromosome	gene name	gene source	Description
rs5949599	7,19	1,73	29,97	0,00508055	0,894103771				
rs111335229	13,91	1,52	127,66	0,00705132	0,894103771				
rs35408243	5,85	1,34	25,48	0,01705025	0,894103771	X	DIAPH2	HGNC Symbol	diaphanous-related formin 2 [Source:HGNC Symbol;Acc:2877]
rs400586	0,15	0,02	1,16	0,01733168	0,894103771				
rs5921103	0,68	0,44	1,04	0,02994148	0,894103771				
rs16982961	2,83	1,15	6,96	0,02703764	0,894103771				
rs5920819	6,91	1,22	38,98	0,02347917	0,894103771	X	PCDH19	HGNC Symbol	protocadherin 19 [Source:HGNC Symbol;Acc:14270]
rs28545751	10,2	1,04	100,47	0,02871442	0,894103771	X	PCDH19	HGNC Symbol	protocadherin 19 [Source:HGNC Symbol;Acc:14270]
rs41300169	5,85	1,34	25,48	0,01705025	0,894103771	X	PCDH19	HGNC Symbol	protocadherin 19 [Source:HGNC Symbol;Acc:14270]
rs62600479	6,86	1,22	38,73	0,02394692	0,894103771				
rs1204411	1,42	1,02	1,97	0,02501862	0,894103771	X	SRPX2	HGNC Symbol	sushi-repeat containing protein, X-linked 2 [Source:HGNC Symbol;Acc:30668]
rs73250616	1,52	1,09	2,13	0,01289931	0,894103771				
rs2154370	1,53	1,09	2,16	0,01254542	0,894103771				
rs11092279	0,67	0,47	0,94	0,01735829	0,894103771				

SNP	OR	lower	upper	p-value	FDR	Chromosome	gene name	gene source	Description
rs5920863	0,66	0,47	0,93	0,01400497	0,894103771	X	CSTF2	HGNC Symbol	cleavage stimulation factor, 3' pre-RNA, subunit 2, 64kDa [Source:HGNC Symbol;Acc:2484]
rs4828068	0,56	0,39	0,82	0,00077382	0,894103771	X	NOX1	HGNC Symbol	NADPH oxidase 1 [Source:HGNC Symbol;Acc:7889]
rs5967207	1,74	1,1	2,74	0,02718669	0,894103771				
rs17323346	3,09	1,4	6,83	0,00641417	0,894103771				
rs5921690	1,4	1,01	1,95	0,01700824	0,894103771				
rs12156914	0,62	0,42	0,91	0,02490913	0,894103771	X	TRMT2B-AS1	HGNC Symbol	TRMT2B antisense RNA 1 [Source:HGNC Symbol;Acc:41116]
rs17257634	10,2	1,04	100,47	0,02871442	0,894103771	X	DRP2	HGNC Symbol	dystrophin related protein 2 [Source:HGNC Symbol;Acc:3032]
rs41309506	5,85	1,34	25,48	0,01705025	0,894103771	X	TIMM8A	HGNC Symbol	translocase of inner mitochondrial membrane 8 homolog A (yeast) [Source:HGNC Symbol;Acc:11817]
rs2239460	0,18	0,02	1,42	0,0399202	0,894103771	X	BTK	HGNC Symbol	Bruton agammaglobulinemia tyrosine kinase [Source:HGNC Symbol;Acc:1133]
rs149192215	10,2	1,04	100,47	0,02871442	0,894103771				
rs147068824	0	0		0,04108943	0,894103771	X	NXF4	HGNC Symbol	nuclear RNA export factor 4 pseudogene [Source:HGNC Symbol;Acc:8074]
rs209095	0,64	0,42	0,98	0,03007978	0,894103771	X	NRK	HGNC Symbol	Nik related kinase [Source:HGNC Symbol;Acc:25391]
rs5917052	1,49	1,07	2,07	0,01855571	0,894103771				
rs1012633	0,46	0,19	1,08	0,01108997	0,894103771				

SNP	OR	lower	upper	p-value	FDR	Chromosome	gene name	gene source	Description
rs17254207	1,43	1,03	1,99	0,01715824	0,894103771	X	TSC22D3	HGNC Symbol	TSC22 domain family, member 3 [Source:HGNC Symbol;Acc:3051]
rs5917070	1,35	0,97	1,88	0,03494453	0,894103771	X	NCBP2L	HGNC Symbol	nuclear cap binding protein subunit 2-like [Source:HGNC Symbol;Acc:31795]
rs141633839	1,67	0,93	3,02	0,04290728	0,894103771				
rs5973850	0,66	0,45	0,97	0,02764055	0,894103771	X	COL4A6	HGNC Symbol	collagen, type IV, alpha 6 [Source:HGNC Symbol;Acc:2208]
rs1042071	0,66	0,45	0,97	0,02764055	0,894103771	X	COL4A6	HGNC Symbol	collagen, type IV, alpha 6 [Source:HGNC Symbol;Acc:2208]
rs7061716	2,35	1,09	5,05	0,0201542	0,894103771				
rs5942780	2,35	1,09	5,07	0,01972191	0,894103771				
rs10521523	4,38	1,36	14,14	0,00315229	0,894103771				
rs55676345	3	1,42	6,36	0,00492649	0,894103771				
rs111904771	5,85	1,34	25,48	0,01705025	0,894103771	X	PAK3	HGNC Symbol	p21 protein (Cdc42/Rac)-activated kinase 3 [Source:HGNC Symbol;Acc:8592]
rs7050529	3,12	1,07	9,1	0,04320988	0,894103771	X	TRPC5	HGNC Symbol	transient receptor potential cation channel, subfamily C, member 5 [Source:HGNC Symbol;Acc:12337]
rs140629422	2,63	1,08	6,39	0,03758606	0,894103771	X	TRPC5	HGNC Symbol	transient receptor potential cation channel, subfamily C, member 5 [Source:HGNC Symbol;Acc:12337]
rs73550175	10,2	1,04	100,47	0,02871442	0,894103771				
rs73213486	10,2	1,04	100,47	0,02871442	0,894103771				

SNP	OR	lower	upper	p-value	FDR	Chromosome	gene name	gene source	Description
rs7888133	1,45	1,01	2,07	0,03156042	0,894103771	X;HG1434_PATCH	RP5-964N17.1	Clone-based (Vega)	
rs56296825	1,63	1,17	2,27	0,00691882	0,894103771				
rs5929431	0,63	0,39	1,02	0,04320387	0,894103771				
rs5946131	2,56	1,1	5,99	0,0343008	0,894103771				
rs1537755	3,11	1,25	7,75	0,01741549	0,894103771				
rs112561107	0,58	0,35	0,95	0,047032	0,894103771				
rs5987938	0,38	0,09	1,63	0,03893986	0,894103771	X;HG1462_PATCH	PLS3	HGNC Symbol	plastin 3 [Source:HGNC Symbol;Acc:9091]
rs11091036	2,48	1,22	5,03	0,01326328	0,894103771				
rs61638747	0,68	0,48	0,97	0,02677244	0,894103771				
rs7889653	0,35	0,14	0,87	0,01400471	0,894103771				
rs5952101	3,83	1,15	12,74	0,01188059	0,894103771	X;HG1463_PATCH	SLC6A14	HGNC Symbol;UniProtKB	solute carrier family 6 (amino acid transporter), member 14 [Source:HGNC Symbol;Acc:11047];
rs5958709	0,14	0,02	1,09	0,01305372	0,894103771				
rs7058127	0,57	0,36	0,89	0,00331198	0,894103771				
rs12008294	0,56	0,31	0,99	0,0117504	0,894103771				

SNP	OR	lower	upper	p-value	FDR	Chromosome	gene name	gene source	Description
rs5910238	0,59	0,37	0,96	0,01080455	0,894103771				
rs5956024	0	0		0,00152805	0,894103771				
rs73216861	5,36	1,44	19,87	0,01180561	0,894103771				
rs1294826	0,58	0,37	0,92	0,0365639	0,894103771	X	DOCK11	HGNC Symbol	dedicator of cytokinesis 11 [Source:HGNC Symbol;Acc:23483]
rs5910376	2,08	1,08	4,02	0,02962633	0,894103771	X	DOCK11	HGNC Symbol	dedicator of cytokinesis 11 [Source:HGNC Symbol;Acc:23483]
rs2286977	2,08	1,08	4,02	0,02962633	0,894103771	X	DOCK11	HGNC Symbol	dedicator of cytokinesis 11 [Source:HGNC Symbol;Acc:23483]
rs17326920	0,26	0,06	1,15	0,03733431	0,894103771				
rs3788941	0,57	0,39	0,83	0,00345817	0,894103771	X	ATP1B4	HGNC Symbol	ATPase, Na+/K+ transporting, beta 4 polypeptide [Source:HGNC Symbol;Acc:808]
rs2192257	0,36	0,13	1	0,03735654	0,894103771				
rs6646733	2,64	1,28	5,46	0,00988983	0,894103771				
rs5909764	1,48	1,06	2,08	0,04671217	0,894103771				
rs73221033	3,59	1,19	10,81	0,02665157	0,894103771				
rs5909792	0,65	0,44	0,96	0,01120434	0,894103771				
rs2110461	4,36	1,12	16,95	0,03565503	0,894103771				

SNP	OR	lower	upper	p-value	FDR	Chromosome	gene name	gene source	Description
rs5909870	0,58	0,38	0,89	0,00402385	0,894103771				
rs5956458	0	0		0,02413192	0,894103771				
rs2622953	0	0		0,04410144	0,894103771				
rs35464043	10,2	1,04	100,47	0,02871442	0,894103771				
rs6648836	0,71	0,46	1,1	0,04264399	0,894103771				
rs5958090	0,71	0,46	1,1	0,04264399	0,894103771				
rs4372129	1,32	0,95	1,83	0,04386232	0,894103771				
rs5956496	0,72	0,48	1,09	0,04895456	0,894103771				
rs5909973	1,41	1,01	1,97	0,04329878	0,894103771	X	GRIA3	HGNC Symbol	glutamate receptor, ionotropic, AMPA 3 [Source:HGNC Symbol;Acc:4573]
rs56385541	2,25	1,14	4,44	0,01308185	0,894103771	X	GRIA3	HGNC Symbol	glutamate receptor, ionotropic, AMPA 3 [Source:HGNC Symbol;Acc:4573]
rs5911547	1,5	1,07	2,11	0,02056078	0,894103771	X	GRIA3	HGNC Symbol	glutamate receptor, ionotropic, AMPA 3 [Source:HGNC Symbol;Acc:4573]
rs2157292	1,74	1,1	2,74	0,02718669	0,894103771	X	GRIA3	HGNC Symbol	glutamate receptor, ionotropic, AMPA 3 [Source:HGNC Symbol;Acc:4573]
rs142397190	10,13	1,03	99,81	0,02916878	0,894103771	X	GRIA3	HGNC Symbol	glutamate receptor, ionotropic, AMPA 3 [Source:HGNC Symbol;Acc:4573]
rs4825856	0,29	0,11	0,82	0,00267515	0,894103771	X	GRIA3	HGNC Symbol	glutamate receptor, ionotropic, AMPA 3 [Source:HGNC Symbol;Acc:4573]

SNP	OR	lower	upper	p-value	FDR	Chromosome	gene name	gene source	Description
rs17325366	1,45	1,04	2,01	0,0277404	0,894103771				
rs5956566	1,48	1,05	2,09	0,04216745	0,894103771	X	THOC2	HGNC Symbol	THO complex 2 [Source:HGNC Symbol;Acc:19073]
rs36053352	4,21	1,34	13,22	0,01526178	0,894103771				
rs62604518	1,6	1,07	2,39	0,03350981	0,894103771				
rs3005882	3,11	1,25	7,75	0,01741549	0,894103771				
rs3005851	2,97	1,25	7,09	0,01660396	0,894103771				
rs6608181	1,55	1,11	2,17	0,01914929	0,894103771	X	STAG2	HGNC Symbol	stromal antigen 2 [Source:HGNC Symbol;Acc:11355]
rs5911806	1,8	1,29	2,52	0,00056894	0,894103771	X	STAG2	HGNC Symbol	stromal antigen 2 [Source:HGNC Symbol;Acc:11355]
rs151184635	5,92	2,55	13,72	3,3797E-05	0,480531966	X	STAG2	HGNC Symbol	stromal antigen 2 [Source:HGNC Symbol;Acc:11355]
rs2356408	0,63	0,45	0,9	0,00818242	0,894103771	X	STAG2	HGNC Symbol	stromal antigen 2 [Source:HGNC Symbol;Acc:11355]
rs2072886	0,48	0,25	0,95	0,00622897	0,894103771	X	TENM1;STAG2	HGNC Symbol	teneurin transmembrane protein 1 [Source:HGNC Symbol;Acc:8117];stromal antigen 2 [Source:HGNC Symbol;Acc:11355]
rs111472283	1,61	1,12	2,32	0,00638795	0,894103771	X	TENM1	HGNC Symbol	teneurin transmembrane protein 1 [Source:HGNC Symbol;Acc:8117]
rs2858445	0,17	0,02	1,32	0,03031022	0,894103771	X	TENM1	HGNC Symbol	teneurin transmembrane protein 1 [Source:HGNC Symbol;Acc:8117]
rs5958557	2,16	1,07	4,36	0,0331915	0,894103771	X	TENM1	HGNC Symbol	teneurin transmembrane protein 1 [Source:HGNC Symbol;Acc:8117]

SNP	OR	lower	upper	p-value	FDR	Chromosome	gene name	gene source	Description
rs2206237	3,52	1,08	11,49	0,04144192	0,894103771	X	TENM1	HGNC Symbol	teneurin transmembrane protein 1 [Source:HGNC Symbol;Acc:8117]
rs3859908	4,21	1,34	13,22	0,01526178	0,894103771	X	TENM1	HGNC Symbol	teneurin transmembrane protein 1 [Source:HGNC Symbol;Acc:8117]
rs73215144	13,91	1,52	127,66	0,00705132	0,894103771	X	TENM1	HGNC Symbol	teneurin transmembrane protein 1 [Source:HGNC Symbol;Acc:8117]
rs1569565	4,36	1,12	16,95	0,03565503	0,894103771	X	TENM1	HGNC Symbol	teneurin transmembrane protein 1 [Source:HGNC Symbol;Acc:8117]
rs2213484	1,46	0,98	2,16	0,0298138	0,894103771				
rs16998333	2,42	1,25	4,7	0,00916434	0,894103771				
rs4830000	0,51	0,26	0,99	0,04390521	0,894103771				
rs73216164	4,33	1,11	16,84	0,0364709	0,894103771				
rs2858278	1,42	1,01	1,98	0,04253737	0,894103771				
rs73214455	17,79	2,02	156,37	0,00167723	0,894103771				
rs73214463	17,79	2,02	156,37	0,00167723	0,894103771				
rs1305003	1,42	1,02	1,98	0,03590368	0,894103771				
rs139880202	4,26	1,24	14,64	0,02342047	0,894103771				
rs7056175	0,41	0,16	1,03	0,04133245	0,894103771				

SNP	OR	lower	upper	p-value	FDR	Chromosome	gene name	gene source	Description
rs5975024	4,47	1,92	10,37	0,00059286	0,894103771	X	RP1-30E17.2	Clone-based (Vega)	
rs11796949	0,66	0,44	1	0,01476177	0,894103771	X	RP1-30E17.2	Clone-based (Vega)	
rs55839915	5,98	2,37	15,12	0,00014632	0,522979691	X	RP1-30E17.2	Clone-based (Vega)	
rs5976834	4,4	1,85	10,46	0,00097295	0,894103771				
rs5932462	0,65	0,38	1,1	0,04811707	0,894103771				
rs5975081	2,14	1,04	4,41	0,04320157	0,894103771				
rs737159	2,33	1,08	5	0,0340345	0,894103771				
rs5977001	10,2	1,04	100,47	0,02871442	0,894103771				
rs5930347	3,2	0,91	11,24	0,04244904	0,894103771				
rs73633939	4,2	1,44	12,28	0,00986748	0,894103771				
rs73225593	13,91	1,52	127,66	0,00705132	0,894103771	X	BCORL1	HGNC Symbol	BCL6 corepressor-like 1 [Source:HGNC Symbol;Acc:25657]
rs142984078	0,4	0,09	1,74	0,033512	0,894103771				
rs3788	2,35	1,13	4,88	0,02424036	0,894103771	X	ARHGAP36	HGNC Symbol	Rho GTPase activating protein 36 [Source:HGNC Symbol;Acc:26388]
rs17251489	0,63	0,35	1,14	0,04568756	0,894103771	X	GPC3	HGNC Symbol	glypican 3 [Source:HGNC Symbol;Acc:4451]

SNP	OR	lower	upper	p-value	FDR	Chromosome	gene name	gene source	Description
rs5977998	3,52	1,08	11,49	0,04144192	0,894103771				
rs5976162	1,88	1,13	3,11	0,01228084	0,894103771				
rs72616246	1,56	0,95	2,57	0,04585181	0,894103771	X	FGF13	HGNC Symbol	fibroblast growth factor 13 [Source:HGNC Symbol;Acc:3670]
rs619373	0	0		0,04410144	0,894103771	X	FGF13	HGNC Symbol	fibroblast growth factor 13 [Source:HGNC Symbol;Acc:3670]
rs7880571	1,34	0,97	1,87	0,03841173	0,894103771	X	FGF13	HGNC Symbol	fibroblast growth factor 13 [Source:HGNC Symbol;Acc:3670]
rs145457335	0,4	0,09	1,74	0,033512	0,894103771				
rs12842104	0,63	0,39	1,02	0,04320387	0,894103771				
rs374508	0,59	0,35	1	0,01650904	0,894103771				
rs140150717	1,42	0,97	2,09	0,03608932	0,894103771				
rs4829994	1,51	1,05	2,16	0,01731104	0,894103771				
rs5931666	1,51	1,05	2,16	0,01731104	0,894103771				
rs6048	1,46	1,03	2,07	0,01565096	0,894103771	X	F9	HGNC Symbol	coagulation factor IX [Source:HGNC Symbol;Acc:3551]
rs978818	1,46	0,97	2,19	0,0402281	0,894103771	X	MCF2	HGNC Symbol	MCF.2 cell line derived transforming sequence [Source:HGNC Symbol;Acc:6940]
rs5907587	1,99	1,11	3,59	0,01518958	0,894103771				

SNP	OR	lower	upper	p-value	FDR	Chromosome	gene name	gene source	Description
rs62618215	0,74	0,53	1,03	0,03863564	0,894103771	X	LINC00632	HGNC Symbol	long intergenic non-protein coding RNA 632 [Source:HGNC Symbol;Acc:27865]
rs12556960	0,71	0,46	1,1	0,04264399	0,894103771				
rs12557948	0,52	0,33	0,82	0,00481993	0,894103771				
rs844956	0,62	0,42	0,92	0,01243659	0,894103771				
rs926809	3,81	1,92	7,58	0,00013632	0,522979691				
rs6636278	3,32	1,7	6,49	0,00040729	0,894103771				
rs2864953	0,62	0,42	0,92	0,01243659	0,894103771				
rs17282467	0,47	0,22	0,98	0,03714834	0,894103771				
rs111329029	1,41	1,01	1,97	0,02361863	0,894103771				
rs12390344	2,11	1,07	4,15	0,03339507	0,894103771				
rs12390872	0,66	0,45	0,95	0,02116545	0,894103771				
rs717631	2,16	1,05	4,44	0,04104458	0,894103771				
rs150982418	3,12	1,07	9,1	0,04320988	0,894103771				
rs62600572	17,79	2,02	156,37	0,00167723	0,894103771				

SNP	OR	lower	upper	p-value	FDR	Chromosome	gene name	gene source	Description
rs2224815	0,75	0,53	1,05	0,03749686	0,894103771				
rs5907416	2,2	1,19	4,08	0,00859538	0,894103771	X;HG1458_PATCH	GS1-256O22.5	Clone-based (Vega)	
rs5908648	1,95	1	3,79	0,04637084	0,894103771	X;HG1458_PATCH	GS1-256O22.5	Clone-based (Vega)	
rs5908660	2,07	1,04	4,12	0,03996443	0,894103771	X;HG1458_PATCH	GS1-256O22.5	Clone-based (Vega)	
rs73232180	10,2	1,04	100,47	0,02871442	0,894103771				
rs12013685	6,91	1,22	38,98	0,02347917	0,894103771				
rs73233724	4,23	1,23	14,54	0,02405029	0,894103771				
rs1325103	1,52	0,95	2,44	0,04858541	0,894103771				
rs4827773	0,62	0,42	0,91	0,00951302	0,894103771				
rs5919803	0,62	0,43	0,89	0,00708215	0,894103771				
rs57580842	0,63	0,43	0,92	0,01189092	0,894103771				
rs5966452	0,64	0,45	0,92	0,01277047	0,894103771				
rs6626331	1,97	1,02	3,81	0,0446672	0,894103771				
rs41416052	3,3	1,2	9,13	0,02477728	0,894103771				

SNP	OR	lower	upper	p-value	FDR	Chromosome	gene name	gene source	Description
rs2891670	2,16	1,06	4,4	0,03669399	0,894103771				
rs138367545	5,85	1,34	25,48	0,01705025	0,894103771				
rs146458110	5,45	1,47	20,23	0,01104169	0,894103771				
rs142778097	4,36	1,12	16,95	0,03565503	0,894103771				
rs7052650	1,56	0,95	2,57	0,04585181	0,894103771				
rs147425194	0,16	0,02	1,24	0,0229508	0,894103771				
rs5920027	1,39	1	1,93	0,02362651	0,894103771				
rs6626484	0,33	0,14	0,76	0,00468208	0,894103771				
rs5920062	0,36	0,13	0,99	0,03012882	0,894103771				
rs5920112	0,76	0,55	1,06	0,04689343	0,894103771				
rs5951898	1,38	0,98	1,93	0,03336647	0,894103771				
rs2392722	0,71	0,46	1,1	0,04264399	0,894103771				
rs111830525	0,69	0,44	1,09	0,03710255	0,894103771				
rs11798727	0,59	0,38	0,9	0,00395907	0,894103771				

SNP	OR	lower	upper	p-value	FDR	Chromosome	gene name	gene source	Description
rs73249437	0,52	0,22	1,24	0,04154107	0,894103771	X;HG1459_PATCH	AFF2	HGNC Symbol	AF4/FMR2 family, member 2 [Source:HGNC Symbol;Acc:3776]
rs241132	6,91	1,22	38,98	0,02347917	0,894103771	X;HG1459_PATCH	AFF2	HGNC Symbol	AF4/FMR2 family, member 2 [Source:HGNC Symbol;Acc:3776]
rs12848014	0,56	0,36	0,87	0,01203181	0,894103771	X	AFF2	HGNC Symbol	AF4/FMR2 family, member 2 [Source:HGNC Symbol;Acc:3776]
rs5980419	1,39	0,99	1,95	0,03226754	0,894103771				
rs5925421	2,8	1,1	7,14	0,03597851	0,894103771				
rs34964358	4,26	1,24	14,64	0,02342047	0,894103771				
rs693913	1,68	1,19	2,38	0,00394363	0,894103771	X	MAMLD1	HGNC Symbol	mastermind-like domain containing 1 [Source:HGNC Symbol;Acc:2568]
rs150042509	8,59	2,13	34,7	0,0014357	0,894103771	X	MAMLD1	HGNC Symbol	mastermind-like domain containing 1 [Source:HGNC Symbol;Acc:2568]
rs5925148	0,56	0,37	0,85	0,00684688	0,894103771	X	MAMLD1	HGNC Symbol	mastermind-like domain containing 1 [Source:HGNC Symbol;Acc:2568]
rs598334	0,74	0,52	1,06	0,0341299	0,894103771	X	MAMLD1	HGNC Symbol	mastermind-like domain containing 1 [Source:HGNC Symbol;Acc:2568]
rs56348703	1,41	1	1,99	0,02672613	0,894103771				
rs73250555	0,25	0,06	1,09	0,02904938	0,894103771	X;HG1459_PATCH	MTM1	HGNC Symbol	myotubularin 1 [Source:HGNC Symbol;Acc:7448]
rs5925388	0,72	0,51	1,01	0,0248492	0,894103771	X;HG1459_PATCH	MTM1	HGNC Symbol	myotubularin 1 [Source:HGNC Symbol;Acc:7448]
rs73250569	0,25	0,06	1,09	0,02904938	0,894103771	X;HG1459_PATCH	MTMR1	HGNC Symbol	myotubularin related protein 1 [Source:HGNC Symbol;Acc:7449]

SNP	OR	lower	upper	p-value	FDR	Chromosome	gene name	gene source	Description
rs6877	0,76	0,53	1,08	0,04920442	0,894103771	X;HG1459_PATCH	CD99L2	HGNC Symbol	CD99 molecule-like 2 [Source:HGNC Symbol;Acc:18237]
rs237397	1,37	0,99	1,91	0,0231028	0,894103771				
rs7057942	0,33	0,13	0,82	0,00880915	0,894103771				
rs5924858	0,43	0,18	1,02	0,04216059	0,894103771				
rs5925505	2,01	0,99	4,08	0,03636517	0,894103771				
rs180488	0	0		0,04410144	0,894103771				
rs78972460	2	1,21	3,32	0,01259683	0,894103771				
rs1937215	1,74	1,04	2,93	0,02556772	0,894103771				
rs741725	3,59	1,19	10,81	0,02665157	0,894103771				
rs5970148	1,31	0,94	1,82	0,0495858	0,894103771				
rs73241852	0,53	0,27	1,04	0,01633705	0,894103771				
rs56053914	0,65	0,38	1,1	0,04820937	0,894103771				
rs210567	0,57	0,34	0,97	0,0102174	0,894103771				
rs17320283	0,67	0,41	1,09	0,03071866	0,894103771	X	RP11-329E24.6	Clone-based (Vega)	

SNP	OR	lower	upper	p-value	FDR	Chromosome	gene name	gene source	Description
rs7051108	0,44	0,19	1,02	0,04196205	0,894103771				
rs4833	1,5	1,08	2,1	0,00900367	0,894103771	X;HG1497_PATCH	BGN	HGNC Symbol	biglycan [Source:HGNC Symbol;Acc:1044]
rs80276708	1,37	0,99	1,9	0,02394504	0,894103771	X;HG1497_PATCH	ABCD1	HGNC Symbol	ATP-binding cassette, sub-family D (ALD), member 1 [Source:HGNC Symbol;Acc:61]
rs2070097	0,5	0,25	1	0,0461045	0,894103771	X;HG1497_PATCH	ARHGAP4	HGNC Symbol	Rho GTPase activating protein 4 [Source:HGNC Symbol;Acc:674]
rs73247656	0	0		0,00819276	0,894103771				
rs28370214	0	0		0,02417139	0,894103771	X;HG1497_PATCH	F8	HGNC Symbol	coagulation factor VIII, procoagulant component [Source:HGNC Symbol;Acc:3546]
rs306890	0,54	0,3	0,97	0,03716959	0,894103771				
rs55857040	10,2	1,04	100,47	0,02871442	0,894103771	X	SPRY3	HGNC Symbol	sprouty homolog 3 (Drosophila) [Source:HGNC Symbol;Acc:11271]
rs28729587	2,32	1,24	4,33	0,00910932	0,894103771	X	SPRY3	HGNC Symbol	sprouty homolog 3 (Drosophila) [Source:HGNC Symbol;Acc:11271]
rs77442791	0,46	0,24	0,89	0,01519468	0,894103771	X	SPRY3	HGNC Symbol	sprouty homolog 3 (Drosophila) [Source:HGNC Symbol;Acc:11271]
rs306873	0,62	0,39	1	0,04502159	0,894103771	X	SPRY3	HGNC Symbol	sprouty homolog 3 (Drosophila) [Source:HGNC Symbol;Acc:11271]
rs700462	1,74	1,08	2,82	0,02283275	0,894103771				
rs802480	1,82	1,13	2,94	0,01333428	0,894103771				
rs35519384	2,41	1,23	4,7	0,01096737	0,894103771				

SNP	OR	lower	upper	p-value	FDR	Chromosome	gene name	gene source	Description
rs1883079	0,18	0,02	1,42	0,0399202	0,894103771	X	IL9R	HGNC Symbol	interleukin 9 receptor [Source:HGNC Symbol;Acc:6030]
rs3093493	0,63	0,29	1,35	0,03299386	0,894103771	X	IL9R	HGNC Symbol	interleukin 9 receptor [Source:HGNC Symbol;Acc:6030]
rs6644961	1,14	0,66	1,97	0,01690409	0,894103771				
rs7892580	0,43	0,2	0,91	0,01738022	0,894103771	X	PLCXD1	HGNC Symbol	phosphatidylinositol-specific phospholipase C, X domain containing 1 [Source:HGNC Symbol;Acc:23148]
rs5950688	0,51	0,28	0,95	0,02535697	0,894103771				
rs28599889	0,56	0,33	0,97	0,03314952	0,894103771				
rs61080188	1,71	1,04	2,8	0,03226586	0,894103771				
rs6645165	0,59	0,36	0,96	0,02847291	0,894103771				
rs35099437	1,87	1,13	3,08	0,01262782	0,894103771	X	SHOX	HGNC Symbol	short stature homeobox [Source:HGNC Symbol;Acc:10853]
rs6579694	0,58	0,36	0,95	0,02717904	0,894103771				
rs2037897	0,5	0,29	0,88	0,0109329	0,894103771				
rs5988645	2,63	1,45	4,77	0,00146535	0,894103771				
rs4300160	0,47	0,11	2,04	0,0147159	0,894103771				
rs5946521	1,54	0,86	2,78	0,02986237	0,894103771				

SNP	OR	lower	upper	p-value	FDR	Chromosome	gene name	gene source	Description
rs5946343	1,97	1,03	3,76	0,0041208	0,894103771				
rs28780986	1,77	1,06	2,95	0,02875458	0,894103771				
rs5946570	1,65	1,06	2,59	0,02621589	0,894103771				
rs7885174	1,85	1,16	2,95	0,01011634	0,894103771				
rs4131911	0	0		0,04097006	0,894103771				
rs34453751	1,93	1,14	3,26	0,0140881	0,894103771				
rs35811834	2,29	1,33	3,93	0,00261589	0,894103771				
rs62605876	1,76	1,07	2,9	0,02610839	0,894103771				
rs67734326	1,57	1	2,48	0,04871715	0,894103771				
rs34745620	1,99	1,23	3,21	0,0044976	0,894103771				
rs114538020	0,43	0,21	0,86	0,04141279	0,894103771				
rs28535804	1,68	1,02	2,76	0,03923763	0,894103771	X	CSF2RA	HGNC Symbol	colony stimulating factor 2 receptor, alpha, low-affinity (granulocyte-macrophage) [Source:HGNC Symbol;Acc:2435]
rs28377023	1,88	1,14	3,11	0,01288862	0,894103771	X	CSF2RA	HGNC Symbol	colony stimulating factor 2 receptor, alpha, low-affinity (granulocyte-macrophage) [Source:HGNC Symbol;Acc:2435]
rs35544304	0,38	0,19	0,78	0,02432536	0,894103771	X	P2RY8	HGNC Symbol	purinergic receptor P2Y, G-protein coupled, 8 [Source:HGNC Symbol;Acc:15524]

SNP	OR	lower	upper	p-value	FDR	Chromosome	gene name	gene source	Description
rs28578016	2,63	1,45	4,77	0,00146535	0,894103771	X	P2RY8	HGNC Symbol	purinergic receptor P2Y, G-protein coupled, 8 [Source:HGNC Symbol;Acc:15524]
rs28391357	0,61	0,37	0,98	0,03626779	0,894103771	X	P2RY8	HGNC Symbol	purinergic receptor P2Y, G-protein coupled, 8 [Source:HGNC Symbol;Acc:15524]
rs5989833	0,56	0,31	1,01	0,04306654	0,894103771				
rs4639690	0,56	0,33	0,95	0,02656821	0,894103771	X	ASMT	HGNC Symbol	acetylserotonin O-methyltransferase [Source:HGNC Symbol;Acc:750]
rs5989869	0,94	0,45	1,95	0,0232664	0,894103771				
rs6644678	0,62	0,39	1	0,04368578	0,894103771	X	RP13-297E16.4	Clone-based (Vega)	
rs7054570	1,81	1,05	3,1	0,03384083	0,894103771				
rs73181537	1,72	0,93	3,17	0,02053117	0,894103771				
rs56048312	0,69	0,32	1,46	0,00601243	0,894103771	X	DHRSX	HGNC Symbol	dehydrogenase/reductase (SDR family) X-linked [Source:HGNC Symbol;Acc:18399]
rs7879755	1,84	1,06	3,2	0,03345085	0,894103771	X	DHRSX	HGNC Symbol	dehydrogenase/reductase (SDR family) X-linked [Source:HGNC Symbol;Acc:18399]
rs6642167	2,01	1,2	3,35	0,00786881	0,894103771	X	DHRSX	HGNC Symbol	dehydrogenase/reductase (SDR family) X-linked [Source:HGNC Symbol;Acc:18399]
rs6641784	2,88	1,07	7,78	0,04255325	0,894103771	X	DHRSX	HGNC Symbol	dehydrogenase/reductase (SDR family) X-linked [Source:HGNC Symbol;Acc:18399]
rs140302401	2,47	1,06	5,78	0,04184056	0,894103771	X	DHRSX;ZBED1	HGNC Symbol	dehydrogenase/reductase (SDR family) X-linked [Source:HGNC Symbol;Acc:18399];zinc finger, BED-type containing 1 [Source:HGNC Symbol;Acc:447]
rs5982733	3,52	1,08	11,49	0,04144192	0,894103771				

SNP	OR	lower	upper	p-value	FDR	Chromosome	gene name	gene source	Description
rs5939245	1,66	1,01	2,73	0,04597642	0,894103771	X	CD99P1	HGNC Symbol	CD99 molecule pseudogene 1 [Source:HGNC Symbol;Acc:7083]
rs6567632	0,52	0,27	0,98	0,031041	0,894103771	X	CD99P1	HGNC Symbol	CD99 molecule pseudogene 1 [Source:HGNC Symbol;Acc:7083]
rs184301	0,14	0,02	1,09	0,01305372	0,894103771	X	CD99	HGNC Symbol	CD99 molecule [Source:HGNC Symbol;Acc:7082]
rs112675992	0,28	0,06	1,22	0,04779993	0,894103771	X	CD99	HGNC Symbol	CD99 molecule [Source:HGNC Symbol;Acc:7082]
rs73188898	4,28	1,69	10,84	0,00253226	0,894103771				
rs2857316	0,37	0,17	0,8	0,0090016	0,894103771	X	XG	HGNC Symbol	Xg blood group [Source:HGNC Symbol;Acc:12806]

	SNP	OR	lower	upper	p-value	FDR	Chromosome	gene name	gene_source	description
Female (unadjusted)	rs11730582	0,54	0,34	0,86	0,00768722	0,130682711	4	SPP1	HGNC Symbol	Osteopontin; Secreted phosphoprotein 1; HGNC:11255
	rs1718119	0,59	0,35	0,98	0,03589786	0,205150804	12	P2RX7	HGNC Symbol	purinergic receptor P2X, ligand-gated ion channel, 7 [Source:HGNC Symbol;Acc:8537]
	rs8086340	0,62	0,4	0,98	0,03620308	0,205150804	18	TNFRSF11A	HGNC Symbol	tumor necrosis factor receptor superfamily, member 11a, NFKB activator [Source:HGNC Symbol;Acc:11908]
	rs1419931	0,48	0,25	0,94	0,0227369	1	X	XG	HGNC Symbol	Xg blood group [Source:HGNC Symbol;Acc:12806]
	rs4892892	0,59	0,36	0,99	0,04059256	1	X	XG	HGNC Symbol	Xg blood group [Source:HGNC Symbol;Acc:12806]
	rs311196	0,58	0,35	0,95	0,02385703	1	X	XG	HGNC Symbol	Xg blood group [Source:HGNC Symbol;Acc:12806]

SNP	OR	lower	upper	p-value	FDR	Chromosome	gene name	gene source	Description
rs12008127	0,42	0,19	0,93	0,01775394	1	X	GYG2	HGNC Symbol	glycogenin 2 [Source:HGNC Symbol;Acc:4700]
rs5939184	1,58	1,02	2,47	0,04103277	1	X	MXRA5	HGNC Symbol	matrix-remodelling associated 5 [Source:HGNC Symbol;Acc:7539]
rs56157309	1,8	1,11	2,92	0,01661601	1				
rs7889116	0,25	0,06	1,08	0,02617192	1				
rs140881654	0,27	0,06	1,19	0,04133343	1				
rs6567569	1,64	1,03	2,61	0,03894328	1	X	PRKX	HGNC Symbol	protein kinase, X-linked [Source:HGNC Symbol;Acc:9441]
rs73178179	2,43	1,05	5,63	0,04537612	1				
rs4826886	1,77	1,05	3	0,03362217	1				
rs113862114	0	0		0,04723886	1				
rs17219044	5,56	1,43	21,52	0,01501004	1				
rs144047820	2,37	1,09	5,15	0,03546395	1				
rs5962226	5,16	2,22	12,02	0,00021199	1				
rs144409421	2,4	1,15	5,02	0,01838783	1				
rs2218680	1,66	1,06	2,61	0,0267537	1				

SNP	OR	lower	upper	p-value	FDR	Chromosome	gene name	gene source	Description
rs115944192	2,06	1,04	4,08	0,02098387	1				
rs7065808	2,41	1,21	4,76	0,00951228	1				
rs1993794	0	0		0,02863285	1				
rs113732757	3,34	1,49	7,44	0,00601681	1				
rs144132782	0,26	0,08	0,85	0,04737554	1				
rs73436810	0,64	0,27	1,5	0,03141371	1				
rs12010896	0,68	0,29	1,59	0,04637966	1				
rs149156025	0,2	0,03	1,52	0,04916524	1				
rs5916158	0,59	0,37	0,96	0,02737094	1				
rs5916162	0,59	0,37	0,95	0,02508841	1				
rs17220204	2,27	1,23	4,2	0,01003305	1				
rs5916174	0,61	0,37	1	0,04096718	1				
rs5961325	3,05	1,17	7,92	0,02842224	1				
rs11094777	1,47	0,84	2,56	0,0491582	1				

SNP	OR	lower	upper	p-value	FDR	Chromosome	gene name	gene source	Description
rs5961751	1,49	0,87	2,57	0,0223769	1				
rs9698745	0,56	0,33	0,94	0,02092561	1				
rs12837275	4,58	1,53	13,74	0,00861367	1				
rs73186886	3,84	1,23	11,98	0,02617295	1				
rs55909210	3,09	1,26	7,56	0,00945937	1				
rs114443125	2,96	1,17	7,52	0,01579095	1				
rs55958419	1,73	1,03	2,91	0,04137658	1				
rs5980048	12,96	1,32	127,35	0,01587279	1				
rs12007994	1,75	0,89	3,44	0,04703971	1	X	KAL1	HGNC Symbol	Kallmann syndrome 1 sequence [Source:HGNC Symbol;Acc:6211]
rs2521584	1,17	0,57	2,39	0,03602596	1	X	TBL1X	HGNC Symbol	transducin (beta)-like 1X-linked [Source:HGNC Symbol;Acc:11585]
rs11795877	0,73	0,44	1,21	0,02159751	1	X	CLCN4	HGNC Symbol	chloride channel, voltage-sensitive 4 [Source:HGNC Symbol;Acc:2022]
rs5978399	2,6	1,18	5,7	0,02155539	1				
rs7054879	2,37	1,09	5,15	0,03546395	1				
rs960420	1,31	0,64	2,69	0,04813416	1	X	MID1	HGNC Symbol	midline 1 (Opitz/BBB syndrome) [Source:HGNC Symbol;Acc:7095]

SNP	OR	lower	upper	p-value	FDR	Chromosome	gene name	gene source	Description
rs974582	2,23	1,1	4,53	0,03120733	1	X	MID1	HGNC Symbol	midline 1 (Opitz/BBB syndrome) [Source:HGNC Symbol;Acc:7095]
rs5978438	0,54	0,31	0,93	0,02822041	1	X	ARHGAP6	HGNC Symbol	Rho GTPase activating protein 6 [Source:HGNC Symbol;Acc:676]
rs7350366	0,55	0,32	0,93	0,02016098	1	X	ARHGAP6	HGNC Symbol	Rho GTPase activating protein 6 [Source:HGNC Symbol;Acc:676]
rs6654942	0,54	0,31	0,93	0,02005806	1	X	ARHGAP6	HGNC Symbol	Rho GTPase activating protein 6 [Source:HGNC Symbol;Acc:676]
rs66948843	1,71	1,03	2,83	0,04284002	1	X	FRMPD4	HGNC Symbol	FERM and PDZ domain containing 4 [Source:HGNC Symbol;Acc:29007]
rs10521627	0,61	0,21	1,8	0,0420528	1	X	FRMPD4	HGNC Symbol	FERM and PDZ domain containing 4 [Source:HGNC Symbol;Acc:29007]
rs190831088	0,3	0,09	1,01	0,02521298	1	X	FRMPD4	HGNC Symbol	FERM and PDZ domain containing 4 [Source:HGNC Symbol;Acc:29007]
rs12557959	0,54	0,29	1,03	0,04825501	1	X	FRMPD4	HGNC Symbol	FERM and PDZ domain containing 4 [Source:HGNC Symbol;Acc:29007]
rs4639691	1,74	1,08	2,81	0,0235871	1	X	PRPS2	HGNC Symbol	phosphoribosyl pyrophosphate synthetase 2 [Source:HGNC Symbol;Acc:9465]
rs1266349	4,58	1,53	13,74	0,00861367	1	X	PRPS2	HGNC Symbol	phosphoribosyl pyrophosphate synthetase 2 [Source:HGNC Symbol;Acc:9465]
rs5979746	0,55	0,31	0,97	0,03012685	1				
rs5743740	2,58	1,11	6,01	0,03432421	1	X	TLR7	HGNC Symbol	toll-like receptor 7 [Source:HGNC Symbol;Acc:15631]
rs179009	0,54	0,29	1,01	0,04146717	1	X	TLR7	HGNC Symbol	toll-like receptor 7 [Source:HGNC Symbol;Acc:15631]
rs5979785	1,78	1,11	2,85	0,01672466	1				

SNP	OR	lower	upper	p-value	FDR	Chromosome	gene name	gene source	Description
rs5934071	0,6	0,35	1,02	0,04994728	1				
rs6526375	3,97	1,37	11,53	0,01483759	1				
rs1431726	0,41	0,18	0,93	0,01947582	1				
rs6527809	1,52	1	2,32	0,0481043	1				
rs73195762	0,28	0,07	1,13	0,00652148	1				
rs6654096	1,56	1	2,43	0,0470235	1	X	GPM6B	HGNC Symbol	glycoprotein M6B [Source:HGNC Symbol;Acc:4461]
rs66560303	1,75	1,11	2,76	0,01475854	1	X	GPM6B	HGNC Symbol	glycoprotein M6B [Source:HGNC Symbol;Acc:4461]
rs11798993	2,55	1,22	5,33	0,01615116	1				
rs1989844	1,72	1,13	2,63	0,01121004	1				
rs73189523	0,19	0,02	1,43	0,03946527	1	X	ASB9	HGNC Symbol	ankyrin repeat and SOCS box containing 9 [Source:HGNC Symbol;Acc:17184]
rs5935984	1,63	1,03	2,59	0,0381672	1	X	PIR;BMX	HGNC Symbol	pirin (iron-binding nuclear protein) [Source:HGNC Symbol;Acc:30048];BMX non-receptor tyrosine kinase [Source:HGNC Symbol;Acc:1079]
rs62578899	1,47	0,63	3,44	0,04910668	1	X	BMX	HGNC Symbol	BMX non-receptor tyrosine kinase [Source:HGNC Symbol;Acc:1079]
rs35803318	2,98	1,36	6,54	0,01075845	1	X	ACE2	HGNC Symbol	angiotensin I converting enzyme 2 [Source:HGNC Symbol;Acc:13557]
rs12006589	2,33	1,1	4,96	0,02479641	1				

SNP	OR	lower	upper	p-value	FDR	Chromosome	gene name	gene source	Description
rs62585413	0,19	0,02	1,46	0,0418357	1				
rs4831035	0,62	0,39	0,97	0,03316681	1				
rs73189179	2,78	1,09	7,09	0,04014789	1	X	RP3-410B11.1	Clone-based (Vega)	
rs145926052	2,3	1,06	5	0,03621787	1	X	SCML2	HGNC Symbol	sex comb on midleg-like 2 (Drosophila) [Source:HGNC Symbol;Acc:10581]
rs239746	0,38	0,17	0,85	0,00800565	1				
rs5990977	0,47	0,23	0,95	0,02318942	1				
rs73635509	0,51	0,26	1	0,03519691	1				
rs73197282	2,46	1,31	4,65	0,00651794	1				
rs35657111	0,18	0,02	1,35	0,03096199	1	X	PHEX	HGNC Symbol	phosphate regulating endopeptidase homolog, X-linked [Source:HGNC Symbol;Acc:8918]
rs73201130	5,56	1,43	21,52	0,01501004	1	X	PHEX	HGNC Symbol	phosphate regulating endopeptidase homolog, X-linked [Source:HGNC Symbol;Acc:8918]
rs2071585	0,57	0,32	1,01	0,04427681	1	X	PHEX	HGNC Symbol	phosphate regulating endopeptidase homolog, X-linked [Source:HGNC Symbol;Acc:8918]
rs6528091	0,54	0,3	0,97	0,03222233	1	X	PHEX	HGNC Symbol	phosphate regulating endopeptidase homolog, X-linked [Source:HGNC Symbol;Acc:8918]
rs2214521	0,54	0,29	0,99	0,03793709	1	X	RP11-40F8.2;GS1-433O24.1	Clone-based (Vega)	
rs12556950	0,62	0,4	0,98	0,03745275	1	X	RP11-40F8.2	Clone-based (Vega)	

SNP	OR	lower	upper	p-value	FDR	Chromosome	gene name	gene source	Description
rs5970898	1,7	1,05	2,74	0,0338417	1	X	RP11-40F8.2	Clone-based (Vega)	
rs66476440	0,54	0,29	1,02	0,04607594	1	X	RP11-40F8.2	Clone-based (Vega)	
rs2107306	1,57	1,01	2,44	0,04669588	1	X	RP11-40F8.2	Clone-based (Vega)	
rs5926152	2	1,19	3,36	0,01030758	1	X	RP11-40F8.2	Clone-based (Vega)	
rs56201502	1,71	1,02	2,87	0,04499982	1	X	RP11-40F8.2	Clone-based (Vega)	
rs5926231	2,33	1,1	4,96	0,03354393	1	X	RP11-40F8.2	Clone-based (Vega)	
rs2202958	2,76	1,29	5,89	0,01068837	1				
rs545426401	0,2	0,03	1,51	0,04827401	1				
rs151043588	0,15	0,02	1,16	0,01610366	1				
rs1972978	0,61	0,37	1,01	0,04775978	1	X	RP11-268G12.3	Clone-based (Vega)	
rs2197785	1,7	1,08	2,68	0,02281183	1	X	RP11-268G12.1	Clone-based (Vega)	
rs7055337	0,61	0,37	1,01	0,04775978	1	X	RP11-268G12.1	Clone-based (Vega)	
rs4534271	0,6	0,37	0,96	0,0279836	1				
rs6526672	0,33	0,14	0,8	0,00558721	1				

SNP	OR	lower	upper	p-value	FDR	Chromosome	gene name	gene source	Description
rs5926450	0,56	0,35	0,91	0,01480281	1				
rs221385	0,55	0,34	0,87	0,00931661	1				
rs5926847	0,56	0,35	0,9	0,01338786	1	X	DCAF8L2	HGNC Symbol	DDB1 and CUL4 associated factor 8-like 2 [Source:HGNC Symbol;Acc:31811]
rs5971429	0,47	0,29	0,77	0,00157266	1	X	DCAF8L2	HGNC Symbol	DDB1 and CUL4 associated factor 8-like 2 [Source:HGNC Symbol;Acc:31811]
rs3905591	0,45	0,28	0,74	0,00108998	1	X	DCAF8L2	HGNC Symbol	DDB1 and CUL4 associated factor 8-like 2 [Source:HGNC Symbol;Acc:31811]
rs5926888	1,8	1,14	2,85	0,01104956	1	X	DCAF8L2	HGNC Symbol	DDB1 and CUL4 associated factor 8-like 2 [Source:HGNC Symbol;Acc:31811]
rs5926892	0,43	0,24	0,77	0,00264278	1	X	DCAF8L2	HGNC Symbol	DDB1 and CUL4 associated factor 8-like 2 [Source:HGNC Symbol;Acc:31811]
rs5926895	0,52	0,31	0,85	0,00738181	1	X	DCAF8L2;AC 107613.1	HGNC Symbol;Cl one-based	DDB1 and CUL4 associated factor 8-like 2 [Source:HGNC Symbol;Acc:31811];
rs45553337	0,51	0,28	0,93	0,02103553	1	X	DCAF8L2;AC 107613.1	HGNC Symbol;Cl one-based	DDB1 and CUL4 associated factor 8-like 2 [Source:HGNC Symbol;Acc:31811];
rs7891169	1,59	1,01	2,51	0,04460123	1				
rs1368769	1,63	1,03	2,59	0,03694958	1	X	MAGEB10	HGNC Symbol	melanoma antigen family B, 10 [Source:HGNC Symbol;Acc:25377]
rs5926503	1,61	1,01	2,55	0,04238665	1				
rs5926917	2,99	1,32	6,75	0,01187695	1				
rs138124070	0,64	0,24	1,69	0,037462	1	X	IL1RAPL1	HGNC Symbol	interleukin 1 receptor accessory protein-like 1 [Source:HGNC Symbol;Acc:5996]

SNP	OR	lower	upper	p-value	FDR	Chromosome	gene name	gene source	Description
rs5927223	1,81	1,13	2,88	0,01226927	1	X	IL1RAPL1	HGNC Symbol	interleukin 1 receptor accessory protein-like 1 [Source:HGNC Symbol;Acc:5996]
rs73221614	0,36	0,16	0,81	0,00496558	1	X	IL1RAPL1	HGNC Symbol	interleukin 1 receptor accessory protein-like 1 [Source:HGNC Symbol;Acc:5996]
rs62589084	0	0		0,04730516	1				
rs12848838	0,91	0,55	1,5	0,02187637	1				
rs2864927	0,56	0,35	0,89	0,01153146	1				
rs73205886	3,2	1,41	7,27	0,00685498	1				
rs73205899	2,39	1,06	5,39	0,02676635	1				
rs12860337	1,64	1,01	2,68	0,04915409	1	X	TAB3	HGNC Symbol	TGF-beta activated kinase 1/MAP3K7 binding protein 3 [Source:HGNC Symbol;Acc:30681]
rs5972275	0,56	0,32	0,99	0,03622732	1	X	TAB3	HGNC Symbol	TGF-beta activated kinase 1/MAP3K7 binding protein 3 [Source:HGNC Symbol;Acc:30681]
rs2404151	0,47	0,24	0,9	0,01402879	1				
rs2606663	3,65	1,07	12,48	0,04802863	1	X	DMD	HGNC Symbol	dystrophin [Source:HGNC Symbol;Acc:2928]
rs2606665	0	0		0,03012712	1	X	DMD	HGNC Symbol	dystrophin [Source:HGNC Symbol;Acc:2928]
rs73221139	6,41	1,94	21,17	0,00251731	1	X	DMD	HGNC Symbol	dystrophin [Source:HGNC Symbol;Acc:2928]
rs16990375	0,5	0,24	1,03	0,04092314	1	X	DMD	HGNC Symbol	dystrophin [Source:HGNC Symbol;Acc:2928]

SNP	OR	lower	upper	p-value	FDR	Chromosome	gene name	gene source	Description
rs5972580	0	0		0,04730516	1	X	DMD	HGNC Symbol	dystrophin [Source:HGNC Symbol;Acc:2928]
rs5972586	0	0		0,04730516	1	X	DMD	HGNC Symbol	dystrophin [Source:HGNC Symbol;Acc:2928]
rs1158629	0,13	0,02	0,98	0,03299233	1	X	DMD	HGNC Symbol	dystrophin [Source:HGNC Symbol;Acc:2928]
rs73621844	3,67	1,07	12,55	0,04721099	1	X	DMD	HGNC Symbol	dystrophin [Source:HGNC Symbol;Acc:2928]
rs7879662	0,46	0,26	0,82	0,00507868	1	X	DMD	HGNC Symbol	dystrophin [Source:HGNC Symbol;Acc:2928]
rs5927113	2,26	1,17	4,36	0,02007701	1	X	DMD	HGNC Symbol	dystrophin [Source:HGNC Symbol;Acc:2928]
rs6631731	1,61	1,01	2,59	0,04853965	1	X	DMD	HGNC Symbol	dystrophin [Source:HGNC Symbol;Acc:2928]
rs12559939	0,27	0,1	0,68	0,01161941	1	X	DMD	HGNC Symbol	dystrophin [Source:HGNC Symbol;Acc:2928]
rs141927233	0,31	0,12	0,77	0,03194972	1	X	DMD	HGNC Symbol	dystrophin [Source:HGNC Symbol;Acc:2928]
rs73623943	0,47	0,24	0,92	0,01644011	1	X	DMD	HGNC Symbol	dystrophin [Source:HGNC Symbol;Acc:2928]
rs143580525	0,96	0,45	2,06	0,01452735	1				
rs146002754	1,18	0,55	2,55	0,02895122	1				
rs112512284	6,18	2,18	17,54	0,00076067	1	X	TMEM47	HGNC Symbol	transmembrane protein 47 [Source:HGNC Symbol;Acc:18515]
rs150433532	2,63	1,09	6,37	0,0399736	1				

SNP	OR	lower	upper	p-value	FDR	Chromosome	gene name	gene source	Description
rs12391979	3,03	1,12	8,2	0,03768402	1				
rs11798799	2,87	1,28	6,46	0,01190527	1	X	CXorf22	HGNC Symbol	chromosome X open reading frame 22 [Source:HGNC Symbol;Acc:28546]
rs28438823	0	0		0,04730516	1				
rs993441	1,93	1,14	3,29	0,01777273	1	X	TM4SF2;AL121578.2	UniProtKB Gene Name;Clo	Uncharacterized protein; cDNA FLJ59144, highly similar to Tetraspanin-7 [Source:UniProtKB/TrEMBL;Acc:B4E171];
rs3810691	0,56	0,3	1,03	0,04980566	1	X	RPGR;TM4SF2	HGNC Symbol;UniProtKB	retinitis pigmentosa GTPase regulator [Source:HGNC Symbol;Acc:10295];Uncharacterized protein; cDNA FLJ59144, highly similar to Tetraspanin-7
rs73192570	1,69	1,02	2,8	0,04155916	1	X	RPGR;TM4SF2	HGNC Symbol;UniProtKB	retinitis pigmentosa GTPase regulator [Source:HGNC Symbol;Acc:10295];Uncharacterized protein; cDNA FLJ59144, highly similar to Tetraspanin-7
rs6609582	1,62	1,04	2,51	0,03142096	1	X	TM4SF2	UniProtKB Gene Name	Uncharacterized protein; cDNA FLJ59144, highly similar to Tetraspanin-7 [Source:UniProtKB/TrEMBL;Acc:B4E171]
rs17246924	0,39	0,2	0,76	0,01569877	1	X	OTC;TM4SF2	HGNC Symbol;UniProtKB	ornithine carbamoyltransferase [Source:HGNC Symbol;Acc:8512];Uncharacterized protein; cDNA FLJ59144, highly similar to Tetraspanin-7
rs5963578	0,61	0,38	0,96	0,03042895	1				
rs6610244	0,92	0,55	1,54	0,04160854	1				
rs5963663	1,6	1,04	2,47	0,03106096	1				
rs6610294	1,69	1,02	2,8	0,04506378	1				
rs138436947	0,23	0,05	1	0,01766909	1				
rs7055988	1,94	1,06	3,54	0,03610593	1				

SNP	OR	lower	upper	p-value	FDR	Chromosome	gene name	gene source	Description
rs113395359	2,5	1,22	5,14	0,0149725	1				
rs35235436	1,91	1,07	3,41	0,03052341	1				
rs6610530	0,5	0,28	0,91	0,03778319	1				
rs6610538	0,4	0,21	0,77	0,01846312	1				
rs873336	1,78	1,02	3,12	0,04705215	1	X	NYX	HGNC Symbol	nyctalopin [Source:HGNC Symbol;Acc:8082]
rs5917441	2,62	1,21	5,69	0,01823363	1	X	CASK	HGNC Symbol	calcium/calmodulin-dependent serine protein kinase (MAGUK family) [Source:HGNC Symbol;Acc:1497]
rs5964067	0	0		0,04730516	1				
rs707440	0	0		0,02846115	1				
rs17146289	4,64	1,65	13,07	0,0048783	1	X	RP1-154K9.2	Clone-based (Vega)	
rs11266342	0,13	0,02	0,94	0,02665853	1				
rs12851446	5,33	1,83	15,51	0,00271787	1				
rs146896335	3,35	1,56	7,2	0,00274024	1				
rs147544363	0,2	0,03	1,52	0,04916524	1				
rs73212025	6,45	1,05	39,67	0,04542165	1				

SNP	OR	lower	upper	p-value	FDR	Chromosome	gene name	gene source	Description
rs142677545	3,03	1,17	7,88	0,02913761	1	X	MAOA	HGNC Symbol	monoamine oxidase A [Source:HGNC Symbol;Acc:6833]
rs3027450	1,77	1,09	2,88	0,02273698	1	X	MAOB	HGNC Symbol	monoamine oxidase B [Source:HGNC Symbol;Acc:6834]
rs140925586	3,68	1,37	9,87	0,01308186	1	X	EFHC2	HGNC Symbol	EF-hand domain (C-terminal) containing 2 [Source:HGNC Symbol;Acc:26233]
rs59597370	1,1	0,65	1,89	0,04803435	1				
rs6521042	0,45	0,27	0,74	0,00076708	1				
rs5906083	0,5	0,31	0,81	0,00338617	1				
rs2009184	0,47	0,29	0,76	0,00146056	1				
rs7058787	0,44	0,26	0,74	0,00092309	1				
rs5906093	0,61	0,39	0,95	0,02479877	1				
rs5952332	0,63	0,4	0,99	0,04131964	1				
rs28445915	1,72	1,09	2,73	0,0212394	1	X	SLC9A7	HGNC Symbol	solute carrier family 9, subfamily A (NHE7, cation proton antiporter 7), member 7 [Source:HGNC Symbol;Acc:17123]
rs1805147	3,36	1,21	9,36	0,03164349	1	X;HG29_PATCH	RP2	HGNC Symbol	retinitis pigmentosa 2 (X-linked recessive) [Source:HGNC Symbol;Acc:10274]
rs12560179	2,25	1,14	4,42	0,01109438	1	X	JADE3	HGNC Symbol	jade family PHD finger 3 [Source:HGNC Symbol;Acc:22982]
rs73201990	0,17	0,02	1,27	0,02418772	1				

SNP	OR	lower	upper	p-value	FDR	Chromosome	gene name	gene source	Description
rs73204096	6,45	1,05	39,67	0,04542165	1				
rs491610	0,19	0,02	1,43	0,03946527	1	X;HG1436_HG1432_PATCH	ZNF630;ZNF630-AS1	HGNC Symbol	zinc finger protein 630 [Source:HGNC Symbol;Acc:28855];ZNF630 antisense RNA 1 [Source:HGNC Symbol;Acc:41215]
rs4824755	1,61	1	2,59	0,04699093	1	X	GAGE10	HGNC Symbol	G antigen 10 [Source:HGNC Symbol;Acc:30968]
rs112296322	0,52	0,26	1,03	0,04481667	1				
rs12850774	1,9	1,09	3,31	0,02519094	1				
rs1110404	1,97	1,18	3,31	0,01029	1				
rs1936037	1,62	1,03	2,53	0,03561476	1				
rs12558898	1,78	1,13	2,81	0,01298714	1				
rs5951102	1,63	1,04	2,57	0,03598827	1				
rs150767800	1,99	1,18	3,35	0,01049181	1				
rs5991756	1,73	1,03	2,89	0,04067672	1				
rs141061448	1,57	0,89	2,76	0,02641736	1				
rs4129866	1,73	1,03	2,89	0,04067672	1				
rs145117130	1,68	1,01	2,81	0,04885102	1				

SNP	OR	lower	upper	p-value	FDR	Chromosome	gene name	gene source	Description
rs141219156	1,73	1,04	2,88	0,0357478	1				
rs12394834	1,73	1,04	2,88	0,0357478	1				
rs11795843	1,58	0,89	2,8	0,03862418	1				
rs17251419	4,4	1,22	15,84	0,02856373	1				
rs4462043	2,09	1,08	4,03	0,01498452	1				
rs17301297	2,07	1,03	4,19	0,04995456	1	X	ZC4H2	HGNC Symbol	zinc finger, C4H2 domain containing [Source:HGNC Symbol;Acc:24931]
rs62610373	3,49	1,23	9,89	0,02402584	1				
rs17249650	2,12	1,03	4,38	0,022921	1				
rs17216533	2,58	1,07	6,24	0,04364999	1				
rs111873599	2,04	1,05	3,99	0,04205596	1	X	HEPH	HGNC Symbol	hephaestin [Source:HGNC Symbol;Acc:4866]
rs112069404	1,95	1,09	3,48	0,02803551	1				
rs113989766	2,14	1,08	4,27	0,03577715	1				
rs62604342	2,92	1,07	7,99	0,04538545	1				
rs5965519	0,17	0,04	0,72	0,01335281	1	X	OPHN1	HGNC Symbol	oligophrenin 1 [Source:HGNC Symbol;Acc:8148]

SNP	OR	lower	upper	p-value	FDR	Chromosome	gene name	gene source	Description
rs41303733	1,77	0,87	3,6	0,04705121	1	X	OPHN1	HGNC Symbol	oligophrenin 1 [Source:HGNC Symbol;Acc:8148]
rs66998265	0	0		0,02863285	1				
rs877817	0,31	0,11	0,92	0,01672353	1	X	EFNB1	HGNC Symbol	ephrin-B1 [Source:HGNC Symbol;Acc:3226]
rs73528831	3,99	1,37	11,59	0,01449011	1				
rs4844335	0,49	0,27	0,9	0,01474924	1				
rs1317200	1,72	1,08	2,75	0,02189406	1				
rs147121494	1,9	1,03	3,49	0,04624451	1	X	EDA	HGNC Symbol	ectodysplasin A [Source:HGNC Symbol;Acc:3157]
rs35407838	1,96	1,14	3,36	0,01759546	1	X	EDA	HGNC Symbol	ectodysplasin A [Source:HGNC Symbol;Acc:3157]
rs5936806	1,64	1,05	2,57	0,03028735	1	X	EDA	HGNC Symbol	ectodysplasin A [Source:HGNC Symbol;Acc:3157]
rs73226452	0	0		0,04730516	1	X	EDA	HGNC Symbol	ectodysplasin A [Source:HGNC Symbol;Acc:3157]
rs6625563	0,61	0,37	0,98	0,0363376	1	X	EDA	HGNC Symbol	ectodysplasin A [Source:HGNC Symbol;Acc:3157]
rs111714615	2,35	1,26	4,36	0,00795842	1	X	DGAT2L6	HGNC Symbol	diacylglycerol O-acyltransferase 2-like 6 [Source:HGNC Symbol;Acc:23250]
rs145495213	3,71	1,08	12,71	0,04540361	1	X	KIF4A	HGNC Symbol	kinesin family member 4A [Source:HGNC Symbol;Acc:13339]
rs11796215	0	0		0,04723886	1	X	OGT	HGNC Symbol	O-linked N-acetylglucosamine (GlcNAc) transferase [Source:HGNC Symbol;Acc:8127]

SNP	OR	lower	upper	p-value	FDR	Chromosome	gene name	gene source	Description
rs149580659	3,24	1,23	8,54	0,02265344	1	X	ACRC	HGNC Symbol	acidic repeat containing [Source:HGNC Symbol;Acc:15805]
rs4986604	0,2	0,03	1,52	0,04916524	1				
rs138097931	2,09	1,19	3,67	0,01206161	1	X	NHSL2	HGNC Symbol	NHS-like 2 [Source:HGNC Symbol;Acc:33737]
rs59036635	0,17	0,02	1,28	0,0247382	1	X	NHSL2	HGNC Symbol	NHS-like 2 [Source:HGNC Symbol;Acc:33737]
rs62612130	2,92	1,21	7,05	0,01135964	1	X	NHSL2	HGNC Symbol	NHS-like 2 [Source:HGNC Symbol;Acc:33737]
rs148426364	6,45	1,05	39,67	0,04542165	1	X	NHSL2	HGNC Symbol	NHS-like 2 [Source:HGNC Symbol;Acc:33737]
rs3012627	1,71	0,93	3,15	0,03384219	1	X	PIN4;CITED1	HGNC Symbol	protein (peptidylprolyl cis/trans isomerase) NIMA-interacting, 4 (parvulin) [Source:HGNC Symbol;Acc:8992];Cbp/p300-interacting transactivator, with Glu/Asp-rich carboxy-terminal
rs62613008	2,85	1,45	5,59	0,00288749	1	X;HG1438_PATCH	PHKA1	HGNC Symbol	phosphorylase kinase, alpha 1 (muscle) [Source:HGNC Symbol;Acc:8925]
rs2428415	1,13	0,55	2,32	0,03716698	1				
rs62610693	1,96	1,04	3,71	0,0446201	1				
rs147312656	0,24	0,06	1,05	0,02251337	1				
rs62612360	3,67	1,07	12,55	0,04721099	1	X	KIAA2022	HGNC Symbol	KIAA2022 [Source:HGNC Symbol;Acc:29433]
rs41307373	0,56	0,24	1,32	0,01835942	1	X	UPRT	HGNC Symbol	uracil phosphoribosyltransferase (FUR1) homolog (S. cerevisiae) [Source:HGNC Symbol;Acc:28334]
rs113228883	0,57	0,24	1,33	0,02224917	1				

SNP	OR	lower	upper	p-value	FDR	Chromosome	gene name	gene source	Description
rs17303393	1,9	1,06	3,41	0,03370684	1	X;HG1426_PATCH	ATP7A	HGNC Symbol	ATPase, Cu++ transporting, alpha polypeptide [Source:HGNC Symbol;Acc:869]
rs12839556	1,69	1,07	2,68	0,02408207	1				
rs7064039	0,4	0,21	0,77	0,01806188	1				
rs12013328	1,68	1,01	2,8	0,04626746	1				
rs6622648	1,72	1,1	2,71	0,01701417	1				
rs73230915	2,37	1,18	4,76	0,00917286	1				
rs150558137	3,16	1,14	8,77	0,03454735	1				
rs707675	1,75	1,03	2,99	0,0439434	1				
rs112304795	2,42	1,05	5,6	0,04665846	1	X	KLHL4	HGNC Symbol	kelch-like family member 4 [Source:HGNC Symbol;Acc:6355]
rs7891751	2,75	1,17	6,45	0,02543556	1				
rs137863336	4,42	1,23	15,92	0,02805871	1				
rs73234137	3,15	1,28	7,71	0,01567794	1				
rs5942473	1,6	1,04	2,46	0,03400911	1				
rs142307315	0,62	0,38	0,99	0,04258463	1				

SNP	OR	lower	upper	p-value	FDR	Chromosome	gene name	gene source	Description
rs149838623	0,6	0,37	0,97	0,03330498	1				
rs73251336	2,04	1,13	3,7	0,0210462	1				
rs150957703	0,29	0,1	0,84	0,0457667	1				
rs73250402	2,61	1,3	5,23	0,00825601	1				
rs62594204	1,21	0,71	2,06	0,04895469	1				
rs72605109	1,08	0,66	1,78	0,00987971	1				
rs6618575	0,55	0,3	1,01	0,04321789	1				
rs12387371	0,58	0,35	0,95	0,02594395	1				
rs55674632	2,82	1,33	5,98	0,00569521	1				
rs145134624	2,58	1,23	5,42	0,01889237	1	X	PCDH11X	HGNC Symbol	protocadherin 11 X-linked [Source:HGNC Symbol;Acc:8656]
rs7891218	0,43	0,23	0,82	0,0343948	1	X	FAM133A	HGNC Symbol	family with sequence similarity 133, member A [Source:HGNC Symbol;Acc:26748]
rs1207446	0,56	0,31	1,02	0,04829856	1	X	RP1-60G11.1	Clone-based (Vega)	
rs73254374	0,47	0,27	0,83	0,00573913	1				
rs6619554	0,59	0,34	1,01	0,04306548	1				

SNP	OR	lower	upper	p-value	FDR	Chromosome	gene name	gene source	Description
rs185837010	0,14	0,02	1,08	0,01129703	1				
rs66864266	0,56	0,33	0,93	0,0187852	1				
rs73245118	2,52	1,07	5,93	0,03371643	1				
rs413169	1,54	1,01	2,34	0,04083953	1	X	DIAPH2	HGNC Symbol	diaphanous-related formin 2 [Source:HGNC Symbol;Acc:2877]
rs707287	0,63	0,4	0,98	0,03546887	1	X	DIAPH2	HGNC Symbol	diaphanous-related formin 2 [Source:HGNC Symbol;Acc:2877]
rs11796386	0,19	0,02	1,43	0,03946527	1	X	DIAPH2	HGNC Symbol	diaphanous-related formin 2 [Source:HGNC Symbol;Acc:2877]
rs12857090	0,64	0,4	1,01	0,04906097	1	X	DIAPH2	HGNC Symbol	diaphanous-related formin 2 [Source:HGNC Symbol;Acc:2877]
rs6620351	0,5	0,26	0,94	0,02150158	1				
rs6615988	0,58	0,34	0,97	0,03014362	1				
rs5921108	1,86	1,2	2,9	0,00553034	1				
rs6616015	1,72	1,1	2,7	0,01615083	1				
rs2473218	0,49	0,31	0,78	0,00205733	1				
rs16982961	0,3	0,11	0,78	0,0302988	1				
rs7889840	0,45	0,23	0,85	0,0080239	1				

SNP	OR	lower	upper	p-value	FDR	Chromosome	gene name	gene source	Description
rs4827953	0,42	0,25	0,72	0,00088919	1				
rs112879811	0,52	0,29	0,93	0,01969009	1				
rs16982964	2,78	1,09	7,14	0,0411892	1				
rs5921285	0,48	0,28	0,81	0,0036704	1				
rs5967145	1,62	1,01	2,59	0,04320396	1				
rs34429223	0,24	0,06	1,04	0,02127138	1				
rs1343213	2,52	1,34	4,75	0,00424091	1	X	SRPX2	HGNC Symbol	sushi-repeat containing protein, X-linked 2 [Source:HGNC Symbol;Acc:30668]
rs5966722	5,83	1,26	26,93	0,02630924	1				
rs60097212	4,63	1,8	11,91	0,00263893	1	X	ARL13A	HGNC Symbol	ADP-ribosylation factor-like 13A [Source:HGNC Symbol;Acc:31709]
rs17257516	0,15	0,02	1,1	0,01249	1	X	ARL13A	HGNC Symbol	ADP-ribosylation factor-like 13A [Source:HGNC Symbol;Acc:31709]
rs111917350	1,94	1,09	3,45	0,02829974	1	X	TRMT2B;TRMT2B-AS1	HGNC Symbol	tRNA methyltransferase 2 homolog B (S. cerevisiae) [Source:HGNC Symbol;Acc:25748];TRMT2B antisense RNA 1 [Source:HGNC Symbol;Acc:41116]
rs113487380	1,89	1,06	3,38	0,00158493	1	X	TRMT2B-AS1	HGNC Symbol	TRMT2B antisense RNA 1 [Source:HGNC Symbol;Acc:41116]
rs6621026	1,72	1,08	2,74	0,02140658	1	X	TAF7L	HGNC Symbol	TAF7-like RNA polymerase II, TATA box binding protein (TBP)-associated factor, 50kDa [Source:HGNC Symbol;Acc:11548]
rs2180271	0,37	0,21	0,65	0,00022113	1	X	TAF7L	HGNC Symbol	TAF7-like RNA polymerase II, TATA box binding protein (TBP)-associated factor, 50kDa [Source:HGNC Symbol;Acc:11548]

SNP	OR	lower	upper	p-value	FDR	Chromosome	gene name	gene source	Description
rs79990723	1,65	1,07	2,55	0,02514343	1				
rs2239460	0,3	0,09	1,01	0,02521298	1	X	BTK	HGNC Symbol	Bruton agammaglobulinemia tyrosine kinase [Source:HGNC Symbol;Acc:1133]
rs3027585	0	0		0,03012712	1	X;HG1439_PATCH	GLA;RPL36A-HNRNPH2	HGNC Symbol	galactosidase, alpha [Source:HGNC Symbol;Acc:4296];RPL36A-HNRNPH2 readthrough [Source:HGNC Symbol;Acc:48349]
rs5991904	0,31	0,12	0,79	0,03269602	1	X;HG1439_PATCH	ARMCX4	HGNC Symbol	armadillo repeat containing, X-linked 4 [Source:HGNC Symbol;Acc:28615]
rs61736018	0	0		0,02196775	1	X;HG1439_PATCH	ARMCX4	HGNC Symbol	armadillo repeat containing, X-linked 4 [Source:HGNC Symbol;Acc:28615]
rs6995	1,75	1,04	2,95	0,03832352	1	X;HG1439_PATCH	ARMCX3	HGNC Symbol	armadillo repeat containing, X-linked 3 [Source:HGNC Symbol;Acc:24065]
rs17284970	0	0		0,0168093	1	X	LINC00630; MTND1P32	HGNC Symbol	long intergenic non-protein coding RNA 630 [Source:HGNC Symbol;Acc:44263];MT-ND1 pseudogene 32 [Source:HGNC Symbol;Acc:42081]
rs17285025	0,2	0,03	1,52	0,04916524	1				
rs11545818	0,26	0,08	0,86	0,03374974	1	X	TCEAL4	HGNC Symbol	transcription elongation factor A (SII)-like 4 [Source:HGNC Symbol;Acc:26121]
rs521895	0,56	0,34	0,93	0,01829713	1	X	PLP1	HGNC Symbol	proteolipid protein 1 [Source:HGNC Symbol;Acc:9086]
rs5945830	1,6	1,02	2,53	0,04023786	1				
rs112217849	2,23	1,05	4,72	0,0476575	1	X	IL1RAPL2	HGNC Symbol	interleukin 1 receptor accessory protein-like 2 [Source:HGNC Symbol;Acc:5997]
rs73243896	6,45	1,05	39,67	0,04542165	1	X	IL1RAPL2	HGNC Symbol	interleukin 1 receptor accessory protein-like 2 [Source:HGNC Symbol;Acc:5997]
rs112962026	1,92	1,11	3,33	0,02242243	1	X	IL1RAPL2	HGNC Symbol	interleukin 1 receptor accessory protein-like 2 [Source:HGNC Symbol;Acc:5997]

SNP	OR	lower	upper	p-value	FDR	Chromosome	gene name	gene source	Description
rs17332218	0,16	0,02	1,24	0,02211863	1	X	IL1RAPL2	HGNC Symbol	interleukin 1 receptor accessory protein-like 2 [Source:HGNC Symbol;Acc:5997]
rs6616577	0,54	0,32	0,92	0,01688856	1	X;HG375_PATCH	IL1RAPL2	HGNC Symbol	interleukin 1 receptor accessory protein-like 2 [Source:HGNC Symbol;Acc:5997]
rs5962292	1,68	1,07	2,63	0,02260051	1	X	IL1RAPL2	HGNC Symbol	interleukin 1 receptor accessory protein-like 2 [Source:HGNC Symbol;Acc:5997]
rs5916932	1,65	1,05	2,59	0,02939185	1	X	IL1RAPL2	HGNC Symbol	interleukin 1 receptor accessory protein-like 2 [Source:HGNC Symbol;Acc:5997]
rs5962556	1,73	1,11	2,72	0,01560129	1	X	IL1RAPL2	HGNC Symbol	interleukin 1 receptor accessory protein-like 2 [Source:HGNC Symbol;Acc:5997]
rs5916936	1,69	1,07	2,66	0,02334312	1	X	IL1RAPL2	HGNC Symbol	interleukin 1 receptor accessory protein-like 2 [Source:HGNC Symbol;Acc:5997]
rs12557027	0,2	0,05	0,87	0,00781586	1	X	IL1RAPL2	HGNC Symbol	interleukin 1 receptor accessory protein-like 2 [Source:HGNC Symbol;Acc:5997]
rs11795816	0,19	0,05	0,84	0,00628445	1				
rs17254207	1,95	1,23	3,09	0,00434723	1	X	TSC22D3	HGNC Symbol	TSC22 domain family, member 3 [Source:HGNC Symbol;Acc:3051]
rs5917070	1,84	1,18	2,88	0,00670882	1	X	NCBP2L	HGNC Symbol	nuclear cap binding protein subunit 2-like [Source:HGNC Symbol;Acc:31795]
rs17254349	1,42	0,8	2,51	0,01062472	1	X	ATG4A	HGNC Symbol	autophagy related 4A, cysteine peptidase [Source:HGNC Symbol;Acc:16489]
rs112557865	1,25	0,71	2,22	0,01950673	1	X	COL4A6	HGNC Symbol	collagen, type IV, alpha 6 [Source:HGNC Symbol;Acc:2208]
rs5942970	1,64	0,93	2,89	0,01316771	1				
rs7061716	0,19	0,02	1,43	0,03868419	1				

SNP	OR	lower	upper	p-value	FDR	Chromosome	gene name	gene source	Description
rs5942780	0,2	0,03	1,51	0,04803667	1				
rs55663048	1,78	1,1	2,87	0,02073897	1				
rs17254838	1,93	1,15	3,26	0,01428727	1	X	AMMECR1	HGNC Symbol	Alport syndrome, mental retardation, midface hypoplasia and elliptocytosis chromosomal region gene 1 [Source:HGNC Symbol;Acc:467]
rs5942909	1,62	1,06	2,49	0,02762676	1	X	AMMECR1	HGNC Symbol	Alport syndrome, mental retardation, midface hypoplasia and elliptocytosis chromosomal region gene 1 [Source:HGNC Symbol;Acc:467]
rs73250295	0,19	0,02	1,46	0,0418357	1	X	AMMECR1	HGNC Symbol	Alport syndrome, mental retardation, midface hypoplasia and elliptocytosis chromosomal region gene 1 [Source:HGNC Symbol;Acc:467]
rs17882616	2,23	1,05	4,72	0,02807908	1	X	CAPN6	HGNC Symbol	calpain 6 [Source:HGNC Symbol;Acc:1483]
rs62613862	1,85	1,03	3,32	0,00654896	1				
rs5943223	1,76	0,99	3,13	0,01549499	1	X	TRPC5	HGNC Symbol	transient receptor potential cation channel, subfamily C, member 5 [Source:HGNC Symbol;Acc:12337]
rs17307746	0,17	0,02	1,27	0,02418772	1	X	TRPC5	HGNC Symbol	transient receptor potential cation channel, subfamily C, member 5 [Source:HGNC Symbol;Acc:12337]
rs5982532	0,39	0,18	0,86	0,04904021	1				
rs138049801	2,2	1,1	4,4	0,03048612	1				
rs3125955	0,48	0,29	0,81	0,00434411	1				
rs2206156	0,6	0,37	0,98	0,03598077	1				
rs142274380	2,18	1,06	4,51	0,04378376	1	X;HG1434_PATCH	RP5-964N17.1	Clone-based (Vega)	

SNP	OR	lower	upper	p-value	FDR	Chromosome	gene name	gene source	Description
rs6642665	0,88	0,54	1,46	0,02149722	1	X;HG1434_PATCH	RN7SL93P	HGNC Symbol	RNA, 7SL, cytoplasmic 93, pseudogene [Source:HGNC Symbol;Acc:46109]
rs73219446	12,96	1,32	127,35	0,01587279	1				
rs6655322	0,58	0,36	0,94	0,02122143	1				
rs72620919	0,87	0,39	1,94	0,00896863	1				
rs73222991	1,18	0,57	2,48	0,03553895	1				
rs6644208	1,09	0,51	2,34	0,03197956	1	X	LRCH2	HGNC Symbol	leucine-rich repeats and calponin homology (CH) domain containing 2 [Source:HGNC Symbol;Acc:29292]
rs11795689	1,09	0,51	2,34	0,03197956	1	X;HG1462_PATCH	LRCH2;RBMXL3	HGNC Symbol	leucine-rich repeats and calponin homology (CH) domain containing 2 [Source:HGNC Symbol;Acc:29292];RNA binding motif protein, X-linked-like 3 [Source:HGNC
rs7880226	0,52	0,16	1,77	0,02056787	1				
rs5987946	0,24	0,06	1,05	0,02251337	1	X;HG1462_PATCH	PLS3	HGNC Symbol	plastin 3 [Source:HGNC Symbol;Acc:9091]
rs5987956	0,29	0,12	0,74	0,00212009	1	X;HG1462_PATCH	PLS3	HGNC Symbol	plastin 3 [Source:HGNC Symbol;Acc:9091]
rs2108099	0,11	0,01	0,82	0,01842298	1	X;HG1462_PATCH	PLS3	HGNC Symbol	plastin 3 [Source:HGNC Symbol;Acc:9091]
rs12396000	0,1	0,01	0,75	0,00586664	1				
rs5987981	0,12	0,02	0,91	0,0204832	1				
rs9724449	1,66	1,09	2,54	0,01900604	1				

SNP	OR	lower	upper	p-value	FDR	Chromosome	gene name	gene source	Description
rs11091036	0,49	0,26	0,91	0,01674392	1				
rs5991107	0,57	0,35	0,93	0,01824819	1	X;HG1463_PATCH	RP11-761E20.1	Clone-based (Vega)	
rs142491398	3,06	1,35	6,93	0,01110314	1				
rs12847037	2	1,17	3,39	0,01157068	1				
rs73224882	2,18	1,03	4,61	0,04937967	1				
rs5950534	1,58	1,03	2,43	0,03513445	1				
rs148759028	0,61	0,23	1,63	0,02916508	1				
rs17231478	2,47	1,18	5,16	0,01528109	1	X	AGTR2	HGNC Symbol	angiotensin II receptor, type 2 [Source:HGNC Symbol;Acc:338]
rs111766629	2,28	1,14	4,57	0,00996435	1				
rs6608623	0,18	0,02	1,35	0,03144858	1				
rs113946157	2,41	1,16	5,04	0,01621661	1				
rs5910361	1,76	1,08	2,86	0,02304031	1				
rs6646819	0,41	0,19	0,88	0,01092301	1				
rs35251144	0,33	0,15	0,71	0,00157204	1				

SNP	OR	lower	upper	p-value	FDR	Chromosome	gene name	gene source	Description
rs73217404	6,45	1,05	39,67	0,04542165	1				
rs73636904	0	0		0,01690523	1				
rs6645774	0,63	0,4	1	0,04665172	1				
rs6645355	0,61	0,38	0,97	0,03266498	1				
rs17243490	3,65	1,07	12,48	0,04802863	1				
rs4825643	0,18	0,02	1,34	0,03030835	1				
rs2018358	0,53	0,3	0,94	0,02324008	1				
rs3848877	0,42	0,19	0,92	0,01526274	1	X	SEPT6	HGNC Symbol	septin 6 [Source:HGNC Symbol;Acc:15848]
rs2528711	0,61	0,38	0,97	0,03309748	1				
rs5910768	0,51	0,28	0,96	0,02537463	1	X;HG1442_PATCH	NKAPP1;RP4-755D9.1	HGNC Symbol;Clone-based	NFKB activating protein pseudogene 1 [Source:HGNC Symbol;Acc:26706];
rs5956194	0,59	0,36	0,99	0,03787169	1	X	NKAPP1	HGNC Symbol	NFKB activating protein pseudogene 1 [Source:HGNC Symbol;Acc:26706]
rs45453700	0,6	0,37	0,97	0,03215378	1	X;HG1442_PATCH	TMEM255A	HGNC Symbol	transmembrane protein 255A [Source:HGNC Symbol;Acc:26086]
rs5911060	4,26	1,63	11,14	0,00437724	1				
rs144911663	3,52	1,56	7,94	0,00340339	1				

SNP	OR	lower	upper	p-value	FDR	Chromosome	gene name	gene source	Description
rs149535880	2,2	1,1	4,4	0,01618538	1				
rs17258545	0	0		0,01046738	1				
rs151214516	2,2	1,08	4,48	0,02452741	1				
rs72609475	1,75	0,89	3,43	0,04677128	1				
rs1293473	0,46	0,25	0,85	0,03095766	1				
rs17259204	3,46	1,38	8,69	0,01132864	1				
rs4825832	0,5	0,28	0,9	0,01491037	1				
rs4145466	0,39	0,2	0,77	0,02303433	1				
rs11798709	0,63	0,39	1,01	0,04947415	1				
rs5911554	0,57	0,32	1	0,03964739	1	X	GRIA3	HGNC Symbol	glutamate receptor, ionotropic, AMPA 3 [Source:HGNC Symbol;Acc:4573]
rs2294414	0,62	0,38	1	0,04526477	1	X	TENM1;STAG2	HGNC Symbol	teneurin transmembrane protein 1 [Source:HGNC Symbol;Acc:8117];stromal antigen 2 [Source:HGNC Symbol;Acc:11355]
rs111472283	2,51	1,43	4,41	0,00153072	1	X	TENM1	HGNC Symbol	teneurin transmembrane protein 1 [Source:HGNC Symbol;Acc:8117]
rs5911935	2,01	1,07	3,77	0,03468429	1				
rs66831211	1,96	1,16	3,3	0,01337921	1				

SNP	OR	lower	upper	p-value	FDR	Chromosome	gene name	gene source	Description
rs56340315	3,84	1,23	11,98	0,02617295	1				
rs5933485	1,84	1,02	3,35	0,04598366	1				
rs732182	0,59	0,2	1,72	0,03435732	1	X	ACTRT1	HGNC Symbol	actin-related protein T1 [Source:HGNC Symbol;Acc:24027]
rs145339824	2,17	1,02	4,6	0,03081829	1				
rs1923842	2,47	1,28	4,78	0,0086422	1				
rs11795787	1,79	1,12	2,87	0,01648358	1	X	XPNPEP2	HGNC Symbol	X-prolyl aminopeptidase (aminopeptidase P) 2, membrane-bound [Source:HGNC Symbol;Acc:12823]
rs5903790	2,53	0,98	6,58	0,03538822	1				
rs2281278	0,59	0,24	1,46	0,02601754	1	X	UTP14A;RP4-537K23.4	HGNC Symbol;Clone-based	UTP14, U3 small nucleolar ribonucleoprotein, homolog A (yeast) [Source:HGNC Symbol;Acc:10665];
rs144741515	2,06	1,04	4,08	0,04519468	1	X	RBMX2	HGNC Symbol	RNA binding motif protein, X-linked 2 [Source:HGNC Symbol;Acc:24282]
rs17305432	0,15	0,02	1,16	0,01610366	1				
rs73233186	2,29	1,18	4,45	0,01745612	1	X	IGSF1	HGNC Symbol	immunoglobulin superfamily, member 1 [Source:HGNC Symbol;Acc:5948]
rs73235176	2,74	1,3	5,77	0,01044583	1				
rs4142509	1,76	1,11	2,81	0,01679776	1	X	MST4	UniProtKB Gene Name	Serine/threonine-protein kinase MST4 [Source:UniProtKB/Swiss-Prot;Acc:Q9P289]
rs5977623	1,71	1,09	2,71	0,02174369	1	X	FRMD7	HGNC Symbol	FERM domain containing 7 [Source:HGNC Symbol;Acc:8079]

SNP	OR	lower	upper	p-value	FDR	Chromosome	gene name	gene source	Description
rs5977658	3,18	1,3	7,79	0,01514625	1	X	RAP2C	HGNC Symbol	RAP2C, member of RAS oncogene family [Source:HGNC Symbol;Acc:21165]
rs6637988	4,06	1,48	11,15	0,00866529	1	X	RAP2C-AS1	HGNC Symbol	RAP2C antisense RNA 1 [Source:HGNC Symbol;Acc:40957]
rs144346774	0,19	0,02	1,46	0,0418357	1	X	HS6ST2	HGNC Symbol	heparan sulfate 6-O-sulfotransferase 2 [Source:HGNC Symbol;Acc:19133]
rs147826244	3,04	1,3	7,13	0,00681227	1	X	GPC3	HGNC Symbol	glypican 3 [Source:HGNC Symbol;Acc:4451]
rs138697029	3,65	1,07	12,48	0,04802863	1	X	AF003529.2	Clone-based (Vega)	
rs6638162	2,52	1,52	4,16	0,00023748	1				
rs62599383	0,62	0,38	1,01	0,04817646	1				
rs12850804	2,56	1,36	4,81	0,0052147	1				
rs73241337	0,15	0,02	1,1	0,01281681	1	X	PHF6	HGNC Symbol	PHD finger protein 6 [Source:HGNC Symbol;Acc:18145]
rs12558462	5,37	1,72	16,78	0,00475444	1				
rs41300299	2,86	1,05	7,79	0,04984649	1	X	PLAC1;RP11-308B5.2	HGNC Symbol;Clone-based	placenta-specific 1 [Source:HGNC Symbol;Acc:9044];
rs5975507	1,61	1,03	2,51	0,03326805	1				
rs139701979	3,51	1,39	8,88	0,01081404	1	X	RP11-432N13.4	Clone-based (Vega)	
rs140864304	2,59	1,15	5,85	0,02682737	1				

SNP	OR	lower	upper	p-value	FDR	Chromosome	gene name	gene source	Description
rs139890432	0,2	0,03	1,51	0,04824669	1	X	ARHGEF6	HGNC Symbol	Rac/Cdc42 guanine nucleotide exchange factor (GEF) 6 [Source:HGNC Symbol;Acc:685]
rs6633864	1,85	1,06	3,21	0,03259632	1				
rs10521782	1,8	1,1	2,96	0,02105464	1				
rs7886303	2	1,18	3,41	0,01116791	1				
rs1408308	1,59	1	2,54	0,04643441	1				
rs1366934	1,86	1,18	2,96	0,00703601	1				
rs34207960	0,17	0,02	1,28	0,02529575	1				
rs4829934	1,72	1,09	2,71	0,01859422	1				
rs6635599	1,72	1,1	2,68	0,01662378	1				
rs73236854	1,81	1,14	2,88	0,01129942	1				
rs1994451	0,6	0,38	0,93	0,0186893	1				
rs5931380	1,72	1,09	2,71	0,02008624	1				
rs5931431	0,52	0,12	2,22	0,01336768	1				
rs73241070	0,33	0,1	1,13	0,04439902	1	X	FGF13	HGNC Symbol	fibroblast growth factor 13 [Source:HGNC Symbol;Acc:3670]

SNP	OR	lower	upper	p-value	FDR	Chromosome	gene name	gene source	Description
rs73241079	0,15	0,02	1,1	0,01249	1	X	FGF13	HGNC Symbol	fibroblast growth factor 13 [Source:HGNC Symbol;Acc:3670]
rs141792138	0,18	0,02	1,34	0,03030835	1	X	FGF13	HGNC Symbol	fibroblast growth factor 13 [Source:HGNC Symbol;Acc:3670]
rs479265	1,85	1,03	3,32	0,04083248	1	X	FGF13	HGNC Symbol	fibroblast growth factor 13 [Source:HGNC Symbol;Acc:3670]
rs73243310	0,12	0,02	0,87	0,01626423	1	X	FGF13	HGNC Symbol	fibroblast growth factor 13 [Source:HGNC Symbol;Acc:3670]
rs12387343	0,35	0,17	0,73	0,01427996	1	X	FGF13	HGNC Symbol	fibroblast growth factor 13 [Source:HGNC Symbol;Acc:3670]
rs73245304	2,46	1,37	4,4	0,00283756	1				
rs6635942	1,76	1,06	2,92	0,03048786	1				
rs5955267	2,09	1,19	3,67	0,01078424	1				
rs4354481	2,22	1,27	3,91	0,00588212	1				
rs73585731	2,22	1,27	3,91	0,00588212	1				
rs2496271	1,66	1,02	2,71	0,0454397	1				
rs1541375	1,71	1,09	2,68	0,01817578	1				
rs6636011	1,77	1,14	2,74	0,00926826	1				
rs17322765	3,5	1,15	10,62	0,03455698	1				

SNP	OR	lower	upper	p-value	FDR	Chromosome	gene name	gene source	Description
rs67401583	0,63	0,39	1,01	0,04901868	1				
rs6528734	0,6	0,36	0,97	0,03085672	1				
rs11796840	5,38	1,96	14,77	0,00145421	1				
rs56239823	1,59	1,02	2,47	0,04441834	1	X	LINC00632	HGNC Symbol	long intergenic non-protein coding RNA 632 [Source:HGNC Symbol;Acc:27865]
rs41299075	0	0		0,04518843	1	X	CDR1	HGNC Symbol	cerebellar degeneration-related protein 1, 34kDa [Source:HGNC Symbol;Acc:1798]
rs6634214	2,2	1,1	4,4	0,02968836	1	X;HG1453_PATCH	RP11-298A8.2	Clone-based (Vega)	
rs5907910	1,6	1,03	2,5	0,03710471	1				
rs5907150	0,54	0,32	0,94	0,02079005	1				
rs6528868	1,97	1,17	3,32	0,01285489	1				
rs5907949	1,89	1,2	2,97	0,00571429	1				
rs7063314	2,11	1,34	3,31	0,00100508	1				
rs2239835	1,89	1,11	3,23	0,02058776	1	X	RP1-171K16.5;SPANXA2-OT1	Clone-based (Vega)	;SPANXA2 overlapping transcript 1 (non-protein coding) [Source:HGNC Symbol;Acc:31683]
rs6636421	1,78	1,04	3,03	0,03821117	1	X	SPANXA2-OT1	HGNC Symbol	SPANXA2 overlapping transcript 1 (non-protein coding) [Source:HGNC Symbol;Acc:31683]
rs6636440	1,89	1,13	3,16	0,01486461	1				

SNP	OR	lower	upper	p-value	FDR	Chromosome	gene name	gene source	Description
rs17257697	0,17	0,02	1,28	0,02529575	1				
rs5953652	2,2	1,15	4,21	0,02048839	1				
rs5954551	0	0		0,0282272	1				
rs12710619	2,16	1,3	3,61	0,00322704	1				
rs56254936	1,69	1,01	2,83	0,04905478	1				
rs62613621	0,61	0,38	1	0,04588256	1				
rs146573698	2,06	1,1	3,84	0,02661621	1				
rs5907416	2,4	1,04	5,57	0,04895245	1	X;HG1458_PATCH	GS1-256O22.5	Clone-based (Vega)	
rs138291813	0	0		0,02863285	1				
rs6649631	0,59	0,35	0,99	0,03786036	1				
rs12559795	0,16	0,02	1,2	0,01881448	1				
rs7049661	0,44	0,26	0,75	0,00096494	1				
rs11797855	0,52	0,3	0,9	0,01696011	1				
rs186982583	0,19	0,02	1,43	0,03868419	1				

SNP	OR	lower	upper	p-value	FDR	Chromosome	gene name	gene source	Description
rs6627094	5,56	1,43	21,52	0,01501004	1				
rs143116064	2,58	1,11	6,01	0,03432421	1				
rs1781502	0,55	0,32	0,95	0,02434149	1				
rs2748588	2,16	1,16	4,02	0,01811292	1	X;HG1459_PATCH	SLITRK2	HGNC Symbol	SLIT and NTRK-like family, member 2 [Source:HGNC Symbol;Acc:13449]
rs73237985	1,21	0,58	2,55	0,04531162	1				
rs73237986	0,27	0,06	1,17	0,03836182	1				
rs73237998	1,18	0,57	2,48	0,03553895	1				
rs5966154	0,63	0,3	1,29	0,02699004	1				
rs112561283	2,63	1,34	5,19	0,00635794	1				
rs78953490	2,91	1,28	6,66	0,01459375	1				
rs2891838	2,93	1,15	7,42	0,02808481	1				
rs6626213	0,61	0,37	1	0,0445116	1				
rs12839843	1,88	1,17	3,01	0,0094603	1				
rs5904936	1,56	1,02	2,39	0,04005957	1				

SNP	OR	lower	upper	p-value	FDR	Chromosome	gene name	gene source	Description
rs16994035	1,9	1,1	3,29	0,02377883	1				
rs2392722	2,05	1,25	3,37	0,00455175	1				
rs111830525	1,69	1,03	2,77	0,03909714	1				
rs2954104	0,58	0,35	0,96	0,03083741	1				
rs111900525	0,45	0,19	1,08	0,04787526	1				
rs45631658	0,53	0,28	0,99	0,03621729	1				
rs5904675	2,59	1,15	5,85	0,02682737	1				
rs2109881	0,63	0,4	0,98	0,0371311	1				
rs5936201	2,78	1,32	5,87	0,00925333	1				
rs1582301	0,56	0,36	0,88	0,01052411	1				
rs6641219	0,59	0,39	0,91	0,01525348	1				
rs1265414	0	0		0,04730516	1	X;HG1459_PATCH	AFF2	HGNC Symbol	AF4/FMR2 family, member 2 [Source:HGNC Symbol;Acc:3776]
rs148101966	2,58	1,07	6,24	0,04364999	1				
rs73244660	2,29	1,08	4,86	0,03571902	1				

SNP	OR	lower	upper	p-value	FDR	Chromosome	gene name	gene source	Description
rs56288522	2,64	1,3	5,35	0,00912954	1	X;HG1459_PATCH	LINC00894	HGNC Symbol	long intergenic non-protein coding RNA 894 [Source:HGNC Symbol;Acc:48579]
rs73246815	1,05	0,55	2,01	0,03322177	1				
rs17318777	1,07	0,41	2,77	0,00808676	1				
rs693913	1,87	1,2	2,91	0,00507315	1	X	MAMLD1	HGNC Symbol	mastermind-like domain containing 1 [Source:HGNC Symbol;Acc:2568]
rs1983610	0,63	0,41	0,97	0,03413046	1	X	MAMLD1	HGNC Symbol	mastermind-like domain containing 1 [Source:HGNC Symbol;Acc:2568]
rs12013750	1,85	1,05	3,27	0,03790332	1	X	MAMLD1	HGNC Symbol	mastermind-like domain containing 1 [Source:HGNC Symbol;Acc:2568]
rs12389317	1,66	1,02	2,69	0,04595821	1	X	MAMLD1	HGNC Symbol	mastermind-like domain containing 1 [Source:HGNC Symbol;Acc:2568]
rs5925221	1,55	1	2,39	0,04969836	1				
rs7065239	0,6	0,37	0,97	0,03414206	1				
rs12557844	1,63	1,03	2,58	0,03491363	1				
rs909084	0,1	0,01	0,72	0,00104361	1				
rs909086	0,2	0,07	0,59	0,00292585	1				
rs237397	0,54	0,33	0,87	0,0089306	1				
rs7057942	1,76	1,1	2,84	0,02048885	1				

SNP	OR	lower	upper	p-value	FDR	Chromosome	gene name	gene source	Description
rs5924858	1,63	1,01	2,63	0,0462143	1				
rs741726	2,1	1,31	3,35	0,0020786	1				
rs7054854	1,58	1,01	2,48	0,04305201	1				
rs5970118	1,84	1,18	2,86	0,00603191	1				
rs720378	0	0		0,04730516	1				
rs5925043	1,67	1,07	2,6	0,0219514	1				
rs149642212	2,54	1,29	4,99	0,00354635	1				
rs2051529	1,57	1	2,47	0,04690372	1				
rs145754054	0,15	0,02	1,16	0,01610366	1				
rs73241852	0,67	0,31	1,45	0,04426414	1				
rs5970181	0,51	0,27	0,99	0,03651562	1				
rs5925105	1,87	1,17	3	0,00749364	1				
rs5970194	0,45	0,22	0,93	0,02159043	1				
rs145701879	3,06	1,41	6,66	0,00644886	1	X	GABRA3;RP11-329E24.6	HGNC Symbol;Clone-based	gamma-aminobutyric acid (GABA) A receptor, alpha 3 [Source:HGNC Symbol;Acc:4077];

SNP	OR	lower	upper	p-value	FDR	Chromosome	gene name	gene source	Description
rs5970335	0,6	0,38	0,96	0,03028267	1				
rs5970440	1,77	1,13	2,77	0,01156306	1				
rs11094626	0,58	0,34	0,97	0,02967513	1				
rs3213466	0,57	0,34	0,95	0,02316233	1	X;HG1497_PATCH	ZNF275	HGNC Symbol	zinc finger protein 275 [Source:HGNC Symbol;Acc:13069]
rs62596388	0,55	0,33	0,92	0,01770326	1				
rs12400823	2,17	1,34	3,5	0,0015294	1				
rs2071028	0,22	0,05	0,93	0,01201206	1	X;HG1497_PATCH	SLC6A8	HGNC Symbol	solute carrier family 6 (neurotransmitter transporter), member 8 [Source:HGNC Symbol;Acc:11055]
rs149814383	2,83	0,99	8,06	0,02497529	1	X;HG1497_PATCH	L1CAM	HGNC Symbol	L1 cell adhesion molecule [Source:HGNC Symbol;Acc:6470]
rs3027924	2,36	1,17	4,73	0,01983979	1	X;HG1497_PATCH	MECP2	HGNC Symbol	methyl CpG binding protein 2 (Rett syndrome) [Source:HGNC Symbol;Acc:6990]
rs36051194	0,65	0,15	2,75	0,01974489	1	X;HG1497_PATCH	FLNA	HGNC Symbol	filamin A, alpha [Source:HGNC Symbol;Acc:3754]
rs4898495	0	0		0,0282272	1	X	ATP6AP1	HGNC Symbol	ATPase, H+ transporting, lysosomal accessory protein 1 [Source:HGNC Symbol;Acc:868]
rs113038688	2,47	1,03	5,89	0,04866326	1				
rs6642320	1,53	1	2,35	0,04949547	1	X	SPRY3	HGNC Symbol	sprouty homolog 3 (Drosophila) [Source:HGNC Symbol;Acc:11271]
rs28729587	2,82	1,48	5,4	0,00052727	1	X	SPRY3	HGNC Symbol	sprouty homolog 3 (Drosophila) [Source:HGNC Symbol;Acc:11271]

SNP	OR	lower	upper	p-value	FDR	Chromosome	gene name	gene source	Description
rs28425172	1,62	1,04	2,53	0,03240849	1				
rs34445725	0,55	0,31	0,99	0,03532305	1				
rs5940638	0,62	0,4	0,96	0,03032228	1				
rs3093493	2,13	1,17	3,86	0,01513336	1	X	IL9R	HGNC Symbol	interleukin 9 receptor [Source:HGNC Symbol;Acc:6030]
rs6649919	1,73	1,07	2,81	0,02669408	1				
rs28429757	0,3	0,11	0,76	0,02377936	1				
rs6645102	0,59	0,35	1,01	0,03314185	1	X	PPP2R3B	HGNC Symbol	protein phosphatase 2, regulatory subunit B'', beta [Source:HGNC Symbol;Acc:13417]
rs113150534	1,7	1,03	2,81	0,03923013	1				
rs5991299	0,58	0,36	0,92	0,01740821	1				
rs28404660	2,24	1,02	4,89	0,03275346	1				
rs28667393	0,64	0,41	1	0,04757171	1				
rs4472693	0,49	0,26	0,94	0,02182378	1				
rs2037897	0,62	0,38	1	0,0436264	1				
rs4911918	1,92	1,07	3,45	0,02998792	1				

SNP	OR	lower	upper	p-value	FDR	Chromosome	gene name	gene source	Description
rs2027987	0,62	0,39	0,99	0,03915568	1				
rs4911921	1,67	1,03	2,69	0,0364877	1				
rs28420575	0,67	0,27	1,67	0,03702215	1				
rs73178390	5,4	1,58	18,49	0,00867024	1				
rs5988437	0,49	0,29	0,83	0,00460032	1				
rs17148729	0,46	0,28	0,77	0,00158378	1				
rs5946570	1,71	1,09	2,68	0,01766501	1				
rs73182371	1,47	0,85	2,54	0,01750298	1				
rs73188058	1,62	1,03	2,57	0,0356367	1	X;HG480_HG481_PATCH	RP11-309M23.1	Clone-based (Vega)	
rs7878830	0,61	0,38	0,98	0,0376459	1				
rs73191918	2,08	0,99	4,38	0,03907917	1				
rs73191974	1,96	1,05	3,63	0,03836316	1				
rs66923305	0,59	0,38	0,93	0,01947855	1				
rs28377393	0,24	0,08	0,77	0,0283662	1				

SNP	OR	lower	upper	p-value	FDR	Chromosome	gene name	gene source	Description
rs34745620	1,66	1,07	2,59	0,02398019	1				
rs28460331	0,49	0,23	1,04	0,04524126	1				
rs73175748	1,64	1,05	2,57	0,03020476	1				
rs28404117	0,89	0,38	2,05	0,01030587	1	X	CSF2RA	HGNC Symbol	colony stimulating factor 2 receptor, alpha, low-affinity (granulocyte-macrophage) [Source:HGNC Symbol;Acc:2435]
rs73624872	2,27	1,23	4,18	0,00404934	1				
rs28540518	1,57	1,01	2,45	0,04514176	1				
rs113486847	2,14	1,27	3,6	0,00531576	1	X	LINC00106	HGNC Symbol	long intergenic non-protein coding RNA 106 [Source:HGNC Symbol;Acc:31843]
rs112670469	2,03	1,21	3,42	0,00911403	1	X	LINC00106	HGNC Symbol	long intergenic non-protein coding RNA 106 [Source:HGNC Symbol;Acc:31843]
rs113370738	2,19	1,24	3,88	0,00882306	1				
rs112133773	2,09	1,14	3,83	0,02073219	1	X	ASMTL	HGNC Symbol	acetylserotonin O-methyltransferase-like [Source:HGNC Symbol;Acc:751]
rs73182962	2,58	1,14	5,82	0,02767955	1				
rs28513563	0,61	0,37	1,01	0,04931381	1	X	AKAP17A	HGNC Symbol	A kinase (PRKA) anchor protein 17A [Source:HGNC Symbol;Acc:18783]
rs34397308	1,36	0,78	2,37	0,02821594	1	X	ASMT	HGNC Symbol	acetylserotonin O-methyltransferase [Source:HGNC Symbol;Acc:750]
rs6588807	0,73	0,44	1,2	0,01161794	1	X	ASMT	HGNC Symbol	acetylserotonin O-methyltransferase [Source:HGNC Symbol;Acc:750]

SNP	OR	lower	upper	p-value	FDR	Chromosome	gene name	gene source	Description
rs6588867	0,57	0,35	0,94	0,02240529	1				
rs5949181	1,77	1,12	2,8	0,01459918	1				
rs28405659	2,17	1,11	4,27	0,01396583	1				
rs28418610	1,91	1,05	3,47	0,03893786	1	X	DHRSX	HGNC Symbol	dehydrogenase/reductase (SDR family) X-linked [Source:HGNC Symbol;Acc:18399]
rs1044307	1,6	1,04	2,47	0,03100128	1	X	DHRSX;ZBED1	HGNC Symbol	dehydrogenase/reductase (SDR family) X-linked [Source:HGNC Symbol;Acc:18399];zinc finger, BED-type containing 1 [Source:HGNC Symbol;Acc:447]
rs951323	0,58	0,35	0,94	0,0253909	1				
rs35603448	0,64	0,41	1	0,04908738	1				
rs5939089	1,74	1,12	2,7	0,01255443	1				
rs6567636	0,52	0,3	0,9	0,01382922	1	X	CD99P1	HGNC Symbol	CD99 molecule pseudogene 1 [Source:HGNC Symbol;Acc:7083]
rs3813164	0,63	0,4	1,01	0,04943696	1				
rs311115	1,78	0,95	3,33	0,03601683	1	X	XG	HGNC Symbol	Xg blood group [Source:HGNC Symbol;Acc:12806]

SNP	OR	lower	upper	p-value	FDR	Chromosome	gene name	gene_source	description

	SNP	OR	lower	upper	p-value	FDR	Chromosome	gene name	gene source	Description
Male (adjusted)	rs5982611	0	0		0,02450869	0,89913013				
	rs2228431	0	0		0,04624257	0,89913013	X	ARSD	HGNC Symbol	arylsulfatase D [Source:HGNC Symbol;Acc:717]
	rs61978642	10,52	1,06	104,04	0,02694689	0,89913013	X	ARSH	HGNC Symbol	arylsulfatase family, member H [Source:HGNC Symbol;Acc:32488]
	rs112655645	10,52	1,06	104,04	0,02694689	0,89913013	X	ARSF	HGNC Symbol	arylsulfatase F [Source:HGNC Symbol;Acc:721]
	rs12009309	0,62	0,39	0,99	0,03093635	0,89913013				
	rs4892924	0,37	0,18	0,77	0,00068758	0,778251314				
	rs5982670	0,39	0,19	0,82	0,00150792	0,778251314				
	rs1194776	1,91	1,26	2,91	0,00290941	0,836207794				
	rs12559033	2,37	1,07	5,24	0,03762683	0,89913013	X	CXorf28	HGNC Symbol	chromosome X open reading frame 28 [Source:HGNC Symbol;Acc:27336]
	rs73175543	0	0		0,00140644	0,778251314				
	rs150932516	1,63	1,11	2,39	0,01402017	0,89913013				
	rs138239409	3,83	1,53	9,58	0,00485604	0,86719195	X	PRKX	HGNC Symbol	protein kinase, X-linked [Source:HGNC Symbol;Acc:9441]
	rs2287238	0,7	0,49	1	0,04461497	0,89913013	X	PRKX	HGNC Symbol	protein kinase, X-linked [Source:HGNC Symbol;Acc:9441]
	rs6641804	4,2	1,58	11,15	0,0046261	0,86719195	X	PRKX	HGNC Symbol	protein kinase, X-linked [Source:HGNC Symbol;Acc:9441]

SNP	OR	lower	upper	p-value	FDR	Chromosome	gene name	gene source	Description
rs12839071	0,62	0,44	0,88	0,00560504	0,86719195	X	PRKX	HGNC Symbol	protein kinase, X-linked [Source:HGNC Symbol;Acc:9441]
rs17335275	1,47	1,05	2,07	0,02785177	0,89913013	X	PRKX	HGNC Symbol	protein kinase, X-linked [Source:HGNC Symbol;Acc:9441]
rs17331167	2,92	1,37	6,24	0,00637973	0,86719195	X	PRKX	HGNC Symbol	protein kinase, X-linked [Source:HGNC Symbol;Acc:9441]
rs7880937	0,48	0,2	1,13	0,04398291	0,89913013	X	PRKX	HGNC Symbol	protein kinase, X-linked [Source:HGNC Symbol;Acc:9441]
rs6641847	1,72	1,13	2,61	0,0124825	0,89913013	X	PRKX	HGNC Symbol	protein kinase, X-linked [Source:HGNC Symbol;Acc:9441]
rs5916456	0,41	0,14	1,16	0,03196162	0,89913013				
rs3876184	0,4	0,19	0,84	0,01185003	0,89913013				
rs111602303	3,46	1,5	7,99	0,00438528	0,86719195				
rs17313406	2,51	1,21	5,18	0,01462106	0,89913013				
rs6649581	0,68	0,48	0,97	0,02739038	0,89913013				
rs28465437	1,46	1,04	2,03	0,02555361	0,89913013				
rs62575757	1,43	1,02	1,99	0,03589061	0,89913013				
rs5916684	0,61	0,43	0,86	0,00376466	0,86719195				
rs5915430	1,58	1,13	2,21	0,0065935	0,86719195	X	RP11-706O15.1	Clone-based (Vega)	HCG1981372, isoform CRA_c; Uncharacterized protein [Source:UniProtKB/TrEMBL;Acc:B1B108]

SNP	OR	lower	upper	p-value	FDR	Chromosome	gene name	gene source	Description
rs113371969	0	0		0,02994532	0,89913013				
rs113862114	0	0		0,02994532	0,89913013				
rs6640754	1,57	1,09	2,24	0,01546699	0,89913013				
rs73180343	2,57	1,33	5	0,00456457	0,86719195				
rs5915780	0,63	0,41	0,98	0,02911202	0,89913013				
rs6638512	0,33	0,16	0,67	0,00150979	0,778251314				
rs5961309	2,27	1,12	4,59	0,02534636	0,89913013				
rs73451282	3,08	1,05	9,03	0,04646913	0,89913013				
rs62588650	6,34	1,11	36,17	0,03148812	0,89913013				
rs62590383	0	0		0,01247407	0,89913013				
rs55909210	4,74	1,54	14,57	0,00689588	0,86719195				
rs114443125	4,74	1,54	14,57	0,00689588	0,86719195				
rs4830729	0,68	0,46	1,01	0,04702559	0,89913013				
rs6639942	0,45	0,19	1,03	0,04734682	0,89913013				

SNP	OR	lower	upper	p-value	FDR	Chromosome	gene name	gene source	Description
rs5933639	2,05	1,02	4,09	0,04473729	0,89913013				
rs73194488	0	0		0,03020564	0,89913013				
rs12008804	0,31	0,09	1,07	0,03607432	0,89913013	X	KAL1	HGNC Symbol	Kallmann syndrome 1 sequence [Source:HGNC Symbol;Acc:6211]
rs1232116	1,46	1,04	2,04	0,02692981	0,89913013				
rs10521598	2,41	1,15	5,03	0,02157176	0,89913013				
rs1395790	0,4	0,16	1,02	0,03844288	0,89913013				
rs5934523	1,45	1,03	2,04	0,02909182	0,89913013				
rs62581812	0	0		0,00088968	0,778251314	X	FAM9B	HGNC Symbol	family with sequence similarity 9, member B [Source:HGNC Symbol;Acc:18404]
rs1466984	0,69	0,47	1,01	0,04953437	0,89913013	X	FAM9B	HGNC Symbol	family with sequence similarity 9, member B [Source:HGNC Symbol;Acc:18404]
rs35912577	0	0		0,00425134	0,86719195	X	FAM9B	HGNC Symbol	family with sequence similarity 9, member B [Source:HGNC Symbol;Acc:18404]
rs73186314	0,61	0,41	0,91	0,01090082	0,89913013				
rs5934617	0,27	0,08	0,93	0,01736882	0,89913013				
rs2040616	4,06	1,02	16,13	0,04896082	0,89913013	X	TBL1X	HGNC Symbol	transducin (beta)-like 1X-linked [Source:HGNC Symbol;Acc:11585]
rs146927570	1,89	1,14	3,14	0,01664192	0,89913013	X	SHROOM2	HGNC Symbol	shroom family member 2 [Source:HGNC Symbol;Acc:630]

SNP	OR	lower	upper	p-value	FDR	Chromosome	gene name	gene source	Description
rs79628572	0	0		0,0093616	0,89913013	X	SHROOM2	HGNC Symbol	shroom family member 2 [Source:HGNC Symbol;Acc:630]
rs5979272	0,63	0,42	0,94	0,01785079	0,89913013	X	CLCN4	HGNC Symbol	chloride channel, voltage-sensitive 4 [Source:HGNC Symbol;Acc:2022]
rs11795877	0,6	0,37	0,98	0,02595814	0,89913013	X	CLCN4	HGNC Symbol	chloride channel, voltage-sensitive 4 [Source:HGNC Symbol;Acc:2022]
rs5934812	1,99	1,02	3,86	0,04059373	0,89913013	X	CLCN4	HGNC Symbol	chloride channel, voltage-sensitive 4 [Source:HGNC Symbol;Acc:2022]
rs1012782	0,5	0,3	0,85	0,00330254	0,853796107	X	RP6-1O2.1	Clone-based (Vega)	
rs17327648	0,6	0,38	0,94	0,01646322	0,89913013	X	RP6-1O2.1	Clone-based (Vega)	
rs5979305	0,44	0,21	0,94	0,02652644	0,89913013				
rs2525070	0,39	0,16	0,94	0,02384285	0,89913013	X	MID1	HGNC Symbol	midline 1 (Opitz/BBB syndrome) [Source:HGNC Symbol;Acc:7095]
rs7878745	0,26	0,06	1,17	0,04044873	0,89913013	X	MID1	HGNC Symbol	midline 1 (Opitz/BBB syndrome) [Source:HGNC Symbol;Acc:7095]
rs974581	0	0		0,02899791	0,89913013	X	MID1	HGNC Symbol	midline 1 (Opitz/BBB syndrome) [Source:HGNC Symbol;Acc:7095]
rs184989752	4,77	1,02	22,31	0,04899603	0,89913013	X	MID1	HGNC Symbol	midline 1 (Opitz/BBB syndrome) [Source:HGNC Symbol;Acc:7095]
rs6530417	0,25	0,06	1,11	0,03163122	0,89913013	X	RP11-120D5.1	Clone-based (Vega)	
rs5933977	0,59	0,39	0,87	0,00498175	0,86719195	X	FRMPD4	HGNC Symbol	FERM and PDZ domain containing 4 [Source:HGNC Symbol;Acc:29007]
rs5933996	0,71	0,51	1	0,045821	0,89913013	X	FRMPD4	HGNC Symbol	FERM and PDZ domain containing 4 [Source:HGNC Symbol;Acc:29007]

SNP	OR	lower	upper	p-value	FDR	Chromosome	gene name	gene source	Description
rs55761104	2,06	1,14	3,75	0,01921089	0,89913013	X	FRMPD4	HGNC Symbol	FERM and PDZ domain containing 4 [Source:HGNC Symbol;Acc:29007]
rs5978532	0,18	0,02	1,37	0,034951	0,89913013	X	FRMPD4	HGNC Symbol	FERM and PDZ domain containing 4 [Source:HGNC Symbol;Acc:29007]
rs6419058	1,51	1,07	2,14	0,0219043	0,89913013	X	FRMPD4	HGNC Symbol	FERM and PDZ domain containing 4 [Source:HGNC Symbol;Acc:29007]
rs149324165	5,1	1,44	18,11	0,01294241	0,89913013	X	FRMPD4	HGNC Symbol	FERM and PDZ domain containing 4 [Source:HGNC Symbol;Acc:29007]
rs12838746	0	0		0,03749837	0,89913013	X	FRMPD4	HGNC Symbol	FERM and PDZ domain containing 4 [Source:HGNC Symbol;Acc:29007]
rs5935361	1,53	1,09	2,16	0,01446424	0,89913013	X	FRMPD4	HGNC Symbol	FERM and PDZ domain containing 4 [Source:HGNC Symbol;Acc:29007]
rs5978611	4,89	1,05	22,82	0,04531298	0,89913013				
rs12851640	0,63	0,41	0,97	0,0238193	0,89913013				
rs5979841	0,47	0,23	0,94	0,02920422	0,89913013				
rs6631468	0,46	0,21	0,98	0,03616833	0,89913013				
rs17256262	7,19	1,71	30,21	0,00534576	0,86719195	X	GS1-600G8.5	Clone-based (Vega)	
rs4830840	1,49	1,06	2,11	0,02430319	0,89913013	X	GS1-600G8.5	Clone-based (Vega)	
rs113670844	0	0		0,01098344	0,89913013	X	GS1-600G8.3	Clone-based (Vega)	
rs5934088	0	0		0,00395559	0,86719195				

SNP	OR	lower	upper	p-value	FDR	Chromosome	gene name	gene source	Description
rs73192267	3,51	1,07	11,51	0,04332408	0,89913013	X	RP11-142G7.2	Clone-based (Vega)	
rs12844566	1,45	1,04	2,02	0,02959166	0,89913013				
rs11095629	0,66	0,44	0,99	0,03492145	0,89913013	X	GPM6B	HGNC Symbol	glycoprotein M6B [Source:HGNC Symbol;Acc:4461]
rs7052395	0,65	0,42	1	0,03721631	0,89913013	X	GPM6B	HGNC Symbol	glycoprotein M6B [Source:HGNC Symbol;Acc:4461]
rs73189523	0	0		0,02148298	0,89913013	X	ASB9	HGNC Symbol	ankyrin repeat and SOCS box containing 9 [Source:HGNC Symbol;Acc:17184]
rs1139868	0,35	0,12	1,04	0,03563218	0,89913013	X	ASB9	HGNC Symbol	ankyrin repeat and SOCS box containing 9 [Source:HGNC Symbol;Acc:17184]
rs731197	0,35	0,12	1,04	0,03563218	0,89913013	X	ASB9	HGNC Symbol	ankyrin repeat and SOCS box containing 9 [Source:HGNC Symbol;Acc:17184]
rs35859007	0	0		0,03569563	0,89913013	X	ASB11	HGNC Symbol	ankyrin repeat and SOCS box containing 11 [Source:HGNC Symbol;Acc:17186]
rs5935944	1,52	1,08	2,12	0,01493564	0,89913013	X	ASB11	HGNC Symbol	ankyrin repeat and SOCS box containing 11 [Source:HGNC Symbol;Acc:17186]
rs2071178	1,46	1,04	2,03	0,02570549	0,89913013	X	PIR	HGNC Symbol	pirin (iron-binding nuclear protein) [Source:HGNC Symbol;Acc:30048]
rs8094	1,54	1,1	2,16	0,01065082	0,89913013	X	PIR	HGNC Symbol	pirin (iron-binding nuclear protein) [Source:HGNC Symbol;Acc:30048]
rs5935984	1,41	1,01	1,98	0,04699839	0,89913013	X	PIR;BMX	HGNC Symbol	pirin (iron-binding nuclear protein) [Source:HGNC Symbol;Acc:30048];BMX non-receptor tyrosine kinase [Source:HGNC Symbol;Acc:1079]
rs35803318	0	0		0,01808579	0,89913013	X	ACE2	HGNC Symbol	angiotensin I converting enzyme 2 [Source:HGNC Symbol;Acc:13557]
rs62578920	0,19	0,02	1,47	0,04651374	0,89913013	X	CA5BP1	HGNC Symbol	carbonic anhydrase VB pseudogene 1 [Source:HGNC Symbol;Acc:29544]

SNP	OR	lower	upper	p-value	FDR	Chromosome	gene name	gene source	Description
rs1557807	0	0		0,02003685	0,89913013	X	ZRSR2	HGNC Symbol	zinc finger (CCCH type), RNA-binding motif and serine/arginine rich 2 [Source:HGNC Symbol;Acc:23019]
rs11798628	0,55	0,29	1,03	0,03256461	0,89913013				
rs4355975	0,47	0,26	0,88	0,00568653	0,86719195				
rs140173662	1,81	1,02	3,19	0,04756315	0,89913013	X	CTPS2	HGNC Symbol	CTP synthase 2 [Source:HGNC Symbol;Acc:2520]
rs4528028	1,86	1,05	3,3	0,03763789	0,89913013				
rs4828523	1,57	1,05	2,37	0,03326525	0,89913013	X	REPS2	HGNC Symbol	RALBP1 associated Eps domain containing 2 [Source:HGNC Symbol;Acc:9963]
rs1365528	1,53	1,02	2,29	0,04378103	0,89913013	X	REPS2	HGNC Symbol	RALBP1 associated Eps domain containing 2 [Source:HGNC Symbol;Acc:9963]
rs12396700	1,58	1,05	2,37	0,03156602	0,89913013	X	REPS2	HGNC Symbol	RALBP1 associated Eps domain containing 2 [Source:HGNC Symbol;Acc:9963]
rs2382815	0,15	0,02	1,19	0,01930704	0,89913013				
rs7878986	0,17	0,02	1,35	0,03266834	0,89913013				
rs5950862	1,61	1,06	2,47	0,03084137	0,89913013				
rs73189153	0	0		0,03408304	0,89913013	X	NHS;RP1-60N8.1	HGNC Symbol;Cl one-based	Nance-Horan syndrome (congenital cataracts and dental anomalies) [Source:HGNC Symbol;Acc:7820];
rs150066059	6,01	1,99	18,16	0,00139017	0,778251314	X	RAI2	HGNC Symbol	retinoic acid induced 2 [Source:HGNC Symbol;Acc:9835]
rs139411880	5,17	1,67	16,01	0,00453877	0,86719195				

SNP	OR	lower	upper	p-value	FDR	Chromosome	gene name	gene source	Description
rs73191522	0	0		0,02149978	0,89913013	X	CDKL5	HGNC Symbol	cyclin-dependent kinase-like 5 [Source:HGNC Symbol;Acc:11411]
rs5955504	2,12	1,1	4,11	0,02450337	0,89913013	X	RS1	HGNC Symbol	retinoschisin 1 [Source:HGNC Symbol;Acc:10457]
rs7065106	0	0		0,01545296	0,89913013				
rs7890403	0	0		0,04023913	0,89913013				
rs5955548	0	0		0,02122711	0,89913013	X	PDHA1	HGNC Symbol	pyruvate dehydrogenase (lipoamide) alpha 1 [Source:HGNC Symbol;Acc:8806]
rs7881721	0,46	0,22	0,95	0,030681	0,89913013				
rs5990881	0,16	0,02	1,21	0,02152561	0,89913013				
rs73450881	0	0		0,03951369	0,89913013				
rs57666057	0	0		0,03951369	0,89913013				
rs5990821	5,63	1,86	17	0,00208441	0,792635449				
rs5990929	3,68	1,36	9,98	0,01224579	0,89913013				
rs1900730	3,85	1,41	10,56	0,01005439	0,89913013				
rs1531597	0	0		0,04835937	0,89913013				
rs73197282	0	0		0,01575585	0,89913013				

SNP	OR	lower	upper	p-value	FDR	Chromosome	gene name	gene source	Description
rs5951620	0	0		0,03539454	0,89913013				
rs10284169	1,45	1,03	2,03	0,03126476	0,89913013				
rs3213451	1,41	1,01	1,98	0,04568083	0,89913013	X	MBTPS2	HGNC Symbol	membrane-bound transcription factor peptidase, site 2 [Source:HGNC Symbol;Acc:15455]
rs5951640	0	0		0,04719041	0,89913013	X	MBTPS2	HGNC Symbol	membrane-bound transcription factor peptidase, site 2 [Source:HGNC Symbol;Acc:15455]
rs2361662	1,41	1,01	1,97	0,04455518	0,89913013				
rs5951709	2,61	1,34	5,09	0,00462431	0,86719195	X	PHEX	HGNC Symbol	phosphate regulating endopeptidase homolog, X-linked [Source:HGNC Symbol;Acc:8918]
rs6633525	1,54	1,1	2,15	0,01035036	0,89913013	X	PHEX	HGNC Symbol	phosphate regulating endopeptidase homolog, X-linked [Source:HGNC Symbol;Acc:8918]
rs12396173	10,56	2,04	54,54	0,00204796	0,792635449	X	PHEX	HGNC Symbol	phosphate regulating endopeptidase homolog, X-linked [Source:HGNC Symbol;Acc:8918]
rs112585845	2,77	1,08	7,08	0,03876489	0,89913013	X	PHEX	HGNC Symbol	phosphate regulating endopeptidase homolog, X-linked [Source:HGNC Symbol;Acc:8918]
rs56196258	0,67	0,46	0,97	0,02877779	0,89913013	X	RP11-40F8.2	Clone-based (Vega)	
rs7057115	2,55	1,15	5,68	0,0246717	0,89913013	X	RP11-494I9.2;RP11-40F8.2	Clone-based (Vega)	
rs67802966	0,25	0,09	0,69	0,0023676	0,808955961	X	RP11-494I9.2;RP11-40F8.2	Clone-based (Vega)	
rs12399184	0,46	0,22	0,95	0,03053465	0,89913013	X	RP11-494I9.2;RP11-40F8.2	Clone-based (Vega)	
rs16982185	0,19	0,02	1,5	0,04933252	0,89913013	X	RP11-40F8.2	Clone-based (Vega)	

SNP	OR	lower	upper	p-value	FDR	Chromosome	gene name	gene source	Description
rs112505478	0	0		0,00569459	0,86719195	X	RP11-40F8.2	Clone-based (Vega)	
rs61463999	0	0		0,00025086	0,57740729	X	RP11-40F8.2	Clone-based (Vega)	
rs5925668	0,63	0,42	0,94	0,01689697	0,89913013	X	RP11-40F8.2	Clone-based (Vega)	
rs34934530	0,68	0,48	0,96	0,02482288	0,89913013	X	RP11-40F8.2	Clone-based (Vega)	
rs66476440	1,5	1,04	2,18	0,03401059	0,89913013	X	RP11-40F8.2	Clone-based (Vega)	
rs11094865	0,39	0,18	0,8	0,00812367	0,897527099	X	RP11-40F8.2	Clone-based (Vega)	
rs111326022	0	0		0,04484045	0,89913013	X	RP11-40F8.2	Clone-based (Vega)	
rs12690411	0	0		0,01297397	0,89913013	X	RP11-40F8.2	Clone-based (Vega)	
rs142914756	0,32	0,13	0,81	0,00862637	0,89913013	X	RP11-40F8.2	Clone-based (Vega)	
rs67994587	1,54	1,06	2,23	0,02719604	0,89913013	X	RP11-40F8.2	Clone-based (Vega)	
rs67040061	1,57	1,07	2,3	0,02345633	0,89913013	X	RP11-40F8.2	Clone-based (Vega)	
rs5970700	0	0		0,02903538	0,89913013	X	RP11-40F8.2	Clone-based (Vega)	
rs5926265	0,34	0,16	0,75	0,00418619	0,86719195	X	RP11-40F8.2	Clone-based (Vega)	
rs6629602	4,58	1,16	18,18	0,03226447	0,89913013	X	RP11-40F8.2	Clone-based (Vega)	

SNP	OR	lower	upper	p-value	FDR	Chromosome	gene name	gene source	Description
rs578606	0,29	0,07	1,25	0,01581741	0,89913013				
rs2665363	0,31	0,07	1,35	0,027946	0,89913013				
rs34092038	4,52	1,3	15,67	0,0191636	0,89913013	X	RP13-314C10.5	Clone-based (Vega)	
rs73197327	5,73	1,31	25,07	0,01875192	0,89913013	X	KLHL15	HGNC Symbol	kelch-like family member 15 [Source:HGNC Symbol;Acc:29347]
rs5970824	1,58	1,11	2,25	0,01122295	0,89913013	X	KLHL15	HGNC Symbol	kelch-like family member 15 [Source:HGNC Symbol;Acc:29347]
rs73203501	4,27	1,35	13,49	0,01486176	0,89913013				
rs111323038	6,17	1,63	23,33	0,00720542	0,86719195				
rs113045766	6,16	1,89	20,03	0,00227153	0,808955961				
rs146194002	10,55	2,04	54,48	0,00206531	0,792635449				
rs12689847	2,2	1,18	4,1	0,01306567	0,89913013	X	PCYT1B	HGNC Symbol	phosphate cytidylyltransferase 1, choline, beta [Source:HGNC Symbol;Acc:8755]
rs111855794	0	0		0,02821899	0,89913013	X	PCYT1B	HGNC Symbol	phosphate cytidylyltransferase 1, choline, beta [Source:HGNC Symbol;Acc:8755]
rs11573406	8,08	1,4	46,53	0,0156838	0,89913013	X	POLA1	HGNC Symbol	polymerase (DNA directed), alpha 1, catalytic subunit [Source:HGNC Symbol;Acc:9173]
rs73209032	7,09	1,69	29,86	0,00576773	0,86719195				
rs959644	1,56	1,11	2,19	0,00910211	0,89913013				

SNP	OR	lower	upper	p-value	FDR	Chromosome	gene name	gene source	Description
rs73195057	0,67	0,47	0,95	0,02098828	0,89913013				
rs5986536	12,49	1,24	125,93	0,01875133	0,89913013				
rs67066022	0,18	0,02	1,36	0,03386409	0,89913013				
rs140120359	0	0		0,00134247	0,778251314				
rs148361973	4,59	1,16	18,11	0,03152536	0,89913013				
rs141815299	0,16	0,02	1,24	0,02300468	0,89913013				
rs73204844	8,73	1,62	46,96	0,00709014	0,86719195				
rs73204855	0	0		0,02157191	0,89913013				
rs1234509	0,52	0,27	1,04	0,03204666	0,89913013				
rs4893551	1,56	1,1	2,21	0,01339054	0,89913013				
rs7876256	1,57	1,02	2,4	0,04432549	0,89913013				
rs1384575	0	0		0,00571374	0,86719195	X	IL1RAPL1	HGNC Symbol	interleukin 1 receptor accessory protein-like 1 [Source:HGNC Symbol;Acc:5996]
rs5943585	0	0		0,01502245	0,89913013	X	IL1RAPL1	HGNC Symbol	interleukin 1 receptor accessory protein-like 1 [Source:HGNC Symbol;Acc:5996]
rs147730436	0	0		0,04231101	0,89913013	X	IL1RAPL1	HGNC Symbol	interleukin 1 receptor accessory protein-like 1 [Source:HGNC Symbol;Acc:5996]

SNP	OR	lower	upper	p-value	FDR	Chromosome	gene name	gene source	Description
rs6628511	0,43	0,19	0,96	0,02945631	0,89913013				
rs5929084	0,61	0,41	0,9	0,00806052	0,897527099				
rs60676446	0,46	0,25	0,85	0,00352929	0,86719195				
rs17282991	0,59	0,39	0,88	0,00635537	0,86719195				
rs5927624	0,39	0,14	1,06	0,04542915	0,89913013				
rs6631208	0	0		0,0348337	0,89913013	X	TAB3	HGNC Symbol	TGF-beta activated kinase 1/MAP3K7 binding protein 3 [Source:HGNC Symbol;Acc:30681]
rs12860337	0,42	0,18	1,02	0,04039133	0,89913013	X	TAB3	HGNC Symbol	TGF-beta activated kinase 1/MAP3K7 binding protein 3 [Source:HGNC Symbol;Acc:30681]
rs7067182	0,12	0,02	0,91	0,00527098	0,86719195	X	TAB3	HGNC Symbol	TGF-beta activated kinase 1/MAP3K7 binding protein 3 [Source:HGNC Symbol;Acc:30681]
rs113429490	0,39	0,14	1,1	0,02015872	0,89913013				
rs62588738	1,51	1,08	2,12	0,01770318	0,89913013				
rs12012680	0,42	0,15	1,2	0,0401398	0,89913013				
rs5927716	0,35	0,11	1,07	0,04240309	0,89913013	X	DMD	HGNC Symbol	dystrophin [Source:HGNC Symbol;Acc:2928]
rs2646305	1,99	1,02	3,9	0,04605533	0,89913013	X	DMD	HGNC Symbol	dystrophin [Source:HGNC Symbol;Acc:2928]
rs112663088	0	0		0,0294481	0,89913013	X	DMD	HGNC Symbol	dystrophin [Source:HGNC Symbol;Acc:2928]

SNP	OR	lower	upper	p-value	FDR	Chromosome	gene name	gene source	Description
rs5972472	4,07	1,04	15,97	0,04595432	0,89913013	X	DMD	HGNC Symbol	dystrophin [Source:HGNC Symbol;Acc:2928]
rs11095222	2,96	1,2	7,34	0,022205	0,89913013	X	DMD	HGNC Symbol	dystrophin [Source:HGNC Symbol;Acc:2928]
rs145272492	0	0		0,00138738	0,778251314	X	DMD	HGNC Symbol	dystrophin [Source:HGNC Symbol;Acc:2928]
rs7058785	0	0		0,00299463	0,836207794	X	DMD	HGNC Symbol	dystrophin [Source:HGNC Symbol;Acc:2928]
rs55686834	0,19	0,02	1,51	0,04984209	0,89913013	X	DMD	HGNC Symbol	dystrophin [Source:HGNC Symbol;Acc:2928]
rs111826558	3,66	1,67	8,03	0,0014041	0,778251314	X	DMD	HGNC Symbol	dystrophin [Source:HGNC Symbol;Acc:2928]
rs966451	0	0		0,02399705	0,89913013	X	DMD	HGNC Symbol	dystrophin [Source:HGNC Symbol;Acc:2928]
rs1456727	2,38	1,13	5,02	0,02588357	0,89913013	X	DMD	HGNC Symbol	dystrophin [Source:HGNC Symbol;Acc:2928]
rs1456729	2,67	1,25	5,69	0,01260594	0,89913013	X	DMD	HGNC Symbol	dystrophin [Source:HGNC Symbol;Acc:2928]
rs1408983	2,91	1,28	6,62	0,01275132	0,89913013	X	DMD	HGNC Symbol	dystrophin [Source:HGNC Symbol;Acc:2928]
rs331317	0,65	0,46	0,92	0,01242492	0,89913013	X	DMD	HGNC Symbol	dystrophin [Source:HGNC Symbol;Acc:2928]
rs142762831	0,42	0,15	1,2	0,04212623	0,89913013	X	DMD	HGNC Symbol	dystrophin [Source:HGNC Symbol;Acc:2928]
rs5972699	2,14	1,09	4,18	0,02453795	0,89913013	X	DMD	HGNC Symbol	dystrophin [Source:HGNC Symbol;Acc:2928]
rs9887516	0,45	0,21	0,99	0,03829932	0,89913013	X	DMD	HGNC Symbol	dystrophin [Source:HGNC Symbol;Acc:2928]

SNP	OR	lower	upper	p-value	FDR	Chromosome	gene name	gene source	Description
rs12395380	0,55	0,29	1,03	0,03262253	0,89913013	X	DMD	HGNC Symbol	dystrophin [Source:HGNC Symbol;Acc:2928]
rs17338877	0,33	0,12	0,91	0,00311689	0,836207794	X	DMD	HGNC Symbol	dystrophin [Source:HGNC Symbol;Acc:2928]
rs67636309	0,29	0,08	1,01	0,02639269	0,89913013	X	DMD	HGNC Symbol	dystrophin [Source:HGNC Symbol;Acc:2928]
rs35874041	0	0		0,04828332	0,89913013	X	DMD	HGNC Symbol	dystrophin [Source:HGNC Symbol;Acc:2928]
rs150494081	0	0		0,01169231	0,89913013	X	DMD	HGNC Symbol	dystrophin [Source:HGNC Symbol;Acc:2928]
rs3006092	0,32	0,09	1,11	0,04385449	0,89913013				
rs138401839	0	0		0,03977945	0,89913013	X	RP11-545D19.1	Clone-based (Vega)	
rs5973200	0,62	0,39	0,98	0,02864496	0,89913013				
rs11095347	1,55	1,04	2,31	0,03498781	0,89913013				
rs146419966	0	0		0,01331885	0,89913013	X	RP11-87M18.2	Clone-based (Vega)	
rs35184915	1,74	1,05	2,88	0,03629286	0,89913013	X;HG1424_PATCH	TM4SF2	UniProtKB Gene Name	Uncharacterized protein; cDNA FLJ59144, highly similar to Tetraspanin-7 [Source:UniProtKB/TrEMBL;Acc:B4E171]
rs34097325	1,96	1,02	3,75	0,04973141	0,89913013	X;HG1424_PATCH	CYBB;TM4SF2	HGNC Symbol;UniProtKB	cytochrome b-245, beta polypeptide [Source:HGNC Symbol;Acc:2578];Uncharacterized protein; cDNA FLJ59144, highly similar to Tetraspanin-7
rs112906393	3,07	1,17	8,04	0,02596215	0,89913013	X	RP11-265P11.1	Clone-based (Vega)	
rs138436947	0	0		0,0072576	0,86719195				

SNP	OR	lower	upper	p-value	FDR	Chromosome	gene name	gene source	Description
rs584465	0,47	0,24	0,93	0,02652174	0,89913013				
rs150222	0,46	0,24	0,92	0,025103	0,89913013				
rs4614139	0,5	0,26	0,99	0,04431361	0,89913013				
rs2919834	0	0		0,04839984	0,89913013				
rs114454893	4,31	1,1	16,98	0,03868033	0,89913013				
rs140911585	5,27	1,4	19,77	0,0135273	0,89913013	X	MIR1587	HGNC Symbol	microRNA 1587 [Source:HGNC Symbol;Acc:41596]
rs17314146	0,36	0,12	1,1	0,04938837	0,89913013				
rs5963756	0,32	0,09	1,13	0,0459396	0,89913013				
rs62584872	0	0		0,0118116	0,89913013				
rs140319336	2,08	1,17	3,7	0,01456643	0,89913013				
rs35865200	1,5	1,03	2,17	0,03680053	0,89913013				
rs1150527	6,82	1,53	30,35	0,01073348	0,89913013	X	USP9X	HGNC Symbol	ubiquitin specific peptidase 9, X-linked [Source:HGNC Symbol;Acc:12632]
rs6610530	2,53	1,22	5,26	0,01439909	0,89913013				
rs150255888	22,23	2,48	199,29	0,00080614	0,778251314				

SNP	OR	lower	upper	p-value	FDR	Chromosome	gene name	gene source	Description
rs5918155	1,62	1,15	2,27	0,00507265	0,86719195				
rs5918284	0,69	0,47	1	0,04453366	0,89913013				
rs3921116	1,4	1,01	1,96	0,04457265	0,89913013				
rs12353585	2,52	1,11	5,72	0,0316996	0,89913013				
rs5950942	1,46	1,05	2,04	0,02350651	0,89913013				
rs5905418	0	0		0,04089701	0,89913013	X	MAOA	HGNC Symbol	monoamine oxidase A [Source:HGNC Symbol;Acc:6833]
rs150326947	8,01	1,38	46,56	0,01654546	0,89913013				
rs55779415	2,13	1,11	4,07	0,02521052	0,89913013				
rs149975561	0	0		0,0177735	0,89913013	X	EFHC2	HGNC Symbol	EF-hand domain (C-terminal) containing 2 [Source:HGNC Symbol;Acc:26233]
rs6610918	0,71	0,5	1	0,04595795	0,89913013				
rs6610953	0,34	0,11	1,02	0,03275761	0,89913013	X	FUNDC1	HGNC Symbol	FUN14 domain containing 1 [Source:HGNC Symbol;Acc:28746]
rs59597370	0,64	0,41	1,02	0,04509009	0,89913013				
rs142255983	0	0		0,0162354	0,89913013				
rs12849327	1,55	1,05	2,29	0,03283961	0,89913013				

SNP	OR	lower	upper	p-value	FDR	Chromosome	gene name	gene source	Description
rs4335267	1,46	1,05	2,05	0,02452494	0,89913013	X	CXorf36;RP11-342D14.1	HGNC Symbol;Clone-based	chromosome X open reading frame 36 [Source:HGNC Symbol;Acc:25866];
rs10854928	0,51	0,26	1	0,04829626	0,89913013				
rs41312098	10,28	1,02	103,99	0,03038951	0,89913013	X	RP11-245M24.1	Clone-based (Vega)	
rs5952318	2,18	1,12	4,26	0,02219004	0,89913013	X	RP11-245M24.1	Clone-based (Vega)	
rs1536278	2,1	1,07	4,1	0,02986206	0,89913013	X	RP11-245M24.1	Clone-based (Vega)	
rs35857391	2,19	1,1	4,34	0,02503165	0,89913013				
rs149674363	0	0		0,03067775	0,89913013	X	KRBOX4	HGNC Symbol	KRAB box domain containing 4 [Source:HGNC Symbol;Acc:26007]
rs148555208	0	0		0,01651359	0,89913013				
rs4567179	4,76	1,43	15,84	0,00208114	0,792635449	X	JADE3	HGNC Symbol	jade family PHD finger 3 [Source:HGNC Symbol;Acc:22982]
rs12852223	0,65	0,45	0,95	0,02149854	0,89913013	X;HG29_PATCH	RBM10	HGNC Symbol	RNA binding motif protein 10 [Source:HGNC Symbol;Acc:9896]
rs7063875	1,45	1,04	2,03	0,02755594	0,89913013				
rs1142636	2,11	1,07	4,16	0,0322319	0,89913013	X	SYN1	HGNC Symbol	synapsin I [Source:HGNC Symbol;Acc:11494]
rs4824624	2,11	1,06	4,19	0,03404414	0,89913013	X	SYN1	HGNC Symbol	synapsin I [Source:HGNC Symbol;Acc:11494]
rs1998837	1,42	1,01	2,01	0,04587149	0,89913013	X	ELK1	HGNC Symbol	ELK1, member of ETS oncogene family [Source:HGNC Symbol;Acc:3321]

SNP	OR	lower	upper	p-value	FDR	Chromosome	gene name	gene source	Description
rs77265236	3,57	0,99	12,8	0,0329299	0,89913013				
rs41305753	10,42	1,03	105,55	0,02942477	0,89913013	X;HG1436_HG1432_PATCH	GAGE1	HGNC Symbol	G antigen 1 [Source:HGNC Symbol;Acc:4098]
rs4824497	5,19	1,1	24,47	0,03925059	0,89913013				
rs5915296	0,35	0,13	0,95	0,02420662	0,89913013	X;HG1433_PATCH	CCNB3	HGNC Symbol	cyclin B3 [Source:HGNC Symbol;Acc:18709]
rs73202119	0	0		0,00720315	0,86719195				
rs62597512	0	0		0,04563801	0,89913013				
rs62597211	0	0		0,04563801	0,89913013				
rs17316052	0	0		0,03102943	0,89913013	X;HG1433_PATCH	IQSEC2	HGNC Symbol	IQ motif and Sec7 domain 2 [Source:HGNC Symbol;Acc:29059]
rs141138279	0	0		0,01081338	0,89913013				
rs45468097	0	0		0,04899147	0,89913013	X	ALAS2	HGNC Symbol	aminolevulinate, delta-, synthase 2 [Source:HGNC Symbol;Acc:397]
rs10521479	0,55	0,31	0,99	0,02384449	0,89913013				
rs2375465	4,78	1,01	22,55	0,04953323	0,89913013	X	FOXR2	HGNC Symbol	forkhead box R2 [Source:HGNC Symbol;Acc:30469]
rs149700928	0,68	0,46	1,01	0,04760627	0,89913013				
rs6521388	0,67	0,45	0,99	0,03495854	0,89913013				

SNP	OR	lower	upper	p-value	FDR	Chromosome	gene name	gene source	Description
rs73224076	0,18	0,02	1,43	0,040816	0,89913013	X	FAAH2	HGNC Symbol	fatty acid amide hydrolase 2 [Source:HGNC Symbol;Acc:26440]
rs73226027	0	0		0,03877066	0,89913013				
rs73226044	0	0		0,03877066	0,89913013				
rs73209413	0	0		0,01988631	0,89913013				
rs2942863	0	0		0,03877066	0,89913013				
rs144255958	0	0		0,03980955	0,89913013				
rs73211275	0	0		0,00722757	0,86719195				
rs62610407	0	0		0,04184631	0,89913013				
rs1152311	0	0		0,03976671	0,89913013				
rs7056244	0	0		0,03099586	0,89913013				
rs149483793	0	0		0,04938076	0,89913013				
rs142169094	1,54	1,05	2,25	0,03002263	0,89913013				
rs62604342	5,67	1,19	27,03	0,03119965	0,89913013				
rs5919427	0,42	0,17	1,03	0,04409626	0,89913013				

SNP	OR	lower	upper	p-value	FDR	Chromosome	gene name	gene source	Description
rs61098894	0,41	0,16	1,06	0,04939597	0,89913013				
rs146376239	0,35	0,14	0,85	0,01258235	0,89913013				
rs4562482	0,45	0,22	0,94	0,02896746	0,89913013				
rs62604532	5,19	1,1	24,5	0,03919375	0,89913013				
rs2765951	0,33	0,15	0,72	0,00299664	0,836207794				
rs7888212	0,32	0,14	0,7	0,00238949	0,808955961	X	OPHN1	HGNC Symbol	oligophrenin 1 [Source:HGNC Symbol;Acc:8148]
rs143483183	4,24	1,22	14,7	0,02443885	0,89913013	X	OPHN1	HGNC Symbol	oligophrenin 1 [Source:HGNC Symbol;Acc:8148]
rs5965497	0,41	0,2	0,83	0,01037631	0,89913013	X	OPHN1	HGNC Symbol	oligophrenin 1 [Source:HGNC Symbol;Acc:8148]
rs73212857	0,58	0,38	0,88	0,00564495	0,86719195	X	OPHN1	HGNC Symbol	oligophrenin 1 [Source:HGNC Symbol;Acc:8148]
rs12836444	0,41	0,2	0,86	0,01395497	0,89913013	X	OPHN1	HGNC Symbol	oligophrenin 1 [Source:HGNC Symbol;Acc:8148]
rs111365012	0,63	0,41	0,97	0,02477032	0,89913013	X	OPHN1	HGNC Symbol	oligophrenin 1 [Source:HGNC Symbol;Acc:8148]
rs150294060	1,75	1,07	2,88	0,03113274	0,89913013	X	OPHN1	HGNC Symbol	oligophrenin 1 [Source:HGNC Symbol;Acc:8148]
rs5918825	0,36	0,12	1,09	0,04736234	0,89913013	X	OPHN1	HGNC Symbol	oligophrenin 1 [Source:HGNC Symbol;Acc:8148]
rs5965561	0,3	0,09	1,03	0,02933506	0,89913013	X	OPHN1	HGNC Symbol	oligophrenin 1 [Source:HGNC Symbol;Acc:8148]

SNP	OR	lower	upper	p-value	FDR	Chromosome	gene name	gene source	Description
rs5919571	0	0		0,02890373	0,89913013				
rs705896	3,7	1,84	7,45	0,00014101	0,501260262				
rs241396	2,22	1,13	4,33	0,01952511	0,89913013				
rs17302682	0,69	0,49	0,96	0,02705436	0,89913013				
rs4844341	0,71	0,5	0,99	0,04256354	0,89913013				
rs1408783	0	0		0,04747503	0,89913013				
rs147121494	1,75	1,09	2,81	0,0229875	0,89913013	X	EDA	HGNC Symbol	ectodysplasin A [Source:HGNC Symbol;Acc:3157]
rs2296765	0,7	0,49	0,99	0,0404778	0,89913013	X	EDA	HGNC Symbol	ectodysplasin A [Source:HGNC Symbol;Acc:3157]
rs55933614	0,42	0,2	0,88	0,01676992	0,89913013				
rs62604281	0,4	0,19	0,84	0,011316	0,89913013				
rs607614	0,67	0,44	1,01	0,04674315	0,89913013				
rs150656555	0	0		0,00631385	0,86719195				
rs12689032	1,49	1,01	2,2	0,04813179	0,89913013	X	DGAT2L6	HGNC Symbol	diacylglycerol O-acyltransferase 2-like 6 [Source:HGNC Symbol;Acc:23250]
rs113548026	1,51	1,04	2,19	0,03340417	0,89913013				

SNP	OR	lower	upper	p-value	FDR	Chromosome	gene name	gene source	Description
rs73216714	1,55	1,08	2,22	0,01915864	0,89913013				
rs111499414	2,01	1,06	3,82	0,03538714	0,89913013	X	DLG3	HGNC Symbol	discs, large homolog 3 (Drosophila) [Source:HGNC Symbol;Acc:2902]
rs7472797	12,63	1,25	127,79	0,01841654	0,89913013				
rs142435968	12,09	1,19	122,62	0,0206162	0,89913013				
rs145367267	0,16	0,02	1,21	0,02105329	0,89913013	X	OGT	HGNC Symbol	O-linked N-acetylglucosamine (GlcNAc) transferase [Source:HGNC Symbol;Acc:8127]
rs36018136	0	0		0,0332433	0,89913013	X	NHSL2	HGNC Symbol	NHS-like 2 [Source:HGNC Symbol;Acc:33737]
rs78046994	0,23	0,05	1,02	0,02098371	0,89913013	X;HG1438_PATCH	HDAC8	HGNC Symbol	histone deacetylase 8 [Source:HGNC Symbol;Acc:13315]
rs3012642	0	0		0,01410736	0,89913013	X	HDAC8	HGNC Symbol	histone deacetylase 8 [Source:HGNC Symbol;Acc:13315]
rs5912109	0,32	0,09	1,11	0,04360175	0,89913013	X;HG1438_PATCH	PHKA1	HGNC Symbol	phosphorylase kinase, alpha 1 (muscle) [Source:HGNC Symbol;Acc:8925]
rs12688343	0	0		0,01240194	0,89913013	X;HG1438_PATCH	PHKA1	HGNC Symbol	phosphorylase kinase, alpha 1 (muscle) [Source:HGNC Symbol;Acc:8925]
rs62610693	1,76	1,03	3,03	0,04491497	0,89913013				
rs112573145	1,61	1,04	2,5	0,03806651	0,89913013	X	SLC16A2	HGNC Symbol	solute carrier family 16, member 2 (thyroid hormone transporter) [Source:HGNC Symbol;Acc:10923]
rs73625833	0	0		0,04591318	0,89913013				
rs12687919	0	0		0,04543333	0,89913013				

SNP	OR	lower	upper	p-value	FDR	Chromosome	gene name	gene source	Description
rs5937939	0	0		0,04591318	0,89913013	X	ABCB7	HGNC Symbol	ATP-binding cassette, sub-family B (MDR/TAP), member 7 [Source:HGNC Symbol;Acc:48]
rs5937362	0	0		0,02605322	0,89913013				
rs5937370	0	0		0,03412261	0,89913013	X	ZDHHC15	HGNC Symbol	zinc finger, DHHC-type containing 15 [Source:HGNC Symbol;Acc:20342]
rs58191293	0	0		0,03412261	0,89913013				
rs138831049	0	0		0,02200277	0,89913013				
rs145934807	0	0		0,04861353	0,89913013				
rs5912731	0	0		0,00910098	0,89913013				
rs148878183	2,77	1,03	7,43	0,04980804	0,89913013				
rs73223443	0,43	0,15	1,21	0,04332117	0,89913013				
rs115562366	2,68	1,09	6,58	0,03627224	0,89913013				
rs3123266	4,34	1,25	15,12	0,02286662	0,89913013				
rs11798879	2,37	1,04	5,37	0,04384666	0,89913013				
rs17328555	2,37	1,05	5,38	0,04336194	0,89913013				
rs147166358	2,75	1,3	5,8	0,00637507	0,86719195				

SNP	OR	lower	upper	p-value	FDR	Chromosome	gene name	gene source	Description
rs35199902	3,12	1,05	9,29	0,04690454	0,89913013				
rs5922652	0	0		0,03275316	0,89913013				
rs7884462	2,47	1,09	5,62	0,03533278	0,89913013				
rs12841496	1,6	1,14	2,24	0,00548404	0,86719195				
rs59117209	0,65	0,41	1,03	0,04902081	0,89913013	X	RP1-223D17.1	Clone-based (Vega)	
rs62615013	0	0		0,01638862	0,89913013				
rs148046761	1,93	1,16	3,22	0,01335711	0,89913013				
rs5922838	0,63	0,39	1,03	0,04917443	0,89913013				
rs5968205	0,63	0,39	1,03	0,04812554	0,89913013				
rs825552	0	0		0,00826894	0,897527099				
rs4375148	0,63	0,39	1,03	0,04626039	0,89913013				
rs17304335	0	0		0,01433062	0,89913013				
rs73232922	0	0		0,0184067	0,89913013				
rs9887469	0	0		0,00591737	0,86719195	X	RPS6KA6	HGNC Symbol	ribosomal protein S6 kinase, 90kDa, polypeptide 6 [Source:HGNC Symbol;Acc:10435]

SNP	OR	lower	upper	p-value	FDR	Chromosome	gene name	gene source	Description
rs5922934	1,51	1,08	2,11	0,01411354	0,89913013				
rs144084720	0	0		0,03128699	0,89913013				
rs12688565	1,53	1,09	2,15	0,01183833	0,89913013				
rs144829573	0	0		0,04115088	0,89913013	X	HDX	HGNC Symbol	highly divergent homeobox [Source:HGNC Symbol;Acc:26411]
rs151066610	0	0		0,02667721	0,89913013	X	HDX	HGNC Symbol	highly divergent homeobox [Source:HGNC Symbol;Acc:26411]
rs6623194	2,79	1,2	6,49	0,0133766	0,89913013				
rs1573689	0	0		0,02112517	0,89913013	X	RP1-215K18.4	Clone-based (Vega)	
rs7062047	0,46	0,21	1,01	0,04227316	0,89913013				
rs10126146	0,46	0,21	1	0,04094795	0,89913013	X	SATL1	HGNC Symbol	spermidine/spermine N1-acetyl transferase-like 1 [Source:HGNC Symbol;Acc:27992]
rs5968458	0,38	0,18	0,81	0,00818081	0,897527099				
rs16980121	0,17	0,02	1,34	0,03215707	0,89913013				
rs17252084	3,5	1,51	8,1	0,00407737	0,86719195				
rs73239767	0,35	0,13	0,96	0,02580871	0,89913013				
rs5923356	1,99	1,02	3,86	0,04190712	0,89913013				

SNP	OR	lower	upper	p-value	FDR	Chromosome	gene name	gene source	Description
rs1113139	0	0		0,042395	0,89913013				
rs2887578	0,69	0,49	0,99	0,03908714	0,89913013				
rs4828178	2,26	1,12	4,56	0,02448541	0,89913013				
rs5969235	1,45	1,03	2,06	0,0310668	0,89913013	X	KLHL4	HGNC Symbol	kelch-like family member 4 [Source:HGNC Symbol;Acc:6355]
rs1321406	0,58	0,32	1,04	0,0432408	0,89913013				
rs73241638	0	0		0,01710153	0,89913013				
rs2370013	0	0		0,00533376	0,86719195				
rs5969333	0,44	0,25	0,77	0,00071712	0,778251314				
rs2507106	0	0		0,00306197	0,836207794				
rs7063284	0,19	0,06	0,66	0,00182867	0,792635449				
rs5924181	0	0		0,0029881	0,836207794				
rs73504440	0	0		0,03865706	0,89913013				
rs7052636	0,42	0,18	0,97	0,0307613	0,89913013				
rs1883401	0,65	0,43	0,98	0,02993918	0,89913013				

SNP	OR	lower	upper	p-value	FDR	Chromosome	gene name	gene source	Description
rs35490131	2,26	1,16	4,42	0,01711381	0,89913013				
rs1540303	2,25	1,14	4,41	0,01931995	0,89913013				
rs2209054	1,42	1,02	1,98	0,03728832	0,89913013				
rs73244272	0	0		0,04846024	0,89913013				
rs113715166	0,59	0,34	1,02	0,03639403	0,89913013				
rs151000882	0,33	0,08	1,41	0,03529947	0,89913013				
rs112899483	0,18	0,02	1,39	0,03752808	0,89913013				
rs34273331	0	0		0,0355032	0,89913013				
rs2481333	0	0		0,01213957	0,89913013				
rs12853340	0	0		0,02799334	0,89913013				
rs142289898	1,54	1,03	2,3	0,03776738	0,89913013				
rs513756	0,68	0,47	0,97	0,02885309	0,89913013				
rs525869	1,45	1,04	2,04	0,02810346	0,89913013				
rs73242509	1,63	1,15	2,31	0,00682927	0,86719195				

SNP	OR	lower	upper	p-value	FDR	Chromosome	gene name	gene source	Description
rs138579599	1,84	1,24	2,71	0,00267297	0,836207794				
rs4020637	0,61	0,39	0,96	0,02129517	0,89913013	X	PCDH11X	HGNC Symbol	protocadherin 11 X-linked [Source:HGNC Symbol;Acc:8656]
rs2578894	0,64	0,43	0,96	0,02170023	0,89913013				
rs12841376	0,13	0,02	1,01	0,00885155	0,89913013				
rs785748	0,17	0,02	1,32	0,02980809	0,89913013				
rs785754	0,17	0,02	1,32	0,02980809	0,89913013				
rs1578337	0	0		0,00966356	0,89913013				
rs1198717	0,16	0,02	1,24	0,02333876	0,89913013				
rs1198718	0,16	0,02	1,24	0,02333876	0,89913013				
rs7891218	2,58	1,22	5,49	0,01553491	0,89913013	X	FAM133A	HGNC Symbol	family with sequence similarity 133, member A [Source:HGNC Symbol;Acc:26748]
rs73252252	0	0		0,04490713	0,89913013				
rs73538015	0	0		0,01916865	0,89913013				
rs5983168	2,4	1,16	4,95	0,02026286	0,89913013				
rs114786886	2,03	1,07	3,87	0,03405221	0,89913013				

SNP	OR	lower	upper	p-value	FDR	Chromosome	gene name	gene source	Description
rs4969708	0,3	0,09	1,07	0,03646468	0,89913013				
rs60757162	4,18	1,21	14,45	0,02580242	0,89913013				
rs5949599	7,23	1,71	30,53	0,00540401	0,86719195				
rs111335229	13,49	1,46	124,84	0,00808432	0,897527099				
rs73247163	0	0		0,03484934	0,89913013				
rs73250794	0	0		0,01369268	0,89913013	X	DIAPH2	HGNC Symbol	diaphanous-related formin 2 [Source:HGNC Symbol;Acc:2877]
rs35408243	6,12	1,39	27,01	0,01540034	0,89913013	X	DIAPH2	HGNC Symbol	diaphanous-related formin 2 [Source:HGNC Symbol;Acc:2877]
rs5967324	0	0		0,01078488	0,89913013	X	DIAPH2;DIAPH2-AS1	HGNC Symbol	diaphanous-related formin 2 [Source:HGNC Symbol;Acc:2877];DIAPH2 antisense RNA 1 [Source:HGNC Symbol;Acc:16972]
rs73258371	0,4	0,14	1,16	0,03374424	0,89913013				
rs400586	0,14	0,02	1,11	0,01473423	0,89913013				
rs6615988	1,48	1,06	2,08	0,02245488	0,89913013				
rs113294980	0	0		0,03237929	0,89913013				
rs16982961	2,92	1,18	7,21	0,0240584	0,89913013				
rs112879811	0,44	0,19	1,02	0,0429012	0,89913013				

SNP	OR	lower	upper	p-value	FDR	Chromosome	gene name	gene source	Description
rs17322905	0	0		0,03253769	0,89913013				
rs7062296	0	0		0,01234817	0,89913013				
rs5920819	6,34	1,11	36,25	0,0318293	0,89913013	X	PCDH19	HGNC Symbol	protocadherin 19 [Source:HGNC Symbol;Acc:14270]
rs28545751	10,37	1,05	102,58	0,02785807	0,89913013	X	PCDH19	HGNC Symbol	protocadherin 19 [Source:HGNC Symbol;Acc:14270]
rs41300169	6,29	1,44	27,56	0,0134698	0,89913013	X	PCDH19	HGNC Symbol	protocadherin 19 [Source:HGNC Symbol;Acc:14270]
rs62600479	7,51	1,32	42,82	0,01899135	0,89913013				
rs1204411	1,4	1	1,95	0,04973388	0,89913013	X	SRPX2	HGNC Symbol	sushi-repeat containing protein, X-linked 2 [Source:HGNC Symbol;Acc:30668]
rs73250616	1,52	1,08	2,13	0,01345651	0,89913013				
rs2154370	1,55	1,1	2,19	0,01104617	0,89913013				
rs11092279	0,66	0,47	0,93	0,01520831	0,89913013				
rs5920863	0,65	0,46	0,92	0,01285967	0,89913013	X	CSTF2	HGNC Symbol	cleavage stimulation factor, 3' pre-RNA, subunit 2, 64kDa [Source:HGNC Symbol;Acc:2484]
rs4828068	0,57	0,39	0,83	0,00176965	0,792635449	X	NOX1	HGNC Symbol	NADPH oxidase 1 [Source:HGNC Symbol;Acc:7889]
rs5967207	1,7	1,07	2,7	0,02921906	0,89913013				
rs17323346	3,08	1,38	6,84	0,00697493	0,86719195				

SNP	OR	lower	upper	p-value	FDR	Chromosome	gene name	gene source	Description
rs5921690	1,44	1,03	2,02	0,03047998	0,89913013				
rs12156914	0,62	0,42	0,91	0,00972133	0,89913013	X	TRMT2B-AS1	HGNC Symbol	TRMT2B antisense RNA 1 [Source:HGNC Symbol;Acc:41116]
rs17257634	11,44	1,13	115,65	0,02342017	0,89913013	X	DRP2	HGNC Symbol	dystrophin related protein 2 [Source:HGNC Symbol;Acc:3032]
rs41309506	5,77	1,3	25,56	0,01940604	0,89913013	X	TIMM8A	HGNC Symbol	translocase of inner mitochondrial membrane 8 homolog A (yeast) [Source:HGNC Symbol;Acc:11817]
rs2239460	0,18	0,02	1,39	0,03677942	0,89913013	X	BTK	HGNC Symbol	Bruton agammaglobulinemia tyrosine kinase [Source:HGNC Symbol;Acc:1133]
rs73250691	0	0		0,03623942	0,89913013	X;HG1439_PATCH	ARMCX4	HGNC Symbol	armadillo repeat containing, X-linked 4 [Source:HGNC Symbol;Acc:28615]
rs149192215	9,68	0,96	97,28	0,03481071	0,89913013				
rs147068824	0	0		0,00682226	0,86719195	X	NXF4	HGNC Symbol	nuclear RNA export factor 4 pseudogene [Source:HGNC Symbol;Acc:8074]
rs588849	0,57	0,33	1,01	0,03153896	0,89913013				
rs209095	0,62	0,4	0,96	0,02123498	0,89913013	X	NRK	HGNC Symbol	Nik related kinase [Source:HGNC Symbol;Acc:25391]
rs5917007	0	0		0,04461174	0,89913013				
rs5917052	1,53	1,09	2,14	0,01327544	0,89913013				
rs1012633	0,46	0,2	1,09	0,03138759	0,89913013				
rs17254207	1,51	1,07	2,12	0,01711097	0,89913013	X	TSC22D3	HGNC Symbol	TSC22 domain family, member 3 [Source:HGNC Symbol;Acc:3051]

SNP	OR	lower	upper	p-value	FDR	Chromosome	gene name	gene source	Description
rs5917070	1,44	1,02	2,02	0,03656719	0,89913013	X	NCBP2L	HGNC Symbol	nuclear cap binding protein subunit 2-like [Source:HGNC Symbol;Acc:31795]
rs73249873	0	0		0,01151822	0,89913013				
rs1581754	0	0		0,02619422	0,89913013				
rs5973822	0	0		0,03192697	0,89913013	X	ATG4A;COL4A6	HGNC Symbol	autophagy related 4A, cysteine peptidase [Source:HGNC Symbol;Acc:16489];collagen, type IV, alpha 6 [Source:HGNC Symbol;Acc:2208]
rs5973850	0,64	0,43	0,94	0,01818351	0,89913013	X	COL4A6	HGNC Symbol	collagen, type IV, alpha 6 [Source:HGNC Symbol;Acc:2208]
rs1042071	0,64	0,43	0,94	0,01818351	0,89913013	X	COL4A6	HGNC Symbol	collagen, type IV, alpha 6 [Source:HGNC Symbol;Acc:2208]
rs73251796	0	0		0,03192697	0,89913013	X	COL4A6	HGNC Symbol	collagen, type IV, alpha 6 [Source:HGNC Symbol;Acc:2208]
rs7061716	2,32	1,06	5,05	0,03296205	0,89913013				
rs5942780	2,33	1,07	5,08	0,03200333	0,89913013				
rs10521523	4,45	1,35	14,66	0,00292526	0,836207794				
rs140663280	0	0		0,03296698	0,89913013	X	AMMECR1; RGAG1	HGNC Symbol	Alport syndrome, mental retardation, midface hypoplasia and elliptocytosis chromosomal region gene 1 [Source:HGNC Symbol;Acc:467];retrotransposon gag domain containing 1
rs55676345	3,03	1,42	6,44	0,00478749	0,86719195				
rs111904771	5,75	1,31	25,15	0,01854466	0,89913013	X	PAK3	HGNC Symbol	p21 protein (Cdc42/Rac)-activated kinase 3 [Source:HGNC Symbol;Acc:8592]
rs6642884	0	0		0,01746995	0,89913013	X	CAPN6	HGNC Symbol	calpain 6 [Source:HGNC Symbol;Acc:1483]

SNP	OR	lower	upper	p-value	FDR	Chromosome	gene name	gene source	Description
rs7050529	3,18	1,08	9,38	0,04168948	0,89913013	X	TRPC5	HGNC Symbol	transient receptor potential cation channel, subfamily C, member 5 [Source:HGNC Symbol;Acc:12337]
rs73550175	14,92	1,42	156,93	0,01394723	0,89913013				
rs67057973	1,54	1,04	2,26	0,03273179	0,89913013				
rs150675937	0	0		0,04922634	0,89913013				
rs73213486	10,51	1,05	104,67	0,0276277	0,89913013				
rs17308368	0	0		0,03922405	0,89913013	X;HG1434_PATCH	RP5-964N17.1	Clone-based (Vega)	
rs17223372	0	0		0,03457224	0,89913013	X;HG1434_PATCH	RP5-964N17.1	Clone-based (Vega)	
rs56296825	1,65	1,18	2,3	0,00326196	0,853796107				
rs5929431	0,63	0,38	1,02	0,04274576	0,89913013				
rs5946131	2,43	1,03	5,73	0,04762552	0,89913013				
rs12557575	1,82	1,05	3,16	0,03724024	0,89913013				
rs1401414	1,41	1,01	1,96	0,04403783	0,89913013				
rs4911805	0	0		0,01555926	0,89913013	X;HG1462_PATCH	HTR2C	HGNC Symbol	5-hydroxytryptamine (serotonin) receptor 2C, G protein-coupled [Source:HGNC Symbol;Acc:5295]
rs1537755	3,06	1,22	7,67	0,01961783	0,89913013				

SNP	OR	lower	upper	p-value	FDR	Chromosome	gene name	gene source	Description
rs112561107	0,57	0,34	0,94	0,01461675	0,89913013				
rs139091357	0	0		0,03147117	0,89913013				
rs11091036	2,61	1,27	5,37	0,01004697	0,89913013				
rs61638747	0,68	0,48	0,98	0,03304755	0,89913013				
rs7889653	0,33	0,13	0,83	0,01018885	0,89913013				
rs12156686	0,19	0,02	1,48	0,04658787	0,89913013				
rs11798303	1,54	1,02	2,32	0,04446045	0,89913013				
rs5905270	2,06	1,05	4,06	0,03629584	0,89913013				
rs5952101	3,97	1,17	13,4	0,00993886	0,89913013	X;HG1463_PATCH	SLC6A14	HGNC Symbol;UniProtKB	solute carrier family 6 (amino acid transporter), member 14 [Source:HGNC Symbol;Acc:11047];
rs5905289	1,98	1,01	3,87	0,0478534	0,89913013	X	SLC6A14	HGNC Symbol	solute carrier family 6 (amino acid transporter), member 14 [Source:HGNC Symbol;Acc:11047]
rs62603537	0	0		0,00679565	0,86719195				
rs5911671	0	0		0,01487942	0,89913013				
rs6646884	0	0		0,02759889	0,89913013				
rs5958709	0,14	0,02	1,06	0,01166717	0,89913013				

SNP	OR	lower	upper	p-value	FDR	Chromosome	gene name	gene source	Description
rs7058127	0,57	0,37	0,9	0,00819257	0,897527099				
rs12008294	0,56	0,31	1,01	0,02989749	0,89913013				
rs5910238	0,6	0,37	0,98	0,02486303	0,89913013				
rs67316363	0	0		0,04921531	0,89913013				
rs12557784	0,53	0,28	0,99	0,02254833	0,89913013				
rs5956024	0	0		0,00021759	0,57740729				
rs73216861	5,15	1,37	19,32	0,01471113	0,89913013				
rs1294826	0,59	0,37	0,93	0,01454905	0,89913013	X	DOCK11	HGNC Symbol	dedicator of cytokinesis 11 [Source:HGNC Symbol;Acc:23483]
rs5910376	2,04	1,05	3,97	0,03531769	0,89913013	X	DOCK11	HGNC Symbol	dedicator of cytokinesis 11 [Source:HGNC Symbol;Acc:23483]
rs2286977	2,04	1,05	3,97	0,03531769	0,89913013	X	DOCK11	HGNC Symbol	dedicator of cytokinesis 11 [Source:HGNC Symbol;Acc:23483]
rs17326920	0,25	0,06	1,11	0,03179596	0,89913013				
rs7891792	0	0		0,03946148	0,89913013				
rs17261236	0	0		0,00635374	0,86719195	X	UPF3B	HGNC Symbol	UPF3 regulator of nonsense transcripts homolog B (yeast) [Source:HGNC Symbol;Acc:20439]
rs3788941	0,57	0,39	0,83	0,00185022	0,792635449	X	ATP1B4	HGNC Symbol	ATPase, Na+/K+ transporting, beta 4 polypeptide [Source:HGNC Symbol;Acc:808]

SNP	OR	lower	upper	p-value	FDR	Chromosome	gene name	gene source	Description
rs2192257	0,35	0,12	0,98	0,00740019	0,87580657				
rs6646733	2,81	1,34	5,87	0,0068559	0,86719195				
rs5909764	1,52	1,08	2,15	0,01736041	0,89913013				
rs73221033	3,82	1,25	11,62	0,02125436	0,89913013				
rs5909792	0,65	0,44	0,97	0,02655453	0,89913013				
rs2110461	4,33	1,1	17,07	0,03824009	0,89913013				
rs5957861	0	0		0,02662403	0,89913013				
rs5909870	0,58	0,38	0,89	0,00751626	0,87601443				
rs5957981	0	0		0,04378107	0,89913013				
rs5956458	0	0		0,0021183	0,792635449				
rs2622953	0	0		0,00378485	0,86719195				
rs35464043	9,15	0,9	93,01	0,04142898	0,89913013				
rs5909973	1,42	1,01	1,98	0,04207182	0,89913013	X	GRIA3	HGNC Symbol	glutamate receptor, ionotropic, AMPA 3 [Source:HGNC Symbol;Acc:4573]
rs56385541	2,27	1,15	4,5	0,01829588	0,89913013	X	GRIA3	HGNC Symbol	glutamate receptor, ionotropic, AMPA 3 [Source:HGNC Symbol;Acc:4573]

SNP	OR	lower	upper	p-value	FDR	Chromosome	gene name	gene source	Description
rs5911547	1,51	1,07	2,13	0,0202626	0,89913013	X	GRIA3	HGNC Symbol	glutamate receptor, ionotropic, AMPA 3 [Source:HGNC Symbol;Acc:4573]
rs2157292	1,75	1,11	2,77	0,01924619	0,89913013	X	GRIA3	HGNC Symbol	glutamate receptor, ionotropic, AMPA 3 [Source:HGNC Symbol;Acc:4573]
rs5911552	0	0		0,04208651	0,89913013	X	GRIA3	HGNC Symbol	glutamate receptor, ionotropic, AMPA 3 [Source:HGNC Symbol;Acc:4573]
rs142397190	10,48	1,05	104,21	0,02769308	0,89913013	X	GRIA3	HGNC Symbol	glutamate receptor, ionotropic, AMPA 3 [Source:HGNC Symbol;Acc:4573]
rs4825856	0,3	0,11	0,83	0,00083429	0,778251314	X	GRIA3	HGNC Symbol	glutamate receptor, ionotropic, AMPA 3 [Source:HGNC Symbol;Acc:4573]
rs4825483	0	0		0,02818823	0,89913013				
rs35551694	1,49	1,03	2,15	0,03805394	0,89913013				
rs17325366	1,48	1,06	2,07	0,02070029	0,89913013				
rs5956566	1,51	1,07	2,15	0,02161871	0,89913013	X	THOC2	HGNC Symbol	THO complex 2 [Source:HGNC Symbol;Acc:19073]
rs36053352	4,5	1,42	14,28	0,01185264	0,89913013				
rs62604518	1,57	1,05	2,35	0,03062797	0,89913013				
rs3005882	3,32	1,31	8,39	0,01348686	0,89913013				
rs3005851	3,11	1,29	7,46	0,01345925	0,89913013				
rs199426	0	0		0,01272246	0,89913013	X	STAG2	HGNC Symbol	stromal antigen 2 [Source:HGNC Symbol;Acc:11355]

SNP	OR	lower	upper	p-value	FDR	Chromosome	gene name	gene source	Description
rs35941521	0	0		0,00848863	0,89913013	X	STAG2	HGNC Symbol	stromal antigen 2 [Source:HGNC Symbol;Acc:11355]
rs1279819	0	0		0,02681337	0,89913013	X	STAG2	HGNC Symbol	stromal antigen 2 [Source:HGNC Symbol;Acc:11355]
rs1279817	1,44	1,03	2,01	0,03094524	0,89913013	X	STAG2	HGNC Symbol	stromal antigen 2 [Source:HGNC Symbol;Acc:11355]
rs6608181	1,63	1,15	2,3	0,00487808	0,86719195	X	STAG2	HGNC Symbol	stromal antigen 2 [Source:HGNC Symbol;Acc:11355]
rs73557901	0	0		0,02836699	0,89913013	X	STAG2	HGNC Symbol	stromal antigen 2 [Source:HGNC Symbol;Acc:11355]
rs5911806	1,87	1,32	2,63	0,00032487	0,57740729	X	STAG2	HGNC Symbol	stromal antigen 2 [Source:HGNC Symbol;Acc:11355]
rs151184635	6,09	2,6	14,23	2,9193E-05	0,41510095	X	STAG2	HGNC Symbol	stromal antigen 2 [Source:HGNC Symbol;Acc:11355]
rs2356408	0,62	0,44	0,88	0,00638857	0,86719195	X	STAG2	HGNC Symbol	stromal antigen 2 [Source:HGNC Symbol;Acc:11355]
rs2239478	1,97	1,02	3,84	0,04481631	0,89913013	X	TENM1;STAG2	HGNC Symbol	teneurin transmembrane protein 1 [Source:HGNC Symbol;Acc:8117];stromal antigen 2 [Source:HGNC Symbol;Acc:11355]
rs2072886	0,49	0,25	0,97	0,01637806	0,89913013	X	TENM1;STAG2	HGNC Symbol	teneurin transmembrane protein 1 [Source:HGNC Symbol;Acc:8117];stromal antigen 2 [Source:HGNC Symbol;Acc:11355]
rs7058630	0	0		0,02916666	0,89913013	X	TENM1	HGNC Symbol	teneurin transmembrane protein 1 [Source:HGNC Symbol;Acc:8117]
rs111472283	1,66	1,14	2,4	0,00872253	0,89913013	X	TENM1	HGNC Symbol	teneurin transmembrane protein 1 [Source:HGNC Symbol;Acc:8117]
rs2858445	0,18	0,02	1,38	0,03613442	0,89913013	X	TENM1	HGNC Symbol	teneurin transmembrane protein 1 [Source:HGNC Symbol;Acc:8117]
rs5958557	2,25	1,11	4,57	0,02664739	0,89913013	X	TENM1	HGNC Symbol	teneurin transmembrane protein 1 [Source:HGNC Symbol;Acc:8117]

SNP	OR	lower	upper	p-value	FDR	Chromosome	gene name	gene source	Description
rs2206237	3,91	1,18	12,94	0,0295903	0,89913013	X	TENM1	HGNC Symbol	teneurin transmembrane protein 1 [Source:HGNC Symbol;Acc:8117]
rs3859908	4,7	1,47	14,98	0,01007462	0,89913013	X	TENM1	HGNC Symbol	teneurin transmembrane protein 1 [Source:HGNC Symbol;Acc:8117]
rs73215144	14,32	1,54	132,87	0,00680407	0,86719195	X	TENM1	HGNC Symbol	teneurin transmembrane protein 1 [Source:HGNC Symbol;Acc:8117]
rs1569565	4,6	1,17	18,03	0,03056798	0,89913013	X	TENM1	HGNC Symbol	teneurin transmembrane protein 1 [Source:HGNC Symbol;Acc:8117]
rs16998333	2,37	1,21	4,64	0,01218999	0,89913013				
rs4830000	0,5	0,26	0,99	0,04257709	0,89913013				
rs73216164	5,18	1,3	20,72	0,02164301	0,89913013				
rs73214455	18,68	2,1	166,4	0,00153253	0,778251314				
rs73214463	18,68	2,1	166,4	0,00153253	0,778251314				
rs1305003	1,42	1,02	1,99	0,03725472	0,89913013				
rs139880202	4,16	1,18	14,66	0,02854828	0,89913013				
rs7056175	0,41	0,16	1,03	0,04225677	0,89913013				
rs2878431	1,43	1,01	2,02	0,04627524	0,89913013				
rs7882213	0	0		0,03810421	0,89913013				

SNP	OR	lower	upper	p-value	FDR	Chromosome	gene name	gene source	Description
rs6635844	0,69	0,47	1	0,04306228	0,89913013				
rs73216923	3,93	1,06	14,51	0,04560695	0,89913013				
rs73236598	3,93	1,06	14,51	0,04560695	0,89913013				
rs5975024	4,93	2,09	11,67	0,00031682	0,57740729	X	RP1-30E17.2	Clone-based (Vega)	
rs55839915	6,86	2,65	17,81	6,417E-05	0,456213349	X	RP1-30E17.2	Clone-based (Vega)	
rs5976834	4,69	1,94	11,35	0,00069562	0,778251314				
rs5932549	0	0		0,04137046	0,89913013				
rs4830111	0,57	0,3	1,07	0,04810306	0,89913013				
rs737159	2,26	1,05	4,88	0,04190694	0,89913013				
rs5977001	9,39	0,95	93,11	0,0355467	0,89913013				
rs5930347	3,22	0,9	11,52	0,04858293	0,89913013				
rs3116744	0,65	0,43	0,99	0,03509935	0,89913013				
rs1997670	0	0		0,0403914	0,89913013				
rs5977166	0	0		0,030528	0,89913013	X	RP4-537K23.4	Clone-based (Vega)	

SNP	OR	lower	upper	p-value	FDR	Chromosome	gene name	gene source	Description
rs73633939	4,57	1,51	13,78	0,00766572	0,886170178				
rs73225593	15,09	1,62	140,63	0,00581791	0,86719195	X	BCORL1	HGNC Symbol	BCL6 corepressor-like 1 [Source:HGNC Symbol;Acc:25657]
rs3788	2,21	1,04	4,68	0,04264728	0,89913013	X	ARHGAP36	HGNC Symbol	Rho GTPase activating protein 36 [Source:HGNC Symbol;Acc:26388]
rs62617185	1,66	1,04	2,66	0,03980533	0,89913013	X	MBNL3	HGNC Symbol	muscleblind-like splicing regulator 3 [Source:HGNC Symbol;Acc:20564]
rs146450227	0	0		0,03303747	0,89913013				
rs242160	0,66	0,44	1,01	0,04322191	0,89913013				
rs5977998	3,48	1,06	11,47	0,04522879	0,89913013				
rs5930917	1,4	1	1,95	0,04676561	0,89913013				
rs144729234	0	0		0,04829538	0,89913013	X	RP11-308D16.4;RP11-	Clone-based (Vega)	
rs5976162	1,97	1,18	3,29	0,01099612	0,89913013				
rs619373	0	0		0,00721314	0,86719195	X	FGF13	HGNC Symbol	fibroblast growth factor 13 [Source:HGNC Symbol;Acc:3670]
rs9988241	0,42	0,15	1,2	0,0403286	0,89913013	X	FGF13	HGNC Symbol	fibroblast growth factor 13 [Source:HGNC Symbol;Acc:3670]
rs12842104	0,62	0,38	1,01	0,03867785	0,89913013				
rs374508	0,58	0,35	0,99	0,02669266	0,89913013				

SNP	OR	lower	upper	p-value	FDR	Chromosome	gene name	gene source	Description
rs4829994	1,5	1,04	2,16	0,03125473	0,89913013				
rs5931666	1,5	1,04	2,16	0,03125473	0,89913013				
rs6048	1,45	1,03	2,06	0,03811173	0,89913013	X	F9	HGNC Symbol	coagulation factor IX [Source:HGNC Symbol;Acc:3551]
rs5909021	0	0		0,04848419	0,89913013				
rs5907587	1,99	1,1	3,59	0,02453798	0,89913013				
rs147682400	0	0		0,04787387	0,89913013				
rs41381447	2,68	1,1	6,54	0,03524322	0,89913013				
rs12387620	0	0		0,04339548	0,89913013				
rs12557948	0,52	0,33	0,83	0,00207752	0,792635449				
rs146681286	0,19	0,02	1,5	0,04885058	0,89913013				
rs844956	0,63	0,42	0,93	0,01548365	0,89913013				
rs926809	3,95	1,97	7,92	0,00010606	0,501260262				
rs6636278	3,33	1,7	6,53	0,00041844	0,661092438				
rs2864953	0,63	0,42	0,93	0,01371004	0,89913013				

SNP	OR	lower	upper	p-value	FDR	Chromosome	gene name	gene source	Description
rs17282467	0,44	0,2	0,93	0,02602898	0,89913013				
rs5908013	0,48	0,23	1,02	0,04830999	0,89913013				
rs12009454	0,58	0,34	1,02	0,03571856	0,89913013				
rs73577990	0	0		0,04190474	0,89913013	X	MAGEC3	HGNC Symbol	melanoma antigen family C, 3 [Source:HGNC Symbol;Acc:23798]
rs16980287	0	0		0,02063883	0,89913013				
rs111329029	1,42	1,01	1,98	0,04174453	0,89913013				
rs12390344	2,07	1,05	4,09	0,03859825	0,89913013				
rs12390872	0,65	0,45	0,95	0,01931718	0,89913013				
rs717631	2,17	1,04	4,52	0,04186386	0,89913013				
rs73229758	0	0		0,03931469	0,89913013				
rs62600572	16,57	1,87	146,71	0,00234725	0,808955961				
rs2224815	0,71	0,5	1,01	0,04981619	0,89913013				
rs139194674	0	0		0,01331679	0,89913013				
rs5907416	2,38	1,26	4,47	0,00745289	0,87580657	X;HG1458_PATCH	GS1-256O22.5	Clone-based (Vega)	

SNP	OR	lower	upper	p-value	FDR	Chromosome	gene name	gene source	Description
rs728395	1,6	1,04	2,46	0,03522028	0,89913013	X;HG1458_PATCH	GS1-256O22.5	Clone-based (Vega)	
rs5908648	1,95	1	3,81	0,04675683	0,89913013	X;HG1458_PATCH	GS1-256O22.5	Clone-based (Vega)	
rs5908660	2,1	1,05	4,2	0,03808359	0,89913013	X;HG1458_PATCH	GS1-256O22.5	Clone-based (Vega)	
rs73235693	0	0		0,0375892	0,89913013				
rs7056059	0	0		0,01912506	0,89913013				
rs73232180	12,49	1,24	125,43	0,01861477	0,89913013				
rs12013685	6,41	1,11	36,93	0,03177299	0,89913013				
rs73233724	4,38	1,26	15,21	0,0217096	0,89913013				
rs1980775	0,62	0,37	1,02	0,04059037	0,89913013				
rs4827773	0,63	0,43	0,92	0,01261976	0,89913013				
rs5919803	0,63	0,43	0,91	0,00996289	0,89913013				
rs57580842	0,64	0,44	0,93	0,01552055	0,89913013				
rs5966452	0,65	0,45	0,94	0,01761332	0,89913013				
rs6626331	1,98	1,02	3,84	0,04492055	0,89913013				

SNP	OR	lower	upper	p-value	FDR	Chromosome	gene name	gene source	Description
rs41416052	3,32	1,19	9,21	0,02495767	0,89913013				
rs2891670	2,33	1,13	4,8	0,02423303	0,89913013				
rs138367545	5,84	1,32	25,85	0,01850999	0,89913013				
rs146458110	5,57	1,48	20,97	0,01094727	0,89913013				
rs142778097	4,36	1,1	17,19	0,03770567	0,89913013				
rs55810646	0	0		0,02409952	0,89913013	X	SLITRK2	HGNC Symbol	SLIT and NTRK-like family, member 2 [Source:HGNC Symbol;Acc:13449]
rs147425194	0,16	0,02	1,26	0,02486252	0,89913013				
rs12556597	2,25	1,08	4,68	0,03365265	0,89913013				
rs5965660	0,58	0,34	1,01	0,03279048	0,89913013	X;HG1459_PATCH	MIR888	HGNC Symbol	microRNA 888 [Source:HGNC Symbol;Acc:33648]
rs5920027	1,42	1,02	1,98	0,03783037	0,89913013				
rs6626484	0,32	0,14	0,74	0,00426641	0,86719195				
rs5920062	0,35	0,13	0,94	0,02322297	0,89913013				
rs72611215	0,35	0,11	1,06	0,0408938	0,89913013				
rs6626232	0	0		0,00966747	0,89913013				

SNP	OR	lower	upper	p-value	FDR	Chromosome	gene name	gene source	Description
rs1323747	0,67	0,44	1,01	0,04436516	0,89913013				
rs12014784	0	0		0,0289477	0,89913013				
rs11797123	0	0		0,04606652	0,89913013				
rs1805423	0	0		0,02561954	0,89913013	X;HG1459_PATCH	FMR1	HGNC Symbol	fragile X mental retardation 1 [Source:HGNC Symbol;Acc:3775]
rs11798727	0,6	0,39	0,92	0,01174422	0,89913013				
rs241132	7,63	1,3	44,86	0,02007359	0,89913013	X;HG1459_PATCH	AFF2	HGNC Symbol	AF4/FMR2 family, member 2 [Source:HGNC Symbol;Acc:3776]
rs12848014	0,57	0,37	0,88	0,00630761	0,86719195	X	AFF2	HGNC Symbol	AF4/FMR2 family, member 2 [Source:HGNC Symbol;Acc:3776]
rs5741912	0	0		0,04088854	0,89913013				
rs7052815	0	0		0,03936712	0,89913013				
rs5925421	2,7	1,05	6,96	0,04486543	0,89913013				
rs34964358	3,89	1,11	13,65	0,03650553	0,89913013				
rs147270834	0	0		0,01269524	0,89913013				
rs693913	1,68	1,19	2,37	0,00250089	0,826979086	X	MAMLD1	HGNC Symbol	mastermind-like domain containing 1 [Source:HGNC Symbol;Acc:2568]
rs150042509	8,99	2,21	36,65	0,00122056	0,778251314	X	MAMLD1	HGNC Symbol	mastermind-like domain containing 1 [Source:HGNC Symbol;Acc:2568]

SNP	OR	lower	upper	p-value	FDR	Chromosome	gene name	gene source	Description
rs5925148	0,57	0,38	0,87	0,0047471	0,86719195	X	MAMLD1	HGNC Symbol	mastermind-like domain containing 1 [Source:HGNC Symbol;Acc:2568]
rs1515963	5,53	1,14	26,88	0,03551337	0,89913013	X;HG1459_PATCH	MTM1	HGNC Symbol	myotubularin 1 [Source:HGNC Symbol;Acc:7448]
rs73250555	0,23	0,05	1,03	0,02289458	0,89913013	X;HG1459_PATCH	MTM1	HGNC Symbol	myotubularin 1 [Source:HGNC Symbol;Acc:7448]
rs5925388	0,69	0,49	0,98	0,03532244	0,89913013	X;HG1459_PATCH	MTM1	HGNC Symbol	myotubularin 1 [Source:HGNC Symbol;Acc:7448]
rs73250569	0,24	0,05	1,05	0,02443409	0,89913013	X;HG1459_PATCH	MTMR1	HGNC Symbol	myotubularin related protein 1 [Source:HGNC Symbol;Acc:7449]
rs237397	1,42	1,02	1,99	0,03920105	0,89913013				
rs7057942	0,31	0,12	0,78	0,00638351	0,86719195				
rs5924858	0,4	0,16	0,97	0,03110427	0,89913013				
rs5925505	2,11	1,03	4,31	0,04370938	0,89913013				
rs12171762	0	0		0,02295894	0,89913013				
rs7881137	5,13	1,09	24,09	0,03991765	0,89913013				
rs12009129	0	0		0,02065496	0,89913013				
rs180488	0	0		0,00601468	0,86719195				
rs78972460	2	1,2	3,33	0,00902046	0,89913013				

SNP	OR	lower	upper	p-value	FDR	Chromosome	gene name	gene source	Description
rs1937215	1,87	1,1	3,19	0,02428786	0,89913013				
rs12010578	1,57	1,06	2,32	0,02567376	0,89913013				
rs741725	3,76	1,23	11,53	0,02323498	0,89913013				
rs145754054	0	0		0,02613196	0,89913013				
rs73241852	0,51	0,26	1,01	0,02659347	0,89913013				
rs210567	0,58	0,34	0,97	0,02250883	0,89913013				
rs7051108	0,44	0,19	1	0,03928073	0,89913013				
rs58707270	0	0		0,00366668	0,86719195				
rs4833	1,56	1,11	2,19	0,01095382	0,89913013	X;HG1497_PATCH	BGN	HGNC Symbol	biglycan [Source:HGNC Symbol;Acc:1044]
rs80276708	1,4	1	1,95	0,04692663	0,89913013	X;HG1497_PATCH	ABCD1	HGNC Symbol	ATP-binding cassette, sub-family D (ALD), member 1 [Source:HGNC Symbol;Acc:61]
rs766420	0,59	0,34	1,03	0,03906681	0,89913013	X;HG1497_PATCH	TKTL1	HGNC Symbol	transketolase-like 1 [Source:HGNC Symbol;Acc:11835]
rs73247656	0	0		0,00187835	0,792635449				
rs62617845	0	0		0,03022753	0,89913013	X;HG1497_PATCH	G6PD	HGNC Symbol	glucose-6-phosphate dehydrogenase [Source:HGNC Symbol;Acc:4057]
rs28370229	0	0		0,04078604	0,89913013	X	F8	HGNC Symbol	coagulation factor VIII, procoagulant component [Source:HGNC Symbol;Acc:3546]

SNP	OR	lower	upper	p-value	FDR	Chromosome	gene name	gene source	Description
rs28370241	0	0		0,04309874	0,89913013	X	F8	HGNC Symbol	coagulation factor VIII, procoagulant component [Source:HGNC Symbol;Acc:3546]
rs28370214	0	0		0,00450889	0,86719195	X;HG1497_PATCH	F8	HGNC Symbol	coagulation factor VIII, procoagulant component [Source:HGNC Symbol;Acc:3546]
rs62619847	0	0		0,03627172	0,89913013	X;HG1497_PATCH	FUNDC2	HGNC Symbol	FUN14 domain containing 2 [Source:HGNC Symbol;Acc:24925]
rs5940560	0	0		0,01697879	0,89913013				
rs142918084	0	0		0,01643398	0,89913013				
rs306890	0,52	0,29	0,94	0,02801536	0,89913013				
rs73249628	1,82	1,04	3,18	0,03742219	0,89913013				
rs55857040	9,6	0,95	96,99	0,03586639	0,89913013	X	SPRY3	HGNC Symbol	sprouty homolog 3 (Drosophila) [Source:HGNC Symbol;Acc:11271]
rs28729587	2,42	1,29	4,54	0,00672067	0,86719195	X	SPRY3	HGNC Symbol	sprouty homolog 3 (Drosophila) [Source:HGNC Symbol;Acc:11271]
rs77442791	0,42	0,21	0,82	0,00789557	0,897527099	X	SPRY3	HGNC Symbol	sprouty homolog 3 (Drosophila) [Source:HGNC Symbol;Acc:11271]
rs700455	0,6	0,36	0,99	0,03839945	0,89913013	X	SPRY3	HGNC Symbol	sprouty homolog 3 (Drosophila) [Source:HGNC Symbol;Acc:11271]
rs306873	0,58	0,35	0,94	0,02447844	0,89913013	X	SPRY3	HGNC Symbol	sprouty homolog 3 (Drosophila) [Source:HGNC Symbol;Acc:11271]
rs700462	1,81	1,11	2,94	0,01708695	0,89913013				
rs802480	1,97	1,21	3,23	0,00612754	0,86719195				

SNP	OR	lower	upper	p-value	FDR	Chromosome	gene name	gene source	Description
rs35519384	2,67	1,34	5,3	0,00561689	0,86719195				
rs1883079	0,18	0,02	1,42	0,04069694	0,89913013	X	IL9R	HGNC Symbol	interleukin 9 receptor [Source:HGNC Symbol;Acc:6030]
rs7892580	0,44	0,21	0,94	0,02253251	0,89913013	X	PLCXD1	HGNC Symbol	phosphatidylinositol-specific phospholipase C, X domain containing 1 [Source:HGNC Symbol;Acc:23148]
rs5950688	0,53	0,29	0,99	0,03759345	0,89913013				
rs28599889	0,55	0,32	0,96	0,02792906	0,89913013				
rs61080188	1,68	1,02	2,76	0,04063565	0,89913013				
rs6645165	0,57	0,35	0,94	0,0242275	0,89913013				
rs28404660	0,19	0,02	1,45	0,04356787	0,89913013				
rs35099437	1,96	1,18	3,26	0,00822113	0,897527099	X	SHOX	HGNC Symbol	short stature homeobox [Source:HGNC Symbol;Acc:10853]
rs28603143	0,52	0,26	1,03	0,04676883	0,89913013	X	SHOX	HGNC Symbol	short stature homeobox [Source:HGNC Symbol;Acc:10853]
rs73190324	0	0		0,02852416	0,89913013	X	SHOX	HGNC Symbol	short stature homeobox [Source:HGNC Symbol;Acc:10853]
rs4472693	0,43	0,22	0,85	0,00973953	0,89913013				
rs6579694	0,58	0,35	0,94	0,0238253	0,89913013				
rs2037897	0,49	0,28	0,86	0,00944866	0,89913013				

SNP	OR	lower	upper	p-value	FDR	Chromosome	gene name	gene source	Description
rs5988600	0,47	0,24	0,93	0,02064391	0,89913013				
rs5988645	2,66	1,45	4,85	0,00143123	0,778251314				
rs73180407	0	0		0,04856688	0,89913013				
rs5946343	2	1,04	3,83	0,04165909	0,89913013				
rs28780986	1,74	1,04	2,91	0,03377398	0,89913013				
rs5946570	1,59	1,01	2,5	0,04437974	0,89913013				
rs7885174	1,78	1,11	2,86	0,01769723	0,89913013				
rs5946611	0,34	0,1	1,14	0,04488111	0,89913013				
rs4131911	0	0		0,00556284	0,86719195				
rs34453751	2,02	1,19	3,45	0,00956916	0,89913013				
rs35811834	2,3	1,33	3,96	0,00264613	0,836207794				
rs62605876	1,81	1,09	3	0,02123595	0,89913013				
rs67734326	1,64	1,03	2,6	0,03443001	0,89913013				
rs34745620	2,02	1,25	3,28	0,00384695	0,86719195				

SNP	OR	lower	upper	p-value	FDR	Chromosome	gene name	gene source	Description
rs114538020	0,43	0,21	0,86	0,00999348	0,89913013				
rs28535804	1,67	1,01	2,74	0,04346955	0,89913013	X	CSF2RA	HGNC Symbol	colony stimulating factor 2 receptor, alpha, low-affinity (granulocyte-macrophage) [Source:HGNC Symbol;Acc:2435]
rs28377023	1,9	1,14	3,15	0,01208915	0,89913013	X	CSF2RA	HGNC Symbol	colony stimulating factor 2 receptor, alpha, low-affinity (granulocyte-macrophage) [Source:HGNC Symbol;Acc:2435]
rs6647005	0,32	0,09	1,09	0,0370106	0,89913013	X	IL3RA	HGNC Symbol	interleukin 3 receptor, alpha (low affinity) [Source:HGNC Symbol;Acc:6012]
rs35544304	0,37	0,18	0,76	0,00311438	0,836207794	X	P2RY8	HGNC Symbol	purinergic receptor P2Y, G-protein coupled, 8 [Source:HGNC Symbol;Acc:15524]
rs28578016	2,72	1,49	4,98	0,00108524	0,778251314	X	P2RY8	HGNC Symbol	purinergic receptor P2Y, G-protein coupled, 8 [Source:HGNC Symbol;Acc:15524]
rs28391357	0,62	0,38	1,01	0,04853501	0,89913013	X	P2RY8	HGNC Symbol	purinergic receptor P2Y, G-protein coupled, 8 [Source:HGNC Symbol;Acc:15524]
rs73186942	0,37	0,13	1,11	0,04845183	0,89913013	X	P2RY8	HGNC Symbol	purinergic receptor P2Y, G-protein coupled, 8 [Source:HGNC Symbol;Acc:15524]
rs28619240	0,35	0,12	1,05	0,03609611	0,89913013	X	P2RY8	HGNC Symbol	purinergic receptor P2Y, G-protein coupled, 8 [Source:HGNC Symbol;Acc:15524]
rs73186974	0,37	0,13	1,11	0,04845183	0,89913013	X	P2RY8	HGNC Symbol	purinergic receptor P2Y, G-protein coupled, 8 [Source:HGNC Symbol;Acc:15524]
rs28490591	1,64	1,02	2,63	0,03934155	0,89913013				
rs5989833	0,56	0,31	1,01	0,04404109	0,89913013				
rs4639690	0,55	0,32	0,94	0,02521529	0,89913013	X	ASMT	HGNC Symbol	acetylserotonin O-methyltransferase [Source:HGNC Symbol;Acc:750]
rs6644678	0,61	0,38	0,99	0,04132591	0,89913013	X	RP13-297E16.4	Clone-based (Vega)	

SNP	OR	lower	upper	p-value	FDR	Chromosome	gene name	gene source	Description
rs5989945	0,42	0,19	0,89	0,01477725	0,89913013				
rs7878782	0	0		0,01750005	0,89913013				
rs116865723	1,79	1,02	3,15	0,04434182	0,89913013				
rs7054570	1,91	1,1	3,31	0,02241588	0,89913013				
rs7879755	1,92	1,09	3,38	0,02484265	0,89913013	X	DHRSX	HGNC Symbol	dehydrogenase/reductase (SDR family) X-linked [Source:HGNC Symbol;Acc:18399]
rs73173783	1,74	1,01	3	0,04916019	0,89913013	X	DHRSX	HGNC Symbol	dehydrogenase/reductase (SDR family) X-linked [Source:HGNC Symbol;Acc:18399]
rs61148409	0	0		0,00507358	0,86719195	X	DHRSX	HGNC Symbol	dehydrogenase/reductase (SDR family) X-linked [Source:HGNC Symbol;Acc:18399]
rs6642167	1,95	1,17	3,28	0,01152807	0,89913013	X	DHRSX	HGNC Symbol	dehydrogenase/reductase (SDR family) X-linked [Source:HGNC Symbol;Acc:18399]
rs6641784	2,88	1,05	7,88	0,04494392	0,89913013	X	DHRSX	HGNC Symbol	dehydrogenase/reductase (SDR family) X-linked [Source:HGNC Symbol;Acc:18399]
rs140302401	2,5	1,06	5,91	0,04062963	0,89913013	X	DHRSX;ZBED1	HGNC Symbol	dehydrogenase/reductase (SDR family) X-linked [Source:HGNC Symbol;Acc:18399];zinc finger, BED-type containing 1 [Source:HGNC Symbol;Acc:447]
rs5982733	3,39	1,03	11,16	0,04920866	0,89913013				
rs6641973	0,57	0,33	0,98	0,03798619	0,89913013	X	CD99P1	HGNC Symbol	CD99 molecule pseudogene 1 [Source:HGNC Symbol;Acc:7083]
rs6567632	0,5	0,26	0,96	0,02607372	0,89913013	X	CD99P1	HGNC Symbol	CD99 molecule pseudogene 1 [Source:HGNC Symbol;Acc:7083]
rs145435329	0	0		0,03591861	0,89913013	X	CD99P1	HGNC Symbol	CD99 molecule pseudogene 1 [Source:HGNC Symbol;Acc:7083]

	SNP	OR	lower	upper	p-value	FDR	Chromosome	gene name	gene source	Description
	rs184301	0,13	0,02	0,99	0,00872956	0,89913013	X	CD99	HGNC Symbol	CD99 molecule [Source:HGNC Symbol;Acc:7082]
	rs1136470	0,46	0,2	1,04	0,04158426	0,89913013	X	CD99	HGNC Symbol	CD99 molecule [Source:HGNC Symbol;Acc:7082]
	rs73188898	4,75	1,84	12,27	0,00150999	0,778251314				
	rs2857316	0,36	0,17	0,8	0,00885927	0,89913013	X	XG	HGNC Symbol	Xg blood group [Source:HGNC Symbol;Acc:12806]

	SNP	OR	lower	upper	p-value	FDR	Chromosome	gene name	gene_source	description
Female (adjusted)	rs11730582	0,54	0,34	0,86	0,00838627	0,142566552	4	SPP1	HGNC Symbol	Osteopontin; Secreted phosphoprotein 1; HGNC:11255
	rs1718119	0,6	0,36	1,01	0,04708687	0,266825576	12	P2RX7	HGNC Symbol	purinergic receptor P2X, ligand-gated ion channel, 7 [Source:HGNC Symbol;Acc:8537]
	rs8086340	0,6	0,38	0,95	0,0248215	0,210982745	18	TNFRSF11A	HGNC Symbol	tumor necrosis factor receptor superfamily, member 11a, NFKB activator [Source:HGNC Symbol;Acc:11908]
	rs1419931	0,48	0,25	0,94	0,02356344	0,909373673	X	XG	HGNC Symbol	Xg blood group [Source:HGNC Symbol;Acc:12806]
	rs4892892	0,61	0,36	1,01	0,049083	0,909373673	X	XG	HGNC Symbol	Xg blood group [Source:HGNC Symbol;Acc:12806]
	rs311196	0,59	0,36	0,97	0,03104218	0,909373673	X	XG	HGNC Symbol	Xg blood group [Source:HGNC Symbol;Acc:12806]
	rs12008127	0,43	0,19	0,96	0,02190869	0,909373673	X	GYG2	HGNC Symbol	glycogenin 2 [Source:HGNC Symbol;Acc:4700]

SNP	OR	lower	upper	p-value	FDR	Chromosome	gene name	gene source	Description
rs73193064	0	0		0,01922319	0,909373673				
rs41304689	0	0		0,02603051	0,909373673	X	MXRA5	HGNC Symbol	matrix-remodelling associated 5 [Source:HGNC Symbol;Acc:7539]
rs5939496	0,33	0,1	1,08	0,03054488	0,909373673	X	MXRA5	HGNC Symbol	matrix-remodelling associated 5 [Source:HGNC Symbol;Acc:7539]
rs5939184	1,57	1	2,45	0,04769517	0,909373673	X	MXRA5	HGNC Symbol	matrix-remodelling associated 5 [Source:HGNC Symbol;Acc:7539]
rs11152506	1,59	1,02	2,46	0,03752818	0,909373673				
rs56157309	1,87	1,15	3,03	0,01149529	0,909373673				
rs7889116	0,24	0,05	1,05	0,0227701	0,909373673				
rs140881654	0,27	0,06	1,17	0,03882608	0,909373673				
rs72619401	0,16	0,02	1,2	0,01549737	0,909373673				
rs6567569	1,65	1,04	2,64	0,03627786	0,909373673	X	PRKX	HGNC Symbol	protein kinase, X-linked [Source:HGNC Symbol;Acc:9441]
rs73178179	2,53	1,09	5,89	0,03816648	0,909373673				
rs7877157	0,45	0,22	0,92	0,01611722	0,909373673				
rs17331195	0,32	0,1	1,09	0,03706319	0,909373673				
rs4826886	1,76	1,04	2,99	0,03872933	0,909373673				

SNP	OR	lower	upper	p-value	FDR	Chromosome	gene name	gene source	Description
rs113371969	0	0		0,01429798	0,909373673				
rs113862114	0	0		0,0070683	0,89300867				
rs7052908	1,57	1	2,46	0,04820285	0,909373673				
rs3843796	0,48	0,25	0,92	0,01959204	0,909373673				
rs17219044	5,57	1,43	21,64	0,01504621	0,909373673				
rs4110045	0,32	0,09	1,07	0,03236669	0,909373673				
rs144047820	2,42	1,11	5,28	0,03170083	0,909373673				
rs5962226	5,31	2,27	12,43	0,00017955	0,873550665				
rs111959810	0	0		0,04900756	0,909373673				
rs144409421	2,36	1,12	4,99	0,02871283	0,909373673				
rs2218680	1,67	1,06	2,63	0,02665838	0,909373673				
rs17304608	0	0		0,01334084	0,909373673				
rs7065808	2,31	1,16	4,61	0,02083979	0,909373673				
rs1993794	0	0		0,00637044	0,884530262				

SNP	OR	lower	upper	p-value	FDR	Chromosome	gene name	gene source	Description
rs113732757	3,32	1,48	7,44	0,00444582	0,873550665				
rs144132782	0,26	0,08	0,85	0,00718198	0,89300867				
rs149156025	0,2	0,03	1,52	0,04913501	0,909373673				
rs5916158	0,59	0,36	0,95	0,02572878	0,909373673				
rs5916162	0,58	0,36	0,95	0,02357803	0,909373673				
rs17220204	2,25	1,22	4,17	0,01145101	0,909373673				
rs5916174	0,6	0,37	0,99	0,0398373	0,909373673				
rs5961325	3,08	1,18	8,09	0,02815479	0,909373673				
rs9698745	0,53	0,32	0,9	0,01431118	0,909373673				
rs12837275	5,03	1,65	15,28	0,00592699	0,884530262				
rs73186886	3,89	1,23	12,28	0,02609573	0,909373673				
rs73188618	0,25	0,06	1,05	0,02138507	0,909373673				
rs55909210	2,91	1,15	7,34	0,02674078	0,909373673				
rs114443125	2,83	1,08	7,42	0,03877582	0,909373673				

SNP	OR	lower	upper	p-value	FDR	Chromosome	gene name	gene source	Description
rs55958419	1,74	1,03	2,95	0,04118467	0,909373673				
rs5980048	13,88	1,38	139,73	0,01449489	0,909373673				
rs148770494	0,33	0,11	0,94	0,01705367	0,909373673				
rs56857867	0	0		0,03924402	0,909373673				
rs60957056	0,43	0,17	1,09	0,04680269	0,909373673				
rs142675995	0	0		0,03935464	0,909373673	X	GS1-519E5.1	Clone-based (Vega)	
rs2681642	1,6	1,02	2,52	0,04146341	0,909373673	X	TBL1X	HGNC Symbol	transducin (beta)-like 1X-linked [Source:HGNC Symbol;Acc:11585]
rs5934690	0,56	0,31	1,01	0,04426724	0,909373673	X	SHROOM2; RP11-98L4.1	HGNC Symbol;Clone-based	shroom family member 2 [Source:HGNC Symbol;Acc:630];
rs5978399	2,76	1,24	6,16	0,01636415	0,909373673				
rs7054879	2,48	1,13	5,45	0,02926036	0,909373673				
rs147699219	0,35	0,12	1,02	0,03063165	0,909373673	X	MID1	HGNC Symbol	midline 1 (Opitz/BBB syndrome) [Source:HGNC Symbol;Acc:7095]
rs974582	2,24	1,1	4,56	0,03154622	0,909373673	X	MID1	HGNC Symbol	midline 1 (Opitz/BBB syndrome) [Source:HGNC Symbol;Acc:7095]
rs5935088	0	0		0,01169228	0,909373673	X	ARHGAP6	HGNC Symbol	Rho GTPase activating protein 6 [Source:HGNC Symbol;Acc:676]
rs5978438	0,54	0,31	0,94	0,02149397	0,909373673	X	ARHGAP6	HGNC Symbol	Rho GTPase activating protein 6 [Source:HGNC Symbol;Acc:676]

SNP	OR	lower	upper	p-value	FDR	Chromosome	gene name	gene source	Description
rs7350366	0,55	0,33	0,94	0,02196289	0,909373673	X	ARHGAP6	HGNC Symbol	Rho GTPase activating protein 6 [Source:HGNC Symbol;Acc:676]
rs6654942	0,54	0,32	0,94	0,02165793	0,909373673	X	ARHGAP6	HGNC Symbol	Rho GTPase activating protein 6 [Source:HGNC Symbol;Acc:676]
rs66948843	1,7	1,02	2,83	0,04446465	0,909373673	X	FRMPD4	HGNC Symbol	FERM and PDZ domain containing 4 [Source:HGNC Symbol;Acc:29007]
rs112046747	0,3	0,1	0,87	0,01029134	0,900996292	X	FRMPD4	HGNC Symbol	FERM and PDZ domain containing 4 [Source:HGNC Symbol;Acc:29007]
rs62588580	0	0		0,01728748	0,909373673	X	FRMPD4	HGNC Symbol	FERM and PDZ domain containing 4 [Source:HGNC Symbol;Acc:29007]
rs147532402	0,22	0,05	0,95	0,012576	0,909373673	X	FRMPD4	HGNC Symbol	FERM and PDZ domain containing 4 [Source:HGNC Symbol;Acc:29007]
rs190831088	0,29	0,09	0,99	0,02330736	0,909373673	X	FRMPD4	HGNC Symbol	FERM and PDZ domain containing 4 [Source:HGNC Symbol;Acc:29007]
rs11095580	1,86	1,04	3,35	0,04180128	0,909373673	X	FRMPD4	HGNC Symbol	FERM and PDZ domain containing 4 [Source:HGNC Symbol;Acc:29007]
rs12557959	0,53	0,28	1,01	0,04001736	0,909373673	X	FRMPD4	HGNC Symbol	FERM and PDZ domain containing 4 [Source:HGNC Symbol;Acc:29007]
rs1731480	0	0		0,04862814	0,909373673				
rs1266360	0,49	0,24	1,02	0,03919479	0,909373673				
rs4639691	1,74	1,07	2,81	0,02472741	0,909373673	X	PRPS2	HGNC Symbol	phosphoribosyl pyrophosphate synthetase 2 [Source:HGNC Symbol;Acc:9465]
rs1266349	4,31	1,42	13,1	0,0125874	0,909373673	X	PRPS2	HGNC Symbol	phosphoribosyl pyrophosphate synthetase 2 [Source:HGNC Symbol;Acc:9465]
rs5979746	0,56	0,31	0,98	0,03507335	0,909373673				

SNP	OR	lower	upper	p-value	FDR	Chromosome	gene name	gene source	Description
rs5743740	2,61	1,11	6,12	0,03376442	0,909373673	X	TLR7	HGNC Symbol	toll-like receptor 7 [Source:HGNC Symbol;Acc:15631]
rs179009	0,55	0,3	1,02	0,04672546	0,909373673	X	TLR7	HGNC Symbol	toll-like receptor 7 [Source:HGNC Symbol;Acc:15631]
rs5979785	1,76	1,09	2,82	0,01964177	0,909373673				
rs6526375	3,96	1,35	11,64	0,01595628	0,909373673				
rs12834438	0,28	0,07	1,14	0,02775941	0,909373673	X	GS1-600G8.5	Clone-based (Vega)	
rs5979882	0,25	0,06	1,07	0,02352056	0,909373673				
rs113081319	0	0		0,03957719	0,909373673				
rs1431726	0,41	0,18	0,94	0,02030551	0,909373673				
rs73193934	0	0		0,02444964	0,909373673				
rs113747363	0,17	0,02	1,29	0,02170165	0,909373673				
rs73195762	0,27	0,07	1,11	0,02399225	0,909373673				
rs6654096	1,56	1	2,43	0,04658134	0,909373673	X	GPM6B	HGNC Symbol	glycoprotein M6B [Source:HGNC Symbol;Acc:4461]
rs66560303	1,76	1,12	2,78	0,01407003	0,909373673	X	GPM6B	HGNC Symbol	glycoprotein M6B [Source:HGNC Symbol;Acc:4461]
rs11798993	2,54	1,21	5,33	0,01701824	0,909373673				

SNP	OR	lower	upper	p-value	FDR	Chromosome	gene name	gene source	Description
rs7050970	0	0		0,04293148	0,909373673				
rs1989844	1,71	1,12	2,61	0,0124203	0,909373673				
rs73199669	0	0		0,03880512	0,909373673	X	GLRA2	HGNC Symbol	glycine receptor, alpha 2 [Source:HGNC Symbol;Acc:4327]
rs41305211	0	0		0,02324695	0,909373673	X	MOSPD2	HGNC Symbol	motile sperm domain containing 2 [Source:HGNC Symbol;Acc:28381]
rs17216162	1,89	1,02	3,5	0,04956244	0,910168444				
rs73189523	0,19	0,03	1,48	0,04434019	0,909373673	X	ASB9	HGNC Symbol	ankyrin repeat and SOCS box containing 9 [Source:HGNC Symbol;Acc:17184]
rs5935984	1,68	1,05	2,68	0,03082714	0,909373673	X	PIR;BMX	HGNC Symbol	pirin (iron-binding nuclear protein) [Source:HGNC Symbol;Acc:30048];BMX non-receptor tyrosine kinase [Source:HGNC Symbol;Acc:1079]
rs35803318	3	1,37	6,57	0,00785076	0,89300867	X	ACE2	HGNC Symbol	angiotensin I converting enzyme 2 [Source:HGNC Symbol;Acc:13557]
rs5980180	1,59	1,02	2,48	0,03933101	0,909373673	X	CA5B	HGNC Symbol	carbonic anhydrase VB, mitochondrial [Source:HGNC Symbol;Acc:1378]
rs5936081	0,58	0,33	1,01	0,04301876	0,909373673				
rs1024459	0,56	0,32	0,99	0,03454776	0,909373673				
rs12006589	2,25	1,05	4,83	0,04471496	0,909373673				
rs62585413	0,19	0,02	1,44	0,04056082	0,909373673				
rs4831035	0,61	0,39	0,97	0,03240803	0,909373673				

SNP	OR	lower	upper	p-value	FDR	Chromosome	gene name	gene source	Description
rs73636638	0,27	0,06	1,16	0,03570619	0,909373673				
rs73189179	2,88	1,13	7,39	0,0350743	0,909373673	X	RP3-410B11.1	Clone-based (Vega)	
rs145926052	2,3	1,05	5,02	0,04352332	0,909373673	X	SCML2	HGNC Symbol	sex comb on midleg-like 2 (Drosophila) [Source:HGNC Symbol;Acc:10581]
rs239746	0,39	0,18	0,87	0,00911549	0,89300867				
rs16980924	2,93	1,07	8,04	0,04616281	0,909373673	X	PHKA2	HGNC Symbol	phosphorylase kinase, alpha 2 (liver) [Source:HGNC Symbol;Acc:8926]
rs60594383	0,49	0,24	0,97	0,02939371	0,909373673	X	GPR64	HGNC Symbol	G protein-coupled receptor 64 [Source:HGNC Symbol;Acc:4516]
rs41304719	0	0		0,01896789	0,909373673	X	GPR64	HGNC Symbol	G protein-coupled receptor 64 [Source:HGNC Symbol;Acc:4516]
rs5955735	0	0		0,0130326	0,909373673				
rs10218356	0,29	0,07	1,2	0,03935323	0,909373673				
rs7065106	0,29	0,07	1,23	0,04394289	0,909373673				
rs7890403	0,13	0,02	0,97	0,00505843	0,873550665				
rs7059647	0,33	0,12	0,94	0,01567419	0,909373673				
rs4969754	0,41	0,16	1,04	0,03391491	0,909373673				
rs5950391	0,41	0,16	1,09	0,04829471	0,909373673				

SNP	OR	lower	upper	p-value	FDR	Chromosome	gene name	gene source	Description
rs5990977	0,47	0,24	0,96	0,0247708	0,909373673				
rs73635509	0,52	0,27	1,01	0,03840555	0,909373673				
rs73197282	2,44	1,29	4,63	0,00725909	0,89300867				
rs62590551	0,38	0,13	1,09	0,04348429	0,909373673	X	CNKSR2	HGNC Symbol	connector enhancer of kinase suppressor of Ras 2 [Source:HGNC Symbol;Acc:19701]
rs140079749	0,34	0,1	1,14	0,04290906	0,909373673				
rs35657111	0,18	0,02	1,38	0,03369582	0,909373673	X	PHEX	HGNC Symbol	phosphate regulating endopeptidase homolog, X-linked [Source:HGNC Symbol;Acc:8918]
rs73201130	6,06	1,54	23,8	0,01160089	0,909373673	X	PHEX	HGNC Symbol	phosphate regulating endopeptidase homolog, X-linked [Source:HGNC Symbol;Acc:8918]
rs2071585	0,55	0,31	0,99	0,03673052	0,909373673	X	PHEX	HGNC Symbol	phosphate regulating endopeptidase homolog, X-linked [Source:HGNC Symbol;Acc:8918]
rs6528091	0,55	0,31	0,99	0,03629898	0,909373673	X	PHEX	HGNC Symbol	phosphate regulating endopeptidase homolog, X-linked [Source:HGNC Symbol;Acc:8918]
rs7891648	0,42	0,17	1,03	0,03805135	0,909373673	X	RP11-40F8.2	Clone-based (Vega)	
rs2214521	0,55	0,3	1,02	0,04636733	0,909373673	X	RP11-40F8.2;GS1-433O24.1	Clone-based (Vega)	
rs12556950	0,63	0,4	0,99	0,03933463	0,909373673	X	RP11-40F8.2	Clone-based (Vega)	
rs5970898	1,7	1,05	2,75	0,03482842	0,909373673	X	RP11-40F8.2	Clone-based (Vega)	
rs66476440	0,54	0,29	1,02	0,04797656	0,909373673	X	RP11-40F8.2	Clone-based (Vega)	

SNP	OR	lower	upper	p-value	FDR	Chromosome	gene name	gene source	Description
rs5926152	2,13	1,25	3,63	0,00580151	0,884530262	X	RP11-40F8.2	Clone-based (Vega)	
rs56201502	1,84	1,08	3,12	0,02679943	0,909373673	X	RP11-40F8.2	Clone-based (Vega)	
rs4828955	0	0		0,04183498	0,909373673	X	RP11-40F8.2	Clone-based (Vega)	
rs5970980	0,36	0,14	0,89	0,01016596	0,900996292	X	RP11-40F8.2	Clone-based (Vega)	
rs5926231	2,38	1,11	5,08	0,03015929	0,909373673	X	RP11-40F8.2	Clone-based (Vega)	
rs5971044	0	0		0,04621816	0,909373673	X	RP11-40F8.2	Clone-based (Vega)	
rs6629611	0,44	0,2	0,98	0,02576445	0,909373673	X	RP11-40F8.2	Clone-based (Vega)	
rs757478	0,42	0,18	1	0,0302145	0,909373673				
rs17332584	0,49	0,23	1,04	0,04810798	0,909373673				
rs7052177	0,32	0,13	0,82	0,00561356	0,884530262	X	PTCHD1	HGNC Symbol	patched domain containing 1 [Source:HGNC Symbol;Acc:26392]
rs2202958	2,83	1,31	6,11	0,00961636	0,900996292				
rs545426401	0,19	0,03	1,48	0,0447721	0,909373673				
rs58988588	0,19	0,02	1,49	0,04428277	0,909373673	X	PDK3	HGNC Symbol	pyruvate dehydrogenase kinase, isozyme 3 [Source:HGNC Symbol;Acc:8811]
rs5986370	0,42	0,17	1,08	0,04346401	0,909373673				

SNP	OR	lower	upper	p-value	FDR	Chromosome	gene name	gene source	Description
rs151043588	0,14	0,02	1,08	0,01239026	0,909373673				
rs12007793	0,35	0,11	1,1	0,03382945	0,909373673				
rs1972978	0,61	0,37	1,01	0,0484751	0,909373673	X	RP11-268G12.3	Clone-based (Vega)	
rs2197785	1,71	1,08	2,7	0,02232373	0,909373673	X	RP11-268G12.1	Clone-based (Vega)	
rs7055337	0,61	0,37	1,01	0,0484751	0,909373673	X	RP11-268G12.1	Clone-based (Vega)	
rs55780661	0	0		0,03077105	0,909373673				
rs4534271	0,59	0,37	0,95	0,02410391	0,909373673				
rs6526672	0,31	0,13	0,76	0,00358124	0,873550665				
rs5926450	0,56	0,34	0,9	0,01362073	0,909373673				
rs221385	0,54	0,34	0,86	0,0079202	0,89300867				
rs5926847	0,55	0,34	0,88	0,01062354	0,900996292	X	DCAF8L2	HGNC Symbol	DDB1 and CUL4 associated factor 8-like 2 [Source:HGNC Symbol;Acc:31811]
rs5971429	0,47	0,29	0,76	0,00143547	0,873550665	X	DCAF8L2	HGNC Symbol	DDB1 and CUL4 associated factor 8-like 2 [Source:HGNC Symbol;Acc:31811]
rs3905591	0,45	0,28	0,75	0,00120162	0,873550665	X	DCAF8L2	HGNC Symbol	DDB1 and CUL4 associated factor 8-like 2 [Source:HGNC Symbol;Acc:31811]
rs5926888	1,81	1,14	2,88	0,01078745	0,901191224	X	DCAF8L2	HGNC Symbol	DDB1 and CUL4 associated factor 8-like 2 [Source:HGNC Symbol;Acc:31811]

SNP	OR	lower	upper	p-value	FDR	Chromosome	gene name	gene source	Description
rs5926892	0,44	0,24	0,78	0,0030947	0,873550665	X	DCAF8L2	HGNC Symbol	DDB1 and CUL4 associated factor 8-like 2 [Source:HGNC Symbol;Acc:31811]
rs5926895	0,52	0,31	0,86	0,00801779	0,89300867	X	DCAF8L2;AC 107613.1	HGNC Symbol;Cl one-based	DDB1 and CUL4 associated factor 8-like 2 [Source:HGNC Symbol;Acc:31811];
rs45553337	0,51	0,28	0,92	0,0197791	0,909373673	X	DCAF8L2;AC 107613.1	HGNC Symbol;Cl one-based	DDB1 and CUL4 associated factor 8-like 2 [Source:HGNC Symbol;Acc:31811];
rs7891169	1,58	1	2,5	0,0474468	0,909373673				
rs1368769	1,62	1,02	2,58	0,04105573	0,909373673	X	MAGEB10	HGNC Symbol	melanoma antigen family B, 10 [Source:HGNC Symbol;Acc:25377]
rs5926503	1,59	1	2,53	0,04768505	0,909373673				
rs5926917	3,05	1,33	6,96	0,00988462	0,900996292				
rs5927223	1,8	1,13	2,87	0,0131188	0,909373673	X	IL1RAPL1	HGNC Symbol	interleukin 1 receptor accessory protein-like 1 [Source:HGNC Symbol;Acc:5996]
rs12859816	0	0		0,03703116	0,909373673	X	IL1RAPL1	HGNC Symbol	interleukin 1 receptor accessory protein-like 1 [Source:HGNC Symbol;Acc:5996]
rs73221614	0,36	0,16	0,79	0,00414182	0,873550665	X	IL1RAPL1	HGNC Symbol	interleukin 1 receptor accessory protein-like 1 [Source:HGNC Symbol;Acc:5996]
rs1344448	0	0		0,03080258	0,909373673	X	IL1RAPL1	HGNC Symbol	interleukin 1 receptor accessory protein-like 1 [Source:HGNC Symbol;Acc:5996]
rs144057297	0	0		0,03745122	0,909373673				
rs62589084	0	0		0,01055449	0,900996292				
rs2864927	0,56	0,35	0,89	0,01185993	0,909373673				

SNP	OR	lower	upper	p-value	FDR	Chromosome	gene name	gene source	Description
rs4829424	0,45	0,23	0,89	0,01509138	0,909373673				
rs73205886	3,24	1,43	7,35	0,00565882	0,884530262				
rs73205899	2,39	1,06	5,39	0,04314786	0,909373673				
rs12860337	1,68	1,02	2,76	0,04200443	0,909373673	X	TAB3	HGNC Symbol	TGF-beta activated kinase 1/MAP3K7 binding protein 3 [Source:HGNC Symbol;Acc:30681]
rs6631216	0,51	0,26	1	0,03613444	0,909373673	X	TAB3	HGNC Symbol	TGF-beta activated kinase 1/MAP3K7 binding protein 3 [Source:HGNC Symbol;Acc:30681]
rs5972275	0,55	0,31	0,98	0,03270574	0,909373673	X	TAB3	HGNC Symbol	TGF-beta activated kinase 1/MAP3K7 binding protein 3 [Source:HGNC Symbol;Acc:30681]
rs2404151	0,46	0,24	0,89	0,01289702	0,909373673				
rs139539984	0	0		0,01431354	0,909373673	X	DMD	HGNC Symbol	dystrophin [Source:HGNC Symbol;Acc:2928]
rs5927747	0	0		0,02232142	0,909373673	X	DMD	HGNC Symbol	dystrophin [Source:HGNC Symbol;Acc:2928]
rs1800278	0	0		0,04605661	0,909373673	X	DMD	HGNC Symbol	dystrophin [Source:HGNC Symbol;Acc:2928]
rs72466572	0	0		0,03675563	0,909373673	X	DMD	HGNC Symbol	dystrophin [Source:HGNC Symbol;Acc:2928]
rs2606663	3,96	1,15	13,7	0,03732708	0,909373673	X	DMD	HGNC Symbol	dystrophin [Source:HGNC Symbol;Acc:2928]
rs2606665	0	0		0,00174734	0,873550665	X	DMD	HGNC Symbol	dystrophin [Source:HGNC Symbol;Acc:2928]
rs73221139	6,79	2,01	22,95	0,00224258	0,873550665	X	DMD	HGNC Symbol	dystrophin [Source:HGNC Symbol;Acc:2928]

SNP	OR	lower	upper	p-value	FDR	Chromosome	gene name	gene source	Description
rs16990375	0,49	0,24	1,01	0,03672376	0,909373673	X	DMD	HGNC Symbol	dystrophin [Source:HGNC Symbol;Acc:2928]
rs5972580	0	0		0,01040966	0,900996292	X	DMD	HGNC Symbol	dystrophin [Source:HGNC Symbol;Acc:2928]
rs5972586	0	0		0,01040966	0,900996292	X	DMD	HGNC Symbol	dystrophin [Source:HGNC Symbol;Acc:2928]
rs1158629	0,13	0,02	0,95	0,00544936	0,884530262	X	DMD	HGNC Symbol	dystrophin [Source:HGNC Symbol;Acc:2928]
rs5928080	1,61	1,01	2,56	0,0416898	0,909373673	X	DMD	HGNC Symbol	dystrophin [Source:HGNC Symbol;Acc:2928]
rs73621844	3,76	1,08	13,12	0,04565291	0,909373673	X	DMD	HGNC Symbol	dystrophin [Source:HGNC Symbol;Acc:2928]
rs7879662	0,46	0,26	0,82	0,00510671	0,873550665	X	DMD	HGNC Symbol	dystrophin [Source:HGNC Symbol;Acc:2928]
rs5927113	2,25	1,16	4,34	0,01846681	0,909373673	X	DMD	HGNC Symbol	dystrophin [Source:HGNC Symbol;Acc:2928]
rs143027254	0,31	0,09	0,99	0,01888273	0,909373673	X	DMD	HGNC Symbol	dystrophin [Source:HGNC Symbol;Acc:2928]
rs5971696	0	0		0,00561215	0,884530262	X	DMD	HGNC Symbol	dystrophin [Source:HGNC Symbol;Acc:2928]
rs12559939	0,27	0,1	0,68	0,00102473	0,873550665	X	DMD	HGNC Symbol	dystrophin [Source:HGNC Symbol;Acc:2928]
rs141927233	0,31	0,12	0,77	0,00306905	0,873550665	X	DMD	HGNC Symbol	dystrophin [Source:HGNC Symbol;Acc:2928]
rs73623943	0,46	0,24	0,9	0,01405344	0,909373673	X	DMD	HGNC Symbol	dystrophin [Source:HGNC Symbol;Acc:2928]
rs112512284	6,47	2,25	18,6	0,00062411	0,873550665	X	TMEM47	HGNC Symbol	transmembrane protein 47 [Source:HGNC Symbol;Acc:18515]

SNP	OR	lower	upper	p-value	FDR	Chromosome	gene name	gene source	Description
rs3109864	0,33	0,11	0,93	0,01530725	0,909373673				
rs12396884	0,31	0,11	0,89	0,01130077	0,909373673				
rs150433532	2,77	1,13	6,77	0,03227169	0,909373673				
rs150121548	0,35	0,12	0,97	0,02017746	0,909373673				
rs12391979	3,16	1,17	8,56	0,02817093	0,909373673				
rs11798799	2,94	1,31	6,6	0,01041279	0,900996292	X	CXorf22	HGNC Symbol	chromosome X open reading frame 22 [Source:HGNC Symbol;Acc:28546]
rs73203147	0,41	0,16	1,05	0,03661944	0,909373673				
rs28438823	0	0		0,00718261	0,89300867				
rs993441	1,93	1,13	3,3	0,01857701	0,909373673	X	TM4SF2;AL121578.2	UniProtKB Gene Name;Clo	Uncharacterized protein; cDNA FLJ59144, highly similar to Tetraspanin-7 [Source:UniProtKB/TrEMBL;Acc:B4E171];
rs5918419	1,66	1,02	2,7	0,04706435	0,909373673	X	TM4SF2;AL121578.2	UniProtKB Gene Name;Clo	Uncharacterized protein; cDNA FLJ59144, highly similar to Tetraspanin-7 [Source:UniProtKB/TrEMBL;Acc:B4E171];
rs58807730	2,36	1,05	5,31	0,045948	0,909373673	X	TM4SF2	UniProtKB Gene Name	Uncharacterized protein; cDNA FLJ59144, highly similar to Tetraspanin-7 [Source:UniProtKB/TrEMBL;Acc:B4E171]
rs6609582	1,59	1,02	2,49	0,04043518	0,909373673	X	TM4SF2	UniProtKB Gene Name	Uncharacterized protein; cDNA FLJ59144, highly similar to Tetraspanin-7 [Source:UniProtKB/TrEMBL;Acc:B4E171]
rs17246924	0,39	0,2	0,76	0,00242289	0,873550665	X	OTC;TM4SF2	HGNC Symbol;UniProtKB	ornithine carbamoyltransferase [Source:HGNC Symbol;Acc:8512];Uncharacterized protein; cDNA FLJ59144, highly similar to Tetraspanin-7
rs11795872	0,21	0,03	1,55	0,048782	0,909373673				

SNP	OR	lower	upper	p-value	FDR	Chromosome	gene name	gene source	Description
rs149340726	0	0		0,02495036	0,909373673				
rs5963578	0,61	0,38	0,98	0,03633754	0,909373673				
rs73205021	0,25	0,06	1,06	0,02016403	0,909373673	X	RP11-265P11.1	Clone-based (Vega)	
rs5917783	0,17	0,02	1,28	0,02271971	0,909373673	X	RP11-265P11.2	Clone-based (Vega)	
rs958398	0	0		0,04909266	0,909373673				
rs11796466	0	0		0,04393548	0,909373673	X	RP11-157D23.2	Clone-based (Vega)	
rs5963663	1,63	1,06	2,52	0,02594216	0,909373673				
rs17274776	0	0		0,01487221	0,909373673				
rs4589010	1,57	1	2,46	0,0498452	0,913553229				
rs6610294	1,73	1,04	2,88	0,03706167	0,909373673				
rs5963671	0	0		0,01604534	0,909373673				
rs138436947	0,23	0,05	1,01	0,01886058	0,909373673				
rs5963155	0,39	0,14	1,12	0,04850352	0,909373673	X	BCOR	HGNC Symbol	BCL6 corepressor [Source:HGNC Symbol;Acc:20893]
rs7055988	1,91	1,04	3,5	0,04173397	0,909373673				

SNP	OR	lower	upper	p-value	FDR	Chromosome	gene name	gene source	Description
rs113395359	2,62	1,27	5,42	0,01168858	0,909373673				
rs3008956	0,18	0,03	1,34	0,02377116	0,909373673				
rs35235436	1,88	1,05	3,37	0,03514153	0,909373673				
rs6610530	0,51	0,28	0,92	0,01733435	0,909373673				
rs6610538	0,4	0,2	0,77	0,00296422	0,873550665				
rs873336	1,93	1,09	3,42	0,02716977	0,909373673	X	NYX	HGNC Symbol	nyctalopin [Source:HGNC Symbol;Acc:8082]
rs5917441	2,55	1,16	5,61	0,02275395	0,909373673	X	CASK	HGNC Symbol	calcium/calmodulin-dependent serine protein kinase (MAGUK family) [Source:HGNC Symbol;Acc:1497]
rs147839261	0	0		0,02029979	0,909373673	X	CASK	HGNC Symbol	calcium/calmodulin-dependent serine protein kinase (MAGUK family) [Source:HGNC Symbol;Acc:1497]
rs5964067	0	0		0,00737938	0,89300867				
rs707440	0	0		0,00381689	0,873550665				
rs17146289	4,74	1,67	13,42	0,00455927	0,873550665	X	RP1-154K9.2	Clone-based (Vega)	
rs138493039	0,19	0,03	1,43	0,03511286	0,909373673				
rs11266342	0,13	0,02	0,95	0,00499577	0,873550665				
rs12851446	5,39	1,84	15,77	0,0026696	0,873550665				

SNP	OR	lower	upper	p-value	FDR	Chromosome	gene name	gene source	Description
rs146896335	3,33	1,55	7,17	0,00296903	0,873550665				
rs2213606	0,24	0,06	1,07	0,02405988	0,909373673				
rs73210281	0	0		0,02571598	0,909373673				
rs73212025	6,47	1,05	40,03	0,04561135	0,909373673				
rs140826571	0	0		0,03644893	0,909373673				
rs73208986	4,54	1,09	18,96	0,04534309	0,909373673				
rs145269866	0	0		0,04651965	0,909373673				
rs142677545	3,16	1,21	8,28	0,02487297	0,909373673	X	MAOA	HGNC Symbol	monoamine oxidase A [Source:HGNC Symbol;Acc:6833]
rs3027450	1,76	1,08	2,87	0,02455152	0,909373673	X	MAOB	HGNC Symbol	monoamine oxidase B [Source:HGNC Symbol;Acc:6834]
rs140925586	3,71	1,38	9,99	0,01281276	0,909373673	X	EFHC2	HGNC Symbol	EF-hand domain (C-terminal) containing 2 [Source:HGNC Symbol;Acc:26233]
rs5906072	0,5	0,24	1,02	0,04323367	0,909373673				
rs5906076	0,47	0,23	0,93	0,02152221	0,909373673				
rs6521042	0,46	0,28	0,75	0,00103547	0,873550665				
rs5906083	0,51	0,31	0,82	0,00419143	0,873550665				

SNP	OR	lower	upper	p-value	FDR	Chromosome	gene name	gene source	Description
rs2009184	0,47	0,29	0,77	0,00175059	0,873550665				
rs7058787	0,44	0,26	0,74	0,00105061	0,873550665				
rs5906093	0,6	0,38	0,93	0,02077758	0,909373673				
rs5952332	0,62	0,39	0,98	0,03729368	0,909373673				
rs28445915	1,76	1,11	2,81	0,01682478	0,909373673	X	SLC9A7	HGNC Symbol	solute carrier family 9, subfamily A (NHE7, cation proton antiporter 7), member 7 [Source:HGNC Symbol;Acc:17123]
rs1805147	3,32	1,19	9,27	0,02451044	0,909373673	X;HG29_PATCH	RP2	HGNC Symbol	retinitis pigmentosa 2 (X-linked recessive) [Source:HGNC Symbol;Acc:10274]
rs12560179	2,29	1,16	4,52	0,02040599	0,909373673	X	JADE3	HGNC Symbol	jade family PHD finger 3 [Source:HGNC Symbol;Acc:22982]
rs73201990	0,17	0,02	1,29	0,02639108	0,909373673				
rs139127466	0,17	0,02	1,29	0,02340007	0,909373673				
rs73204096	6,5	1,05	40,12	0,04471411	0,909373673				
rs183846665	0	0		0,02837427	0,909373673	X;HG1436_HG1432_PATCH	ZNF81	HGNC Symbol	zinc finger protein 81 [Source:HGNC Symbol;Acc:13156]
rs491610	0,19	0,02	1,44	0,04023447	0,909373673	X;HG1436_HG1432_PATCH	ZNF630;ZNF630-AS1	HGNC Symbol	zinc finger protein 630 [Source:HGNC Symbol;Acc:28855];ZNF630 antisense RNA 1 [Source:HGNC Symbol;Acc:41215]
rs5905692	0,53	0,29	0,97	0,02904447	0,909373673	X;HG1436_HG1432_PATCH	FTSJ1	HGNC Symbol	FtsJ RNA methyltransferase homolog 1 (E. coli) [Source:HGNC Symbol;Acc:13254]
rs5906710	0	0		0,02072608	0,909373673				

SNP	OR	lower	upper	p-value	FDR	Chromosome	gene name	gene source	Description
rs73199936	0	0		0,03178607	0,909373673	X;HG1433_PATCH	SHROOM4	HGNC Symbol	shroom family member 4 [Source:HGNC Symbol;Acc:29215]
rs112296322	0,5	0,25	1,01	0,03641233	0,909373673				
rs12850774	1,88	1,08	3,28	0,02861554	0,909373673				
rs1110404	1,99	1,18	3,36	0,01044729	0,900996292				
rs1936037	1,59	1,01	2,51	0,04653781	0,909373673				
rs12558898	1,76	1,11	2,79	0,01645421	0,909373673				
rs5951102	1,62	1,02	2,57	0,0425853	0,909373673				
rs150767800	2,03	1,19	3,45	0,00958205	0,900996292				
rs5991756	1,75	1,04	2,96	0,0381283	0,909373673				
rs4129866	1,75	1,04	2,96	0,0381283	0,909373673				
rs145117130	1,71	1,02	2,89	0,04474471	0,909373673				
rs141219156	1,78	1,06	2,98	0,03076487	0,909373673				
rs12394834	1,77	1,06	2,97	0,03164621	0,909373673				
rs139768820	0	0		0,01971557	0,909373673	X;HG1433_PATCH	GPR173;RP1-290F12.3	HGNC Symbol;Clone-based	G protein-coupled receptor 173 [Source:HGNC Symbol;Acc:18186];

SNP	OR	lower	upper	p-value	FDR	Chromosome	gene name	gene source	Description
rs5978144	0,24	0,05	1,07	0,02575543	0,909373673	X;HG1433_PATCH	KDM5C	HGNC Symbol	lysine (K)-specific demethylase 5C [Source:HGNC Symbol;Acc:11114]
rs41308616	0	0		0,03530917	0,909373673	X;HG1433_PATCH	KDM5C	HGNC Symbol	lysine (K)-specific demethylase 5C [Source:HGNC Symbol;Acc:11114]
rs17276442	0,27	0,06	1,17	0,03722994	0,909373673	X;HG1433_PATCH	IQSEC2	HGNC Symbol	IQ motif and Sec7 domain 2 [Source:HGNC Symbol;Acc:29059]
rs55998917	0	0		0,03098263	0,909373673	X;HG1433_PATCH	WNK3	HGNC Symbol	WNK lysine deficient protein kinase 3 [Source:HGNC Symbol;Acc:14543]
rs62618081	0	0		0,0251513	0,909373673	X	GNL3L	HGNC Symbol	guanine nucleotide binding protein-like 3 (nucleolar)-like [Source:HGNC Symbol;Acc:25553]
rs17251419	4,38	1,21	15,83	0,02950698	0,909373673				
rs17842839	0	0		0,02414619	0,909373673				
rs4462043	2,11	1,09	4,09	0,03142906	0,909373673				
rs1547338	1,76	1,03	2,99	0,03905091	0,909373673				
rs17301297	2,12	1,04	4,3	0,04435789	0,909373673	X	ZC4H2	HGNC Symbol	zinc finger, C4H2 domain containing [Source:HGNC Symbol;Acc:24931]
rs62610373	3,47	1,21	9,93	0,02591153	0,909373673				
rs17249650	2,18	1,05	4,53	0,04232044	0,909373673				
rs17216533	2,52	1,04	6,12	0,04959718	0,910168444				
rs111873599	2,05	1,05	4,02	0,04137459	0,909373673	X	HEPH	HGNC Symbol	hephaestin [Source:HGNC Symbol;Acc:4866]

SNP	OR	lower	upper	p-value	FDR	Chromosome	gene name	gene source	Description
rs112069404	2,01	1,12	3,62	0,02245534	0,909373673				
rs113989766	2,18	1,09	4,37	0,03247942	0,909373673				
rs62604342	2,96	1,08	8,17	0,04447369	0,909373673				
rs6625282	0,29	0,1	0,85	0,00886222	0,89300867	X	OPHN1	HGNC Symbol	oligophrenin 1 [Source:HGNC Symbol;Acc:8148]
rs5965519	0,16	0,04	0,68	0,00131597	0,873550665	X	OPHN1	HGNC Symbol	oligophrenin 1 [Source:HGNC Symbol;Acc:8148]
rs150294060	0,28	0,06	1,24	0,04934883	0,909373673	X	OPHN1	HGNC Symbol	oligophrenin 1 [Source:HGNC Symbol;Acc:8148]
rs11796608	2,68	1,05	6,86	0,04887614	0,909373673	X	OPHN1	HGNC Symbol	oligophrenin 1 [Source:HGNC Symbol;Acc:8148]
rs7886230	0,33	0,1	1,07	0,02979722	0,909373673	X	STARD8	HGNC Symbol	StAR-related lipid transfer (START) domain containing 8 [Source:HGNC Symbol;Acc:19161]
rs5980922	1,57	1	2,46	0,04703915	0,909373673				
rs66998265	0	0		0,0050638	0,873550665				
rs5936920	0,2	0,03	1,52	0,0494273	0,909373673				
rs877817	0,28	0,09	0,84	0,01023321	0,900996292	X	EFNB1	HGNC Symbol	ephrin-B1 [Source:HGNC Symbol;Acc:3226]
rs73543630	0	0		0,04843709	0,909373673				
rs11796961	0	0		0,0320245	0,909373673				

SNP	OR	lower	upper	p-value	FDR	Chromosome	gene name	gene source	Description
rs73528831	3,71	1,2	11,47	0,02708268	0,909373673				
rs17217584	0,38	0,13	1,1	0,04478662	0,909373673				
rs4844335	0,47	0,26	0,88	0,01075623	0,901191224				
rs1317200	1,77	1,1	2,84	0,01689914	0,909373673				
rs147121494	1,9	1,03	3,5	0,04702913	0,909373673	X	EDA	HGNC Symbol	ectodysplasin A [Source:HGNC Symbol;Acc:3157]
rs5936511	0,52	0,28	0,96	0,02836936	0,909373673	X	EDA	HGNC Symbol	ectodysplasin A [Source:HGNC Symbol;Acc:3157]
rs1938029	0,53	0,27	1,02	0,04633934	0,909373673	X	EDA	HGNC Symbol	ectodysplasin A [Source:HGNC Symbol;Acc:3157]
rs35407838	2	1,16	3,46	0,01494491	0,909373673	X	EDA	HGNC Symbol	ectodysplasin A [Source:HGNC Symbol;Acc:3157]
rs5936806	1,66	1,06	2,6	0,02744726	0,909373673	X	EDA	HGNC Symbol	ectodysplasin A [Source:HGNC Symbol;Acc:3157]
rs73226452	0	0		0,00784247	0,89300867	X	EDA	HGNC Symbol	ectodysplasin A [Source:HGNC Symbol;Acc:3157]
rs6625563	0,6	0,37	0,97	0,03303744	0,909373673	X	EDA	HGNC Symbol	ectodysplasin A [Source:HGNC Symbol;Acc:3157]
rs111714615	2,37	1,27	4,41	0,00764454	0,89300867	X	DGAT2L6	HGNC Symbol	diacylglycerol O-acyltransferase 2-like 6 [Source:HGNC Symbol;Acc:23250]
rs145495213	3,72	1,07	12,95	0,04734596	0,909373673	X	KIF4A	HGNC Symbol	kinesin family member 4A [Source:HGNC Symbol;Acc:13339]
rs5936905	0	0		0,02112867	0,909373673				

SNP	OR	lower	upper	p-value	FDR	Chromosome	gene name	gene source	Description
rs11796215	0	0		0,00822633	0,89300867	X	OGT	HGNC Symbol	O-linked N-acetylglucosamine (GlcNAc) transferase [Source:HGNC Symbol;Acc:8127]
rs149580659	3,24	1,22	8,64	0,02363201	0,909373673	X	ACRC	HGNC Symbol	acidic repeat containing [Source:HGNC Symbol;Acc:15805]
rs4986604	0,19	0,02	1,45	0,04136857	0,909373673				
rs138097931	2,16	1,23	3,82	0,00931355	0,900996292	X	NHSL2	HGNC Symbol	NHS-like 2 [Source:HGNC Symbol;Acc:33737]
rs59036635	0,14	0,02	1,11	0,0148911	0,909373673	X	NHSL2	HGNC Symbol	NHS-like 2 [Source:HGNC Symbol;Acc:33737]
rs62612130	2,94	1,22	7,12	0,02040627	0,909373673	X	NHSL2	HGNC Symbol	NHS-like 2 [Source:HGNC Symbol;Acc:33737]
rs73225129	0,39	0,18	0,85	0,00908837	0,89300867	X;HG1438_PATCH	HDAC8	HGNC Symbol	histone deacetylase 8 [Source:HGNC Symbol;Acc:13315]
rs62613008	2,88	1,46	5,7	0,00285836	0,873550665	X;HG1438_PATCH	PHKA1	HGNC Symbol	phosphorylase kinase, alpha 1 (muscle) [Source:HGNC Symbol;Acc:8925]
rs139180358	0,31	0,11	0,91	0,01420761	0,909373673				
rs62610693	1,95	1,02	3,72	0,049399	0,909373673				
rs147312656	0,25	0,06	1,08	0,02612298	0,909373673				
rs5937813	0	0		0,01922579	0,909373673	X	SLC16A2	HGNC Symbol	solute carrier family 16, member 2 (thyroid hormone transporter) [Source:HGNC Symbol;Acc:10923]
rs62612360	3,68	1,06	12,74	0,04857884	0,909373673	X	KIAA2022	HGNC Symbol	KIAA2022 [Source:HGNC Symbol;Acc:29433]
rs5981937	0	0		0,02697279	0,909373673				

SNP	OR	lower	upper	p-value	FDR	Chromosome	gene name	gene source	Description
rs17303393	1,95	1,08	3,51	0,0286213	0,909373673	X;HG1426_PATCH	ATP7A	HGNC Symbol	ATPase, Cu++ transporting, alpha polypeptide [Source:HGNC Symbol;Acc:869]
rs3943322	0,61	0,37	1,01	0,04643367	0,909373673				
rs12839556	1,66	1,04	2,64	0,03134044	0,909373673				
rs7064039	0,39	0,2	0,76	0,00295858	0,873550665				
rs12013328	1,68	1,01	2,81	0,04615911	0,909373673				
rs2444580	0,48	0,22	1,05	0,04743602	0,909373673	X	RP13-52K8.1	Clone-based (Vega)	
rs12556404	0,32	0,1	1,06	0,03013142	0,909373673				
rs6622648	1,71	1,08	2,7	0,01948066	0,909373673				
rs1923010	0,44	0,2	0,96	0,02499057	0,909373673				
rs73230915	2,43	1,2	4,91	0,01621921	0,909373673				
rs150558137	3,26	1,17	9,13	0,03105273	0,909373673				
rs707675	1,83	1,06	3,14	0,03212917	0,909373673				
rs6623194	0	0		0,00646361	0,884530262				
rs5923933	0,49	0,23	1,04	0,04462779	0,909373673				

SNP	OR	lower	upper	p-value	FDR	Chromosome	gene name	gene source	Description
rs112304795	2,51	1,07	5,85	0,04073366	0,909373673	X	KLHL4	HGNC Symbol	kelch-like family member 4 [Source:HGNC Symbol;Acc:6355]
rs5924107	0,36	0,11	1,17	0,04849202	0,909373673				
rs7891751	2,6	1,09	6,2	0,03773831	0,909373673				
rs73232261	0	0		0,04640555	0,909373673				
rs5924181	0,34	0,13	0,87	0,00987373	0,900996292				
rs17253529	0,29	0,09	1	0,02346875	0,909373673				
rs137863336	4,52	1,25	16,38	0,02659296	0,909373673				
rs73234137	3,21	1,29	7,98	0,01403428	0,909373673				
rs5942473	1,59	1,03	2,45	0,03711011	0,909373673				
rs73636407	0	0		0,03483341	0,909373673				
rs2558769	0	0		0,047015	0,909373673				
rs149838623	0,61	0,37	0,98	0,03932866	0,909373673				
rs5941247	1,87	1,03	3,4	0,04598937	0,909373673				
rs73251336	1,98	1,09	3,6	0,02894487	0,909373673				

SNP	OR	lower	upper	p-value	FDR	Chromosome	gene name	gene source	Description
rs150957703	0,29	0,1	0,84	0,00773424	0,89300867				
rs73250402	2,59	1,29	5,21	0,00907366	0,89300867				
rs6618575	0,56	0,31	1,02	0,04565979	0,909373673				
rs12387371	0,56	0,34	0,93	0,02147088	0,909373673				
rs55674632	2,93	1,37	6,27	0,00704235	0,89300867				
rs145134624	3,05	1,4	6,63	0,00620408	0,884530262	X	PCDH11X	HGNC Symbol	protocadherin 11 X-linked [Source:HGNC Symbol;Acc:8656]
rs7891218	0,44	0,23	0,83	0,00713368	0,89300867	X	FAM133A	HGNC Symbol	family with sequence similarity 133, member A [Source:HGNC Symbol;Acc:26748]
rs73254374	0,48	0,27	0,85	0,00769739	0,89300867				
rs185837010	0,14	0,02	1,05	0,01028422	0,900996292				
rs66864266	0,57	0,34	0,95	0,02337732	0,909373673				
rs111314151	0	0		0,02968836	0,909373673				
rs73245118	2,5	1,06	5,91	0,04286271	0,909373673				
rs413169	1,53	1	2,32	0,04639817	0,909373673	X	DIAPH2	HGNC Symbol	diaphanous-related formin 2 [Source:HGNC Symbol;Acc:2877]
rs707287	0,63	0,4	0,98	0,03651596	0,909373673	X	DIAPH2	HGNC Symbol	diaphanous-related formin 2 [Source:HGNC Symbol;Acc:2877]

SNP	OR	lower	upper	p-value	FDR	Chromosome	gene name	gene source	Description
rs11796386	0,19	0,02	1,45	0,04171214	0,909373673	X	DIAPH2	HGNC Symbol	diaphanous-related formin 2 [Source:HGNC Symbol;Acc:2877]
rs138140181	0	0		0,03646403	0,909373673	X	DIAPH2-AS1	HGNC Symbol	DIAPH2 antisense RNA 1 [Source:HGNC Symbol;Acc:16972]
rs5967343	0,2	0,03	1,46	0,03291602	0,909373673				
rs6620351	0,5	0,26	0,94	0,020662	0,909373673				
rs6615988	0,58	0,35	0,98	0,0351825	0,909373673				
rs5921108	1,82	1,17	2,85	0,00806132	0,89300867				
rs6616015	1,69	1,08	2,66	0,02060665	0,909373673				
rs2473218	0,48	0,3	0,77	0,00166085	0,873550665				
rs16982961	0,29	0,11	0,76	0,00358962	0,873550665				
rs7889840	0,43	0,23	0,83	0,00634558	0,884530262				
rs4827953	0,42	0,24	0,72	0,00081341	0,873550665				
rs112879811	0,51	0,29	0,91	0,01701723	0,909373673				
rs16982964	3,01	1,15	7,87	0,03086782	0,909373673				
rs5921285	0,47	0,28	0,8	0,0035115	0,873550665				

SNP	OR	lower	upper	p-value	FDR	Chromosome	gene name	gene source	Description
rs5920732	0,4	0,15	1,03	0,03380562	0,909373673				
rs5966968	0,37	0,16	0,88	0,01102099	0,909373673	X	XRCC6P5	HGNC Symbol	X-ray repair complementing defective repair in Chinese hamster cells 6 pseudogene 5 [Source:HGNC Symbol;Acc:45187]
rs6616087	0,19	0,05	0,82	0,00406664	0,873550665	X	XRCC6P5	HGNC Symbol	X-ray repair complementing defective repair in Chinese hamster cells 6 pseudogene 5 [Source:HGNC Symbol;Acc:45187]
rs62614761	0,42	0,19	0,89	0,01305627	0,909373673				
rs73557954	0,15	0,02	1,17	0,01606406	0,909373673				
rs5967145	1,68	1,04	2,69	0,03211898	0,909373673				
rs34429223	0,23	0,05	1	0,01855702	0,909373673				
rs1343213	2,49	1,32	4,7	0,00572258	0,884530262	X	SRPX2	HGNC Symbol	sushi-repeat containing protein, X-linked 2 [Source:HGNC Symbol;Acc:30668]
rs5966722	6,24	1,33	29,21	0,02216031	0,909373673				
rs60097212	4,41	1,71	11,39	0,0022094	0,873550665	X	ARL13A	HGNC Symbol	ADP-ribosylation factor-like 13A [Source:HGNC Symbol;Acc:31709]
rs17257516	0,14	0,02	1,08	0,01185681	0,909373673	X	ARL13A	HGNC Symbol	ADP-ribosylation factor-like 13A [Source:HGNC Symbol;Acc:31709]
rs111917350	1,87	1,05	3,36	0,03964017	0,909373673	X	TRMT2B;TRMT2B-AS1	HGNC Symbol	tRNA methyltransferase 2 homolog B (S. cerevisiae) [Source:HGNC Symbol;Acc:25748];TRMT2B antisense RNA 1 [Source:HGNC Symbol;Acc:41116]
rs6621026	1,72	1,08	2,75	0,02302188	0,909373673	X	TAF7L	HGNC Symbol	TAF7-like RNA polymerase II, TATA box binding protein (TBP)-associated factor, 50kDa [Source:HGNC Symbol;Acc:11548]
rs2180271	0,35	0,2	0,64	0,00017865	0,873550665	X	TAF7L	HGNC Symbol	TAF7-like RNA polymerase II, TATA box binding protein (TBP)-associated factor, 50kDa [Source:HGNC Symbol;Acc:11548]

SNP	OR	lower	upper	p-value	FDR	Chromosome	gene name	gene source	Description
rs79990723	1,66	1,07	2,58	0,02397204	0,909373673				
rs2239462	0,42	0,19	0,92	0,01886065	0,909373673	X;HG1439_PATCH	BTK	HGNC Symbol	Bruton agammaglobulinemia tyrosine kinase [Source:HGNC Symbol;Acc:1133]
rs2239460	0,29	0,08	0,98	0,02191741	0,909373673	X	BTK	HGNC Symbol	Bruton agammaglobulinemia tyrosine kinase [Source:HGNC Symbol;Acc:1133]
rs2071225	0,19	0,03	1,36	0,02513553	0,909373673	X;HG1439_PATCH	GLA;RPL36A-HNRNPH2	HGNC Symbol	galactosidase, alpha [Source:HGNC Symbol;Acc:4296];RPL36A-HNRNPH2 readthrough [Source:HGNC Symbol;Acc:48349]
rs3027585	0	0		0,00238416	0,873550665	X;HG1439_PATCH	GLA;RPL36A-HNRNPH2	HGNC Symbol	galactosidase, alpha [Source:HGNC Symbol;Acc:4296];RPL36A-HNRNPH2 readthrough [Source:HGNC Symbol;Acc:48349]
rs5991904	0,3	0,12	0,77	0,00356636	0,873550665	X;HG1439_PATCH	ARMCX4	HGNC Symbol	armadillo repeat containing, X-linked 4 [Source:HGNC Symbol;Acc:28615]
rs61736018	0	0		0,00090046	0,873550665	X;HG1439_PATCH	ARMCX4	HGNC Symbol	armadillo repeat containing, X-linked 4 [Source:HGNC Symbol;Acc:28615]
rs6995	1,72	1,02	2,93	0,04555636	0,909373673	X;HG1439_PATCH	ARMCX3	HGNC Symbol	armadillo repeat containing, X-linked 3 [Source:HGNC Symbol;Acc:24065]
rs12687779	0,28	0,06	1,23	0,04641574	0,909373673				
rs17284970	0	0		0,00309824	0,873550665	X	LINC00630; MTND1P32	HGNC Symbol	long intergenic non-protein coding RNA 630 [Source:HGNC Symbol;Acc:44263];MT-ND1 pseudogene 32 [Source:HGNC Symbol;Acc:42081]
rs17285025	0,19	0,03	1,5	0,04749239	0,909373673				
rs150540333	0	0		0,03442867	0,909373673				
rs11545818	0,25	0,07	0,83	0,00701409	0,89300867	X	TCEAL4	HGNC Symbol	transcription elongation factor A (SII)-like 4 [Source:HGNC Symbol;Acc:26121]
rs521895	0,55	0,33	0,91	0,01506515	0,909373673	X	PLP1	HGNC Symbol	proteolipid protein 1 [Source:HGNC Symbol;Acc:9086]

SNP	OR	lower	upper	p-value	FDR	Chromosome	gene name	gene source	Description
rs5945830	1,6	1,01	2,52	0,04219079	0,909373673				
rs112217849	2,25	1,05	4,83	0,04287087	0,909373673	X	IL1RAPL2	HGNC Symbol	interleukin 1 receptor accessory protein-like 2 [Source:HGNC Symbol;Acc:5997]
rs112962026	1,97	1,13	3,43	0,01894438	0,909373673	X	IL1RAPL2	HGNC Symbol	interleukin 1 receptor accessory protein-like 2 [Source:HGNC Symbol;Acc:5997]
rs141124309	0	0		0,02315335	0,909373673	X	IL1RAPL2	HGNC Symbol	interleukin 1 receptor accessory protein-like 2 [Source:HGNC Symbol;Acc:5997]
rs17332218	0,17	0,02	1,28	0,02487323	0,909373673	X	IL1RAPL2	HGNC Symbol	interleukin 1 receptor accessory protein-like 2 [Source:HGNC Symbol;Acc:5997]
rs141365747	0	0		0,03794144	0,909373673	X;HG375_PATCH	IL1RAPL2	HGNC Symbol	interleukin 1 receptor accessory protein-like 2 [Source:HGNC Symbol;Acc:5997]
rs6616577	0,52	0,3	0,9	0,01323936	0,909373673	X;HG375_PATCH	IL1RAPL2	HGNC Symbol	interleukin 1 receptor accessory protein-like 2 [Source:HGNC Symbol;Acc:5997]
rs17332274	0,54	0,29	1	0,03727894	0,909373673	X	IL1RAPL2	HGNC Symbol	interleukin 1 receptor accessory protein-like 2 [Source:HGNC Symbol;Acc:5997]
rs5962292	1,77	1,11	2,8	0,01371931	0,909373673	X	IL1RAPL2	HGNC Symbol	interleukin 1 receptor accessory protein-like 2 [Source:HGNC Symbol;Acc:5997]
rs5916932	1,69	1,07	2,67	0,02310142	0,909373673	X	IL1RAPL2	HGNC Symbol	interleukin 1 receptor accessory protein-like 2 [Source:HGNC Symbol;Acc:5997]
rs5962556	1,79	1,13	2,82	0,01179832	0,909373673	X	IL1RAPL2	HGNC Symbol	interleukin 1 receptor accessory protein-like 2 [Source:HGNC Symbol;Acc:5997]
rs150891050	0	0		0,01981741	0,909373673	X	IL1RAPL2	HGNC Symbol	interleukin 1 receptor accessory protein-like 2 [Source:HGNC Symbol;Acc:5997]
rs5916936	1,74	1,1	2,77	0,0171981	0,909373673	X	IL1RAPL2	HGNC Symbol	interleukin 1 receptor accessory protein-like 2 [Source:HGNC Symbol;Acc:5997]
rs12557027	0,19	0,04	0,82	0,00597443	0,884530262	X	IL1RAPL2	HGNC Symbol	interleukin 1 receptor accessory protein-like 2 [Source:HGNC Symbol;Acc:5997]

SNP	OR	lower	upper	p-value	FDR	Chromosome	gene name	gene source	Description
rs209119	0,56	0,31	1	0,04245254	0,909373673				
rs11795816	0,19	0,04	0,82	0,00562622	0,884530262				
rs1298577	1,68	1,02	2,76	0,04214478	0,909373673	X	NUP62CL	HGNC Symbol	nucleoporin 62kDa C-terminal like [Source:HGNC Symbol;Acc:25960]
rs6622173	1,69	1,03	2,78	0,03942179	0,909373673	X	NUP62CL	HGNC Symbol	nucleoporin 62kDa C-terminal like [Source:HGNC Symbol;Acc:25960]
rs58958250	1,65	1,01	2,71	0,04769367	0,909373673				
rs17254207	1,93	1,22	3,07	0,00487663	0,873550665	X	TSC22D3	HGNC Symbol	TSC22 domain family, member 3 [Source:HGNC Symbol;Acc:3051]
rs5917070	1,82	1,17	2,85	0,00797884	0,89300867	X	NCBP2L	HGNC Symbol	nuclear cap binding protein subunit 2-like [Source:HGNC Symbol;Acc:31795]
rs2273051	0	0		0,04433308	0,909373673	X	COL4A5	HGNC Symbol	collagen, type IV, alpha 5 [Source:HGNC Symbol;Acc:2207]
rs73255611	0	0		0,02614592	0,909373673				
rs73255702	0	0		0,02614592	0,909373673				
rs7061716	0,19	0,02	1,42	0,03837135	0,909373673				
rs73261736	0	0		0,01596385	0,909373673				
rs5942780	0,2	0,03	1,51	0,04817113	0,909373673				
rs4893424	0	0		0,02687857	0,909373673				

SNP	OR	lower	upper	p-value	FDR	Chromosome	gene name	gene source	Description
rs41307405	0	0		0,04285865	0,909373673	X	ACSL4	HGNC Symbol	acyl-CoA synthetase long-chain family member 4 [Source:HGNC Symbol;Acc:3571]
rs55663048	1,79	1,11	2,89	0,02016946	0,909373673				
rs17254838	1,93	1,14	3,26	0,0149175	0,909373673	X	AMMECR1	HGNC Symbol	Alport syndrome, mental retardation, midface hypoplasia and elliptocytosis chromosomal region gene 1 [Source:HGNC Symbol;Acc:467]
rs5942909	1,61	1,04	2,47	0,03200425	0,909373673	X	AMMECR1	HGNC Symbol	Alport syndrome, mental retardation, midface hypoplasia and elliptocytosis chromosomal region gene 1 [Source:HGNC Symbol;Acc:467]
rs73250295	0,2	0,03	1,52	0,04925092	0,909373673	X	AMMECR1	HGNC Symbol	Alport syndrome, mental retardation, midface hypoplasia and elliptocytosis chromosomal region gene 1 [Source:HGNC Symbol;Acc:467]
rs146735686	0	0		0,03503179	0,909373673				
rs6642884	0	0		0,00802369	0,89300867	X	CAPN6	HGNC Symbol	calpain 6 [Source:HGNC Symbol;Acc:1483]
rs62613862	1,85	1,03	3,32	0,04505826	0,909373673				
rs16986618	0,14	0,02	1,01	0,00627328	0,884530262	X	ALG13	HGNC Symbol	ALG13, UDP-N-acetylglucosaminyltransferase subunit [Source:HGNC Symbol;Acc:30881]
rs10521536	0,29	0,07	1,19	0,03496841	0,909373673	X	TRPC5;TRPC5OS	HGNC Symbol	transient receptor potential cation channel, subfamily C, member 5 [Source:HGNC Symbol;Acc:12337];TRPC5 opposite strand [Source:HGNC Symbol;Acc:40593]
rs116763278	0,29	0,07	1,18	0,03437133	0,909373673	X	TRPC5;TRPC5OS	HGNC Symbol	transient receptor potential cation channel, subfamily C, member 5 [Source:HGNC Symbol;Acc:12337];TRPC5 opposite strand [Source:HGNC Symbol;Acc:40593]
rs17307746	0,17	0,02	1,29	0,02604795	0,909373673	X	TRPC5	HGNC Symbol	transient receptor potential cation channel, subfamily C, member 5 [Source:HGNC Symbol;Acc:12337]
rs5982530	0,38	0,18	0,81	0,00501549	0,873550665				
rs5982532	0,37	0,17	0,82	0,00495844	0,873550665				

SNP	OR	lower	upper	p-value	FDR	Chromosome	gene name	gene source	Description
rs138049801	2,19	1,09	4,4	0,03158316	0,909373673				
rs3125955	0,48	0,28	0,81	0,00409456	0,873550665				
rs2206156	0,6	0,37	0,98	0,03598004	0,909373673				
rs6642625	0,21	0,05	0,88	0,00611031	0,884530262				
rs142274380	2,2	1,05	4,58	0,0408653	0,909373673	X;HG1434_PATCH	RP5-964N17.1	Clone-based (Vega)	
rs5929190	0,34	0,12	0,99	0,02489685	0,909373673	X;HG1434_PATCH	RP5-964N17.1	Clone-based (Vega)	
rs73219446	12,85	1,28	129,27	0,01764261	0,909373673				
rs6655322	0,58	0,36	0,95	0,02422308	0,909373673				
rs12009026	0,27	0,06	1,13	0,02940081	0,909373673	X;HG1462_PATCH	LRCH2;RBMXL3	HGNC Symbol	leucine-rich repeats and calponin homology (CH) domain containing 2 [Source:HGNC Symbol;Acc:29292];RNA binding motif protein, X-linked-like 3 [Source:HGNC
rs5987946	0,24	0,05	1,04	0,02149851	0,909373673	X;HG1462_PATCH	PLS3	HGNC Symbol	plastin 3 [Source:HGNC Symbol;Acc:9091]
rs5987956	0,3	0,12	0,75	0,002513	0,873550665	X;HG1462_PATCH	PLS3	HGNC Symbol	plastin 3 [Source:HGNC Symbol;Acc:9091]
rs2108099	0,11	0,01	0,82	0,00195875	0,873550665	X;HG1462_PATCH	PLS3	HGNC Symbol	plastin 3 [Source:HGNC Symbol;Acc:9091]
rs12396000	0,1	0,01	0,75	0,00115682	0,873550665				
rs5987981	0,12	0,02	0,9	0,00382542	0,873550665				

SNP	OR	lower	upper	p-value	FDR	Chromosome	gene name	gene source	Description
rs12836051	0,49	0,23	1,01	0,03674887	0,909373673				
rs9724449	1,68	1,09	2,57	0,01791262	0,909373673				
rs11091036	0,48	0,26	0,89	0,01399498	0,909373673				
rs5991107	0,57	0,35	0,93	0,02011443	0,909373673	X;HG1463_PATCH	RP11-761E20.1	Clone-based (Vega)	
rs142491398	3,13	1,36	7,18	0,00875917	0,89300867				
rs12847037	1,94	1,13	3,31	0,01683966	0,909373673				
rs73224882	2,24	1,06	4,75	0,04230285	0,909373673				
rs5950534	1,55	1,01	2,39	0,0447372	0,909373673				
rs17231478	2,41	1,15	5,06	0,02402813	0,909373673	X	AGTR2	HGNC Symbol	angiotensin II receptor, type 2 [Source:HGNC Symbol;Acc:338]
rs111766629	2,27	1,13	4,57	0,02556497	0,909373673				
rs6608623	0,17	0,02	1,32	0,02891576	0,909373673				
rs113946157	2,4	1,14	5,02	0,02491529	0,909373673				
rs5910361	1,82	1,11	2,98	0,01788352	0,909373673				
rs62606183	0,47	0,22	1,04	0,03859822	0,909373673				

SNP	OR	lower	upper	p-value	FDR	Chromosome	gene name	gene source	Description
rs6646819	0,41	0,19	0,88	0,01092604	0,907493182				
rs35251144	0,32	0,15	0,71	0,00152291	0,873550665				
rs73217404	7,44	1,19	46,49	0,03324101	0,909373673				
rs73636904	0	0		0,00298654	0,873550665				
rs6645774	0,62	0,39	0,98	0,03618093	0,909373673				
rs4353017	0	0		0,0238422	0,909373673				
rs6645355	0,59	0,36	0,96	0,02711123	0,909373673				
rs2192283	0,61	0,38	1	0,04399277	0,909373673				
rs12008294	0,43	0,21	0,88	0,01291055	0,909373673				
rs17243490	3,85	1,1	13,44	0,04295484	0,909373673				
rs4825643	0,18	0,02	1,37	0,03317461	0,909373673				
rs2018358	0,54	0,31	0,96	0,02892994	0,909373673				
rs3848877	0,42	0,19	0,92	0,0151801	0,909373673	X	SEPT6	HGNC Symbol	septin 6 [Source:HGNC Symbol;Acc:15848]
rs2528711	0,61	0,38	0,97	0,03358971	0,909373673				

SNP	OR	lower	upper	p-value	FDR	Chromosome	gene name	gene source	Description
rs2528727	0	0		0,03605895	0,909373673				
rs5910768	0,49	0,26	0,93	0,01868635	0,909373673	X;HG1442_PATCH	NKAPP1;RP4-755D9.1	HGNC Symbol;Clone-based	NFKB activating protein pseudogene 1 [Source:HGNC Symbol;Acc:26706];
rs5956194	0,58	0,35	0,97	0,03255232	0,909373673	X	NKAPP1	HGNC Symbol	NFKB activating protein pseudogene 1 [Source:HGNC Symbol;Acc:26706]
rs45453700	0,6	0,37	0,97	0,03283926	0,909373673	X;HG1442_PATCH	TMEM255A	HGNC Symbol	transmembrane protein 255A [Source:HGNC Symbol;Acc:26086]
rs5911060	4,37	1,66	11,51	0,00392815	0,873550665				
rs144911663	3,56	1,57	8,06	0,00296174	0,873550665				
rs2110461	0	0		0,04641019	0,909373673				
rs149535880	2,37	1,17	4,77	0,01980312	0,909373673				
rs17258545	0	0		0,00102073	0,873550665				
rs151214516	2,27	1,11	4,64	0,02999135	0,909373673				
rs5911475	0,2	0,03	1,53	0,04699781	0,909373673				
rs1293473	0,47	0,25	0,85	0,00857922	0,89300867				
rs17259204	3,42	1,36	8,59	0,01228806	0,909373673				
rs5911528	0,36	0,16	0,83	0,00757619	0,89300867				

SNP	OR	lower	upper	p-value	FDR	Chromosome	gene name	gene source	Description
rs4825832	0,5	0,28	0,91	0,01572923	0,909373673				
rs4145466	0,4	0,2	0,78	0,00378714	0,873550665				
rs11798709	0,63	0,39	1,01	0,04894578	0,909373673				
rs5911554	0,56	0,32	1	0,03894106	0,909373673	X	GRIA3	HGNC Symbol	glutamate receptor, ionotropic, AMPA 3 [Source:HGNC Symbol;Acc:4573]
rs35506394	0,19	0,03	1,42	0,03546593	0,909373673				
rs3021238	0,61	0,38	0,98	0,03926262	0,909373673				
rs10284179	0	0		0,02568152	0,909373673	X	STAG2	HGNC Symbol	stromal antigen 2 [Source:HGNC Symbol;Acc:11355]
rs2294414	0,6	0,37	0,98	0,03541699	0,909373673	X	TENM1;STAG2	HGNC Symbol	teneurin transmembrane protein 1 [Source:HGNC Symbol;Acc:8117];stromal antigen 2 [Source:HGNC Symbol;Acc:11355]
rs111472283	2,56	1,45	4,53	0,00130086	0,873550665	X	TENM1	HGNC Symbol	teneurin transmembrane protein 1 [Source:HGNC Symbol;Acc:8117]
rs5911935	2,04	1,08	3,86	0,03234874	0,909373673				
rs5911985	2,05	1,02	4,1	0,04830253	0,909373673				
rs66831211	1,92	1,13	3,24	0,017123	0,909373673				
rs212266	0	0		0,01746825	0,909373673				
rs3128747	0	0		0,01342561	0,909373673				

SNP	OR	lower	upper	p-value	FDR	Chromosome	gene name	gene source	Description
rs56340315	3,78	1,2	11,89	0,02869784	0,909373673				
rs5933485	1,86	1,02	3,39	0,04527839	0,909373673				
rs62609661	0,55	0,3	1	0,04060657	0,909373673				
rs144080138	0,25	0,07	0,81	0,0049877	0,873550665	X	RP1-30E17.2	Clone-based (Vega)	
rs1923842	2,47	1,27	4,79	0,0091358	0,89300867				
rs11795787	1,8	1,12	2,89	0,01641916	0,909373673	X	XPNPEP2	HGNC Symbol	X-prolyl aminopeptidase (aminopeptidase P) 2, membrane-bound [Source:HGNC Symbol;Acc:12823]
rs73633939	0,34	0,1	1,13	0,04285754	0,909373673				
rs144741515	2,04	1,03	4,06	0,04805912	0,909373673	X	RBMX2	HGNC Symbol	RNA binding motif protein, X-linked 2 [Source:HGNC Symbol;Acc:24282]
rs17305432	0,16	0,02	1,2	0,01880892	0,909373673				
rs73233186	2,41	1,23	4,71	0,01276904	0,909373673	X	IGSF1	HGNC Symbol	immunoglobulin superfamily, member 1 [Source:HGNC Symbol;Acc:5948]
rs73235176	2,82	1,33	5,97	0,0088406	0,89300867				
rs4142509	1,74	1,09	2,77	0,02072535	0,909373673	X	MST4	UniProtKB Gene Name	Serine/threonine-protein kinase MST4 [Source:UniProtKB/Swiss-Prot;Acc:Q9P289]
rs5977623	1,69	1,07	2,67	0,0266556	0,909373673	X	FRMD7	HGNC Symbol	FERM domain containing 7 [Source:HGNC Symbol;Acc:8079]
rs5977658	3,06	1,24	7,54	0,01807511	0,909373673	X	RAP2C	HGNC Symbol	RAP2C, member of RAS oncogene family [Source:HGNC Symbol;Acc:21165]

SNP	OR	lower	upper	p-value	FDR	Chromosome	gene name	gene source	Description
rs144412136	0	0		0,0160767	0,909373673	X	RAP2C-AS1	HGNC Symbol	RAP2C antisense RNA 1 [Source:HGNC Symbol;Acc:40957]
rs6637988	3,9	1,41	10,8	0,01126064	0,909373673	X	RAP2C-AS1	HGNC Symbol	RAP2C antisense RNA 1 [Source:HGNC Symbol;Acc:40957]
rs62617185	0,38	0,13	1,07	0,03592918	0,909373673	X	MBNL3	HGNC Symbol	muscleblind-like splicing regulator 3 [Source:HGNC Symbol;Acc:20564]
rs17324091	0,4	0,17	0,94	0,01763446	0,909373673	X	HS6ST2	HGNC Symbol	heparan sulfate 6-O-sulfotransferase 2 [Source:HGNC Symbol;Acc:19133]
rs17251211	2,63	1,05	6,59	0,04612777	0,909373673	X	HS6ST2	HGNC Symbol	heparan sulfate 6-O-sulfotransferase 2 [Source:HGNC Symbol;Acc:19133]
rs73638579	0,4	0,16	1,01	0,02640314	0,909373673	X	HS6ST2	HGNC Symbol	heparan sulfate 6-O-sulfotransferase 2 [Source:HGNC Symbol;Acc:19133]
rs144346774	0,18	0,02	1,4	0,03621444	0,909373673	X	HS6ST2	HGNC Symbol	heparan sulfate 6-O-sulfotransferase 2 [Source:HGNC Symbol;Acc:19133]
rs5977769	0,45	0,19	1,07	0,04620173	0,909373673	X	HS6ST2	HGNC Symbol	heparan sulfate 6-O-sulfotransferase 2 [Source:HGNC Symbol;Acc:19133]
rs147826244	3,02	1,27	7,17	0,01513215	0,909373673	X	GPC3	HGNC Symbol	glypican 3 [Source:HGNC Symbol;Acc:4451]
rs138697029	4	1,15	13,87	0,03686221	0,909373673	X	AF003529.2	Clone-based (Vega)	
rs6638162	2,53	1,52	4,19	0,00024109	0,873550665				
rs12850804	2,57	1,36	4,88	0,00416164	0,873550665				
rs73241337	0,15	0,02	1,12	0,01401572	0,909373673	X	PHF6	HGNC Symbol	PHD finger protein 6 [Source:HGNC Symbol;Acc:18145]
rs12558462	5,98	1,88	19	0,00310704	0,873550665				

SNP	OR	lower	upper	p-value	FDR	Chromosome	gene name	gene source	Description
rs2503984	0	0		0,01746874	0,909373673	X	MIR503HG	HGNC Symbol	MIR503 host gene (non-protein coding) [Source:HGNC Symbol;Acc:28258]
rs41300299	2,95	1,08	8,11	0,04431557	0,909373673	X	PLAC1;RP11-308B5.2	HGNC Symbol;Clone-based	placenta-specific 1 [Source:HGNC Symbol;Acc:9044];
rs17278226	0	0		0,04605879	0,909373673				
rs149875145	0	0		0,02791951	0,909373673	X;HG1443_HG1444_PATCH	RP11-85L21.4	Clone-based (Vega)	
rs5975507	1,63	1,04	2,55	0,03016631	0,909373673				
rs17283962	0,24	0,06	1	0,01365358	0,909373673				
rs17330752	0,36	0,15	0,85	0,00788168	0,89300867	X;HG1443_HG1444_PATCH	DDX26B	HGNC Symbol	DEAD/H (Asp-Glu-Ala-Asp/His) box polypeptide 26B [Source:HGNC Symbol;Acc:27334]
rs139701979	3,45	1,36	8,76	0,01232704	0,909373673	X	RP11-432N13.4	Clone-based (Vega)	
rs140864304	2,56	1,13	5,78	0,02973859	0,909373673				
rs56165875	0,25	0,06	1,09	0,02640592	0,909373673				
rs17330843	0	0		0,02521225	0,909373673	X	VGLL1	HGNC Symbol	vestigial like 1 (Drosophila) [Source:HGNC Symbol;Acc:20985]
rs6633864	1,84	1,05	3,21	0,0344513	0,909373673				
rs10521782	1,78	1,08	2,92	0,02440592	0,909373673				
rs4269687	0,2	0,03	1,46	0,03596273	0,909373673				

SNP	OR	lower	upper	p-value	FDR	Chromosome	gene name	gene source	Description
rs7886303	2	1,17	3,41	0,01145812	0,909373673				
rs1408308	1,63	1,02	2,59	0,03897684	0,909373673				
rs1366934	1,86	1,17	2,97	0,00814921	0,89300867				
rs34207960	0,17	0,02	1,27	0,02406729	0,909373673				
rs4829934	1,75	1,1	2,78	0,0158916	0,909373673				
rs1929364	0,61	0,37	1,01	0,04938643	0,909373673				
rs6635599	1,79	1,14	2,82	0,01137208	0,909373673				
rs73236854	1,85	1,16	2,94	0,00951073	0,900996292				
rs1994451	0,59	0,38	0,91	0,01618668	0,909373673				
rs5931380	1,76	1,11	2,77	0,01591608	0,909373673				
rs147018514	0,19	0,02	1,4	0,03278399	0,909373673	X	FGF13	HGNC Symbol	fibroblast growth factor 13 [Source:HGNC Symbol;Acc:3670]
rs73241070	0,33	0,1	1,12	0,04408374	0,909373673	X	FGF13	HGNC Symbol	fibroblast growth factor 13 [Source:HGNC Symbol;Acc:3670]
rs73241079	0,15	0,02	1,12	0,01364028	0,909373673	X	FGF13	HGNC Symbol	fibroblast growth factor 13 [Source:HGNC Symbol;Acc:3670]
rs141792138	0,17	0,02	1,28	0,0255522	0,909373673	X	FGF13	HGNC Symbol	fibroblast growth factor 13 [Source:HGNC Symbol;Acc:3670]

SNP	OR	lower	upper	p-value	FDR	Chromosome	gene name	gene source	Description
rs479265	1,91	1,06	3,45	0,03334988	0,909373673	X	FGF13	HGNC Symbol	fibroblast growth factor 13 [Source:HGNC Symbol;Acc:3670]
rs73243310	0,11	0,01	0,83	0,00239759	0,873550665	X	FGF13	HGNC Symbol	fibroblast growth factor 13 [Source:HGNC Symbol;Acc:3670]
rs72616266	0	0		0,01774529	0,909373673	X	FGF13	HGNC Symbol	fibroblast growth factor 13 [Source:HGNC Symbol;Acc:3670]
rs12387343	0,34	0,17	0,71	0,00149995	0,873550665	X	FGF13	HGNC Symbol	fibroblast growth factor 13 [Source:HGNC Symbol;Acc:3670]
rs9988241	0,41	0,16	1,08	0,04559123	0,909373673	X	FGF13	HGNC Symbol	fibroblast growth factor 13 [Source:HGNC Symbol;Acc:3670]
rs392959	0,25	0,06	1,08	0,02495187	0,909373673	X	F9	HGNC Symbol	coagulation factor IX [Source:HGNC Symbol;Acc:3551]
rs61751333	0	0		0,03560466	0,909373673	X	MCF2	HGNC Symbol	MCF.2 cell line derived transforming sequence [Source:HGNC Symbol;Acc:6940]
rs73245304	2,54	1,4	4,61	0,00231092	0,873550665				
rs6635942	1,75	1,05	2,92	0,03124034	0,909373673				
rs5955267	2,13	1,21	3,76	0,00934298	0,900996292				
rs4354481	2,26	1,29	3,99	0,00509889	0,873550665				
rs73585731	2,26	1,29	3,99	0,00509889	0,873550665				
rs2496271	1,67	1,02	2,75	0,04347509	0,909373673				
rs1541375	1,7	1,08	2,67	0,02067792	0,909373673				

SNP	OR	lower	upper	p-value	FDR	Chromosome	gene name	gene source	Description
rs6636011	1,77	1,14	2,76	0,00967447	0,900996292				
rs17322765	3,36	1,09	10,36	0,04361309	0,909373673				
rs7061468	1,79	1,03	3,11	0,04418874	0,909373673				
rs1407833	1,76	1,02	3,04	0,04511002	0,909373673				
rs143006078	0	0		0,02916963	0,909373673				
rs6528734	0,6	0,37	0,99	0,03754848	0,909373673				
rs11796840	6,7	2,35	19,1	0,00050879	0,873550665				
rs56239823	1,58	1,01	2,47	0,04731867	0,909373673	X	LINC00632	HGNC Symbol	long intergenic non-protein coding RNA 632 [Source:HGNC Symbol;Acc:27865]
rs41299075	0	0		0,00891934	0,89300867	X	CDR1	HGNC Symbol	cerebellar degeneration-related protein 1, 34kDa [Source:HGNC Symbol;Acc:1798]
rs7886564	0	0		0,03440215	0,909373673				
rs6634214	2,35	1,16	4,72	0,02070896	0,909373673	X;HG1453_PATCH	RP11-298A8.2	Clone-based (Vega)	
rs5907910	1,63	1,04	2,55	0,03380637	0,909373673				
rs5907150	0,54	0,31	0,92	0,01827392	0,909373673				
rs6528868	1,98	1,16	3,36	0,01332478	0,909373673				

SNP	OR	lower	upper	p-value	FDR	Chromosome	gene name	gene source	Description
rs5907949	1,88	1,2	2,96	0,00610295	0,884530262				
rs7063314	2,16	1,37	3,41	0,00070919	0,873550665				
rs2239835	1,86	1,09	3,19	0,02487957	0,909373673	X	RP1-171K16.5;SPANXA2-OT1	Clone-based (Vega)	;SPANXA2 overlapping transcript 1 (non-protein coding) [Source:HGNC Symbol;Acc:31683]
rs6636421	1,76	1,03	3	0,04320881	0,909373673	X	SPANXA2-OT1	HGNC Symbol	SPANXA2 overlapping transcript 1 (non-protein coding) [Source:HGNC Symbol;Acc:31683]
rs6636440	1,86	1,11	3,1	0,01849215	0,909373673				
rs55763214	0	0		0,00575561	0,884530262				
rs17257697	0,15	0,02	1,17	0,01784323	0,909373673				
rs5953652	2,22	1,16	4,28	0,01958644	0,909373673				
rs7053223	0	0		0,0441342	0,909373673				
rs5954551	0	0		0,00378417	0,873550665				
rs12710619	2,17	1,29	3,64	0,00330406	0,873550665				
rs56254936	1,7	1,01	2,86	0,04696838	0,909373673				
rs146573698	2,07	1,1	3,87	0,02683935	0,909373673				
rs138880414	0	0		0,03867855	0,909373673				

SNP	OR	lower	upper	p-value	FDR	Chromosome	gene name	gene source	Description
rs2206216	0,58	0,33	1,02	0,0489191	0,909373673				
rs35739866	0,52	0,27	0,98	0,03436662	0,909373673				
rs75201119	0,14	0,02	1,07	0,00952479	0,900996292				
rs138291813	0	0		0,00584836	0,884530262				
rs6529189	0,23	0,05	0,97	0,01470904	0,909373673				
rs6649631	0,58	0,34	0,98	0,03287582	0,909373673				
rs12559795	0,16	0,02	1,24	0,02169296	0,909373673				
rs12013685	0	0		0,02326513	0,909373673				
rs7049661	0,43	0,25	0,73	0,00094701	0,873550665				
rs11797855	0,5	0,28	0,88	0,01012773	0,900996292				
rs186982583	0,18	0,02	1,37	0,03363978	0,909373673				
rs6627094	5,79	1,49	22,57	0,01314658	0,909373673				
rs143116064	2,63	1,13	6,15	0,0318054	0,909373673				
rs1781502	0,56	0,32	0,96	0,0267678	0,909373673				

SNP	OR	lower	upper	p-value	FDR	Chromosome	gene name	gene source	Description
rs1174503	0,65	0,42	1	0,04601867	0,909373673				
rs2748588	2,08	1,11	3,9	0,02609739	0,909373673	X;HG1459_PATCH	SLITRK2	HGNC Symbol	SLIT and NTRK-like family, member 2 [Source:HGNC Symbol;Acc:13449]
rs73237986	0,28	0,06	1,22	0,04621264	0,909373673				
rs112561283	2,57	1,29	5,1	0,00850248	0,89300867				
rs78953490	2,89	1,26	6,62	0,01567139	0,909373673				
rs2891838	2,92	1,15	7,41	0,02906861	0,909373673				
rs5951853	0,34	0,1	1,15	0,04697035	0,909373673				
rs5904872	0,64	0,4	1	0,04795864	0,909373673				
rs6626213	0,61	0,37	1	0,04418851	0,909373673				
rs12839843	1,9	1,18	3,04	0,00844134	0,89300867				
rs5904936	1,58	1,02	2,43	0,03727888	0,909373673				
rs16994035	1,88	1,08	3,27	0,02591383	0,909373673				
rs2392722	2,1	1,27	3,48	0,00366566	0,873550665				
rs111830525	1,71	1,04	2,8	0,03534292	0,909373673				

SNP	OR	lower	upper	p-value	FDR	Chromosome	gene name	gene source	Description
rs2954104	0,6	0,36	0,99	0,04057103	0,909373673				
rs2742911	0	0		0,04239947	0,909373673				
rs111900525	0,44	0,18	1,04	0,03772728	0,909373673				
rs45631658	0,52	0,28	0,99	0,03422213	0,909373673				
rs5904675	2,64	1,17	5,98	0,02464216	0,909373673				
rs2109881	0,63	0,4	0,98	0,03620614	0,909373673				
rs5936201	2,95	1,38	6,27	0,0066277	0,89300867				
rs1582301	0,57	0,36	0,89	0,01197414	0,909373673				
rs6641219	0,6	0,39	0,92	0,01801169	0,909373673				
rs241132	0	0		0,02070927	0,909373673	X;HG1459_PATCH	AFF2	HGNC Symbol	AF4/FMR2 family, member 2 [Source:HGNC Symbol;Acc:3776]
rs1265414	0	0		0,00786884	0,89300867	X;HG1459_PATCH	AFF2	HGNC Symbol	AF4/FMR2 family, member 2 [Source:HGNC Symbol;Acc:3776]
rs55711809	0	0		0,02523645	0,909373673	X;HG1459_PATCH	AFF2	HGNC Symbol	AF4/FMR2 family, member 2 [Source:HGNC Symbol;Acc:3776]
rs73249456	0	0		0,03041107	0,909373673				
rs148101966	2,75	1,13	6,74	0,0332423	0,909373673				

SNP	OR	lower	upper	p-value	FDR	Chromosome	gene name	gene source	Description
rs73244660	2,35	1,1	5,01	0,03161405	0,909373673				
rs56288522	2,69	1,32	5,48	0,0081554	0,89300867	X;HG1459_PATCH	LINC00894	HGNC Symbol	long intergenic non-protein coding RNA 894 [Source:HGNC Symbol;Acc:48579]
rs3900055	0,35	0,11	1,15	0,04370113	0,909373673	X;HG1459_PATCH	LINC00894	HGNC Symbol	long intergenic non-protein coding RNA 894 [Source:HGNC Symbol;Acc:48579]
rs9284560	1,59	1,01	2,51	0,04649674	0,909373673				
rs693913	1,87	1,2	2,93	0,00509283	0,873550665	X	MAMLD1	HGNC Symbol	mastermind-like domain containing 1 [Source:HGNC Symbol;Acc:2568]
rs1983610	0,63	0,41	0,98	0,03534995	0,909373673	X	MAMLD1	HGNC Symbol	mastermind-like domain containing 1 [Source:HGNC Symbol;Acc:2568]
rs12013750	1,9	1,07	3,38	0,03226335	0,909373673	X	MAMLD1	HGNC Symbol	mastermind-like domain containing 1 [Source:HGNC Symbol;Acc:2568]
rs73250543	0,34	0,13	0,88	0,01065971	0,900996292	X	MAMLD1	HGNC Symbol	mastermind-like domain containing 1 [Source:HGNC Symbol;Acc:2568]
rs7065239	0,61	0,37	0,99	0,03907852	0,909373673				
rs12557844	1,61	1,01	2,56	0,04045461	0,909373673				
rs57102007	0	0		0,03132216	0,909373673	X;HG1459_PATCH	MTM1	HGNC Symbol	myotubularin 1 [Source:HGNC Symbol;Acc:7448]
rs6653501	0	0		0,03524498	0,909373673	X;HG1459_PATCH	CD99L2	HGNC Symbol	CD99 molecule-like 2 [Source:HGNC Symbol;Acc:18237]
rs6627335	0	0		0,04461853	0,909373673				
rs1483959	0,5	0,26	0,97	0,02900947	0,909373673				

SNP	OR	lower	upper	p-value	FDR	Chromosome	gene name	gene source	Description
rs148304180	0,38	0,14	1,01	0,03215711	0,909373673				
rs909084	0,1	0,01	0,72	0,00101212	0,873550665				
rs909086	0,21	0,07	0,59	0,00034885	0,873550665				
rs237397	0,54	0,33	0,88	0,01051504	0,900996292				
rs7057942	1,83	1,13	2,96	0,01477812	0,909373673				
rs5924858	1,68	1,04	2,73	0,03600962	0,909373673				
rs7879350	0,2	0,03	1,5	0,04411644	0,909373673				
rs78972460	0,21	0,05	0,89	0,00862378	0,89300867				
rs12392387	1,59	1	2,54	0,04768111	0,909373673	X	PASD1;RP11-45D17.1	HGNC Symbol;Clone-based	PAS domain containing 1 [Source:HGNC Symbol;Acc:20686];
rs7471911	0,27	0,09	0,78	0,00418137	0,873550665				
rs12008689	0,48	0,24	0,95	0,02400726	0,909373673				
rs741726	2,07	1,3	3,32	0,00245993	0,873550665				
rs5970118	1,79	1,15	2,8	0,00854028	0,89300867				
rs720378	0	0		0,0088894	0,89300867				

SNP	OR	lower	upper	p-value	FDR	Chromosome	gene name	gene source	Description
rs5925043	1,64	1,05	2,56	0,02742541	0,909373673				
rs5925050	0,43	0,19	1	0,03109044	0,909373673				
rs5969828	0,48	0,23	1	0,03288117	0,909373673				
rs12387812	0	0		0,04245311	0,909373673				
rs113954542	0,44	0,19	1,01	0,03625826	0,909373673				
rs149642212	2,65	1,34	5,23	0,00641812	0,884530262				
rs2051529	1,63	1,03	2,57	0,03420907	0,909373673				
rs145754054	0,16	0,02	1,22	0,02081149	0,909373673				
rs5970181	0,49	0,25	0,95	0,02710248	0,909373673				
rs5925105	1,93	1,2	3,11	0,00548601	0,884530262				
rs5970194	0,43	0,21	0,9	0,01659364	0,909373673				
rs145701879	3,23	1,47	7,08	0,00479482	0,873550665	X	GABRA3;RP 11-329E24.6	HGNC Symbol;Clone-based	gamma-aminobutyric acid (GABA) A receptor, alpha 3 [Source:HGNC Symbol;Acc:4077];
rs5970335	0,62	0,39	0,98	0,0366248	0,909373673				
rs5970440	1,77	1,13	2,79	0,01174792	0,909373673				

SNP	OR	lower	upper	p-value	FDR	Chromosome	gene name	gene source	Description
rs11094626	0,58	0,34	0,97	0,03152293	0,909373673				
rs3213466	0,57	0,34	0,94	0,02268791	0,909373673	X;HG1497_PATCH	ZNF275	HGNC Symbol	zinc finger protein 275 [Source:HGNC Symbol;Acc:13069]
rs62596388	0,55	0,33	0,92	0,0176917	0,909373673				
rs12400823	2,15	1,33	3,47	0,00183712	0,873550665				
rs2071028	0,22	0,05	0,95	0,0137257	0,909373673	X;HG1497_PATCH	SLC6A8	HGNC Symbol	solute carrier family 6 (neurotransmitter transporter), member 8 [Source:HGNC Symbol;Acc:11055]
rs3027924	2,34	1,16	4,7	0,02070695	0,909373673	X;HG1497_PATCH	MECP2	HGNC Symbol	methyl CpG binding protein 2 (Rett syndrome) [Source:HGNC Symbol;Acc:6990]
rs4898495	0	0		0,00450536	0,873550665	X	ATP6AP1	HGNC Symbol	ATPase, H+ transporting, lysosomal accessory protein 1 [Source:HGNC Symbol;Acc:868]
rs113038688	2,48	1,03	5,94	0,04937235	0,909373673				
rs6642320	1,61	1,04	2,49	0,03154808	0,909373673	X	SPRY3	HGNC Symbol	sprouty homolog 3 (Drosophila) [Source:HGNC Symbol;Acc:11271]
rs28729587	2,88	1,5	5,54	0,00183643	0,873550665	X	SPRY3	HGNC Symbol	sprouty homolog 3 (Drosophila) [Source:HGNC Symbol;Acc:11271]
rs28425172	1,64	1,04	2,57	0,02985599	0,909373673				
rs34445725	0,55	0,3	0,98	0,03382232	0,909373673				
rs5940638	0,61	0,39	0,96	0,02721789	0,909373673				
rs3093493	2,13	1,17	3,88	0,01539465	0,909373673	X	IL9R	HGNC Symbol	interleukin 9 receptor [Source:HGNC Symbol;Acc:6030]

SNP	OR	lower	upper	p-value	FDR	Chromosome	gene name	gene source	Description
rs6649919	1,69	1,04	2,75	0,03672207	0,909373673				
rs34304242	0,51	0,26	1	0,03963662	0,909373673				
rs6644961	0,53	0,28	1,02	0,04559925	0,909373673				
rs34264278	0,44	0,21	0,94	0,02363681	0,909373673				
rs28429757	0,3	0,12	0,77	0,00366348	0,873550665				
rs28605266	0,44	0,21	0,95	0,02471075	0,909373673	X	PLCXD1	HGNC Symbol	phosphatidylinositol-specific phospholipase C, X domain containing 1 [Source:HGNC Symbol;Acc:23148]
rs6645102	0,59	0,34	1	0,04474803	0,909373673	X	PPP2R3B	HGNC Symbol	protein phosphatase 2, regulatory subunit B'', beta [Source:HGNC Symbol;Acc:13417]
rs113150534	1,74	1,05	2,88	0,03362492	0,909373673				
rs5991299	0,58	0,37	0,93	0,02093925	0,909373673				
rs28404660	2,26	1,03	4,96	0,04908262	0,909373673				
rs28667393	0,63	0,4	0,98	0,03837838	0,909373673				
rs4472693	0,49	0,26	0,94	0,0217044	0,909373673				
rs2037897	0,6	0,37	0,98	0,0357689	0,909373673				
rs4911918	2,02	1,12	3,67	0,02103994	0,909373673				

SNP	OR	lower	upper	p-value	FDR	Chromosome	gene name	gene source	Description
rs2027987	0,63	0,4	1	0,04600239	0,909373673				
rs4911921	1,71	1,05	2,78	0,03028564	0,909373673				
rs73178390	6,07	1,74	21,17	0,00566329	0,884530262				
rs5988437	0,48	0,28	0,81	0,00374406	0,873550665				
rs17148729	0,45	0,27	0,75	0,00115935	0,873550665				
rs5946343	0,4	0,17	0,91	0,0160336	0,909373673				
rs5946570	1,7	1,09	2,67	0,01848556	0,909373673				
rs73188058	1,69	1,06	2,68	0,02607297	0,909373673	X;HG480_HG481_PATCH	RP11-309M23.1	Clone-based (Vega)	
rs7878830	0,6	0,37	0,97	0,03218922	0,909373673				
rs73191918	2,21	1,04	4,71	0,04707526	0,909373673				
rs73191974	1,98	1,06	3,69	0,0364688	0,909373673				
rs66923305	0,6	0,39	0,94	0,02251297	0,909373673				
rs28377393	0,25	0,08	0,8	0,00337528	0,873550665				
rs34745620	1,7	1,09	2,66	0,01978596	0,909373673				

SNP	OR	lower	upper	p-value	FDR	Chromosome	gene name	gene source	Description
rs28460331	0,48	0,23	1,02	0,04226422	0,909373673				
rs73175748	1,62	1,04	2,54	0,03539857	0,909373673				
rs28736568	0,37	0,13	1,06	0,03536103	0,909373673	X	CSF2RA	HGNC Symbol	colony stimulating factor 2 receptor, alpha, low-affinity (granulocyte-macrophage) [Source:HGNC Symbol;Acc:2435]
rs35012795	0	0		0,03330494	0,909373673				
rs73624872	2,32	1,25	4,28	0,0087784	0,89300867				
rs28540518	1,6	1,02	2,5	0,040932	0,909373673				
rs113486847	2,17	1,28	3,66	0,00472013	0,873550665	X	LINC00106	HGNC Symbol	long intergenic non-protein coding RNA 106 [Source:HGNC Symbol;Acc:31843]
rs112670469	2,08	1,23	3,51	0,00746457	0,89300867	X	LINC00106	HGNC Symbol	long intergenic non-protein coding RNA 106 [Source:HGNC Symbol;Acc:31843]
rs113370738	2,24	1,26	3,98	0,00747507	0,89300867				
rs112133773	2,12	1,16	3,91	0,01843207	0,909373673	X	ASMTL	HGNC Symbol	acetylserotonin O-methyltransferase-like [Source:HGNC Symbol;Acc:751]
rs73182962	2,52	1,11	5,7	0,03263225	0,909373673				
rs28675287	0,42	0,16	1,07	0,04182156	0,909373673	X	ASMT	HGNC Symbol	acetylserotonin O-methyltransferase [Source:HGNC Symbol;Acc:750]
rs1470876	1,85	1,03	3,35	0,04362238	0,909373673				
rs6588867	0,56	0,34	0,92	0,01738177	0,909373673				

SNP	OR	lower	upper	p-value	FDR	Chromosome	gene name	gene source	Description
rs5949181	1,76	1,11	2,79	0,016251	0,909373673				
rs28405659	2,14	1,09	4,22	0,03265044	0,909373673				
rs28418610	1,91	1,05	3,47	0,03989663	0,909373673	X	DHRSX	HGNC Symbol	dehydrogenase/reductase (SDR family) X-linked [Source:HGNC Symbol;Acc:18399]
rs1044307	1,58	1,02	2,44	0,03776573	0,909373673	X	DHRSX;ZBED1	HGNC Symbol	dehydrogenase/reductase (SDR family) X-linked [Source:HGNC Symbol;Acc:18399];zinc finger, BED-type containing 1 [Source:HGNC Symbol;Acc:447]
rs951323	0,58	0,35	0,94	0,02520678	0,909373673				
rs35603448	0,64	0,41	1	0,04563342	0,909373673				
rs5939089	1,79	1,14	2,8	0,00974844	0,900996292				
rs6567636	0,53	0,31	0,91	0,01605454	0,909373673	X	CD99P1	HGNC Symbol	CD99 molecule pseudogene 1 [Source:HGNC Symbol;Acc:7083]
rs3813164	0,62	0,39	0,99	0,04309728	0,909373673				
rs28758440	0,19	0,03	1,42	0,03514962	0,909373673	X	XG	HGNC Symbol	Xg blood group [Source:HGNC Symbol;Acc:12806]

aEARR: aggressive external apical root resorption
